# Supplementary material for: A general synthesis of azetidines by copper-catalysed photoinduced anti-Baldwin radical cyclization of ynamides
Source: Nat Commun. 2022 Jan 28;13:560. doi: 10.1038/s41467-022-28098-x (PMC8799647; doi:10.1038/s41467-022-28098-x)
Supplement: Supplementary file 2 — Supplementary Information [file 41467_2022_28098_MOESM2_ESM.pdf]

Supplementary Information

A General Synthesis of Azetidines by Copper-Catalyzed Photoinduced anti-Baldwin Radical Cyclization of Ynamides

Clément Jacob[1,2], Hajar Baguia[1], Amaury Dubart[1], Samuel Oger[1], Pierre Thilmany[1], Jérôme Beaudelot[1,3], Christopher Deldaele[1], Stefano Peruško[1,2], Yohann Landrain[1], Bastien Michelet[1], Samuel Neale[4], Eugénie Romero[1], Cécile Moucheron[3]*, Veronique Van Speybroeck[4]*, Cédric Theunissen[1]* and Gwilherm Evano[1]*

[1] *Laboratoire de Chimie Organique, Service de Chimie et PhysicoChimie Organiques, Université libre de Bruxelles (ULB), Avenue F. D. Roosevelt 50, CP160/06, 1050 Brussels, Belgium.*

[2] *Organic Synthesis Division, Department of Chemistry, University of Antwerp, Groenenborgerlaan 171, 2020 Antwerp, Belgium.*

[3] *Laboratoire de Chimie Organique et Photochimie, Service de Chimie et PhysicoChimie Organiques, Université libre de Bruxelles (ULB), Avenue F. D. Roosevelt 50, CP160/08, 1050 Brussels, Belgium.*

[4] *Center for Molecular Modeling, Ghent University, Tech Lane Ghent Science Park Campus A, Technologiepark, 46, 9052 Zwijnaarde, Belgium.*

1. Supplementary Methods 3

2. Supplementary Discussion 5

2.1. Experimental Procedures and Characterization Data:
Synthesis of [Cu(bcp)DPEphos]PF_6 5

2.2. Experimental Procedures and Characterization Data:
Synthesis of Unreported Starting Materials **SA**-**SAA** 6

2.3. Experimental Procedures and Characterization Data:
Synthesis of *O*-Protected-Ynamides **S1a**-**S1aj** 23

2.4. Experimental Procedures and Characterization Data:
Synthesis of Hydroxyethyl-Ynamides **S2a**-**S2aj** 44

2.5. Experimental Procedures and Characterization Data:
Synthesis of Methanesulfonyloxyethyl-Ynamides **S3v**-**S3ah** 64

2.6. Experimental Procedures and Characterization Data:
Synthesis of Iodoethyl-Ynamides **1a**-**1aj** 68

2.7. Experimental Procedures and Characterization Data:
Synthesis of Chloroethyl-Ynamide **1a**$_{\mathbf{Cl}}$ 88

2.8. Experimental Procedures and Characterization Data:
Synthesis of Compound **7**. 89
2.9. Experimental Procedures and Characterization Data:
Copper-Catalyzed Photoredox Cyclization of Ynamides to Azetidines **2a**-**2aj** 91
2.10. Experimental Procedures and Characterization Data:
Hydrogenation of Azetidines 120
2.11. Experimental Procedures and Characterization Data:
Additional Diversification of Azetidines 127
2.12. Experimental Procedures and Characterization Data:
Isomerization of Azetidines 131
2.13. Experimental Procedures and Characterization Data:
Chemical Correlation for the Confirmation of the Structure of Azetidine **2b**......... 136
2.14. ^{1}H and ^{13}C NMR Spectra 137
2.15. NOESY Experiments on Azetidines **(*Z*)-2a**, **(*E*)-2a**, **2b**, **15f**, **15h** and **15i** 353
2.16. Computational Studies 360

3. Supplementary References 388

1. Supplementary Methods.

All reactions were carried out in oven-dried glassware under an argon atmosphere employing standard techniques in handling air-sensitive materials.

All solvents were reagent grade. Acetonitrile, dichloromethane and 1,2-dichloroethane were freshly distilled from calcium hydride under argon and acetonitrile was further degassed by using the freeze-pump-thaw method. Tetrahydrofuran was freshly distilled from sodium/benzophenone under argon.

N,*N*-Diisopropylethylamine ((*i*-Pr)$_2$NEt) was distilled from KOH under argon and stored away from light. Copper(I) iodide (99.999% purity) was purchased from Aldrich and used as supplied.

Reactions were magnetically stirred and monitored by thin layer chromatography using Merck-Kiesegel 60F$_{254}$ plates. Flash chromatography was performed with silica gel 60 (particle size 35-70 µm) supplied by Merck. Yields refer to chromatographically and spectroscopically pure compounds unless otherwise stated. Photoinduced copper-catalyzed reactions were performed with commercially available blue LED strips or in a Luzchem CCP-4V photoreactor using 420 nm light tubes supplied by Luzchem or in a parallel photoreactor (EvoluChem^{TM} PhotoRedOx Box) supplied by Interchim using a blue Kessil LED lamp (H150-Blue, 440 nm, 34 W) as the source of light. Ozonolysis was performed using a Triogen® system supplied by Degrémont Technologies (Suez).

Proton NMR spectra were recorded using an internal deuterium lock at ambient temperature on Bruker 300 MHz, Jeol 400 MHz, Jeol 600 MHz or Varian 400 MHz spectrometers. Internal reference of δ_H 7.26 was used for $CDCl_3$, and δ_H 2.50 was used for DMSO-d6. Data are presented as follows: chemical shift (in ppm on the δ scale relative to δ_{TMS} = 0), multiplicity (*s = singlet, d = doublet, t = triplet, q = quartet, quint. = quintuplet, sext. = sextuplet, sept. = septuplet, oct. = octuplet, m = multiplet, br. = broad, app. = apparent*), coupling constant (*J*/Hz) and integration. Resonances that are either partially or fully obscured are denoted obscured (obs.). Carbon-13 NMR spectra were recorded at 75, 100 or 150 MHz using $CDCl_3$ (δ_C 77.16) or DMSO-d6 (δ_C 39.52) as internal references. Fluorine-19 NMR spectra were recorded at 376 MHz using α,α,α-trifluorotoluene (δ_F -63.24) as internal reference.

Absorption spetra were recorded on a UV-Visible Perkin Elmer Lambda 35 spectrophotometer. Emission spectra were recorded on a Shimadzu RF-5301PC

spectrofluorimeter equipped with a 150 W Xenon lamp as excitation source and a Hamamatsu R928 phototube as detector. Corrections for the emission spectra were achieved by measuring the spectrum of a calibrated tungsten-halogen Edinburgh Analytical Instrument lamp of 10 W (200 to 600 nm).

Optical rotations were recorded on an Anton Paar MCP-100 automatic polarimeter at 589 nm and reported as follows: $[\alpha]_{\mathrm{D}}^{\mathrm{T}}$, concentration (*c* in g/100 mL), and solvent. Melting points were recorded on a Stuart Scientific Analogue SMP11. High-resolution mass spectra in positive mode were recorded using a 6520 series quadrupole time-of-flight (Q-TOF) mass spectrometer (Agilent) fitted with a multimode ion source.

Irradiation systems

Three different irradiation systems have been used for the copper-catalyzed photoredox cyclization of iodoethyl-ynamides into the corresponding azetidines: *a)* commercially available blue LED strips, *b)* a photoreactor (Luzchem CCP-4V) using 420 nm light tubes, and *c)* a parallel photoreactor (EvoluChem^{TM} PhotoRedOx Box) using a blue Kessil LED lamp (H150-Blue, 440 nm, 34 W).

The nature of the irradiation system only has a little impact on the outcome of the cyclization of iodoethyl-ynamides into the corresponding azetidines and all three systems can therefore be used, the last system (EvoluChem^{TM} PhotoRedOx Box) being a little less efficient both in terms of yield and stereoselectivity.

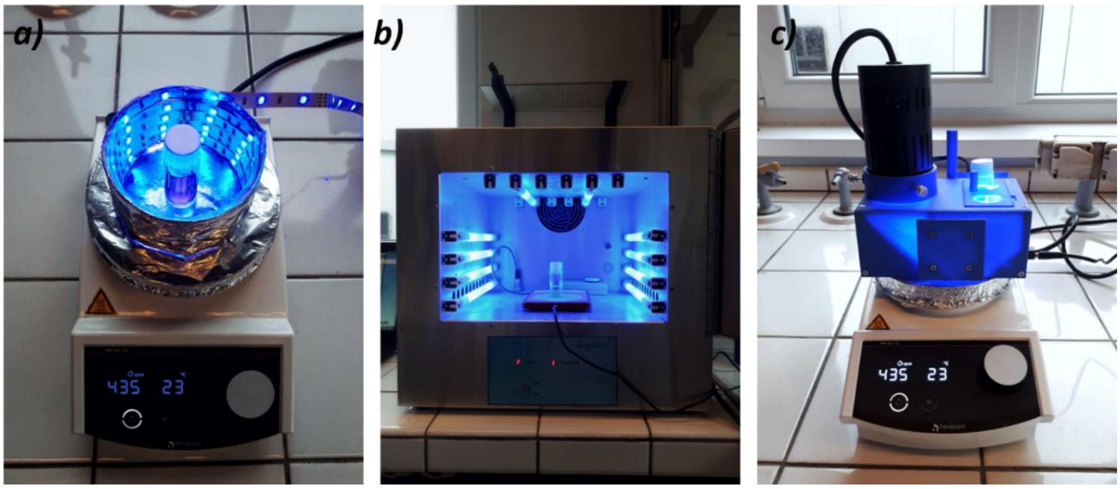

Supplementary Fig 1. a Commercially available blue LEDs strips. **b** Photoreactor (Luzchem CCP-4V) using 420 nm light tubes. **c** Parallel photoreactor (EvoluChem^{TM} PhotoRedOx Box) using a blue Kessil lamp (H150-Blue, 440 nm, 34W)..

2. Supplementary Discussion.

2.1. Experimental Procedure and Characterization Data: Synthesis of [Cu(bcp)DPEphos]PF_6

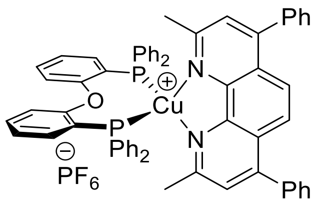

A mixture of tetrakisacetonitrile copper(I) hexafluorophosphate (3.73 g, 10.0 mmol) and bis[(2-diphenylphosphino)phenyl] ether (DPEphos, 5.39 g, 10.0 mmol) in anhydrous dichloromethane (800 mL) was stirred for 2 hours at rt under argon in the dark. A solution of bathocuproine (bcp, 3.60 g, 10.0 mmol) in anhydrous dichloromethane (200 mL) was then added and the mixture was stirred for an additional hour in the dark. The mixture was then filtered through a pad of Celite® and concentrated to ca. 50-100 mL under reduced pressure. The concentrate was then added dropwise to 1 L of Et_2O with vigorous stirring in the dark. The precipitate was collected by filtration and dried under vacuum to afford [Cu(bcp)DPEphos]PF_6 (10.1 g, 9.12 mmol, 91%) as a bright yellow solid. The spectroscopic data correspond to those previously described in the literature.[1,2]

2.2. Experimental Procedures and Characterization Data: Synthesis of Unreported Starting Materials

General procedure: synthesis of *N*-(*tert*-butyldimethylsilyloxy)alkylmethanesulfonamides

To a solution of the (*tert*-butyldimethylsilyloxy)alkylamine derivative (1.0 equiv.) and triethylamine (2.0 equiv.) in anhydrous dichloromethane (0.1 M) was added methanesulfonyl chloride (1.2 equiv.) dropwise at 0 °C under argon. The resulting mixture was stirred at rt overnight, quenched with a saturated aqueous solution of $NaHCO_3$ and extracted with dichloromethane. The combined organic layers were then washed with water, brine, dried over $MgSO_4$, filtered and concentrated under reduced pressure to afford the desired product without further purification, unless otherwise stated.

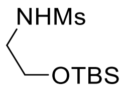

SA

***N*-{2-[(*tert*-Butyldimethylsilyl)oxy]ethyl}methanesulfonamide SA**. Prepared according to general procedure (47.3 mmol of 2-[(*tert*-butyldimethylsilyl)oxy]ethan-1-amine). Yield: 88% (10.6 g, 41.8 mmol). Off-white solid; Mp: 52 °C; ^{1}H NMR (400 MHz, $CDCl_3$): δ 4.74 (br. s, 1H), 3.72 (t, *J* = 5.2 Hz, 2H), 3.21 (app. q, *J* = 5.5 Hz, 2H), 2.96 (s, 3H), 0.88 (s, 9H), 0.06 (s, 6H); ^{13}C NMR (100 MHz, $CDCl_3$): δ 62.1, 45.5, 40.5, 26.0, 18.4, -5.3; ESIHRMS *m/z* calcd for $C_9H_{24}NO_3SSi$ $[M+H]^+$ 254.1241, found 254.1247.

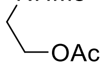

SB

***N*-(2-Acetoxyethyl)methanesulfonamide SB**. To a solution of *N*-{2-[(*tert*-butyldimethylsilyl)oxy]ethyl}methanesulfonamide **SA** (1.27 g, 5.0 mmol) in anhydrous tetrahydrofuran (25 mL) was added a solution of tetrabutylammonium fluoride (TBAF, 1 M solution in tetrahydrofuran, 7.5 mL, 7.5 mmol) dropwise at 0 °C under argon. The resulting mixture was stirred at rt for 2 hours, quenched by addition of a 4 M solution of HCl in dioxane

(1.5 mL, 6.0 mmol) and concentrated under reduced pressure. To a solution of this crude residue and triethylamine (1.39 mL, 10.0 mmol) in dichloromethane (25 mL) was added acetic anhydride (567 µL, 6.0 mmol) dropwise at 0 °C under argon. The resulting mixture was stirred at rt overnight, quenched with a 1 M aqueous solution of HCl and extracted with dichloromethane. The combined organic layers were then washed with brine, dried over $MgSO_4$, filtered and concentrated under reduced pressure. The crude residue was finally purified by flash column chromatography over silica gel (petroleum ether/EtOAc: 75/25) to afford the desired product as a colorless oil (360 mg, 1.99 mmol, 40%). ^1H NMR (400 MHz, $CDCl_3$): δ 5.00 (br. t, J = 6.3 Hz, 1H), 4.19 (t, J = 5.3 Hz, 2H), 3.39 (app. q, J = 5.6 Hz, 2H), 2.97 (s, 3H), 2.08 (s, 3H); ^{13}C NMR (100 MHz, $CDCl_3$): δ 171.0, 63.4, 42.3, 40.9, 20.9; ESIHRMS *m/z* calcd for $C_5H_{12}NO_4S$ $[M+H]^+$ 182.0482, found 182.0476.

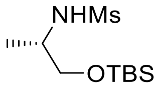

SC

(*S*)-*N*-{1-[(*tert*-Butyldimethylsilyl)oxy]propan-2-yl}methanesulfonamide SC. Prepared according to general procedure (36.9 mmol of (*S*)-1-[(*tert*-butyldimethylsilyl)oxy]propan-2-amine). Yield: 75% (7.4 g, 27.7 mmol). Yellow oil; $[\alpha]_D^{25}$ + 2 (c 1.2, $CHCl_3$); ^1H NMR (400 MHz, $CDCl_3$): δ 4.50 (d, J = 6.8 Hz, 1H), 3.66 (A of ABX syst., J = 9.8 and 4.0 Hz, 1H), 3.62-3.52 (m, 1H), 3.49 (B of ABX syst., J = 9.8 and 5.3 Hz, 1H), 2.98 (s, 3H), 1.22 (d, J = 6.5 Hz, 3H), 0.90 (s, 9H), 0.07 (s, 3H), 0.07 (s, 3H); ^{13}C NMR (100 MHz, $CDCl_3$): δ 66.8, 51.6, 41.7, 25.9, 18.7, 18.4, -5.4, -5.4; ESIHRMS *m/z* calcd for $C_{10}H_{26}NO_3SSi$ $[M+H]^+$ 268.1397, found 268.1398.

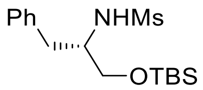

SD

(*S*)-*N*-{1-[(*tert*-Butyldimethylsilyl)oxy]-3-phenylpropan-2-yl}methanesulfonamide SD. Prepared according to general procedure (12.2 mmol of (*S*)-1-[(*tert*-butyldimethylsilyl)oxy]-3-phenylpropan-2-amine). Yield: 86% (3.6 g, 10.5 mmol). Pale yellow solid; Mp: 73 °C; $[\alpha]_D^{20}$ - 15 (c 1.0, $CHCl_3$); ^1H NMR (400 MHz, $CDCl_3$): δ 7.34-7.29 (m, 2H), 7.26-7.19 (m, 3H), 4.52 (d, J =

8.4 Hz, 1H), 3.69-3.57 (m, 3H), 2.92 (A of ABX syst., *J* = 13.6 and 5.9 Hz, 1H), 2.80 (B of ABX syst., *J* = 13.6 and 8.0 Hz, 1H), 2.50 (s, 3H), 0.93 (s, 9H), 0.08 (s, 6H); ^{13}C NMR (100 MHz, $CDCl_3$): *δ* 138.2, 129.7, 128.8, 127.0, 65.2, 57.5, 41.2, 38.9, 26.0, 18.4, -5.3; ESIHRMS *m/z* calcd for $C_{16}H_{30}NO_3SSi$ $[M+H]^+$ 344.1710, found 344.1717.

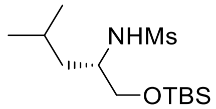

SE

(*S*)-*N*-{1-[(*tert*-Butyldimethylsilyl)oxy]-4-methylpentan-2-yl}methanesulfonamide SE. Prepared according to general procedure (4.3 mmol of (*S*)-1-[(*tert*-butyldimethylsilyl)oxy]-4-methylpentan-2-amine). Yield: 98% (1.3 g, 4.2 mmol). Yellow oil; $[\alpha]_D^{20}$ - 30 (*c* 0.5, $CHCl_3$); ^{1}H NMR (400 MHz, $CDCl_3$): *δ* 4.38 (d, *J* = 8.8 Hz, 1H), 3.71 (A of ABX syst., *J* = 10.1 and 3.8 Hz, 1H), 3.55 (B of ABX syst., *J* = 10.1 and 4.1 Hz, 1H), 3.53-3.45 (m, 1H), 2.99 (s, 3H), 1.72 (app. sept., *J* = 6.6 Hz, 1H), 1.45-1.30 (m, 2H), 0.94 (d, *J* = 6.6 Hz, 6H), 0.90 (s, 9H), 0.07 (s, 3H), 0.07 (s, 3H); ^{13}C NMR (100 MHz, $CDCl_3$): *δ* 65.8, 54.0, 42.3, 41.9, 26.0, 24.7, 23.1, 22.4, 18.5, -5.3, -5.3; ESIHRMS *m/z* calcd for $C_{13}H_{32}NO_3SSi$ $[M+H]^+$ 310.1867, found 310.1866.

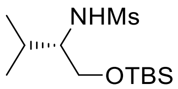

SF

(*S*)-*N*-{1-[(*tert*-Butyldimethylsilyl)oxy]-3-methylbutan-2-yl}methanesulfonamide SF. Prepared according to general procedure (9.7 mmol of (*S*)-1-[(*tert*-butyldimethylsilyl)oxy]-3-methylbutan-2-amine). Yield: 94% (2.7 g, 9.1 mmol). Solvent system for flash column chromatography: petroleum ether/EtOAc: 80/20; Colorless oil; $[\alpha]_D^{20}$ - 24 (*c* 1.4, $CHCl_3$); ^{1}H NMR (400 MHz, $CDCl_3$): *δ* 4.65 (d, *J* = 9.2 Hz, 1H), 3.66 (d, *J* = 4.7 Hz, 2H), 3.16 (ddt, *J* = 9.3, 6.3 and 4.7 Hz, 1H), 2.97 (s, 3H), 1.88 (app. oct., *J* = 6.8 Hz, 1H), 0.94 (d, *J* = 6.8 Hz, 3H), 0.93 (d, *J* = 6.9 Hz, 3H), 0.88 (s, 9H), 0.06 (s, 3H), 0.05 (s, 3H); ^{13}C NMR (100 MHz, $CDCl_3$): *δ* 63.8, 61.1, 41.8, 29.7, 26.0, 19.5, 18.5, 18.4, -5.4; ESIHRMS *m/z* calcd for $C_{12}H_{30}NO_3SSi$ $[M+H]^+$ 296.1710, found 296.1726.

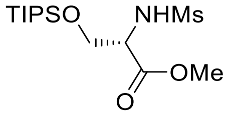

SG

Methyl *N*-(methanesulfonyl)-*O*-(triisopropylsilyl)-L-serinate SG. To a solution of L-serine methyl ester hydrochloride (202 mg, 1.3 mmol) in anhydrous dichloromethane (6.5 mL) were successively added triisopropylsilyl chloride (584 µL, 2.73 mmol) and imidazole (265 mg, 3.9 mmol) at 0 °C under argon. The resulting mixture was stirred at rt overnight, quenched with methanol and concentrated under reduced pressure. The residue was then diluted with dichloromethane, washed with a saturated aqueous solution of $NaHCO_3$, water and brine. The organic layer was then dried over $MgSO_4$, filtered and concentrated under reduced pressure. To a solution of this crude residue and triethylamine (996 µL, 7.15 mmol) in anhydrous dichloromethane (13 mL) was added methanesulfonyl chloride (302 µL, 3.9 mmol) dropwise at 0 °C under argon. The resulting mixture was stirred at rt overnight, diluted with dichloromethane and washed with a saturated aqueous solution of $NaHCO_3$, water and brine. The organic layer was then dried over $MgSO_4$, filtered and concentrated under reduced pressure. Residual triisopropylmethoxysilane was evaporated at 90 °C under high vacuum to afford the desired product as a dark orange oil (289 mg, 817 µmol, 63%). $[\alpha]_D^{20}$ - 8 (*c* 1.7, $CHCl_3$); ^1H NMR (400 MHz, $CDCl_3$): *δ* 5.21 (d, *J* = 9.1 Hz, 1H), 4.26-4.18 (m, 2H), 3.98 (app. dd, *J* = 9.4 and 2.7 Hz, 1H), 3.78 (s, 3H), 3.01 (s, 3H), 1.10-1.00 (m, 21H); ^{13}C NMR (100 MHz, $CDCl_3$): *δ* 171.0, 65.5, 58.2, 52.8, 42.0, 17.9, 17.9, 11.9; ESIHRMS *m/z* calcd for $C_{14}H_{32}NO_5SSi$ $[M+H]^+$ 354.1765, found 354.1765.

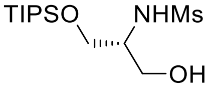

SH

(*R*)-*N*-{1-Hydroxy-3-[(triisopropylsilyl)oxy]propan-2-yl}methanesulfonamide SH. To a solution of methyl *N*-(methanesulfonyl)-*O*-(triisopropylsilyl)-L-serinate **SG** (505 mg, 1.43 mmol) in anhydrous tetrahydrofuran (7 mL) was added $LiBH_4$ (125 mg, 5.72 mmol) in three portions at 0 °C under argon. The resulting mixture was stirred at rt for 1 hour, quenched with a saturated aqueous solution of NH_4Cl and extracted with ethyl acetate. The combined organic layers were then washed with brine, dried over $MgSO_4$, filtered and concentrated under

reduced pressure. The crude residue was finally purified by flash column chromatography over silica gel (petroleum ether/EtOAc: 60/40) to afford the desired product as a colorless oil (313 mg, 962 µmol, 67%). $[\alpha]_D^{25}$ + 13 (c 0.3, $CHCl_3$); ^1H NMR (400 MHz, $CDCl_3$): δ 4.95 (d, J = 8.1 Hz, 1H), 3.88 (d, J = 4.6 Hz, 2H), 3.85-3.72 (m, 2H), 3.54 (app. dquint., J = 8.0 and 4.7 Hz, 1H), 3.03 (s, 3H), 2.31 (br. t, J = 5.3 Hz, 1H), 1.08-1.04 (m, 21H); ^{13}C NMR (100 MHz, $CDCl_3$): δ 64.7, 63.6, 56.5, 41.6, 18.1, 11.9; ESIHRMS m/z calcd for $C_{13}H_{32}NO_4SSi$ $[M+H]^+$ 326.1816, found 326.1811.

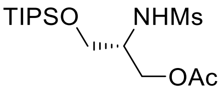

SI

***(R)*-*N*-{1-Acetoxy-3-[(triisopropylsilyl)oxy]propan-2-yl}methanesulfonamide SI**. To a solution of (*R*)-*N*-{1-hydroxy-3-[(triisopropylsilyl)oxy]propan-2-yl}methanesulfonamide **SH** (518 mg, 1.59 mmol) in anhydrous dichloromethane (4 mL) were successively added pyridine (180 µL, 2.23 mmol) and acetic anhydride (165 µL, 1.75 mmol) at 0 °C. The resulting mixture was stirred at rt overnight, quenched with a saturated aqueous solution of $NaHCO_3$ and extracted with ethyl acetate. The combined organic layers were then washed with brine, dried over $MgSO_4$, filtered and concentrated under reduced pressure to afford the desired product as a colorless oil without further purification (560 mg, 1.52 mmol, 96%). $[\alpha]_D^{25}$ + 10 (c 2.0, $CHCl_3$); ^1H NMR (400 MHz, $CDCl_3$): δ 4.86 (d, J = 8.6 Hz, 1H), 4.21 (A of ABX syst., J = 11.3 and 6.4 Hz, 1H), 4.16 (B of ABX syst., J = 11.3 and 5.6 Hz, 1H), 3.84 (A' of A'B'X syst., J = 10.0 and 4.0 Hz, 1H), 3.78 (B' of A'B'X syst., J = 10.0 and 4.9 Hz, 1H), 3.75-3.67 (m, 1H), 3.00 (s, 3H), 2.07 (s, 3H), 1.14-1.01 (m, 21H); ^{13}C NMR (100 MHz, $CDCl_3$): δ 170.8, 63.8, 63.3, 54.1, 41.9, 20.9, 18.0, 11.9; ESIHRMS m/z calcd for $C_{15}H_{34}NO_5SSi$ $[M+H]^+$ 368.1921, found 368.1918.

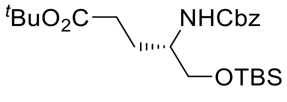

SJ

Benzyl (*S*)-{1-[(*tert*-butyldimethylsilyl)oxy]-4-*tert*-butoxycarbonylbutan-2-yl}carbamate SJ. To a solution of benzyl (*S*)-(1-hydroxy-4-*tert*-butoxycarbonylbutan-2-yl)carbamate (303 mg,

937 µmol), imidazole (76 mg, 1.12 mmol) and 4-dimethylaminopyridine (11 mg, 90 µmol) in anhydrous dichloromethane (4.7 mL) was added *tert*-butyldimethylsilyl chloride (169 mg, 1.12 mmol) in one portion at 0 °C under argon. The resulting mixture was stirred at rt overnight, quenched with a saturated aqueous solution of $NaHCO_3$ and extracted with dichloromethane. The combined organic layers were washed with a 1 M aqueous solution of HCl, a saturated aqueous solution of $NaHCO_3$, brine, dried over $MgSO_4$, filtered and concentrated under reduced pressure. The crude residue was finally purified by flash column chromatography over silica gel (petroleum ether/EtOAc: 80/20) to afford the desired product as a yellow oil (360 mg, 823 µmol, 88%). $[\alpha]_D^{25}$ - 11 (*c* 0.9, $CHCl_3$); ^1H NMR (400 MHz, $CDCl_3$): *δ* 7.37-7.28 (m, 5H), 5.09 (s, 2H), 4.94 (d, *J* = 8.8 Hz, 1H), 3.74-3.64 (m, 1H), 3.61 (app. d, *J* = 3.4 Hz, 2H), 2.30 (app. td, *J* = 7.6 and 2.2 Hz, 2H), 1.88-1.72 (m, 2H), 1.43 (s, 9H), 0.88 (s, 9H), 0.04 (s, 3H), 0.03 (s, 3H); ^{13}C NMR (100 MHz, $CDCl_3$): *δ* 172.9, 156.2, 136.8, 128.7, 128.2 (2C), 80.5, 66.8, 65.1, 52.4, 32.4, 28.2, 27.1, 26.0, 18.4, -5.4; ESIHRMS *m/z* calcd for $C_{23}H_{40}NO_5Si$ $[M+H]^+$ 438.2670, found 438.2671.

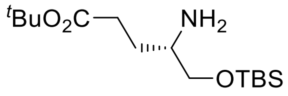

SK

(*S*)-1-[(*tert*-Butyldimethylsilyl)oxy]-(4-*tert*-butoxycarbonyl)butan-2-amine SK. A glass vial was charged with benzyl (*S*)-{1-[(*tert*-butyldimethylsilyl)oxy]-4-*tert*-butoxycarbonylbutan-2-yl}carbamate **SJ** (713 mg, 1.63 mmol), palladium on carbon 10% wt. (60 mg, 57 µmol) and methanol (5.4 mL). The vial was placed in an autoclave, purged with N_2, pressurized with H_2 (2 bars) and stirred at rt for 3 hours. After the reactor was depressurized and purged with N_2, the reaction mixture was filtered over a plug of Celite® (washed with methanol) and concentrated under reduced pressure to afford the desired product as a yellow oil without further purification (473 mg, 1.56 mmol, 96%). $[\alpha]_D^{25}$ + 8 (*c* 1.2, $CHCl_3$); ^1H NMR (400 MHz, $CDCl_3$): *δ* 3.55 (A of ABX syst., *J* = 9.8 and 4.1 Hz, 1H), 3.33 (B of ABX syst., *J* = 9.8 and 7.0 Hz, 1H), 2.83-2.75 (m, 1H), 2.40-2.22 (m, 2H), 1.70 (dddd, *J* = 13.7, 8.6, 7.0 and 5.0 Hz, 1H), 1.54-1.45 (obs. m, 1H), 1.46-1.40 (obs. m, 2H), 1.43 (s, 9H), 0.88 (s, 9H), 0.04 (s, 6H); ^{13}C NMR

(100 MHz, $CDCl_3$): δ 173.2, 80.3, 68.4, 52.6, 32.5, 29.3, 28.2, 26.0, 18.4, -5.2, -5.2; ESIHRMS *m/z* calcd for $C_{15}H_{34}NO_3Si$ $[M+H]^+$ 304.2302, found 304.2303.

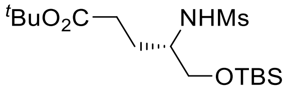

SL

(*S*)-{1-[(*tert*-Butyldimethylsilyl)oxy]-(4-*tert*-butoxycarbonyl)butan-2-yl}methanesulfonamide SL. Prepared according to general procedure (1.20 mmol of (*S*)-1-[(*tert*-butyldimethylsilyl)oxy]-(4-*tert*-butoxycarbonyl)butan-2-amine **SK**). Yield: 87% (401 mg, 1.05 mmol). Solvent system for flash column chromatography: petroleum ether/EtOAc: 80/20; Off-white solid; Mp: 47 °C; $[\alpha]_D^{25}$ - 12 (*c* 1.7, $CHCl_3$); ^1H NMR (400 MHz, $CDCl_3$): δ 4.58 (d, *J* = 8.9 Hz, 1H), 3.72 (A of ABX syst., *J* = 10.2 and 4.2 Hz, 1H), 3.59 (B of ABX syst., *J* = 10.2 and 4.2 Hz, 1H), 3.47 (app. oct., *J* = 4.4 Hz, 1H), 2.99 (s, 3H), 2.38 (app. t, *J* = 7.2 Hz, 2H), 1.87-1.70 (m, 2H), 1.45 (s, 9H), 0.90 (s, 9H), 0.08 (s, 3H), 0.07 (s, 3H); ^{13}C NMR (100 MHz, $CDCl_3$): δ 172.8, 80.9, 65.7, 55.1, 41.9, 31.8, 28.2, 27.6, 26.0, 18.5, -5.3, -5.3; ESIHRMS *m/z* calcd for $C_{16}H_{39}N_2O_5SSi$ $[M+NH_4]^+$ 399.2343, found 399.2346.

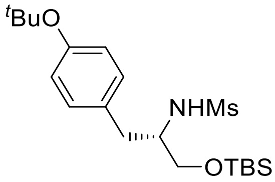

SM

(*S*)-*N*-{1-[4-(*tert*-Butoxy)phenyl]-3-[(*tert*-butyldimethylsilyl)oxy]propan-2-yl}methanesulfonamide SM. Prepared according to general procedure (2.37 mmol of (*S*)-1-[4-(*tert*-butoxy)phenyl]-3-[(*tert*-butyldimethylsilyl)oxy]propan-2-amine). Yield: 50% (490 mg, 1.18 mmol). Solvent system for flash column chromatography: petroleum ether/EtOAc: 90/10, then 80/20; Colorless oil; $[\alpha]_D^{20}$ - 55 (*c* 0.7, $CHCl_3$); ^1H NMR (400 MHz, $CDCl_3$): δ 7.11 (d, *J* = 8.4 Hz, 2H), 6.94 (d, *J* = 8.4 Hz, 2H), 4.52 (d, *J* = 8.7 Hz, 1H), 3.68-3.63 (m, 2H), 3.62-3.54 (m, 1H), 2.87 (A of ABX syst., *J* = 13.8 and 5.9 Hz, 1H), 2.74 (B of ABX syst., *J* = 13.8 and 8.5 Hz, 1H), 2.49 (s, 3H), 1.32 (s, 9H), 0.92 (s, 9H), 0.07 (s, 3H), 0.07 (s, 3H); ^{13}C NMR (100 MHz, $CDCl_3$): δ 154.3,

133.2, 130.2, 124.6, 78.6, 65.4, 57.6, 41.2, 38.2, 28.9, 26.0, 18.4, -5.3, -5.3; ESIHRMS *m/z* calcd for $C_{20}H_{41}N_2O_4SSi$ $[M+NH_4]^+$ 433.2551, found 433.2536.

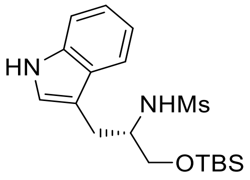

SN

(*S*)-*N*-{1-[(*tert*-Butyldimethylsilyl)oxy]-3-(1*H*-indol-3-yl)propan-2-yl}methanesulfonamide SN. Prepared according to general procedure (4.9 mmol of (*S*)-1-[(*tert*-butyldimethylsilyl)oxy]-3-(1*H*-indol-3-yl)propan-2-amine). Yield: 86% (1.61 g, 4.20 mmol). Yellow oil; $[\alpha]_D^{20}$ - 27 (*c* 0.5, $CHCl_3$); ^1H NMR (400 MHz, $CDCl_3$): δ 8.05 (br. s, 1H), 7.65 (d, *J* = 7.9 Hz, 1H), 7.37 (app. d, *J* = 7.8 Hz, 1H), 7.21 (app. td, *J* = 7.2 and 1.1 Hz, 1H), 7.14 (app. td, *J* = 7.2 and 1.1 Hz, 1H), 7.07 (d, *J* = 2.3 Hz, 1H), 4.52 (d, *J* = 8.3 Hz, 1H), 3.80-3.72 (m, 1H), 3.69 (A of ABX syst., *J* = 10.0 and 3.8 Hz, 1H), 3.65 (B of ABX syst., *J* = 10.0 and 4.9 Hz, 1H), 3.10 (A' of A'B'X syst., *J* = 14.5 and 6.6 Hz, 1H), 3.01 (B' of A'B'X syst., *J* = 14.5 and 7.0 Hz, 1H), 2.57 (s, 3H), 0.94 (s, 9H), 0.08 (s, 6H); ^{13}C NMR (100 MHz, $CDCl_3$): δ 136.4, 127.6, 123.0, 122.6, 120.0, 119.0, 112.1, 111.4, 65.1, 56.1, 41.2, 28.4, 26.1, 18.5, -5.2; ESIHRMS *m/z* calcd for $C_{18}H_{31}N_2O_3SSi$ $[M+H]^+$ 383.1819, found 383.1821.

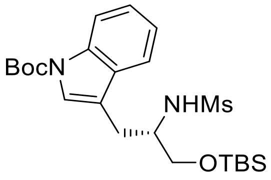

SO

(*S*)-*N*-{1-[(*tert*-Butyldimethylsilyl)oxy]-3-(*N*-*tert*-butoxycarbonyl-1*H*-indol-3-yl)propan-2-yl}methanesulfonamide SO. To a solution of (*S*)-*N*-{1-[(*tert*-butyldimethylsilyl)oxy]-3-(1*H*-indol-3-yl)propan-2-yl}methanesulfonamide **SN** (688 mg, 1.80 mmol) and 4-dimethylaminopyridine (9 mg, 74 µmol) in anhydrous dichloromethane (18 mL) was added di-*tert*-butyl dicarbonate (472 mg, 2.16 mmol). The resulting mixture was stirred at rt for 2 hours, quenched with H_2O and extracted with dichloromethane. The combined organic layers were

washed with brine, dried over $MgSO_4$, filtered and concentrated under reduced pressure. The crude residue was finally purified by flash column chromatography over silica gel (petroleum ether/EtOAc: 90/10) to afford the desired product as an off-white solid (621 mg, 1.29 mmol, 72%). Mp: 91 °C; $[\alpha]_D^{20}$ - 18 (*c* 0.7, $CHCl_3$); ^1H NMR (400 MHz, $CDCl_3$): *δ* 8.14 (d, *J* = 8.1 Hz, 1H), 7.60 (d, *J* = 7.7 Hz, 1H), 7.43 (s, 1H), 7.33 (app. td, *J* = 7.3 and 1.2 Hz, 1H), 7.26 (obs. app. td, *J* = 7.3 and 1.1 Hz, 1H), 4.60 (d, *J* = 8.5 Hz, 1H), 3.81-3.72 (m, 1H), 3.67 (A of ABX syst., *J* = 10.5 and 3.7 Hz, 1H), 3.64 (B of ABX syst., *J* = 10.5 and 4.4 Hz, 1H), 3.03 (A' of A'B'X syst., *J* = 14.3 and 7.3 Hz, 1H), 2.98 (B' of A'B'X syst., *J* = 14.3 and 6.8 Hz, 1H), 2.71 (s, 3H), 1.67 (s, 9H), 0.94 (s, 9H), 0.08 (s, 6H); ^{13}C NMR (100 MHz, $CDCl_3$): *δ* 149.7, 135.7, 130.4, 124.9, 124.3, 122.9, 119.2, 116.6, 115.5, 83.9, 64.6, 55.3, 41.6, 28.4, 28.3, 26.1, 18.4, -5.3; ESIHRMS *m/z* calcd for $C_{23}H_{38}N_2O_5SSiNa$ $[M+Na]^+$ 505.2163, found 505.2163.

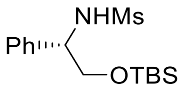

SP

(*S*)-*N*-{2-[(*tert*-Butyldimethylsilyl)oxy]-1-phenylethyl}methanesulfonamide SP. Prepared according to general procedure (33.8 mmol of (*S*)-2-[(*tert*-butyldimethylsilyl)oxy]-1-phenylethan-1-amine). Yield: 75% (8.40 g, 25.5 mmol). Solvent system for flash column chromatography: petroleum ether/EtOAc: 85/15; White solid; Mp: 82 °C; $[\alpha]_D^{20}$ + 49 (*c* 1.1, $CHCl_3$); ^1H NMR (400 MHz, $CDCl_3$): *δ* 7.39-7.28 (m, 5H), 5.17 (d, *J* = 5.4 Hz, 1H), 4.57 (ddd, *J* = 6.7, 5.4 and 4.2 Hz, 1H), 3.88 (A of ABX syst., *J* = 10.2 and 4.3 Hz, 1H), 3.68 (B of ABX syst., *J* = 10.2 and 6.8 Hz, 1H), 2.66 (s, 3H), 0.88 (s, 9H), 0.01 (s, 3H), -0.01 (s, 3H); ^{13}C NMR (100 MHz, $CDCl_3$): *δ* 138.6, 128.8, 128.4, 127.5, 67.0, 59.4, 42.0, 25.9, 18.4, -5.4, -5.4; ESIHRMS *m/z* calcd for $C_{15}H_{28}NO_3SSi$ $[M+H]^+$ 330.1554, found 330.1543.

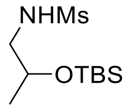

SQ

***N*-{2-[(*tert*-Butyldimethylsilyl)oxy]propyl}methanesulfonamide SQ**. Prepared according to general procedure (11.1 mmol of 2-[(*tert*-butyldimethylsilyl)oxy]propan-1-amine). Yield: 51% (1.5 g, 5.61 mmol). Orange solid; Mp: 53 °C; ^{1}H NMR (400 MHz, $CDCl_3$): δ 4.48 (br. s, 1H), 4.00 (app. quint.d, *J* = 6.3 and 3.6 Hz, 1H), 3.18 (ddd, *J* = 12.5, 7.0 and 3.6 Hz, 1H), 3.01-2.94 (obs. m, 1H), 2.96 (s, 3H), 1.17 (d, *J* = 6.2 Hz, 3H), 0.89 (s, 9H), 0.10 (s, 3H), 0.09 (s, 3H); ^{13}C NMR (100 MHz, $CDCl_3$): δ 67.6, 50.4, 40.4, 25.9, 21.1, 18.1, -4.3, -4.7; ESIHRMS *m/z* calcd for $C_{10}H_{26}NO_3SSi$ $[M+H]^+$ 268.1397, found 268.1405.

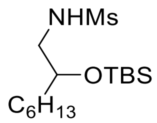

SR

***N*-{2-[(*tert*-Butyldimethylsilyl)oxy]octyl}methanesulfonamide SR**. To a solution of 1-aminooctan-2-ol (581 mg, 4.0 mmol) and imidazole (409 mg, 6.0 mmol) in anhydrous dichloromethane (16 mL) was added *tert*-butyldimethylsilyl chloride (663 mg, 4.4 mmol) in one portion at 0 °C under argon. The resulting mixture was stirred at rt for 16 hours, quenched with a saturated aqueous solution of $NaHCO_3$ and extracted with dichloromethane. The combined organic layers were washed with a 1 M aqueous solution of HCl, brine, dried over $MgSO_4$, filtered and concentrated under reduced pressure. To a solution of this crude residue and triethylamine (1.12 mL, 8.0 mmol) in anhydrous dichloromethane (16 mL) was added methanesulfonyl chloride (372 µL, 4.8 mmol) dropwise at 0 °C under argon. The resulting mixture was stirred at rt overnight, quenched with a saturated aqueous solution of $NaHCO_3$ and extracted with dichloromethane. The combined organic layers were then washed with water, brine, dried over $MgSO_4$, filtered and concentrated under reduced pressure. The crude residue was finally purified by flash column chromatography over silica gel (petroleum ether/EtOAc: 75/25) to afford the desired product as a pale yellow oil (823 mg, 2.44 mmol, 61%). ^{1}H NMR (400 MHz, $CDCl_3$): δ 4.48 (t, *J* = 5.7 Hz, 1H), 3.80 (app. quint., *J* = 5.9 Hz, 1H), 3.22-3.13 (m, 1H), 3.08-3.00 (m, 1H), 2.94 (s, 3H), 1.54-1.43 (m, 2H), 1.34-1.22 (m, 9H),

0.90-0.86 (obs. m, 2H), 0.89 (s, 9H), 0.09 (s, 3H), 0.07 (s, 3H); ^{13}C NMR (100 MHz, $CDCl_3$): δ 71.3, 48.3, 40.3, 34.9, 31.8, 29.4, 25.9, 25.2, 22.7, 18.2, 14.2, -4.3, -4.4; ESIHRMS *m/z* calcd for $C_{15}H_{36}NO_3SSi$ $[M+H]^+$ 338.2180, found 338.2186.

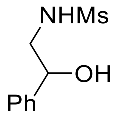

SS

***N*-(2-Hydroxy-2-phenylethyl)methanesulfonamide SS**. A solution of 1-(methanesulfonyl)-2-phenylaziridine (1.0 g, 5.1 mmol) and sodium bisulfite (1.06 g, 10.2 mmol) in a 1:1 mixture of acetone and water (50 mL) was stirred at 55 °C for 6 hours. Acetone was evaporated under reduced pressure and the aqueous layer was then extracted with dichloromethane. The combined organic layers were washed with brine, dried over $MgSO_4$, filtered and concentrated under reduced pressure. The crude residue was finally purified by flash column chromatography over silica gel (petroleum ether/EtOAc: 50/50) to afford the desired product as a white solid (844 mg, 3.92 mmol, 77%). Mp: 101 °C; ^{1}H NMR (400 MHz, $CDCl_3$): δ 7.39-7.36 (m, 4H), 7.36-7.28 (m, 1H), 4.92 (obs. br. s, 1H), 4.89 (dd, J = 8.1 and 3.7 Hz, 1H), 3.43 (ddd, J = 13.7, 7.8 and 4.0 Hz, 1H), 3.27 (ddd, J = 13.2, 8.1 and 4.6 Hz, 1H), 2.91 (s, 3H), 2.66 (br. s, 1H); ^{13}C NMR (100 MHz, $CDCl_3$): δ 140.9, 128.9, 128.5, 126.0, 73.3, 50.4, 40.7; ESIHRMS *m/z* calcd for $C_9H_{17}N_2O_3S$ $[M+NH_4]^+$ 233.0954, found 233.0956.

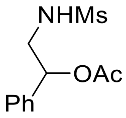

ST

***N*-(2-Acetoxy-2-phenylethyl)methanesulfonamide ST**. To a solution of *N*-(2-hydroxy-2-phenylethyl)methanesulfonamide **SS** (538 mg, 2.5 mmol) and triethylamine (697 µL, 5.0 mmol) in anhydrous dichloromethane (10 mL) was added acetic anhydride (284 µL, 3.0 mmol) dropwise at 0 °C under argon. The resulting mixture was stirred at rt overnight, quenched with a 1 M aqueous solution of HCl and extracted with dichloromethane. The combined organic layers were washed with brine, dried over $MgSO_4$, filtered and concentrated under reduced

pressure. The crude residue was finally purified by flash column chromatography over silica gel (petroleum ether/EtOAc: 70/30) to afford the desired product as a colorless oil (381 mg, 1.48 mmol, 59%). ^{1}H NMR (400 MHz, $CDCl_3$): *δ* 7.39-7.29 (m, 5H), 5.85 (dd, *J* = 6.7 and 5.3 Hz, 1H), 5.06 (t, *J* = 6.4 Hz, 1H), 3.52-3.47 (m, 2H), 2.82 (s, 3H), 2.12 (s, 3H); ^{13}C NMR (100 MHz, $CDCl_3$): *δ* 170.2, 137.2, 128.9, 128.8, 126.6, 74.5, 47.9, 40.8, 21.2; ESIHRMS *m/z* calcd for $C_{11}H_{19}N_2O_4S$ $[M+NH_4]^+$ 275.1060, found 275.1062.

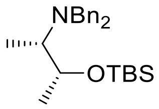

SU

(2*S*,3*R*)-*N*,*N*-Dibenzyl-3-[(*tert*-butyldimethylsilyl)oxy]butan-2-amine SU. To a solution of (2*S*,3*R*)-3-(dibenzylamino)butan-2-ol (8.51 g, 31.6 mmol), imidazole (4.30 g, 63.2 mmol) and *tert*-butyldimethylsilyl chloride (7.14 g, 47.4 mmol) in anhydrous dichloromethane (125 mL) was added 4-dimethylaminopyridine (579 mg, 4.74 mmol) at 0 °C under argon. The resulting mixture was stirred at rt for 60 hours and successively washed with a saturated aqueous solution of $NaHCO_3$, water and brine. The organic layer was dried over $MgSO_4$, filtered and concentrated under reduced pressure. The crude residue was finally purified by flash column chromatography over silica gel (petroleum ether/EtOAc: 95/5) to yield the desired product as a yellow oil (10.8 g, 28.1 mmol, 89%). $[\alpha]_D^{25}$ + 93 (*c* 1.0, $CHCl_3$); ^{1}H NMR (400 MHz, $CDCl_3$): *δ* 7.36 (app. d, *J* = 7.9 Hz, 4H), 7.29 (app. t, *J* = 7.5 Hz, 4H), 7.24-7.18 (m, 2H), 3.78 (dq, *J* = 8.0 and 6.1 Hz, 1H), 3.72 (A of AB syst., *J* = 13.8 Hz, 2H), 3.41 (B of AB syst., *J* = 13.9 Hz, 2H), 2.51 (dq, *J* = 7.9 and 6.6 Hz, 1H), 1.19 (d, *J* = 6.0 Hz, 3H), 1.07 (d, *J* = 6.6 Hz, 3H), 0.85 (s, 9H), 0.02 (s, 6H); ^{13}C NMR (100 MHz, $CDCl_3$): *δ* 140.6, 128.9, 128.3, 126.9, 70.9, 59.7, 54.7, 26.0, 22.6, 18.2, 8.8, -3.8, -4.6; ESIHRMS *m/z* calcd for $C_{24}H_{38}NOSi$ $[M+H]^+$ 384.2717, found 384.2723.

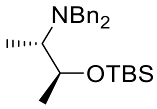

SV

(2*S*,3*S*)-*N*,*N*-Dibenzyl-3-[(*tert*-butyldimethylsilyl)oxy]butan-2-amine SV. To a solution of (2*S*,3*S*)-3-(dibenzylamino)butan-2-ol (1.1 g, 4.1 mmol), imidazole (558 mg, 8.2 mmol) and *tert*-butyldimethylsilyl chloride (927 mg, 6.15 mmol) in anhydrous tetrahydrofuran (16 mL) was added 4-dimethylaminopyridine (75 mg, 615 µmol) at 0 °C under argon. The resulting mixture was stirred at 40 °C overnight and successively washed with a saturated aqueous solution of $NaHCO_3$, water and brine. The organic layer was dried over $MgSO_4$, filtered and concentrated under reduced pressure. The crude residue was finally purified by flash column chromatography over silica gel (petroleum ether/EtOAc: 95/5) to yield the desired product as a yellow oil (1.14 g, 2.97 mmol, 72%). $[\alpha]_D^{25}$ + 11 (*c* 1.5, $CHCl_3$); ^1H NMR (400 MHz, $CDCl_3$): δ 7.39 (app. d, J = 7.5 Hz, 4H), 7.28 (app. t, J = 7.4 Hz, 4H), 7.23-7.17 (m, 2H), 4.06 (A of AB syst., J = 13.7 Hz, 2H), 3.75 (qd, J = 6.2 and 3.7 Hz, 1H), 3.35 (B of AB syst., J = 13.7 Hz, 2H), 2.57 (dq, J = 6.9 and 3.7 Hz, 1H), 1.18 (d, J = 6.3 Hz, 3H), 1.09 (d, J = 6.8 Hz, 3H), 0.85 (s, 9H), 0.00 (s, 3H), -0.02 (s, 3H); ^{13}C NMR (100 MHz, $CDCl_3$): δ 141.4, 129.0, 128.1, 126.6, 73.3, 57.3, 55.4, 26.0, 21.5, 18.2, 9.5, -4.2, -4.9; ESIHRMS *m/z* calcd for $C_{24}H_{38}NOSi$ $[M+H]^+$ 384.2717, found 384.2731.

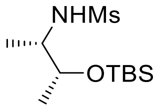

SW

***N*-{(2*S*,3*R*)-3-[(*tert*-Butyldimethylsilyl)oxy]butan-2-yl}methanesulfonamide SW**. A glass vial was charged with (2*S*,3*R*)-*N*,*N*-dibenzyl-3-[(*tert*-butyldimethylsilyl)oxy]butan-2-amine **SU** (2.22 g, 5.8 mmol), palladium on carbon 10% wt. (1.1 g, 1.03 mmol) and dichloromethane (15 mL). The vial was placed in an autoclave, purged with N_2, pressurized with H_2 (12 bars) and stirred at rt for 60 hours. After the reactor was depressurized and purged with N_2, the reaction mixture was filtered over a plug of Celite® (washed with dichloromethane) and concentrated under reduced pressure. To a solution of this crude residue and triethylamine (1.62 mL, 11.6 mmol) in anhydrous dichloromethane (58 mL) was added methanesulfonyl chloride (539 µL,

6.96 mmol) dropwise at 0 °C under argon. The resulting mixture was stirred at rt overnight, quenched with a saturated aqueous solution of $NaHCO_3$ and extracted with dichloromethane. The combined organic layers were washed with brine, dried over $MgSO_4$, filtered and concentrated under reduced pressure. The crude residue was finally purified by flash column chromatography over silica gel (petroleum ether/EtOAc: 80/20) to afford the desired product as a yellow oil (1.60 g, 5.68 mmol, 98%). $[\alpha]_D^{25}$ + 87 (*c* 4.0, $CHCl_3$); ^1H NMR (400 MHz, $CDCl_3$): *δ* 4.37 (d, *J* = 8.1 Hz, 1H), 3.91 (qd, *J* = 6.3 and 3.2 Hz, 1H), 3.44 (dqd, *J* = 8.1, 6.7 and 3.2 Hz, 1H), 2.98 (s, 3H), 1.17 (d, *J* = 6.7 Hz, 3H), 1.11 (d, *J* = 6.3 Hz, 3H), 0.90 (s, 9H), 0.10 (s, 3H), 0.08 (s, 3H); ^{13}C NMR (100 MHz, $CDCl_3$): *δ* 71.3, 55.3, 42.0, 26.0, 19.4, 18.2, 16.1, -4.2, -4.7; ESIHRMS *m/z* calcd for $C_{11}H_{28}NO_3SSi$ $[M+H]^+$ 282.1554, found 282.1561.

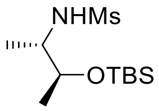

SX

***N*-{(2*S*,3*S*)-3-[(*tert*-Butyldimethylsilyl)oxy]butan-2-yl}methanesulfonamide SX**. A glass vial was charged with (2*S*,3*S*)-*N*,*N*-dibenzyl-3-[(*tert*-butyldimethylsilyl)oxy]butan-2-amine **SV** (1.31 g, 3.41 mmol), palladium on carbon 10% wt. (727 mg, 683 µmol) and dichloromethane (6.8 mL). The vial was placed in an autoclave, purged with N_2, pressurized with H_2 (12 bars) and stirred at 40 °C for 4 hours. After the reactor was depressurized and purged with N_2, the reaction mixture was filtered over a plug of Celite® (washed with dichloromethane) and concentrated under reduced pressure. To a solution of this crude residue and triethylamine (953 µL, 6.84 mmol) in anhydrous dichloromethane (34 mL) was added methanesulfonyl chloride (317 µL, 4.1 mmol) dropwise at 0 °C under argon. The resulting mixture was stirred at rt overnight, quenched with a saturated aqueous solution of $NaHCO_3$ and extracted with dichloromethane. The combined organic layers were washed with brine, dried over $MgSO_4$, filtered and concentrated under reduced pressure. The crude residue was finally purified by flash column chromatography over silica gel (petroleum ether/EtOAc: 80/20) to afford the desired product as a yellow oil (786 mg, 2.79 mmol, 82%). $[\alpha]_D^{25}$ + 5 (*c* 1.0, $CHCl_3$); ^1H NMR (400 MHz, $CDCl_3$): *δ* 4.48 (d, *J* = 8.6 Hz, 1H), 3.76 (qd, *J* = 6.2 and 2.5 Hz, 1H), 3.35 (dqd, *J* = 9.1, 6.6 and 2.5 Hz, 1H), 2.98 (s, 3H), 1.24 (d, *J* = 6.6 Hz, 3H), 1.20 (d, *J* = 6.2 Hz, 3H), 0.89 (s, 9H),

0.08 (s, 3H), 0.07 (s, 3H); ^{13}C NMR (100 MHz, $CDCl_3$): δ 71.2, 55.3, 42.2, 26.0, 21.1, 20.2, 18.2, -4.1, -4.8; ESIHRMS *m/z* calcd for $C_{11}H_{28}NO_3SSi$ $[M+H]^+$ 282.1554, found 282.1563.

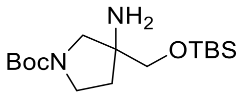

SY

1-(*tert*-Butoxycarbonyl)-3-{[(*tert*-butyldimethylsilyl)oxy]methyl}pyrrolidin-3-amine SY. To a solution of *tert*-butyl 3-amino-3-(hydroxymethyl)pyrrolidine-1-carboxylate (1.0 g, 4.62 mmol), imidazole (472 mg, 6.93 mmol) and 4-dimethylaminopyridine (85 mg, 696 μmol) in anhydrous dichloromethane (20 mL) was added *tert*-butyldimethylsilyl chloride (767 mg, 5.09 mmol) in one portion at 0 °C under argon. The resulting mixture was stirred at rt overnight, quenched with water, and exctracted with dichloromethane. The combined organic layers were washed with a saturated aqueous solution of $NaHCO_3$, brine, dried over $MgSO_4$, filtered and concentrated under reduced pressure. The crude residue was finally purified by flash column chromatography over silica gel (petroleum ether/EtOAc: 50/50) to afford the desired product as a colorless oil (1.3 g, 3.93 mmol, 85%). ^{1}H NMR (400 MHz, $CDCl_3$): δ 3.52-3.44 (m, 3H), 3.44-3.36 (m, 1H), 3.32 (d, *J* = 11.4 Hz, 1H), 3.14 (d, *J* = 11.1 Hz, 0.50H, rotamer), 3.02 (d, *J* = 11.0 Hz, 0.50H, rotamer), 1.86 (dt, *J* = 12.6 and 7.9 Hz, 1H), 1.68-1.58 (m, 1H), 1.55-1.44 (br. s, 2H), 1.44 (s, 9H), 0.89 (s, 9H), 0.05 (s, 6H); ^{13}C NMR (100 MHz, $CDCl_3$): δ 154.8, 79.3, 69.3 and 68.8 (rotamers), 61.1 and 60.4 (rotamers), 55.7, 44.9 and 44.4 (rotamers), 35.5 and 34.9 (rotamers), 28.6, 26.0, 18.4, -5.4; ESIHRMS *m/z* calcd for $C_{16}H_{35}N_2O_3Si$ $[M+H]^+$ 331.2411, found 331.2418.

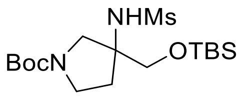

SZ

***N*-{1-(*tert*-Butoxycarbonyl)-3-{[(*tert*-butyldimethylsilyl)oxy]methyl}pyrrolidin-3-yl} methanesulfonamide SZ**. Prepared according to general procedure I (3.72 mmol of 1-(*tert*-butoxycarbonyl)-3-{[(*tert*-butyldimethylsilyl)oxy]methyl}pyrrolidin-3-amine **SY**). Yield: 90% (1.37 g, 3.35 mmol). Purification by filtration on a short pad of silica gel (washed with Et_2O); White solid; Mp: 108 °C; ^{1}H NMR (400 MHz, $CDCl_3$): δ 4.69 (d, *J* = 16.9 Hz, 1H), 3.79-3.55 (m,

3H), 3.54-3.46 (m, 1H), 3.45-3.35 (m, 2H), 3.02 (s, 3H), 2.25-2.09 (m, 1H), 2.05-1.93 (m, 1H), 1.45 (s, 9H), 0.90 (s, 9H), 0.08 (s, 3H), 0.07 (s, 3H); ^{13}C NMR (100 MHz, $CDCl_3$): δ 154.5, 80.0 and 79.9 (rotamers), 66.1 and 66.0 (rotamers), 65.8 and 65.7 (rotamers), 52.8 and 52.4 (rotamers), 44.4 and 44.3 (rotamers), 44.2 and 43.6 (rotamers), 33.4 and 32.5 (rotamers), 28.6, 26.0, 18.4, -5.4; ESIHRMS *m/z* calcd for $C_{17}H_{36}N_2O_5SSi$ $[M+Na]^+$ 431.2006, found 431.2001.

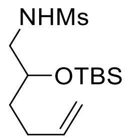

SAA

***N*-{2-[(*tert*-Butyldimethylsilyl)oxy]hex-5-en-1-yl}methanesulfonamide SAA**. A solution of 1,2-epoxy-5-hexene (2.88 mL, 25.5 mmol) in a 5:1 mixture of 25% aqueous ammonium hydroxide and acetonitrile (25 mL) was stirred at 120 °C for 1 hour in a pressure tube. The reaction mixture was then concentrated under reduced pressure. To a solution of this crude residue in anhydrous dichloromethane (100 mL) were successively added *tert*-butyldimethylsilyl chloride (4.22 g, 28.0 mmol), imidazole (2.60 g, 38.2 mmol) and 4-dimethylaminopyridine (467 mg, 3.82 mmol) at 0 °C under argon. The resulting mixture was stirred at rt overnight and washed with water, a saturated solution of $NaHCO_3$ and brine. The organic layer was then dried over $MgSO_4$, filtered and concentrated under reduced pressure. To a solution of this crude residue and triethylamine (7.11 mL, 51.0 mmol) in anhydrous dichloromethane (125 mL) was added methanesulfonyl chloride (2.37 mL, 30.6 mmol) dropwise at 0 °C. The resulting mixture was stirred at rt overnight, quenched with a saturated aqueous solution of $NaHCO_3$ and extracted with dichloromethane. The combined organic layers were washed with brine, dried over $MgSO_4$, filtered and concentrated under reduced pressure. The crude residue was finally purified by flash column chromatography over silica gel (petroleum ether/EtOAc: 85/15) to afford the desired product as a yellow oil (2.99 g, 9.72 mmol, 38%). ^{1}H NMR (400 MHz, $CDCl_3$): δ 5.77 (ddt, *J* = 16.9, 10.2 and 6.6 Hz, 1H), 5.01 (dq, *J* = 17.1 and 1.7 Hz, 1H), 4.96 (dq, *J* = 10.2 and 1.4 Hz, 1H), 4.56 (t, *J* = 6.1 Hz, 1H), 3.82 (tdd, *J* = 6.2, 5.2 and 4.0 Hz, 1H), 3.17 (ddd, *J* = 12.6, 5.8 and 4.1 Hz, 1H), 3.07 (ddd, *J* = 12.6, 6.5 and 5.3 Hz, 1H), 2.94 (s, 3H), 2.06 (dtt, *J* = 8.0, 6.5 and 1.5 Hz, 2H), 1.64-1.57 (m, 2H), 0.88 (s, 9H), 0.09

(s, 3H), 0.07 (s, 3H); ^{13}C NMR (100 MHz, $CDCl_3$): δ 137.9, 115.2, 70.7, 48.1, 40.3, 33.9, 29.4, 25.9, 18.1, -4.4; ESIHRMS *m/z* calcd for $C_{13}H_{30}NO_3SSi$ $[M+H]^+$ 308.1710, found 308.1712.

2.3. Experimental Procedures and Characterization Data: Synthesis of *O*-Protected-Ynamides

General procedure I: synthesis of *O*-protected-ynamides from bromoalkynes[3]

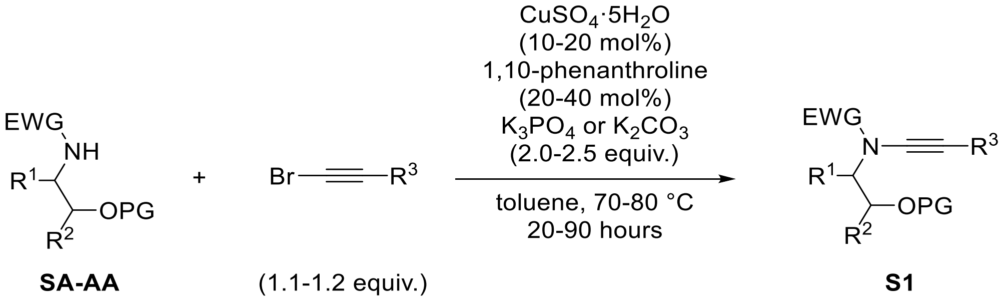

A pressure tube was charged with the nitrogen nucleophile **SA-AA** (1.0 equiv.), potassium phosphate or potassium carbonate (2.0-3.5 equiv.), copper(II) sulfate pentahydrate (10-20 mol%), 1,10-phenanthroline (20-40 mol%) and the bromoalkyne (1.1-1.5 equiv.). The tube was fitted with a rubber septum, evacuated under high vacuum and backfilled with argon three times. Anhydrous toluene (0.33-1.0 M) was next added, the rubber septum was replaced by a Teflon-coated screw cap and the mixture was stirred at the appropriate temperature (70-80 °C) for the appropriate amount of time (20-90 hours). The reaction mixture was then cooled to rt, filtered over a plug of silica (washed with EtOAc) or Celite® (washed with Et_2O) and concentrated under reduced pressure. The crude residue was finally purified by flash column chromatography over silica gel to afford the desired *O*-protected-ynamide **S1**.

General procedure II: synthesis of *O*-protected-ynamides from 1,1-dibromo-1-alkenes[4]

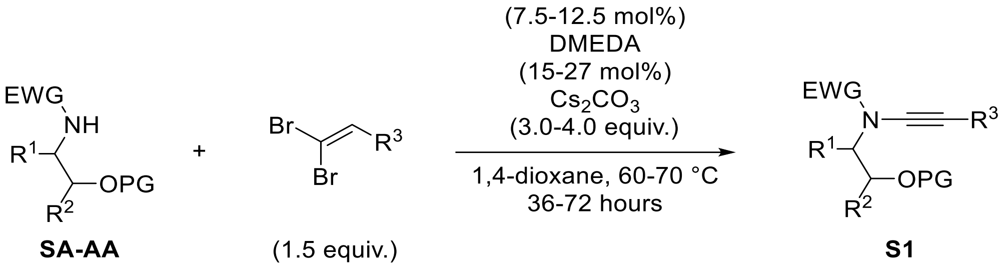

A pressure tube was charged with the nitrogen nucleophile **SA-AA** (1.0 equiv.), the 1,1-dibromo-1-alkene (1.5 equiv.), cesium carbonate (3.2-4.0 equiv.) and copper(I) iodide (7.5-12.5 mol%). The tube was fitted with a rubber septum, evacuated under high vacuum and backfilled with argon three times. Anhydrous 1,4-dioxane (0.5 M) and

N,*N'*-dimethylethylenediamine (DMEDA, 15-27 mol%) were next added, the rubber septum was replaced by Teflon-coated screw cap and the light blue-green suspension was heated at the appropriate temperature (60-70 °C) for the appropriate amount of time (36-72 hours). The reaction mixture was then cooled to rt, filtered over a plug of silica gel (washed with EtOAc) or Celite® (washed with Et_2O) and concentrated under reduced pressure. The crude residue was finally purified by flash column chromatography over silica gel to afford the desired *O*-protected-ynamide **S1**.

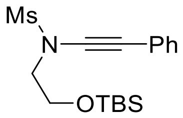

S1a

***N*-{2-[(*tert*-Butyldimethylsilyl)oxy]ethyl}-*N*-(methanesulfonyl)phenylethynylamine S1a**. Prepared according to general procedure I (2.0 mmol of nitrogen nucleophile **SA**, 1.2 equiv. of bromoalkyne, 0.2 equiv. of $CuSO_4 \cdot 5H_2O$, 0.4 equiv. of 1,10-phenanthroline, 2.4 equiv. of K_3PO_4, 0.5 M in toluene, 80 °C, 20 hours, filtered over Celite®). Yield: 76% (535 mg, 1.51 mmol). Solvent system for flash column chromatography: petroleum ether/EtOAc: 90/10; Yellow oil; ^{1}H NMR (400 MHz, $CDCl_3$): δ 7.43-7.38 (m, 2H), 7.32-7.28 (m, 3H), 3.93 (t, J = 5.2 Hz, 2H), 3.70 (t, J = 5.2 Hz, 2H), 3.20 (s, 3H), 0.92 (s, 9H), 0.11 (s, 6H); ^{13}C NMR (100 MHz, $CDCl_3$): δ 131.7, 128.4, 128.1, 122.8, 81.3, 71.3, 60.4, 53.8, 38.9, 26.1, 18.6, -5.2; ESIHRMS *m/z* calcd for $C_{17}H_{28}NO_3SSi$ $[M+H]^+$ 354.1554, found 354.1562.

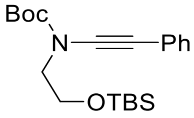

S1b

***N*-*tert*-Butoxycarbonyl-*N*-{2-[(*tert*-butyldimethylsilyl)oxy]ethyl}phenylethynylamine S1b**. Prepared according to general procedure I (10.2 mmol of nitrogen nucleophile, 1.2 equiv. of bromoalkyne, 0.2 equiv. of $CuSO_4 \cdot 5H_2O$, 0.4 equiv. of 1,10-phenanthroline, 2.5 equiv. of K_3PO_4, 0.5 M in toluene, 80 °C, 60 hours, filtered over Celite®). Yield: 60% (2.29 g, 6.1 mmol). Solvent system for flash column chromatography: petroleum ether/EtOAc: 90/10; Yellow oil; ^{1}H NMR (600 MHz, $CDCl_3$): δ 7.35 (br. s, 2H), 7.28 (t, J = 7.4 Hz, 2H), 7.25-7.22 (m, 1H), 3.88 (t, J = 5.9 Hz, 2H), 3.63 (t, J = 5.9 Hz, 2H), 1.53 (s, 9H), 0.90 (s, 9H), 0.09 (s, 6H); ^{13}C NMR (150 MHz,

CDCl3): δ 154.1, 130.7, 128.3, 127.1, 124.1, 84.4, 82.4, 70.1, 60.4, 51.5, 28.2, 26.0, 18.4, -5.3; ESIHRMS *m/z* calcd for $C_{21}H_{34}NO_3Si$ $[M+H]^+$ 376.2302, found 376.2304.

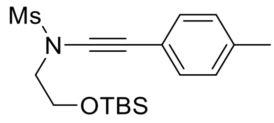

S1c

***N*-{2-[(*tert*-Butyldimethylsilyl)oxy]ethyl}-*N*-(methanesulfonyl)(*p*-tolyl)ethynylamine S1c**. Prepared according to general procedure I (2.0 mmol of nitrogen nucleophile **SA**, 1.1 equiv. of bromoalkyne, 0.2 equiv. of $CuSO_4{\cdot}5H_2O$, 0.4 equiv. of 1,10-phenanthroline, 2.4 equiv. of K_3PO_4, 0.5 M in toluene, 70 °C, 67 hours, filtered over Celite®). Yield: 52% (382 mg, 1.04 mmol). Solvent system for flash column chromatography: petroleum ether/EtOAc: 90/10; Yellow oil; ^{1}H NMR (400 MHz, CDCl3): δ 7.31 (d, *J* = 8.1 Hz, 2H), 7.11 (d, *J* = 8.0 Hz, 2H), 3.93 (t, *J* = 5.2 Hz, 2H), 3.69 (t, *J* = 5.2 Hz, 2H), 3.19 (s, 3H), 2.35 (s, 3H), 0.92 (s, 9H), 0.11 (s, 6H); ^{13}C NMR (100 MHz, CDCl3): δ 138.3, 131.8, 129.2, 119.6, 80.6, 71.2, 60.4, 53.8, 38.8, 26.1, 21.6, 18.6, -5.2; ESIHRMS *m/z* calcd for $C_{18}H_{30}NO_3SSi$ $[M+H]^+$ 368.1710, found 368.1708.

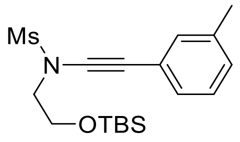

S1d

***N*-{2-[(*tert*-Butyldimethylsilyl)oxy]ethyl}-*N*-(methanesulfonyl)(*m*-tolyl)ethynylamine S1d**. Prepared according to general procedure I (10.0 mmol of nitrogen nucleophile **SA**, 1.1 equiv. of bromoalkyne, 0.2 equiv. of $CuSO_4{\cdot}5H_2O$, 0.4 equiv. of 1,10-phenanthroline, 2.4 equiv. of K_3PO_4, 0.5 M in toluene, 70 °C, 66 hours, filtered over Celite®). Yield: 79% (2.9 g, 7.89 mmol). Solvent system for flash column chromatography: petroleum ether/EtOAc: 90/10; Yellow oil; ^{1}H NMR (400 MHz, CDCl3): δ 7.24 (br. s, 1H), 7.21-7.16 (m, 2H), 7.12-7.09 (m, 1H), 3.93 (t, *J* = 5.2 Hz, 2H), 3.69 (t, *J* = 5.2 Hz, 2H), 3.19 (s, 3H), 2.32 (s, 3H), 0.92 (s, 9H), 0.11 (s, 6H); ^{13}C NMR (100 MHz, CDCl3): δ 138.1, 132.3, 129.0, 128.7, 128.3, 122.5, 81.0, 71.4, 60.4, 53.8,

38.8, 26.1, 21.3, 18.6, -5.2; ESIHRMS *m/z* calcd for $C_{18}H_{30}NO_3SSi$ $[M+H]^+$ 368.1710, found 368.1726.

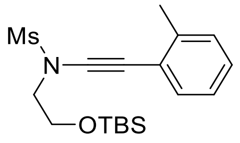

S1e

***N*-{2-[(*tert*-Butyldimethylsilyl)oxy]ethyl}-*N*-(methanesulfonyl)(*o*-tolyl)ethynylamine S1e**. Prepared according to general procedure I (7.50 mmol of nitrogen nucleophile **SA**, 1.1 equiv. of bromoalkyne, 0.2 equiv. of $CuSO_4{\cdot}5H_2O$, 0.4 equiv. of 1,10-phenanthroline, 2.4 equiv. of K_3PO_4, 0.5 M in toluene, 70 °C, 24 hours, filtered over silica gel). Yield: 86% (2.36 g, 6.42 mmol). Solvent system for flash column chromatography: petroleum ether/EtOAc: 90/10; Yellow oil; ^1H NMR (400 MHz, $CDCl_3$): δ 7.36 (d, *J* = 7.6 Hz, 1H), 7.22-7.17 (m, 2H), 7.15-7.10 (m, 1H), 3.95 (t, *J* = 5.2 Hz, 2H), 3.71 (t, *J* = 5.2 Hz, 2H), 3.20 (s, 3H), 2.42 (s, 3H), 0.91 (s, 9H), 0.11 (s, 6H); ^{13}C NMR (100 MHz, $CDCl_3$): δ 139.9, 131.5, 129.6, 128.0, 125.7, 122.6, 85.2, 70.4, 60.4, 53.9, 38.8, 26.0, 20.9, 18.6, -5.3; ESIHRMS *m/z* calcd for $C_{18}H_{30}NO_3SSi$ $[M+H]^+$ 368.1710, found 368.1726.

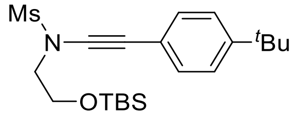

S1f

***N*-{2-[(*tert*-Butyldimethylsilyl)oxy]ethyl}-*N*-(methanesulfonyl)(4-*tert*-butylphenyl)ethynyl amine S1f**. Prepared according to general procedure I (2.0 mmol of nitrogen nucleophile **SA**, 1.1 equiv. of bromoalkyne, 0.2 equiv. of $CuSO_4{\cdot}5H_2O$, 0.4 equiv. of 1,10-phenanthroline, 2.4 equiv. of K_3PO_4, 0.5 M in toluene, 70 °C, 64 hours, filtered over Celite®). Yield: quant. (818 mg, 2.0 mmol). Solvent system for flash column chromatography: petroleum ether/EtOAc: 90/10; Yellow oil; ^1H NMR (400 MHz, $CDCl_3$): δ 7.36 (d, *J* = 8.8 Hz, 2H), 7.32 (d, *J* = 8.8 Hz, 2H), 3.93 (t, *J* = 5.2 Hz, 2H), 3.69 (t, *J* = 5.2 Hz, 2H), 3.18 (s, 3H), 1.31 (s, 9H), 0.92 (s, 9H), 0.11 (s, 6H);

^{13}C NMR (100 MHz, $CDCl_3$): δ 151.6, 131.7, 125.4, 119.6, 80.6, 71.2, 60.4, 53.8, 38.7, 34.9, 31.3, 26.1, 18.6, -5.2; ESIHRMS *m/z* calcd for $C_{21}H_{36}NO_3SSi$ $[M+H]^+$ 410.2180, found 410.2184.

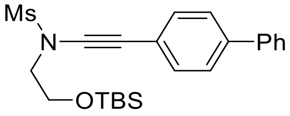

S1g

***N*-{2-[(*tert*-Butyldimethylsilyl)oxy]ethyl}-*N*-(methanesulfonyl)(4-biphenyl)ethynylamine S1g**. Prepared according to general procedure I (4.24 mmol of nitrogen nucleophile **SA**, 1.1 equiv. of bromoalkyne, 0.1 equiv. of $CuSO_4{\cdot}5H_2O$, 0.2 equiv. of 1,10-phenanthroline, 2.0 equiv. of K_2CO_3, 1.0 M in toluene, 80 °C, 48 hours, filtered over silica gel). Yield: 79% (1.43 g, 3.33 mmol). Solvent system for flash column chromatography: petroleum ether/EtOAc: 90/10; Yellow oil; ^1H NMR (400 MHz, $CDCl_3$): δ 7.61-7.57 (m, 2H), 7.55 (d, *J* = 8.4 Hz, 2H), 7.48 (d, *J* = 8.5 Hz, 2H), 7.45 (app. t, *J* = 7.9 Hz, 2H), 7.36 (app. t, *J* = 7.4 Hz, 1H), 3.95 (t, *J* = 5.2 Hz, 2H), 3.72 (t, *J* = 5.2 Hz, 2H), 3.21 (s, 3H), 0.93 (s, 9H), 0.12 (s, 6H); ^{13}C NMR (100 MHz, $CDCl_3$): δ 140.9, 140.5, 132.1, 129.0, 127.8, 127.1 (2C), 121.7, 81.9, 71.2, 60.4, 53.9, 38.9, 26.1, 18.6, -5.2; ESIHRMS *m/z* calcd for $C_{23}H_{31}NO_3SSiK$ $[M+K]^+$ 468.1425, found 468.1432.

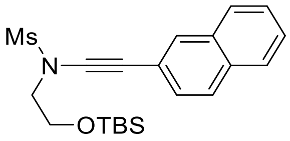

S1h

***N*-{2-[(*tert*-Butyldimethylsilyl)oxy]ethyl}-*N*-(methanesulfonyl)(naphth-3-yl)ethynylamine S1h**. Prepared according to general procedure II (4.0 mmol of nitrogen nucleophile **SA**, 1.5 equiv. of 1,1-dibromo-1-alkene, 0.1 equiv. of CuI, 0.2 equiv. of DMEDA, 4 equiv. of Cs_2CO_3, 0.5 M in 1,4-dioxane, 70 °C, 60 hours, filtered over Celite®). Yield: 79% (1.28 g, 3.17 mmol). Solvent system for flash column chromatography: petroleum ether/EtOAc: 90/10; Yellow oil; ^1H NMR (400 MHz, $CDCl_3$): δ 7.93 (br. s, 1H), 7.83-7.75 (m, 3H), 7.51-7.42 (m, 3H), 3.98 (t, *J* = 5.2 Hz, 2H), 3.74 (t, *J* = 5.2 Hz, 2H), 3.24 (s, 3H), 0.93 (s, 9H), 0.13 (s, 6H); ^{13}C NMR (100 MHz, $CDCl_3$): δ 133.2, 132.8, 131.3, 128.5, 128.1, 127.9, 127.8, 126.7, 126.7,

120.1, 81.7, 71.7, 60.5, 53.9, 38.9, 26.1, 18.6, -5.2; ESIHRMS *m/z* calcd for $C_{21}H_{30}NO_3SSi$ $[M+H]^+$ 404.1710, found 404.1708.

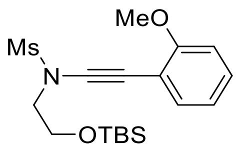

S1i

***N*-{2-[(*tert*-Butyldimethylsilyl)oxy]ethyl}-*N*-(methanesulfonyl)(*o*-methoxyphenyl)ethynyl amine S1i**. Prepared according to general procedure II (2.0 mmol of nitrogen nucleophile **SA**, 1.5 equiv. of 1,1-dibromo-1-alkene, 0.1 equiv. of CuI, 0.2 equiv. of DMEDA, 4 equiv. of Cs_2CO_3, 0.5 M in 1,4-dioxane, 60 °C, 65 hours, filtered over silica gel). Yield: 78% (595 mg, 1.55 mmol). Solvent system for flash column chromatography: petroleum ether/EtOAc: 80/20; Pale yellow oil; ^{1}H NMR (400 MHz, $CDCl_3$): δ 7.36 (dd, *J* = 7.6 and 1.6 Hz, 1H), 7.26 (obs. td, *J* = 7.7 and 1.6 Hz, 1H), 6.88 (m, 2H), 3.96 (t, *J* = 5.3 Hz, 2H), 3.85 (s, 3H), 3.71 (t, *J* = 5.3 Hz, 2H), 3.22 (s, 3H), 0.91 (s, 9H), 0.11 (s, 6H); ^{13}C NMR (100 MHz, $CDCl_3$): δ 160.1, 133.2, 129.5, 120.5, 112.1, 110.7, 85.1, 67.9, 60.4, 55.8, 53.8, 38.6, 26.1, 18.6, -5.3; ESIHRMS *m/z* calcd for $C_{18}H_{30}NO_4SSi$ $[M+H]^+$ 384.1659, found 384.1672.

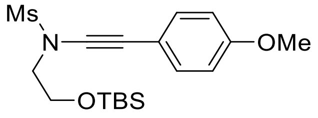

S1j

***N*-{2-[(*tert*-Butyldimethylsilyl)oxy]ethyl}-*N*-(methanesulfonyl)(*p*-methoxyphenyl)ethynyl amine S1j**. Prepared according to general procedure I (7.89 mmol of nitrogen nucleophile **SA**, 1.1 equiv. of bromoalkyne, 0.2 equiv. of $CuSO_4{\cdot}5H_2O$, 0.4 equiv. of 1,10-phenanthroline, 2.0 equiv. of K_2CO_3, 0.5 M in toluene, 80 °C, 60 hours, filtered over silica gel). Yield: 91% (2.76 g, 7.19 mmol). Solvent system for flash column chromatography: petroleum ether/EtOAc: 90/10; Yellow oil; ^{1}H NMR (400 MHz, $CDCl_3$): δ 7.36 (d, *J* = 8.9 Hz, 2H), 6.83 (d, *J* = 8.9 Hz, 2H), 3.92 (t, *J* = 5.2 Hz, 2H), 3.81 (s, 3H), 3.68 (t, *J* = 5.2 Hz, 2H), 3.18 (s, 3H), 0.92 (s, 9H), 0.11 (s, 6H);

^{13}C NMR (100 MHz, $CDCl_3$): δ 159.8, 133.8, 114.6, 114.0, 79.8, 70.8, 60.4, 55.4, 53.8, 38.7, 26.1, 18.6, -5.2; ESIHRMS *m/z* calcd for $C_{18}H_{30}NO_4SSi$ $[M+H]^+$ 384.1659, found 384.1673.

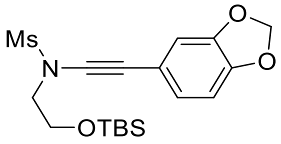

S1k

***N*-{2-[(*tert*-Butyldimethylsilyl)oxy]ethyl}-*N*-(methanesulfonyl)(benzo[*d*][1,3]dioxol-5-yl)ethynylamine S1k**. Prepared according to general procedure II (2.0 mmol of nitrogen nucleophile **SA**, 1.5 equiv. of 1,1-dibromo-1-alkene, 0.1 equiv. of CuI, 0.2 equiv. of DMEDA, 4.0 equiv. of Cs_2CO_3, 0.5 M in 1,4-dioxane, 60 °C, 48 hours, filtered over silica gel). Yield: 83% (660 mg, 1.66 mmol). Solvent system for flash column chromatography: petroleum ether/EtOAc: 90/10; Orange oil; ^1H NMR (400 MHz, $CDCl_3$): δ 6.95 (dd, J = 8.0 and 1.6 Hz, 1H), 6.87 (d, J = 1.5 Hz, 1H), 6.74 (d, J = 8.0 Hz, 1H), 5.97 (s, 2H), 3.91 (t, J = 5.2 Hz, 2H), 3.68 (t, J = 5.2 Hz, 2H), 3.18 (s, 3H), 0.92 (s, 9H), 0.11 (s, 6H); ^{13}C NMR (100 MHz, $CDCl_3$): δ 148.0, 147.6, 126.9, 115.9, 112.2, 108.5, 101.5, 79.6, 71.0, 60.4, 53.8, 38.8, 26.1, 18.6, -5.2; ESIHRMS *m/z* calcd for $C_{18}H_{28}NO_5SSi$ $[M+H]^+$ 398.1452, found 398.1450.

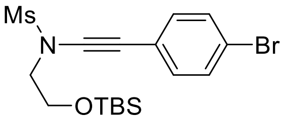

S1l

***N*-{2-[(*tert*-Butyldimethylsilyl)oxy]ethyl}-*N*-(methanesulfonyl)(*p*-bromophenyl)ethynyl amine S1l**. Prepared according to general procedure I (10.0 mmol of nitrogen nucleophile **SA**, 1.1 equiv. of bromoalkyne, 0.2 equiv. of $CuSO_4{\cdot}5H_2O$, 0.4 equiv. of 1,10-phenanthroline, 2.4 equiv. of K_3PO_4, 0.5 M in toluene, 70 °C, 66 hours, filtered over Celite®). Yield: 88% (3.8 g, 8.79 mmol). Solvent system for flash column chromatography: petroleum ether/EtOAc: 90/10; Brown solid; Mp: 52 °C; ^1H NMR (400 MHz, $CDCl_3$): δ 7.43 (d, J = 8.5 Hz, 2H), 7.26 (d, J = 8.5 Hz, 2H), 3.92 (t, J = 5.2 Hz, 2H), 3.69 (t, J = 5.2 Hz, 2H), 3.19 (s, 3H), 0.91 (s, 9H), 0.10 (s, 6H); ^{13}C NMR (100 MHz, $CDCl_3$): δ 133.0, 131.7, 122.2, 121.8, 82.5, 70.4, 60.4, 53.9, 39.0, 26.1, 18.6,

-5.2; ESIHRMS *m/z* calcd for $C_{17}H_{27}{}^{79}BrNO_3SSi$ $[M+H]^+$ 432.0659, found 432.0657, *m/z* calcd for $C_{17}H_{27}{}^{81}BrNO_3SSi$ $[M+H]^+$ 434.0638, found 434.0636.

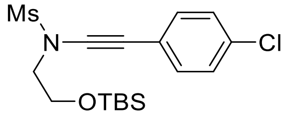

S1m

***N*-{2-[(*tert*-Butyldimethylsilyl)oxy]ethyl}-*N*-(methanesulfonyl)(*p*-chlorophenyl)ethynyl amine S1m**. Prepared according to general procedure I (2.0 mmol of nitrogen nucleophile **SA**, 1.2 equiv. of bromoalkyne, 0.2 equiv. of $CuSO_4{\cdot}5H_2O$, 0.4 equiv. of 1,10-phenanthroline, 4.0 equiv. of K_3PO_4, 0.5 M in toluene, 80 °C, 72 hours, filtered over Celite®). Yield: 64% (495 mg, 1.28 mmol). Solvent system for flash column chromatography: petroleum ether/EtOAc: gradient from 95/5 to 80/20; Yellow oil; ^1H NMR (400 MHz, $CDCl_3$): δ 7.33 (d, *J* = 8.7 Hz, 2H), 7.27 (d, *J* = 8.8 Hz, 2H), 3.92 (t, *J* = 5.2 Hz, 2H), 3.69 (t, *J* = 5.2 Hz, 2H), 3.19 (s, 3H), 0.91 (s, 9H), 0.10 (s, 6H); ^{13}C NMR (100 MHz, $CDCl_3$): δ 134.1, 132.8, 128.8, 121.3, 82.3, 70.2, 60.4, 53.8, 39.0, 26.0, 18.6, -5.3; ESIHRMS *m/z* calcd for $C_{17}H_{27}{}^{35}ClNO_3SSi$ $[M+H]^+$ 388.1164, found 388.1170.

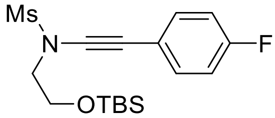

S1n

***N*-{2-[(*tert*-Butyldimethylsilyl)oxy]ethyl}-*N*-(methanesulfonyl)(*p*-fluorophenyl)ethynyl amine S1n**. Prepared according to general procedure I (1.87 mmol of nitrogen nucleophile **SA**, 1.2 equiv. of bromoalkyne, 0.2 equiv. of $CuSO_4{\cdot}5H_2O$, 0.4 equiv. of 1,10-phenanthroline, 2.4 equiv. of K_3PO_4, 0.57 M in toluene, 80 °C, 90 hours, filtered over Celite®). Yield: 65% (455 mg, 1.22 mmol). Solvent system for flash column chromatography: petroleum ether/EtOAc: 90/10; Yellow oil; ^1H NMR (600 MHz, $CDCl_3$): δ 7.39 (app. dd, *J* = 8.9 and 5.4 Hz, 2H), 7.00 (app. t, *J* = 8.7 Hz, 2H), 3.91 (t, *J* = 5.2 Hz, 2H), 3.68 (app. t, *J* = 5.2 Hz, 2H), 3.18 (s, 3H), 0.91 (s, 9H), 0.10 (s, 6H); ^{13}C NMR (150 MHz, $CDCl_3$): δ 162.5 (d, *J* = 247.8 Hz), 133.8 (d, *J* = 8.4 Hz), 118.8 (d, *J* =

3.6 Hz), 115.7 (d, J = 22.1 Hz), 81.0, 70.1, 60.4, 53.8, 38.9, 26.0, 18.5, -5.3; ^{19}F NMR (376 MHz, $CDCl_3$): δ -111.8; ESIHRMS *m/z* calcd for $C_{17}H_{27}FNO_3SSi$ $[M+H]^+$ 372.1459, found 372.1459.

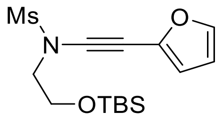

S1o

***N*-{2-[(*tert*-Butyldimethylsilyl)oxy]ethyl}-*N*-(methanesulfonyl)(furan-2-yl)ethynylamine S1o**. Prepared according to general procedure II (2.0 mmol of nitrogen nucleophile **SA**, 1.5 equiv. of 1,1-dibromo-1-alkene, 0.1 equiv. of CuI, 0.2 equiv. of DMEDA, 4.0 equiv. of Cs_2CO_3, 0.5 M in 1,4-dioxane, 60 °C, 65 hours, filtered over silica gel). Yield: 75% (514 mg, 1.50 mmol). Solvent system for flash column chromatography: petroleum ether/EtOAc: 90/10; Orange oil; ^{1}H NMR (400 MHz, $CDCl_3$): δ 7.41 (d, J = 1.8 Hz, 1H), 6.64 (d, J = 3.4 Hz, 1H), 6.39 (dd, J = 3.4 and 1.9 Hz, 1H), 3.90 (t, J = 5.1 Hz, 2H), 3.70 (t, J = 5.1 Hz, 2H), 3.19 (s, 3H), 0.92 (s, 9H), 0.11 (s, 6H); ^{13}C NMR (100 MHz, $CDCl_3$): δ 144.3, 136.8, 117.6, 111.2, 85.5, 62.2, 60.3, 54.0, 39.2, 26.1, 18.6, -5.3; ESIHRMS *m/z* calcd for $C_{15}H_{26}NO_4SSi$ $[M+H]^+$ 344.1346, found 344.1359.

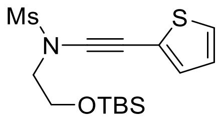

S1p

***N*-{2-[(*tert*-Butyldimethylsilyl)oxy]ethyl}-*N*-(methanesulfonyl)(thiophen-2-yl)ethynylamine S1p**. Prepared according to general procedure II (2.0 mmol of nitrogen nucleophile **SA**, 1.5 equiv. of 1,1-dibromo-1-alkene, 0.1 equiv. of CuI, 0.2 equiv. of DMEDA, 4.0 equiv. of Cs_2CO_3, 0.5 M in 1,4-dioxane, 60 °C, 65 hours, filtered over silica gel). Yield: 80% (573 mg, 1.59 mmol). Solvent system for flash column chromatography: petroleum ether/EtOAc: 90/10; Orange oil; ^{1}H NMR (400 MHz, $CDCl_3$): δ 7.28 (dd, J = 5.2 and 1.1 Hz, 1H), 7.22 (dd, J = 3.6 and 1.2 Hz, 1H), 6.98 (dd, J = 5.2 and 3.6 Hz, 1H), 3.91 (t, J = 5.1 Hz, 2H), 3.70 (t, J = 5.1 Hz, 2H), 3.19 (s, 3H), 0.93 (s, 9H), 0.12 (s, 6H); ^{13}C NMR (100 MHz, $CDCl_3$): δ 133.5, 128.2, 127.1, 122.8, 84.8, 64.5, 60.3, 53.9, 39.1, 26.1, 18.6, -5.2; ESIHRMS *m/z* calcd for $C_{15}H_{26}NO_3S_2Si$ $[M+H]^+$ 360.1118, found 360.1122.

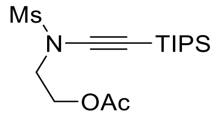

S1q

***N*-(2-Acetoxyethyl)-*N*-(methanesulfonyl)(triisopropylsilyl)ethynylamine S1q**. Prepared according to general procedure I (2.0 mmol of nitrogen nucleophile **SB**, 1.1 equiv. of bromoalkyne, 0.1 equiv. of $CuSO_4{\cdot}5H_2O$, 0.2 equiv. of 1,10-phenanthroline, 2.0 equiv. of K_3PO_4, 0.5 M in toluene, 70 °C, 36 hours, filtered over Celite®). Yield: 40% (287 mg, 794 µmol). Solvent system for flash column chromatography: petroleum ether/EtOAc: 90/10; Yellow oil; ^{1}H NMR (400 MHz, $CDCl_3$): δ 4.33 (t, J = 5.1 Hz, 2H), 3.75 (t, J = 5.2 Hz, 2 H), 3.14 (s, 3H), 2.09 (s, 3H), 1.08 (app. s, 21H); ^{13}C NMR (100 MHz, $CDCl_3$): δ 170.6, 95.2, 70.9, 61.1, 50.2, 38.4, 20.9, 18.8, 11.5; ESIHRMS *m/z* calcd for $C_{16}H_{32}NO_4SSi$ $[M+H]^+$ 362.1816, found 362.1816.

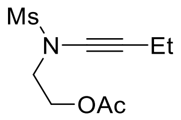

S1r

***N*-(2-Acetoxyethyl)-*N*-(methanesulfonyl)but-1-yn-1-ylamine S1r**. Prepared according to general procedure I (2.5 mmol of nitrogen nucleophile **SB**, 1.1 equiv. of bromoalkyne (0.42 M solution in toluene),* 0.2 equiv. of $CuSO_4{\cdot}5H_2O$, 0.4 equiv. of 1,10-phenanthroline, 2.4 equiv. of K_3PO_4, 0.33 M in toluene, 80 °C, 20 hours, filtered over silica gel). Yield: 27% (170 mg, 672 µmol). Solvent system for flash column chromatography: petroleum ether/EtOAc: 80/20; Orange oil; ^{1}H NMR (400 MHz, $CDCl_3$): δ 4.30 (t, J = 5.2 Hz, 2H), 3.69 (t, J = 5.2 Hz, 2H), 3.09 (s, 3H), 2.30 (q, J = 7.5 Hz, 2H), 2.08 (s, 3H), 1.15 (t, J = 7.5 Hz, 3H); ^{13}C NMR (100 MHz, $CDCl_3$): δ 170.7, 72.7, 71.3, 61.1, 50.0, 38.2, 20.9, 14.2, 12.3; ESIHRMS *m/z* calcd for $C_9H_{16}NO_4S$ $[M+H]^+$ 234.0795, found 234.0800.

* Prepared by dropwise addition of a solution of KHMDS in toluene (0.5 M, 5.5 mL, 2.75 mmol) to a solution of 1,1-dibromobut-1-ene (588 mg, 2.75 mmol) in anhydrous toluene (1 mL) at -78 °C and subsequent stirring of the reaction mixture at -78 °C for 1h.

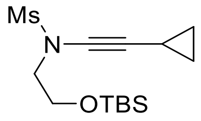

S1s

***N*-{2-[(*tert*-Butyldimethylsilyl)oxy]ethyl}-*N*-(methanesulfonyl)cyclopropylethynylamine S1s**. Prepared according to general procedure II (4.0 mmol of nitrogen nucleophile **SA**, 1.5 equiv. of 1,1-dibromo-1-alkene, 0.1 equiv. of CuI, 0.2 equiv. of DMEDA, 4.0 equiv. of Cs_2CO_3, 0.5 M in 1,4-dioxane, 65 °C, 65 hours, filtered over silica gel). Yield: 73% (921 mg, 2.90 mmol). Solvent system for flash column chromatography: petroleum ether/EtOAc: 90/10; Yellow oil; ^{1}H NMR (400 MHz, $CDCl_3$): δ 3.83 (t, *J* = 5.2 Hz, 2H), 3.54 (t, *J* = 5.2 Hz, 2H), 3.09 (s, 3H), 1.38-1.29 (m, 1H), 0.91 (s, 9H), 0.84-0.77 (m, 2H), 0.71-0.65 (m, 2H), 0.09 (s, 6H); ^{13}C NMR (100 MHz, $CDCl_3$): δ 75.4, 67.9, 60.3, 53.5, 38.3, 26.1, 18.6, 9.0, -0.6, -5.3; ESIHRMS *m/z* calcd for $C_{14}H_{28}NO_3SSi$ $[M+H]^+$ 318.1554, found 318.1555.

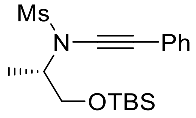

S1t

(*S*)-*N*-{1-[(*tert*-Butyldimethylsilyl)oxy]propan-2-yl}-*N*-(methanesulfonyl)phenylethynyl amine S1t. Prepared according to general procedure II (748 µmol of nitrogen nucleophile **SC**, 1.5 equiv. of 1,1-dibromo-1-alkene, 0.125 equiv. of CuI, 0.187 equiv. of DMEDA, 4.0 equiv. of Cs_2CO_3, 0.5 M in 1,4-dioxane, 70 °C, 48 hours, filtered over Celite®). Yield: 56% (154 mg, 419 µmol). Solvent system for flash column chromatography: petroleum ether/EtOAc: 90/10; Pale yellow oil; $[\alpha]_D^{25}$ + 18 (*c* 1.4, $CHCl_3$); ^{1}H NMR (400 MHz, $CDCl_3$): δ 7.44-7.40 (m, 2H), 7.32-7.28 (m, 3H), 4.20 (dqd, *J* = 9.2, 6.8 and 4.2 Hz, 1H), 3.82 (A of ABX syst., *J* = 10.8 and 4.2 Hz, 1H), 3.59 (B of ABX syst., *J* = 10.8 and 4.2 Hz, 1H), 3.18 (s, 3H), 1.29 (d, *J* = 6.8 Hz, 3H), 0.92 (s, 9H), 0.10 (s, 6H); ^{13}C NMR (100 MHz, $CDCl_3$): δ 131.7, 128.4, 128.0, 123.0, 78.6, 73.2, 64.9, 58.5, 39.6, 26.1, 18.7, 16.1, -5.2, -5.2; ESIHRMS *m/z* calcd for $C_{18}H_{30}NO_3SSi$ $[M+H]^+$ 368.1710, found 368.1712.

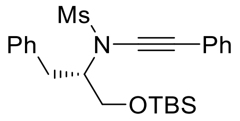

S1u

(*S*)-*N*-{1-[(*tert*-Butyldimethylsilyl)oxy]-3-phenylpropan-2-yl}-*N*-(methanesulfonyl)phenyl ethynylamine S1u. Prepared according to general procedure I (9.8 mmol of nitrogen nucleophile **SD**, 1.1 equiv. of bromoalkyne, 0.2 equiv. of $CuSO_4{\cdot}5H_2O$, 0.4 equiv. of 1,10-phenanthroline, 2.0 equiv. of K_2CO_3, 1 M in toluene, 80 °C, 48 hours, filtered over silica gel). Yield: 46% (2.02 g, 4.55 mmol). Solvent system for flash column chromatography: petroleum ether/EtOAc: 95/5; Yellow oil; $[\alpha]_D^{20}$ - 107 (*c* 1.0, $CHCl_3$); ^{1}H NMR (400 MHz, $CDCl_3$): *δ* 7.49-7.45 (m, 2H), 7.38-7.31 (m, 7H), 7.28-7.22 (obs. m, 1H), 4.29 (ddt, *J* = 10.3, 7.6 and 5.3 Hz, 1H), 3.89 (A of ABX syst., *J* = 10.5 and 7.7 Hz, 1H), 3.74 (B of ABX syst., *J* = 10.5 and 5.5 Hz, 1H), 3.01 (A' of A'B'X syst., *J* = 14.0 and 5.0 Hz, 1H), 2.93 (B' of A'B'X syst., *J* = 14.0 and 10.0 Hz, 1H), 2.51 (s, 3H), 0.92 (s, 9H), 0.11 (s, 3H), 0.10 (s, 3H); ^{13}C NMR (100 MHz, $CDCl_3$): *δ* 137.9, 131.7, 129.7, 128.8, 128.5, 128.2, 127.2, 123.0, 79.0, 73.9, 64.5, 64.1, 38.8, 35.9, 26.1, 18.5, -5.2, -5.3; ESIHRMS *m/z* calcd for $C_{24}H_{34}NO_3SSi$ $[M+H]^+$ 444.2023, found 444.2028.

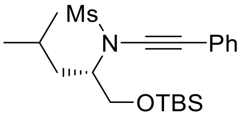

S1v

(*S*)-*N*-{1-[(*tert*-Butyldimethylsilyl)oxy]-4-methylpentan-2-yl}-*N*-(methanesulfonyl)phenyl ethynylamine S1v. Prepared according to general procedure II (38.8 mmol of nitrogen nucleophile **SE**, 1.5 equiv. of 1,1-dibromo-1-alkene, 0.1 equiv. of CuI, 0.2 equiv. of DMEDA, 4.0 equiv. of Cs_2CO_3, 0.5 M in 1,4-dioxane, 60 °C, 60 hours, filtered over Celite®). Yield: 74% (11.7 g, 28.6 mmol). Solvent system for flash column chromatography: petroleum ether/EtOAc: 90/10; Yellow oil; $[\alpha]_D^{20}$ - 5 (*c* 3.0, $CHCl_3$); ^{1}H NMR (400 MHz, $CDCl_3$): *δ* 7.43-7.39 (m, 2H), 7.34-7.28 (m, 3H), 4.15 (ddt, *J* = 10.3, 9.4 and 4.0 Hz, 1H), 3.80 (A of ABX syst., *J* = 10.8 and 9.4 Hz, 1H), 3.58 (B of ABX syst., *J* = 10.8 and 3.9 Hz, 1H), 3.22 (s, 3H), 1.86-1.73 (m, 1H), 1.63 (ddd, *J* = 14.5, 10.3 and 4.4 Hz, 1H), 1.17 (ddd, *J* = 13.7, 9.4 and 4.0 Hz, 1H), 0.98 (d, *J* = 6.7 Hz, 6H), 0.92 (s, 9H), 0.11 (s, 3H), 0.10 (s, 3H); ^{13}C NMR (100 MHz, $CDCl_3$): *δ* 131.7, 128.4, 128.0, 123.1,

78.8, 73.2, 64.5, 61.3, 39.4, 38.4, 26.2, 24.5, 23.6, 21.8, 18.8, -5.2, -5.2; ESIHRMS *m/z* calcd for $C_{21}H_{36}NO_3SSi$ [M+H]$^+$ 410.2180, found 410.2183.

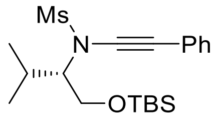

S1w

(*S*)-*N*-{1-[(*tert*-Butyldimethylsilyl)oxy]-3-methylbutan-2-yl}-*N*-(methanesulfonyl)phenyl ethynylamine S1w. Prepared according to general procedure II (1.5 mmol of nitrogen nucleophile **SF**, 1.5 equiv. of 1,1-dibromo-1-alkene, 0.1 equiv. of CuI, 0.2 equiv. of DMEDA, 4.0 equiv. of Cs_2CO_3, 0.5 M in 1,4-dioxane, 60 °C, 36 hours, filtered over silica gel). Yield: 80% (473 mg, 1.20 mmol). Solvent system for flash column chromatography: petroleum ether/EtOAc: 90/10; White solid; Mp: 51 °C; $[\alpha]_D^{20}$ + 30.7 (*c* 0.6, $CHCl_3$); ^1H NMR (400 MHz, $CDCl_3$): *δ* 7.43-7.38 (m, 2H), 7.33-7.26 (m, 3H), 3.90 (app. t, *J* = 10.2 Hz, 1H), 3.83 (dd, *J* = 10.8 and 3.3 Hz, 1H), 3.75 (app. td, *J* = 9.0 and 3.2 Hz, 1H), 3.20 (s, 3H), 1.90 (app. oct., *J* = 7.3 Hz, 1H), 1.09 (d, *J* = 6.7 Hz, 3H), 1.03 (d, *J* = 6.7 Hz, 3H), 0.92 (s, 9H), 0.11 (s, 6H); ^{13}C NMR (100 MHz, $CDCl_3$): *δ* 131.6, 128.4, 127.9, 123.2, 79.7, 73.1, 68.7, 62.4, 39.1, 29.1, 26.2, 20.0, 19.9, 18.8, -5.2; ESIHRMS *m/z* calcd for $C_{20}H_{34}NO_3SSi$ [M+H]$^+$ 396.2023, found 396.2047.

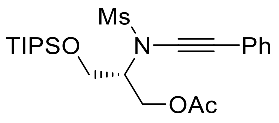

S1x

(*R*)-*N*-{(1-Acetoxy)-3-[(triisopropylsilyl)oxy]propan-2-yl}-*N*-(methanesulfonyl)phenyl ethynylamine S1x. Prepared according to general procedure II (3.8 mmol of nitrogen nucleophile **SI**, 1.5 equiv. of 1,1-dibromo-1-alkene, 0.075 equiv. of CuI, 0.27 equiv. of DMEDA, 3.2 equiv. of Cs_2CO_3, 0.4 M in 1,4-dioxane, 60 °C, 72 hours, filtered over silica gel). Yield: 95% (1.68 g, 3.59 mmol). Solvent system for flash column chromatography: petroleum ether/EtOAc: 90/10; Yellow oil; $[\alpha]_D^{25}$ + 10 (*c* 2.9, $CHCl_3$); ^1H NMR (400 MHz, $CDCl_3$): *δ* 7.42-7.38 (m, 2H), 7.32-7.27 (m, 3H), 4.40-4.31 (m, 2H), 4.30-4.23 (m, 1H), 4.00 (A of ABX syst., *J* = 10.5 and 7.4 Hz, 1H), 3.84 (B of ABX syst., *J* = 10.4 and 5.1 Hz, 1H), 3.20 (s, 3H), 2.10 (s, 3H), 1.17-1.02

(m, 21H); ^{13}C NMR (100 MHz, $CDCl_3$): δ 170.5, 131.7, 128.4, 128.2, 122.5, 78.2, 73.4, 61.8, 61.8, 60.7, 39.2, 20.9, 18.0, 12.0; ESIHRMS *m/z* calcd for $C_{23}H_{38}NO_5SSi$ $[M+H]^+$ 468.2234, found 468.2258.

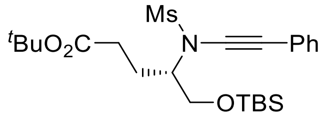

S1y

(*S*)-*N*-{4-[(*tert*-Butyldimethylsilyl)oxy]-1-*tert*-butoxycarbonylbutan-3-yl}-*N*-(methane sulfonyl)phenylethynylamine S1y. Prepared according to general procedure I (330 µmol of nitrogen nucleophile **SL**, 1.5 equiv. of bromoalkyne, 0.1 equiv. of $CuSO_4{\cdot}5H_2O$, 0.2 equiv. of 1,10-phenanthroline, 3.5 equiv. of K_2CO_3, 0.5 M in toluene, 80 °C, 48 hours, filtered over Celite®). Yield: 43% (68 mg, 141 µmol). Solvent system for flash column chromatography: petroleum ether/EtOAc: 85/15; Orange oil; $[\alpha]_D^{25}$ - 14 (*c* 0.7, $CHCl_3$); ^{1}H NMR (400 MHz, $CDCl_3$): δ 7.43-7.38 (m, 2H), 7.33-7.27 (m, 3H), 4.10 (dddd, *J* = 9.5, 8.3, 5.8 and 3.9 Hz, 1H), 3.84 (A of ABX syst., *J* = 10.8 and 9.5 Hz, 1H), 3.66 (B of ABX syst., *J* = 10.8 and 3.9 Hz, 1H), 3.20 (s, 3H), 2.46 (app. dt, *J* = 17.0 and 6.8 Hz, 1H), 2.39 (app. dt, *J* = 17.0 and 7.7 Hz, 1H), 1.84-1.75 (m, 2H), 1.45 (s, 9H), 0.91 (s, 9H), 0.09 (s, 6H); ^{13}C NMR (100 MHz, $CDCl_3$): δ 172.3, 131.7, 128.4, 128.1, 122.9, 80.7, 78.3, 73.3, 64.1, 62.3, 39.4, 31.5, 28.2, 26.1, 24.4, 18.7, -5.2; ESIHRMS *m/z* calcd for $C_{24}H_{39}NO_5SSiNa$ $[M+Na]^+$ 504.2210, found 504.2214.

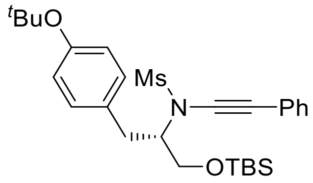

S1z

(*S*)-*N*-{1-[(*tert*-Butyldimethylsilyl)oxy]-3-(4-*tert*-butoxyphenyl)propan-2-yl}-*N*-(methane sulfonyl)phenylethynylamine S1z. Prepared according to general procedure I (2.17 mmol of nitrogen nucleophile **SM**, 1.1 equiv. of bromoalkyne, 0.1 equiv. of $CuSO_4{\cdot}5H_2O$, 0.2 equiv. of 1,10-phenanthroline, 2.0 equiv. of K_2CO_3, 1 M in toluene, 80 °C, 48 hours, filtered over silica gel). Yield: 41% (460 mg, 892 µmol). Solvent system for flash column chromatography:

petroleum ether/EtOAc: 95/5; Yellow oil; $[\alpha]_D^{20}$ - 166 (*c* 0.8, $CHCl_3$); ^1H NMR (400 MHz, $CDCl_3$): δ 7.50-7.44 (m, 2H), 7.38-7.31 (m, 3H), 7.23 (d, *J* = 8.4 Hz, 2H), 6.96 (d, *J* = 8.4 Hz, 2H), 4.24 (ddt, *J* = 10.4, 7.4 and 5.3 Hz, 1H), 3.88 (A of ABX syst., *J* = 10.5 and 7.5 Hz, 1H), 3.72 (B of ABX syst., *J* = 10.5 and 5.6 Hz, 1H), 2.97 (A' of A'B'X syst., *J* = 14.1 and 5.0 Hz, 1H), 2.89 (B' of A'B'X syst., *J* = 14.1 and 10.1 Hz, 1H), 2.52 (s, 3H), 1.32 (s, 9H), 0.91 (s, 9H), 0.09 (s, 3H), 0.09 (s, 3H); ^{13}C NMR (100 MHz, $CDCl_3$): δ 154.5, 132.9, 131.7, 130.1, 128.5, 128.1, 124.7, 123.1, 79.1, 78.7, 73.8, 64.6, 64.1, 38.8, 35.2, 29.0, 26.0, 18.5, -5.2, -5.3; ESIHRMS *m/z* calcd for $C_{28}H_{42}NO_4SSi$ $[M+H]^+$ 516.2598, found 516.2589.

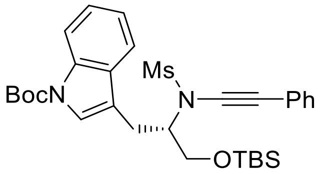

S1aa

(*S*)-*N*-{1-(*N*-*tert*-Butoxycarbonyl-1*H*-indol-3-yl)-3-[(*tert*-butyldimethylsilyl)oxy]propan-2-yl}-*N*-(methanesulfonyl)phenylethynylamine S1aa. Prepared according to general procedure II (1.2 mmol of nitrogen nucleophile **SO**, 1.5 equiv. of 1,1-dibromo-1-alkene, 0.1 equiv. of CuI, 0.2 equiv. of DMEDA, 4.0 equiv. of Cs_2CO_3, 0.5 M in 1,4-dioxane, 60 °C, 72 hours, filtered over Celite®). Yield: 53% (373 mg, 640 µmol). Solvent system for flash column chromatography: petroleum ether/EtOAc: 95/5; Yellow oil; $[\alpha]_D^{20}$ - 80 (*c* 0.88, $CHCl_3$); ^1H NMR (400 MHz, $CDCl_3$): δ 8.15 (d, *J* = 8.0 Hz, 1H), 7.62 (d, *J* = 7.6 Hz, 1H), 7.58 (s, 1H), 7.49-7.44 (m, 2H), 7.37-7.31 (m, 4H), 7.29 (dd, *J* = 7.5 and 1.2 Hz, 1H), 4.40 (tdd, *J* = 7.9, 6.7 and 5.1 Hz, 1H), 3.94 (A of ABX syst., *J* = 10.6 and 7.9 Hz, 1H), 3.77 (B of ABX syst., *J* = 10.6 and 5.1 Hz, 1H), 3.11 (A' of A'B'X syst., *J* = 15.1 and 7.9 Hz, 1H), 3.07 (B' of A'B'X syst., *J* = 15.3 and 6.8 Hz, 1H), 2.78 (s, 3H), 1.65 (s, 9H), 0.91 (s, 9H), 0.10 (s, 3H), 0.09 (s, 3H); ^{13}C NMR (100 MHz, $CDCl_3$): δ 149.7, 135.6, 131.9, 130.2, 128.5, 128.2, 124.8, 124.5, 122.9 (2C), 119.0, 116.2, 115.5, 83.9, 78.8, 73.7, 64.0, 62.1, 39.1, 28.3, 26.1, 25.9, 18.6, -5.2; ESIHRMS *m/z* calcd for $C_{31}H_{43}N_2O_5SSi$ $[M+H]^+$ 583.2656, found 583.2655.

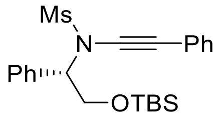

S1ab

(*S*)-*N*-{2-[(*tert*-Butyldimethylsilyl)oxy]-1-phenylethyl}-*N*-(methanesulfonyl)phenylethynyl amine S1ab. Prepared according to general procedure I (10.0 mmol of nitrogen nucleophile **SP**, 1.1 equiv. of bromoalkyne, 0.1 equiv. of $CuSO_4{\cdot}5H_2O$, 0.2 equiv. of 1,10-phenanthroline, 2.0 equiv. of K_2CO_3, 1 M in toluene, 70 °C, 36 hours, filtered over silica gel). Yield: 33% (1.4 g, 3.26 mmol). Solvent system for flash column chromatography: petroleum ether/EtOAc: 95/5; Yellow oil; $[\alpha]_D^{20}$ - 97 (*c* 1.2, $CHCl_3$); ^1H NMR (400 MHz, $CDCl_3$): *δ* 7.49-7.30 (m, 10H), 5.12 (X of ABX syst., *J* = 10.1 and 4.2 Hz, 1H), 4.31 (A of ABX syst., *J* = 10.9 and 10.1 Hz, 1H), 3.92 (B of ABX syst., *J* = 10.9 and 4.2 Hz, 1H), 3.09 (s, 3H), 0.93 (s, 9H), 0.12 (s, 3H), 0.11 (s, 3H); ^{13}C NMR (100 MHz, $CDCl_3$): *δ* 136.6, 131.7, 128.9, 128.8, 128.5, 128.1, 127.4, 123.0, 79.7, 73.8, 65.4, 63.7, 39.4, 26.1, 18.6, -5.1, -5.2; ESIHRMS *m/z* calcd for $C_{23}H_{35}N_2O_3SSi$ $[M+NH_4]^+$ 447.2132, found 447.2120.

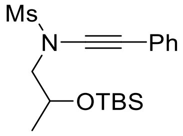

S1ac

***N*-{2-[(*tert*-Butyldimethylsilyl)oxy]propyl}-*N*-(methanesulfonyl)phenylethynylamine S1ac**. Prepared according to general procedure I (4.5 mmol of nitrogen nucleophile **SQ**, 1.0 equiv. of bromoalkyne, 0.1 equiv. of $CuSO_4{\cdot}5H_2O$, 0.2 equiv. of 1,10-phenanthroline, 2.0 equiv. of K_2CO_3, 1.0 M in toluene, 80 °C, 48 hours, filtered over silica gel). Yield: 85% (1.40 g, 3.81 mmol). Solvent system for flash column chromatography: petroleum ether/EtOAc: 90/10; Yellow oil; ^1H NMR (400 MHz, $CDCl_3$): *δ* 7.42-7.37 (m, 2H), 7.33-7.28 (m, 3H), 4.19 (app. sext., *J* = 6.1 Hz, 1H), 3.52 (A of ABX syst., *J* = 13.5 and 6.8 Hz, 1H), 3.44 (B of ABX syst., *J* = 13.5 and 4.9 Hz, 1H), 3.15 (s, 3H), 1.26 (d, *J* = 6.2 Hz, 3H), 0.90 (s, 9H), 0.13 (s, 3H), 0.11 (s, 3H); ^{13}C NMR (100 MHz, $CDCl_3$): *δ* 131.6, 128.4, 128.1, 122.8, 82.5, 70.8, 66.9, 58.8, 38.6, 26.0, 21.4, 18.2, -4.4, -4.6; ESIHRMS *m/z* calcd for $C_{18}H_{30}NO_3SSi$ $[M+H]^+$ 368.1710, found 368.1715.

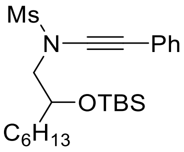

S1ad

***N*-{2-[(*tert*-Butyldimethylsilyl)oxy]octyl}-*N*-(methanesulfonyl)phenylethynylamine S1ad**. Prepared according to general procedure II (1.5 mmol of nitrogen nucleophile **SR**, 1.5 equiv. of 1,1-dibromo-1-alkene, 0.1 equiv. of CuI, 0.2 equiv. of DMEDA, 4.0 equiv. of Cs_2CO_3, 0.5 M in 1,4-dioxane, 70 °C, 48 hours, filtered over silica gel). Yield: 66% (431 mg, 985 µmol). Solvent system for flash column chromatography: petroleum ether/EtOAc: 90/10; Yellow oil; ^{1}H NMR (400 MHz, $CDCl_3$): δ 7.42-7.37 (m, 2H), 7.33-7.28 (m, 3H), 4.02 (app. quint., J = 5.8 Hz, 1H), 3.50 (d, J = 5.9 Hz, 2H), 3.15 (s, 3H), 1.72-1.61 (m, 1H), 1.60-1.49 (m, 1H), 1.43-1.23 (m, 8H), 0.90 (s, 9H), 0.87 (t, J = 7.0 Hz, 3H), 0.14 (s, 3H), 0.10 (s, 3H); ^{13}C NMR (100 MHz, $CDCl_3$): δ 131.6, 128.4, 128.1, 122.8, 82.7, 70.7, 70.6, 57.0, 38.4, 35.1, 31.9, 29.6, 26.0, 24.8, 22.7, 18.2, 14.2, -4.3, -4.6; ESIHRMS *m/z* calcd for $C_{23}H_{40}NO_3SSi$ $[M+H]^+$ 438.2493, found 438.2500.

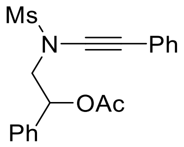

S1ae

***N*-[2-(Acetoxy)-2-phenylethyl]-*N*-(methanesulfonyl)phenylethynylamine S1ae**. Prepared according to general procedure II (1.0 mmol of nitrogen nucleophile **ST**, 1.5 equiv. of 1,1-dibromo-1-alkene, 0.1 equiv. of CuI, 0.2 equiv. of DMEDA, 4.0 equiv. of Cs_2CO_3, 0.5 M in 1,4-dioxane, 70 °C, 48 hours, filtered over silica gel). Yield: 98% (350 mg, 979 µmol). Solvent system for flash column chromatography: petroleum ether/EtOAc: 85/15; Pale yellow oil; ^{1}H NMR (400 MHz, $CDCl_3$): δ 7.46-7.30 (m, 10H), 6.16 (X of ABX syst., J = 8.5 and 4.5 Hz, 1H), 3.98 (A of ABX syst., J = 14.4 and 8.5 Hz, 1H), 3.89 (B of ABX syst., J = 14.4 and 4.6 Hz, 1H), 3.00 (s, 3H), 2.13 (s, 3H); ^{13}C NMR (100 MHz, $CDCl_3$): δ 170.0, 136.8, 131.8, 129.1, 129.0, 128.5, 128.4, 127.0, 122.4, 81.1, 73.1, 71.6, 55.3, 38.9, 21.2; ESIHRMS *m/z* calcd for $C_{19}H_{23}N_2O_4S$ $[M+NH_4]^+$ 375.1373, found 375.1380.

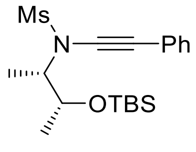

S1af

***N*-{(2*S*,3*R*)-3-[(*tert*-Butyldimethylsilyl)oxy]butan-2-yl}-*N*-(methanesulfonyl)phenylethynyl amine S1af**. Prepared according to general procedure I (3.5 mmol of nitrogen nucleophile **SW**, 1.1 equiv. of bromoalkyne, 0.2 equiv. of $CuSO_4{\cdot}5H_2O$, 0.4 equiv. of 1,10-phenanthroline, 2.0 equiv. of K_2CO_3, 0.5 M in toluene, 80 °C, 72 hours, filtered over Celite®). Yield: 45% (597 mg, 1.56 mmol). Solvent system for flash column chromatography: petroleum ether/EtOAc: 90/10; Yellow oil; $[\alpha]_D^{25}$ + 20 (*c* 1.8, $CHCl_3$); ^1H NMR (400 MHz, $CDCl_3$): δ 7.41-7.38 (m, 2H), 7.33-7.28 (m, 3H), 3.94 (app. quint., *J* = 6.6 Hz, 1H), 3.89 (app. quint., *J* = 6.1 Hz, 1H), 3.17 (s, 3H), 1.39 (d, *J* = 6.6 Hz, 3H), 1.28 (d, *J* = 6.0 Hz, 3H), 0.89 (s, 9H), 0.10 (s, 3H), 0.09 (s, 3H); ^{13}C NMR (100 MHz, $CDCl_3$): δ 131.4, 128.4, 127.9, 123.1, 80.0, 73.1, 71.4, 61.4, 39.5, 26.0, 20.6, 18.2, 15.4, -4.3, -4.7; ESIHRMS *m/z* calcd for $C_{19}H_{32}NO_3SSi$ $[M+H]^+$ 382.1867, found 382.1879.

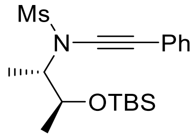

S1af'

***N*-{(2*S*,3*S*)-3-[(*tert*-Butyldimethylsilyl)oxy]butan-2-yl}-*N*-(methanesulfonyl)phenylethynyl amine S1af'**. Prepared according to general procedure I (15.0 mmol of nitrogen nucleophile **SX**, 1.1 equiv. of bromoalkyne, 0.2 equiv. of $CuSO_4{\cdot}5H_2O$, 0.4 equiv. of 1,10-phenanthroline, 2.0 equiv. of K_2CO_3, 0.5 M in toluene, 80 °C, 72 hours, filtered over Celite®). Yield: 11% (633 mg, 1.66 mmol). Solvent system for flash column chromatography: petroleum ether/EtOAc: 90/10; Yellow oil; $[\alpha]_D^{25}$ + 45 (*c* 1.1, $CHCl_3$); ^1H NMR (400 MHz, $CDCl_3$): δ 7.42-7.39 (m, 2H), 7.31-7.28 (m, 3H), 3.95 (app. quint., *J* = 6.2 Hz, 1H), 3.91 (app. quint., *J* = 6.7 Hz, 1H), 3.14 (s, 3H), 1.34 (d, *J* = 6.6 Hz, 3H), 1.22 (d, *J* = 5.9 Hz, 3H), 0.89 (s, 9H), 0.13 (s, 3H), 0.10 (s, 3H); ^{13}C NMR (100 MHz, $CDCl_3$): δ 131.6, 128.4, 128.0, 123.1, 79.8, 73.2, 70.0, 61.7, 39.6, 26.0, 20.4, 18.2, 15.3, -4.1, -4.6; ESIHRMS *m/z* calcd for $C_{19}H_{32}NO_3SSi$ $[M+H]^+$ 382.1867, found 382.1884.

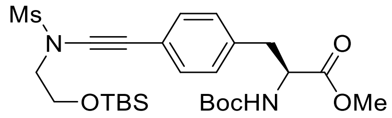

S1ag

(*S*)-*N*-{2-[(*tert*-Butyldimethylsilyl)oxy]ethyl}-*N*-(methanesulfonyl){*p*-{2-[(*tert*-butoxy carbonyl)amino]-2-methoxy carbonylethyl}phenyl}ethynylamine S1ag. Prepared according to general procedure I (2.0 mmol of nitrogen nucleophile **SA**, 1.1 equiv. of bromoalkyne, 0.2 equiv. of $CuSO_4{\cdot}5H_2O$, 0.4 equiv. of 1,10-phenanthroline, 2.0 equiv. of K_2CO_3, 0.2 M in toluene, 80 °C, 60 hours, filtered over silica gel). Yield: 70% (775 mg, 1.40 mmol). Solvent system for flash column chromatography: petroleum ether/EtOAc: 80/20; White solid; Mp: 84 °C; $[\alpha]_D^{20}$ 0 (*c* 0.95, $CHCl_3$); ^1H NMR (400 MHz, $CDCl_3$): δ 7.33 (d, *J* = 8.2 Hz, 2H), 7.06 (d, *J* = 8.1 Hz, 2H), 4.97 (d, *J* = 7.8 Hz, 1H), 4.57 (app. q, *J* = 6.7 Hz, 1H), 3.92 (t, *J* = 5.1 Hz, 2H), 3.70 (s, 3H), 3.68 (obs. t, *J* = 5.2 Hz, 2H), 3.18 (s, 3H), 3.11 (A of ABX syst., *J* = 13.9 and 5.9 Hz, 1H), 3.03 (B of ABX syst., *J* = 13.6 and 5.8 Hz, 1H), 1.41 (s, 9H), 0.91 (s, 9H), 0.10 (s, 6H); ^{13}C NMR (100 MHz, $CDCl_3$): δ 172.3, 155.1, 136.3, 131.9, 129.4, 121.5, 81.4, 80.2, 70.9, 60.4, 54.4, 53.8, 52.4, 38.8, 38.4, 28.4, 26.0, 18.6, -5.3; ESIHRMS *m/z* calcd for $C_{26}H_{46}N_3O_7SSi$ $[M+NH_4]^+$ 572.2820, found 572.2842.

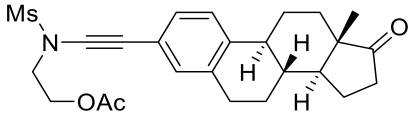

S1ah

(8*R*,9*S*,13*S*,14*S*)-*N*-(2-Acetoxyethyl)-*N*-(methanesulfonyl)-(13-methyl-17-oxo-7,8,9,11,12, 13,14,15,16,17-decahydro-6*H*-cyclopenta[a]phenanthren-3-yl)ethynylamine S1ah. Prepared according to general procedure I (1.12 mmol of nitrogen nucleophile **SB**, 1.1 equiv. of bromoalkyne, 0.2 equiv. of $CuSO_4{\cdot}5H_2O$, 0.4 equiv. of 1,10-phenanthroline, 2.0 equiv. of K_2CO_3, 0.2 M in toluene, 80 °C, 60 hours, filtered over silica gel). Yield: 86% (439 mg, 959 µmol). Solvent system for flash column chromatography: petroleum ether/EtOAc: 70/30; White solid; Mp: 94 °C; $[\alpha]_D^{20}$ + 100 (*c* 1.0, $CHCl_3$); ^1H NMR (400 MHz, $CDCl_3$): δ 7.25-7.16 (m, 3H), 4.38 (t, *J* = 5.0 Hz, 2H), 3.83 (t, *J* = 5.2 Hz, 2H), 3.18 (s, 3H), 2.87 (dd, *J* = 8.9 and 4.3 Hz, 2H), 2.50 (dd, *J* = 19.0 and 8.8 Hz, 1H), 2.44-2.36 (m, 1H), 2.33-2.24 (m, 1H), 2.20-2.10 (m, 1H), 2.10 (s, 3H), 2.09-1.93 (m, 3H), 1.68-1.37 (m, 6H), 0.91 (s, 3H); ^{13}C NMR (100 MHz, $CDCl_3$): δ 220.8, 170.7,

140.5, 136.8, 132.3, 129.1, 125.5, 119.5, 80.2, 71.5, 61.2, 50.6, 50.4, 48.0, 44.5, 38.7, 38.1, 35.9, 31.7, 29.2, 26.4, 25.7, 21.7, 21.0, 13.9; ESIHRMS *m/z* calcd for $C_{25}H_{32}NO_5S$ $[M+H]^+$ 458.1996, found 458.2001.

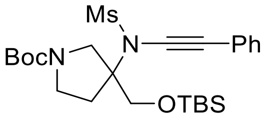

S1ai

***N*-{1-(*tert*-Butoxycarbonyl)-3-{[(tert-butyldimethylsilyl)oxy]methyl}pyrrolidin-3-yl}-*N*-(methanesulfonyl)phenylethynylamine S1ai**. Prepared according to general procedure I (2.0 mmol of nitrogen nucleophile **SZ**, 1.1 equiv. of bromoalkyne, 0.2 equiv. $CuSO_4{\cdot}5H_2O$, 0.4 equiv. of 1,10-phenanthroline, 2.0 equiv. of K_2CO_3, 0.2 M in toluene, 80 °C, 60 hours, filtered over silica gel). Yield: 76% (775 mg, 1.52 mmol). Solvent system for flash column chromatography: petroleum ether/EtOAc: 80/20; Yellow oil; ^1H NMR (400 MHz, $CDCl_3$): δ 7.40-7.36 (m, 2H), 7.31-7.26 (br. m, 3H), 4.00-3.87 (m, 2H), 3.85-3.78 (m, 1H), 3.65 (app. d, J = 12.0 Hz, 1H), 3.62-3.49 (m, 1H), 3.36 (dt, J = 11.0 and 7.6 Hz, 1H), 3.25 (s, 3H), 2.57-2.43 (m, 1H), 2.36-2.25 (m, 1H), 1.44 (s, 9H), 0.92 (s, 9H), 0.10 (s, 6H); ^{13}C NMR (100 MHz, $CDCl_3$): δ 154.3 and 154.2 (rotamers), 131.6 and 131.5 (rotamers), 128.4, 128.1 and 128.0 (rotamers), 122.8, 81.7, 80.1 and 79.8 (rotamers), 73.3 and 73.1 (rotamers), 72.0, 64.2 and 63.8 (rotamers), 52.9 and 52.7 (rotamers), 44.1 and 43.4 (rotamers), 40.1 and 40.0 (rotamers), 33.9 and 33.1 (rotamers), 28.5, 25.8, 18.2, -5.4; ESIHRMS *m/z* calcd for $C_{25}H_{40}N_2O_5SSiNa$ $[M+Na]^+$ 531.2319, found 531.2325.

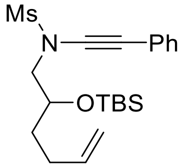

S1aj

***N*-{2-[(*tert*-Butyldimethylsilyl)oxy]hex-5-en-1-yl}-*N*-(methanesulfonyl)phenylethynylamine S1aj**. Prepared according to general procedure II (7.3 mmol of nitrogen nucleophile **SAA**, 1.5 equiv. of 1,1-dibromo-1-alkene, 0.12 equiv. of CuI, 0.18 equiv. of DMEDA, 4.0 equiv. of Cs_2CO_3, 0.5 M in 1,4-dioxane, 70 °C, 48 hours, filtered over silica gel). Yield: 59% (1.75 g, 4.29 mmol).

Solvent system for flash column chromatography: petroleum ether/EtOAc: 95/5; Off-white solid; Mp: 55 °C; ^{1}H NMR (400 MHz, $CDCl_3$): *δ* 7.42-7.37 (m, 2H), 7.34-7.28 (m, 3H), 5.82 (ddt, *J* = 16.8, 10.2 and 6.5 Hz, 1H), 5.04 (dq, *J* = 17.2 and 1.7 Hz, 1H), 4.97 (dq, *J* = 10.2 and 1.4 Hz, 1H), 4.06 (app. quint., *J* = 5.9 Hz, 1H), 3.52 (dd, *J* = 6.0 and 0.9 Hz, 2H), 3.15 (s, 3H), 2.25-2.10 (m, 2H), 1.80 (dddd, *J* = 14.2, 9.0, 6.4 and 5.3 Hz, 1H), 1.65 (dddd, *J* = 13.9, 9.2, 6.9 and 6.0 Hz, 1H), 0.91 (s, 9H), 0.15 (s, 3H), 0.11 (s, 3H); ^{13}C NMR (100 MHz, $CDCl_3$): *δ* 138.2, 131.6, 128.5, 128.2, 122.7, 115.1, 82.6, 70.8, 70.0, 56.9, 38.4, 34.2, 29.1, 26.0, 18.2, -4.2, -4.5; ESIHRMS *m/z* calcd for $C_{21}H_{34}NO_3SSi$ $[M+H]^+$ 408.2023, found 408.2025.

2.4. Experimental Procedures and Characterization Data: Synthesis of Hydroxyethyl-Ynamides

General procedure I: deprotection of OTBS-protected-ynamides

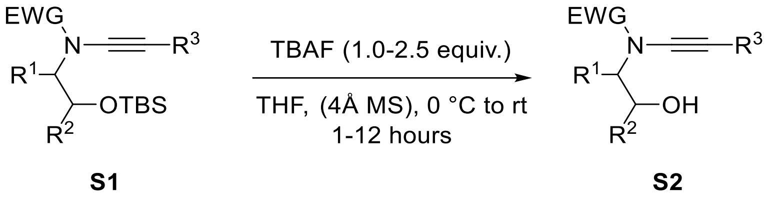

To a solution of OTBS-protected-ynamide **S1** (1.0 equiv.) in anhydrous tetrahydrofuran (0.09-0.60 M), was added a solution of tetrabutylammonium fluoride (1 M solution in tetrahydrofuran, 1.0-2.5 equiv.) dropwise at 0 °C under argon. The resulting mixture was stirred at the appropriate temperature (0 °C to rt) for the appropriate amount of time (1-12 hours), quenched with a saturated aqueous solution of NH_4Cl and extracted with diethyl ether. The combined organic layers were then washed with brine, dried over $MgSO_4$, filtered and concentrated under reduced pressure. The crude residue was finally purified by flash column chromatography over silica gel (unless otherwise stated) to afford the desired hydroxyethyl-ynamide **S2**.

⚠ ***Caution***: *The quality of the TBAF used for the deprotection of OTBS-protected-ynamides had a dramatic impact on the outcome of the reaction. In some cases, addition of 4Å molecular sieves (200 mg/mmol) was beneficial to the reaction and decreased the amount of byproducts observed.*

General procedure II: deprotection of OAc-protected-ynamides

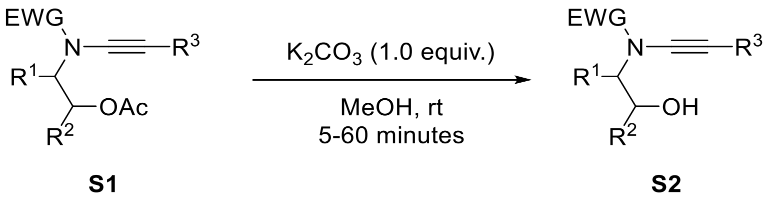

To a solution of OAc-protected-ynamide **S1** (1.0 equiv.) in anhydrous methanol (0.10-0.12 M) was added potassium carbonate (1.0 equiv.) at rt under argon. The resulting mixture was stirred at rt for the appropriate amount of time (5-60 minutes), filtered through a short pad of Celite® (washed with methanol) and concentrated under reduced pressure. The crude

residue was then diluted with a saturated aqueous solution of NH_4Cl and extracted with ethyl acetate. The combined organic layers were then washed with brine, dried over $MgSO_4$, filtered and concentrated under reduced pressure. The crude residue was finally purified by flash column chromatography over silica gel (unless otherwise stated) to afford the desired hydroxyethyl-ynamide **S2**.

Caution*: Overall, hydroxyethyl-ynamides are quite sensitive and may undergo hydrolysis or spontaneous cyclization when stored for prolonged periods of time. They must be engaged as quickly as possible in the following reaction to minimize potential decomposition.*

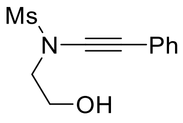

S2a

***N*-(2-Hydroxyethyl)-*N*-(methanesulfonyl)phenylethynylamine S2a**. Prepared according to general procedure I (1.5 mmol of OTBS-protected-ynamide **S1a**, 1.5 equiv. of TBAF, 0.15 M in THF, rt, 3 hours). Yield: 93% (333 mg, 1.39 mmol). Solvent system for flash column chromatography: dichloromethane/EtOAc: 60/40; Pale yellow oil; ^{1}H NMR (400 MHz, $CDCl_3$): δ 7.44-7.38 (m, 2H), 7.33-7.27 (m, 3H), 3.93 (app. q, J = 5.2 Hz, 2H), 3.71 (app. t, J = 5.0 Hz, 2H), 3.21 (s, 3H) 2.46 (t, J = 5.7 Hz, 1H); ^{13}C NMR (100 MHz, $CDCl_3$): δ 131.6, 128.4, 128.2, 122.4, 81.1, 71.3, 59.8, 53.9, 38.5; ESIHRMS *m/z* calcd for $C_{11}H_{14}NO_3S$ $[M+H]^+$ 240.0689, found 240.0693.

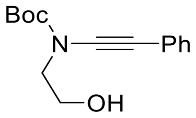

S2b

***N*-*tert*-Butoxycarbonyl-*N*-(2-hydroxyethyl)phenylethynylamine S2b**. Prepared according to general procedure I (1.8 mmol of OTBS-protected-ynamide **S1b**, 1.0 equiv. of TBAF, 0.36 M in THF, rt, 2 hours). Yield: 88% (412 mg, 1.58 mmol). Solvent system for flash column chromatography: petroleum ether/EtOAc: gradient from 90/10 to 70/30; Pale yellow oil; ^{1}H NMR (400 MHz, $CDCl_3$): δ 7.39-7.33 (m, 2H), 7.31-7.22 (m, 3H), 3.91 (br. app. q, J = 4.5 Hz,

2H), 3.70 (t, *J* = 5.3 Hz, 2H), 2.27 (br. s, 1H), 1.53 (s, 9H); ^{13}C NMR (100 MHz, DMSO-d6, 80 °C): *δ* 152.9, 129.8, 128.0, 126.8, 123.0, 84.5, 81.5, 69.1, 57.7, 51.2, 27.3; ESIHRMS *m/z* calcd for $C_{15}H_{20}NO_3$ [M+H]$^+$ 262.1438, found 262.1452.

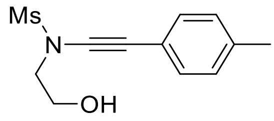

S2c

***N*-(2-Hydroxyethyl)-*N*-(methanesulfonyl)(*p*-tolyl)ethynylamine S2c**. Prepared according to general procedure I (1.1 mmol of OTBS-protected-ynamide **S1c**, 1.5 equiv. of TBAF, 0.11 M in THF, rt, 1 hour). Yield: 75% (210 mg, 829 µmol). Solvent system for flash column chromatography: petroleum ether/EtOAc: 60/40; Yellow solid; Mp: 64 °C; ^{1}H NMR (400 MHz, $CDCl_3$): *δ* 7.31 (d, *J* = 8.1 Hz, 2H), 7.12 (d, *J* = 8.0 Hz, 2H), 3.96 (app. q, *J* = 5.1 Hz, 2H), 3.74 (app. t, *J* = 4.8 Hz, 2H), 3.23 (s, 3H), 2.35 (s, 3H), 1.87 (br. t, *J* = 5.5 Hz, 1H); ^{13}C NMR (100 MHz, $CDCl_3$): *δ* 138.6, 131.8, 129.3, 119.2, 80.4, 71.4, 60.1, 53.9, 38.5, 21.6; ESIHRMS *m/z* calcd for $C_{12}H_{16}NO_3S$ [M+H]$^+$ 254.0845, found 254.0844.

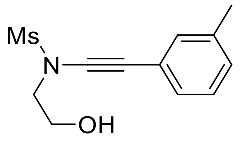

S2d

***N*-(2-Hydroxyethyl)-*N*-(methanesulfonyl)(*m*-tolyl)ethynylamine S2d**. Prepared according to general procedure I (756 µmol of OTBS-protected-ynamide **S1d**, 1.5 equiv. of TBAF, 0.16 M in THF, rt, 2 hours). Yield: 93% (178 mg, 703 µmol). Solvent system for flash column chromatography: petroleum ether/EtOAc: gradient from 80/20 to 50/50; Colorless oil; ^{1}H NMR (400 MHz, $CDCl_3$): *δ* 7.25 (br. s, 1H), 7.24-7.16 (m, 2H), 7.12 (app. br. d, *J* = 7.1 Hz, 1H), 3.96 (app. q, *J* = 5.3 Hz, 2H), 3.74 (t, *J* = 5.1 Hz 2H), 3.23 (s, 3H), 2.32 (s, 3H), 1.98 (t, *J* = 5.7 Hz, 1H); ^{13}C NMR (100 MHz, $CDCl_3$): *δ* 138.2, 132.3, 129.2, 128.7, 128.4, 122.2, 80.7, 71.6, 60.1, 53.9, 38.5, 21.3; ESIHRMS *m/z* calcd for $C_{12}H_{16}NO_3S$ [M+H]$^+$ 254.0845, found 254.0845.

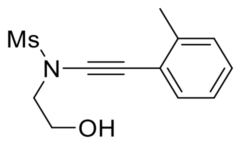

S2e

***N*-(2-Hydroxyethyl)-*N*-(methanesulfonyl)(*o*-tolyl)ethynylamine S2e**. Prepared according to general procedure I (3.3 mmol of OTBS-protected-ynamide **S1e**, 1.5 equiv. of TBAF, 0.16 M in THF, rt, 1 hour). Yield: 98% (820 mg, 3.24 mmol). Solvent system for flash column chromatography: petroleum ether/EtOAc: 50/50; Colorless solid; Mp: 31 °C; ^{1}H NMR (400 MHz, $CDCl_3$): δ 7.36 (d, *J* = 7.5 Hz, 1H), 7.21-7.18 (m, 2H), 7.16-7.10 (m, 1H), 3.96 (br. app. q, *J* = 4.9 Hz, 2H), 3.74 (dd, *J* = 5.6 and 4.4 Hz, 2H), 3.23 (s, 3H), 2.42 (s, 3H), 2.22 (t, *J* = 5.3 Hz, 1H); ^{13}C NMR (100 MHz, $CDCl_3$): δ 139.8, 131.5, 129.6, 128.2, 125.7, 122.2, 84.9, 70.4, 59.9, 53.9, 38.4, 20.9; ESIHRMS *m/z* calcd for $C_{12}H_{16}NO_3S$ $[M+H]^+$ 254.0845, found 254.0844.

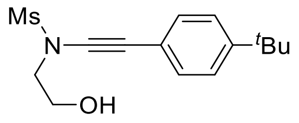

S2f

***N*-(2-Hydroxyethyl)-*N*-(methanesulfonyl)(*p*-*tert*-butylphenyl)ethynylamine S2f**. Prepared according to general procedure I (1.76 mmol of OTBS-protected-ynamide **S1f**, 1.5 equiv. of TBAF, 0.18 M in THF, rt, 2 hours). Yield: 79% (410 mg, 1.39 mmol). Solvent system for flash column chromatography: petroleum ether/EtOAc: gradient from 80/20 to 50/50; Orange oil; ^{1}H NMR (400 MHz, $CDCl_3$): δ 7.37 (d, *J* = 8.8 Hz, 2H), 7.33 (d, *J* = 8.8 Hz, 2H), 3.97 (app. dt, *J* = 5.3 and 4.2 Hz, 2H), 3.74 (app. t, *J* = 5.0 Hz, 2H), 3.23 (s, 3H), 1.82 (t, *J* = 5.7 Hz, 1H), 1.31 (s, 9H); ^{13}C NMR (100 MHz, $CDCl_3$): δ 151.8, 131.7, 125.5, 119.3, 80.4, 71.4, 60.1, 53.9, 38.4, 34.9, 31.3; ESIHRMS *m/z* calcd for $C_{15}H_{22}NO_3S$ $[M+H]^+$ 296.1315, found 296.1312.

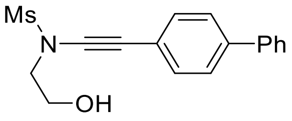

S2g

***N*-(2-Hydroxyethyl)-*N*-(methanesulfonyl)(*p*-biphenyl)ethynylamine S2g.** Prepared according to general procedure I (3.03 mmol of OTBS-protected-ynamide **S1g**, 1.5 equiv. of TBAF, 4Å molecular sieves, 0.20 M in THF, 0 °C, 1 hour). Yield: 88% (840 mg, 2.66 mmol). Solvent system for flash column chromatography: petroleum ether/EtOAc: 60/40; White solid; Mp: 132 °C; ^{1}H NMR (400 MHz, $CDCl_3$): *δ* 7.61-7.55 (m, 2H), 7.55 (d, *J* = 8.3 Hz, 2H), 7.49 (app. d, *J* = 8.3 Hz, 2H), 7.44 (t, *J* = 7.8 Hz, 2H), 7.39 (app. t, *J* = 7.3 Hz, 1H), 3.99 (app. t, *J* = 5.1 Hz, 2H), 3.77 (app. t, *J* = 5.0 Hz, 2H), 3.26 (s, 3H), 1.71 (br. s, 1H); ^{13}C NMR (100 MHz, $CDCl_3$): *δ* 141.1, 140.4, 132.2, 129.0, 127.8, 127.2, 127.1, 121.3, 81.7, 71.4, 60.1, 54.0, 38.6; ESIHRMS *m/z* calcd for $C_{17}H_{18}NO_3S$ $[M+H]^+$ 316.1002, found 316.1013.

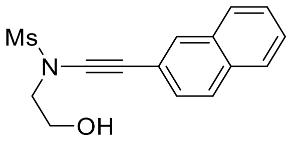

S2h

***N*-(2-Hydroxyethyl)-*N*-(methanesulfonyl)(naphtha-3-yl)ethynylamine S2h.** Prepared according to general procedure I (1.36 mmol of OTBS-protected-ynamide **S1h**, 1.5 equiv. of TBAF, 0.14 M in THF, rt, 1 hour). Yield: 79% (311 mg, 1.07 mmol). Solvent system for flash column chromatography: petroleum ether/EtOAc: 60/40; Off-white solid; Mp: 74 °C; ^{1}H NMR (400 MHz, $CDCl_3$): *δ* 7.94 (s, 1H), 7.83-7.75 (m, 3H), 7.51-7.44 (m, 3H), 4.01 (app. q, *J* = 5.3 Hz, 2H), 3.79 (app. t, *J* = 4.9 Hz, 2H), 3.27 (s, 3H), 1.99 (t, *J* = 5.7 Hz, 1H); ^{13}C NMR (100 MHz, $CDCl_3$): *δ* 133.1, 132.8, 131.4, 128.5, 128.2, 127.9, 127.8, 126.8, 126.8, 119.7, 81.4, 71.9, 60.1, 54.0, 38.7; ESIHRMS *m/z* calcd for $C_{15}H_{16}NO_3S$ $[M+H]^+$ 290.0845, found 290.0842.

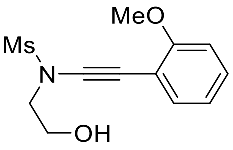

S2i

***N*-(2-Hydroxyethyl)-*N*-(methanesulfonyl)(*o*-methoxyphenyl)ethynylamine S2i**. Prepared according to general procedure I (1.38 mmol of OTBS-protected-ynamide **S1i**, 1.5 equiv. of TBAF, 0.28 M in THF, rt, 2 hours). Yield: 83% (308 mg, 1.14 mmol). Solvent system for flash column chromatography: petroleum ether/EtOAc: gradient from 80/20 to 50/50; Colorless oil; ^1H NMR (400 MHz, $CDCl_3$): δ 7.37 (dd, J = 7.6 and 1.7 Hz, 1H), 7.27 (ddd, J = 8.3, 7.5 and 1.8 Hz, 1H), 6.91 (td, J = 7.5 and 1.0 Hz, 1H), 6.87 (d, J = 8.3 Hz, 1H), 4.04-3.98 (m, 2H), 3.86 (s, 3H), 3.76-3.73 (m, 2H), 3.23 (s, 3H), 2.57 (t, J = 6.4 Hz, 1H); ^{13}C NMR (100 MHz, $CDCl_3$): δ 159.9, 132.8, 129.6, 120.8, 111.7, 110.6, 85.2, 67.9, 60.2, 55.9, 53.7, 38.2; ESIHRMS *m/z* calcd for $C_{12}H_{16}NO_4S$ $[M+H]^+$ 270.0795, found 270.0793.

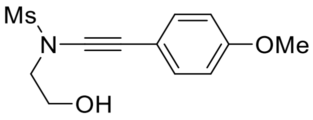

S2j

***N*-(2-Hydroxyethyl)-*N*-(methanesulfonyl)(*p*-methoxyphenyl)ethynylamine S2j**. Prepared according to general procedure I (1.74 mmol of OTBS-protected-ynamide **S1j**, 1.5 equiv. of TBAF, 0.20 M in THF, rt, 1 hour). Yield: 98% (457 mg, 1.70 mmol). Solvent system for flash column chromatography: petroleum ether/EtOAc: 60/40; Pale yellow oil; ^1H NMR (400 MHz, $CDCl_3$): δ 7.37 (d, J = 8.5 Hz, 2H), 6.84 (d, J = 8.6 Hz, 2H), 3.96 (br. app. t, J = 4.9 Hz, 2H), 3.81 (s, 3H), 3.73 (app. t, J = 5.0 Hz, 2H), 3.23 (s, 3H), 1.90 (br. s, 1H); ^{13}C NMR (100 MHz, $CDCl_3$): δ 159.9, 133.8, 114.3, 114.1, 79.7, 71.1, 60.1, 55.5, 54.0, 38.4; ESIHRMS *m/z* calcd for $C_{12}H_{16}NO_4S$ $[M+H]^+$ 270.0795, found 270.0794.

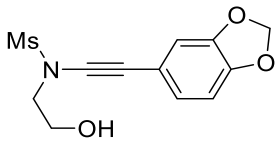

S2k

***N*-(2-Hydroxyethyl)-*N*-(methanesulfonyl)(benzo[*d*][1,3]dioxol-5-yl)ethynylamine S2k**. Prepared according to general procedure I (3.70 mmol of OTBS-protected-ynamide **S1k**, 1.5 equiv. of TBAF, 0.12 M in THF rt, 1 hour). Yield: 89% (938 mg, 3.31 mmol). Solvent system for flash column chromatography: petroleum ether/EtOAc: 70/30; Yellow oil; ^{1}H NMR (400 MHz, $CDCl_3$): δ 6.96 (dd, J = 8.0 and 1.6 Hz, 1H), 6.87 (d, J = 1.6 Hz, 1H), 6.74 (d, J = 8.0 Hz, 1H), 5.97 (s, 2H), 3.95 (app. q, J = 5.2 Hz, 2H), 3.72 (app. t, J = 5.0 Hz, 2H), 3.22 (s, 3H), 1.93 (t, J = 5.7 Hz, 1H); ^{13}C NMR (100 MHz, $CDCl_3$): δ 148.2, 147.6, 126.9, 115.5, 112.2, 108.6, 101.5, 79.4, 71.2, 60.1, 53.9, 38.5; ESIHRMS *m/z* calcd for $C_{12}H_{14}NO_5S$ $[M+H]^+$ 284.0587, found 284.0591.

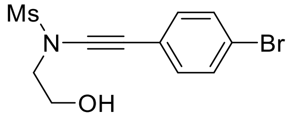

S2l

***N*-(2-Hydroxyethyl)-*N*-(methanesulfonyl)(*p*-bromophenyl)ethynylamine S2l**. Prepared according to general procedure I (925 µmol of OTBS-protected-ynamide **S1l**, 1.5 equiv. of TBAF, 0.09 M in THF, rt, 1 hour). Yield: 88% (260 mg, 817 µmol). Solvent system for flash column chromatography: petroleum ether/EtOAc: gradient 80/20 to 50/50; Yellow solid; Mp: 65 °C; ^{1}H NMR (400 MHz, $CDCl_3$): δ 7.44 (d, J = 8.5 Hz, 2H), 7.27 (d, J = 7.7 Hz, 2H), 3.97 (app. q, J = 5.2 Hz, 2H), 3.75 (app. t, J = 5.0 Hz, 2H), 3.24 (s, 3H), 1.80 (t, J = 5.5 Hz, 1H); ^{13}C NMR (100 MHz, $CDCl_3$): δ 133.0, 131.8, 122.5, 121.5, 82.2, 70.6, 60.1, 53.9, 38.8; ESIHRMS *m/z* calcd for $C_{11}H_{13}{}^{79}BrNO_3S$ $[M+H]^+$ 317.9794, found 317.9795, *m/z* calcd for $C_{11}H_{13}{}^{81}BrNO_3S$ $[M+H]^+$ 319.9774, found 319.9775.

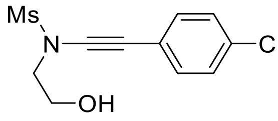

S2m

***N*-(2-Hydroxyethyl)-*N*-(methanesulfonyl)(*p*-chlorophenyl)ethynylamine S2m**. Prepared according to general procedure I (567 µmol of OTBS-protected-ynamide **S1m**, 1.5 equiv. of TBAF, 0.60 M in THF, rt, 2 hours). Yield: 87% (135 mg, 493 µmol). Solvent system for flash column chromatography: petroleum ether/EtOAc: 40/60; Colorless oil; ^{1}H NMR (400 MHz, $CDCl_3$): δ 7.34 (d, J = 8.7 Hz, 2H), 7.28 (d, J = 8.6 Hz, 2H), 3.95 (br. app. q, J = 4.8 Hz, 2H), 3.74 (app. t, J = 5.0 Hz, 2H), 3.23 (s, 3H), 1.98 (br. t, J = 5.3 Hz, 1H); ^{13}C NMR (100 MHz, $CDCl_3$): δ 134.3, 132.9, 128.8, 121.0, 82.0, 70.4, 60.0, 53.9, 38.7; ESIHRMS *m/z* calcd for $C_{11}H_{13}{}^{35}ClNO_3S$ $[M+H]^+$ 274.0299, found 274.0301.

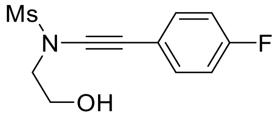

S2n

***N*-(2-Hydroxyethyl)-*N*-(methanesulfonyl)(*p*-fluorophenyl)ethynylamine S2n**. Prepared according to general procedure I (1.34 mmol of OTBS-protected-ynamide **S1n**, 1.5 equiv. of TBAF, 0.13 M in THF, rt, 1 hour). Yield: 97% (334 mg, 1.30 mmol). Solvent system for flash column chromatography: petroleum ether/EtOAc: 60/40; Yellow oil; ^{1}H NMR (400 MHz, $CDCl_3$): δ 7.39 (app. dd, J = 8.9 and 5.4 Hz, 2H), 6.99 (app. t., J = 8.7 Hz, 2H), 3.92 (app. q, J = 4.9 Hz, 2H), 3.71 (app. t, J = 4.9 Hz, 2H), 3.21 (s, 3H), 2.35 (t, J = 5.3 Hz, 1H); ^{13}C NMR (100 MHz, $CDCl_3$): δ 162.6 (d, J = 248.3 Hz), 133.8 (d, J = 8.4 Hz), 118.5 (d, J = 3.6 Hz), 115.8 (d, J = 22.0 Hz), 80.8, 70.3, 60.0, 53.9, 38.6; ^{19}F NMR (376 MHz, $CDCl_3$): δ -111.5; ESIHRMS *m/z* calcd for $C_{11}H_{13}FNO_3S$ $[M+H]^+$ 258.0595, found 258.0597.

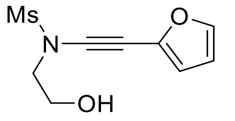

S2o

***N*-(2-Hydroxyethyl)-*N*-(methanesulfonyl)(furan-2-yl)ethynylamine S2o**. Prepared according to general procedure I (1.31 mmol of OTBS-protected-ynamide **S1o**, 1.5 equiv. of TBAF, 0.26 M in THF, rt, 2 hours). Yield: 83% (251 mg, 1.09 mmol). Solvent system for flash column chromatography: petroleum ether/EtOAc: gradient from 80/20 to 50/50; Light brown solid; Mp: 42 °C; ^{1}H NMR (400 MHz, $CDCl_3$): δ 7.41 (d, J = 1.9 Hz, 1H), 6.67 (d, J = 3.4 Hz, 1H), 6.40 (dd, J = 3.4 and 1.8 Hz, 1H), 3.94 (br. app. q, J = 4.6 Hz, 2H), 3.75 (app. t, J = 5.0 Hz, 2H), 3.24 (s, 3H), 1.93 (br. t, J = 4.8 Hz, 1H); ^{13}C NMR (100 MHz, $CDCl_3$): δ 144.5, 136.5, 117.9, 111.3, 85.4, 62.4, 60.0, 54.0, 38.9; ESIHRMS *m/z* calcd for $C_9H_{12}NO_4S$ $[M+H]^+$ 230.0482, found 230.0482.

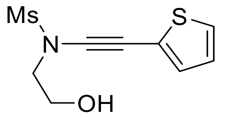

S2p

***N*-(2-Hydroxyethyl)-*N*-(methanesulfonyl)(thiophen-2-yl)ethynylamine S2p**. Prepared according to general procedure I (1.11 mmol of OTBS-protected-ynamide **S1p**, 1.5 equiv. of TBAF, 0.22 M in THF, rt, 2 hours). Yield: 95% (260 mg, 1.06 mmol). Solvent system for flash column chromatography: petroleum ether/EtOAc: gradient from 80/20 to 50/50; Yellow oil; ^{1}H NMR (400 MHz, $CDCl_3$): δ 7.28 (dd, J = 5.2 and 1.2 Hz, 1H), 7.23 (dd, J = 3.6 and 1.2 Hz, 1H), 6.97 (dd, J = 5.2 and 3.6 Hz, 1H), 3.91 (app. q, J = 5.1 Hz, 2H), 3.71 (app. t, J = 5.0 Hz, 2H), 3.22 (s, 3H), 2.42 (t, J = 5.6 Hz, 1H); ^{13}C NMR (100 MHz, $CDCl_3$): δ 133.7, 128.3, 127.2, 122.3, 84.6, 64.6, 59.8, 54.0, 38.7; ESIHRMS *m/z* calcd for $C_9H_{12}NO_3S_2$ $[M+H]^+$ 246.0253, found 246.0252.

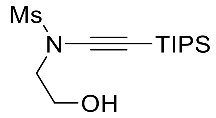

S2q

***N*-(2-Hydroxyethyl)-*N*-(methanesulfonyl)(triisopropylsilyl)ethynylamine S2q**. Prepared according to general procedure II (553 µmol of OAc-protected-ynamide **S1q**, 1.0 equiv. of K_2CO_3, 0.12 M in MeOH, rt, 1 hour). Yield: 51% (90 mg, 282 µmol). Solvent system for flash column chromatography: petroleum ether/EtOAc: 80/20; Colorless oil; ^{1}H NMR (400 MHz, $CDCl_3$): δ 3.92 (app. q, J = 5.1 Hz, 2H), 3.65 (app. t, J = 5.0 Hz, 2H), 3.18 (s, 3H), 1.88 (br. s, 1H), 1.08 (app. s, 21H); ^{13}C NMR (100 MHz, $CDCl_3$): δ 95.4, 70.7, 60.0, 53.6, 38.1, 18.8, 11.5; ESIHRMS *m/z* calcd for $C_{14}H_{30}NO_3SSi$ [M+H]$^+$ 320.1710, found 320.1706.

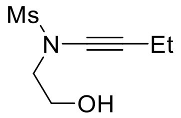

S2r

***N*-(2-Hydroxyethyl)-*N*-(methanesulfonyl)but-1-yn-1-ylamine S2r**. Prepared according to general procedure II (860 µmol of OAc-protected-ynamide **S1r**, 1.0 equiv. of K_2CO_3, 0.10 M in MeOH, rt, 10 minutes). *Due to the instability of hydroxyethyl-ynamide **S2r**, the crude residue was directly used in the next step without further purification (see compound **1r**, page 77).*

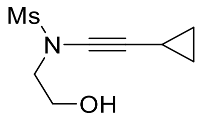

S2s

***N*-(2-Hydroxyethyl)-*N*-(methanesulfonyl)cyclopropylethynylamine S2s**. Prepared according to general procedure I (2.50 mmol of OTBS-protected-ynamide **S1s**, 1.5 equiv. of TBAF, 0.20 M in THF, rt, 2 hours). Yield: 79% (399 mg, 1.96 mmol). Solvent system for flash column chromatography: petroleum ether/EtOAc: 40/60; Yellow oil; ^{1}H NMR (400 MHz, $CDCl_3$): δ 3.87 (br. app. q, J = 4.8 Hz, 2H), 3.59 (app. t, J = 5.0 Hz, 2H), 3.13 (s, 3H), 1.84 (br. s, 1H), 1.33 (tt, J = 8.1 and 4.9 Hz, 1H), 0.85-0.79 (m, 2H), 0.72-0.67 (m, 2H); ^{13}C NMR (100 MHz, $CDCl_3$): δ 75.7,

67.8, 60.1, 53.7, 38.0, 9.1, -0.7; ESIHRMS *m/z* calcd for $C_8H_{14}NO_3S$ $[M+H]^+$ 204.0689, found 204.0689.

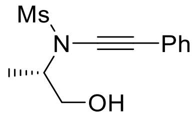

S2t

(*S*)-*N*-(1-Hydroxypropan-2-yl)-*N*-(methanesulfonyl)phenylethynylamine S2t. Prepared according to general procedure I (3.88 mmol of OTBS-protected-ynamide **S1t**, 1.5 equiv. of TBAF, 0.20 M in THF, rt, 2 hours). Yield: 87% (853 mg, 3.37 mmol). Solvent system for flash column chromatography: petroleum ether/EtOAc: 65/35; Colorless oil; $[\alpha]_D^{25}$ + 29 (*c* 3.3, $CHCl_3$); ^1H NMR (400 MHz, $CDCl_3$): δ 7.44-7.40 (m, 2H), 7.33-7.29 (m, 3H), 4.26 (dqd, *J* = 9.4, 6.8 and 3.8 Hz, 1H), 3.82 (ddd, *J* = 11.5, 9.6 and 5.5 Hz, 1H), 3.67 (ddd, *J* = 11.5, 5.3 and 3.9 Hz, 1H), 3.24 (s, 3H), 1.83 (br. t, *J* = 5.7 Hz, 1H), 1.31 (d, *J* = 6.8 Hz, 3H); ^{13}C NMR (100 MHz, $CDCl_3$): δ 131.6, 128.5, 128.2, 122.7, 78.3, 73.6, 64.4, 58.4, 39.4, 15.8; ESIHRMS *m/z* calcd for $C_{12}H_{16}NO_3S$ $[M+H]^+$ 254.0845 found 254.0845.

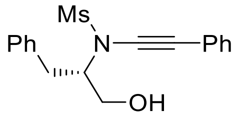

S2u

(*S*)-*N*-(1-Hydroxy-3-phenylpropan-2-yl)-*N*-(methanesulfonyl)phenylethynylamine S2u. Prepared according to general procedure I (3.38 mmol of OTBS-protected-ynamide **S1u**, 1.5 equiv. of TBAF, 0.20 M in THF, rt, 1 hour). Yield: 69% (766 mg, 2.33 mmol). Solvent system for flash column chromatography: petroleum ether/EtOAc: 70/30; Yellow oil; $[\alpha]_D^{20}$ - 187 (*c* 1.0, $CHCl_3$); ^1H NMR (400 MHz, $CDCl_3$): δ 7.52-7.46 (m, 2H), 7.39-7.31 (m, 7H), 7.30-7.23 (m, 1H), 4.37 (tdd, *J* = 9.0, 5.9 and 4.3 Hz, 1H), 3.95-3.86 (m, 1H), 3.84-3.76 (m, 1H), 2.98 (A of ABX syst., *J* = 14.0 and 9.3 Hz, 1H), 2.93 (B of ABX syst., *J* = 14.1 and 5.8 Hz, 1H), 2.50 (s, 3H), 1.91 (br. s, 1H); ^{13}C NMR (100 MHz, $CDCl_3$): δ 137.5, 131.7, 129.6, 128.9, 128.6, 128.4, 127.3, 122.6, 78.4, 74.2, 64.8, 63.5, 38.7, 35.7; ESIHRMS *m/z* calcd for $C_{18}H_{20}NO_3S$ $[M+H]^+$ 330.1158, found 330.1163.

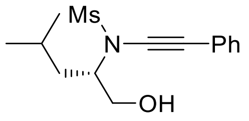

S2v

(*S*)-*N*-(1-Hydroxy-4-methylpentan-2-yl)-*N*-(methanesulfonyl)phenylethynylamine S2v. Prepared according to general procedure I (11.0 mmol of OTBS-protected-ynamide **S1v**, 1.5 equiv. of TBAF, 0.20 M in THF, rt, 1 hour). Yield: 97% (3.17 g, 10.7 mmol). Solvent system for flash column chromatography: petroleum ether/EtOAc: 70/30; Yellow oil; $[\alpha]_D^{20}$ - 23 (*c* 1.1, $CHCl_3$); ^1H NMR (400 MHz, $CDCl_3$): δ 7.43-7.39 (m, 2H), 7.34-7.28 (m, 3H) 4.22 (app. tt, *J* = 10.0 and 3.8 Hz, 1H), 3.79 (br. t, *J* = 10.7 Hz, 1H), 3.67 (br. dd, *J* = 11.7 and 3.4 Hz, 1H), 3.27 (s, 3H), 1.87-1.74 (m, 2H), 1.66 (ddd, *J* = 14.1, 10.1 and 4.4 Hz, 1H), 1.20 (ddd, *J* = 14.2, 9.3 and 4.1 Hz, 1H), 0.98 (d, *J* = 6.7 Hz, 6H); ^{13}C NMR (100 MHz, $CDCl_3$): δ 131.6, 128.5, 128.2, 122.8, 78.5, 73.6, 63.9, 61.2, 39.3, 38.1, 24.6, 23.5, 21.7; ESIHRMS *m/z* calcd for: $C_{15}H_{22}NO_3S$ $[M+H]^+$ 296.1315, found 296.1317.

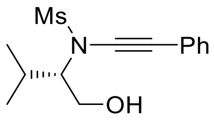

S2w

(*S*)-*N*-(1-Hydroxy-3-methylbutan-2-yl)-*N*-(methanesulfonyl)phenylethynylamine S2w. Prepared according to general procedure I (5.0 mmol of OTBS-protected-ynamide **S1w**, 1.5 equiv. of TBAF, 4Å molecular sieves, 0.20 M in THF, 0 °C, 1 hour). Yield: 99% (1.4 g, 4.98 mmol). Solvent system for flash column chromatography: petroleum ether/EtOAc: 75/25; Yellow oil; $[\alpha]_D^{20}$ - 9.1 (*c* 1.4, $CHCl_3$); ^1H NMR (400 MHz, $CDCl_3$): δ 7.43-7.38 (m, 2H), 7.33-7.27 (m, 3H), 3.94-3.82 (m, 2H), 3.77 (td, *J* = 8.8 and 3.9 Hz, 1H), 3.24 (s, 3H), 2.08 (t, *J* = 5.1 Hz, 1H), 1.92 (dsept., *J* = 8.7 and 6.7 Hz, 1H), 1.08 (d, *J* = 6.8 Hz, 3H), 1.01 (d, *J* = 6.7 Hz, 3H); ^{13}C NMR (100 MHz, $CDCl_3$): δ 131.5, 128.4, 128.1, 122.8, 79.3, 73.3, 68.6, 61.6, 39.0, 28.9, 20.0, 19.7; ESIHRMS *m/z* calcd for $C_{14}H_{20}NO_3S$ $[M+H]^+$ 282.1158, found 282.1160.

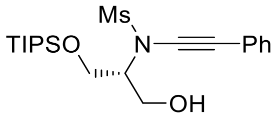

S2x

(*R*)-*N*-{1-Hydroxy-3-[(triisopropylsilyl)oxy]propan-2-yl}-*N*-(methanesulfonyl)phenylethynyl amine S2x. Prepared according to general procedure II (111 µmol of OAc-protected-ynamide **S1x**, 1.0 equiv. of K_2CO_3, 0.10 M in MeOH, rt, 5 minutes). ***Caution:*** *Due to the instability of hydroxyethyl-ynamide* ***S2x****, no aqueous workup was performed after the reaction: the reaction mixture was immediately concentrated under reduced pressure and purified by flash column chromatography*. Yield: 53% (25 mg, 59 µmol). Solvent system for flash column chromatography: petroleum ether/EtOAc: 80/20; Pale yellow oil; $[\alpha]_D^{25}$ - 23 (*c* 2.0, $CHCl_3$); ^1H NMR (400 MHz, $CDCl_3$): *δ* 7.43-7.38 (m, 2H), 7.33-7.29 (m, 3H), 4.20 (app. tt, *J* = 7.4 and 5.6 Hz, 1H), 4.02 (A of ABX syst., *J* = 10.5 and 7.5 Hz, 1H), 3.91 (app. dd, *J* = 5.9 and 2.9 Hz, 1H), 3.89 (app. dd, *J* = 5.8 and 1.1 Hz, 1H), 3.86 (B of ABX syst., *J* = 10.5 and 5.6 Hz, 1H), 3.24 (s, 3H), 1.84 (t, *J* = 5.9 Hz, 1H), 1.12-1.05 (m, 21H); ^{13}C NMR (100 MHz, $CDCl_3$): *δ* 131.7, 128.5, 128.3, 122.7, 78.8, 73.2, 63.9, 62.2, 61.5, 39.1, 18.1, 12.0; ESIHRMS *m/z* calcd for $C_{21}H_{36}NO_4SSi$ $[M+H]^+$ 426.2129, found 426.2129.

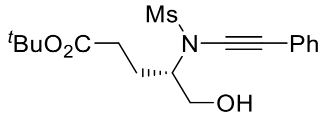

S2y

(*S*)-*N*-(4-Hydroxy-1-*tert*-butoxycarbonylbutan-3-yl)-*N*-(methanesulfonyl)phenylethynyl amine S2y. Prepared according to general procedure I (216 µmol of OTBS-protected-ynamide **S1y**, 1.5 equiv. of TBAF, 4Å molecular sieves, 0.20 M in THF, rt, 4 hours). Yield: 94% (75 mg, 204 µmol). Solvent system for flash column chromatography: petroleum ether/EtOAc: 60/40; Yellow oil; $[\alpha]_D^{25}$ - 5 (*c* 1.6, $CHCl_3$); ^1H NMR (400 MHz, $CDCl_3$): *δ* 7.44-7.38 (m, 2H), 7.33-7.28 (m, 3H), 4.20-4.10 (m, 1H), 3.84 (ddd, *J* = 11.4, 9.5 and 5.3 Hz, 1H), 3.73 (app. dt, *J* = 11.6 and 4.0 Hz, 1H), 3.26 (s, 3H), 2.46 (app. dt, *J* = 16.9 and 6.9 Hz, 1H), 2.39 (app. dt, *J* = 16.9 and 7.7 Hz, 1H), 2.06 (br. t, *J* = 5.4 Hz, 1H), 1.82 (app. q, *J* = 7.3 Hz, 2H), 1.45 (s, 9H); ^{13}C NMR (100 MHz,

$CDCl_3$): δ 172.3, 131.6, 128.5, 128.2, 122.6, 81.0, 78.1, 73.6, 63.4, 62.1, 39.3, 31.4, 28.2, 24.3; ESIHRMS *m/z* calcd for $C_{18}H_{26}NO_5S$ $[M+H]^+$ 368.1526, found 368.1527.

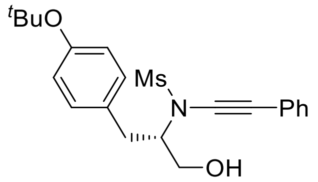

S2z

(*S*)-*N*-[1-Hydroxy-3-(4-*tert*-butoxyphenyl)propan-2-yl]-*N*-(methanesulfonyl)phenylethynyl amine S2z. Prepared according to general procedure I (1.11 mmol of OTBS-protected-ynamide **S1z**, 1.5 equiv. of TBAF, 4Å molecular sieves, 0.20 M in THF, 0 °C, 1 hour). Yield: 70% (310 mg, 772 µmol). Solvent system for flash column chromatography: petroleum ether/EtOAc: 50/50; Colorless oil; $[\alpha]_D^{20}$ - 118 (*c* 1.7, $CHCl_3$); ^1H NMR (400 MHz, $CDCl_3$): δ 7.50-7.46 (m, 2H), 7.36-7.34 (m, 3H), 7.24 (d, *J* = 8.4 Hz, 2H), 6.97 (d, *J* = 8.4 Hz, 2H), 4.37-4.26 (m, 1H), 3.89 (A of ABX syst., *J* = 11.7 and 8.6 Hz, 1H), 3.79 (B of ABX syst., *J* = 11.7 and 4.3 Hz, 1H), 2.93 (A' of A'B'X syst., *J* = 14.1 and 9.6 Hz, 1H), 2.89 (B' of A'B'X syst., *J* = 14.2 and 5.7 Hz, 1H), 2.50 (s, 3H), 1.74 (br. s, 1H), 1.32 (s, 9H); ^{13}C NMR (100 MHz, $CDCl_3$): δ 154.7, 132.3, 131.7, 130.1, 128.6, 128.4, 124.7, 122.7, 78.7, 78.4, 74.2, 64.9, 63.6, 38.7, 35.0, 29.0; ESIHRMS *m/z* calcd for $C_{22}H_{28}NO_4S$ $[M+H]^+$ 402.1734, found 402.1718.

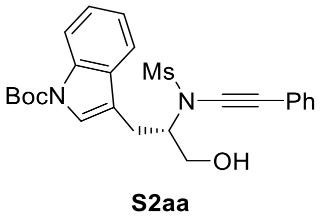

S2aa

(*S*)-*N*-[1-(*N*-*tert*-Butoxycarbonyl-1*H*-indol-3-yl)-3-hydroxypropan-2-yl]-*N*-(methanesulfonyl) phenylethynylamine S2aa. Prepared according to general procedure I (789 µmol of OTBS-protected-ynamide **S1aa**, 2.0 equiv. of TBAF, 0.20 M in THF, rt, 3.5 hours). Yield: 65% (239 mg, 510 µmol). Solvent system for flash column chromatography: petroleum ether/EtOAc: 70/30; Colorless oil; $[\alpha]_D^{20}$ - 105 (*c* 0.40, $CHCl_3$); ^1H NMR (400 MHz, $CDCl_3$): δ 8.15 (d, *J* = 8.1 Hz, 1H), 7.62 (d, *J* = 7.8 Hz, 1H), 7.58 (s, 1H), 7.50-7.45 (m, 2H), 7.38-7.32 (m, 4H), 7.29 (dd, *J* = 7.7 and

1.0 Hz, 1H), 4.47 (tdd, *J* = 8.7, 6.5 and 4.1 Hz, 1H), 3.94 (A of ABX syst., *J* = 11.7 and 8.7 Hz, 1H), 3.82 (B of ABX syst., *J* = 11.6 and 4.1 Hz, 1H), 3.15 (A' of A'B'X syst., *J* = 15.1 and 8.7 Hz, 1H), 3.02 (B' of A'B'X syst., *J* = 15.0 and 6.5 Hz, 1H), 2.82 (s, 3H), 1.91 (br. s, 1H), 1.65 (s, 9H); ^{13}C NMR (100 MHz, $CDCl_3$): *δ* 149.7, 135.6, 131.9, 130.1, 128.6, 128.4, 124.9, 124.6, 123.0, 122.6, 119.0, 115.8, 115.6, 84.0, 78.4, 74.1, 63.3, 62.3, 39.0, 28.3, 25.9; ESIHRMS *m/z* calcd for $C_{25}H_{28}N_2O_5SNa$ $[M+Na]^+$ 491.1611, found 491.1614.

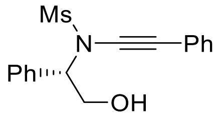

S2ab

(*S*)-*N*-(2-Hydroxy-1-phenylethyl)-*N*-(methanesulfonyl)phenylethynylamine S2ab. Prepared according to general procedure I (4.0 mmol of OTBS-protected-ynamide **S1ab**, 1.5 equiv. of TBAF, 0.20 M in THF, 0 °C, 1 hour). Yield: 79% (999 mg, 3.17 mmol). Solvent system for flash column chromatography: petroleum ether/EtOAc: 70/30; Pale yellow oil; $[\alpha]_D^{20}$ - 153 (*c* 1.0, $CHCl_3$); ^{1}H NMR (400 MHz, $CDCl_3$): *δ* 7.50-7.45 (m, 2H), 7.45-7.36 (m, 5H), 7.35-7.30 (m, 3H), 5.17 (dd, *J* = 10.1 and 4.4 Hz, 1H), 4.34 (ddd, *J* = 11.9, 10.1 and 5.4 Hz, 1H), 3.97 (ddd, *J* = 11.9, 6.8 and 4.4 Hz, 1H), 3.00 (s, 3H), 2.13 (dd, *J* = 6.7 and 5.5 Hz, 1H); ^{13}C NMR (100 MHz, $CDCl_3$): *δ* 135.9, 131.6, 129.2, 129.1, 128.6, 128.4, 127.6, 122.5, 79.3, 74.0, 65.3, 63.0, 39.3; ESIHRMS *m/z* calcd for $C_{17}H_{18}NO_3S$ $[M+H]^+$ 316.1002, found 316.0998.

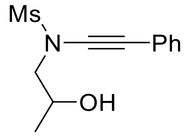

S2ac

***N*-(2-Hydroxypropyl)-*N*-(methanesulfonyl)phenylethynylamine S2ac**. Prepared according to general procedure I (1.49 mmol of OTBS-protected-ynamide **S1ac**, 2.5 equiv. of TBAF, 0.20 M in THF, rt, 12 hours). Yield: 82% (311 mg, 1.23 mmol). Solvent system for flash column chromatography: petroleum ether/EtOAc: 70/30; Colorless oil; ^{1}H NMR (400 MHz, $CDCl_3$): *δ* 7.44-7.39 (m, 2H), 7.33-7.28 (m, 3H), 4.30-4.20 (m, 1H), 3.64 (A of ABX syst., *J* = 14.0 and 8.6

Hz, 1H), 3.49 (B of ABX syst., J = 14.0 and 3.2 Hz, 1H), 3.23 (s, 3H), 1.96 (br. s, 1H), 1.30 (d, J = 6.4 Hz, 3H); ^{13}C NMR (100 MHz, $CDCl_3$): δ 131.7, 128.5, 128.3, 122.4, 81.8, 71.2, 66.1, 58.7, 38.6, 20.6; ESIHRMS m/z calcd for $C_{12}H_{16}NO_3S$ $[M+H]^+$ 254.0845, found 254.0845.

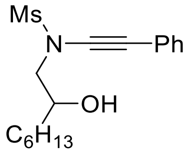

S2ad

***N*-(2-Hydroxyoctyl)-*N*-(methanesulfonyl)phenylethynylamine S2ad**. Prepared according to general procedure I (457 µmol of OTBS-protected-ynamide **S1ad**, 1.5 equiv. of TBAF, 4Å molecular sieves, 0.20 M in THF, 0 °C, 1 hour). Yield: 92% (136 mg, 421 µmol). Solvent system for flash column chromatography: petroleum ether/EtOAc: 75/25; Yellow oil; ^{1}H NMR (400 MHz, $CDCl_3$): δ 7.42-7.38 (m, 2H), 7.32-7.27 (m, 3H), 4.07-3.97 (m, 1H), 3.64 (A of ABX syst., J = 14.1 and 8.7 Hz, 1H), 3.50 (B of ABX syst., J = 14.1 and 3.1 Hz, 1H), 3.23 (s, 3H), 2.10 (br. s, 1H), 1.58-1.42 (m, 3H), 1.40-1.23 (m, 7H), 0.87 (app. t, J = 7.0 Hz, 3H); ^{13}C NMR (100 MHz, $CDCl_3$): δ 131.7, 128.5, 128.3, 122.5, 81.8, 71.2, 69.9, 57.6, 38.6, 34.5, 31.8, 29.3, 25.5, 22.7, 14.2; ESIHRMS m/z calcd for $C_{17}H_{26}NO_3S$ $[M+H]^+$ 324.1628, found 324.1626.

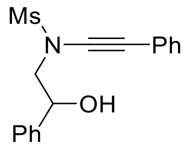

S2ae

***N*-(2-Hydroxy-2-phenylethyl)-*N*-(methanesulfonyl)phenylethynylamine S2ae**. Prepared according to general procedure II (560 µmol of OAc-protected-ynamide **S1ae**, 1.0 equiv. of K_2CO_3, 0.10 M in MeOH, rt, 1 hour). *Due to the instability of hydroxyethyl-ynamide **S2ae**, the crude residue was directly used in the next step without further purification (see compound **S3ae**, page 66).*

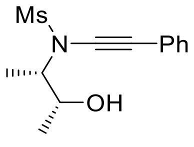

S2af

***N*-[(2*S*,3*R*)-3-Hydroxybutan-2-yl]-*N*-(methanesulfonyl)phenylethynylamine S2af**. Prepared according to general procedure I (1.52 mmol of OTBS-protected-ynamide **S1af**, 1.5 equiv. of TBAF, 0.20 M in THF, rt, 1.5 hours). Yield: 91% (373 mg, 1.39 mmol). Solvent system for flash column chromatography: petroleum ether/EtOAc: 60/40; Yellow oil; $[\alpha]_D^{25}$ + 16 (*c* 1.0, $CHCl_3$); ^1H NMR (400 MHz, $CDCl_3$): δ 7.42-7.39 (m, 2H), 7.33-7.29 (m, 3H), 4.07-3.96 (m, 2H), 3.21 (s, 3H), 1.88 (d, *J* = 4.4 Hz, 1H), 1.44 (d, *J* = 6.6 Hz, 3H), 1.33 (d, *J* = 6.2 Hz, 3H); ^{13}C NMR (100 MHz, $CDCl_3$): δ 131.5, 128.5, 128.2, 122.7, 79.8, 73.3, 71.1, 60.7, 39.4, 19.4, 14.5; ESIHRMS *m/z* calcd for $C_{13}H_{18}NO_3S$ $[M+H]^+$ 268.1002, found 268.1019.

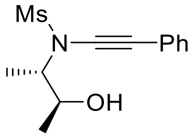

S2af'

***N*-[(2*S*,3*S*)-3-Hydroxybutan-2-yl]-*N*-(methanesulfonyl)phenylethynylamine S2af'**. Prepared according to general procedure I (331 µmol of OTBS-protected-ynamide **S1af'**, 1.5 equiv. of TBAF, 0.20 M in THF, rt, 1.5 hours). Yield: 73% (65 mg, 243 µmol). Solvent system for flash column chromatography: petroleum ether/EtOAc: 60/40; Yellow oil; $[\alpha]_D^{25}$ + 22 (*c* 0.6, $CHCl_3$); ^1H NMR (400 MHz, $CDCl_3$): δ 7.44-7.40 (m, 2H), 7.33-7.29 (m, 3H), 3.98-3.88 (m, 2H), 3.22 (s, 3H), 1.98 (d, *J* = 4,8 Hz, 1H), 1.35 (d, *J* = 6.5 Hz, 3H), 1.29 (d, *J* = 5.9 Hz, 3H); ^{13}C NMR (100 MHz, $CDCl_3$): δ 131.6, 128.5, 128.2, 122.7, 78.9, 73.5, 69.3, 62.8, 39.2, 20.5, 16.4; ESIHRMS *m/z* calcd for $C_{13}H_{18}NO_3S$ $[M+H]^+$ 268.1002, found 268.1014.

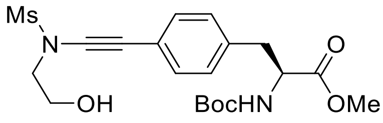

S2ag

(*S*)-*N*-(2-Hydroxyethyl)-*N*-(methanesulfonyl){*p*-{2-[(tert-butoxycarbonyl)amino]-2-methoxy carbonylethyl}phenyl}ethynylamine S2ag. Prepared according to general procedure I (1.0 mmol of OTBS-protected-ynamide **S1ag**, 1.5 equiv. of TBAF, 4Å molecular sieves, 0.2 M in THF, 0 °C, 1 hour). Yield: 80% (354 mg, 804 µmol). Solvent system for flash column chromatography: petroleum ether/Et_2O: 10/90; Colorless oil; $[\alpha]_D^{20}$ + 10 (*c* 1.0, $CHCl_3$); ^1H NMR (400 MHz, $CDCl_3$): δ 7.33 (d, *J* = 8.2 Hz, 2H), 7.06 (d, *J* = 8.1 Hz, 2H), 4.99 (d, *J* = 8.0 Hz, 1H), 4.55 (app. q, *J* = 6.4 Hz, 1H), 3.94 (app. q, *J* = 5.2 Hz, 2H), 3.72 (t, *J* = 5.2 Hz, 2H), 3.70 (s, 3H), 3.22 (s, 3H), 3.10 (A of ABX syst., *J* = 13.8 and 6.0 Hz, 1H), 3.02 (B of ABX syst., *J* = 13.0 and 6.1 Hz, 1H), 2.24-2.14 (m, 1H), 1.41 (s, 9H); ^{13}C NMR (100 MHz, $CDCl_3$): δ 172.3, 155.2, 136.5, 131.9, 129.4, 121.2, 81.3, 80.2, 71.1, 59.9, 54.4, 53.9, 52.4, 38.5, 38.3, 28.4; ESIHRMS *m/z* calcd for $C_{20}H_{28}N_2O_7SNa$ $[M+Na]^+$ 463.1509, found 463.1531.

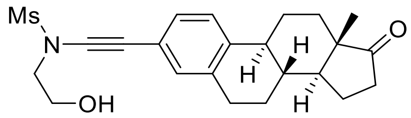

S2ah

(8*R*,9*S*,13*S*,14*S*)-*N*-(2-hydroxyethyl)-*N*-(methanesulfonyl)-(13-methyl-17-oxo-7,8,9,11,12, 13,14,15,16,17-decahydro-6*H*-cyclopenta[a]phenanthren-3-yl)ethynylamine S2ah. Prepared according to general procedure II (642 µmol of OAc-protected-ynamide **S1ah**, 1.0 equiv. of K_2CO_3, 0.1 M in MeOH, rt, 1.5 hours, extractions performed with CH_2Cl_2 instead of EtOAc). Yield: 92% (245 mg, 590 µmol). Purification by precipitation/trituration in CH_2Cl_2/pentane; White solid; Mp: 208 °C; $[\alpha]_D^{20}$ + 114 (*c* 1.0, $CHCl_3$); ^1H NMR (400 MHz, $CDCl_3$): δ 7.25-7.17 (m, 3H), 3.99-3.93 (br. m, 2H), 3.74 (dd, *J* = 5.2 and 4.2 Hz, 2H), 3.22 (s, 3H), 2.88 (dd, *J* = 8.9 and 4.3 Hz, 2H), 2.51 (dd, *J* = 19.1 and 8.7 Hz, 1H), 2.44-2.37 (m, 1H), 2.33-2.24 (m, 1H), 2.21-2.09 (m, 1H), 2.07-1.93 (m, 3H), 1.91 (br. s, 1H), 1.69-1.38 (m, 6H), 0.91 (s, 3H); ^{13}C NMR (100 MHz, $CDCl_3$): δ 220.9, 140.4, 136.8, 132.3, 129.1, 125.5, 119.7, 80.5, 71.4, 60.1, 53.9, 50.6, 48.1, 44.5, 38.5, 38.1, 36.0, 31.7, 29.2, 26.4, 25.7, 21.7, 14.0; ESIHRMS *m/z* calcd for $C_{23}H_{30}NO_4S$ $[M+H]^+$ 416.1890, found 416.1899.

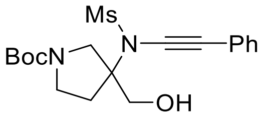

S2ai

***N*-{1-(*tert*-Butoxycarbonyl)-3-(hydroxymethyl)pyrrolidin-3-yl}-*N*-(methanesulfonyl)phenyl ethynylamine S2ai**. Prepared according to general procedure I (255 µmol of OTBS-protected-ynamide **S1ai**, 1.5 equiv. of TBAF, 4Å molecular sieves, 0.20 M in THF, 0 °C, 1 hour). Yield: 71% (72 mg, 182 µmol). Solvent system for flash column chromatography: pentane/Et_2O: 30/70; White solid; Mp: 68 °C; NMR (400 MHz, $CDCl_3$): *δ* 7.41-7.36 (m, 2H), 7.32-7.26 (br. m, 3H), 4.03-3.87 (m, 3H), 3.66 (app. dd, *J* = 23.6 and 12.5 Hz, 1H), 3.60-3.49 (m, 1H), 3.46-3.35 (m, 1H), 3.29 (s, 3H), 3.00 (br. t, *J* = 5.8 Hz, 0.50H, rotamer), 2.78 (br. t, *J* = 5.8 Hz, 0.50H, rotamer), 2.54 (dt, *J* = 14.7 and 7.6 Hz, 1H), 2.29 (ddd, *J* = 13.5, 7.8 and 5.7 Hz, 1H), 1.43 (s, 9H); ^{13}C NMR (100 MHz, $CDCl_3$): *δ* 154.5, 131.6 and 131.4 (rotamers), 128.4, 128.3 and 128.1 (rotamers), 122.5 and 122.5 (rotamers), 81.3 and 81.2 (rotamers), 80.3 and 80.2 (rotamers), 74.1 and 73.5 (rotamers), 72.7 and 72.5 (rotamers), 64.5, 53.0 and 52.7 (rotamers), 44.2 and 43.6 (rotamers), 40.4 and 40.3 (rotamers), 33.9 and 32.9 (rotamers), 28.5; ESIHRMS *m/z* calcd for $C_{19}H_{26}N_2O_5SNa$ $[M+Na]^+$ 417.1455, found 417.1477.

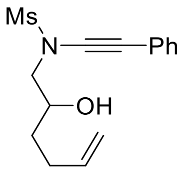

S2aj

***N*-(2-Hydroxyhex-5-en-1-yl)-*N*-(methanesulfonyl)phenylethynylamine S2aj**. Prepared according to general procedure I (4.3 mmol of OTBS-protected-ynamide **S1aj**, 1.5 equiv. of TBAF, 0.20 M in THF, 0 °C, 1 hour). Yield: 90% (1.13 g, 3.86 mmol). Solvent system for flash column chromatography: petroleum ether/EtOAc: 80/20; Colorless oil; ^{1}H NMR (400 MHz, $CDCl_3$): *δ* 7.44-7.38 (m, 2H), 7.33-7.28 (m, 3H), 5.84 (ddt, *J* = 16.9, 10.2 and 6.7 Hz, 1H), 5.09 (dq, *J* = 17.1 and 1.7 Hz, 1H), 5.02 (dq, *J* = 10.2 and 1.4 Hz, 1H), 4.12-4.04 (m, 1H), 3.67 (A of ABX syst., *J* = 14.1 and 8.6 Hz, 1H), 3.52 (B of ABX syst., *J* = 14.1 and 3.1 Hz, 1H), 3.23 (s, 3H), 2.34-2.16 (m, 2H), 2.08 (br. s, 1H), 1.72-1.57 (m, 2H); ^{13}C NMR (100 MHz, $CDCl_3$): *δ* 137.8, 131.7,

128.5, 128.3, 122.4, 115.7, 81.8, 71.2, 69.4, 57.5, 38.6, 33.4, 29.9; ESIHRMS *m/z* calcd for $C_{15}H_{20}NO_3S$ $[M+H]^+$ 294.1158, found 294.1159.

2.5. Experimental Procedures and Characterization Data: Synthesis of Methanesulfonyloxylethyl-Ynamides

General procedure

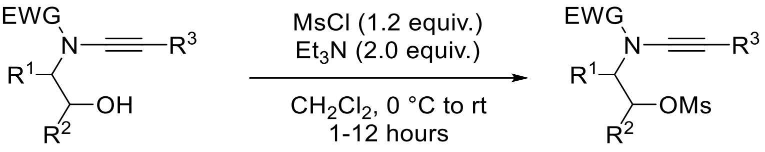

To a solution of the hydroxyethyl-ynamide **S2** (1.0 equiv.) and triethylamine (2.0 equiv.) in anhydrous dichloromethane (0.2 M) was added methanesulfonyl chloride (MsCl, 1.2 equiv.) dropwise at 0 °C under argon. The resulting mixture was stirred at rt for the appropriate amount of time (1-12 hours), quenched with a saturated aqueous solution of $NaHCO_3$ and extracted with dichloromethane. The combined organic layers were then washed with water, brine, dried over $MgSO_4$, filtered and concentrated under reduced pressure. The crude residue was finally purified by flash column chromatography over silica gel to afford the desired methanesulfonyloxyethyl-ynamide **S3**.

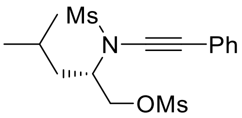

S3v

(*S*)-*N*-(Methanesulfonyl)-*N*-(1-methanesulfonyloxy-4-methylpentan-2-yl)phenylethynyl amine S3v. Prepared according to general procedure (508 µmol of hydroxyethyl-ynamide **S2v**, 1 hour). Yield: 89% (170 mg, 455 µmol). Solvent system for flash column chromatography: petroleum ether/EtOAc: 80/20; Colorless solid; Mp: 60 °C; $[\alpha]_D^{20}$ + 22 (*c* 1.1, $CHCl_3$); ^1H NMR (400 MHz, $CDCl_3$): δ 7.46-7.41 (m, 2H), 7.35-7.30 (m, 3H), 4.43 (app. tt, J = 9.7 and 3.7 Hz, 1H), 4.35 (app. t, J = 10.3 Hz, 1H), 4.20 (dd, J = 10.4 and 3.5 Hz, 1H), 3.25 (s, 3H), 3.09 (s, 3H), 1.86-1.74 (m, 1H), 1.73 (ddd, J = 14.1, 10.0 and 4.4 Hz, 1H), 1.30-1.22 (m, 1H), 1.01 (d, J = 6.6 Hz, 3H), 1.00 (d, J = 6.5 Hz, 3H); ^{13}C NMR (100 MHz, $CDCl_3$): δ 132.0, 128.6, 128.6, 122.2, 77.3, 74.4, 69.1, 57.4, 39.6, 38.0, 37.9, 24.5, 23.4, 21.6; ESIHRMS *m/z* calcd for $C_{16}H_{24}NO_5S_2$ $[M+H]^+$ 374.1090, found 374.1093.

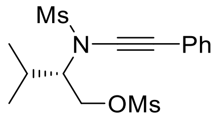

S3w

(*S*)-*N*-(Methanesulfonyl)-*N*-(1-methanesulfonyloxy-3-methylbutan-2-yl)phenylethynyl amine S3w. Prepared according to general procedure (4.97 mmol of hydroxyethyl-ynamide **S2w**, 12 hours). Yield: 91% (1.63 g, 4.53 mmol). Solvent system for flash column chromatography: petroleum ether/EtOAc: 75/25; Yellow oil; $[\alpha]_D^{20}$ + 51 (*c* 1.1, $CHCl_3$); ^1H NMR (400 MHz, $CDCl_3$): *δ* 7.46-7.40 (m, 2H), 7.34-7.29 (m, 3H), 4.46 (A of ABX syst., *J* = 10.7 and 9.5 Hz, 1H), 4.41 (B of ABX syst., *J* = 10.7 and 3.8 Hz, 1H), 4.00 (td, *J* = 9.2 and 3.8 Hz, 1H), 3.23 (s, 3H), 3.08 (s, 3H), 2.00 (dsept., *J* = 9.0 and 6.8 Hz, 1H), 1.12 (d, *J* = 6.7 Hz, 3H), 1.07 (d, *J* = 6.8 Hz, 3H); ^{13}C NMR (100 MHz, $CDCl_3$): *δ* 131.8, 128.5 (2C), 122.2, 78.1, 74.1, 67.9, 64.7, 39.2, 37.7, 29.0, 19.8, 19.5; ESIHRMS *m/z* calcd for $C_{15}H_{22}NO_5S_2$ $[M+H]^+$ 360.0934, found 360.0937.

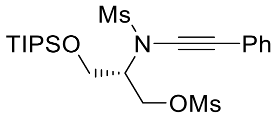

S3x

(*S*)-*N*-(Methanesulfonyl)-*N*-{1-methanesulfonyloxy-3-[(triisopropylsilyl)oxy]propan-2-yl}phenylethynylamine S3x. Prepared according to general procedure (327 µmol of hydroxyethyl-ynamide **S2x**, 1.5 hours). Yield: 84% (138 mg, 274 µmol). Solvent system for flash column chromatography: petroleum ether/EtOAc: 80/20; Yellow oil; $[\alpha]_D^{20}$ -13 (*c* 2.0, $CHCl_3$); ^1H NMR (400 MHz, $CDCl_3$): *δ* 7.45-7.40 (m, 2H), 7.34-7.29 (m, 3H), 4.52 (A of ABX syst., *J* = 10.8 and 8.9 Hz, 1H), 4.46 (B of ABX syst., *J* = 10.7 and 3.9 Hz, 1H), 4.44-4.38 (m, 1H), 4.03 (A' of A'B'X syst., *J* = 10.5 and 6.9 Hz, 1H), 3.87 (B' of A'B'X syst., *J* = 10.5 and 6.1 Hz, 1H), 3.23 (s, 3H), 3.09 (s, 3H), 1.12-1.02 (m, 21H); ^{13}C NMR (100 MHz, $CDCl_3$): *δ* 131.9, 128.6, 128.5, 122.2, 77.8, 73.9, 67.0, 61.9, 60.5, 39.3, 37.8, 18.0, 11.9; ESIHRMS *m/z* calcd for $C_{22}H_{38}NO_6S_2Si$ $[M+H]^+$ 504.1904, found 504.1902.

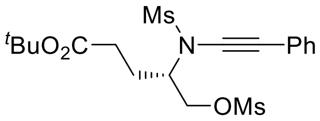

S3y

(*S*)-*N*-(Methanesulfonyl)-*N*-(4-methanesulfonyloxy-1-*tert*-butoxycarbonylbutan-3-yl)phenylethynylamine S3y. Prepared according to general procedure (200 µmol of hydroxyethyl-ynamide **S2y**, 1 hour). Yield: quant. (89 mg, 200 µmol). Solvent system for flash column chromatography: petroleum ether/EtOAc: 70/30; Pale yellow oil; $[\alpha]_D^{25}$ - 11 (*c* 0.7, $CHCl_3$); ^1H NMR (400 MHz, $CDCl_3$): *δ* 7.47-7.42 (m, 2H), 7.35-7.29 (m, 3H), 4.46-4.36 (m, 2H), 4.30-4.20 (m, 1H), 3.25 (s, 3H), 3.08 (s, 3H), 2.48 (app. dt, *J* = 17.1 and 6.6 Hz, 1H), 2.42 (app. dt, *J* = 17.1 and 7.5 Hz, 1H), 1.89 (app. q, *J* = 6.9 Hz, 2H), 1.46 (s, 9H); ^{13}C NMR (100 MHz, $CDCl_3$): *δ* 171.7, 132.0, 128.7, 128.6, 122.0, 81.3, 76.9, 74.4, 68.8, 58.4, 39.6, 37.8, 31.0, 28.2, 24.3; ESIHRMS *m/z* calcd for $C_{19}H_{27}NO_7S_2Na$ $[M+Na]^+$ 468.1121, found 468.1122.

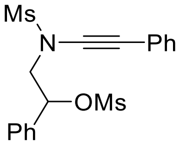

S3ae

***N*-(Methanesulfonyl)-*N*-(2-methanesulfonyloxy-2-phenylethyl)phenylethynylamine S3ae**. Prepared according to general procedure (560 µmol of crude hydroxyethyl-ynamide **S2ae**, 12 hours). Yield: 75% over 2 steps from **S1ae** (165 mg, 419 µmol). Solvent system for flash column chromatography: petroleum ether/EtOAc: 80/20 then 70/30; Pale yellow oil; ^1H NMR (400 MHz, $CDCl_3$): *δ* 7.51-7.43 (m, 7H), 7.36-7.31 (m, 3H), 5.90 (X of ABX syst., *J* = 9.7 and 3.7 Hz, 1H), 4.20 (A of ABX syst., *J* = 14.8 and 9.7 Hz, 1H), 3.77 (B of ABX syst., *J* = 14.8 and 3.6 Hz, 1H), 3.18 (s, 3H), 2.78 (s, 3H); ^{13}C NMR (100 MHz, $CDCl_3$): *δ* 134.6, 132.2, 130.2, 129.4, 128.7, 128.5, 127.2, 122.1, 80.9, 80.3, 72.3, 55.3, 39.4, 39.3; ESIHRMS *m/z* calcd for $C_{17}H_{18}NO_3S$ $[M-SO_2CH_3+2H]^+$ 316.1002, found 316.1007.

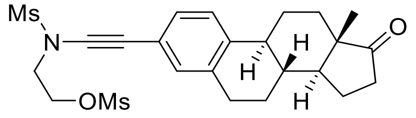

S3ah

(8*R*,9*S*,13*S*,14*S*)-*N*-(2-Methanesulfonyloxyethyl)-*N*-(methanesulfonyl)-(13-methyl-17-oxo-7,8,9,11,12,13,14,15,16,17-decahydro-6*H*-cyclopenta[a]phenanthren-3-yl)ethynylamine S3ah. Prepared according to general procedure (500 µmol of hydroxyethyl-ynamide **S2ah**, 1 hour). Yield: 96% (236 mg, 478 µmol). Purification by precipitation/trituration in CH_2Cl_2/pentane, then in Et_2O; White solid; Mp: 138 °C; $[\alpha]_D^{20}$ + 88 (*c* 1.1, $CHCl_3$); ^1H NMR (400 MHz, $CDCl_3$): δ 7.26-7.17 (m, 3H), 4.51 (t, J = 4.9 Hz, 2H), 3.91 (t, J = 5.0 Hz, 2H), 3.21 (s, 3H), 3.10 (s, 3H), 2.88 (dd, J = 8.9 and 4.4 Hz, 2H), 2.51 (dd, J = 19.0 and 8.9 Hz, 1H), 2.44-2.37 (m, 1H), 2.34-2.24 (m, 1H), 2.20-2.10 (m, 1H), 2.10-1.94 (m, 3H), 1.69-1.38 (m, 6H), 0.91 (s, 3H); ^{13}C NMR (100 MHz, $CDCl_3$): δ 220.8, 140.8, 136.9, 132.5, 129.3, 125.6, 119.2, 79.6, 72.2, 66.0, 50.6, 50.3, 48.1, 44.6, 38.9, 38.1, 38.0, 36.0, 31.7, 29.2, 26.4, 25.7, 21.7, 14.0; ESIHRMS *m/z* calcd for $C_{24}H_{32}NO_6S_2$ $[M+H]^+$ 494.1666, found 494.1670.

2.6. Experimental Procedures and Characterization Data: Synthesis of Iodoethyl-Ynamides

General procedures I, II and III: iodination from hydroxyethyl-ynamides

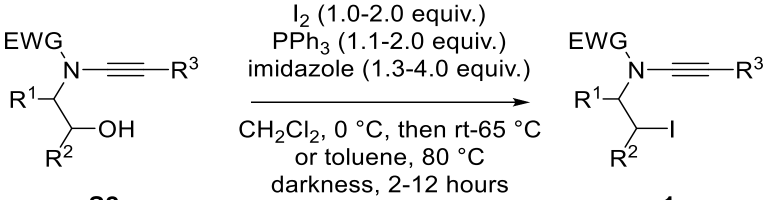

S2 **1**

GP I: To a solution of imidazole (1.3-3.0 equiv.) and triphenylphosphine (1.1-2.0 equiv.) in dichloromethane (half of the total amount) was added iodine (1.0-1.5 equiv.) at 0 °C under argon. The mixture was stirred at 0 °C in the dark for 10 minutes. A solution of the hydroxyethyl-ynamide **S2** (1 equiv.) in dichloromethane (half of the total amount) was then added dropwise via *cannula*. The resulting mixture (0.07-0.20 M) was stirred in the dark at the appropriate temperature (rt-65 °C) for the appropriate amount of time (2-12 hours). The reaction mixture was then diluted with Et_2O, filtered over a short pad of Celite® (washed with Et_2O) and concentrated under reduced pressure. The crude residue was finally purified by flash column chromatography over silica gel to afford the desired iodoethyl-ynamide **1**.

GP II: To a solution of hydroxyethyl-ynamide **S2** (1.0 equiv.) in dichloromethane (0.04 M) were added imidazole (2.8-4.0 equiv.), triphenylphosphine (1.4-2.0 equiv.) and iodine (1.4-2.0 equiv.) at 0 °C under argon. The resulting mixture was stirred in the dark at rt for 4 hours, diluted with Et_2O, filtered over a short pad of Celite® (washed with Et_2O) and concentrated under reduced pressure. The crude residue was finally purified by flash column chromatography over silica gel to afford the desired iodoethyl-ynamide **1**.

GP III: To a solution of iodine (1.42 equiv.), triphenylphosphine (1.42 equiv.) and imidazole (1.42 equiv.) in toluene (half of the total amount) was added a solution of hydroxyethyl-ynamide **S2** (1 equiv.) in toluene (half of the total amount) at rt under argon. The resulting mixture (0.1 M) was stirred in the dark at 80 °C for 4 hours, diluted with Et_2O, filtered over a short pad of Celite® (washed with Et_2O) and concentrated under reduced pressure. The crude residue was finally purified by flash column chromatography over silica gel to afford the desired iodoethyl-ynamide **1**.

General procedure IV: iodination from methanesulfonyloxyethyl-ynamides

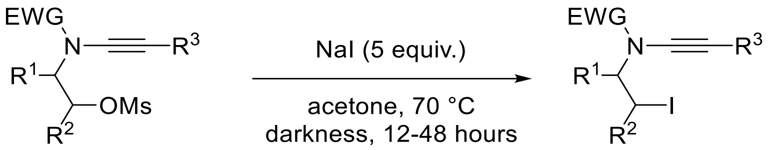

S3 **1**

To a solution of methanesulfonyloxyethyl-ynamide **S3** (1.0 equiv.) in distilled acetone (0.1-0.3 M) was added sodium iodide (5.0 equiv.) under argon. The resulting mixture was stirred in the dark at 70 °C for the appropriate amount of time (12-48 hours). The reaction mixture was then filtered and concentrated under reduced pressure. The residue was diluted in dichloromethane and a saturated aqueous solution of $NaHCO_3$ was added. The layers were separated and the aqueous layer was further extracted with dichloromethane. The combined organic layers were washed with brine, dried over $MgSO_4$, filtered and concentrated under reduced pressure. The crude residue was finally purified by flash column chromatography over silica gel to afford the desired iodoethyl-ynamide **1**.

⚠ ***Caution****: Iodoethyl-ynamides may be sensitive to light and must therefore be handled and stored in the dark.*

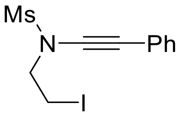

1a

***N*-(2-Iodoethyl)-*N*-(methanesulfonyl)phenylethynylamine 1a**. Prepared according to general procedure I (1.38 mmol of hydroxyethyl-ynamide **S2a**, 1.1 equiv. of I_2, 1.5 equiv. of PPh_3, 1.5 equiv. of imidazole, 0.07 M in dichloromethane, rt, 3 hours). Yield: 74% (358 mg, 1.03 mmol). Solvent system for flash column chromatography: petroleum ether/EtOAc: 90/10 then 80/20; White solid; Mp: 62 °C; ^1H NMR (400 MHz, $CDCl_3$): δ 7.46-7.40 (m, 2H), 7.35-7.29 (m, 3H), 3.92 (t, J = 7.5 Hz, 2H), 3.45 (t, J = 7.5 Hz, 2H), 3.22 (s, 3H); ^{13}C NMR (100 MHz, $CDCl_3$): δ 131.9, 128.6, 128.5, 122.2, 80.3, 72.1, 53.5, 39.3, -0.2; ESIHRMS *m/z* calcd for $C_{11}H_{13}INO_2S$ $[M+H]^+$ 349.9706, found 349.9718.

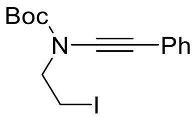

1b

***N-tert*-Butoxycarbonyl-*N*-(2-iodoethyl)phenylethynylamine 1b**. Prepared according to general procedure I (1.57 mmol of hydroxyethyl-ynamide **S2b**, 1.1 equiv. of I_2, 1.5 equiv. of PPh_3, 1.5 equiv. of imidazole, 0.08 M in dichloromethane, rt, 5 hours). Yield: 87% (508 mg, 1.37 mmol). Solvent system for flash column chromatography: petroleum ether/EtOAc: gradient from 100/0 to 90/10; Pale yellow oil; ^{1}H NMR (300 MHz, $CDCl_3$): δ 7.41-7.32 (m, 2H), 7.31-7.23 (m, 3H), 3.86 (t, J = 7.5 Hz, 2H), 3.36 (t, J = 7.7 Hz, 2H), 1.53 (s, 9H); ^{13}C NMR (100 MHz, $CDCl_3$): δ 153.5, 130.9, 128.4, 127.5, 123.4, 83.2, 82.8, 71.1, 51.4, 28.1, 0.0; ESIHRMS *m/z* calcd for $C_{15}H_{19}INO_2$ $[M+H]^+$ 372.0455, found 372.0465.

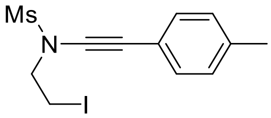

1c

***N*-(2-Iodoethyl)-*N*-(methanesulfonyl)(*p*-tolyl)ethynylamine 1c**. Prepared according to general procedure I (592 µmol of hydroxyethyl-ynamide **S2c**, 1.1 equiv. of I_2, 1.1 equiv. of PPh_3, 3.0 equiv. of imidazole, 0.08 M in dichloromethane, rt, 2 hours). Yield: 53% (114 mg, 314 µmol). Solvent system for flash column chromatography: petroleum ether/EtOAc: 90/10; Yellow oil; ^{1}H NMR (400 MHz, $CDCl_3$): δ 7.33 (d, J = 8.0 Hz, 2H), 7.13 (d, J = 8.0 Hz, 2H), 3.91 (t, J = 7.6 Hz, 2H), 3.44 (t, J = 7.6 Hz, 2H), 3.21 (s, 3H), 2.35 (s, 3H); ^{13}C NMR (100 MHz, $CDCl_3$): δ 138.9, 132.0, 129.3, 119.0, 79.6, 72.2, 53.6, 39.2, 21.6, -0.1; ESIHRMS *m/z* calcd for $C_{12}H_{15}INO_2S$ $[M+H]^+$ 363.9863, found 363.9862.

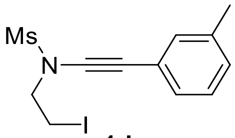

1d

***N*-(2-Iodoethyl)-*N*-(methanesulfonyl)(*m*-tolyl)ethynylamine 1d**. Prepared according to general procedure II (3.5 mmol of hydroxyethyl-ynamide **S2d**, 2.0 equiv. of I_2, 2.0 equiv. of

PPh_3, 4.0 equiv. of imidazole, 0.04 M in dichloromethane, rt, 4 hours). Yield: 64% (813 mg, 2.24 mmol). Solvent system for flash column chromatography: petroleum ether/EtOAc: 80/20; Yellow solid; Mp: 65 °C; ^1H NMR (400 MHz, $CDCl_3$): δ 7.27-7.18 (m, 3H), 7.15-7.12 (m, 1H), 3.92 (t, J = 7.5 Hz, 2H), 3.44 (t, J = 7.5 Hz, 2H), 3.22 (s, 3H), 2.33 (s, 3H); ^{13}C NMR (100 MHz, $CDCl_3$): δ 138.3, 132.4, 129.4, 128.9, 128.4, 121.9, 80.0, 72.3, 53.5, 39.3, 21.3, -0.2; ESIHRMS *m/z* calcd for $C_{12}H_{15}INO_2S$ $[M+H]^+$ 363.9863, found 363.9862.

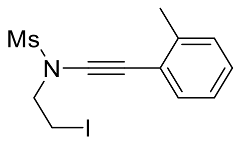

1e

***N*-(2-Iodoethyl)-*N*-(methanesulfonyl)(*o*-tolyl)ethynylamine 1e**. Prepared according to general procedure I (2.0 mmol of hydroxyethyl-ynamide **S2e**, 1.1 equiv. of I_2, 1.5 equiv. of PPh_3, 1.5 equiv. of imidazole, 0.08 M in dichloromethane, rt, 2.5 hours). Yield: 83% (603 mg, 1.66 mmol). Solvent system for flash column chromatography: petroleum ether/Et_2O: 60/40; White solid; Mp: 62 °C; ^1H NMR (400 MHz, $CDCl_3$): δ 7.38 (d, J = 7.4 Hz, 1H), 7.25-7.19 (m, 2H), 7.17-7.10 (m, 1H), 3.94 (t, J = 7.4 Hz, 2H), 3.46 (t, J = 7.4 Hz, 2H), 3.23 (s, 3H), 2.43 (s, 3H); ^{13}C NMR (100 MHz, $CDCl_3$): δ 140.2, 131.8, 129.7, 128.5, 125.8, 122.0, 84.1, 71.2, 53.5, 39.2, 20.9, -0.1; ESIHRMS *m/z* calcd for $C_{12}H_{15}INO_2S$ $[M+H]^+$ 363.9863, found 363.9862.

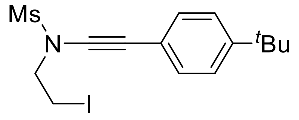

1f

***N*-(2-Iodoethyl)-*N*-(methanesulfonyl)(*p*-*tert*-butylphenyl)ethynylamine 1f**. Prepared according to general procedure I (1.18 mmol of hydroxyethyl-ynamide **S2f**, 1.1 equiv. of I_2, 1.5 equiv. of PPh_3, 1.5 equiv. of imidazole, 0.20 M in dichloromethane, rt, 2 hours). Yield: 73% (351 mg, 866 µmol). Solvent system for flash column chromatography: petroleum ether/EtOAc: 90/10; White solid; Mp: 64 °C; ^1H NMR (400 MHz, $CDCl_3$): δ 7.38 (d, J = 8.5 Hz, 2H), 7.34 (d, J = 8.7 Hz, 2H), 3.91 (t, J = 7.5 Hz, 2H), 3.44 (t, J = 7.5 Hz, 2H), 3.21 (s, 3H), 1.31 (s,

9H); ^{13}C NMR (100 MHz, $CDCl_3$): δ 152.1, 131.8, 125.5, 119.0, 79.7, 72.1, 53.6, 39.2, 35.0, 31.3, -0.1; ESIHRMS *m/z* calcd for $C_{15}H_{21}INO_2S$ $[M+H]^+$ 406.0332, found 406.0330.

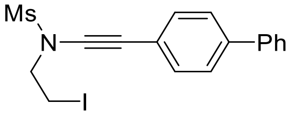

1g

***N*-(2-Iodoethyl)-*N*-(methanesulfonyl)(*p*-biphenyl)ethynylamine 1g**. Prepared according to general procedure I (2.54 mmol of hydroxyethyl-ynamide **S2g**, 1.1 equiv. of I_2, 1.5 equiv. of PPh_3, 1.5 equiv. of imidazole, 0.20 M in dichloromethane, 40 °C, 3 hours). Yield: 93% (1.0 g, 2.35 mmol). Solvent system for flash column chromatography: petroleum ether/EtOAc: 80/20; White solid; Mp: 131 °C; ^1H NMR (400 MHz, $CDCl_3$): δ 7.61-7.57 (m, 2H), 7.56 (d, *J* = 8.2 Hz, 2H), 7.51 (d, *J* = 8.2 Hz, 2H), 7.45 (app. t, *J* = 7.5 Hz, 2H), 7.36 (app. t, *J* = 7.3 Hz, 1H), 3.94 (t, *J* = 7.5 Hz, 2H), 3.47 (t, *J* = 7.5 Hz, 2H), 3.24 (s, 3H); ^{13}C NMR (100 MHz, $CDCl_3$): δ 141.3, 140.4, 132.3, 129.0, 127.9, 127.2, 127.1, 121.0, 80.9, 72.1, 53.6, 39.4, -0.2; ESIHRMS *m/z* calcd for $C_{17}H_{16}INO_2SNa$ $[M+Na]^+$ 447.9839, found 447.9837.

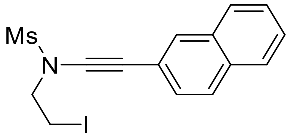

1h

***N*-(2-Iodoethyl)-*N*-(methanesulfonyl)(naphthalen-2-yl)ethynylamine 1h**. Prepared according to general procedure II (518 µmol of hydroxyethyl-ynamide **S2h**, 2.0 equiv. of I_2, 2.0 equiv. of PPh_3, 4.0 equiv. of imidazole, 0.04 M in dichloromethane, rt, 4 hours). Yield: 71% (147 mg, 368 µmol). Solvent system for flash column chromatography: petroleum ether/EtOAc: 80/20; White solid; Mp: 99 °C; ^1H NMR (400 MHz, $CDCl_3$): δ 7.96 (s, 1H), 7.84-7.75 (m, 3H), 7.52-7.44 (m, 3H), 3.96 (t, *J* = 7.5 Hz, 2H), 3.49 (t, *J* = 7.5 Hz, 2H), 3.26 (s, 3H); ^{13}C NMR (100 MHz, $CDCl_3$): δ 133.1, 132.9, 131.6, 128.5, 128.2, 127.9, 127.8, 126.9, 126.8, 119.5, 80.6, 72.6, 53.6, 39.4, -0.2; ESIHRMS *m/z* calcd for $C_{15}H_{14}INO_2SNa$ $[M+Na]^+$ 421.9682, found 421.9681.

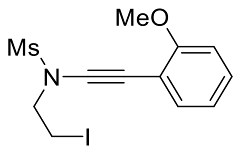

1i

***N*-(2-Iodoethyl)-*N*-(methanesulfonyl)(*o*-methoxyphenyl)ethynylamine 1i**. Prepared according to general procedure I (1.00 mmol of hydroxyethyl-ynamide **S2i**, 1.5 equiv. of I_2, 2.0 equiv. of PPh_3, 2.0 equiv. of imidazole, 0.20 M in dichloromethane, rt, overnight). Yield: 50% (188 mg, 496 µmol). Solvent system for flash column chromatography: petroleum ether/EtOAc: 90/10; White solid; Mp: 89 °C; ^{1}H NMR (400 MHz, $CDCl_3$): δ 7.38 (dd, J = 7.6 and 1.7 Hz, 1H), 7.29 (ddd, J = 8.4, 7.5 and 1.7 Hz, 1H), 6.90 (td, J = 7.5 and 1.0 Hz, 1H), 6.87 (d, J = 8.4 and 1.0 Hz, 1H), 3.93 (t, J = 7.5 Hz, 2H), 3.87 (s, 3H), 3.49 (t, J = 7.5 Hz, 2H), 3.25 (s, 3H); ^{13}C NMR (100 MHz, $CDCl_3$): δ 160.2, 133.1, 129.9, 120.6, 111.5, 110.8, 84.2, 68.8, 55.9, 53.4, 39.1, -0.2; ESIHRMS *m/z* calcd for $C_{12}H_{15}INO_3S$ [M+H]$^+$ 379.9812, found 379.9816.

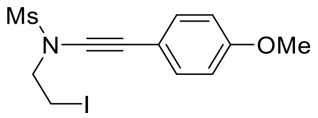

1j

***N*-(2-Iodoethyl)-*N*-(methanesulfonyl)(*p*-methoxyphenyl)ethynylamine 1j**. Prepared according to general procedure I (1.86 mmol of hydroxyethyl-ynamide **S2j**, 1.0 equiv. of I_2, 1.5 equiv. of PPh_3, 1.5 equiv. of imidazole, 0.10 M in dichloromethane, rt, 5 hours). Yield: 82% (577 mg, 1.52 mmol). Solvent system for flash column chromatography: petroleum ether/EtOAc: 90/10; White solid; Mp: 78 °C; ^{1}H NMR (400 MHz, $CDCl_3$): δ 7.39 (d, J = 8.8 Hz, 2H), 6.84 (d, J = 8.8 Hz, 2H), 3.91 (t, J = 7.5 Hz, 2H), 3.82 (s, 3H), 3.44 (t, J = 7.5 Hz, 2H), 3.21 (s, 3H); ^{13}C NMR (100 MHz, $CDCl_3$): δ 160.1, 134.0, 114.2, 114.0, 79.0, 71.8, 55.5, 53.6, 39.2, 0.0; ESIHRMS *m/z* calcd for $C_{12}H_{14}INO_3SNa$ [M+Na]$^+$ 401.9631, found 401.9633.

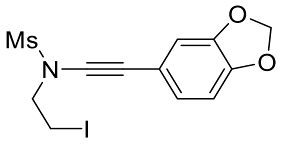

1k

***N*-(2-Iodoethyl)-*N*-(methanesulfonyl)(benzo[*d*][1,3]dioxol-5-yl)ethynylamine 1k**. Prepared according to general procedure II (1.41 mmol of hydroxyethyl-ynamide **S2k**, 2.0 equiv. of I_2, 2.0 equiv. of PPh_3, 4.0 equiv. of imidazole, 0.04 M in dichloromethane, rt, 4 hours). Yield: 76% (422 mg, 1.07 mmol). Solvent system for flash column chromatography: petroleum ether/EtOAc: 80/20; Off-white solid; Mp: 69 °C; ^{1}H NMR (400 MHz, $CDCl_3$): δ 6.98 (dd, J = 8.0 and 1.6 Hz, 1H), 6.89 (d, J = 1.6 Hz, 1H), 6.76 (d, J = 8.0 Hz, 1H), 5.98 (s, 2H), 3.90 (t, J = 7.5 Hz, 2H), 3.43 (t, J = 7.5 Hz, 2H), 3.20 (s, 3H); ^{13}C NMR (100 MHz, $CDCl_3$): δ 148.4, 147.6, 127.1, 115.2, 112.3, 108.6, 101.6, 78.7, 71.9, 53.5, 39.2, -0.1; ESIHRMS *m/z* calcd for $C_{12}H_{12}INO_4SNa$ $[M+Na]^+$ 415.9424, found 415.9424.

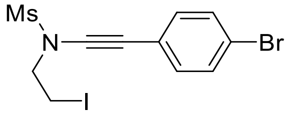

1l

***N*-(2-Iodoethyl)-*N*-(methanesulfonyl)(*p*-bromophenyl)ethynylamine 1l**. Prepared according to general procedure II (628 µmol of hydroxyethyl-ynamide **S2l**, 2.0 equiv. of I_2, 2.0 equiv. of PPh_3, 4.0 equiv. of imidazole, 0.04 M in dichloromethane, rt, 4 hours). Yield: 58% (157 mg, 367 µmol). Solvent system for flash column chromatography: petroleum ether/EtOAc: 80/20; Yellow solid; Mp: 71 °C; ^{1}H NMR (400 MHz, $CDCl_3$): δ 7.45 (d, J = 8.0 Hz, 2H), 7.28 (d, J = 8.2 Hz, 2H), 3.91 (t, J = 7.6 Hz, 2H), 3.43 (t, J = 7.6 Hz, 2H), 3.21 (s, 3H); ^{13}C NMR (100 MHz, $CDCl_3$): δ 133.2, 131.8, 122.7, 121.2, 81.4, 71.3, 53.5, 39.5, -0.3; ESIHRMS *m/z* calcd for $C_{11}H_{12}{}^{79}BrINO_2S$ $[M+H]^+$ 427.8811, found 427.8812, *m/z* calcd for $C_{11}H_{12}{}^{81}BrINO_2S$ $[M+H]^+$ 429.8791, found 429.8791.

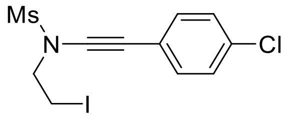

1m

***N*-(2-Iodoethyl)-*N*-(methanesulfonyl)(*p*-chlorophenyl)ethynylamine 1m**. Prepared according to general procedure II (475 µmol of hydroxyethyl-ynamide **S2m**, 2.0 equiv. of I_2, 2.0 equiv. of PPh_3, 4.0 equiv. of imidazole, 0.04 M in dichloromethane, rt, 4 hours). Yield: 69% (126 mg, 328 µmol). Solvent system for flash column chromatography: petroleum ether/EtOAc: 80/20; Yellow solid; Mp: 77 °C; ^{1}H NMR (400 MHz, $CDCl_3$): δ 7.35 (d, J = 8.5 Hz, 2H), 7.29 (d, J = 8.6 Hz, 2H), 3.92 (t, J = 7.5 Hz, 2H), 3.43 (t, J = 7.5 Hz, 2H), 3.22 (s, 3H); ^{13}C NMR (100 MHz, $CDCl_3$): δ 134.6, 133.1, 128.9, 120.7, 81.2, 71.2, 53.6, 39.5, -0.3; ESIHRMS *m/z* calcd for $C_{11}H_{12}{}^{35}ClINO_2S$ [M+H]$^+$ 383.9316, found 383.9316.

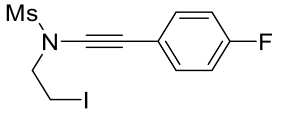

1n

***N*-(2-Iodoethyl)-*N*-(methanesulfonyl)(*p*-fluorophenyl)ethynylamine 1n**. Prepared according to general procedure I (194 µmol of hydroxyethyl-ynamide **S2n**, 1.0 equiv. of I_2, 1.3 equiv. of PPh_3, 1.3 equiv. of imidazole, 0.08 M in dichloromethane, rt, 2 hours). Yield: 53% (38 mg, 103 µmol). Solvent system for flash column chromatography: petroleum ether/EtOAc: 90/10; Yellow solid; Mp: 68 °C; ^{1}H NMR (600 MHz, $CDCl_3$): δ 7.42 (dd, J = 8.0 and 5.5 Hz, 2H), 7.01 (app. t, J = 8.6 Hz, 2H), 3.91 (t, J = 7.5 Hz, 2H), 3.43 (t, J = 7.5 Hz, 2H), 3.21 (s, 3H); ^{13}C NMR (100 MHz, $CDCl_3$): δ 162.8 (d, J = 250.2 Hz), 134.1 (d, J = 8.4 Hz), 118.2 (d, J = 3.4 Hz), 115.8 (d, J = 22.0 Hz), 80.0, 71.1, 53.5, 39.4, -0.2; ^{19}F NMR (376 MHz, $CDCl_3$): δ -110.9; ESIHRMS *m/z* calcd for $C_{11}H_{12}FINO_2S$ [M+H]$^+$ 367.9612, found 367.9611.

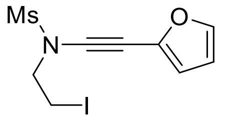

1o

***N*-(2-Iodoethyl)-*N*-(methanesulfonyl)(furan-2-yl)ethynylamine 1o**. Prepared according to general procedure I (406 µmol of hydroxyethyl-ynamide **S2o**, 1.1 equiv. of I_2, 1.5 equiv. of PPh_3, 1.5 equiv. of imidazole, 0.20 M in dichloromethane, rt, 3 hours). Yield: 73% (100 mg, 295 µmol). Solvent system for flash column chromatography: petroleum ether/EtOAc: 90/10; Off-white oil; ^{1}H NMR (400 MHz, $CDCl_3$): δ 7.43 (d, J = 1.8 Hz, 1H), 6.70 (d, J = 3.4 Hz, 1H), 6.42 (dd, J = 3.4 and 1.9 Hz, 1H), 3.92 (t, J = 7.5 Hz, 2H), 3.42 (t, J = 7.5 Hz, 2H), 3.22 (s, 3H); ^{13}C NMR (100 MHz, $CDCl_3$): δ 144.7, 136.3, 118.3, 111.4, 84.7, 63.2, 53.7, 39.6, -0.4.

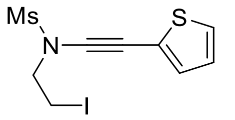

1p

***N*-(2-Iodoethyl)-*N*-(methanesulfonyl)(thiophen-2-yl)ethynylamine 1p**. Prepared according to general procedure I (815 µmol of hydroxyethyl-ynamide **S2p**, 1.5 equiv. of I_2, 2.0 equiv. of PPh_3, 2.0 equiv. of imidazole, 0.08 M in dichloromethane, rt, 3 hours). Yield: 89% (257 mg, 723 µmol). Solvent system for flash column chromatography: petroleum ether/EtOAc: 80/20; Colorless oil; ^{1}H NMR (400 MHz, $CDCl_3$): δ 7.32 (dd, J = 5.2 and 1.1 Hz, 1H), 7.26 (obs. dd, J = 3.6 and 1.2 Hz, 1H), 7.00 (dd, J = 5.2 and 3.6 Hz, 1H), 3.92 (t, J = 7.5 Hz, 2H), 3.43 (t, J = 7.5 Hz, 2H), 3.22 (s, 3H); ^{13}C NMR (100 MHz, $CDCl_3$): δ 134.1, 128.7, 127.3, 122.1, 83.9, 65.6, 53.7, 39.5, -0.3; ESIHRMS *m/z* calcd for $C_9H_{14}IN_2O_2S_2$ $[M+NH_4]^+$ 372.9536, found 372.9536.

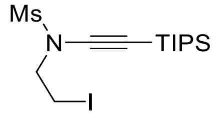

1q

***N*-(2-Iodoethyl)-*N*-(methanesulfonyl)(triisopropylsilyl)ethynylamine 1q**. Prepared according to general procedure I (738 µmol of hydroxyethyl-ynamide **S2q**, 1.1 equiv. of I_2, 1.5 equiv. of PPh_3, 1.5 equiv. of imidazole, 0.14 M in dichloromethane, rt, 2 hours). Yield: 83% (264 mg, 615

µmol). Solvent system for flash column chromatography: petroleum ether/EtOAc: gradient from 90/10 to 80/20; Colorless oil; ^{1}H NMR (400 MHz, $CDCl_3$): δ 3.83 (t, J = 7.3 Hz, 2H), 3.39 (t, J = 7.3 Hz, 2H), 3.18 (s, 3H), 1.08 (app. s, 21H); ^{13}C NMR (100 MHz, $CDCl_3$): δ 94.5, 71.8, 53.1, 39.0, 18.8, 11.4, -0.4; ESIHRMS m/z calcd for $C_{14}H_{29}INO_2SSi$ [M+H]$^+$ 430.0727, found 430.0727.

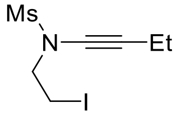

1r

***N*-(2-Iodoethyl)-*N*-(methanesulfonyl)but-1-yn-1-ylamine 1r**. Prepared according to general procedure I (630 µmol of crude hydroxyethyl-ynamide **S2r**, 1.1 equiv. of I_2, 1.5 equiv. of PPh_3, 1.5 equiv. of imidazole, 0.20 M in dichloromethane, 50 °C, 3 hours). Yield: 43% (82 mg, 271 µmol). Solvent system for flash column chromatography: petroleum ether/EtOAc: 90/10; Yellow oil; ^{1}H NMR (400 MHz, $CDCl_3$): δ 3.77 (t, J = 7.4 Hz, 2H), 3.36 (t, J = 7.4 Hz, 2H), 3.12 (s, 3H), 2.31 (t, J = 7.5 Hz, 2H), 1.16 (t, J = 7.5 Hz, 3H); ^{13}C NMR (100 MHz, $CDCl_3$): δ 73.4, 70.9, 53.4, 38.7, 14.2, 12.4, 0.0; ESIHRMS m/z calcd for $C_7H_{13}INO_2S$ [M+H]$^+$ 301.9706, found 301.9710.

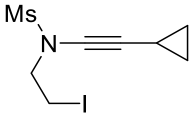

1s

***N*-(2-Iodoethyl)-*N*-(methanesulfonyl)cyclopropylethynylamine 1s**. Prepared according to general procedure I (1.75 mmol of hydroxyethyl-ynamide **S2s** , 1.1 equiv. of I_2, 1.5 equiv. of PPh_3, 1.5 equiv. of imidazole, 0.20 M in dichloromethane, rt, 5 hours). Yield: 58% (318 mg, 1.01 mmol). Solvent system for flash column chromatography: petroleum ether/EtOAc: 80/20; Orange oil; ^{1}H NMR (400 MHz): δ 3.76 (t, J = 7.5 Hz, 2H), 3.34 (t, J = 7.5 Hz, 2H), 3.11 (s, 3H), 1.33 (tt, J = 8.1 and 4.9 Hz, 1H), 0.86-0.80 (m, 2H), 0.73-0.68 (m, 2H); ^{13}C NMR (100 MHz, $CDCl_3$): 76.5, 67.1, 53.3, 38.8, 9.1, 0.1, -0.7; ESIHRMS m/z calcd for $C_8H_{13}INO_2S$ [M+H]$^+$ 313.9706, found 313.9709.

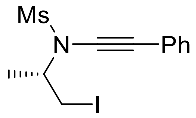

1t

(*S*)-*N*-(1-Iodopropan-2-yl)-*N*-(methanesulfonyl)phenylethynylamine 1t. Prepared according to general procedure I (395 µmol of hydroxyethyl-ynamide **S2t**, 1.1 equiv. of I_2, 1.5 equiv. of PPh_3, 1.5 equiv. of imidazole, 0.20 M in dichloromethane, rt, 5 hours). Yield: 42% (60 mg, 165 µmol). Solvent system for flash column chromatography: petroleum ether/EtOAc: 90/10; Yellow oil; $[\alpha]_D^{25}$ + 103 (*c* 1.4, $CHCl_3$); ^1H NMR (400 MHz, $CDCl_3$): *δ* 7.47-7.43 (m, 2H), 7.35-7.30 (m, 3H), 4.34 (app. dquint., *J* = 8.6 and 6.5 Hz, 1H), 3.44 (A of ABX syst., *J* = 10.6 and 8.5 Hz, 1H), 3.34 (B of ABX syst., *J* = 10.6 and 5.6 Hz, 1H), 3.27 (s, 3H), 1.53 (d, *J* = 6.6 Hz, 3H); ^{13}C NMR (100 MHz, $CDCl_3$): *δ* 131.9, 128.5, 128.5, 122.5, 77.3, 74.3, 57.9, 40.1, 20.0, 7.0; ESIHRMS *m/z* calcd for $C_{12}H_{15}INO_2S$ $[M+H]^+$ 363.9863, found 363.9859.

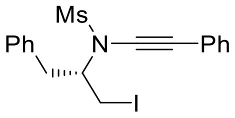

1u

(*S*)-*N*-(1-Iodo-3-phenylpropan-2-yl)-*N*-(methanesulfonyl)phenylethynylamine 1u. Prepared according to general procedure I (1.97 mmol of hydroxyethyl-ynamide **S2u**, 1.1 equiv. of I_2, 1.5 equiv. of PPh_3, 1.5 equiv. of imidazole, 0.20 M in dichloromethane, 40 °C, 5 hours). Yield: 81% (697 mg, 1.59 mmol). Solvent system for flash column chromatography: petroleum ether/EtOAc: 95/5; Colorless oil; $[\alpha]_D^{20}$ - 75 (*c* 1.0, $CHCl_3$); ^1H NMR (400 MHz, $CDCl_3$): *δ* 7.52-7.47 (m, 2H), 7.38-7.32 (m, 7H), 7.31-7.27 (m, 1H), 4.44 (X of ABX and A'B'X syst., *J* = 10.0, 7.7, 6.3 and 4.9 Hz, 1H), 3.50 (A of ABX syst., *J* = 10.6 and 7.7 Hz, 1H), 3.43 (B of ABX syst., *J* = 10.6 and 6.4 Hz, 1H), 3.28 (A' of A'B'X syst., *J* = 14.0 and 4.9 Hz, 1H), 3.03 (B' of A'B'X syst., *J* = 14.0 and 10.0 Hz, 1H), 2.48 (s, 3H); ^{13}C NMR (100 MHz, $CDCl_3$): *δ* 137.2, 131.9, 129.7, 129.0, 128.6, 128.5, 127.6, 122.5, 77.5, 74.9, 64.0, 39.3, 39.0, 5.9; ESIHRMS *m/z* calcd for $C_{18}H_{19}INO_2S$ $[M+H]^+$ 440.0176, found 440.0184.

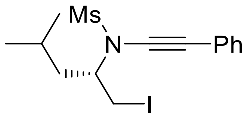

1v

(*S*)-*N*-(1-Iodo-4-methylpentan-2-yl)-*N*-(methanesulfonyl)phenylethynylamine 1v. Prepared according to general procedure IV (8.57 mmol of methanesulfonyloxyethyl-ynamide **S3v**, 5.0 equiv. of NaI, 0.3 M in acetone, 70 °C, overnight). Yield: 80% (2.77 g, 6.83 mmol). Solvent system for flash column chromatography: petroleum ether/EtOAc: 90/10; Orange oil; $[\alpha]_D^{20}$ + 44 (*c* 1.3, $CHCl_3$); ^1H NMR (400 MHz, $CDCl_3$): δ 7.46-7.41 (m, 2H), 7.34-7.29 (m, 3H), 4.33-4.25 (m, 1H), 3.41 (A of ABX syst., *J* = 10.6 and 8.5 Hz, 1H), 3.34 (obs. B of ABX syst., *J* = 10.6 and 5.2 Hz, 1H), 3.34 (s, 3H), 1.84-1.71 (m, 2H), 1.58-1.48 (m, 1H), 0.99 (d, *J* = 6.5 Hz, 6H); ^{13}C NMR (100 MHz, $CDCl_3$): δ 131.9, 128.5, 128.4, 122.5, 74.5, 60.5, 42.7, 40.1, 25.3, 23.4, 21.7, 6.8 (one carbon obscured by $CDCl_3$); ESIHRMS *m/z* calcd for $C_{15}H_{21}INO_2S$ $[M+H]^+$ 406.0332, found 406.0329.

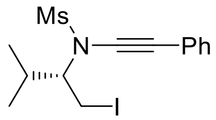

1w

(*S*)-*N*-(1-Iodo-3-methylbutan-2-yl)-*N*-(methanesulfonyl)phenylethynylamine 1w. Prepared according to general procedure IV (3.0 mmol of methanesulfonyloxyethyl-ynamide **S3w**, 5.0 equiv. of NaI, 0.2 M in acetone, 70 °C, 48 hours). Yield: 88% (1.03 g, 2.63 mmol). Solvent system for flash column chromatography: petroleum ether/EtOAc: 95/5; Yellow oil; $[\alpha]_D^{20}$ + 112 (*c* 2.0, $CHCl_3$); ^1H NMR (400 MHz, $CDCl_3$): δ 7.47-7.41 (m, 2H), 7.36-7.29 (m, 3H), 3.94 (ddd, *J* = 10.7, 8.0 and 3.0 Hz, 1H), 3.58 (dd, *J* = 11.0 and 3.0 Hz, 1H), 3.47 (app. t, *J* = 10.7 Hz, 1H), 3.38 (s, 3H), 2.05 (app. oct., *J* = 7.2 Hz, 1H), 1.14 (d, *J* = 6.8 Hz, 3H), 1.08 (d, *J* = 6.8 Hz, 3H); ^{13}C NMR (100 MHz, $CDCl_3$): δ 131.8, 128.5, 128.3, 122.6, 78.2, 74.3, 68.0, 39.8, 33.3, 20.1, 19.5, 5.0; ESIHRMS *m/z* calcd for $C_{14}H_{19}INO_2S$ $[M+H]^+$ 392.0176, found 392.0167.

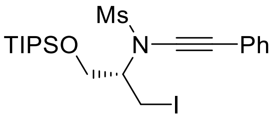

1x

(*S*)-*N*-{1-Iodo-3-[(triisopropylsilyl)oxy]propan-2-yl}-*N*-(methanesulfonyl)phenylethynyl amine 1x. Prepared according to general procedure IV (817 µmol of methanesulfonyloxyethyl-ynamide **S3x**, 5.0 equiv. of I_2, 0.2 M in acetone, 70 °C, overnight). Yield: 83% (363 mg, 678 µmol). Solvent system for flash column chromatography: petroleum ether/EtOAc: gradient from 100/0 to 90/10; Colorless oil; $[\alpha]_D^{25}$ + 9 (*c* 0.9, $CHCl_3$); ^1H NMR (400 MHz, $CDCl_3$): δ 7.45-7.40 (m, 2H), 7.33-7.29 (m, 3H), 4.27 (ddt, *J* = 8.7, 7.0 and 5.8 Hz, 1H), 4.00 (A of ABX syst., *J* = 10.4 and 7.0 Hz, 1H), 3.95 (B of ABX syst., *J* = 10.4 and 6.0 Hz, 1H), 3.49 (A' of A'B'X syst., *J* = 10.8 and 5.7 Hz, 1H), 3.39 (B' of A'B'X syst., *J* = 10.8 and 8.7 Hz, 1H), 3.29 (s, 3H), 1.17-1.02 (m, 21H); ^{13}C NMR (100 MHz, $CDCl_3$): δ 131.9, 128.5, 128.4, 122.5, 77.6, 73.9, 64.1, 63.7, 39.7, 18.1, 12.0, 1.8; ESIHRMS *m/z* calcd for $C_{21}H_{35}INO_3SSi$ $[M+H]^+$ 536.1146, found 536.1149.

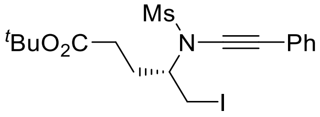

1y

(*S*)-*N*-(4-Iodo-1-*tert*-butoxycarbonylbutan-3-yl)-*N*-(methanesulfonyl)phenylethynylamine 1y. Prepared according to general procedure IV (184 µmol of methanesulfonyloxyethyl-ynamide **S3y**, 5.0 equiv. of NaI, 0.3 M in acetone, 70 °C, overnight). Yield: 56% (49 mg, 103 µmol). Solvent system for flash column chromatography: petroleum ether/EtOAc: 80/20; Orange oil; $[\alpha]_D^{25}$ + 4 (*c* 1.9, $CHCl_3$); ^1H NMR (400 MHz, $CDCl_3$): δ 7.46-7.40 (m, 2H), 7.34-7.30 (m, 3H), 4.28 (dddd, *J* = 10.4, 8.9, 5.2 and 3.8 Hz, 1H), 3.44 (A of ABX syst., *J* = 10.8 and 8.8 Hz, 1H), 3.38 (B of ABX syst., *J* = 10.8 and 5.2 Hz, 1H), 3.35 (s, 3H), 2.52-2.35 (m, 2H), 2.13 (app. dtd, *J* = 14.3, 7.8 and 3.7 Hz, 1H), 1.96 (dddd, *J* = 14.3, 10.3, 7.4 and 5.8 Hz, 1H), 1.46 (s, 9H); ^{13}C NMR (100 MHz, $CDCl_3$): δ 171.8, 131.9, 128.5 (2C), 122.3, 81.1, 77.0, 74.5, 61.4, 40.1, 32.0, 28.8, 28.2, 5.8; ESIHRMS *m/z* calcd for $C_{18}H_{24}INO_4SNa$ $[M+Na]^+$ 500.0363, found 500.0364.

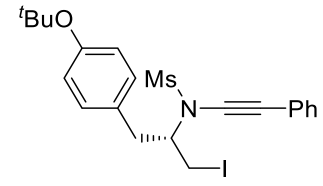

1z

(*S*)-*N*-[1-Iodo-3-(4-*tert*-butoxyphenyl)propan-2-yl]-*N*-(methanesulfonyl)phenylethynyl amine 1z. Prepared according to general procedure I (697 µmol of hydroxyethyl-ynamide **S2z**, 1.1 equiv. of I_2, 1.5 equiv. of PPh_3, 1.5 equiv. of imidazole, 0.20 M in dichloromethane, 50 °C, 5 hours). Yield: 87% (310 mg, 606 µmol). Solvent system for flash column chromatography: petroleum ether/EtOAc: gradient from 95/5 to 90/10; Colorless oil; $[\alpha]_D^{20}$ - 105 (*c* 1.1, $CHCl_3$); ^1H NMR (400 MHz, $CDCl_3$): δ 7.51-7.45 (m, 2H), 7.37-7.33 (m, 3H), 7.23 (d, *J* = 8.5 Hz, 2H), 6.97 (d, *J* = 8.4 Hz, 2H), 4.40 (X of ABX and A'B'X syst., *J* = 10.2, 7.5, 6.5 and 4.6 Hz, 1H), 3.49 (A of ABX syst., *J* = 10.6 and 7.5 Hz, 1H), 3.43 (B of ABX syst., *J* = 10.5 and 6.5 Hz, 1H), 3.24 (A' of A'B'X syst., *J* = 14.1 and 4.6 Hz, 1H), 2.97 (B' of A'B'X syst., *J* = 14.1 and 10.3 Hz, 1H), 2.48 (s, 3H), 1.32 (s, 9H); ^{13}C NMR (100 MHz, $CDCl_3$): δ 154.9, 132.1, 131.9, 130.2, 128.6, 128.5, 124.7, 122.5, 78.8, 77.6, 74.9, 64.1, 39.1, 38.4, 29.0, 5.9; ESIHRMS *m/z* calcd for $C_{22}H_{27}INO_3S$ $[M+H]^+$ 512.0751, found 512.0745.

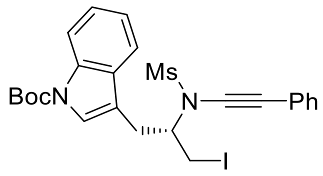

1aa

(*S*)-*N*-[1-(*N*-*tert*-Butoxycarbonyl-1*H*-indol-3-yl)-3-iodopropan-2-yl]-*N*-(methanesulfonyl) phenylethynylamine 1aa. Prepared according to general procedure I (299 µmol of hydroxyethyl-ynamide **S2aa**, 1.3 equiv. of I_2, 1.5 equiv. of PPh_3, 1.5 equiv. of imidazole, 0.20 M in dichloromethane, 40 °C, overnight). Yield: 95% (164 mg, 283 µmol). Solvent system for flash column chromatography: petroleum ether/EtOAc: 90/10; Orange oil; $[\alpha]_D^{20}$ - 36 (*c* 1.0, $CHCl_3$); ^1H NMR (400 MHz, $CDCl_3$): δ 8.15 (d, *J* = 7.9 Hz, 1H), 7.66 (d, *J* = 7.3 Hz, 1H), 7.58 (s, 1H), 7.53-7.45 (m, 2H), 7.38-7.33 (m, 4H), 7.30 (td, *J* = 7.5 and 1.2 Hz, 1H), 4.56-4.47 (m, 1H), 3.51 (A of ABX syst., *J* = 10.6 and 8.2 Hz, 1H), 3.46 (B of ABX syst., *J* = 10.6 and 5.9 Hz, 1H), 3.31 (A' of A'B'X syst., *J* = 14.8 and 6.7 Hz, 1H), 3.24 (B' of A'B'X syst., *J* = 14.8 and 7.9 Hz, 1H), 2.85

(s, 3H), 1.66 (s, 9H); ^{13}C NMR (100 MHz, $CDCl_3$): δ 149.6, 135.6, 132.1, 129.9, 128.6, 128.6, 125.1, 124.8, 123.2, 122.4, 119.1, 115.6, 115.6, 84.1, 77.3, 74.8, 61.9, 39.6, 29.8, 28.3, 5.7; ESIHRMS *m/z* calcd for $C_{25}H_{31}IN_3O_4S$ $[M+NH_4]^+$ 596.1074, found 596.1057.

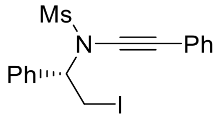

1ab

(*S*)-*N*-(2-Iodo-1-phenylethyl)-*N*-(methanesulfonyl)phenylethynylamine 1ab. Prepared according to general procedure I (1.36 mmol of hydroxyethyl-ynamide **S2ab**, 1.1 equiv. of I_2, 1.5 equiv. of PPh_3, 1.5 equiv. of imidazole, 0.20 M in dichloromethane, 65 °C, 6 hours). Yield: 84% (487 mg, 1.15 mmol). Solvent system for flash column chromatography: petroleum ether/EtOAc: 90/10; Yellow oil; $[\alpha]_D^{20}$ - 72 (*c* 1.3, $CHCl_3$); ^1H NMR (400 MHz, $CDCl_3$): δ 7.52-7.46 (m, 4H), 7.45-7.38 (m, 3H), 7.37-7.32 (m, 3H), 5.26 (X of ABX syst., *J* = 10.0 and 5.6 Hz, 1H), 3.84 (A of ABX syst., *J* = 10.8 and 10.0 Hz, 1H), 3.65 (B of ABX syst., *J* = 10.8 and 5.6 Hz, 1H), 2.97 (s, 3H); ^{13}C NMR (100 MHz, $CDCl_3$): δ 137.6, 131.9, 129.5, 129.3, 128.6 (2C), 127.0, 122.4, 78.3, 74.7, 65.2, 39.4, 4.5; ESIHRMS *m/z* calcd for $C_{17}H_{17}INO_2S$ $[M+H]^+$ 426.0019, found 426.0013.

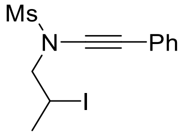

1ac

***N*-(2-Iodopropyl)-*N*-(methanesulfonyl)phenylethynylamine 1ac**. Prepared according to general procedure I (553 µmol of hydroxyethyl-ynamide **S2ac**, 1.3 equiv. of I_2, 1.5 equiv. of PPh_3, 1.5 equiv. of imidazole, 0.20 M in dichloromethane, 40 °C, overnight). Yield: 63% (126 mg, 347 µmol). Solvent system for flash column chromatography: petroleum ether/EtOAc: 90/10; Orange oil; ^1H NMR (400 MHz, $CDCl_3$): δ 7.45-7.41 (m, 2H), 7.34-7.30 (m, 3H), 4.39 (app. dquint., *J* = 8.6 and 6.8 Hz, 1H), 3.98 (A of ABX syst., *J* = 13.8 and 6.5 Hz, 1H), 3.75 (B of ABX syst., *J* = 13.8 and 8.7 Hz, 1H), 3.22 (s, 3H), 2.01 (d, *J* = 6.8 Hz, 3H); ^{13}C NMR (100 MHz, $CDCl_3$):

δ 131.8, 128.5 (2C), 122.2, 80.9, 72.0, 60.6, 39.1, 25.2, 21.1; ESIHRMS *m/z* calcd for $C_{12}H_{18}IN_2O_2S$ $[M+NH_4]^+$ 381.0128, found 381.0132.

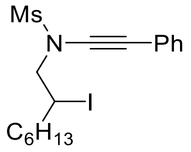

1ad

***N*-(2-Iodooctyl)-*N*-(methanesulfonyl)phenylethynylamine 1ad**. Prepared according to general procedure I (247 µmol of hydroxyethyl-ynamide **S2ad**, 1.3 equiv. of I_2, 1.4 equiv. of PPh_3, 1.4 equiv. of imidazole, 0.20 M in dichloromethane, 55 °C, overnight). Yield: 59% (63 mg, 145 µmol). Solvent system for flash column chromatography: petroleum ether/EtOAc: 95/5 then 90/10; Colorless oil; ^1H NMR (400 MHz, $CDCl_3$): δ 7.45-7.40 (m, 2H), 7.34-7.29 (m, 3H), 4.36 (tdd, *J* = 8.7, 6.7 and 3.8 Hz, 1H), 4.01 (A of ABX syst., *J* = 13.8 and 6.8 Hz, 1H), 3.84 (B of ABX syst., *J* = 13.8 and 8.4 Hz, 1H), 3.21 (s, 3H), 1.96-1.86 (m, 1H), 1.81 (dtd, *J* = 14.4, 9.4 and 4.6 Hz, 1H), 1.68-1.55 (m, 1H), 1.49-1.24 (m, 7H), 0.88 (app. t, *J* = 6.8 Hz, 3H); ^{13}C NMR (100 MHz, $CDCl_3$): δ 131.8, 128.5 (2C), 122.2, 81.0, 72.0, 59.0, 38.9, 36.3, 31.7, 31.5, 29.3, 28.5, 22.7, 14.1; ESIHRMS *m/z* calcd for $C_{17}H_{28}IN_2O_2S$ $[M+NH_4]^+$ 451.0911, found 451.0910.

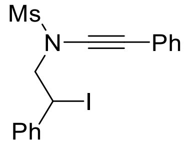

1ae

***N*-(2-Iodo-2-phenylethyl)-*N*-(methanesulfonyl)phenylethynylamine 1ae**. Prepared according to general procedure IV (356 µmol of methanesulfonyloxyethyl-ynamide **S3ae**, 5.0 equiv. of NaI, 0.10 M in acetone, 70 °C, overnight). Yield: 36% (55 mg, 129 µmol). Solvent system for flash column chromatography: petroleum ether/dichloromethane: 50/50; Yellow oil; ^1H NMR (400 MHz, $CDCl_3$): δ 7.55-7.50 (m, 2H), 7.42-7.30 (m, 8H), 5.38 (X of ABX syst., *J* = 10.4 and 6.0 Hz, 1H), 4.40 (A of ABX syst., *J* = 14.0 and 10.4 Hz, 1H), 4.22 (B of ABX syst., *J* = 14.0 and 6.0 Hz, 1H), 2.63 (s, 3H); ^{13}C NMR (100 MHz, $CDCl_3$): δ 140.0, 131.9, 129.1 (2C), 128.5, 128.5, 128.5,

122.2, 80.2, 72.6, 58.9, 38.7, 26.3; ESIHRMS *m/z* calcd for $C_{17}H_{17}INO_2S$ [M+H]$^+$ 426.0019, found 426.0028.

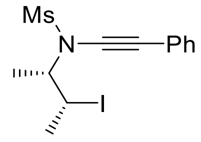

1af

***N*-[(2*S*,3*R*)-3-Iodobutan-2-yl]-*N*-(methanesulfonyl)phenylethynylamine 1af**. Prepared according to general procedure I (380 µmol of hydroxyethyl-ynamide **S2af**, 1.0 equiv. of I_2, 1.5 equiv. of PPh_3, 1.5 equiv. of imidazole, 0.10 M in dichloromethane, 60 °C, overnight). Yield: 49% (70 mg, 185 µmol). Solvent system for flash column chromatography: petroleum ether/EtOAc: 90/10; Colorless oil; $[\alpha]_D^{25}$ + 71 (*c* 1.0, $CHCl_3$); ^{1}H NMR (400 MHz, $CDCl_3$): δ 7.46-7.43 (m, 2H), 7.34-7.30 (m, 3H), 4.39 (app. quint., *J* = 7.2 Hz, 1H), 4.24 (app. quint., *J* = 6.9 Hz, 1H), 3.26 (s, 3H), 2.00 (d, *J* = 7.0 Hz, 3H), 1.50 (d, *J* = 6.7 Hz, 3H); ^{13}C NMR (100 MHz, $CDCl_3$): δ 131.8, 128.5, 128.4, 122.6, 78.3, 74.6, 62.6, 39.8, 28.9, 24.6, 16.9; ESIHRMS *m/z* calcd for $C_{13}H_{16}INO_2SNa$ [M+Na]$^+$ 399.9839, found 399.9859.

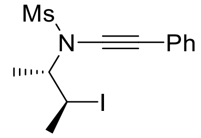

1af'

***N*-[(2*S*,3*S*)-3-Iodobutan-2-yl]-*N*-(methanesulfonyl)phenylethynylamine 1af'**. Prepared according to general procedure I (242 µmol of hydroxyethyl-ynamide **S2af'**, 1.0 equiv. of I_2, 1.5 equiv. of PPh_3, 1.5 equiv. of imidazole, 0.10 M in dichloromethane, 60 °C, overnight). Yield: 71% (65 mg, 172 µmol). Solvent system for flash column chromatography: petroleum ether/EtOAc: 90/10; Colorless oil; $[\alpha]_D^{25}$ + 18 (*c* 1.5, $CHCl_3$); ^{1}H NMR (400 MHz, $CDCl_3$): δ 7.45-7.40 (m, 2H), 7.34-7.30 (m, 3H), 4.23 (app. quint., *J* = 7.6 Hz, 1H), 4.09 (dq, *J* = 8.0 and 6.6 Hz, 1H), 3.18 (s, 3H), 2.03 (d, *J* = 6.9 Hz, 3H), 1.64 (d, *J* = 6.6 Hz, 3H); ^{13}C NMR (100 MHz, $CDCl_3$): δ 131.7, 128.5, 128.4, 122.5, 78.1, 74.2, 62.2, 39.7, 28.8, 26.3, 19.4; ESIHRMS *m/z* calcd for $C_{13}H_{16}INO_2SNa$ [M+Na]$^+$ 399.9839, found 399.9836.

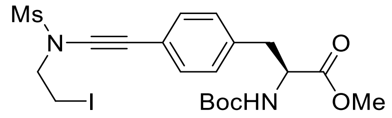

1ag

(*S*)-*N*-(2-Iodoethyl)-*N*-(methanesulfonyl){*p*-{2-[(*tert*-butoxycarbonyl)amino]-2-methoxy carbonylethyl}phenyl}ethynylamine 1ag. Prepared according to general procedure I (722 µmol of hydroxyethyl-ynamide **S2ag**, 1.3 equiv. of I_2, 1.5 equiv. of PPh_3, 1.5 equiv. of imidazole, 0.2 M in dichloromethane, rt, 5 hours). Yield: 66% (261 mg, 474 µmol). Solvent system for flash column chromatography: petroleum ether/EtOAc: 70/30; White solid; Mp: 90 °C; $[\alpha]_D^{20}$ - 0.09 (*c* 2.3, $CHCl_3$); ^1H NMR (400 MHz, $CDCl_3$): δ 7.35 (d, *J* = 8.2 Hz, 2H), 7.07 (d, *J* = 8.1 Hz, 2H), 4.99 (d, *J* = 7.9 Hz, 1H), 4.56 (app. q, *J* = 6.6 Hz, 1H), 3.89 (t, *J* = 7.3 Hz, 2H), 3.69 (s, 3H), 3.42 (t, *J* = 7.6 Hz, 2H), 3.20 (s, 3H), 3.11 (A of ABX syst., *J* = 13.8 and 5.8 Hz, 1H), 3.02 (B of ABX syst., *J* = 13.8 and 6.2 Hz, 1H), 1.41 (s, 9H); ^{13}C NMR (100 MHz, $CDCl_3$): δ 172.2, 155.1, 136.8, 132.0, 129.5, 120.8, 80.4, 80.1, 71.8, 54.4, 53.5, 52.4, 39.2, 38.3, 28.4, -0.2; ESIHRMS *m/z* calcd for $C_{20}H_{27}IN_2O_6SNa$ $[M+Na]^+$ 573.0527, found 573.0534.

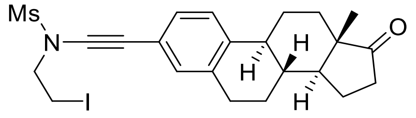

1ah

(8*R*,9*S*,13*S*,14*S*)-*N*-(2-Iodoethyl)-*N*-(methanesulfonyl)-(13-methyl-17-oxo-7,8,9,11,12,13, 14,15,16,17-decahydro-6*H*-cyclopenta[a]phenanthren-3-yl)ethynylamine 1ah. Prepared according to general procedure IV (405 µmol of methanesulfonyloxyethyl-ynamide **S3ah**, 5.0 equiv. of NaI, 0.1 M in acetone, 70 °C, 12 hours). Yield: 89% (189 mg, 360 µmol). Purification by precipitation/trituration in CH_2Cl_2/pentane, then in Et_2O ; White solid; Mp: 125 °C; $[\alpha]_D^{20}$ + 92 (*c* 1.0, $CHCl_3$); ^1H NMR (400 MHz, $CDCl_3$): δ 7.26-7.18 (m, 3H), 3.91 (t, *J* = 7.3 Hz, 2H), 3.43 (t, *J* = 7.4 Hz, 2H), 3.21 (s, 3H), 2.88 (dd, *J* = 9.0 and 4.5 Hz, 2H), 2.51 (dd, *J* = 19.0 and 8.8 Hz, 1H), 2.44-2.37 (m, 1H), 2.34-2.25 (m, 1H), 2.20-2.10 (m, 1H), 2.10-1.93 (m, 3H), 1.69-1.38 (m, 6H), 0.91 (s, 3H); ^{13}C NMR (100 MHz, $CDCl_3$): δ 220.8, 140.7, 136.9, 132.4, 129.2, 125.6, 119.4, 79.7, 72.1, 53.5, 50.6, 48.0, 44.6, 39.2, 38.1, 36.0, 31.7, 29.2, 26.4, 25.7, 21.7, 14.0, - 0.1; ESIHRMS *m/z* calcd for $C_{23}H_{29}INO_3S$ $[M+H]^+$ 526.0907, found 526.0913.

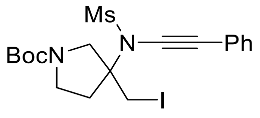

1ai

***N*-{1-(*tert*-Butoxycarbonyl)-3-(iodomethyl)pyrrolidin-3-yl}-*N*-(methanesulfonyl)phenyl ethynylamine 1ai**. Prepared according to general procedure III (190 µmol of hydroxyethyl-ynamide **S2ai**, 1.42 equiv. of I_2, 1.42 equiv. of PPh_3, 1.42 equiv. of imidazole, 0.1 M in toluene, 80 °C, 4 hours). Yield: 48% (46 mg, 91 µmol). Solvent system for flash column chromatography: pentane/Et_2O: 70/30; Yellow oil; ^{1}H NMR (400 MHz, $CDCl_3$): δ 7.45-7.40 (m, 2H), 7.34-7.27 (br. m, 3H), 4.28 (d, J = 12.6 Hz, 0.50H, rotamer), 4.15 (d, J = 12.4 Hz, 0.50H, rotamer), 3.88-3.71 (m, 2H), 3.69-3.57 (m, 2H), 3.56-3.46 (m, 1H), 3.35 (s, 3H), 2.85-2.71 (m, 1H), 2.33 (dt, J = 13.5 and 7.7 Hz, 1H), 1.42 (s, 9H); ^{13}C NMR (100 MHz, $CDCl_3$): δ 154.2, 131.8 and 131.4 (rotamers), 128.5 and 128.4 (rotamers), 128.2, 122.5 and 122.4 (rotamers), 80.4 and 80.2 (rotamers), 80.3, 73.8, 71.9 and 71.5 (rotamers), 55.7, 44.6 and 43.9 (rotamers), 41.1, 37.7 and 36.8 (rotamers), 28.5, 10.4; ESIHRMS *m/z* calcd for $C_{19}H_{26}IN_2O_4S$ $[M+H]^+$ 505.0652, found 505.0673.

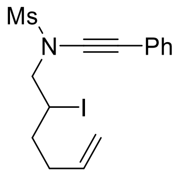

1aj

***N*-(2-Iodohex-5-en-1-yl)-*N*-(methanesulfonyl)phenylethynylamine 1aj**. Prepared according to general procedure I (716 µmol of OH-containing ynamide **S2aj**, 1.1 equiv. of I_2, 1.5 equiv. of PPh_3, 1.5 equiv. of imidazole, 0.175 M in dichloromethane, 50 °C, 6 hours). Yield: 83% (240 mg, 595 µmol). Solvent system for flash column chromatography: petroleum ether/EtOAc: 95/5; White solid; Mp: 61 °C; ^{1}H NMR (400 MHz, $CDCl_3$): δ 7.44-7.40 (m, 2H), 7.35-7.30 (m, 3H), 5.79 (dddd, J = 17.3, 10.2, 7.3 and 6.0 Hz, 1H), 5.13 (dq, J = 17.1 and 1.6 Hz, 1H), 5.04 (dq, J = 10.2 and 1.4 Hz, 1H), 4.34 (dddd, J = 10.0, 8.6, 6.6 and 3.6 Hz, 1H), 4.03 (A of ABX syst., J = 13.8 and 6.6 Hz, 1H), 3.87 (B of ABX syst., J = 13.8 and 8.6 Hz, 1H), 3.21 (s, 3H), 2.46-2.36 (m, 1H), 2.27-2.16 (m, 1H), 2.02 (dddd, J = 15.4, 8.6, 7.1 and 3.6 Hz, 1H), 1.90 (dddd, J = 14.7, 9.7, 8.6 and 5.0 Hz, 1H); ^{13}C NMR (100 MHz, $CDCl_3$): δ 136.4, 131.8, 128.5 (2C), 122.2, 116.4, 80.9,

72.2, 59.1, 39.0, 35.3, 33.4, 30.4; ESIHRMS *m/z* calcd for $C_{15}H_{19}INO_2S$ $[M+H]^+$ 404.0176, found 404.0176.

2.7. Experimental Procedures and Characterization Data: Synthesis of Chloroethyl-Ynamide $1a_{Cl}$

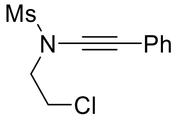

$1a_{Cl}$

***N*-(2-Chloroethyl)-*N*-(methanesulfonyl)phenylethynylamine $1a_{Cl}$.** An oven-dried pressure tube was charged with *N*-(2-iodoethyl)-*N*-(methanesulfonyl)phenylethynylamine **1a** (100 mg, 286 µmol) and tetraethylammonium chloride (156 mg, 945 µmol). The tube was fitted with a rubber septum, evacuated under high vacuum and backfilled with argon. Chloroform (1.2 mL) was then added, the rubber septum was replaced by a Teflon-coated screw cap and the resulting mixture was stirred at 80 °C for 48 hours. The reaction mixture was then cooled to rt, diluted with dichloromethane and washed with water and brine. The organic layer was then dried over $MgSO_4$, filtered and concentrated under reduced pressure. The crude residue was finally purified by flash column chromatography over silica gel (petroleum ether/EtOAc: gradient from 90/10 to 80/20) to afford the desired product as a colorless oil (55 mg, 213 µmol, 75%). ^1H NMR (400 MHz, $CDCl_3$): δ 7.45-7.40 (m, 2H), 7.34-7.29 (m, 3H), 3.93-3.89 (m, 2H), 3.85-3.81 (m, 2H), 3.23 (s, 3H); ^{13}C NMR (100 MHz, $CDCl_3$): δ 131.8, 128.5 (2C), 122.2, 80.3, 72.0, 52.7, 41.1, 39.1; ESIHRMS m/z calcd for $C_{12}H_{18}NO$ $[M+NH_4]^+$ 192.1383, found 192.1376.

2.8. Experimental Procedures and Characterization Data: Synthesis of Compound 7

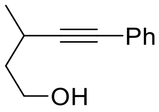

SAB

3-Methyl-5-phenylpent-4-yn-1-ol SAB. To a solution of tris(acetylacetonato)iron(III) (971 mg, 2.75 mmol) and sodium hydrogen carbonate (462 mg, 5.5 mmol) in absolute ethanol (45 mL) were added (bromoethynyl)benzene (996 mg, 5.5 mmol) and but-3-en-1-ol (1.42 mL, 16.5 mmol) under argon. The reaction mixture was then heated to 60 °C and a solution of phenylsilane (1.02 mL, 8.3 mmol) in ethanol (5 mL) was added dropwise. The resulting mixture was stirred at 60 °C for 2 hours, cooled to rt, diluted with dichloromethane (50 mL) and concentrated under reduced pressure. The crude residue was finally purified by flash column chromatography over silica gel (petroleum ether/EtOAc: 90/10) to afford the desired product as a yellow oil (460 mg, 2.64 mmol, 48%). ^{1}H NMR (400 MHz, $CDCl_3$): δ 7.40-7.38 (m, 2H), 7.29-7.27 (m, 3H), 3.91-3.81 (m, 2H), 2.85 (dddd, J = 13.9, 9.1, 6.9 and 5.4 Hz, 1H), 1.84-1.71 (m, 2H), 1.77 (obs. br. s, 1H), 1.30 (d, J = 7.0 Hz, 3H); ^{13}C NMR (100 MHz, $CDCl_3$): δ 131.7, 128.3, 127.8, 123.8, 93.8, 81.5, 61.3, 39.7, 23.6, 21.3; ESIHRMS m/z calcd for $C_{12}H_{18}NO$ $[M+NH_4]^+$ 192.1383, found 192.1376.

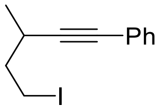

7

(5-Iodo-3-methylpent-1-yn-1-yl)benzene 7. To a solution of imidazole (164 mg, 2.41 mmol) and triphenylphosphine (632 mg, 2.41 mmol) in dichloromethane (4 mL) was added iodine (449 mg, 1.77 mmol) at 0 °C under argon. The mixture was stirred at 0 °C in the dark for 10 minutes. A solution of 3-methyl-5-phenylpent-4-yn-1-ol **SAB** (281 mg, 1.61 mmol) in dichloromethane (4 mL) was then added dropwise via *cannula*. The resulting mixture was stirred in the dark at 50 °C for 2 hours before being cooled to rt. The reaction mixture was then diluted with Et_2O, filtered over a short pad of Celite® (washed with Et_2O) and

concentrated under reduced pressure. The crude residue was finally purified by flash column chromatography over silica gel (petroleum ether/EtOAc: 90/10) to afford the desired product as a colorless oil (230 mg, 810 µmol, 50%). ^{1}H NMR (400 MHz, $CDCl_3$): δ 7.41-7.39 (m, 2H), 7.30-7.28 (m, 3H), 3.41 (dt, *J* = 9.7 and 6.5 Hz, 1H), 3.35 (dt, *J* = 9.7 and 7.7 Hz, 1H), 2.85 (app. sext., *J* = 7.0 Hz, 1H), 2.02 (dt, *J* = 7.6 and 6.5 Hz, 2H), 1.30 (d, *J* = 6.9 Hz, 3H); ^{13}C (100 MHz, $CDCl_3$): δ 131.8, 128.4, 127.9, 123.7, 92.4, 82.0, 40.6, 27.9, 20.7, 4.3; ESIHRMS m/z calcd for $C_{12}H_{14}I$ $[M+H]^+$ 285.0135, found 235.0135.

2.9. Experimental Procedures and Characterization Data: Copper-Catalyzed Photoredox Cyclization of Ynamides to Azetidines

General procedure

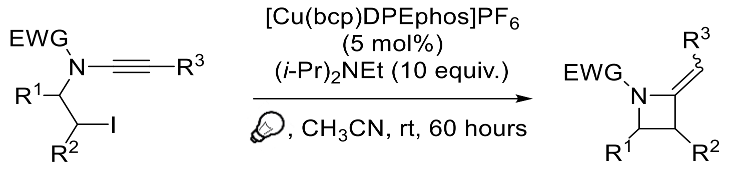

1 **2**

An oven-dried vial was charged with [Cu(bcp)DPEphos]PF_6 (5 mol%) and the iodoethyl-ynamide **1** (1 equiv.). The vial was fitted with a rubber septum, evacuated under high vacuum and backfilled with argon. Freshly distilled and degassed acetonitrile (0.1 M) and distilled *N*,*N*-diisopropylethylamine (10 equiv.) were then added and the reaction mixture was stirred under visible-light irradiation (using a LCZ-CCP-4V photoreactor and 420 nm tubes) or under blue LED irradiation (using either blue LED strips or an EvoluChem™ PhotoRedOx Box and a blue Kessil LED lamp) for 60 hours (unless otherwise stated) at rt. The reaction mixture was then filtered through a pad of Celite® (washed with Et_2O) and concentrated under reduced pressure. The crude residue was finally purified by flash column chromatography over silica gel to afford the desired azetidine **2**.

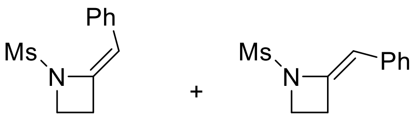

(*Z*)-2a **(*E*)-2a**

(*Z*)-2-Benzylidene-1-(methanesulfonyl)azetidine (*Z*)-2a and (***E***)**-2-benzylidene-1-(methanesulfonyl)azetidine (*E*)-2a**. Prepared according to general procedure (200 µmol of iodoethyl-ynamide **1a**). Yields: 71% (32 mg, 143 µmol) using blue LED strips, 67% (30 mg, 134 µmol) using a LCZ-CCP-4V photoreactor and 420 nm tubes and 60% (27 mg, 121 µmol) using a PhotoRedOx Box and a blue Kessil LED lamp. Obtained as mixtures of hardly separable *Z* and *E* isomers in *Z*/*E* ratios of 85/15 (blue LED strips), 85/15 (LCZ-CCP-4V photoreactor and 420 nm tubes) and 73/27 (PhotoRedOx Box and a blue Kessil LED lamp) in the crude reaction mixtures and 86/14 (blue LED strips), 88/12 (LCZ-CCP-4V photoreactor and 420 nm tubes) and 72/28 (PhotoRedOx Box and a blue Kessil LED lamp) after purification.

- **(*Z*)-2-Benzylidene-1-(methanesulfonyl)azetidine (*Z*)-2a** (faster eluting isomer). Solvent system for flash column chromatography: petroleum ether/dichloromethane: gradient from 90/10 to 80/20; White solid; Mp: 93 °C; ^{1}H NMR (300 MHz, $CDCl_3$): *δ* 7.39-7.17 (m, 5H), 5.74 (br. s, 1H), 4.08 (t, *J* = 7.0 Hz, 2H), 3.02 (td, *J* = 6.9 and 1.9 Hz, 2H), 2.67 (s, 3H); ^{13}C NMR (100 MHz, $CDCl_3$): *δ* 140.3, 134.4, 129.2, 128.1, 127.2, 109.2 48.7, 38.2, 26.8; ESIHRMS *m/z* calcd for $C_{11}H_{14}NO_2S$ $[M+H]^+$ 224.0740, found 224.0739.

  The Z configuration of the double bond of this isomer has been attributed on the basis of NOESY experiments (see page 353 for full NOESY spectrum).

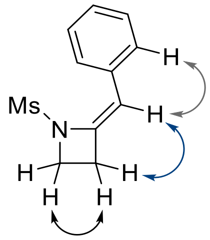

- **(*E*)-2-Benzylidene-1-(methanesulfonyl)azetidine (*E*)-2a** (slower eluting isomer).* Solvent system for flash column chromatography: petroleum ether/dichloromethane: gradient from 90/10 to 80/20; Off-white solid; Mp: 106 °C; ^{1}H NMR (300 MHz, $CDCl_3$): *δ* 7.35-7.27 (m, 3H), 7.18-7.12 (m, 2H), 6.25 (t, *J* = 2.4 Hz, 1H), 4.08 (t, *J* = 6.5 Hz, 2H), 3.25 (td, *J* = 6.5 and 2.3 Hz, 2H), 2.99 (s, 3H); ^{13}C NMR (100 MHz, $CDCl_3$): *δ* 142.9, 135.6, 128.8, 126.7, 126.2, 106.9, 49.8, 34.5, 27.4; ESIHRMS *m/z* calcd for $C_{11}H_{14}NO_2S$ $[M+H]^+$ 224.0740, found 224.0739.

  The E configuration of the double bond of this isomer has been attributed on the basis of NOESY experiments (see page 354 for full NOESY spectrum).

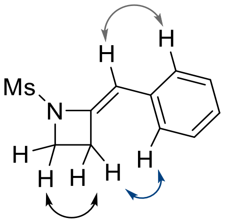

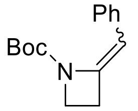

2b

2-Benzylidene-1-(*tert*-butoxycarbonyl)azetidine 2b. Prepared according to general procedure (200 µmol of iodoethyl-ynamide **1b**, blue LED strips). Yield: 59% (29 mg, 118 µmol). Obtained as a mixture of *Z* and *E* isomers in a *Z*/*E* ratio of 80/20 both in the crude reaction

* Compound **(*E*)-2a** could also be obtained by isomerization of **(*Z*)-2a** using catalytic iodine, see page 131 for detailed experimental procedure.

mixture and after purification.* Solvent system for flash column chromatography: petroleum ether/EtOAc: 96/4; Pale yellow oil; ^{1}H NMR (300 MHz, $CDCl_3$): *δ* 7.32-7.07 (m, 5H), 6.55 and 6.16 (br. s and br. s, 0.20H, *E* isomer, rotamers), 5.51 (t, *J* = 1.9 Hz, 0.80H, *Z* isomer), 3.98 (t, *J* = 6.4 Hz, 2H), 3.10 (br. app. t, *J* = 5.5 Hz, 0.40H, *E* isomer), 2.94 (td, *J* = 6.4 and 2.0 Hz, 1.60H, *Z* isomer), 1.58-1.49 (br. m, 1.80 H, *E* isomer), 1.16 (s, 7.20H, *Z* isomer); ^{13}C NMR (75 MHz, $CDCl_3$), *Z* isomer†: *δ* 152.7, 140.1, 136.8, 128.8, 127.5, 125.6, 105.6, 81.1, 47.4, 27.8, 27.6; ESIHRMS *m/z* calcd for $C_{15}H_{20}NO_2$ $[M+H]^+$ 246.1489, found 246.1484.

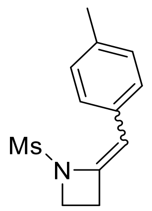

2c

1-(Methanesulfonyl)-2-(*p*-methylbenzylidene)azetidine 2c. Prepared according to general procedure (200 µmol of iodoethyl-ynamide **1c**, blue LED strips). Yield: 67% (32 mg, 135 µmol). Obtained as a mixture of *Z* and *E* isomers in a *Z*/*E* ratio of 80/20 in the crude reaction mixture and 84/16 after purification. Solvent system for flash column chromatography: petroleum ether/EtOAc: gradient from 90/10 to 70/30; Pale brown solid; Mp: 94 °C; ^{1}H NMR (600 MHz, $CDCl_3$): *δ* 7.25 (d, *J* = 7.9 Hz, 2H), 7.10 (d, *J* = 7.9 Hz, 1.68H, *Z* isomer), 7.04 (d, *J* = 8.0 Hz, 0.32H, *E* isomer), 6.22 (t, *J* = 2.3 Hz, 0.16H, *E* isomer), 5.70 (br. s, 0.84H, *Z* isomer), 4.07 (t, *J* = 7.1 Hz, 2H), 3.22 (td, *J* = 6.5 and 2.3 Hz, 0.32H, *E* isomer), 3.01 (td, *J* = 7.0 and 2.0 Hz, 1.68H, *Z* isomer), 2.97 (s, 0.48H, *E* isomer), 2.69 (s, 2.52H, *Z* isomer), 2.32 (br. s, 3H); ^{13}C NMR (150 MHz, $CDCl_3$): *δ* 142.1 (*E* isomer), 139.7 (*Z* isomer), 136.9 (*Z* isomer), 136.0 (*E* isomer), 132.7 (*E* isomer), 131.4 (*Z* isomer), 129.5 (*E* isomer), 129.1 (*Z* isomer), 128.8 (*Z* isomer), 126.6 (*E* isomer), 109.4 (*Z* isomer), 107.0 (*E* isomer), 49.7 (*E* isomer), 48.7 (*Z* isomer), 38.1 (*Z* isomer), 34.3 (*E* isomer), 27.3 (*E* isomer), 26.9 (*Z* isomer), 21.4 (*Z* isomer), 21.2 (*E* isomer); ESIHRMS *m/z* calcd for $C_{12}H_{16}NO_2S$ $[M+H]^+$ 238.0896, found 238.0895.

---

* Elucidation of the structures of the *Z* and *E* isomers has been done via NOESY experiments (see page 355 for details).

† Due to the minor amount of the *E* isomer and the presence of rotamers, some signals of this minor isomer could not be observed. For clarity, only the signals corresponding to the major *Z* isomer are reported.

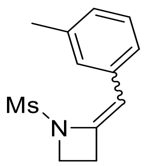

2d

1-(Methanesulfonyl)-2-(*m*-methylbenzylidene)azetidine 2d. Prepared according to general procedure (200 µmol of iodoethyl-ynamide **1d**, blue LED strips). Yield: 72% (34 mg, 143 µmol). Obtained as a mixture of *Z* and *E* isomers in a *Z*/*E* ratio of 79/21 in the crude reaction mixture and 84/16 after purification. Solvent system for flash column chromatography: petroleum ether/EtOAc: gradient from 90/10 to 80/20; Yellow oil; ^{1}H NMR (400 MHz, $CDCl_3$): *δ* 7.22-7.13 (m, 2.68H, *E* + *Z* isomers), 7.04-7.00 (m, 0.84H, *Z* isomer), 7.00-6.93 (m, 0.48H, *E* isomer), 6.22 (t, *J* = 2.3 Hz, 0.16H, *E* isomer), 5.70 (br. t, *J* = 2.0 Hz, 0.84H, *Z* isomer), 4.07 (t, *J* = 7.0 Hz, 1.68H, *Z* isomer), 4.06 (t, *J* = 6.5 Hz, 0.32H, *E* isomer), 3.24 (td, *J* = 6.6 and 2.3 Hz, 0.32H, *E* isomer), 3.00 (td, *J* = 7.0 and 2.1 Hz, 1.68H, *Z* isomer), 2.97 (s, 0.48H, *E* isomer), 2.68 (s, 2.52H, *Z* isomer), 2.33 (s, 3H); ^{13}C NMR (100 MHz, $CDCl_3$): *δ* 142.8 (*E* isomer), 140.2 (*Z* isomer), 138.4 (*E* isomer), 137.6 (*Z* isomer), 135.5 (*E* isomer), 134.3 (*Z* isomer), 129.9 (*Z* isomer), 128.7 (*E* isomer), 128.0 (*Z* isomer), 127.9 (*Z* isomer), 127.6 (*E* isomer), 127.0 (*E* isomer), 126.1 (*Z* isomer), 123.6 (*E* isomer), 109.2 (*Z* isomer), 107.0 (*E* isomer), 49.7 (*E* isomer), 48.7 (*Z* isomer), 38.3 (*Z* isomer), 34.3 (*E* isomer), 27.4 (*E* isomer), 26.7 (*Z* isomer), 21.6 (*E* isomer), 21.4 (*Z* isomer); ESIHRMS *m/z* calcd for $C_{12}H_{16}NO_2S$ $[M+H]^+$ 238.0896, found 238.0895.

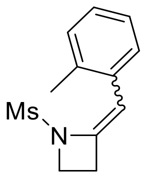

2e

1-(Methanesulfonyl)-2-(*o*-methylbenzylidene)azetidine 2e. Prepared according to general procedure (200 µmol of iodoethyl-ynamide **1e**, blue LED strips). Yield: 74% (35 mg, 147 µmol). Obtained as a mixture of *Z* and *E* isomers in a *Z*/*E* ratio of 84/16 in the crude reaction mixture and 87/13 after purification. Solvent system for flash column chromatography: petroleum ether/EtOAc: gradient from 90/10 to 80/20; Yellow oil; ^{1}H NMR (400 MHz, $CDCl_3$): *δ* 7.33-7.29 (m, 0.87H, *Z* isomer), 7.19-7.09 (m, 3.13H, *E* + *Z* isomers), 6.39 (t, *J* = 2.3 Hz, 0.13H, *E* isomer),

5.68 (br. t, *J* = 2.1 Hz, 0.87H, *Z* isomer), 4.06 (t, *J* = 6.8 Hz, 2H), 3.18 (td, *J* = 6.5 and 2.5 Hz, 0.26H, *E* isomer), 3.02 (td, *J* = 6.9 and 2.0 Hz, 1.74H, *Z* isomer), 3.00 (s, 0.39H, *E* isomer), 2.48 (s, 2.61H, *Z* isomer), 2.31 (s, 3H); ^{13}C NMR (100 MHz, CDCl$_3$): *δ* 142.9 (*E* isomer), 140.9 (*Z* isomer), 137.2 (*Z* isomer), 135.6 (*E* isomer), 134.1 (*Z* isomer), 133.8 (*E* isomer), 130.6 (*E* isomer), 130.0 (*Z* isomer), 129.8 (*Z* isomer), 127.6 (*Z* isomer), 126.5 (*E* isomer), 126.0 (*E* isomer), 125.9 (*E* isomer), 125.3 (*Z* isomer), 107.5 (*Z* isomer), 104.6 (*E* isomer), 49.4 (*E* isomer), 48.0 (*Z* isomer), 39.0 (*Z* isomer), 34.0 (*E* isomer), 26.9 (*E* isomer), 26.1 (*Z* isomer), 20.2 (*Z* isomer), 20.1 (*E* isomer); ESIHRMS *m/z* calcd for $C_{12}H_{16}NO_2S$ [M+H]$^+$ 238.0896, found 238.0893.

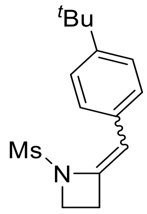

2f

1-(Methanesulfonyl)-2-(*p-tert*-butylbenzylidene)azetidine 2f. Prepared according to general procedure (200 µmol of iodoethyl-ynamide **1f**, blue LED strips). Yield: 81% (45 mg, 161 µmol). Obtained as a mixture of *Z* and *E* isomers in a *Z*/*E* ratio of 78/22 both in the crude reaction mixture and after purification. Solvent system for flash column chromatography: petroleum ether/EtOAc: gradient from 90/10 to 70/30; Pale orange oil; ^{1}H NMR (400 MHz, CDCl$_3$): *δ* 7.34 (obs. d, *J* = 8.4 Hz, 0.44H, *E* isomer), 7.32-7.28 (m, 3.12H, *Z* isomer), 7.10 (d, *J* = 8.4 Hz, 0.44 H, *E* isomer), 6.24 (t, *J* = 2.4 Hz, 0.22H, *E* isomer), 5.71 (t, *J* = 2.1 Hz, 0.78H, *Z* isomer), 4.08 (obs. t, *J* = 7.0 Hz, 1.56H, *Z* isomer), 4.07 (obs. t, *J* = 6.6 Hz, 0.44H, *E* isomer), 3.24 (td, *J* = 6.6 and 2.4 Hz, 0.44H, *E* isomer), 3.01 (td, *J* = 7.0 and 2.0 Hz, 1.56H, *Z* isomer), 2.97 (s, 0.66H, *E* isomer), 2.65 (s, 2.34H, *Z* isomer), 1.31 (s, 1.98H, *E* isomer), 1.30 (s, 7.02H, *Z* isomer); ^{13}C NMR (100 MHz, CDCl$_3$): *δ* 150.2 (*Z* isomer), 149.4 (*E* isomer), 142.3 (*E* isomer), 139.8 (*Z* isomer), 132.7 (*E* isomer), 131.4 (*Z* isomer), 129.0 (*Z* isomer), 126.4 (E isomer), 125.8 (*E* isomer), 125.1 (*Z* isomer), 109.5 (*Z* isomer), 106.9 (*E* isomer), 49.8 (*E* isomer), 48.6 (*Z* isomer), 38.3 (*Z* isomer), 34.7 (*Z* isomer), 34.7 (*E* isomer), 34.1 (*E* isomer), 31.5 (*Z* isomer), 31.4 (*E* isomer), 27.4 (*E* isomer), 27.0 (*Z* isomer); ESIHRMS *m/z* calcd for $C_{15}H_{22}NO_2S$ [M+H]$^+$ 280.1366, found 280.1368.

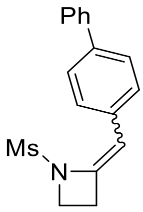

2g

1-(Methanesulfonyl)-2-(*p*-phenylbenzylidene)azetidine 2g. Prepared according to general procedure (200 µmol of iodoethyl-ynamide **1g**, blue LED strips). Yield: 70% (42 mg, 140 µmol). Obtained as a mixture of *Z* and *E* isomers in a *Z*/*E* ratio of 74/26 in the crude reaction mixture and 77/23 after purification. Solvent system for flash column chromatography: petroleum ether/EtOAc: gradient from 90/10 to 80/20; Off-white solid; Mp: 163 °C; ^{1}H NMR (400 MHz, $CDCl_3$): δ 7.62-7.58 (m, 2H), 7.55 (app. d, *J* = 8.2 Hz, 2H), 7.47-7.40 (m, 3.54H, *E* + *Z* isomers), 7.36-7.30 (m, 1H), 7.22 (d, *J* = 8.2 Hz, 0.46H, *E* isomer), 6.30 (t, *J* = 2.3 Hz, 0.23H, *E* isomer), 5.76 (br. t, *J* = 2.1 Hz, 0.77H, *Z* isomer), 4.10 (t, *J* = 6.9 Hz, 1.54H, *Z* isomer), 4.10 (obs. t, *J* = 6.4 Hz, 0.46H, *E* isomer), 3.28 (td, *J* = 6.5 and 2.3 Hz, 0.46H, *E* isomer), 3.05 (td, *J* = 7.0 and 2.1 Hz, 1.54H, *Z* isomer), 3.00 (s, 0.69H, *E* isomer), 2.75 (s, 2.31H, *Z* isomer); ^{13}C NMR (100 MHz, $CDCl_3$): 143.1 (*E* isomer), 140.8 (*Z* isomer), 140.7 (*E* isomer), 140.3 (*Z* isomer), 139.7 (*Z* isomer), 138.9 (*E* isomer), 134.7 (*E* isomer), 133.4 (*Z* isomer), 129.6 (*Z* isomer), 128.9 (*E* isomer), 128.8 (*Z* isomer), 127.5 (*E* isomer), 127.4 (*E* isomer), 127.4 (*Z* isomer), 127.0 (*E* + *Z* isomers), 126.9 (*E* isomer), 126.7 (*Z* isomer), 109.1 (*Z* isomer), 106.5 (*E* isomer), 49.8 (*E* isomer), 48.9 (*Z* isomer), 38.1 (*Z* isomer), 34.5 (*E* isomer), 27.4 (*E* isomer), 27.0 (*Z* isomer); ESIHRMS *m/z* calcd for $C_{17}H_{18}NO_2S$ $[M+H]^+$ 300.1053, found 300.1049.

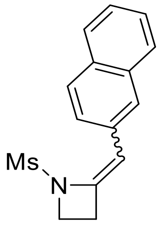

2h

1-(Methanesulfonyl)-2-(naphthalen-2-ylmethylene)azetidine 2h. Prepared according to general procedure (200 µmol of iodoethyl-ynamide **1h**, blue LED strips). Yield: 68% (37 mg, 135 µmol). Obtained as a mixture of *Z* and *E* isomers in a *Z*/*E* ratio of 43/57 in the crude

reaction mixture and 40/60 after purification. Solvent system for flash column chromatography: petroleum ether/EtOAc: gradient from 90/10 to 70/30; Off-white solid; Mp: 146 °C; ^{1}H NMR (400 MHz, $CDCl_3$): *δ* 7.82 (br. s, 0.40H, *Z* isomer), 7.80-7.74 (m, 3H), 7.57 (br. s, 0.60H, *E* isomer), 7.52 (dd, *J* = 8.5 and 1.7 Hz, 0.40H, *Z* isomer), 7.49-7.39 (m, 2H), 7.30 (dd, *J* = 8.6 and 1.7 Hz, 0.60H, *E* isomer), 6.41 (t, *J* = 2.3 Hz, 0.60H, *E* isomer), 5.87 (br. t, *J* = 2.2 Hz, 0.40H, *Z* isomer), 4.12 (obs. t, *J* = 6.8 Hz, 0.80H, *Z* isomer), 4.10 (obs. t, *J* = 6.5 Hz, 1.20H, *E* isomer), 3.33 (td, *J* = 6.5 and 2.3 Hz, 1.20H, *E* isomer), 3.06 (td, *J* = 7.0 and 2.1 Hz, 0.80H, *Z* isomer), 3.01 (s, 1.80H, *E* isomer), 2.68 (s, 1.20H, *Z* isomer); ^{13}C NMR (100 MHz, $CDCl_3$): *δ* 143.3 (*E* isomer), 140.6 (*Z* isomer), 133.8 (*E* isomer), 133.2 (*Z* isomer), 133.2 (*E* isomer), 132.5 (*Z* isomer), 131.9 (*E* + *Z* isomers), 128.4 (*E* isomer), 127.9 (*Z* isomer), 127.8 (*Z* isomer), 127.8 (*Z* isomer), 127.7 (2C, *E* isomer), 127.6 (*Z* isomer), 127.3 (*Z* isomer), 126.5 (*E* isomer), 126.2 (*Z* isomer), 125.9 (*Z* isomer), 125.7 (*E* isomer), 125.4 (*E* isomer), 124.8 (*E* isomer), 109.3 (*Z* isomer), 107.0 (*E* isomer), 49.8 (*E* isomer), 48.9 (*Z* isomer), 38.1 (*Z* isomer), 34.6 (*E* isomer), 27.5 (*E* isomer), 27.0 (*Z* isomer); ESIHRMS *m/z* calcd for $C_{15}H_{16}NO_2S$ $[M+H]^+$ 274.0896, found 274.0894.

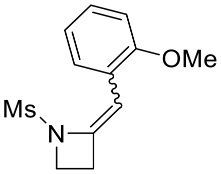

2i

1-(Methanesulfonyl)-2-(*o*-methoxybenzylidene)azetidine 2i. Prepared according to general procedure (200 µmol of iodoethyl-ynamide **1i**, blue LED strips). Yield: 83% (42 mg, 166 µmol). Obtained as a mixture of *Z* and *E* isomers in a *Z/E* ratio of 81/19 in the crude reaction mixture and 72/28 after purification. Solvent system for flash column chromatography: petroleum ether/EtOAc: gradient from 90/10 to 70/30; Off-white oil; ^{1}H NMR (400 MHz, $CDCl_3$): *δ* 7.43 (dd, *J* = 7.5 and 1.5 Hz, 0.72H, *Z* isomer), 7.22 (td, *J* = 7.8 and 1.7 Hz, 0.72H, *Z* isomer), 7.16 (td, *J* = 7.8 and 1.6 Hz, 0.28H, *E* isomer), 7.12 (dd, *J* = 7.7 and 1.6 Hz, 0.28H, *E* isomer), 6.91 (td, *J* = 7.5 and 0.8 Hz, 1H), 6.85 (td, *J* = 7.3 and 0.8 Hz, 1H), 6.54 (t, *J* = 2.4 Hz, 0.28H, *E* isomer), 5.72 (t, *J* = 2.0 Hz, 0.72H, *Z* isomer), 4.05 (obs. t, *J* = 6.9 Hz, 1.44H, *Z* isomer), 4.04 (obs. t, *J* = 6.5 Hz, 0.56H, *E* isomer), 3.83 (s, 2.16H, *Z* isomer), 3.82 (s, 0.84H, *E* isomer), 3.18 (td, *J* = 6.6 and 2.4

Hz, 0.56H, *E* isomer), 3.02 (td, *J* = 6.9 and 2.1 Hz, 1.44H, *Z* isomer), 3.00 (s, 0.84H, *E* isomer), 2.70 (s, 2.16H, *Z* isomer); ^{13}C NMR (100 MHz, $CDCl_3$): δ 156.9 (*Z* isomer), 156.1 (*E* isomer), 142.7 (*E* isomer), 140.8 (*Z* isomer), 131.0 (*Z* isomer), 128.7 (*Z* isomer), 127.5 (*E* isomer), 126.7 (*E* isomer), 124.3 (*E* isomer), 123.3 (*Z* isomer), 120.5 (*E* isomer), 120.1 (*Z* isomer), 110.9 (*E* isomer), 110.3 (*Z* isomer), 104.6 (*Z* isomer), 101.7 (*E* isomer), 55.6 (*Z* isomer), 55.6 (*E* isomer), 49.5 (*E* isomer), 48.8 (*Z* isomer), 37.5 (*Z* isomer), 34.2 (*E* isomer), 27.2 (*E* isomer), 26.5 (*Z* isomer); ESIHRMS *m/z* calcd for $C_{12}H_{16}NO_3S$ [M+H]$^+$ 254.0845, found 254,0849.

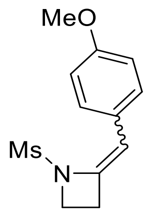

2j

1-(Methanesulfonyl)-2-(*p*-methoxybenzylidene)azetidine 2j. Prepared according to general procedure (200 µmol of iodoethyl-ynamide **1j**, blue LED strips). Yield: 79% (40 mg, 158 µmol). Obtained as a mixture of *Z* and *E* isomers in a *Z*/*E* ratio of 78/22 in the crude reaction mixture and 87/13 after purification. Solvent system for flash column chromatography: petroleum ether/EtOAc: gradient from 90/10 to 70/30; Yellow solid; Mp: 74 °C; ^{1}H NMR (400 MHz, $CDCl_3$): *δ* 7.30 (d, *J* = 8.6 Hz, 1.74H, *Z* isomer), 7.08 (d, *J* = 8.9 Hz, 0.26H, *E* isomer), 6.85 (obs. d, *J* = 8.9 Hz, 0.26H, *E* isomer), 6.83 (d, *J* = 8.8 Hz, 1.74H, *Z* isomer), 6.20 (t, *J* = 2.4 Hz, 0.13H, *E* isomer), 5.67 (t, *J* = 2.0 Hz, 0.87H, *Z* isomer), 4.05 (t, *J* = 7.0 Hz, 2H), 3.79 (s, 0.39H, *E* isomer), 3.78 (s, 2.61H, *Z* isomer), 3.20 (td, *J* = 6.5 and 2.3 Hz, 0.26H, *E* isomer), 3.00 (td, *J* = 7.0 and 2.1 Hz, 1.74H, *Z* isomer), 2.96 (s, 0.39H, *E* isomer), 2.70 (s, 2.61H, *Z* isomer); ^{13}C NMR (100 MHz, $CDCl_3$): *δ* 158.6 (*Z* isomer), 158.1 (*E* isomer), 141.0 (*E* isomer), 139.2 (*Z* isomer), 130.4 (*Z* isomer), 128.1 (*E* isomer), 127.8 (*E* isomer), 126.6 (*Z* isomer), 114.3 (*E* isomer), 113.5 (*Z* isomer), 109.2 (*Z* isomer), 106.7 (*E* isomer), 55.4 (*E* isomer), 55.3 (*Z* isomer), 49.6 (*E* isomer), 48.7 (*Z* isomer), 37.7 (*Z* isomer), 34.0 (*E* isomer), 27.1 (*E* isomer), 26.8 (*Z* isomer); ESIHRMS *m/z* calcd for $C_{12}H_{16}NO_3S$ [M+H]$^+$ 254.0845, found 254.0843.

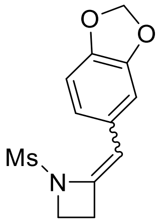

2k

2-(Benzo[*d*][1,3]dioxol-5-ylmethylene)-1-(methanesulfonyl)azetidine 2k. Prepared according to general procedure (200 µmol of iodoethyl-ynamide **1k**, blue LED strips). Yield: 68% (36 mg, 135 µmol). Obtained as a mixture of *Z* and *E* isomers with a *Z*/*E* ratio of 77/23 in the crude reaction mixture and 69/31 after purification. Solvent system for flash column chromatography: petroleum ether/EtOAc: gradient from 90/10 to 80/20; Orange oil; ^{1}H NMR (400 MHz, $CDCl_3$): δ 6.88 (d, *J* = 1.6 Hz, 0.69H, *Z* isomer), 6.81 (app. dd, *J* = 8.0 and 1.6 Hz, 0.69H, *Z* isomer), 6.75 (obs. d, *J* = 8.4 Hz, 0.31H, *E* isomer), 6.73 (obs. d, *J* = 8.0 Hz, 0.69H, *Z* isomer), 6.63 (d, *J* = 1.6 Hz, 0.31H, *E* isomer), 6.61 (dd, *J* = 8.0 and 1.7 Hz, 0.31H, *E* isomer), 6.17 (t, *J* = 2.4 Hz, 0.31H, *E* isomer), 5.93 (s, 0.62H, *E* isomer), 5.93 (s, 1.38H, *Z* isomer), 5.63 (t, *J* = 2.1 Hz, 0.69H, *Z* isomer), 4.05 (t, *J* = 6.9 Hz, 1.38H, *Z* isomer), 4.05 (obs. t, *J* = 6.5 Hz, 0.62H, *E* isomer), 3.19 (td, *J* = 6.5 and 2.4 Hz, 0.62H, *E* isomer), 3.00 (td, *J* = 7.0 and 2.1 Hz, 1.38H, *Z* isomer), 2.97 (s, 0.93H, *E* isomer), 2.76 (s, 2.07H, *Z* isomer); ^{13}C NMR (100 MHz, $CDCl_3$): δ 148.0 (*E* isomer), 147.4 (*Z* isomer), 146.6 (*Z* isomer), 146.0 (*E* isomer), 141.4 (*E* isomer), 139.5 (*Z* isomer), 129.7 (*E* isomer), 128.1 (*Z* isomer), 122.7 (*Z* isomer), 120.7 (*E* isomer), 109.5 (*Z* isomer), 109.2 (*Z* isomer), 108.7 (*E* isomer), 108.0 (*Z* isomer), 106.9 (*E* isomer), 106.4 (*E* isomer), 101.1 (*E* isomer), 101.1 (*Z* isomer), 49.6 (*E* isomer), 48.8 (*Z* isomer), 37.9 (*Z* isomer), 34.3 (*E* isomer), 27.2 (*E* isomer), 26.9 (*Z* isomer); ESIHRMS *m/z* calcd for $C_{12}H_{14}NO_4S$ $[M+H]^+$ 268.0638, found 268.0636.

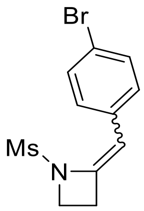

2l

2-(*p*-Bromobenzylidene)-1-(methanesulfonyl)azetidine 2l. Prepared according to general procedure (200 µmol of iodoethyl-ynamide **1l**, blue LED strips). Yield: 76% (46 mg, 152 µmol). Obtained as a mixture of *Z* and *E* isomers in a *Z/E* ratio of 83/17 in the crude reaction mixture and 86/14 after purification. Solvent system for flash column chromatography: petroleum ether/EtOAc: 80/20; Yellow solid; Mp: 70 °C; ^{1}H NMR (400 MHz, $CDCl_3$): δ 7.40 (d, J = 8.4 Hz, 2H), 7.23 (d, J = 8.4 Hz, 1.72 H, *Z* isomer), 7.00 (d, J = 8.5 Hz, 0.28H, *E* isomer), 6.17 (t, J = 2.4 Hz, 0.14H, *E* isomer), 5.63 (t, J = 2.1 Hz, 0.86H, *Z* isomer), 4.08 (obs. t, J = 6.4 Hz, 0.28H, *E* isomer), 4.07 (t, J = 6.9 Hz, 1.72H, *Z* isomer), 3.20 (td, J = 6.4 and 2.4 Hz, 0.28H, *E* isomer), 3.01 (td, J = 6.9 and 2.1 Hz, 1.72H, *Z* isomer), 2.98 (s, 0.42H, *E* isomer), 2.74 (s, 2.58H, *Z* isomer); ^{13}C NMR (100 MHz, $CDCl_3$): δ 143.6 (*E* isomer), 140.8 (*Z* isomer), 134.6 (*E* isomer), 133.2 (*Z* isomer), 131.9 (*E* isomer), 131.2 (*Z* isomer), 130.8 (*Z* isomer), 128.1 (*E* isomer), 120.9 (*Z* isomer), 119.6 (*E* isomer), 108.2 (*Z* isomer), 105.6 (*E* isomer), 49.7 (*E* isomer), 49.0 (*Z* isomer), 37.8 (*Z* isomer), 34.9 (*E* isomer), 27.3 (*E* isomer), 26.9 (*Z* isomer); ESIHRMS *m/z* calcd for $C_{11}H_{13}{}^{79}BrNO_2S$ $[M+H]^+$ 301.9845, found 301.9845; *m/z* calcd for $C_{11}H_{13}{}^{81}BrNO_2S$ $[M+H]^+$ 303.9824, found 303.9824.

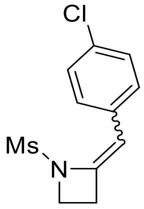

2m

2-(*p*-Chlorobenzylidene)-1-(methanesulfonyl)azetidine 2m. Prepared according to general procedure (200 µmol of iodoethyl-ynamide **1m**, blue LED strips). Yield: 68% (35 mg, 136 µmol). Obtained as a mixture of *Z* and *E* isomers with a *Z/E* ratio of 89/11 in the crude reaction mixture and 96/4 after purification. Solvent system for flash column chromatography: petroleum ether/EtOAc: gradient from 90/10 to 70/30; Pale orange solid; Mp: 83 °C; ^{1}H NMR (400 MHz,

CDCl$_3$): *δ* 7.30 (d, *J* = 8.6 Hz, 2H), 7.25 (d, *J* = 8.6 Hz, 1.92H, *E* + *Z* isomers), 7.06 (d, *J* = 8.6 Hz, 0.08H, *E* isomer), 6.20 (t, *J* = 2.4 Hz, 0.04H, *E* isomer), 5.65 (t, *J* = 2.1 Hz, 0.96H, *Z* isomer), 4.08 (t, *J* = 6.9 Hz, 2H), 3.21 (td, *J* = 6.4 and 2.4 Hz, 0.08H, *E* isomer), 3.02 (td, *J* = 6.9 and 2.1 Hz, 1.92H, *Z* isomer), 2.99 (s, 0.12H, *E* isomer), 2.74 (s, 2.88H, *Z* isomer); ^{13}C NMR (100 MHz, CDCl$_3$), *Z* isomer*: δ 143.4, 134.2, 131.7, 129.0, 127.8, 105.7, 49.7, 35.0, 27.3; ESIHRMS *m/z* calcd for $C_{11}H_{13}{}^{35}ClNO_2S$ [M+H]$^+$ 258.0350, found 258.0347.

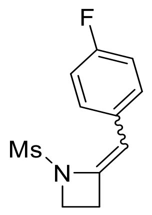

2n

2-(*p*-Fluorobenzylidene)-1-(methanesulfonyl)azetidine 2n. Prepared according to general procedure (200 µmol of iodoethyl-ynamide **1n**, blue LED strips). Yield: 81% (39 mg, 162 µmol). Obtained as a mixture of *Z* and *E* isomers with a *Z/E* ratio of 84/16 in the crude reaction mixture and 87/13 after purification. Solvent system for flash column chromatography: petroleum ether/EtOAc: gradient from 90/10 to 70/30; Pale brown solid; Mp: 75 °C; ^{1}H NMR (400 MHz, CDCl$_3$): *δ* 7.33 (dd, *J* = 8.6 and 5.5 Hz, 1.74H, *Z* isomer), 7.10 (dd, *J* = 8.8 and 5.4 Hz, 0.26H, *E* isomer), 6.98 (t, *J* = 8.7 Hz, 2H), 6.22 (t, *J* = 2.4 Hz, 0.13H, *E* isomer), 5.67 (br. s, 0.87H, *Z* isomer), 4.08 (obs. t, *J* = 6.5 Hz, 0.26H, *E* isomer), 4.07 (t, *J* = 6.9 Hz, 1.74H, *Z* isomer), 3.21 (td, *J* = 6.5 and 2.4 Hz, 0.26H, *E* isomer), 3.01 (td, *J* = 6.9 and 2.1 Hz, 1.74H, *Z* isomer), 2.98 (s, 0.39H, *E* isomer), 2.71 (s, 2.61H, *Z* isomer); ^{13}C NMR (100 MHz, CDCl$_3$), Z isomer†: *δ* 161.9 (d, *J* = 246.4 Hz), 140.3, 130.9 (d, *J* = 8.0 Hz), 130.3 (d, *J* = 3.4 Hz), 115.1 (d, *J* = 21.4 Hz), 108.3, 48.9, 37.8, 26.8; ^{19}F NMR (376 MHz, CDCl$_3$): *δ* -115.2 (*Z* isomer), -116.4 (*E* isomer); ESIHRMS *m/z* calcd for $C_{11}H_{13}FNO_2S$ [M+H]$^+$ 242.0646, found 242.0644.

* Due to the minor amount of the *E* isomer, some signals of this minor isomer could not be observed. For clarity, only the signals corresponding to the major *Z* isomer are reported.
† Due to the minor amount of the *E* isomer, some signals of this minor isomer could not be observed. For clarity, only the signals corresponding to the major *Z* isomer are reported.

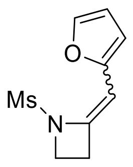

2o

2-(Furan-2-ylmethylene)-1-(methanesulfonyl)azetidine 2o. Prepared according to general procedure (200 µmol of iodoethyl-ynamide **1o**, LCZ-CCP-4V photoreactor and 420 nm tubes). Yield: 61% (26 mg, 122 µmol). Obtained as a mixture of *Z* and *E* isomers in a *Z/E* ratio of 67/33 in the crude reaction mixture and 66/34 after purification. Solvent system for flash column chromatography: petroleum ether/EtOAc: gradient from 90/10 to 70/30; Orange oil; ^{1}H NMR (400 MHz, $CDCl_3$): δ 7.34 (d, *J* = 1.8 Hz, 0.66H, *Z* isomer), 7.31 (d, *J* = 1.8 Hz, 0.34H, *E* isomer), 6.38 (dd, *J* = 3.3 and 1.8 Hz, 0.66H, *Z* isomer), 6.35 (dd, *J* = 3.3 and 1.9 Hz, 0.34H, *E* isomer), 6.29 (d, *J* = 3.3 Hz, 0.66H, *Z* isomer), 6.13 (t, *J* = 2.4 Hz, 0.34H, *E* isomer), 6.05 (d, *J* = 3.3 Hz, 0.34H, *E* isomer), 5.48 (t, *J* = 1.9 Hz, 0.66H, *Z* isomer), 4.13 (t, *J* = 6.7 Hz, 1.32H, *Z* isomer), 4.05 (t, *J* = 6.5 Hz, 0.68H, *E* isomer), 3.23 (td, *J* = 6.5 and 2.4 Hz, 0.68H, *E* isomer), 3.15 (s, 1.98H, *Z* isomer), 2.96 (obs. td, *J* = 6.7 and 2.0 Hz, 1.32H, *Z* isomer), 2.96 (s, 1.02H, *E* isomer); ^{13}C NMR (100 MHz, $CDCl_3$): 151.2 (*E* isomer), 149.2 (*Z* isomer), 142.5 (*E* isomer), 141.3 (*Z* isomer), 140.9 (*Z* isomer), 139.9 (*E* isomer), 111.5 (*Z* isomer), 111.3 (*E* isomer), 108.1 (*Z* isomer), 106.3 (*E* isomer), 96.7 (*Z* isomer), 96.6 (*E* isomer), 49.9 (*E* isomer), 48.9 (*Z* isomer), 40.5 (*Z* isomer), 34.6 (*E* isomer), 27.0 (*E* isomer), 26.2 (*Z* isomer); ESIHRMS *m/z* calcd for $C_9H_{12}NO_3S$ $[M+H]^+$ 214.0532, found 214.0530.

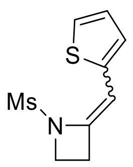

2p

1-(Methanesulfonyl)-2-(thiophen-2-ylmethylene)azetidine 2p. Prepared according to general procedure (200 µmol of iodoethyl-ynamide **1p**, LCZ-CCP-4V photoreactor and 420 nm tubes). Yield: 57% (26 mg, 113 µmol). Obtained as a mixture of *Z* and *E* isomers with a *Z/E* ratio of 64/36 in the crude reaction mixture and 66/34 after purification. Solvent system for flash column chromatography: petroleum ether/EtOAc: gradient from 90/10 to 70/30; Pale brown oil; ^{1}H NMR (400 MHz, $CDCl_3$): δ 7.22 (dd, *J* = 5.2 and 1.1 Hz, 0.66H, *Z* isomer), 7.15 (br. d, *J* =

5.1 Hz, 0.34H, *E* isomer), 7.11 (dt, *J* = 3.5 and 1.0 Hz, 0.66H, *Z* isomer), 6.97 (dd, *J* = 5.2 and 3.6 Hz, 1H), 6.83 (br. d, *J* = 3.5 Hz, 0.34H, *E* isomer), 6.51 (t, *J* = 2.4 Hz, 0.34H, *E* isomer), 5.69 (app. dd, *J* = 3.0 and 1.9 Hz, 0.66H, *Z* isomer), 4.08 (obs. t, *J* = 6.8 Hz, 1.32H, *Z* isomer), 4.06 (obs. t, *J* = 6.8 Hz, 0.68H, *E* isomer), 3.16 (td, *J* = 6.4 and 2.4 Hz, 0.68H, *E* isomer), 2.99 (obs. td, *J* = 6.7 and 2.0 Hz, 1.32H, *Z* isomer), 2.98 (s, 1.02 H, *E* isomer), 2.78 (s, 1.98H, *Z* isomer); ^{13}C NMR (100 MHz, $CDCl_3$): 141.9 (*Z* isomer), 141.8 (*E* isomer), 138.8 (*E* isomer), 136.0 (*Z* isomer), 127.8 (*Z* isomer), 127.3 (*E* isomer), 127.2 (Z isomer), 125.1 (*Z* isomer), 124.6 (*E* isomer), 123.5 (*E* isomer), 101.5 (*E* isomer), 100.8 (*Z* isomer), 49.4 (*E* isomer), 48.7 (*Z* isomer), 39.4 (*Z* isomer), 34.7 (*E* isomer), 26.9 (*E* isomer), 26.7 (*Z* isomer); ESIHRMS *m/z* calcd for $C_9H_{12}NO_2S_2$ $[M+H]^+$ 230.0304, found 230.0301.

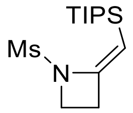

2q

(*Z*)-1-(Methanesulfonyl)-2-(triisopropylsilylmethylene)azetidine 2q. Prepared according to general procedure (200 µmol of iodoethyl-ynamide **1q**, blue LED strips). Yield: 25% (15 mg, 49 µmol). Obtained exclusively as the *Z* isomer. Solvent system for flash column chromatography: petroleum ether/EtOAc: gradient from 90/10 to 50/50; Colorless oil; ^{1}H NMR (400 MHz, $CDCl_3$): δ 4.52 (br. t, *J* = 5.8 Hz, 1H), 3.29 (app. q, *J* = 6.4 Hz, 2H), 2.99 (s, 3H), 2.57 (t, *J* = 6.4 Hz, 2H), 1.08-1.04 (m, 21H); ^{13}C NMR (100 MHz, $CDCl_3$): δ 104.2, 84.0, 42.3, 41.1, 21.8, 18.8, 11.3; ESIHRMS *m/z* calcd for $C_{14}H_{30}NO_2SSi$ $[M+H]^+$ 304.1761, found 304.1761.

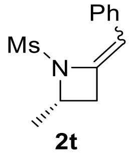

2t

(*S*)-2-Benzylidene-1-(methanesulfonyl)-4-methylazetidine 2t. Prepared according to general procedure (200 µmol of iodoethyl-ynamide **1t**, EvoluChem™ PhotoRedOx Box and a blue Kessil LED lamp). Yield: 72% (34 mg, 143 µmol). Obtained as a mixture of *Z* and *E* isomers with a *Z*/*E* ratio of 72/28 in the crude reaction mixture and 71/29 after purification. Solvent system for

flash column chromatography: petroleum ether/EtOAc: 80/20; Green solid; Mp: 89 °C; $[\alpha]_D^{25}$ - 54 (*c* 0.9, $CHCl_3$); ^1H NMR (400 MHz, $CDCl_3$): *δ* 7.36 (d, *J* = 7.3 Hz, 1.42H, *Z* isomer), 7.30 (t, *J* = 7.5 Hz, 2H), 7.22 (d, *J* = 7.3 Hz, 0.58H, *E* isomer), 7.20-7.12 (m, 1H), 6.24 (t, *J* = 2.4 Hz, 0.29H, *E* isomer), 5.71 (br. s, 0.71H, *Z* isomer), 4.51 (dqd, *J* = 8.3, 6.2 and 4.7 Hz, 0.71H, *Z* isomer), 4.42 (app. sext., *J* = 6.2 Hz, 0.29H, *E* isomer), 3.33 (ddd, *J* = 15.2, 7.5 and 2.3 Hz, 0.29H, *E* isomer), 3.18 (ddd, *J* = 15.0, 8.5 and 2.3 Hz, 0.71H, *Z* isomer), 2.98 (s, 0.87H, *E* isomer), 2.85 (ddd, *J* = 15.4, 4.9 and 2.3 Hz, 0.29H, *E* isomer), 2.65 (s, 2.13H, *Z* isomer), 2.53 (ddd, *J* = 14.9, 4.7 and 1.8 Hz, 0.71H, *Z* isomer), 1.59 (d, *J* = 6.2 Hz, 0.87H, *E* isomer), 1.54 (d, *J* = 6.2 Hz, 2.13H, *Z* isomer); ^{13}C NMR (100 MHz, $CDCl_3$): 140.9 (*E* isomer), 138.2 (*Z* isomer), 135.8 (*E* isomer), 134.6 (*Z* isomer), 129.3 (*Z* isomer), 128.8 (*E* isomer), 128.1 (*Z* isomer), 127.1 (*Z* isomer), 126.6 (*E* isomer), 126.1 (*E* isomer), 109.0 (*Z* isomer), 106.5 (*E* isomer), 59.9 (*E* isomer), 58.4 (*Z* isomer), 38.6 (*Z* isomer), 35.1 (2C, *E* isomer), 33.8 (*Z* isomer), 22.1 (*Z* isomer), 21.6 (*E* isomer); ESIHRMS *m/z* calcd for $C_{12}H_{16}NO_2S$ $[M+H]^+$ 238.0896, found 238.0898.

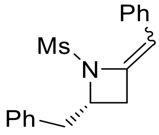

2u

(*S*)-4-Benzyl-2-benzylidene-1-(methanesulfonyl)azetidine 2u. Prepared according to general procedure (200 µmol of iodoethyl-ynamide **1u**, blue LED strips). Yield: 53% (33 mg, 105 µmol). Obtained as a mixture of *Z* and *E* isomers with a *Z/E* ratio of 74/26 in the crude reaction mixture and 75/25 after purification. Solvent system for flash column chromatography: petroleum ether/EtOAc: 90/10; Beige solid; Mp: 189 °C; $[\alpha]_D^{20}$ + 28 (*c* 0.5, $CHCl_3$); ^1H NMR (400 MHz, $CDCl_3$): *δ* 7.37-7.24 (m, 8.50H, *E* + *Z* isomers), 7.23-7.18 (m, 0.75H, *Z* isomer), 7.17-7.09 (m, 0.75H, *Z* isomer), 6.25 (t, *J* = 2.3 Hz, 0.25H, *E* isomer), 5.71 (t, *J* = 1.9 Hz, 0.75H, *Z* isomer), 4.64 (tdd, *J* = 8.5, 5.2 and 4.5 Hz, 0.75H, *Z* isomer), 4.48 (dddd, *J* = 9.2, 7.4, 5.0 and 4.0 Hz, 0.25H, *E* isomer), 3.41 (dd, *J* = 13.7 and 4.0 Hz, 0.25 H, *E* isomer), 3.26 (dd, *J* = 13.6 and 5.2 Hz, 0.75H, *Z* isomer), 3.16 (ddd, *J* = 15.4, 7.4 and 2.3 Hz, 0.25H, *E* isomer), 3.13-3.04 (m, 1.75H, *E* + *Z* isomers), 3.01 (s, 0.75H, *E* isomer), 2.98 (ddd, *J* = 15.5, 5.1 and 2.4 Hz, 0.25H, *E* isomer), 2.65 (ddd, *J* = 15.1, 4.5 and 1.8 Hz, 0.75H, *Z* isomer), 2.56 (s, 2.25H, *Z* isomer); ^{13}C NMR

(100 MHz, $CDCl_3$): δ 140.6 (*E* isomer), 138.0 (*Z* isomer), 136.5 (*Z* isomer), 135.8 (*E* isomer), 135.7 (*E* isomer), 134.8 (*Z* isomer), 129.7 (*Z* isomer), 129.5 (*E* isomer), 129.2 (*Z* isomer), 128.9 (*E* isomer), 128.8 (*E* isomer), 128.7 (*Z* isomer), 128.1 (*Z* isomer), 127.2 (*E* isomer), 127.1 (*Z* isomer), 127.1 (*Z* isomer), 126.6 (*E* isomer), 126.2 (*E* isomer), 109.9 (*Z* isomer), 107.0 (*E* isomer), 63.5 (*E* isomer), 62.1 (*Z* isomer), 41.8 (*Z* isomer), 41.3 (*E* isomer), 38.9 (*Z* isomer), 34.9 (*E* isomer), 33.0 (*E* isomer), 32.1 (*Z* isomer); ESIHRMS *m/z* calcd for $C_{18}H_{20}NO_2S$ $[M+H]^+$ 314.1209, found 314.1218.

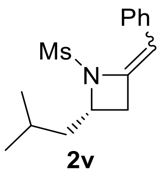

2v

(*S*)-2-Benzylidene-4-isobutyl-1-(methanesulfonyl)azetidine 2v. Prepared according to general procedure (1.0 mmol of iodoethyl-ynamide **1v**, LCZ-CCP-4V photoreactor and 420 nm tubes). Yield: 86% (239 mg, 855 µmol). Obtained as a mixture of *Z* and *E* isomers with a *Z*/*E* ratio of 68/32 in the crude reaction mixture and 70/30 after purification. Solvent system for flash column chromatography: petroleum ether/EtOAc: 80/20; Orange oil; $[\alpha]_D^{20}$ - 30 (*c* 1.0, $CHCl_3$); ^1H NMR (400 MHz, $CDCl_3$): δ 7.36 (app. d, *J* = 8.0 Hz, 1.40H, *Z* isomer), 7.29 (app. t, *J* = 7.5 Hz, 2H), 7.23-7.19 (m, 0.60H, *E* isomer), 7.15 (app. d, *J* = 8.0 Hz, 1H), 6.25 (t, *J* = 2.4 Hz, 0.30H, *E* isomer), 5.73 (t, *J* = 2.2 Hz, 0.70H, *Z* isomer), 4.45 (tdd, *J* = 8.4, 5.5 and 4.5 Hz, 0.70H, *Z* isomer), 4.32 (ddt, *J* = 9.3, 7.4 and 4.6 Hz, 0.30H, *E* isomer), 3.32 (ddd, *J* = 15.3, 7.5 and 2.4 Hz, 0.30H, *E* isomer), 3.21 (ddd, *J* = 14.9, 8.5 and 2.4 Hz, 0.70H, *Z* isomer), 2.99 (s, 0.90H, *E* isomer), 2.90 (ddd, *J* = 15.3, 4.9 and 2.4 Hz, 0.30H, *E* isomer), 2.67 (s, 2.10H, *Z* isomer), 2.55 (ddd, *J* = 14.9, 4.5 and 1.8 Hz, 0.70H, *Z* isomer), 2.07-2.00 (m, 0.30H, *E* isomer), 1.92 (ddd, *J* = 13.1, 6.3 and 5.4 Hz, 0.70H, *Z* isomer), 1.78-1.62 (m, 2H), 0.98-0.94 (m, 6H); ^{13}C NMR (100 MHz, $CDCl_3$): δ 141.3 (*E* isomer), 138.7 (*Z* isomer), 135.9 (*E* isomer), 134.8 (*Z* isomer), 129.3 (*Z* isomer), 128.8 (*E* isomer), 128.1 (*Z* isomer), 127.1 (*Z* isomer), 126.7 (*E* isomer), 126.2 (*E* isomer), 109.9 (*Z* isomer), 107.0 (*E* isomer), 62.9 (*E* isomer), 61.0 (*Z* isomer), 45.1 (*Z* isomer), 44.4 (*E* isomer), 38.8 (*Z* isomer), 34.9 (*E* isomer), 34.4 (*E* isomer), 33.3 (*Z* isomer), 25.3 (*Z* isomer), 25.2 (*E* isomer), 23.1 (*E* isomer), 22.9 (*Z* isomer), 22.9 (*Z* isomer), 22.7 (*E* isomer); ESIHRMS *m/z* calcd for $C_{15}H_{22}NO_2S$ $[M+H]^+$ 280.1366, found 280.1364.

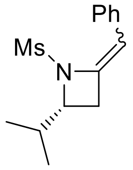

2w

(*R*)-2-Benzylidene-4-isopropyl-1-(methanesulfonyl)azetidine 2w. Prepared according to general procedure (200 µmol of iodoethyl-ynamide **1w**, blue LED strips). Yield: 75% (40 mg, 151 µmol). Obtained as a mixture of *Z* and *E* isomers with a *Z*/*E* ratio of 83/17 both in the crude reaction mixture and after purification. Solvent system for flash column chromatography: petroleum ether/EtOAc: 80/20; Yellow oil; $[\alpha]_D^{20}$ - 14 (*c* 2.1, $CHCl_3$); ^{1}H NMR (400 MHz, $CDCl_3$): δ 7.37 (app. d, *J* = 8.2 Hz, 1.66H, *Z* isomer), 7.30 (app. t, *J* = 7.3 Hz, 2H), 7.23-7.15 (m, 1.34H, *E* + *Z* isomers), 6.29 (t, *J* = 2.4 Hz, 0.17H, *E* isomer), 5.76 (br. t, *J* = 2.1 Hz, 0.83H, *Z* isomer), 4.24 (ddd, *J* = 8.9, 6.5 and 4.2 Hz, 0.83H, *Z* isomer), 4.17 (app. dt, *J* = 7.9 and 5.2 Hz, 0.17H, *E* isomer), 3.16 (ddd, *J* = 15.0, 8.9 and 2.6 Hz, 1H), 2.98 (s, 0.51H, *E* isomer), 2.96 (obs. ddd, *J* = 15.5, 5.0 and 2.3 Hz, 0.17H, *E* isomer), 2.69 (s, 2.49H, *Z* isomer), 2.61 (ddd, *J* = 15.1, 4.2 and 1.7 Hz, 0.83H, *Z* isomer), 2.20 (app. oct., *J* = 6.7 Hz, 0.17H, *E* isomer), 2.09 (app. oct., *J* = 6.7 Hz, 0.83H, *Z* isomer), 1.07-0.98 (m, 6H); ^{13}C NMR (100 MHz, $CDCl_3$): δ 141.1 (*E* isomer), 138.7 (*Z* isomer), 135.8 (*E* isomer), 134.9 (*Z* isomer), 129.1 (*Z* isomer), 128.8 (*E* isomer), 128.1 (*Z* isomer), 127.1 (*Z* isomer), 126.7 (*E* isomer), 126.2 (*E* isomer), 110.6 (*Z* isomer), 107.6 (*E* isomer), 68.3 (*E* isomer), 66.8 (*Z* isomer), 38.5 (*Z* isomer), 34.3 (*E* isomer), 32.3 (*Z* isomer), 31.2 (*E* isomer), 30.1 (*Z* isomer), 29.4 (*E* isomer), 18.3 (*Z* isomer), 18.0 (*E* isomer), 16.6 (*Z* isomer), 15.7 (*E* isomer); ESIHRMS *m/z* calcd for $C_{14}H_{20}NO_2S$ $[M+H]^+$ 266.1209, found 266.1216.

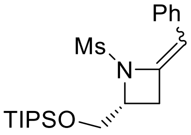

2x

(*R*)-2-Benzylidene-1-(methanesulfonyl)-4-[(triisopropylsilyloxy)methyl]azetidine 2x. Prepared according to general procedure (73 µmol of iodoethyl-ynamide **1x**, LCZ-CCP-4V photoreactor and 420 nm tubes, 120 hours). Yield: 77% (23 mg, 56 µmol). Obtained as a mixture of *Z* and *E* isomers with a *Z*/*E* ratio of 81/19 both in the crude reaction mixture and after purification. Solvent system for flash column chromatography: petroleum ether/EtOAc:

80/20; Orange oil; $[\alpha]_D^{25}$ + 21 (*c* 0.9, $CHCl_3$); ^{1}H NMR (400 MHz, $CDCl_3$): δ 7.35 (app. d, *J* = 8.2 Hz, 1.62H, *Z* isomer), 7.32-7.27 (m, 2H), 7.23-7.14 (m, 1.38H, *E* + *Z* isomers), 6.23 (t, *J* = 2.4 Hz, 0.19H, *E* isomer), 5.72 (br. t, *J* = 2.2 Hz, 0.81H, *Z* isomer), 4.50 (app. dq, *J* = 8.6 and 4.4 Hz, 0.81H, *Z* isomer), 4.39 (app. dtd, *J* = 7.2, 5.1 and 3.4 Hz, 0.19H, *E* isomer), 4.09 (A of ABX syst., *J* = 11.0 and 5.2 Hz, 0.19H, *E* isomer), 4.03 (A of ABX syst., *J* = 11.0 and 4.4 Hz, 0.81H, *Z* isomer), 3.99 (B of ABX syst., *J* = 11.0 and 3.4 Hz, 0.19H, *E* isomer), 3.92 (B of ABX syst., *J* = 11.0 and 4.0 Hz, 0.81H, *Z* isomer), 3.26 (ddd, *J* = 15.3, 5.0 and 2.4 Hz, 0.19H, *E* isomer), 3.20 (ddd, *J* = 15.1, 7.1 and 2.3 Hz, 0.19H, *E* isomer), 3.10 (ddd, *J* = 14.8, 8.7 and 2.4 Hz, 0.81H, *Z* isomer), 3.02 (s, 0.57H, *E* isomer), 2.89 (ddd, *J* = 14.7, 4.5 and 1.8 Hz, 0.81H, *Z* isomer), 2.68 (s, 2.43H, *Z* isomer), 1.23-1.04 (m, 21H); ^{13}C NMR (100 MHz, $CDCl_3$): δ 141.4 (*E* isomer), 138.7 (*Z* isomer), 135.9 (*E* isomer), 135.0 (*Z* isomer), 129.3 (*Z* isomer), 128.8 (*E* isomer), 128.1 (*Z* isomer), 127.0 (*Z* isomer), 126.7 (*E* isomer), 126.1 (*E* isomer), 108.9 (*Z* isomer), 106.5 (*E* isomer), 64.6 (*Z* isomer), 64.2 (*E* isomer), 63.8 (*E* isomer), 62.2 (*Z* isomer), 39.2 (*Z* isomer), 35.5 (*E* isomer), 30.3 (*E* isomer), 29.0 (*Z* isomer), 18.1 (*Z* isomer), 18.1 (*E* isomer), 12.1 (*Z* isomer), 12.1 (*E* isomer); ESIHRMS *m/z* calcd for $C_{21}H_{36}NO_3SSi$ $[M+H]^+$ 410.2180, found 410.2180.

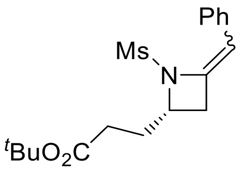

2y

(*S*)-2-Benzylidene-4-(2-*tert*-butoxycarbonylethyl)-1-(methanesulfonyl)azetidine 2y. Prepared according to general procedure (200 µmol of iodoethyl-ynamide **1y**, blue LED strips). Yield: 71% (50 mg, 142 µmol). Obtained as a mixture of *Z* and *E* isomers with a *Z*/*E* ratio of 85/15 in the crude reaction mixture and 87/13 after purification. Solvent system for flash column chromatography: petroleum ether/EtOAc: 85/15; Orange oil; $[\alpha]_D^{25}$ - 43 (*c* 1.5, $CHCl_3$); ^{1}H NMR (400 MHz, $CDCl_3$): δ 7.38-7.34 (m, 1.74H, *Z* isomer), 7.32-7.27 (m, 2H, *E* + *Z* isomers), 7.23-7.19 (m, 0.87H, *Z* isomer), 7.19-7.12 (m, 0.39H, *E* isomer), 6.28 (t, *J* = 2.4 Hz, 0.13H, *E* isomer), 5.76 (t, *J* = 2.1 Hz, 0.87H, *Z* isomer), 4.45 (dtd, *J* = 8.7, 6.5 and 4.4 Hz, 0.87H, *Z* isomer), 4.38 (app. tt, *J* = 7.4 and 5.3 Hz, 0.13H, *E* isomer), 3.32 (ddd, *J* = 15.5, 7.7 and 2.5 Hz, 0.13H, *E* isomer), 3.22 (ddd, *J* = 15.1, 8.7 and 2.4 Hz, 0.87H, *Z* isomer), 2.97 (s, 0.39H, *E* isomer), 2.92 (ddd, *J* = 15.5, 4.8 and 2.3 Hz, 0.13H, *E* isomer), 2.67 (s, 2.61H, *Z* isomer), 2.58 (ddd, *J* = 15.1,

4.5 andd 1.8 Hz, 0.87H, *Z* isomer), 2.46-2.37 (m, 2H, *E* + *Z* isomers), 2.20-2.04 (m, 2H, *E* + *Z* isomers), 1.46 (s, 7.83H, *Z* isomer), 1.45 (s, 1.17H, *E* isomer); ^{13}C NMR (100 MHz, $CDCl_3$): *δ* 172.3 (*Z* isomer), 172.2 (*E* isomer), 140.7 (*E* isomer), 138.2 (*Z* isomer), 135.7 (*E* isomer), 134.5 (*Z* isomer), 129.3 (*Z* isomer), 128.8 (*E* isomer), 128.1 (*Z* isomer), 127.3 (*Z* isomer), 126.7 (*E* isomer), 126.3 (*E* isomer), 110.6 (*Z* isomer), 107.7 (*E* isomer), 80.8 (*E* isomer), 80.7 (*Z* isomer), 62.7 (*E* isomer), 61.1 (*Z* isomer), 38.4 (*Z* isomer), 34.5 (*E* isomer), 33.3 (*E* isomer), 32.5 (*Z* isomer), 31.2 (*Z* isomer), 30.9 (*Z* isomer), 30.8 (*E* isomer), 30.6 (*E* isomer), 28.2 (*Z* + *E* isomer); ESIHRMS *m/z* calcd for $C_{18}H_{26}NO_4S$ $[M+H]^+$ 352.1577, found 352.1579.

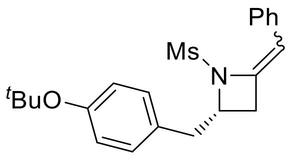

2z

(*S*)-2-Benzylidene-4-[4-(*tert*-butoxy)benzyl]-1-(methanesulfonyl)azetidine 2z. Prepared according to general procedure (200 µmol of iodoethyl-ynamide **1z**, blue LED strips). Yield: 52% (40 mg, 104 µmol). Obtained as a mixture of *Z* and *E* isomers in a *Z/E* ratio of 83/17 in the crude reaction mixture and 89/11 after purification. Solvent system for flash column chromatography: pentane/EtOAc: gradient from 90/10 to 80/20; Yellow oil; $[\alpha]_D^{20}$ + 41 (*c* 0.7, $CHCl_3$); ^{1}H NMR (400 MHz, $CDCl_3$): *δ* 7.34-7.27 (m, 3.78H, *E* + *Z* isomers), 7.22-7.10 (m, 1.22H, *E* + *Z* isomers), 7.16 (obs. d, *J* = 8.4 Hz, 2H), 6.96 (d, *J* = 8.4 Hz, 2H), 6.25 (t, *J* = 2.4 Hz, 0.11H, *E* isomer), 5.70 (br. t, *J* = 1.9 Hz, 0.89H, *Z* isomer), 4.62 (app. tdd, *J* = 8.2, 5.4 and 4.4 Hz, 0.89H, *Z* isomer), 4.44 (dddd, *J* = 9.1, 7.5, 4.8 and 4.0 Hz, 0.11H, *E* isomer), 3.35 (dd, *J* = 13.7 and 4.0 Hz, 0.11H, *E* isomer), 3.18 (dd, *J* = 13.7 and 5.4 Hz, 0.89H, *Z* isomer), 3.11 (ddd, *J* = 15.0, 8.5 and 2.4 Hz, 1H), 3.04 (dd, *J* = 13.7 and 8.1 Hz, 1H), 3.00 (s, 0.33H, *E* isomer), 2.97 (ddd, *J* = 15.5, 5.0 and 2.4 Hz, 0.11H, *E* isomer), 2.63 (ddd, *J* = 15.0, 4.4 and 1.8 Hz, 0.89H, *Z* isomer), 2.55 (s, 2.67H, *Z* isomer), 1.34 (s, 0.99H, *E* isomer), 1.33 (s, 8.01H, *Z* isomer); ^{13}C NMR (100 MHz, $CDCl_3$), *Z* isomer*: *δ* 154.5, 138.0, 134.8, 131.4, 130.1, 129.1, 128.1, 127.1, 124.4, 110.0, 78.6, 62.2, 41.1, 38.9, 32.2, 29.0; ESIHRMS *m/z* calcd for $C_{22}H_{28}NO_3S$ $[M+H]^+$ 386.1784, found 386.1785.

* Due to the minor amount of the *E* isomer, some signals of this minor isomer could not be observed. For clarity, only the signals corresponding to the major *Z* isomer are reported.

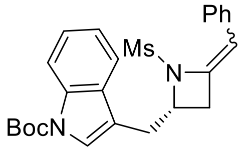

2aa

(*S*)-2-Benzylidene-4-[(*N*-*tert*-butoxycarbonyl-1*H*-indol-3-yl)methyl]-1-(methanesulfonyl) azetidine 2aa. Prepared according to general procedure (200 µmol of iodoethyl-ynamide **1aa**, blue LEDs strips). Yield: 68% (61 mg, 135 µmol). Obtained as a mixture of *Z* and *E* isomers in a *Z*/*E* ratio of 83/17 in the crude reaction mixture and 84/16 after purification. Solvent system for flash column chromatography: pentane/Et_2O: 70/30; Yellow oil; $[\alpha]_D^{20}$ - 31 (*c* 0.7, $CHCl_3$); ^{1}H NMR (400 MHz, $CDCl_3$): δ 8.15 (d, *J* = 7.6 Hz, 1H), 7.65 (d, *J* = 7.4 Hz, 0.84H, *Z* isomer), 7.62 (d, *J* = 7.5 Hz, 0.16H, *E* isomer), 7.52 (s, 0.84H, *Z* isomer), 7.50 (s, 0.16H, *E* isomer), 7.39-7.26 (m, 5.68H, *E* + *Z* isomers), 7.25-7.19 (m, 0.84H, *Z* isomer), 7.18-7.11 (m, 0.48H, *E* isomer), 6.28 (t, *J* = 2.4 Hz, 0.16H, *E* isomer), 5.73 (br. t, *J* = 2.1 Hz, 0.84H, *Z* isomer), 4.72 (app. tt, *J* = 8.8 and 4.4 Hz, 0.84H, *Z* isomer), 4.57 (dddd, *J* = 9.9, 7.4, 4.9 and 3.7 Hz, 0.16H, *E* isomer), 3.53 (ddd, *J* = 14.6, 3.7 and 1.2 Hz, 0.16H, *E* isomer), 3.40 (ddd, *J* = 14.5, 4.2 and 1.1 Hz, 0.84H, *Z* isomer), 3.26-3.13 (m, 1.16H, *E* + *Z* isomers), 3.10 (ddd, *J* = 15.1, 8.5 and 2.4 Hz, 0.84H, *Z* isomer), 3.04 (s, 0.48H, *E* isomer), 2.99 (ddd, *J* = 15.3, 4.9 and 2.4 Hz, 0.16H, *E* isomer), 2.67 (obs. ddd, *J* = 15.1, 4.6 and 1.9 Hz, 0.84H, *Z* isomer), 2.64 (s, 2.52H, *Z* isomer), 1.70 (obs. s, 1.44H, *E* isomer), 1.68 (s, 7.56H, *Z* isomer); ^{13}C NMR (100 MHz, $CDCl_3$): δ 149.8 (*E* + *Z* isomers), 140.5 (*E* isomer), 138.1 (*Z* isomer), 135.6 (*E* isomer), 135.5 (*E* + *Z* isomers), 134.5 (*Z* isomer), 130.7 (*Z* isomer), 130.5 (*E* isomer), 129.3 (*Z* isomer), 128.8 (*E* isomer), 128.1 (*Z* isomer), 127.2 (*Z* isomer), 126.6 (*E* isomer), 126.2 (*E* isomer), 124.8 (*E* isomer), 124.7 (*Z* isomer), 123.8 (*Z* isomer), 123.7 (*E* isomer), 122.9 (*E* isomer), 122.8 (Z isomer), 119.3 (*Z* isomer), 119.0 (*E* isomer), 115.5 (*E* isomer), 115.4 (*Z* isomer), 115.2 (*Z* isomer), 114.8 (*E* isomer), 109.8 (*Z* isomer), 107.2 (*E* isomer), 84.0 (*E* isomer), 83.9 (*Z* isomer), 62.2 (*E* isomer), 60.7 (*Z* isomer), 38.6 (*Z* isomer), 34.8 (*E* isomer), 33.6 (*E* isomer), 32.2 (*Z* isomer), 31.2 (*Z* isomer), 31.0 (*E* isomer), 28.3 (*E* + *Z* isomers); ESIHRMS *m/z* calcd for $C_{25}H_{32}N_3O_4S$ $[M+NH_4]^+$ 470.2108, found 470.2107.

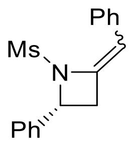

2ab

(*R*)-2-Benzylidene-1-(methanesulfonyl)-4-phenylazetidine 2ab. Prepared according to general procedure (200 µmol of iodoethyl-ynamide **1ab**, blue LEDs strips). Yield: 43% (26 mg, 87 µmol). Obtained as a mixture of *Z* and *E* isomers in a *Z*/*E* ratio of 87/13 in the crude reaction mixture and 88/12 after purification. Solvent system for flash column chromatography: petroleum ether/EtOAc: 85/15; Yellow oil; $[\alpha]_D^{20}$ + 141 (*c* 1.0, $CHCl_3$); ^{1}H NMR (400 MHz, $CDCl_3$): δ 7.54-7.49 (m, 2H), 7.45-7.39 (m, 4H), 7.38-7.30 (m, 2.76H, *E* + *Z* isomers), 7.26-7.21 (obs. m, 0.88H, *Z* isomer), 7.20-7.15 (m, 0.36H, *E* isomer), 6.40 (t, *J* = 2.4 Hz, 0.12H, *E* isomer), 5.82 (br. t, *J* = 2.2 Hz, 0.88H, *Z* isomer), 5.49 (dd, *J* = 9.1 and 4.6 Hz, 0.88H, *Z* isomer), 5.34 (dd, *J* = 7.9 and 4.9 Hz, 0.12H, *E* isomer), 3.66 (ddd, *J* = 15.4, 8.0 and 2.4 Hz, 0.12H, *E* isomer), 3.56 (ddd, *J* = 15.0, 9.1 and 2.4 Hz, 0.88H, *Z* isomer), 3.21 (ddd, *J* = 15.4, 4.9 and 2.3 Hz, 0.12H, *E* isomer), 2.92 (s, 0.36H, *E* isomer), 2.87 (ddd, *J* = 15.0, 4.6 and 1.8 Hz, 0.88H, *Z* isomer), 2.66 (s, 2.64H, *Z* isomer); ^{13}C NMR (100 MHz, $CDCl_3$): δ 140.8 (*E* isomer), 140.1 (*Z* isomer), 138.8 (*E* isomer), 138.3 (*Z* isomer), 135.8 (*E* isomer), 135.1 (*Z* isomer), 129.2 (*Z* isomer), 129.0 (*E* isomer), 129.0 (*Z* isomer), 128.9 (*E* isomer), 128.8 (*E* isomer), 128.5 (*Z* isomer), 128.2 (*Z* isomer), 127.2 (*Z* isomer), 126.7 (*E* isomer), 126.6 (*E* isomer), 126.3 (*Z* isomer), 126.2 (*E* isomer), 109.3 (*Z* isomer), 107.1 (*E* isomer), 64.8 (*E* isomer), 62.9 (*Z* isomer), 39.9 (*Z* isomer), 37.1 (*E* isomer), 36.6 (*E* isomer), 35.8 (*Z* isomer); ESIHRMS *m/z* calcd for $C_{17}H_{18}NO_2S$ $[M+H]^+$ 300.1053, found 300.1038.

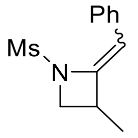

2ac

2-Benzylidene-1-(methanesulfonyl)-3-methylazetidine 2ac. Prepared according to general procedure (200 µmol of iodoethyl-ynamide **1ac**, EvoluChem™ PhotoRedOx Box and a blue Kessil LED lamp). Yield: 51% (24 mg, 101 µmol). Obtained as a mixture of *Z* and *E* isomers in a *Z*/*E* ratio of 75/25 in the crude reaction mixture and 74/26 after purification. Solvent system

for flash column chromatography: petroleum ether/EtOAc: 80/20; Brown oil; ^{1}H NMR (400 MHz, $CDCl_3$): δ 7.37 (app. d, *J* = 8.0 Hz, 1.48H, *Z* isomer), 7.30 (app. t, *J* = 7.3 Hz, 2H), 7.23-7.13 (m, 1.52H, *E* + *Z* isomers), 6.22 (d, *J* = 2.1 Hz, 0.26H, *E* isomer), 5.71 (d, *J* = 1.8 Hz, 0.74H, *Z* isomer), 4.18 (app. t, *J* = 7.9 Hz, 0.74H, *Z* isomer), 4.18 (obs. app. t, *J* = 7.1 Hz, 0.26H, *E* isomer), 3.69 (dd, *J* = 7.8 and 5.1 Hz, 1H), 3.68-3.62 (obs. m, 0.26H, *E* isomer), 3.38-3.28 (m, 0.74H, *Z* isomer), 2.98 (s, 0.78H, *E* isomer), 2.68 (s, 2.22H, *Z* isomer), 1.40 (d, *J* = 6.7 Hz, 0.78H, *E* isomer), 1.36 (d, *J* = 7.0 Hz, 2.22H, *Z* isomer); ^{13}C NMR (100 MHz, $CDCl_3$): δ 147.8 (*E* isomer), 146.4 (*Z* isomer), 135.0 (*E* isomer), 134.4 (*Z* isomer), 129.3 (*Z* isomer), 128.7 (*E* isomer), 128.1 (*Z* isomer), 127.1 (*Z* isomer), 127.1 (*E* isomer), 126.2 (*E* isomer), 107.6 (*Z* isomer), 106.6 (*E* isomer), 56.5 (*Z* isomer), 56.4 (*E* isomer), 38.3 (*Z* isomer), 34.6 (*E* isomer), 34.4 (*E* isomer), 34.3 (*Z* isomer), 18.1 (*Z* isomer), 16.4 (*E* isomer); ESIHRMS *m/z* calcd for $C_{12}H_{16}NO_2S$ $[M+H]^+$ 238.0896, found 238.0893.

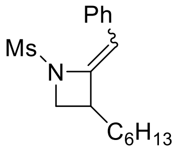

2ad

2-Benzylidene-3-hexyl-1-(methanesulfonyl)azetidine 2ad. Prepared according to general procedure (150 µmol of iodoethyl-ynamide **1ad**, EvoluChem™ PhotoRedOx Box and a blue Kessil LED lamp). Yield: 71% (33 mg, 107 µmol). Obtained as a mixture of *Z* and *E* isomers in a *Z*/*E* ratio of 72/28 in the crude reaction mixture and 73/27 after purification. Solvent system for flash column chromatography: petroleum ether/EtOAc: 90/10; Yellow oil; ^{1}H NMR (400 MHz, $CDCl_3$): δ 7.36 (app. d, *J* = 8.1 Hz, 1.46H, *Z* isomer), 7.32-7.27 (m, 2H), 7.23-7.20 (m, 0.54H, *E* isomer), 7.19-7.13 (m, 1H), 6.22 (d, *J* = 2.3 Hz, 0.27H, *E* isomer), 5.73 (d, *J* = 1.9 Hz, 0.73H, *Z* isomer), 4.14 (app. t, *J* = 7.8 Hz, 0.73H, *Z* isomer), 4.11 (obs. app. t, *J* = 7.2 Hz, 0.27H, *E* isomer), 3.73 (dd, *J* = 7.8 and 5.0 Hz, 0.73H, *Z* isomer), 3.71 (obs. dd, *J* = 7.2 and 3.9 Hz, 0.27H, *E* isomer), 3.57 (ddt, *J* = 13.8, 6.1 and 3.8 Hz, 0.27H, *E* isomer), 3.27-3.17 (m, 0.73H, *Z* isomer), 2.97 (s, 0.81H, *E* isomer), 2.66 (s, 2.19H, *Z* isomer), 1.97-1.86 (m, 0.27H, *E* isomer), 1.85-1.73 (m, 0.73H, *Z* isomer), 1.72-1.54 (m, 1H), 1.42-1.22 (m, 8H), 0.90 (obs. t, *J* = 6.6 Hz, 2.19H, Z isomer), 0.85 (obs. t, *J* = 7.0 Hz, 0.81H, *E* isomer); ^{13}C NMR (100 MHz, $CDCl_3$): δ 146.7 (*E* isomer), 145.4 (*Z* isomer), 135.2 (*E* isomer), 134.5 (*Z* isomer), 129.3 (*Z* isomer), 128.7

(*E* isomer), 128.1 (*Z* isomer), 127.1 (*E* + *Z* isomer), 126.2 (*E* isomer), 107.8 (*Z* isomer), 106.7 (*E* isomer), 55.0 (*Z* isomer), 54.8 (*E* isomer), 39.7 (*E* isomer), 39.4 (*Z* isomer), 38.4 (*Z* isomer), 34.5 (*E* isomer), 33.1 (*Z* isomer), 31.8 (*Z* isomer), 31.7 (*E* isomer), 30.0 (*E* isomer), 29.2 (*Z* isomer), 29.0 (*E* isomer), 26.6 (*Z* isomer), 26.4 (*E* isomer), 22.7 (*Z* isomer), 22.6 (*E* isomer), 14.2 (*Z* isomer), 14.2 (*E* isomer); ESIHRMS *m/z* calcd for $C_{17}H_{26}NO_2S$ $[M+H]^+$ 308.1679, found 308.1687.

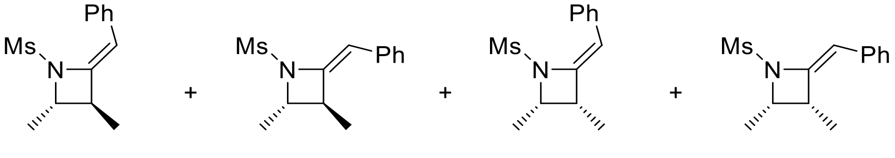

(*Z*)-2af*trans* **(*E*)-2af*trans*** **(*Z*)-2af*cis*** **(*E*)-2af*cis***

(3*S*,4*S*)-2-[(*Z*)-Benzylidene]-3,4-dimethyl-1-(methanesulfonyl)azetidine (*Z*)-2af*trans*, (3*S*,4*S*)-2-[(*E*)-benzylidene]-3,4-dimethyl-1-(methanesulfonyl)azetidine (*E*)-2af*trans*, (3*R*,4*S*)-2-[(*Z*)-benzylidene]-3,4-dimethyl-1-(methanesulfonyl)azetidine (*Z*)-2af*cis* and **(3*R*,4*S*)-2-[(*E*)-benzylidene]-3,4-dimethyl-1-(methanesulfonyl)azetidine (*E*)-2af*cis***. Prepared according to general procedure (159 µmol of iodoethyl-ynamide **1af** or 177 µmol of iodoethyl-ynamide **1af'**, EvoluChem™ PhotoRedOx Box and a blue Kessil LED lamp). Yields: 70% (28 mg, 111 µmol) from **1af** and 63% (28 mg, 111 µmol) from **1af'**. Obtained as hardly separable mixtures of 4 diastereomers with a $Z_{trans}/E_{trans}/Z_{cis}/E_{cis}$ ratio of 60/22/13/5 from **1af** and 58/24/13/5 from **1af'** in the crude reaction mixtures.*

- **(3*S*,4*S*)-2-[(*Z*)-Benzylidene]-3,4-dimethyl-1-(methanesulfonyl)azetidine (*Z*)-2af*trans*** (fastest eluting isomer). Solvent system for flash column chromatography: petroleum ether/EtOAc: 85/15; Orange oil; $[\alpha]_D^{25}$ - 26 (*c* 1.3, $CHCl_3$); ^1H NMR (400 MHz, $CDCl_3$): δ 7.36 (app. d, *J* = 7.6 Hz, 2H), 7.30 (app. t, *J* = 7.4 Hz, 2H), 7.21 (app. t, *J* = 7.1 Hz, 1H), 5.67 (s, 1H), 4.05 (app. quint., *J* = 5.7 Hz, 1H), 2.70 (app. quint., *J* = 6.7 Hz, 1H), 2.64 (s, 3H), 1.51 (d, *J* = 6.2 Hz, 3H), 1.32 (d, *J* = 7.0 Hz, 3H); ^{13}C NMR (100 MHz, $CDCl_3$): δ 144.5, 134.5, 129.5, 128.1, 127.1, 106.3, 67.0, 40.8, 38.8, 21.3, 17.1; ESIHRMS *m/z* calcd for $C_{13}H_{18}NO_2S$ $[M+H]^+$ 252.1053, found 252.1052.

* Isolated yields for each diastereomer are not given as they were only isolated in small amounts for characterization purposes.

- **(3*S*,4*S*)-2-[(*E*)-Benzylidene]-3,4-dimethyl-1-(methanesulfonyl)azetidine (*E*)-2af$_{trans}$** (second-fastest eluting isomer). Solvent system for flash column chromatography: petroleum ether/EtOAc: 85/15; Orange oil; $[\alpha]_D^{25}$ - 41 (*c* 0.6, $CHCl_3$); ^{1}H NMR (400 MHz, $CDCl_3$): δ 7.28 (obs. t, *J* = 7.6 Hz, 2H), 7.20-7.12 (m, 3H), 6.23 (d, *J* = 2.2 Hz, 1H), 3.93 (qd, *J* = 6.2 and 4.0 Hz, 1H), 3.19 (qdd, *J* = 6.7, 4.0 and 2.1 Hz, 1H), 3.01 (s, 3H), 1.57 (d, *J* = 6.2 Hz, 3H), 1.28 (d, *J* = 7.0 Hz, 3H); ^{13}C NMR (100 MHz, $CDCl_3$): δ 146.1, 135.1, 128.7, 127.3, 126.2, 106.3, 67.2, 42.5, 35.8, 20.5, 15.4; ESIHRMS *m/z* calcd for $C_{13}H_{18}NO_2S$ $[M+H]^+$ 252.1053, found 252.1052.

- **(3*R*,4*S*)-2-[(*Z*)-Benzylidene]-3,4-dimethyl-1-(methanesulfonyl)azetidine (*Z*)-2af$_{cis}$** and **(3*R*,4*S*)-2-[(*E*)-benzylidene]-3,4-dimethyl-1-(methanesulfonyl)azetidine (*E*)-2af$_{cis}$** (inseparable third and fourth isomers). Solvent system for flash column chromatography: petroleum ether/EtOAc: gradient from 85/15 to 80/20; Orange oil; $[\alpha]_D^{25}$ - 56 (*c* 0.9, $CHCl_3$); ^{1}H NMR (400 MHz, $CDCl_3$): δ 7.38 (app. d, *J* = 8.4 Hz, 1H), 7.32-7.27 (m, 2H), 7.22-7.13 (m, 2H), 6.20 (d, *J* = 2.3 Hz, 0.50H, *E* isomer), 5.71 (d, *J* = 2.3 Hz, 0.50H, *Z* isomer), 4.57 (dq, *J* = 9.0 and 6.6 Hz, 0.50H, *Z* isomer), 4.47 (dq, *J* = 7.8 and 6.5 Hz, 0.50H, *E* isomer), 3.69 (app. quint.d, *J* = 7.3 and 2.4 Hz, 0.50H, *E* isomer), 3.51 (dqd, *J* = 9.5, 7.2 and 2.4 Hz, 0.50H, *Z* isomer), 2.98 (s, 1.50H, *E* isomer), 2.69 (s, 1.50H, *Z* isomer), 1.45 (d, *J* = 6.5 Hz, 1.50H, *Z* isomer), 1.43 (d, *J* = 6.5 Hz, 1.50H, *E* isomer), 1.30 (d, *J* = 7.3 Hz, 1.50H, *E* isomer), 1.24 (d, *J* = 7.2 Hz, 1.50H, *Z* isomer); ^{13}C NMR (100 MHz, $CDCl_3$): δ 146.9 (*E* isomer), 145.5 (*Z* isomer), 135.3 (*E* isomer), 134.6 (*Z* isomer), 129.2 (*E* or *Z* isomer), 128.7 (*E* or *Z* isomer), 128.1 (*E* or *Z* isomer), 127.1 (*E* + *Z* isomers), 126.1 (*E* or *Z* isomer), 108.5 (*E* isomer), 106.3 (*Z* isomer), 61.9 (*E* isomer), 61.8 (*Z* isomer), 38.5 (*Z* isomer), 37.7 (*Z* isomer), 37.5 (*E* isomer), 34.3 (*E* isomer), 16.2 (*Z* isomer), 16.0 (*E* isomer), 13.1 (*Z* isomer), 10.9 (*E* isomer); ESIHRMS *m/z* calcd for $C_{13}H_{18}NO_2S$ $[M+H]^+$ 252.1053, found 252.1072.

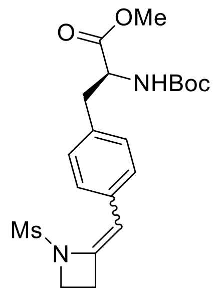

2ag

(*S*)-2-{4-{2-[(*tert*-Butoxycarbonyl)amino]-2-methoxycarbonylethyl}benzylidene}-1-(methanesulfonyl)azetidine 2ag. Prepared according to general procedure (200 µmol of iodoethyl-ynamide **1ag**, blue LED strips). Yield: 63% (53 mg, 125 µmol). Obtained as a mixture of *Z* and *E* isomers in a *Z*/*E* ratio of 86/14 in the crude reaction mixture and 94/6 after purification. Solvent system for flash column chromatography: petroleum ether/EtOAc: 70/30; Yellow oil; $[\alpha]_D^{20}$ + 0.083 (*c* 1.2, $CHCl_3$); ^1H NMR (400 MHz, $CDCl_3$): δ 7.27 (d, *J* = 8.0 Hz, 1.88H, *Z* isomer), 7.04 (app. d, *J* = 8.0 Hz, 2.12H, *E* + *Z* isomers), 6.19 (t, *J* = 2.3 Hz, 0.06H, *E* isomer), 5.67 (br. t, *J* = 2.1 Hz, 0.94H, *Z* isomer), 4.98 (d, *J* = 7.7 Hz, 1H), 4.53 (app. q, *J* = 6.6 Hz, 1H), 4.04 (t, *J* = 6.8 Hz, 2H), 3.70 (s, 0.18H, *E* isomer), 3.67 (s, 2.82H, *Z* isomer), 3.20 (td, *J* = 6.5 and 2.4 Hz, 0.12H, *E* isomer), 3.10-2.96 (obs. m, 2H), 2.99 (td, *J* = 6.9 and 2.1 Hz, 1.88H, *Z* isomer), 2.95 (s, 0.18H, *E* isomer), 2.63 (s, 2.82H, *Z* isomer), 1.40 (s, 9H); ^{13}C NMR (100 MHz, $CDCl_3$): δ 172.3 (*E* + *Z* isomers), 155.2 (*E* + *Z* isomers), 142.9 (*E* isomer), 140.4 (*Z* isomer), 134.9 (*Z* isomer), 134.4 (*E* isomer), 133.9 (*E* isomer), 133.1 (*Z* isomer), 129.7 (*E* isomer), 129.4 (*Z* isomer), 129.0 (*Z* isomer), 126.7 (*E* isomer), 108.7 (*Z* isomer), 106.5 (*E* isomer), 80.1 (*E* + *Z* isomers), 54.5 (*E* + *Z* isomers), 52.3 (*E* isomer), 52.3 (*Z* isomer), 49.7 (*E* isomer), 48.6 (*Z* isomer), 38.2 (*E* isomer + 2C for *Z* isomer), 34.4 (*E* isomer), 28.4 (*E* + *Z* isomers), 27.3 (*E* isomer), 26.7 (*Z* isomer); ESIHRMS *m/z* calcd for $C_{20}H_{28}N_2O_6SNa$ $[M+Na]^+$ 447.1560, found 447.1564.

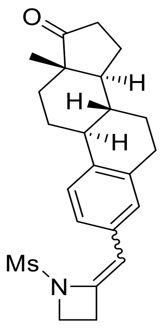

2ah

(8*R*,9*S*,13*S*,14*S*)-[2-(13-Methyl-17-oxo-7,8,9,11,12,13,14,15,16,17-decahydro-6*H*-cyclopenta[*a*]phenanthren-3-ylmethylene)]-1-(methanesulfonyl)azetidine 2ah. Prepared according to general procedure (200 µmol of iodoethyl-ynamide **1ah**, blue LED strips, filtered over Celite® using dichloromethane as solvent). Yield: 59% (47 mg, 118 µmol). Obtained as a mixture of *Z* and *E* isomers in a *Z*/*E* ratio of 82/18 in the crude reaction mixture and 89/11 after purification. Solvent system for flash column chromatography: etroleum ether/EtOAc: 60/40; Off-white solid; Mp: 219 °C; $[\alpha]_D^{20}$ + 102 (*c* 0.5, $CHCl_3$); ^1H NMR (400 MHz, $CDCl_3$): *δ* 7.23 (obs. d, *J* = 8.9 Hz, 0.11H, *E* isomer), 7.21 (d, *J* = 8.7 Hz, 0.89H, *Z* isomer), 7.16-7.11 (m, 1.78H, *Z* isomer), 6.95 (app. dd, *J* = 8.2 and 1.4 Hz, 0.11H, *E* isomer), 6.89 (s, 0.11H, *E* isomer), 6.20 (t, *J* = 2.4 Hz, 0.11H, *E* isomer), 5.66 (br. t, *J* = 2.1 Hz, 0.89H, *Z* isomer), 4.07 (t, *J* = 7.0 Hz, 2H), 3.23 (td, *J* = 6.5 and 2.2 Hz, 0.22H, *E* isomer), 3.01 (td, *J* = 7.1 and 2.0 Hz, 1.78H, *Z* isomer), 2.96 (s, 0.33H, *E* isomer), 2.88 (dd, *J* = 8.8 and 4.0 Hz, 2H), 2.74 (s, 2.67H, *Z* isomer), 2.50 (dd, *J* = 19.0 and 9.0 Hz, 1H), 2.43-2.36 (m, 1H), 2.32-2.23 (m, 1H), 2.19-2.10 (m, 1H), 2.09-1.91 (m, 3H), 1.68-1.36 (m, 6H), 0.90 (s, 3H); ^{13}C NMR (100 MHz, $CDCl_3$): *δ* 221.0 (*Z* isomer), 220.9 (*E* isomer), 142.5 (*E* isomer), 139.7 (*Z* isomer), 138.6 (*Z* isomer), 138.0 (*E* isomer), 136.9 (*E* isomer), 136.1 (*Z* isomer), 133.1 (*E* isomer), 131.6 (*Z* isomer), 129.7 (*Z* isomer), 127.5 (*E* isomer), 126.3 (*Z* isomer), 125.8 (*E* isomer), 125.0 (*Z* isomer), 123.9 (*E* isomer), 109.4 (*Z* isomer), 106.7 (*E* isomer), 50.6 (*Z* isomer), 50.6 (*E* isomer), 49.7 (*E* isomer), 48.9 (*Z* isomer), 48.1 (*E* + *Z* isomers), 44.5 (*Z* isomer), 44.4 (*E* isomer), 38.3 (*E* isomer), 38.2 (*Z* isomer), 37.9 (*Z* isomer), 35.9 (*E* + *Z* isomers), 34.1 (*E* isomer), 31.7 (*E* + *Z* isomer), 29.5 (*E* isomer), 29.3 (*Z* isomer), 27.3 (*E* isomer), 27.0 (*Z* isomer), 26.6 (*Z* isomer), 26.6 (*E* isomer), 25.8 (*E* isomer), 25.7 (*Z* isomer), 21.7 (*E* + *Z* isomers), 14.0 (*Z* isomer), 13.9 (*E* isomer); ESIHRMS *m/z* calcd for $C_{23}H_{30}NO_3S$ $[M+H]^+$ 400.1941, found 400.1949.

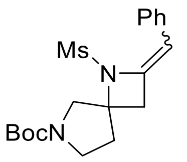

2ai

2-Benzylidene-6-(*tert*-butoxycarbonyl)-1-(methanesulfonyl)-1,6-diazaspiro[3.4]octane 2ai. Prepared according to general procedure (200 µmol of iodoethyl-ynamide **1ai**, blue LED strips). Yield: 66% (50 mg, 132 µmol). Obtained as a mixture of *Z* and *E* isomers in a *Z*/*E* ratio of 91/9 in the crude reaction mixture and 94/6 after purification. Solvent system for flash column chromatography: pentane/Et_2O: 50/50; White solid; Mp: 132 °C; ^1H NMR (400 MHz, $CDCl_3$): δ 7.36-7.27 (m, 3.88H, *E* + *Z* isomers), 7.25-7.20 (m, 0.94H, *Z* isomer), 7.17-7.10 (m, 0.18H, *E* isomer), 6.22 (t, *J* = 2.2 Hz, 0.06H, *E* isomer), 5.67 (br. s, 0.94H, *Z* isomer), 3.95-3.81 (m, 1H), 3.68-3.54 (m, 2H), 3.30 (ddd, *J* = 10.8, 9.4 and 7.1 Hz, 1H), 3.25 (obs. d, *J* = 14.7 Hz, 0.06H, *E* isomer), 3.14 (d, *J* = 14.3 Hz, 0.06H, *E* isomer), 3.07 (s, 0.18H, *E* isomer), 2.94 (dd, *J* = 14.3 and 1.7 Hz, 0.94H, *Z* isomer), 2.90-2.81 (m, 0.94H, *Z* isomer), 2.80-2.67 (m, 1H), 2.58 (s, 2.82, *Z* isomer), 2.15-2.00 (m, 1H), 1.46 (s, 9H); ^{13}C NMR (100 MHz, $CDCl_3$), *Z* isomer*: δ 154.5, 136.2, 134.3 and 134.3 (rotamers), 129.8, 128.0, 127.3, 105.7 and 105.6 (rotamers), 79.9 and 79.8 (rotamers), 73.2 and 73.1 (rotamers), 54.1 and 53.4 (rotamers), 43.8 and 43.0 (rotamers), 41.6 and 41.5 (rotamers), 41.1 and 40.5 (rotamers), 35.5 and 33.7 (rotamers), 28.6; ESIHRMS *m/z* calcd for $C_{19}H_{26}N_2O_4SNa$ $[M+Na]^+$ 401.1505, found 401.1504.

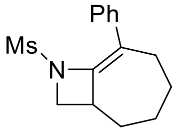

2aj

8-(Methanesulfonyl)-6-phenyl-8-azabicyclo[5.2.0]non-6-ene 2aj. Prepared according to general procedure (200 µmol of iodoethyl-ynamide **1aj**, EvoluChem™ PhotoRedOx Box and a blue Kessil LED lamp, 16 hours). Yield: 39% (22 mg, 79 µmol). Solvent system for flash column chromatography: petroleum ether/EtOAc: 85/15; Off-white solid; Mp: 71 °C; ^1H NMR (400 MHz, $CDCl_3$): δ 7.35-7.27 (m, 4H), 7.25-7.20 (m, 1H), 4.16 (t, *J* = 8.2 Hz, 1H), 3.65 (dd, *J* = 8.0

---

* Due to the minor amount of the *E* isomer, some signals of this minor isomer could not be observed. For clarity, only the signals corresponding to the major *Z* isomer are reported.

and 5.4 Hz, 1H), 3.41-3.31 (m, 1H), 2.62-2.53 (m, 1H), 2.37 (s, 3H), 2.30 (app. dd, *J* = 15.6 and 7.1 Hz, 1H), 2.11-2.02 (m, 1H), 1.94-1.83 (m, 2H), 1.68-1.47 (m, 3H); ^{13}C NMR (100 MHz, $CDCl_3$): *δ* 142.9, 140.7, 128.8, 128.4, 127.1, 123.3, 53.6, 40.5, 39.3, 36.1, 31.8, 30.6, 28.4; ESIHRMS *m/z* calcd for $C_{15}H_{20}NO_2S$ $[M+H]^+$ 278.1209, found 278.1210.

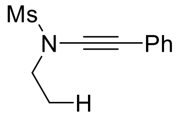

4a

***N*-Ethyl-*N*-(methanesulfonyl)phenylethynylamine 4a**. Obtained as a byproduct in some reactions conducted during the optimization. Solvent system for flash column chromatography: petroleum ether/EtOAc: 85/15; Yellow oil; ^1H NMR (400 MHz, $CDCl_3$): *δ* 3.47 (q, *J* = 7.2 Hz, 2H), 3.04 (s, 3H), 2.31 (t, *J* = 7.5 Hz, 2H), 1.31 (t, *J* = 7.2 Hz, 3H), 1.16 (t, *J* = 7.2 Hz, 3H); ^{13}C NMR (100 MHz, $CDCl_3$): *δ* 131.6, 128.4, 128.1, 122.7, 81.4, 71.3, 47.1, 38.6, 13.9; ESIHRMS *m/z* calcd for $C_{11}H_{14}NO_2S$ $[M+H]^+$ 224.0740, found 224.0748.

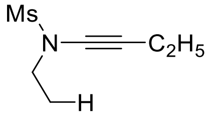

4r

***N*-Ethyl-*N*-(methanesulfonyl)but-1-yn-1-ylamine 4r**. Obtained according to general procedure (200 µmol of iodoethyl-ynamide **1r**, blue LEDs strips). Yield: 91% (32 mg, 182 µmol). Solvent system for flash column chromatography: pentane/Et_2O: 80/20; Yellow oil; ^1H NMR (400 MHz, $CDCl_3$): *δ* 7.44-7.39 (m, 2H), 7.34-7.28 (m, 3H), 3.63 (q, *J* = 7.2 Hz, 2H), 3.13 (s, 3H), 1.41 (t, *J* = 7.2 Hz, 3H); ^{13}C NMR (100 MHz, $CDCl_3$): *δ* 72.3, 71.8, 46.8, 37.9, 14.4, 13.7, 12.4; ESIHRMS *m/z* calcd for $C_7H_{14}NO_2S$ $[M+H]^+$ 176.0740, found 176.0736.

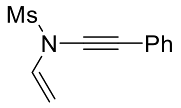

5a

***N*-(Methanesulfonyl)-*N*-vinylphenylethynylamine 5a**. Obtained as a byproduct in some reactions conducted during the optimization. Solvent system for flash column chromatography: petroleum ether/EtOAc: 85/15; Yellow oil; ^{1}H NMR (400 MHz, $CDCl_3$): *δ* 7.51-7.46 (m, 2H), 7.37-7.32 (m, 3H), 6.75 (dd, *J* = 15.0 and 8.2 Hz, 1H), 5.11 (dd, *J* = 15.0 and 1.6 Hz, 1H), 4.77 (dd, *J* = 8.2 and 1.6 Hz, 1H), 3.17 (s, 3H); ^{13}C NMR (100 MHz, $CDCl_3$): *δ* 131.9, 130.4, 128.8, 128.6, 121.9, 98.3, 77.6, 76.5, 38.7; ESIHRMS *m/z* calcd for $C_{11}H_{15}N_2O_2S$ $[M+NH_4]^+$ 239.0549, found 239.0853.

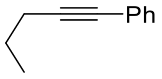

9

Pent-1-yn-1-ylbenzene 9. Obtained according to general procedure (200 µmol of (5-iodopent-1-yn-1-yl)benzene, blue LED strips). Yield: 47% (14 mg, 94 µmol). Solvent system for flash column chromatography: pentane/Et_2O: 90/10; Colorless oil. This compound has already been reported.[5]

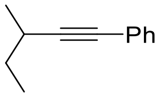

10

(3-Methylpent-1-yn-1-yl)benzene 10. Obtained according to general procedure (200 µmol of (5-iodo-3,3-dimethylpent-1-yn-1-yl)benzene, blue LED strips). Yield: 95% (30 mg, 190 µmol). Solvent system for flash column chromatography: pentane/Et_2O: 90/10; Colorless oil. This compound has already been reported.[6]

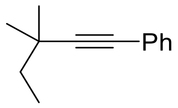

11

(3,3-Dimethylpent-1-yn-1-yl)benzene 11. Obtained according to general procedure (200 µmol of (5-iodo-3,3-dimethylpent-1-yn-1-yl)benzene, blue LED strips). Yield: 96% (33 mg, 191 µmol). Solvent system for flash column chromatography: petroleum ether/EtOAc: gradient from 100/0 to 90/10; Colorless oil. This compound has already been reported.[7]

2.10. Experimental Procedures and Characterization Data: Hydrogenation of Azetidines

General procedure

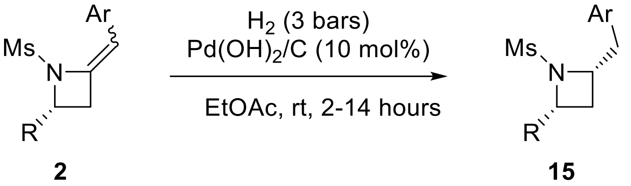

A glass vial was charged with the azetidine **2** (1.0 equiv.), palladium hydroxide on carbon 20% wt. (0.1 molar equiv.) and ethyl acetate (0.01-0.02 M). The vial was placed in an autoclave, purged with N_2, pressurized with H_2 (3 bars) and stirred at rt for the appropriate amount of time (2-14 hours). After the reactor was depressurized and purged with N_2, the reaction mixture was filtered over a plug of Celite® (washed with ethyl acetate) and concentrated under reduced pressure. The crude residue was finally purified by flash column chromatography over silica gel to afford the desired product.

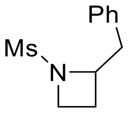

15a

2-Benzyl-1-(methanesulfonyl)azetidine 15a. Prepared according to general procedure (45 µmol of azetidine **2a**, 0.01 M in ethyl acetate, 2 hours). Yield: 98% (10 mg, 44 µmol). Solvent system for flash column chromatography: petroleum ether/EtOAc: 50/50; White solid; Mp: 82 °C; ^{1}H NMR (400 MHz, $CDCl_3$): δ 7.34-7.28 (m, 2H), 7.26-7.19 (m, 3H), 4.52 (app. qd, J = 8.2 and 4.8 Hz, 1H), 3.90 (app. q, J = 8.8 Hz, 1H), 3.64 (ddd, J = 8.8, 7.7 and 4.1 Hz, 1H), 3.14 (A of ABX syst., J = 13.7 and 4.8 Hz, 1H), 2.99 (B of ABX syst., J = 13.7 and 8.5 Hz, 1H), 2.78 (s, 3H), 2.20-2.11 (m, 1H), 2.10-2.02 (m, 1H); ^{13}C NMR (100 MHz, $CDCl_3$): δ 136.5, 129.6, 128.7, 126.9, 63.7, 47.1, 42.6, 37.1, 21.6; ESIHRMS m/z calcd for $C_{11}H_{16}NO_2S$ $[M+H]^+$ 226.0896, found 226.0895.

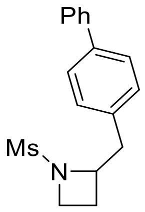

15b

1-(Methanesulfonyl)-2-(*p*-phenylbenzyl)azetidine 15b. Prepared according to general procedure (134 µmol of azetidine **2g**, 0.02 M in ethyl acetate, 9 hours). Yield: 74% (30 mg, 100 µmol). Solvent system for flash column chromatography: petroleum ether/EtOAc: gradient from 80/20 to 70/30; Beige solid; Mp: 134 °C; ^{1}H NMR (400 MHz, $CDCl_3$): δ 7.60-7.56 (m, 2H), 7.55 (d, J = 8.2 Hz, 2H), 7.44 (app. t, J = 7.3 Hz, 2H), 7.37-7.32 (m, 1H), 7.29 (d, J = 8.2 Hz, 2H), 4.55 (app. qd, J = 8.2 and 4.7 Hz, 1H), 3.92 (app. q, J = 8.2 Hz, 1H), 3.66 (ddd, J = 8.8, 7.7 and 4.1 Hz, 1H), 3.18 (A of ABX syst., J = 13.7 and 4.6 Hz, 1H), 3.04 (B of ABX syst., J = 13.7 and 8.6 Hz, 1H), 2.82 (s, 3H), 2.23-2.06 (m, 2H); ^{13}C NMR (100 MHz, $CDCl_3$): δ 140.9, 139.9, 135.5, 130.0, 128.9, 127.4, 127.4, 127.1, 63.6, 47.2, 42.1, 37.1, 21.6; ESIHRMS *m/z* calcd for $C_{17}H_{20}NO_2S$ $[M+H]^+$ 302.1209, found 302.1209.

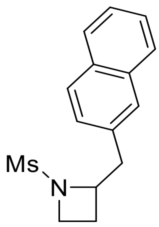

15c

1-(Methanesulfonyl)-2-(naphthalen-2-ylmethyl)azetidine 15c. Prepared according to general procedure (95 µmol of azetidine **2h**, 0.02 M in ethyl acetate, 6 hours). Yield: 77% (20 mg, 73 µmol). Solvent system for flash column chromatography: petroleum ether/EtOAc: 70/30; White solid; Mp: 94 °C; ^{1}H NMR (400 MHz, $CDCl_3$): δ 7.84-7.78 (m, 3H), 7.66 (br. s, 1H), 7.50-7.43 (m, 2H), 7.36 (dd, J = 8.4 and 1.7 Hz, 1H), 4.61 (app. qd, J = 8.1 and 4.6 Hz, 1H), 3.91 (app. q, J = 8.2 Hz, 1H), 3.64 (app. td, J = 7.8 and 5.0 Hz, 1H), 3.31 (A of ABX syst., J = 13.7 and 4.6 Hz, 1H), 3.15 (B of ABX syst., J = 13.7 and 8.6 Hz, 1H), 2.81 (s, 3H), 2.18-2.09 (m, 2H); ^{13}C NMR (100 MHz, $CDCl_3$): δ 134.0, 133.6, 132.5, 128.3, 128.1, 127.9, 127.8, 127.7, 126.3,

125.8, 63.6, 47.2, 42.7, 37.1, 21.6; ESIHRMS *m/z* calcd for $C_{15}H_{18}NO_2S$ $[M+H]^+$ 276.1053, found 276.1049.

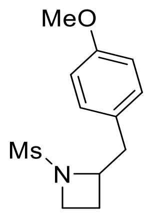

15d

2-(*p*-Methoxybenzyl)-1-(methanesulfonyl)azetidine 15d. Prepared according to general procedure (136 µmol of azetidine **2j**, 0.02 M in ethyl acetate, 14 hours). Yield: 60% (21 mg, 82 µmol). Solvent system for flash column chromatography: petroleum ether/EtOAc: 70/30; Off-white solid; Mp: 91 °C; ^{1}H NMR (400 MHz, $CDCl_3$): δ 7.13 (d, *J* = 8.6 Hz, 2H), 6.84 (d, *J* = 8.7 Hz, 2H), 4.47 (app. qd, *J* = 8.1 and 4.8 Hz, 1H), 3.89 (app. q, *J* = 8.4 Hz, 1H), 3.79 (s, 3H), 3.62 (ddd, *J* = 9.0, 7.7 and 4.1 Hz, 1H), 3.06 (A of ABX syst., *J* = 13.8 and 4.8 Hz, 1H), 2.93 (B of ABX syst., *J* = 13.8 and 8.4 Hz, 1H), 2.78 (s, 3H), 2.19-1.99 (m, 2H); ^{13}C NMR (100 MHz, $CDCl_3$): δ 158.6, 130.6, 128.5, 114.1, 63.9, 55.4, 47.1, 41.6, 37.1, 21.5; ESIHRMS *m/z* calcd for $C_{12}H_{18}NO_3S$ $[M+H]^+$ 256.1002, found 256.1010.

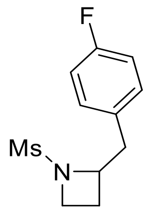

15e

2-(*p*-Fluorobenzyl)-1-(methanesulfonyl)azetidine 15e. Prepared according to general procedure (97 µmol of azetidine **2n**, 0.02 M in ethyl acetate, 3 hours). Yield: 98% (23 mg, 95 µmol). Obtained without purification; White solid; Mp: 97 °C; ^{1}H NMR (600 MHz, $CDCl_3$): δ 7.17 (app. dd, *J* = 8.5 and 5.4 Hz, 2H), 6.99 (app. t, *J* = 8.7 Hz, 2H), 4.48 (app. qd, *J* = 8.1 and 4.6 Hz, 1H), 3.88 (app. q, *J* = 8.5 Hz, 1H), 3.61 (ddd, *J* = 9.0, 7.6 and 3.9 Hz, 1H), 3.08 (A of ABX syst., *J* = 13.9 and 4.6 Hz, 1H), 2.97 (B of ABX syst., *J* = 13.9 and 8.3 Hz, 1H), 2.80 (s, 3H), 2.16-2.10 (m, 1H), 2.07-2.00 (m, 1H); ^{13}C NMR (150 MHz, $CDCl_3$): δ 162.0 (d, *J* = 245.0 Hz), 132.1 (d, *J* =

3.3 Hz), 131.1 (d, J = 7.9 Hz), 115.5 (d, J = 21.2 Hz), 63.4, 47.2, 41.5, 36.9, 21.3; ^{19}F NMR (376 MHz, $CDCl_3$): δ -116.7; ESIHRMS m/z calcd for $C_{11}H_{15}FNO_2S$ $[M+H]^+$ 244.0802, found 244.0804.

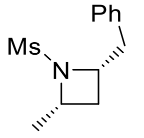

15f

(2*R*,4*S*)-2-Benzyl-4-methyl-1-(methanesulfonyl)azetidine 15f. Prepared according to general procedure (122 µmol of azetidine **2t**, 0.02 M in ethyl acetate, 14 hours). Yield: 89% (26 mg, 109 µmol). Obtained as a mixture of *cis* and *trans* isomers in a *cis*/*trans* ratio of 69/31 in the crude reaction mixture. Solvent system for flash column chromatography: petroleum ether/EtOAc: 80/20; Colorless oil; $[\alpha]_D^{25}$ - 21 (c 1.0, $CHCl_3$); ^{1}H NMR (400 MHz, $CDCl_3$), *cis* isomer:* δ 7.33-7.27 (m, 2H), 7.26-7.19 (m, 3H), 4.31 (app. qd, J = 8.1 and 4.7 Hz, 1H), 4.15 (tq, J = 8.0 and 6.2 Hz, 1H), 3.10 (A of ABX syst., J = 13.7 and 4.7 Hz, 1H), 2.95 (B of ABX syst., J = 13.7 and 8.2 Hz, 1H), 2.80 (s, 3H), 2.31 (app. dt, J = 11.0 and 8.3 Hz, 1H), 1.64 (app. dt, J = 11.0 and 7.8 Hz, 1H), 1.28 (d, J = 6.2 Hz, 3H); ^{13}C NMR (100 MHz, $CDCl_3$), *cis* isomer: δ 136.6, 129.7, 128.6, 126.9, 60.2, 56.2, 42.9, 38.1, 29.8, 22.8; ESIHRMS m/z calcd for $C_{12}H_{18}NO_2S$ $[M+H]^+$ 240.1053, found 240.1064.

The cis configuration of the major isomer has been attributed on the basis of NOESY experiments (see page 356 for full NOESY spectrum).

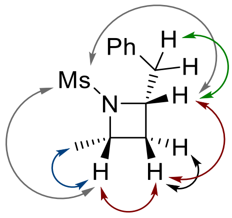

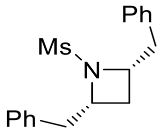

15g

***cis*-2,4-Dibenzyl-1-(methanesulfonyl)azetidine 15g**. Prepared according to general procedure (64 µmol of azetidine **2u**, 0.02 M in ethyl acetate, 14 hours). Yield: 98% (20 mg, 63 µmol).

* A small fraction of the *cis* isomer could be separated from the mixture of *cis* and *trans* isomers and was used for characterization.

Obtained without purification as a mixture of *cis* and *trans* isomers with a *cis*/*trans* ratio of 87/13; White solid; Mp: 130 °C; ^{1}H NMR (400 MHz, $CDCl_3$): *δ* 7.34-7.21 (m, 6H), 7.17-7.13 (m, 4H), 4.46 (ddt, *J* = 10.7, 6.8 and 3.4 Hz, 0.26H, *trans* isomer), 4.33 (tdd, *J* = 8.3, 7.4 and 4.9 Hz, 1.74H, *cis* isomer), 3.46 (A of ABX syst., *J* = 13.1 and 4.0 Hz, 0.26H, *trans* isomer), 2.99 (s, 0.39H, *trans* isomer), 2.95 (A of ABX syst., *J* = 13.7 and 4.9 Hz, 1.74H, *cis* isomer), 2.93 (obs. B of ABX syst., *J* = 10.1 and 2.7 Hz, 0.26H, *trans* isomer), 2.77 (B of ABX syst., *J* = 13.7 and 8.1 Hz, 1.74H, *cis* isomer), 2.66 (s, 2.61H, *cis* isomer), 2.21 (app. dt, *J* = 11.4 and 8.5 Hz, 1H), 1.75 (dt, *J* = 11.3 and 7.4 Hz, 1H); ^{13}C NMR (100 MHz, $CDCl_3$): *δ* 136.6 (*cis* isomer), 136.4 (*trans* isomer), 129.8 (*cis* isomer), 129.4 (*trans* isomer), 128.7 (*trans* isomer), 128.6 (*cis* isomer), 126.9 (*cis* + *trans* isomers), 62.6 (*trans* isomer), 60.0 (*cis* isomer), 42.5 (*cis* isomer), 41.5 (*trans* isomer), 40.6 (*trans* isomer), 38.2 (*cis* isomer), 27.6 (*trans* isomer), 27.0 (*cis* isomer); ESIHRMS *m/z* calcd for $C_{18}H_{22}NO_2S$ $[M+H]^+$ 316.1366, found 316.1372.

*The cis configuration of the major isomer has been attributed by analogy with the configuration of the major isomers of compounds **15f**, **15h** and **15i**.*

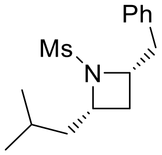

15h

(2*R*,4*S*)-2-Benzyl-4-isobutyl-1-(methanesulfonyl)azetidine 15h. Prepared according to general procedure (300 µmol of azetidine **2v**, 0.02 M in ethyl acetate, 7 hours). Yield: 81% (68 mg, 242 µmol). Obtained as a single isomer. Solvent system for flash column chromatography: petroleum ether/EtOAc: 80/20; White solid; Mp: 103 °C; $[\alpha]_D^{20}$ - 9 (*c* 1.0, $CHCl_3$); ^{1}H NMR (400 MHz, $CDCl_3$): *δ* 7.33-7.28 (m, 2H), 7.26-7.18 (m, 3H), 4.32 (app. qd, *J* = 8.2 and 4.7 Hz, 1H), 4.09 (dtd, *J* = 9.6, 7.9 and 4.5 Hz, 1H), 3.11 (dd, *J* = 13.7 and 4.8 Hz, 1H), 2.93 (dd, *J* = 13.7 and 8.5 Hz, 1H), 2.79 (s, 3H), 2.32 (dt, *J* = 11.0 and 8.3 Hz, 1H), 1.74-1.55 (m, 3H), 1.39 (ddd, *J* = 13.1, 9.6 and 6.4 Hz, 1H), 0.86 (d, *J* = 6.6 Hz, 3H), 0.85 (d, *J* = 6.6 Hz, 3H); ^{13}C NMR (100 MHz, $CDCl_3$): *δ* 136.7, 129.7, 128.6, 126.9, 60.5, 59.2, 46.2, 43.0, 38.4, 29.1, 24.9, 23.1, 22.6; ESIHRMS *m/z* calcd for $C_{15}H_{24}NO_2S$ $[M+H]^+$ 282.1522, found 282.1526.

The cis configuration of the single isomer obtained has been attributed on the basis of NOESY experiments (see page 357 for full NOESY spectrum).

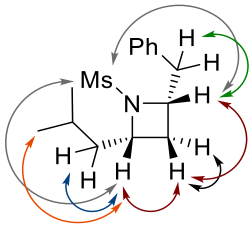

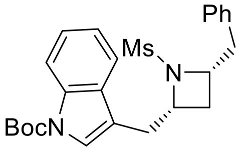

15i

(2*R*,4*S*)-2-Benzyl-4-[1-(*N*-*tert*-butoxycarbonyl-1*H*-indol-3-yl)methyl]-1-(methanesulfonyl) azetidine 15i. Prepared according to general procedure (80 µmol of azetidine **2aa**, 0.02 M in ethyl acetate, 7 hours). Yield: 77% (28 mg, 62 µmol). Obtained as a mixture of *cis* and *trans* isomers in a *cis*/*trans* ratio of 80/20 in the crude reaction mixture and 82/18 after purification. Solvent system for flash column chromatography: petroleum ether/EtOAc: 80/20; Yellow oil; $[\alpha]_D^{20}$ + 18 (*c* 1.4, $CHCl_3$); ^1H NMR (400 MHz, $CDCl_3$): *δ* 8.10 (d, *J* = 7.7 Hz, 1H), 7.54 (d, *J* = 7.7 Hz, 0.18H, *trans* isomer), 7.49 (d, *J* = 7.7 Hz, 0.82H, *cis* isomer), 7.37-7.21 (m, 6H), 7.18-7.12 (m, 2H), 4.61-4.49 (m, 0.36H, *trans* isomer), 4.44-4.31 (m, 1.64H, *cis* isomer), 3.57 (ddd, *J* = 14.1, 3.7 and 1.2 Hz, 0.18H, *trans* isomer), 3.47 (dd, *J* = 13.1 and 4.0 Hz, 0.18H, *trans* isomer), 3.13 (ddd, *J* = 14.6, 4.1 and 1.1 Hz, 0.82H, *cis* isomer), 3.04 (s, 0.54H, *trans* isomer), 2.95 (A of ABX syst., *J* = 13.8 and 5.4 Hz, 1H), 2.90 (B of ABX syst., *J* = 13.7 and 7.5 Hz, 1H), 2.79 (dd, *J* = 14.7 and 9.2, 0.82H, *cis* isomer), 2.75 (s, 2.46H, *cis* isomer), 2.26 (app. dt, *J* = 11.4 and 8.5 Hz, 0.82H, *cis* isomer), 2.08-2.02 (m, 0.18H, *trans* isomer), 2.00-1.93 (m, 0.18H, *trans* isomer), 1.77 (app. dt, *J* = 11.4 and 7.5 Hz, 0.82H, *cis* isomer), 1.68 (s, 7.38H, *cis* isomer), 1.66 (s, 1.62H, *trans* isomer); ^{13}C NMR (100 MHz, $CDCl_3$): *δ* 149.8 (*cis* + *trans* isomer), 136.5 (*cis* isomer), 136.3 (*trans* isomer), 135.5 (*cis* + *trans* isomer), 130.8 (*cis* isomer), 130.5 (*trans* isomer), 129.9 (*cis* isomer), 129.4 (*trans* isomer), 128.8 (*trans* isomer), 128.6 (*cis* isomer), 127.0 (*cis* + *trans* isomers), 124.7 (*trans* isomer), 124.7 (*cis* isomer), 123.7 (*cis* isomer), 123.6 (*trans* isomer), 122.8 (*trans* isomer), 122.7 (*cis* isomer), 119.3 (*cis* isomer), 119.0 (*trans* isomer), 115.5 (*cis* isomer) 115.4 (*trans* isomer), 115.5 (*cis* isomer), 115.3 (*trans* isomer), 83.9 (*cis* + *trans* isomers), 62.7 (*trans* isomer), 61.2 (*trans* isomer), 60.3 (*cis* isomer), 58.7 (*cis* isomer), 42.5 (*cis* isomer), 41.6 (*trans* isomer), 40.6 (*trans* isomer), 38.1 (*cis* isomer), 32.0 (*cis* isomer), 30.2

(*trans* isomer), 28.5 (*cis* isomer), 28.4 (*cis* + *trans* isomers), 27.6 (*cis* isomer); ESIHRMS *m/z* calcd for $C_{25}H_{30}N_2O_4SNa$ $[M+Na]^+$ 477.1818, found 477.1822.

The cis configuration of the major isomer has been attributed on the basis of NOESY experiments (see page 358 for full NOESY spectrum).

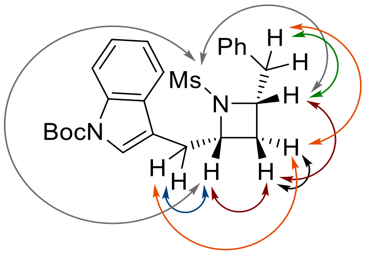

2.11. Experimental Procedures and Characterization Data: Additional Diversification of Azetidines

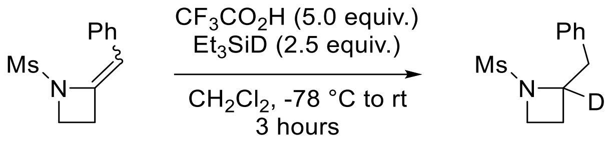

2a **15a$_D$**

2-Benzyl-1-(methanesulfonyl)azetidine-2-d 15a$_D$. To a solution of 2-benzylidene-1-(methanesulfonyl)azetidine **2a** (49 mg, 220 µmol) in anhydrous dichloromethane (1.1 mL) was added trifluoroacetic acid (84 µL, 1.1 mmol) dropwise at -78 °C under argon. The resulting mixture was stirred at -78 °C for 15 minutes and triethylsilane-d$_1$ (88 µL, 550 µmol) was then added in one portion. The resulting mixture was then slowly warmed to rt, stirred for 3 hours, quenched with a 1 M aqueous solution of NaOH and extracted with dichloromethane. The combined organic layers were then washed with brine, dried over $MgSO_4$, filtered and concentrated under reduced pressure. The crude residue was finally purified by flash column chromatography over silica gel (petroleum ether/EtOAc: 80/20) to afford the desired product as an off-white solid (34 mg, 150 µmol, 68%). Mp: 93 °C; ^{1}H NMR (400 MHz, $CDCl_3$): *δ* 7.33-7.28 (m, 2H), 7.26-7.19 (m, 3H), 3.90 (app. q, *J* = 8.4 Hz, 1H), 3.63 (ddd, *J* = 8.9, 7.7 and 4.1 Hz, 1H), 3.13 (A of AB syst., *J* = 13.7 Hz, 1H), 2.99 (B of AB syst., *J* = 13.7 Hz, 1H), 2.78 (s, 3H), 2.15 (app. td, *J* = 11.1 and 4.1 Hz, 1H), 2.07 (app. dt, *J* = 11.1 and 8.7 Hz, 1H); ^{13}C NMR (100 MHz, $CDCl_3$): *δ* 136.5, 129.6, 128.7, 126.9, 63.3 (t, *J* = 22.1 Hz), 47.1, 42.4, 37.1, 21.4; ESIHRMS *m/z* calcd for $C_{11}H_{15}DNO_2S$ [M+H]$^+$ 227.0959, found 227.0957.

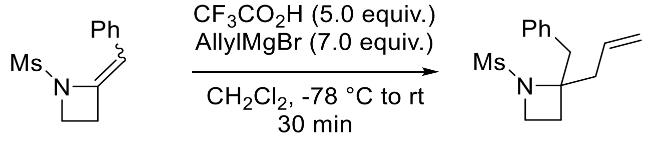

2a **16**

2-Allyl-2-benzyl-1-(methanesulfonyl)azetidine 16. To a solution of 2-benzylidene-1-(methanesulfonyl)azetidine **2a** (45 mg, 200 µmol) in anhydrous dichloromethane (1.0 mL) was added trifluoroacetic acid (77 µL, 1.0 mmol) at -78 °C under argon. The resulting mixture was stirred at -78 °C for 1 minute and allylmagnesium bromide (1 M solution in Et_2O, 1.4 mL,

1.4 mmol) was then added. The resulting mixture was then slowly warmed to rt, stirred for 30 minutes, quenched with a saturated aqueous solution of NH_4Cl and extracted with dichloromethane. The combined organic layers were then washed with brine, dried over $MgSO_4$, filtered and concentrated under reduced pressure. The crude residue was finally purified by flash column chromatography over silica gel (pentane/EtOAc: 60/40) to afford the desired product as a pale yellow oil (39 mg, 147 µmol, 73%). ^{1}H NMR (400 MHz, $CDCl_3$): *δ* 7.36-7.24 (m, 5H), 5.97 (dddd, *J* = 16.8, 10.3, 7.9 and 6.4 Hz, 1H), 5.25-5.16 (m, 2H), 3.70 (dt, *J* = 9.3 and 6.7 Hz, 1H), 3.42 (ddd, *J* = 9.2, 6.8 and 5.4 Hz, 1H), 3.26 (A of AB syst., *J* = 13.8 Hz, 1H), 2.96 (B of AB syst., *J* = 13.8 Hz, 1H), 2.88 (obs. ddt, *J* = 14.0, 6.4 and 1.3 Hz, 1H), 2.87 (s, 3H), 2.58 (app. dd, *J* = 13.9 and 7.9 Hz, 1H), 2.17 (ddd, *J* = 11.1, 9.2 and 6.5 Hz, 1H), 2.03 (ddd, *J* = 11.1, 9.3 and 5.4 Hz, 1H); ^{13}C NMR (100 MHz, $CDCl_3$): *δ* 136.9, 133.0, 130.9, 128.3, 126.8, 119.7, 76.6, 45.8, 44.2, 42.8, 40.7, 23.5; ESIHRMS *m/z* calcd for $C_{14}H_{20}NO_2S$ $[M+H]^+$ 266.1209, found 266.1209.

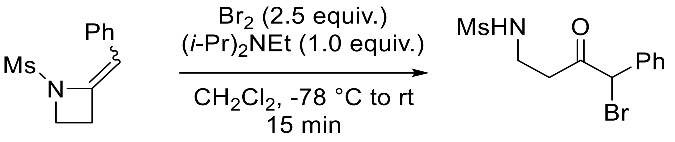

2a **17**

***N*-(4-Bromo-3-oxo-4-phenylbutyl)methanesulfonamide 17**. To a solution of 2-benzylidene-1-(methanesulfonyl)azetidine **2a** (22 mg, 99 µmol) in anhydrous dichloromethane (1.0 mL) were added bromine (13 µL, 254 µmol) and *N*,*N*-diisopropylethylamine (19 µL, 109 µmol) dropwise at -78 °C under argon. The resulting mixture was then slowly warmed to rt, stirred for 15 minutes, quenched with a saturated aqueous solution of $Na_2S_2O_3$ and extracted with dichloromethane. The combined organic layers were then washed with brine, dried over $MgSO_4$, filtered and concentrated under reduced pressure. The crude residue was finally purified by flash column chromatography over silica gel (petroleum ether/EtOAc: 50/50) to afford the desired product as a colorless oil (25 mg, 78 µmol, 79%). ^{1}H NMR (600 MHz, $CDCl_3$): *δ* 7.44-7.41 (m, 2H), 7.40-7.34 (m, 3H), 5.45 (s, 1H), 4.84 (t, *J* = 6.4 Hz, 1H), 3.35 (app. q., *J* = 5.8 Hz, 2H), 2.94 (dd, *J* = 6.4 and 5.4 Hz, 2H), 2.90 (s, 3H); ^{13}C NMR (150 MHz, $CDCl_3$): δ 201.1, 134.6, 129.6, 129.3, 129.0, 55.2, 40.4, 39.4, 38.3; ESIHRMS *m/z* calcd for $C_{11}H_{18}{}^{79}BrN_2O_3S$

$[M+NH_4]^+$ 337.0216, found 337.0217; ESIHRMS *m/z* calcd for $C_{11}H_{18}{}^{81}BrN_2O_3S$ $[M+NH_4]^+$ 339.0196, found 339.0196.

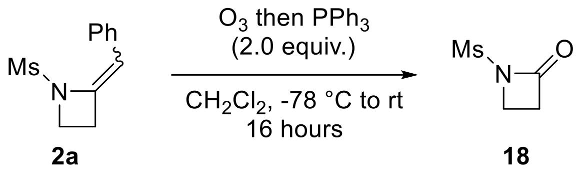

1-(Methanesulfonyl)azetidin-2-one 18. A solution of 2-benzylidene-1-(methanesulfonyl)azetidine **2a** (25 mg, 112 µmol) in anhydrous dichloromethane (2.0 mL) was saturated with ozone at -78 °C. The resulting mixture was then warmed to rt, stirred for 15 minutes and triphenylphosphine (59 mg, 224 µmol) was then added in one portion. The resulting mixture was then stirred at rt overnight and concentrated under reduced pressure. The crude residue was finally purified by flash column chromatography over silica gel (petroleum ether/EtOAc: 60/40) to afford the desired product as an off-white solid (11 mg, 74 µmol, 66%). Mp: 95 °C; ^1H NMR (400 MHz, $CDCl_3$): δ 3.77 (t, J = 5.1 Hz, 2H), 3.19 (s, 3H), 3.17 (t, J = 5.1 Hz, 2H); ^{13}C NMR (100 MHz, $CDCl_3$): δ 164.0, 41.6, 40.2, 37.1; ESIHRMS *m/z* calcd for $C_4H_7NO_3SNa$ $[M+Na]^+$ 172.0039, found 172.0038.

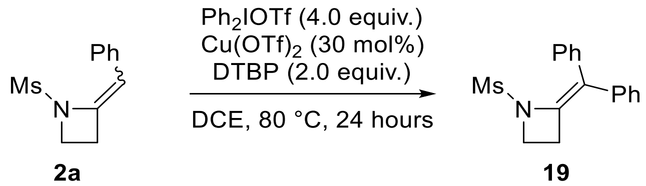

2-(Diphenylmethylene)-1-(methanesulfonyl)azetidine 19. A pressure tube was charged with 2-benzylidene-1-(methanesulfonyl)azetidine **2a** (50 mg, 224 µmol), diphenyliodonium trifluoromethanesulfonate (385 mg, 896 µmol) and copper(II) trifluoromethanesulfonate (24 mg, 67 µmol). The tube was fitted with a rubber septum, evacuated under high vacuum and backfilled with argon three times. Anhydrous 1,2-dichloroethane (560 µL) and 2,6-di-*tert*-butylpyridine (DTBP, 100 µL, 448 µmol) were then added, the rubber septum was replaced by a Teflon-coated screw cap and the resulting mixture was stirred at 80 °C for 24 hours. The reaction mixture was allowed to cool down to rt before being quenched with a saturated

aqueous solution of $NaHCO_3$ and extracted with dichloromethane. The combined organic layers were then washed with brine, dried over $MgSO_4$, filtered and concentrated under reduced pressure. The crude residue was finally purified by flash column chromatography over silica gel (petroleum ether/EtOAc: 85/15) to afford the desired product as a pale yellow solid (30 mg, 100 µmol, 45%). Mp: 101 °C; ^{1}H NMR (400 MHz, $CDCl_3$): δ 7.40-7.34 (m, 4H), 7.33-7.24 (m, 3H), 7.22-7.16 (m, 1H), 7.15-7.11 (m, 2H), 4.13 (t, J = 6.9 Hz, 2H), 3.25 (t, J = 6.9 Hz, 2H), 2.28 (s, 3H); ^{13}C NMR (100 MHz, $CDCl_3$): δ 139.4, 139.1, 139.0, 131.2, 128.5, 128.5, 128.2, 127.7, 126.8, 124.2, 47.9, 40.2, 28.9; ESIHRMS *m/z* calcd for $C_{17}H_{18}NO_2S$ $[M+H]^+$ 300.1053, found 300.1050.

2.12. Experimental Procedures and Characterization Data: Isomerization of Azetidines

General procedure

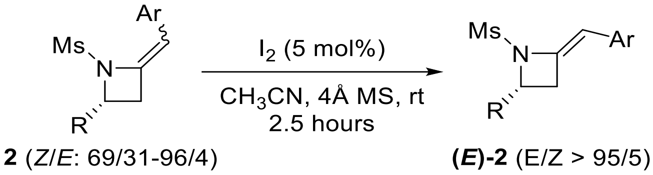

To a solution of azetidine **2** (*Z/E* ratio of 69/31- 96/4, 1.0 equiv.) and 4Å molecular sieves (200 mg/mmol) in anhydrous acetonitrile (0.04 M) was added iodine (5 mol%) at rt under argon. The resulting mixture was stirred at rt for 2.5 hours, filtered and concentrated under reduced pressure. The crude residue was finally purified by flash column chromatography over silica gel to afford the desired (*E*)-azetidine **(*E*)-2** (*E/Z* ratio >95/5).

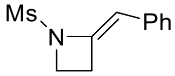

(*E*)-2a

(*E*)-2-Benzylidene-1-(methanesulfonyl)azetidine (*E*)-2a. Prepared according to general procedure (112 µmol of azetidine **2aa** with a *Z/E* ratio of 85/15). Yield: 88% (22 mg, 99 µmol). Obtained with a *E/Z* ratio of >95/5 after purification. Solvent system for flash column chromatography: petroleum ether/EtOAc: 75/25; White solid.*

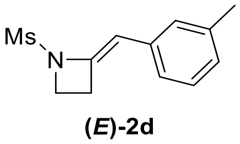

(*E*)-2d

(*E*)-1-(Methanesulfonyl)-2-(*m*-methylbenzylidene)azetidine (*E*)-2d. Prepared according to general procedure (97 µmol of azetidine **2d** with a *Z/E* ratio of 84/16). Yield: 87% (20 mg, 84 µmol). Obtained with a *E/Z* ratio of >95/5 after purification. Solvent system for flash column chromatography: petroleum ether/EtOAc: 70/30; Pale yellow solid; Mp: 127 °C; ^{1}H NMR (400 MHz, $CDCl_3$): δ 7.19 (app. t, J = 7.4 Hz, 1H), 7.00-6.93 (m, 3H), 6.22 (t, J = 2.4 Hz, 1H), 4.07 (t, J

* See page 92 for full characterization.

= 6.5 Hz, 2H), 3.24 (td, *J* = 6.5 and 2.4 Hz, 2H), 2.98 (s, 3H), 2.33 (s, 3H); ^{13}C NMR (100 MHz, $CDCl_3$): *δ* 142.8, 138.4, 135.5, 128.7, 127.6, 127.0, 123.6, 107.0, 49.7, 34.3, 27.4, 21.6.

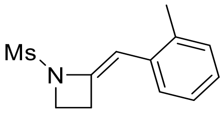

(*E*)-2e

(*E*)-1-(Methanesulfonyl)-2-(*o*-methylbenzylidene)azetidine (*E*)-2e. Prepared according to general procedure (93 µmol of azetidine **2e** with a *Z*/*E* ratio of 87/13). Yield: 86% (19 mg, 80 µmol). Obtained with a *E*/*Z* ratio of >95/5 after purification. Solvent system for flash column chromatography: petroleum ether/EtOAc: 70/30; Pale yellow solid; Mp: 107 °C; ^{1}H NMR (400 MHz, $CDCl_3$): *δ* 7.17 (app. d, *J* = 7.0 Hz, 1H), 7.15-7.07 (m, 3H), 6.39 (t, *J* = 2.4 Hz, 1H), 4.05 (t, *J* = 6.5 Hz, 2H), 3.18 (td, *J* = 6.5 and 2.4 Hz, 2H), 3.00 (s, 3H), 2.31 (s, 3H); ^{13}C NMR (100 MHz, $CDCl_3$): *δ* 142.9, 135.6, 133.8, 130.6, 126.5, 126.0, 125.9, 104.6, 49.5, 34.1, 26.9, 20.1.

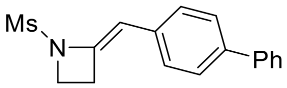

(*E*)-2g

(*E*)-1-(Methanesulfonyl)-2-(*p*-phenylbenzylidene)azetidine (*E*)-2g. Prepared according to general procedure (80 µmol of azetidine **2g** with a *Z*/*E* ratio of 77/23). Yield: 84% (20 mg, 67 µmol). Obtained with a *E*/*Z* ratio of >95/5 after purification. Solvent system for flash column chromatography: petroleum ether/EtOAc: 70/30; Pale yellow solid; Mp: 181 °C; ^{1}H NMR (400 MHz, $CDCl_3$): *δ* 7.61-7.57 (m, 2H), 7.55 (d, *J* = 8.3 Hz, 2H), 7.44 (app. t, *J* = 7.3 Hz, 2H), 7.36-7.31 (m, 1H), 7.22 (d, *J* = 8.4 Hz, 2H), 6.30 (t, *J* = 2.4 Hz, 1H), 4.10 (t, *J* = 6.5 Hz, 2H), 3.28 (td, *J* = 6.5 and 2.4 Hz, 2H), 3.00 (s, 3H); ^{13}C NMR (100 MHz, $CDCl_3$): *δ* 143.1, 140.7, 138.9, 134.7, 128.9, 127.5, 127.4, 127.0, 126.9, 106.5, 49.8, 34.5, 27.4.

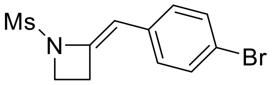

(*E*)-2l

(*E*)-2-(*p*-Bromobenzylidene)-1-(methanesulfonyl)azetidine (*E*)-2l. Prepared according to general procedure (99 µmol of azetidine **2l** with a *Z*/*E* ratio of 86/14). Yield: 80% (24 mg, 79 µmol). Obtained with a *E*/*Z* ratio of >95/5 after purification. Solvent system for flash column chromatography: petroleum ether/EtOAc: 80/20; Yellow solid; Mp: 144 °C; ^{1}H NMR (400 MHz, $CDCl_3$): *δ* 7.40 (d, *J* = 8.5 Hz, 2H), 6.99 (d, *J* = 8.5 Hz, 2H), 6.17 (t, *J* = 2.4 Hz, 1H), 4.08 (t, *J* = 6.4 Hz, 2H), 3.20 (td, *J* = 6.4 and 2.4 Hz, 2H), 2.98 (s, 3H); ^{13}C NMR (100 MHz, $CDCl_3$): *δ* 143.6, 134.6, 131.9, 128.1, 119.6, 105.6, 49.7, 34.9, 27.3.

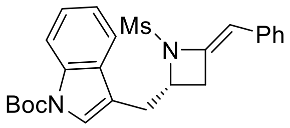

(*E*)-2aa

(*S*,*E*)-2-Benzylidene-4-[(*N*-*tert*-butoxycarbonyl-1*H*-indol-3-yl)methyl]-1-(methanesulfonyl) azetidine (*E*)-2aa. Prepared according to general procedure (88 µmol of azetidine **2aa** with a *Z*/*E* ratio of 84/16). Yield: 85% (34 mg, 75 µmol). Obtained with a *E*/*Z* ratio of >95/5 after purification. Solvent system for flash column chromatography: petroleum ether/EtOAc: 85/15; White solid; Mp: 154 °C; $[\alpha]_D^{20}$ + 41 (*c* 1.3, $CHCl_3$); ^{1}H NMR (400 MHz, $CDCl_3$): *δ* 8.14 (d, *J* = 7.6 Hz, 1H), 7.61 (d, *J* = 7.6 Hz, 1H), 7.49 (s, 1H), 7.38-7.33 (m, 1H), 7.32-7.25 (m, 3H), 7.18-7.10 (m, 3H), 6.28 (t, *J* = 2.3 Hz, 1H), 4.57 (dddd, *J* = 9.9, 7.4, 4.9 and 3.7 Hz, 1H), 3.53 (ddd, *J* = 14.6, 3.7 and 1.2 Hz, 1H), 3.22 (obs. ddd, *J* = 15.5, 7.3 and 2.1 Hz, 1H), 3.18 (obs. dd, *J* = 14.8 and 9.9 Hz, 1H), 3.04 (s, 3H), 2.99 (ddd, *J* = 15.5, 4.9 and 2.4 Hz, 1H), 1.69 (s, 9H); ^{13}C NMR (100 MHz, $CDCl_3$): *δ* 149.8, 140.5, 135.6, 135.5, 130.5, 128.8, 126.7, 126.3, 124.8, 123.8, 122.9, 119.0, 115.5, 114.8, 107.2, 84.0, 62.3, 34.8, 33.6, 31.0, 28.3.

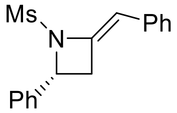

(*E*)-2ab

(*R,E*)-2-Benzylidene-1-(methanesulfonyl)-4-phenylazetidine (*E*)-2ab. Prepared according to general procedure (57 µmol of azetidine **2ab** with a *Z*/*E* ratio of 88/12). Yield: 82% (14 mg, 47 µmol). Obtained with a *E*/*Z* ratio of >95/5 after purification. Solvent system for flash column chromatography: petroleum ether/EtOAc: 70/30; Pale yellow solid; Mp: 146 °C; $[\alpha]_D^{20}$ + 22 (*c* 0.6, $CHCl_3$); ^1H NMR (400 MHz, $CDCl_3$): δ 7.54-7.51 (m, 2H), 7.44-7.39 (m, 2H), 7.38-7.33 (m, 1H), 7.33-7.28 (m, 2H), 7.20-7.14 (m, 3H), 6.40 (t, *J* = 2.3 Hz, 1H), 5.34 (dd, *J* = 7.9 and 4.9 Hz, 1H), 3.66 (ddd, *J* = 15.4, 7.9 and 2.4 Hz, 1H), 3.21 (ddd, *J* = 15.4, 4.9 and 2.4 Hz, 1H), 2.92 (s, 3H); ^{13}C NMR (100 MHz, $CDCl_3$): δ 140.8, 138.8, 135.8, 129.0, 128.9, 128.8, 126.7, 126.6, 126.2, 107.1, 64.9, 37.1, 36.6.

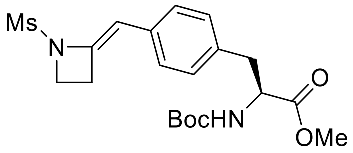

(*E*)-2ag

(*S,E*)-2-{4-{2-[(*tert*-Butoxycarbonyl)amino]-2-methoxycarbonylethyl}benzylidene}-1-(methanesulfonyl)azetidine (*E*)-2ag. Prepared according to general procedure (92 µmol of azetidine **2ag** with a *Z*/*E* ratio of 94/6). Yield: 87% (34 mg, 80 µmol). Obtained with a *E*/*Z* ratio of >95/5 after purification. Solvent system for flash column chromatography: petroleum ether/EtOAc: 60/40; White solid; Mp: 119 °C; $[\alpha]_D^{20}$ + 0.2 (*c* 1.6, $CHCl_3$); ^1H NMR (400 MHz, $CDCl_3$): δ 7.07 (d, *J* = 8.9 Hz, 2H), 7.04 (d, *J* = 8.9 Hz, 2H), 6.20 (t, *J* = 2.4 Hz, 1H), 4.97 (d, *J* = 7.8 Hz, 1H), 4.55 (app. q, *J* = 6.6 Hz, 1H), 4.07 (t, *J* = 6.5 Hz, 2H), 3.71 (s, 3H), 3.22 (td, *J* = 6.5 and 2.3 Hz, 2H), 3.08 (A of ABX syst., *J* = 13.9 and 5.7 Hz, 1H), 2.99 (obs. B of ABX syst., *J* = 13.9 and 6.5 Hz, 1H), 2.97 (s, 3H), 1.41 (s, 9H); ^{13}C NMR (100 MHz, $CDCl_3$): δ 172.4, 155.2, 142.9, 134.4, 133.9, 129.7, 126.8, 106.5, 80.1, 54.5, 52.3, 49.7, 38.1, 34.4, 28.4, 27.3.

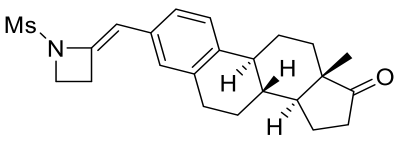

(*E*)-2ah

(8*R*,9*S*,13*S*,14*S*,*E*)-[2-(13-Methyl-17-oxo-7,8,9,11,12,13,14,15,16,17-decahydro-6*H*-cyclopenta[*a*]phenanthren-3-ylmethylene)]-1-(methanesulfonyl)azetidine (*E*)-2ah. Prepared according to general procedure (58 µmol of azetidine **2ah** with a *Z*/*E* ratio of 89/11). Yield: 86% (20 mg, 50 µmol). Obtained with a *E*/*Z* ratio of >95/5 after purification. Solvent system for flash column chromatography: dichloromethane/EtOAc: 100/0 then 90/10; Off-white solid; Mp: 228 °C; $[\alpha]_D^{20}$ + 121 (*c* 1.1, $CHCl_3$); ^1H NMR (400 MHz, $CDCl_3$): *δ* 7.23 (d, *J* = 8.1 Hz, 1H), 6.96 (dd, *J* = 8.1 and 1.4 Hz, 1H), 6.89 (s, 1H), 6.20 (t, *J* = 2.3 Hz, 1H), 4.06 (t, *J* = 6.5 Hz, 2H), 3.23 (td, *J* = 6.5 and 2.3 Hz, 2H), 2.96 (s, 3H), 2.88 (dd, *J* = 8.9 and 4.1 Hz, 2H), 2.51 (dd, *J* = 19.0 and 8.9 Hz, 1H), 2.44-2.37 (m, 1H), 2.33-2.23 (m, 1H), 2.20-2.10 (m, 1H), 2.09-1.92 (m, 3H), 1.70-1.37 (m, 6H), 0.90 (s, 3H); ^{13}C NMR (100 MHz, $CDCl_3$): *δ* 220.9, 142.5, 138.0, 136.9, 133.1, 127.5, 125.8, 123.9, 106.7, 50.6, 49.7, 48.1, 44.5, 38.3, 35.9, 34.1, 31.7, 29.5, 27.3, 26.6, 25.8, 21.7, 13.9.

2.13. Experimental Procedures and Characterization Data: Chemical Correlation for the Confirmation of the Structure of Azetidine 2b

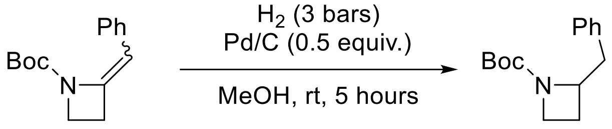

2-Benzyl-1-(*tert*-butoxycarbonyl)azetidine. A glass vial was charged with 2-benzylidene-1-(*tert*-butoxycarbonyl)azetidine **2b** (29 mg, 118 µmol), palladium on carbon 10% wt. (64 mg, 60 µmol) and methanol (25 mL). The vial was placed in an autoclave, purged with N_2, pressurized with H_2 (3 bars) and stirred at rt for 5 hours. After the reactor was depressurized and purged with N_2, the reaction mixture was filtered over a plug of Celite® (washed with ethyl acetate) and concentrated under reduced pressure. The crude residue was finally diluted with diethyl ether, filtered through a short pad of silica gel (washed with diethyl ether) and concentrated under reduced pressure to afford the desired product as a colorless oil (29 mg, 117 µmol, 99%). The spectroscopic data are in agreement with those reported in the literature.[8]

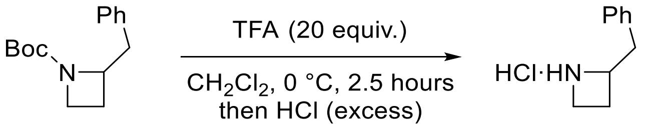

2-Benzylazetidine hydrochloride. To a solution of (±)-2-benzyl-1-(*tert*-butoxycarbonyl)azetidine (29 mg, 117 µmol) in anhydrous dichloromethane (1.4 mL) was added trifluoroacetic acid (179 µL, 2.34 mmol) dropwise at 0 °C under argon. The resulting mixture was stirred at 0 °C for 2.5 hours, quenched with a 1 M aqueous solution of NaOH and extracted with dichloromethane. The combined organic layers were then washed with brine, dried over $MgSO_4$, filtered and concentrated under reduced pressure. The crude residue was then diluted with diethyl ether before adding a 2 M solution of HCl in diethyl ether (excess). The resulting mixture was then concentrated under reduced pressure and the crude residue was triturated in pentane to finally afford the desired product as a grey solid (10 mg, 54 µmol, 46%). The spectroscopic data are in agreement with those reported in the literature.[9]

2.14. ^{1}H and ^{13}C NMR Spectra

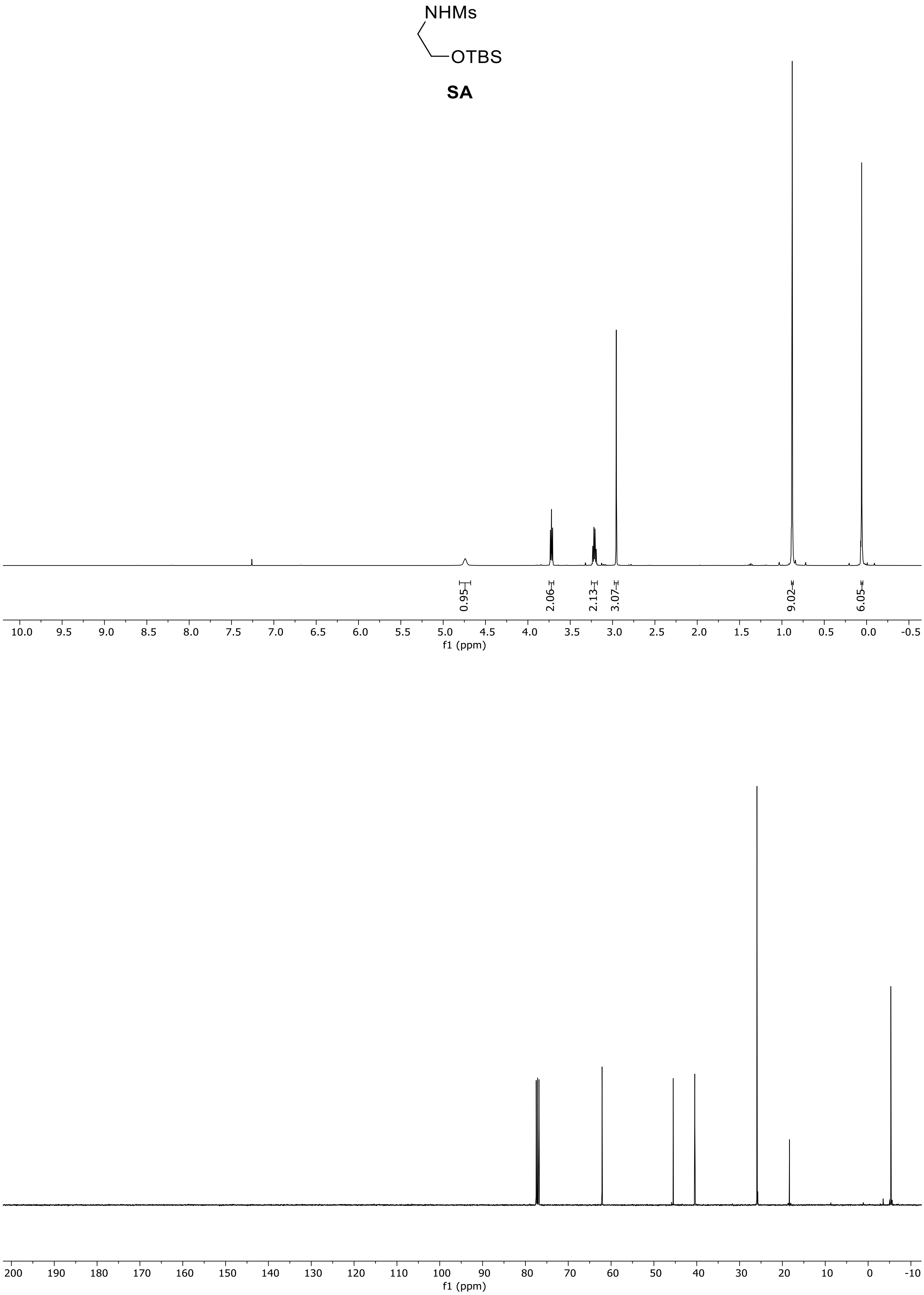

Supplementary Fig 1. ^{1}H (top) and ^{13}C (bottom) NMR spectra of compound **SA**.

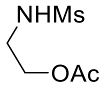

SB

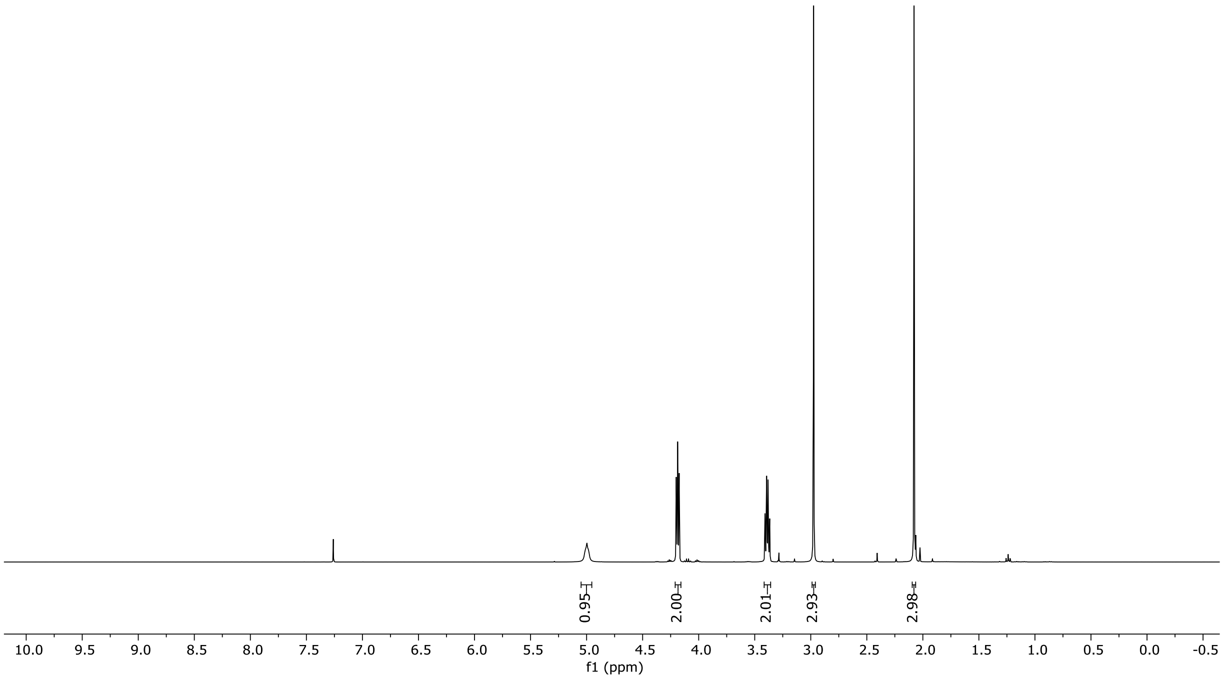

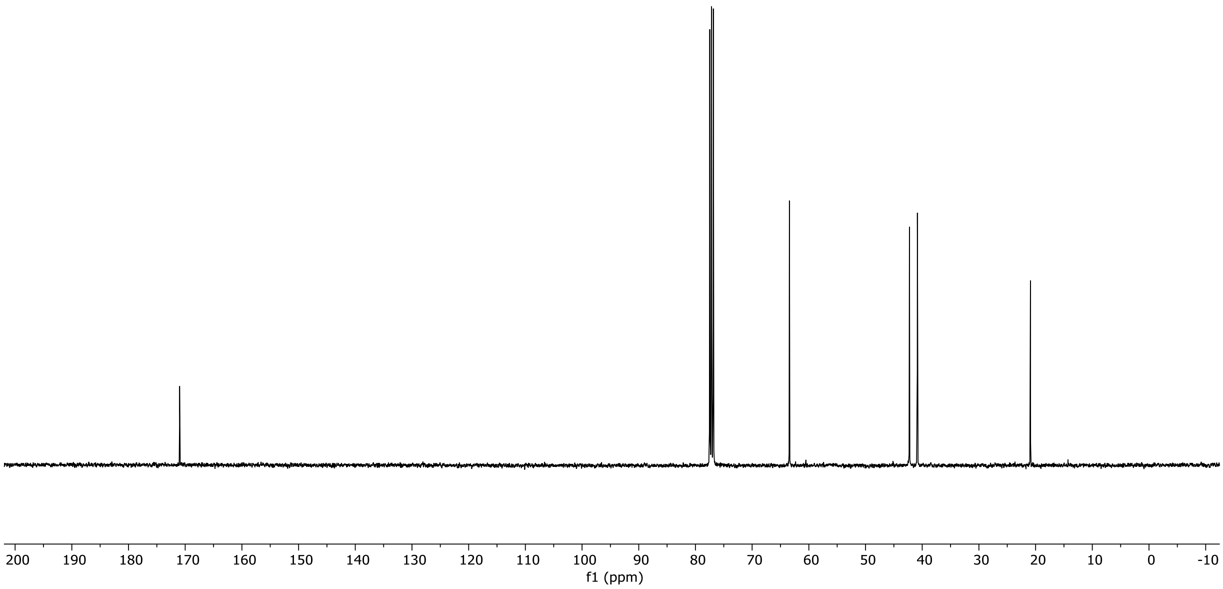

Supplementary Fig 2. ^{1}H (top) and ^{13}C (bottom) NMR spectra of compound **SB**.

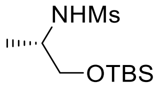

SC

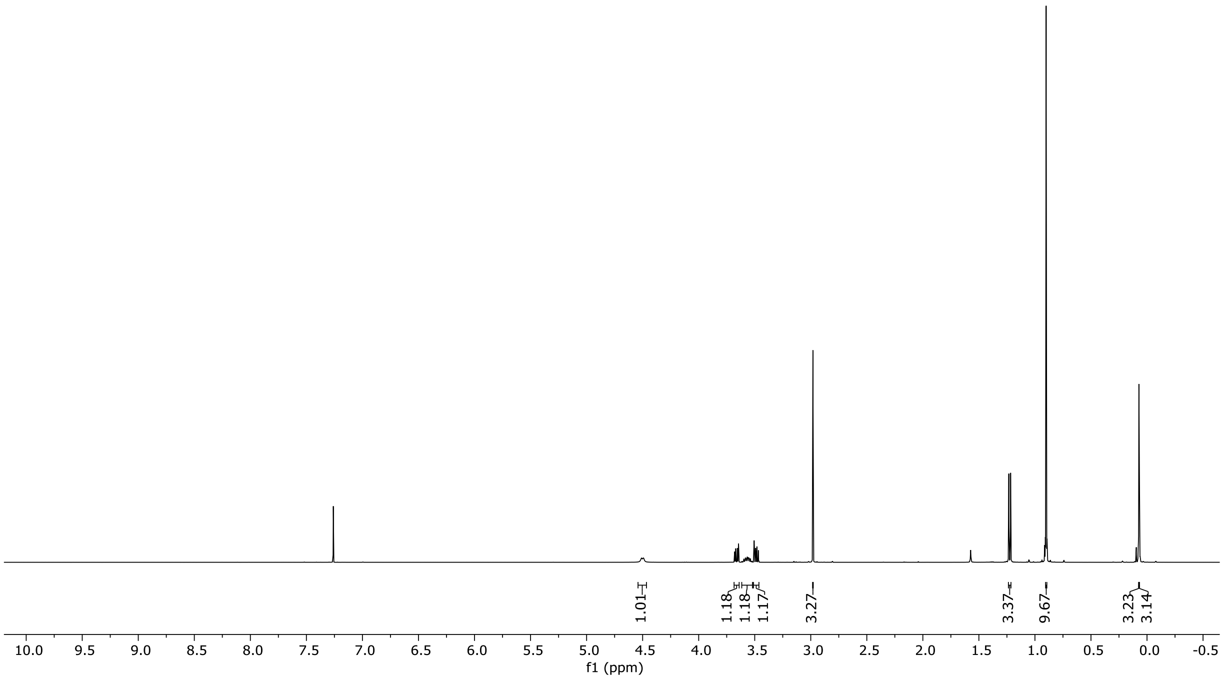

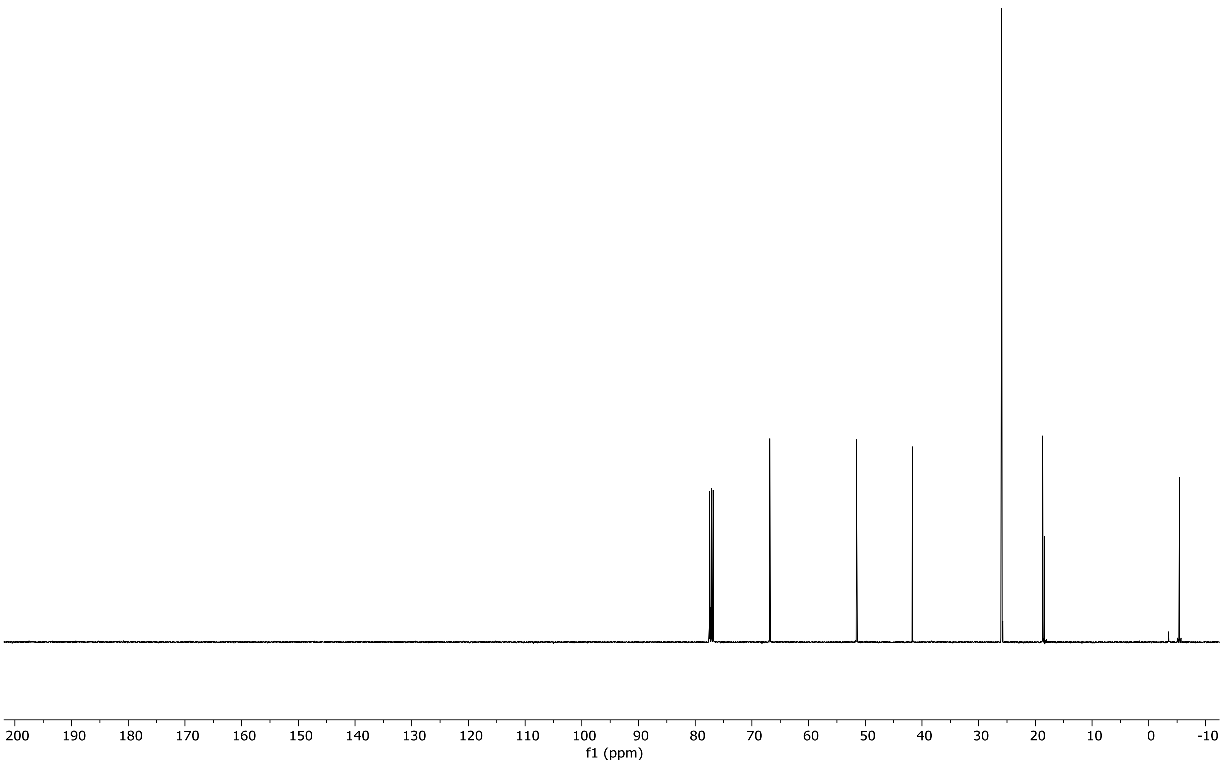

Supplementary Fig 3. ^{1}H (top) and ^{13}C (bottom) NMR spectra of compound **SC**.

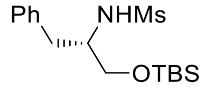

SD

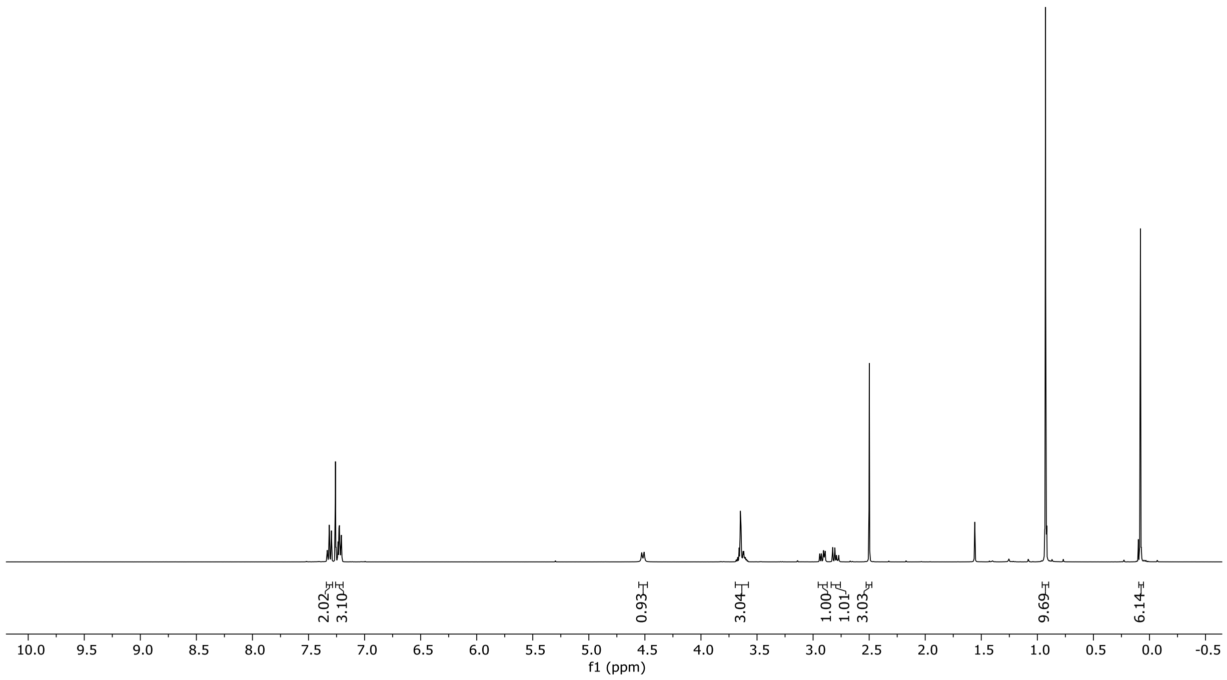

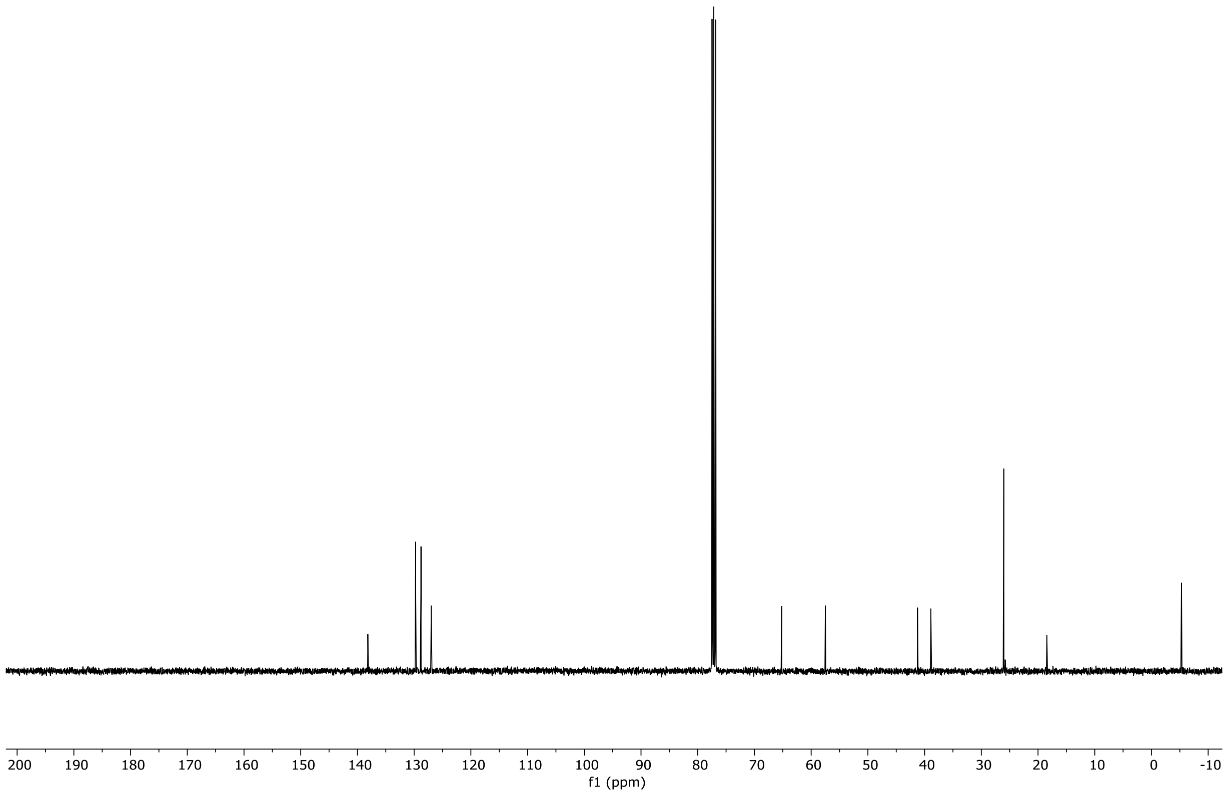

Supplementary Fig 4. ^{1}H (top) and ^{13}C (bottom) NMR spectra of compound **SD**.

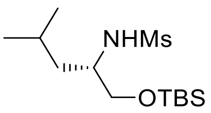

SE

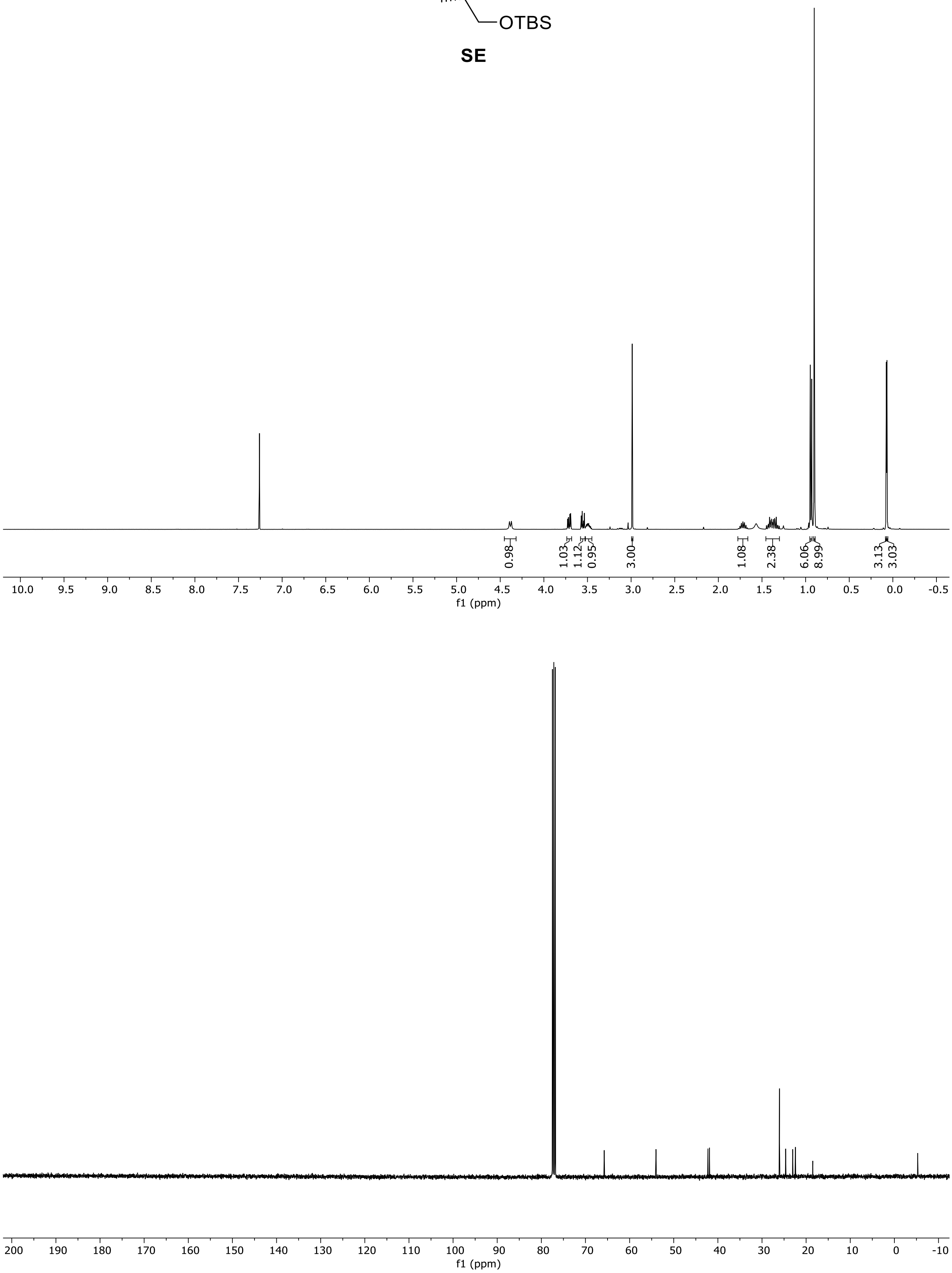

Supplementary Fig 5. ^{1}H (top) and ^{13}C (bottom) NMR spectra of compound **SE**.

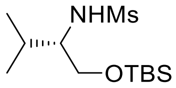

SF

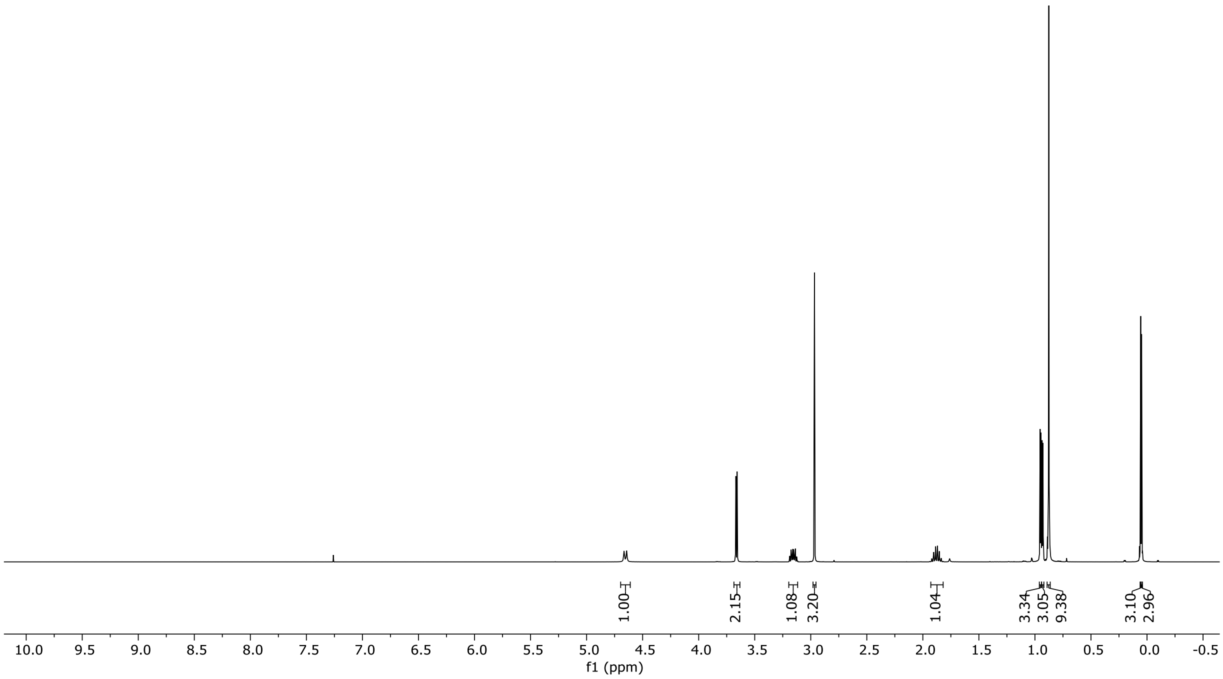

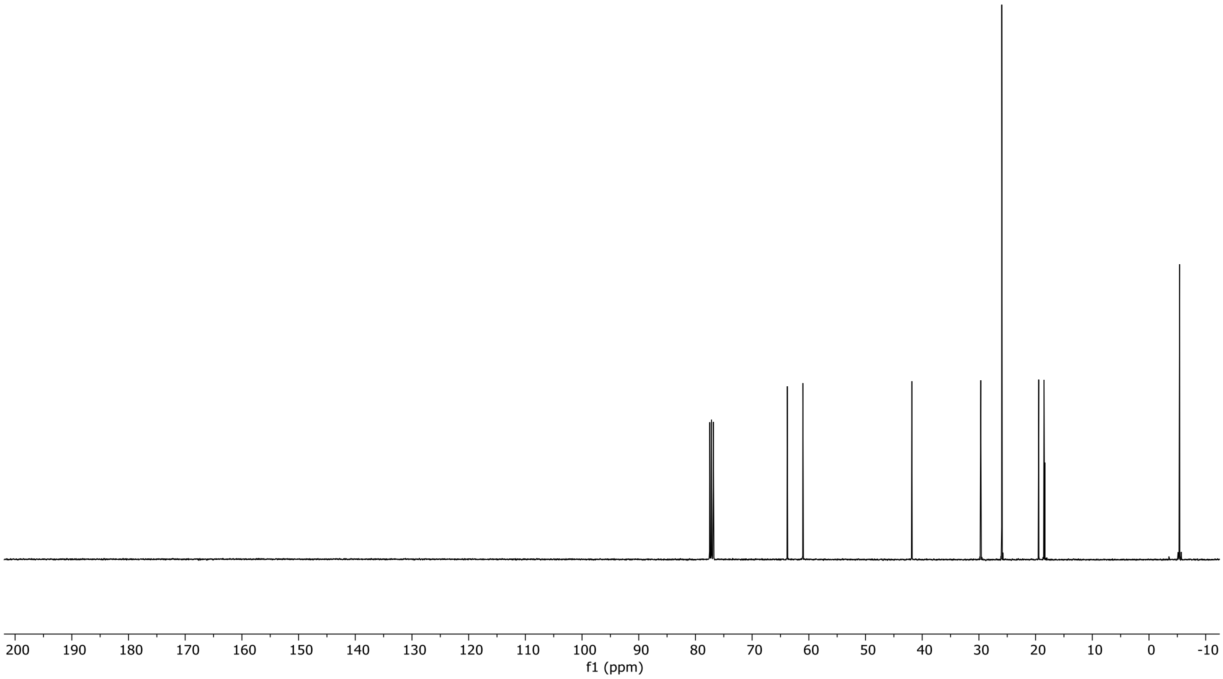

Supplementary Fig 6. ^{1}H (top) and ^{13}C (bottom) NMR spectra of compound **SF**.

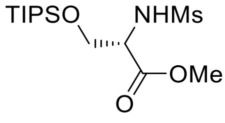

SG

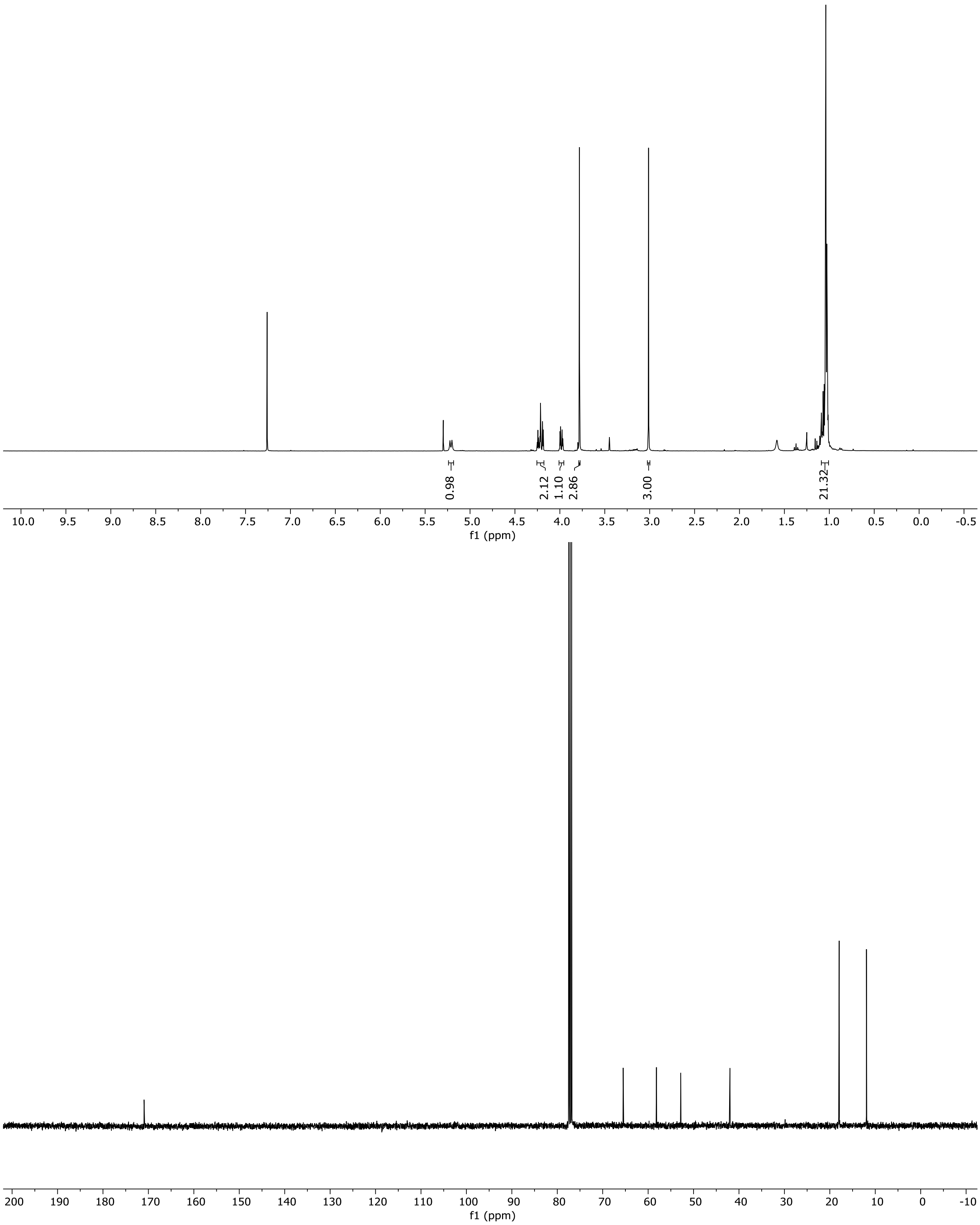

Supplementary Fig 7. ^{1}H (top) and ^{13}C (bottom) NMR spectra of compound **SG**.

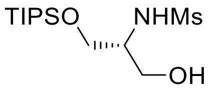

SH

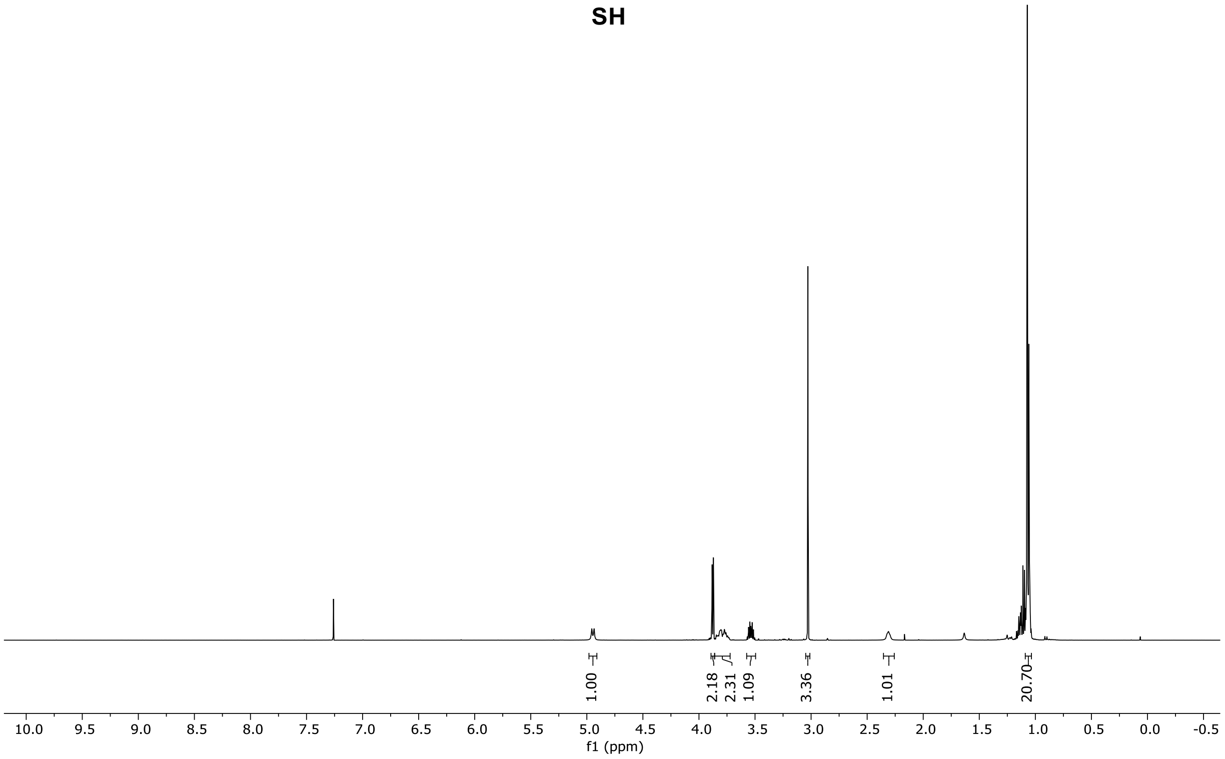

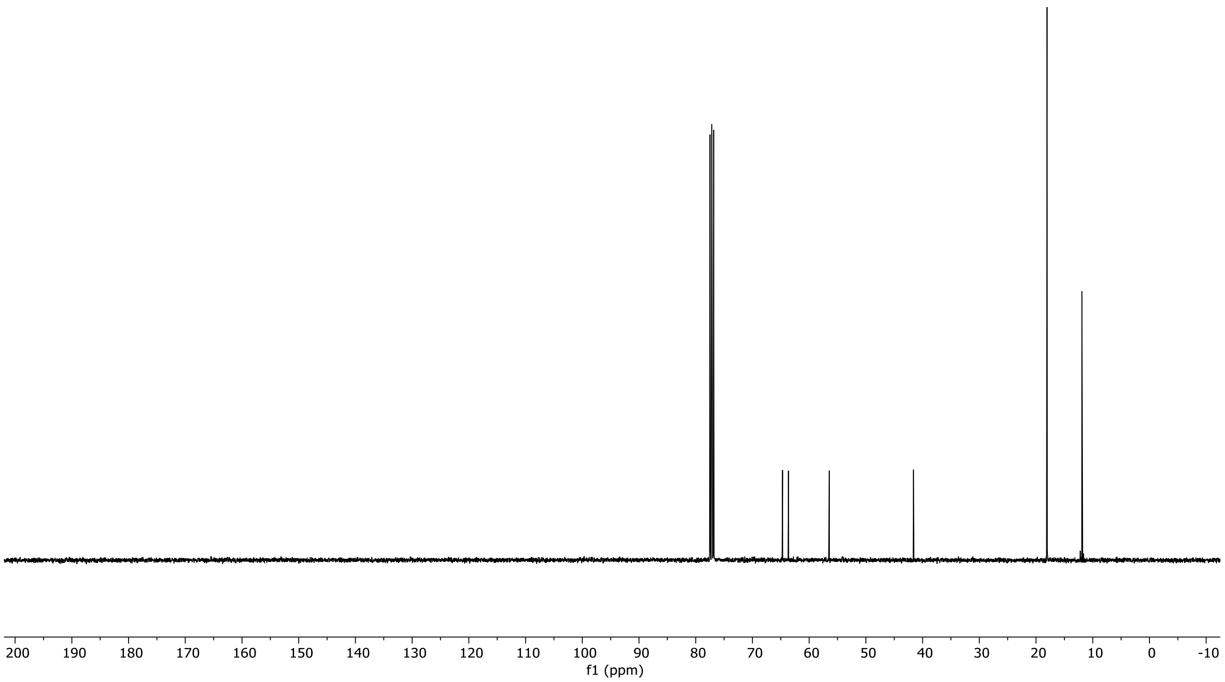

Supplementary Fig 8. ^{1}H (top) and ^{13}C (bottom) NMR spectra of compound **SH**.

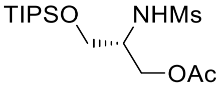

SI

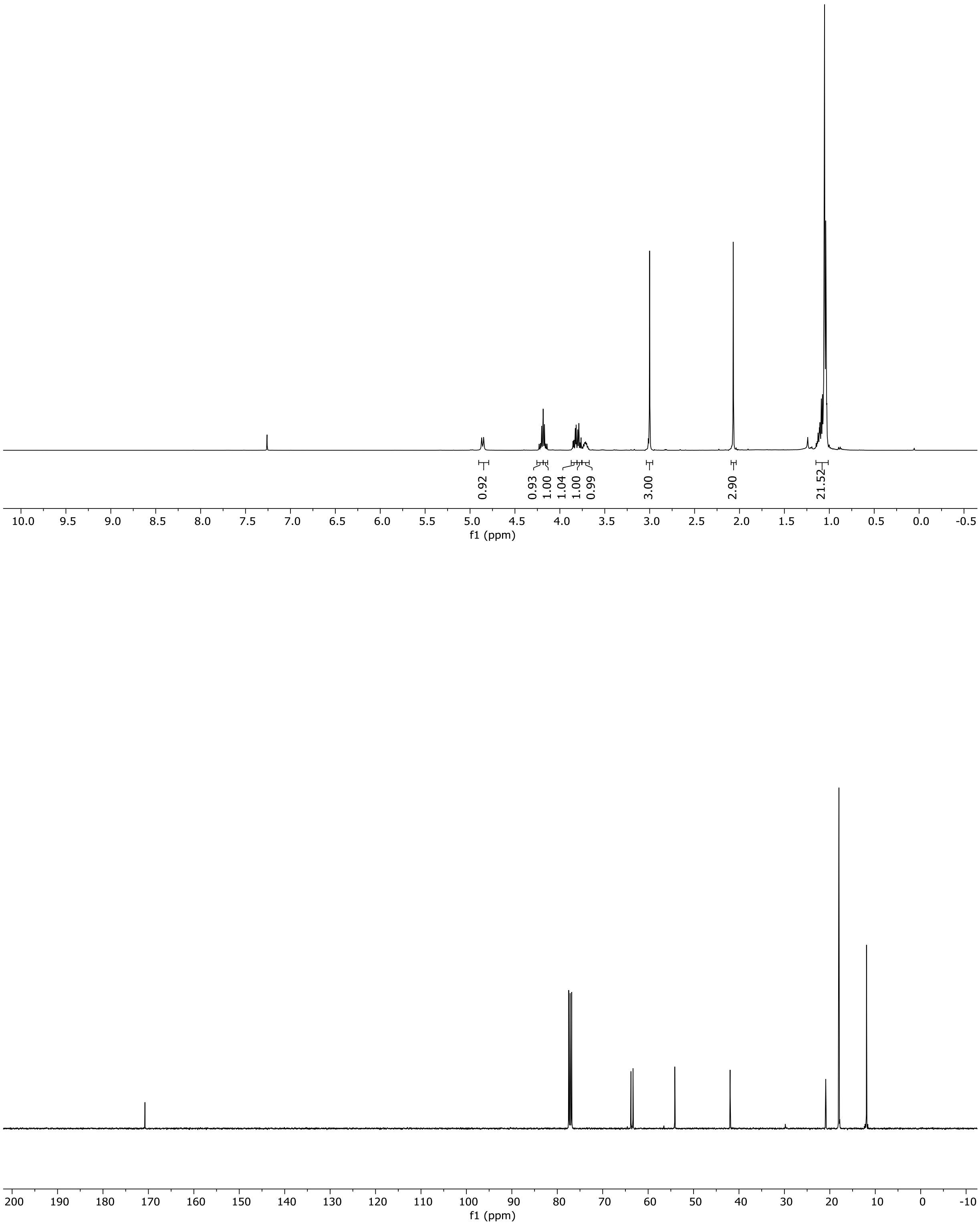

Supplementary Fig 9. ^{1}H (top) and ^{13}C (bottom) NMR spectra of compound **SI**.

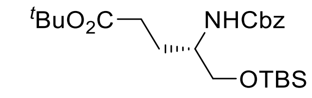

SJ

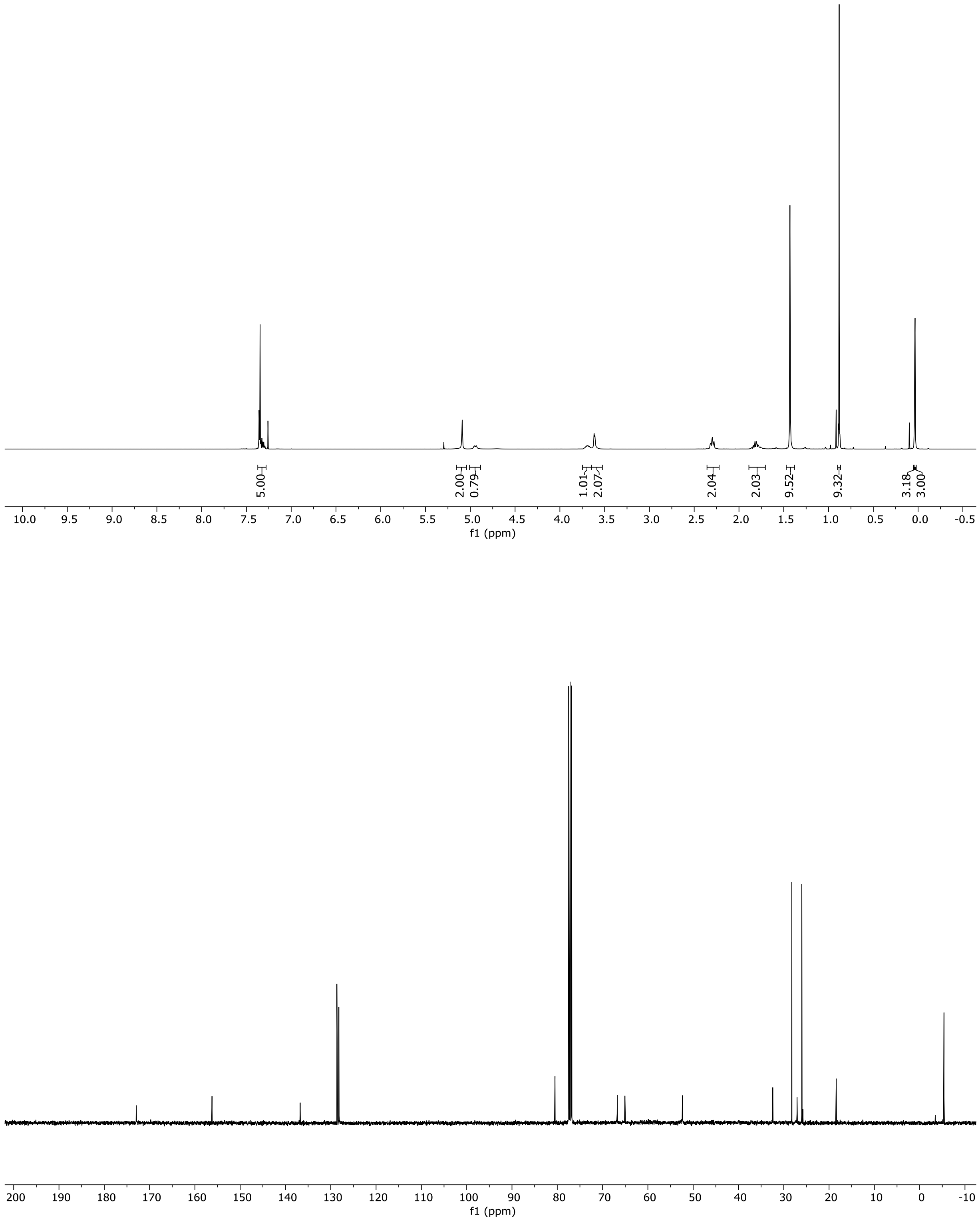

Supplementary Fig 10. ^{1}H (top) and ^{13}C (bottom) NMR spectra of compound **SJ**.

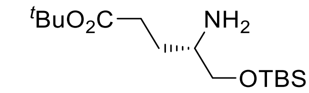

SK

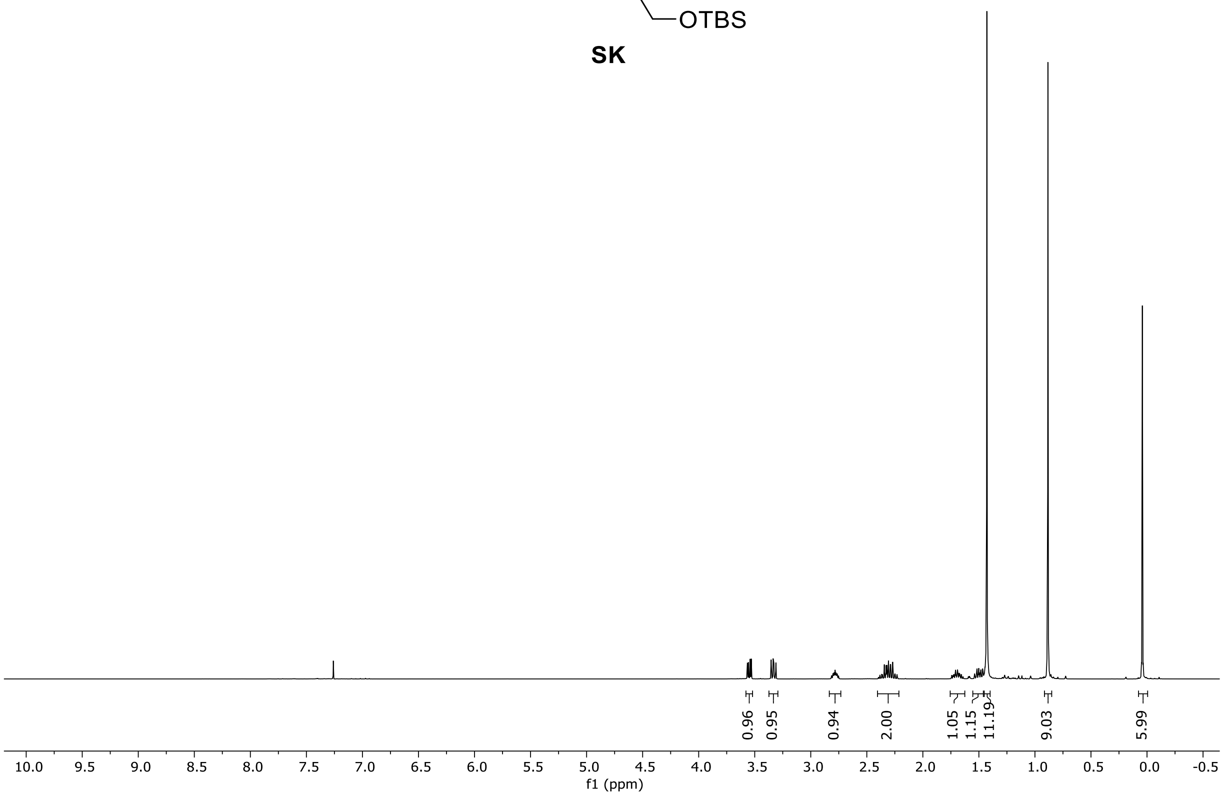

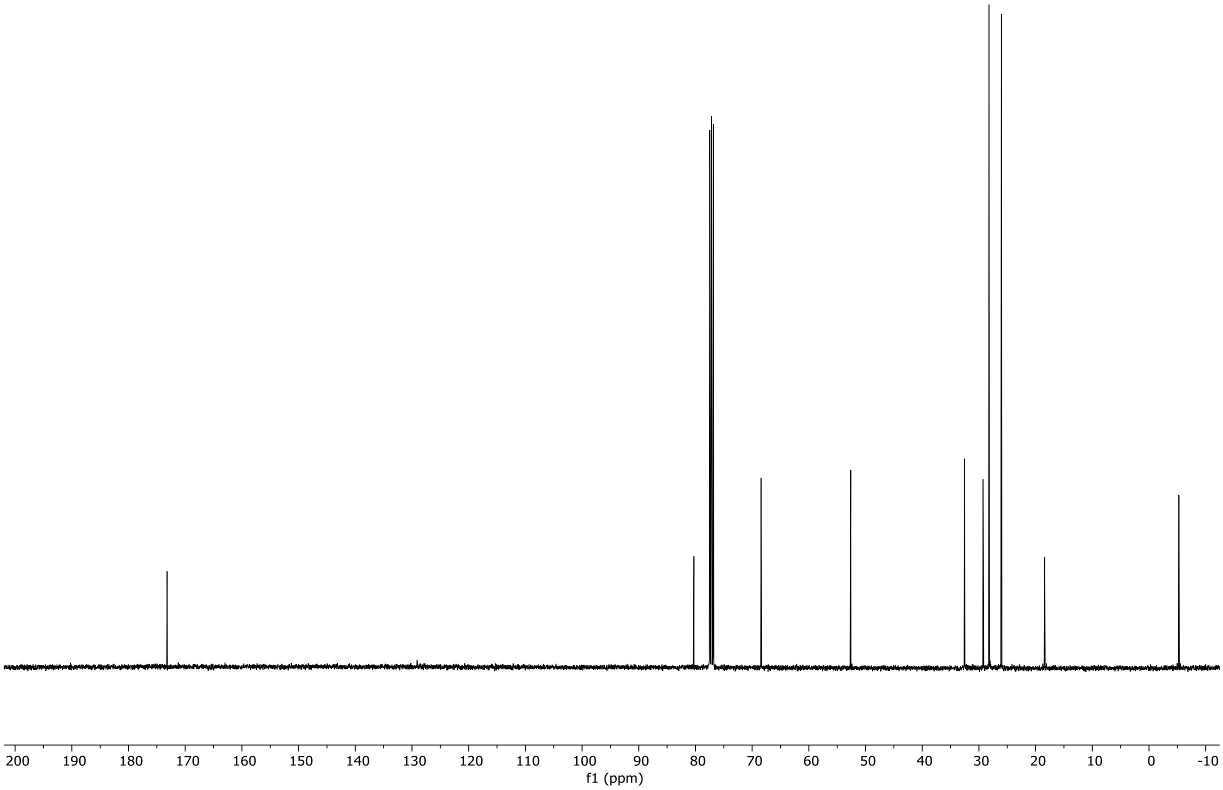

Supplementary Fig 11. ^{1}H (top) and ^{13}C (bottom) NMR spectra of compound **SK**.

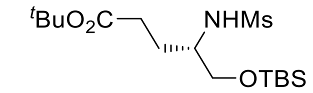

SL

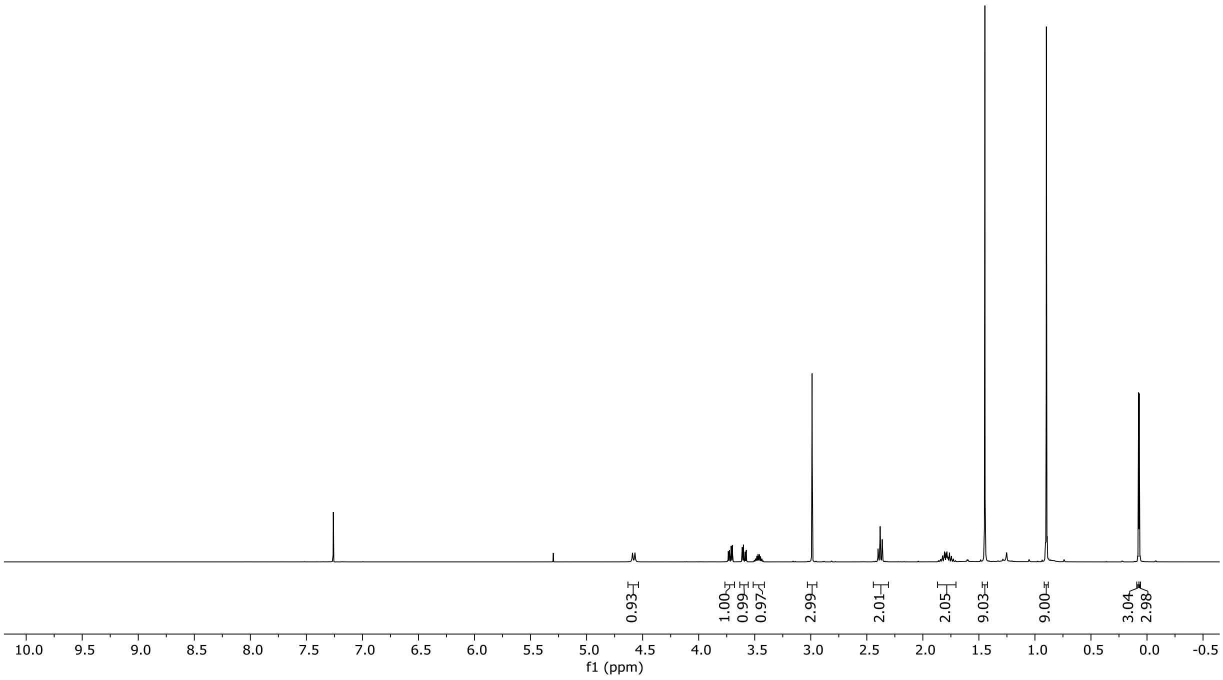

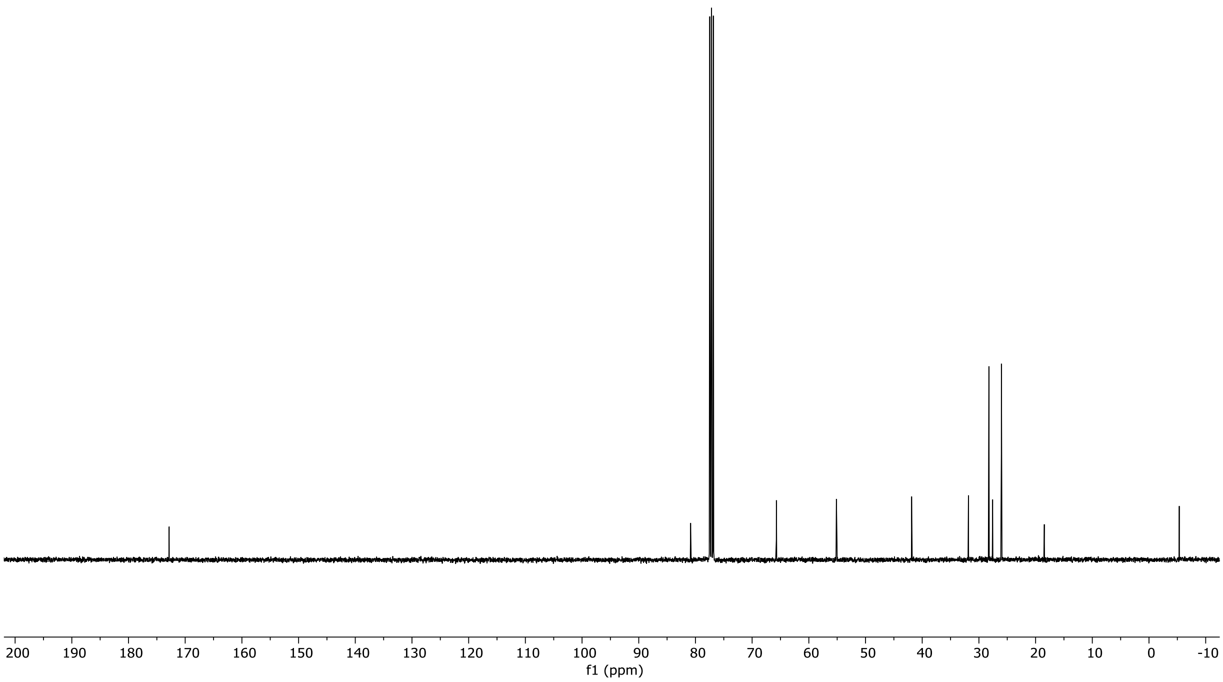

Supplementary Fig 12. ^1H (top) and ^{13}C (bottom) NMR spectra of compound **SL**.

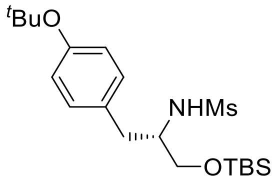

SM

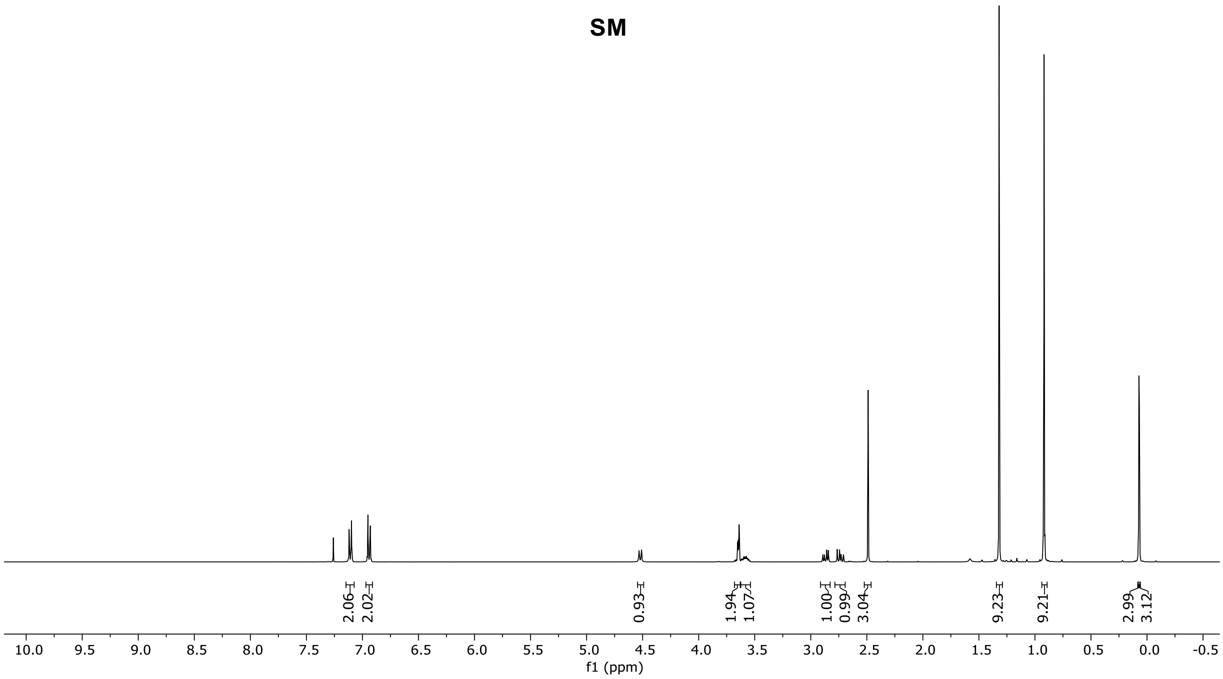

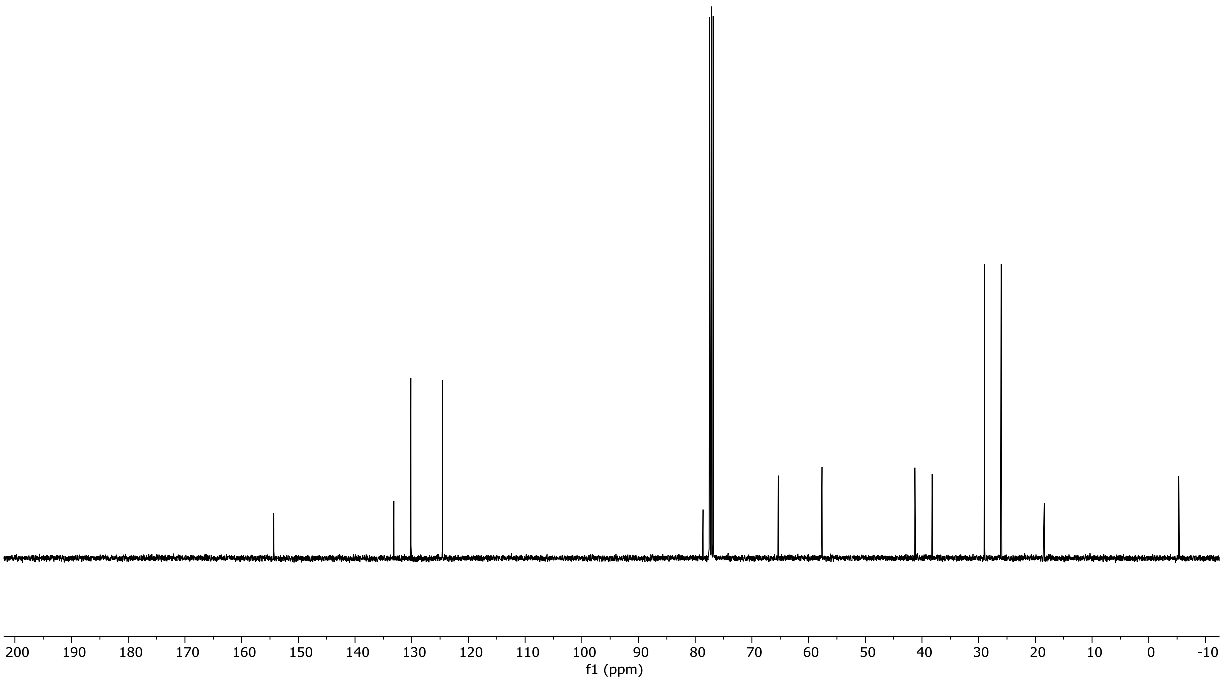

Supplementary Fig 13. ^{1}H (top) and ^{13}C (bottom) NMR spectra of compound **SM**.

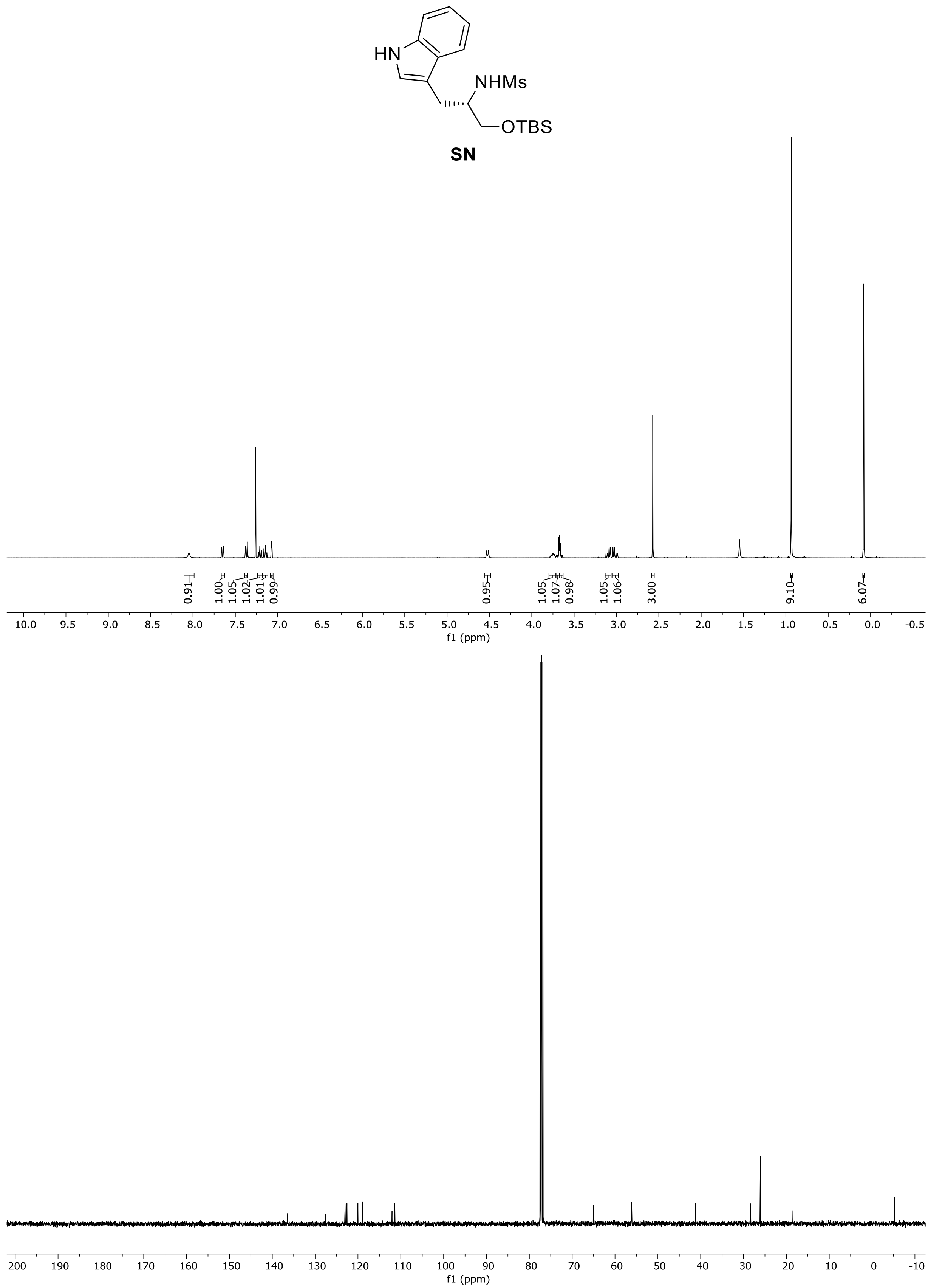

Supplementary Fig 14. ^{1}H (top) and ^{13}C (bottom) NMR spectra of compound **SN**.

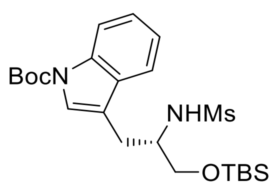

SO

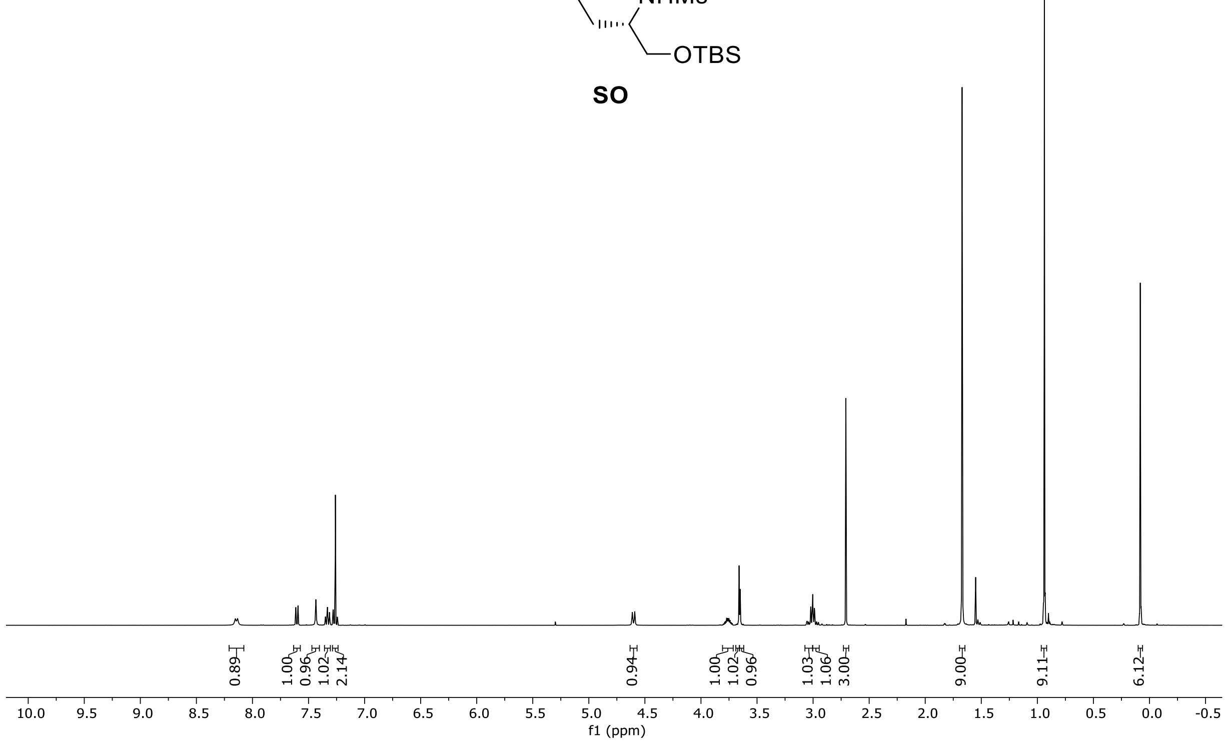

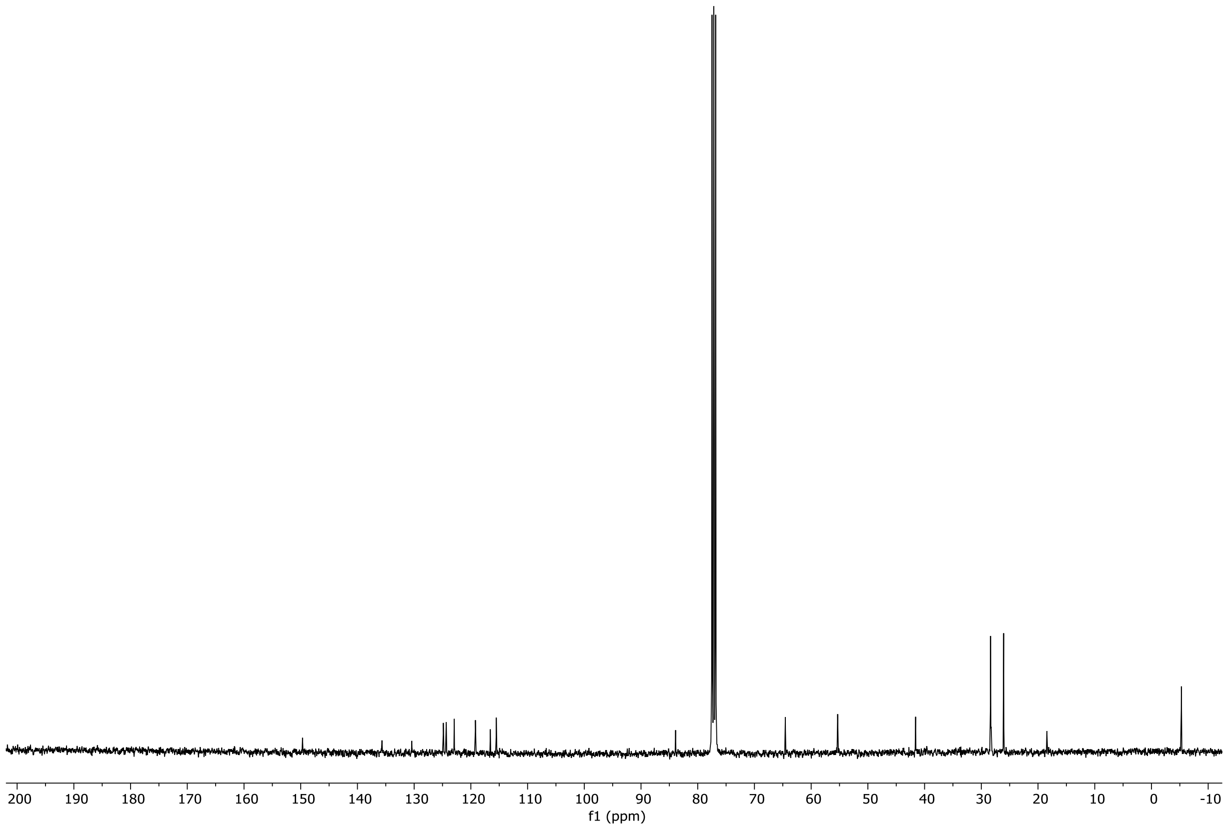

Supplementary Fig 15. ^{1}H (top) and ^{13}C (bottom) NMR spectra of compound **SO**.

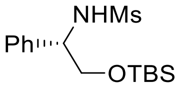

SP

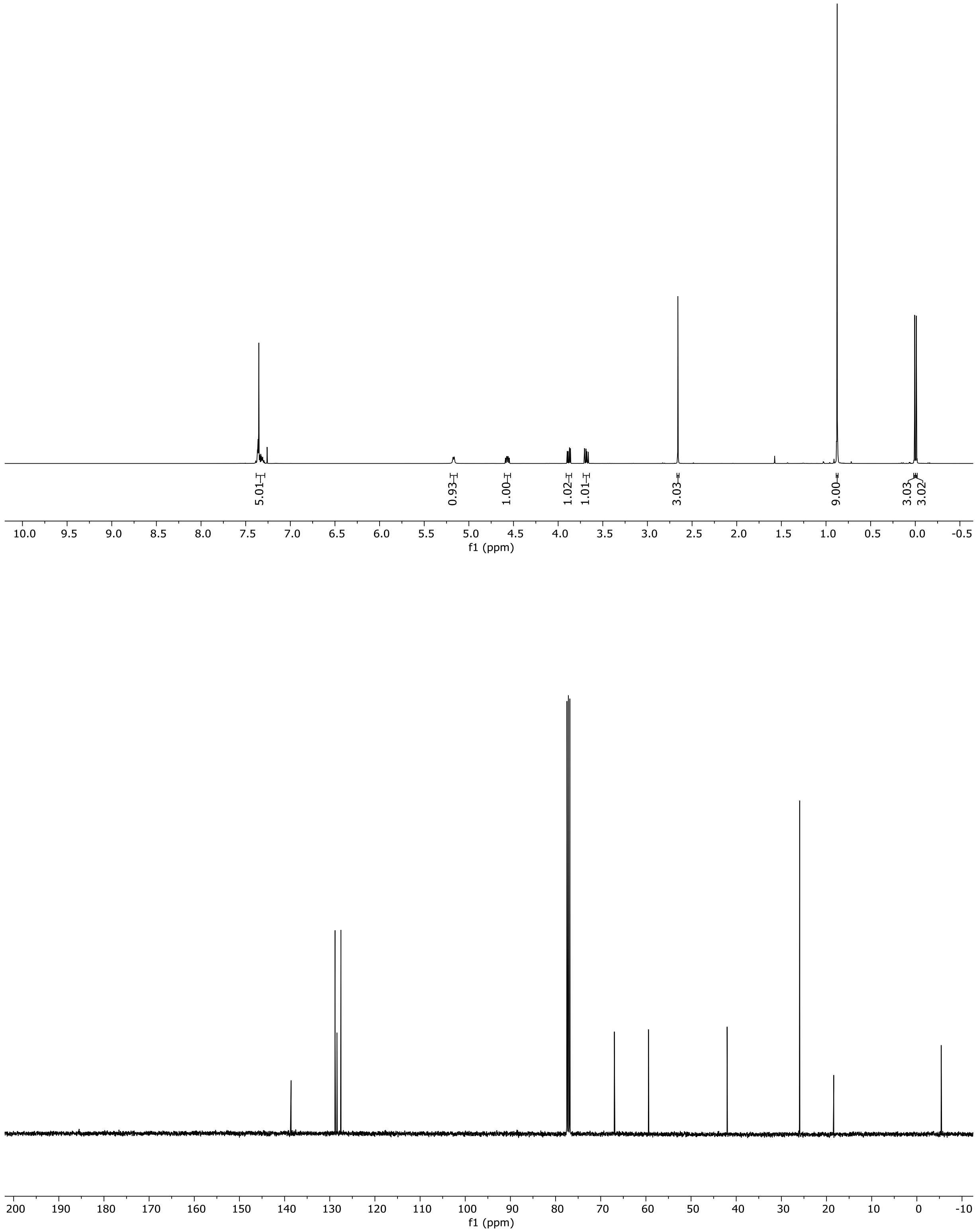

Supplementary Fig 16. ^{1}H (top) and ^{13}C (bottom) NMR spectra of compound **SP**.

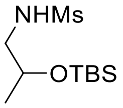

SQ

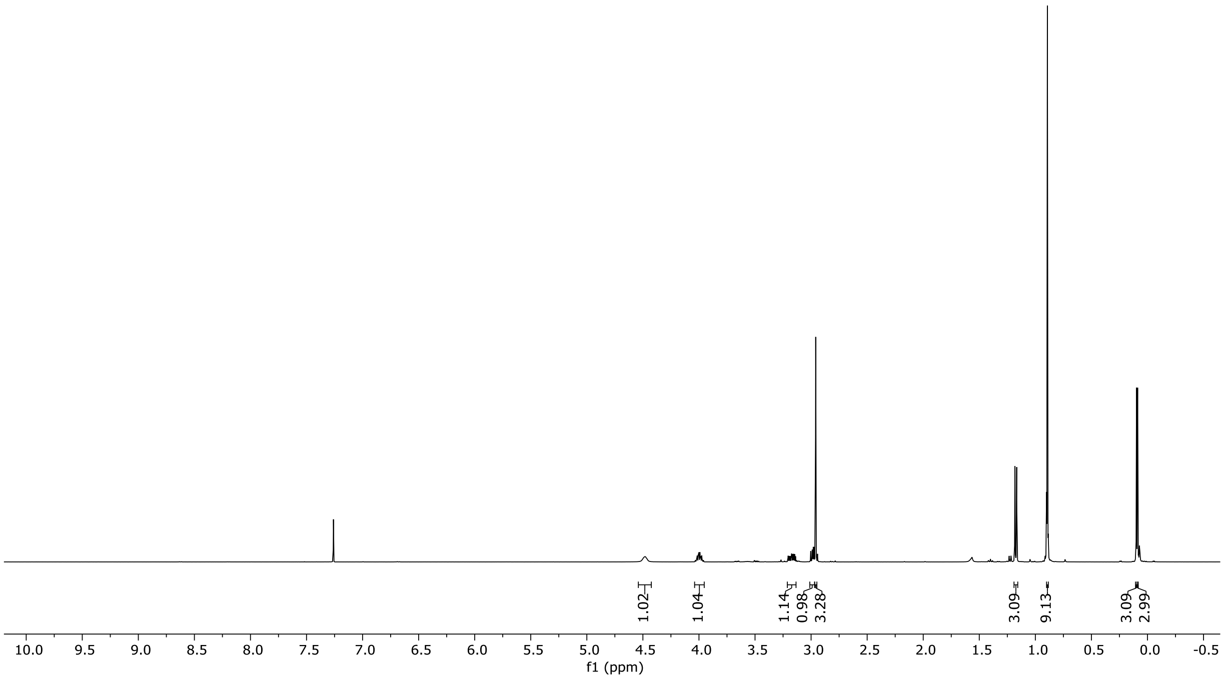

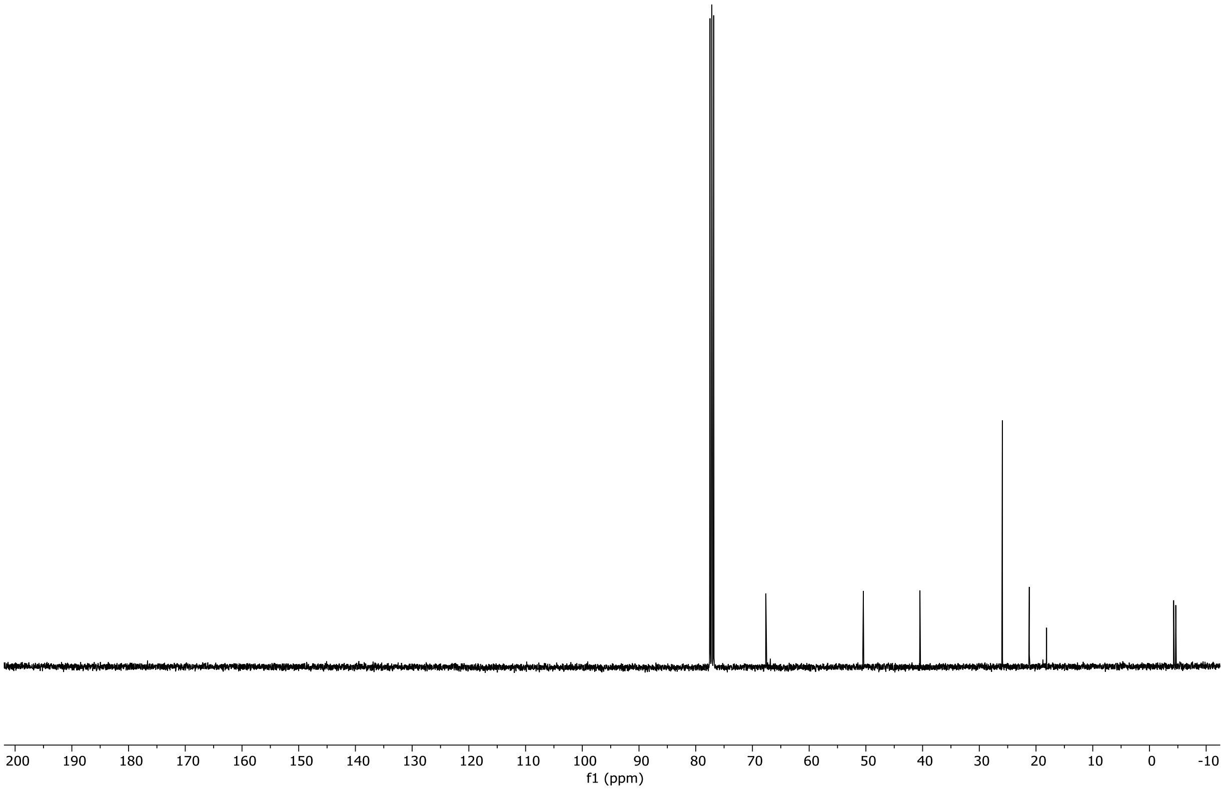

Supplementary Fig 17. ^{1}H (top) and ^{13}C (bottom) NMR spectra of compound **SQ**.

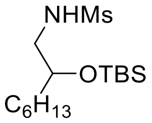

SR

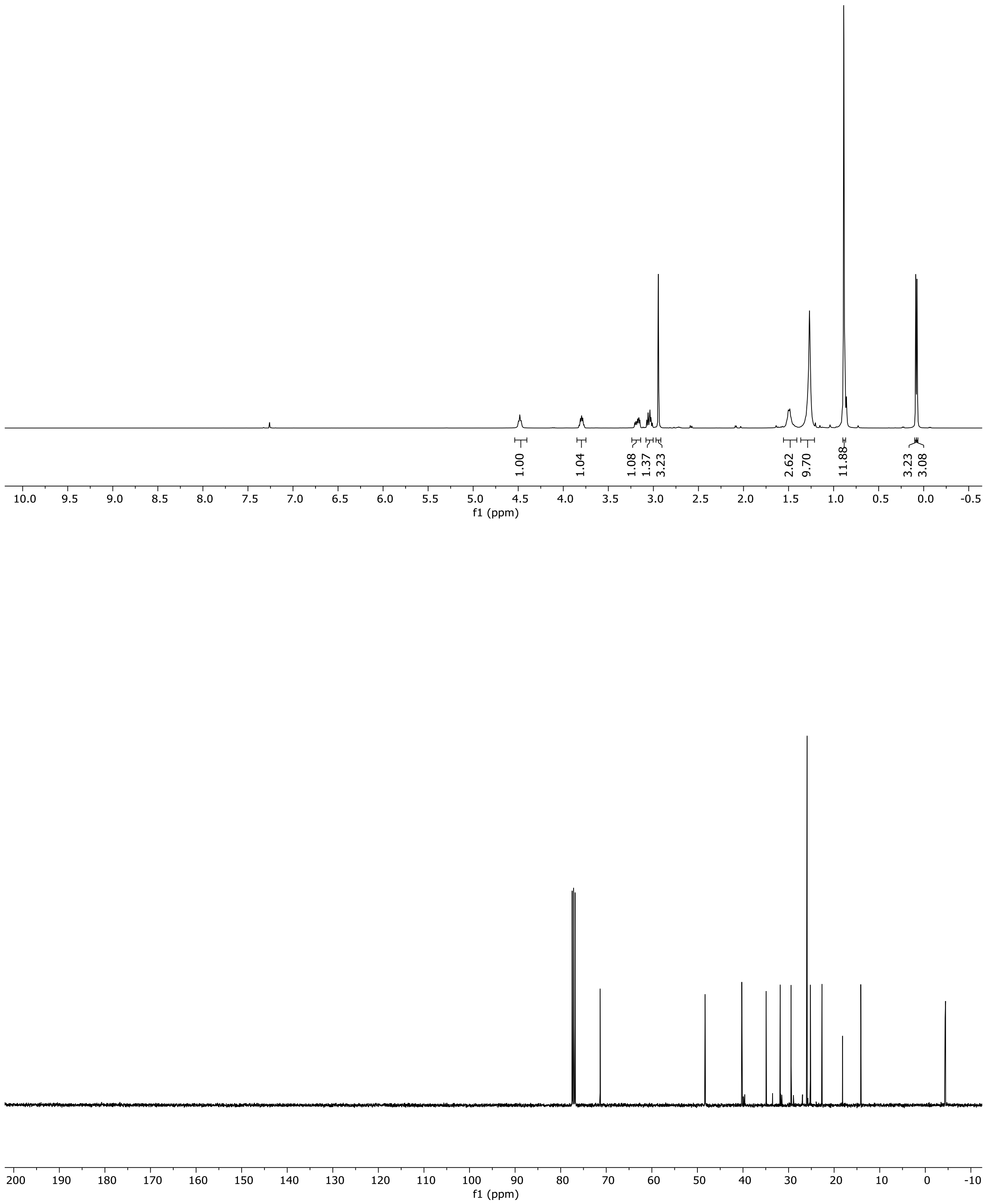

Supplementary Fig 18. ^{1}H (top) and ^{13}C (bottom) NMR spectra of compound **SR**.

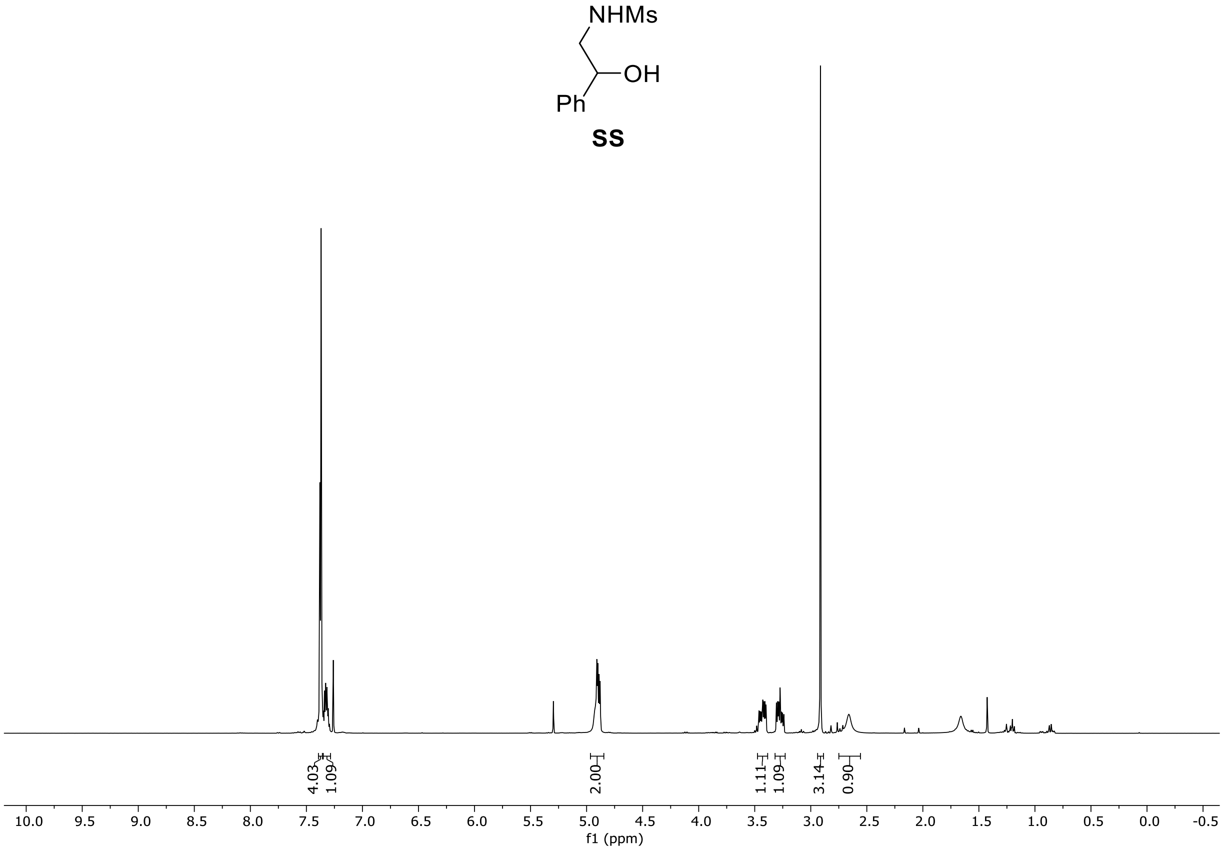

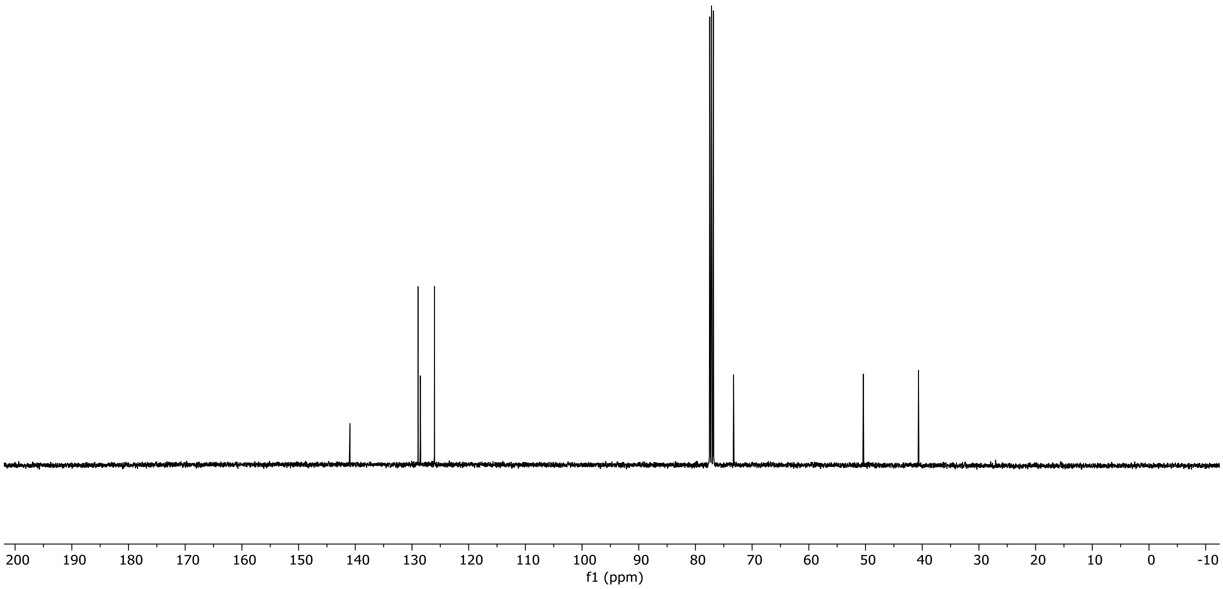

Supplementary Fig 19. ^{1}H (top) and ^{13}C (bottom) NMR spectra of compound **SS**.

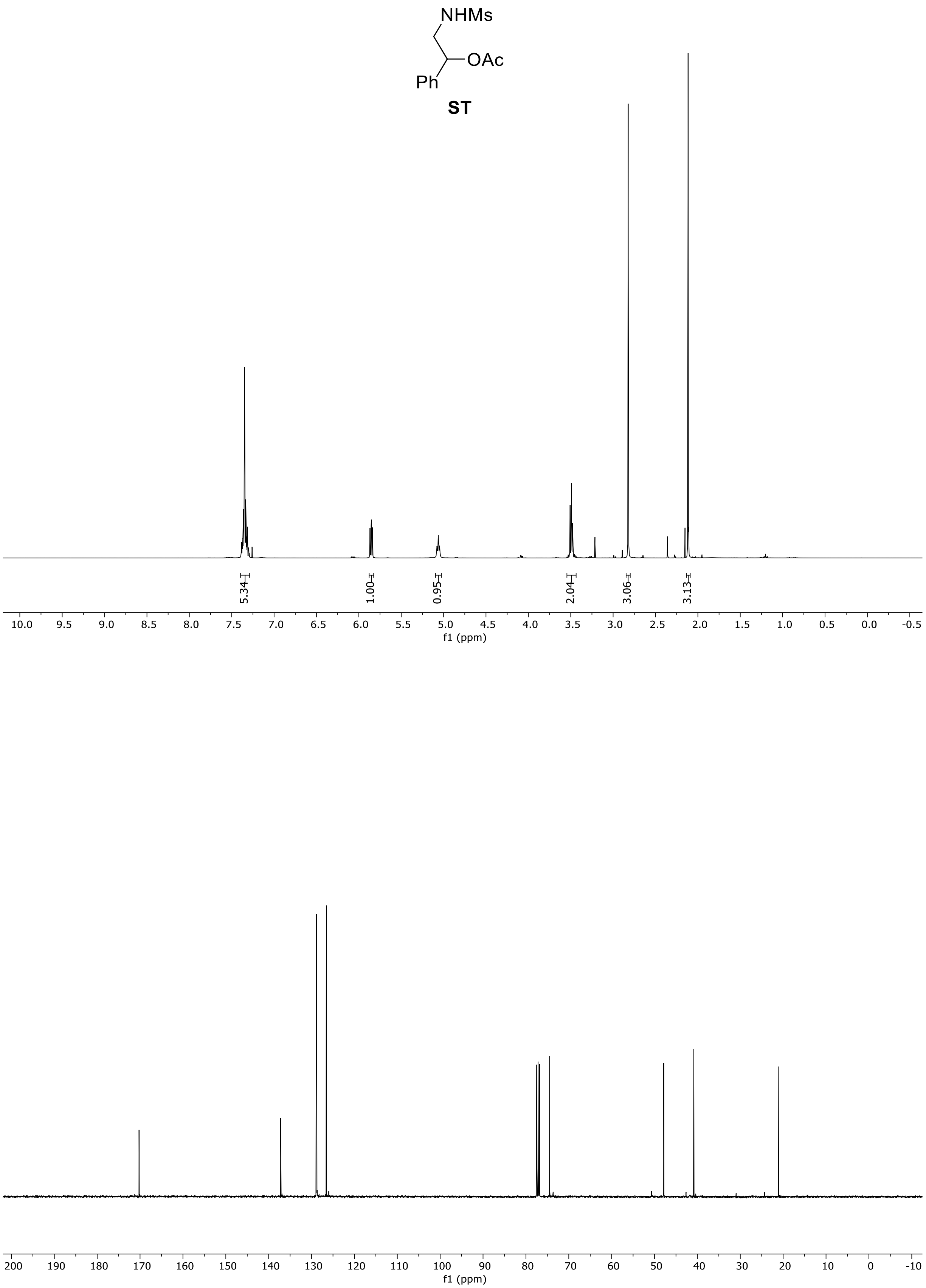

Supplementary Fig 20. ^{1}H (top) and ^{13}C (bottom) NMR spectra of compound **ST**.

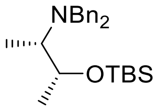

SU

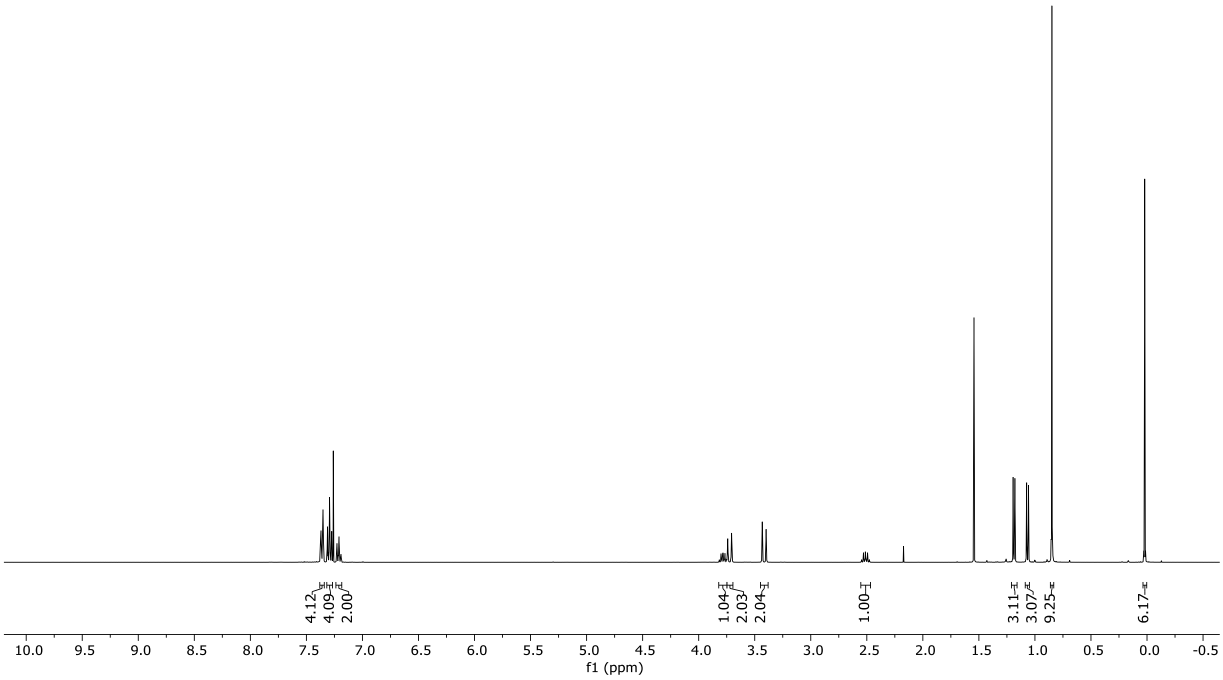

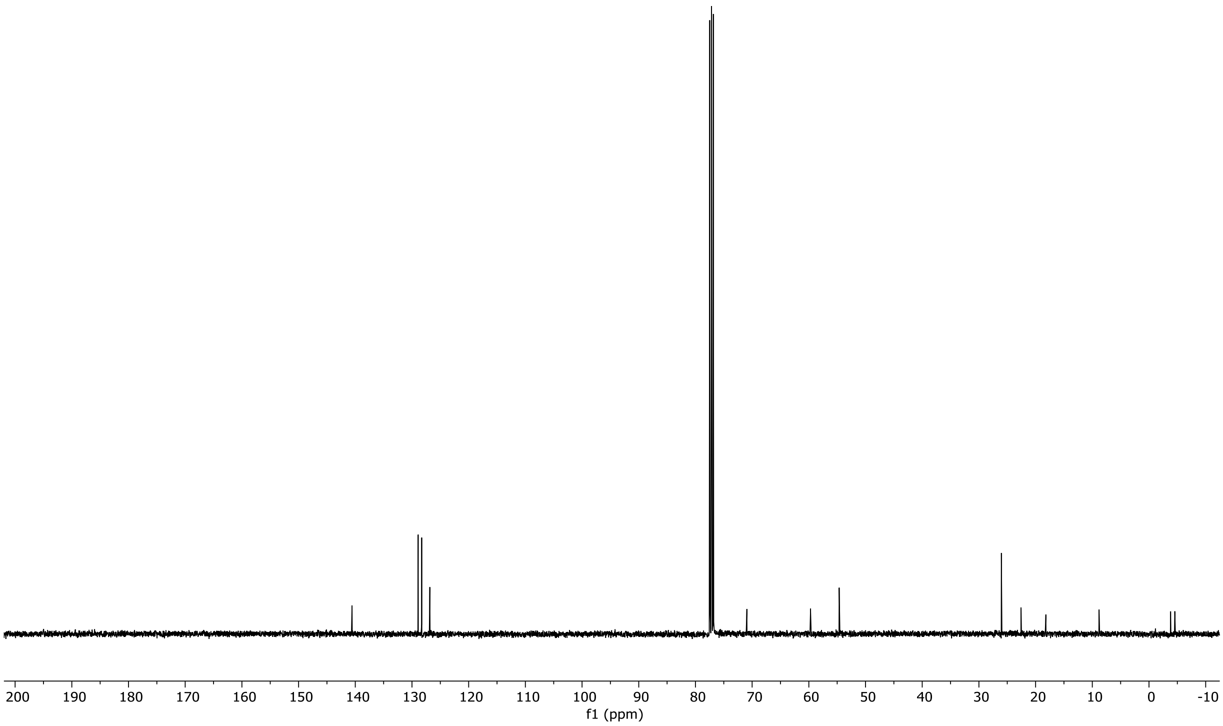

Supplementary Fig 21. ^{1}H (top) and ^{13}C (bottom) NMR spectra of compound **SU**.

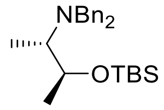

SV

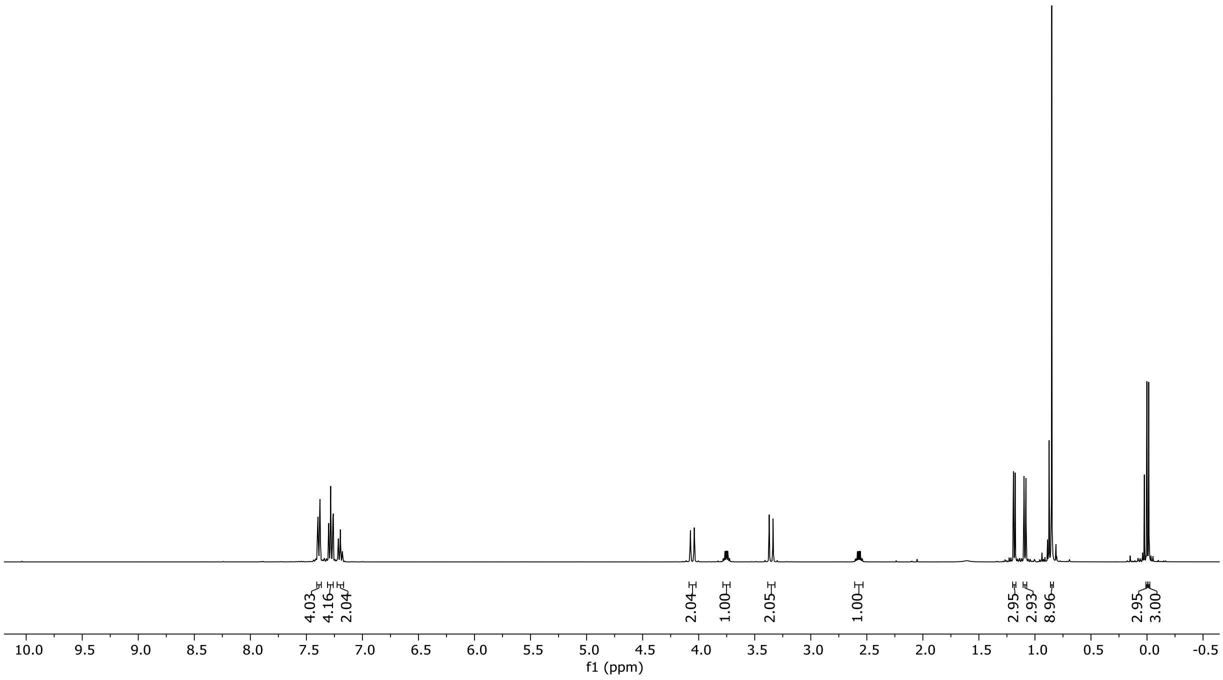

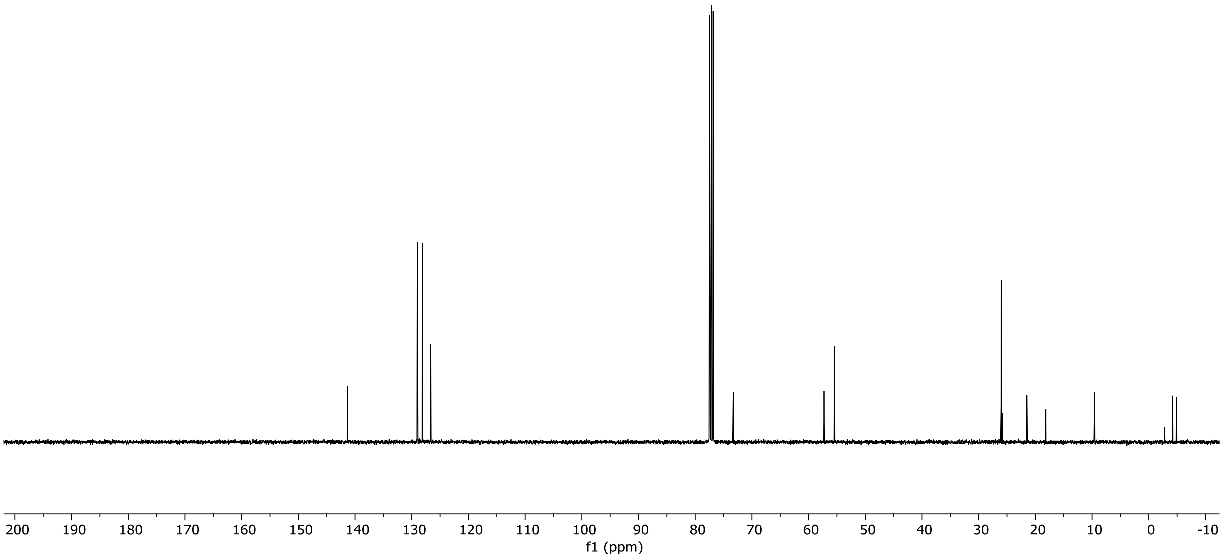

Supplementary Fig 22. ^{1}H (top) and ^{13}C (bottom) NMR spectra of compound **SV**.

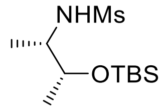

SW

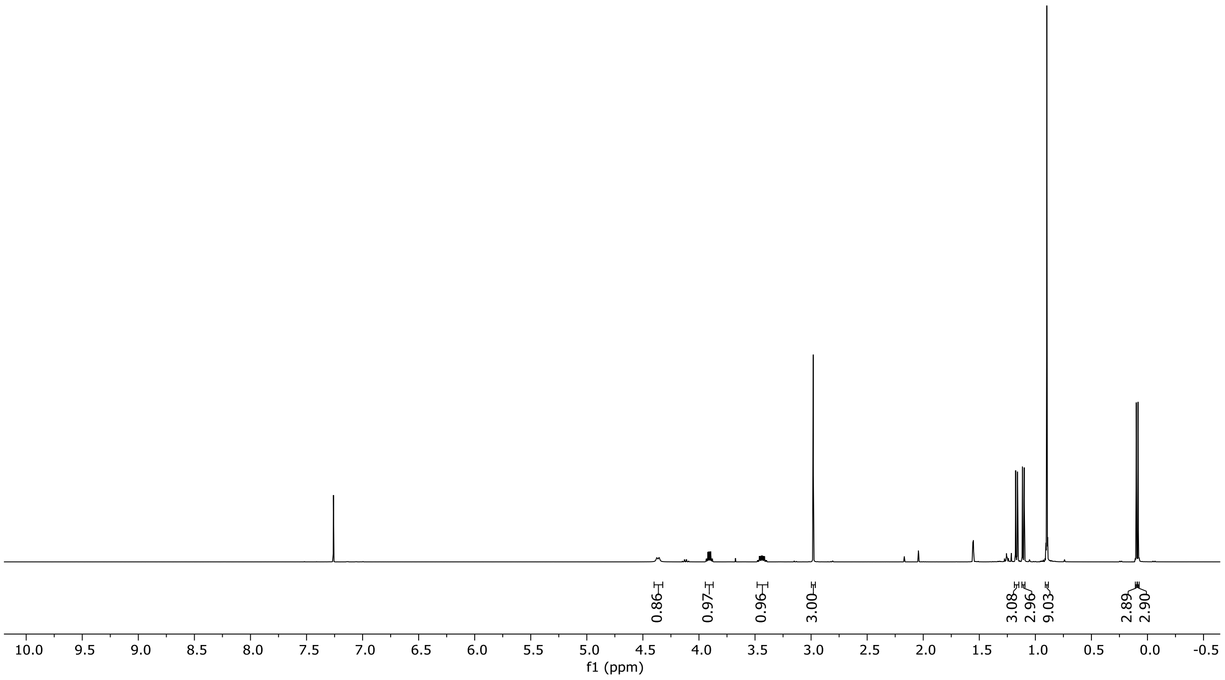

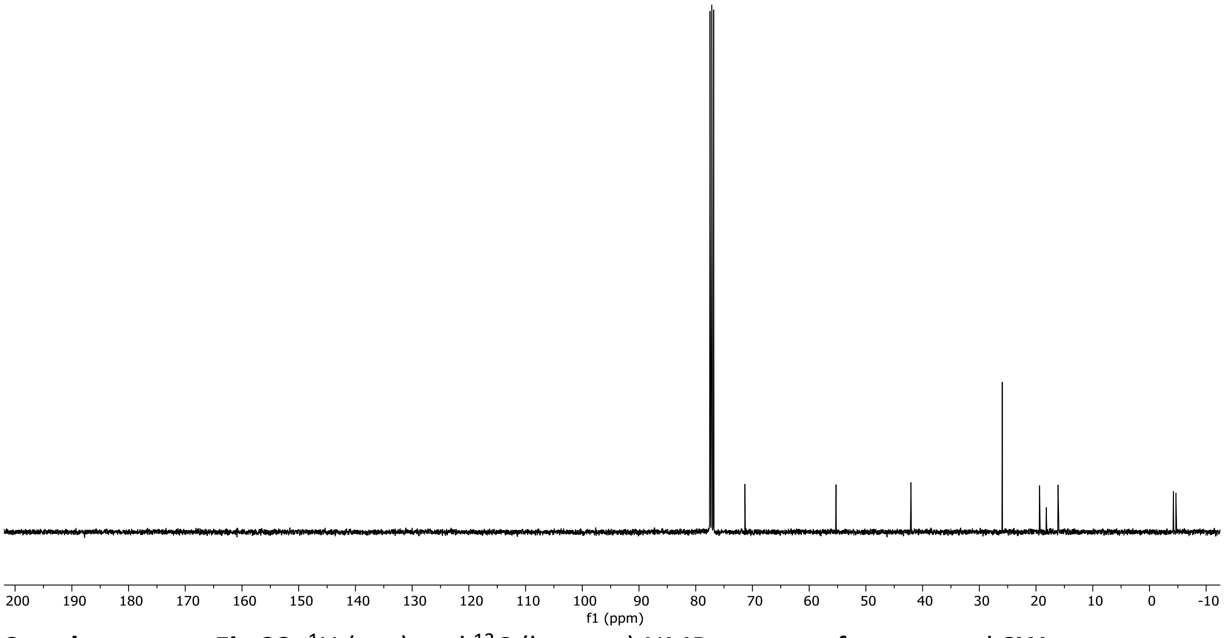

Supplementary Fig 23. ^{1}H (top) and ^{13}C (bottom) NMR spectra of compound **SW**.

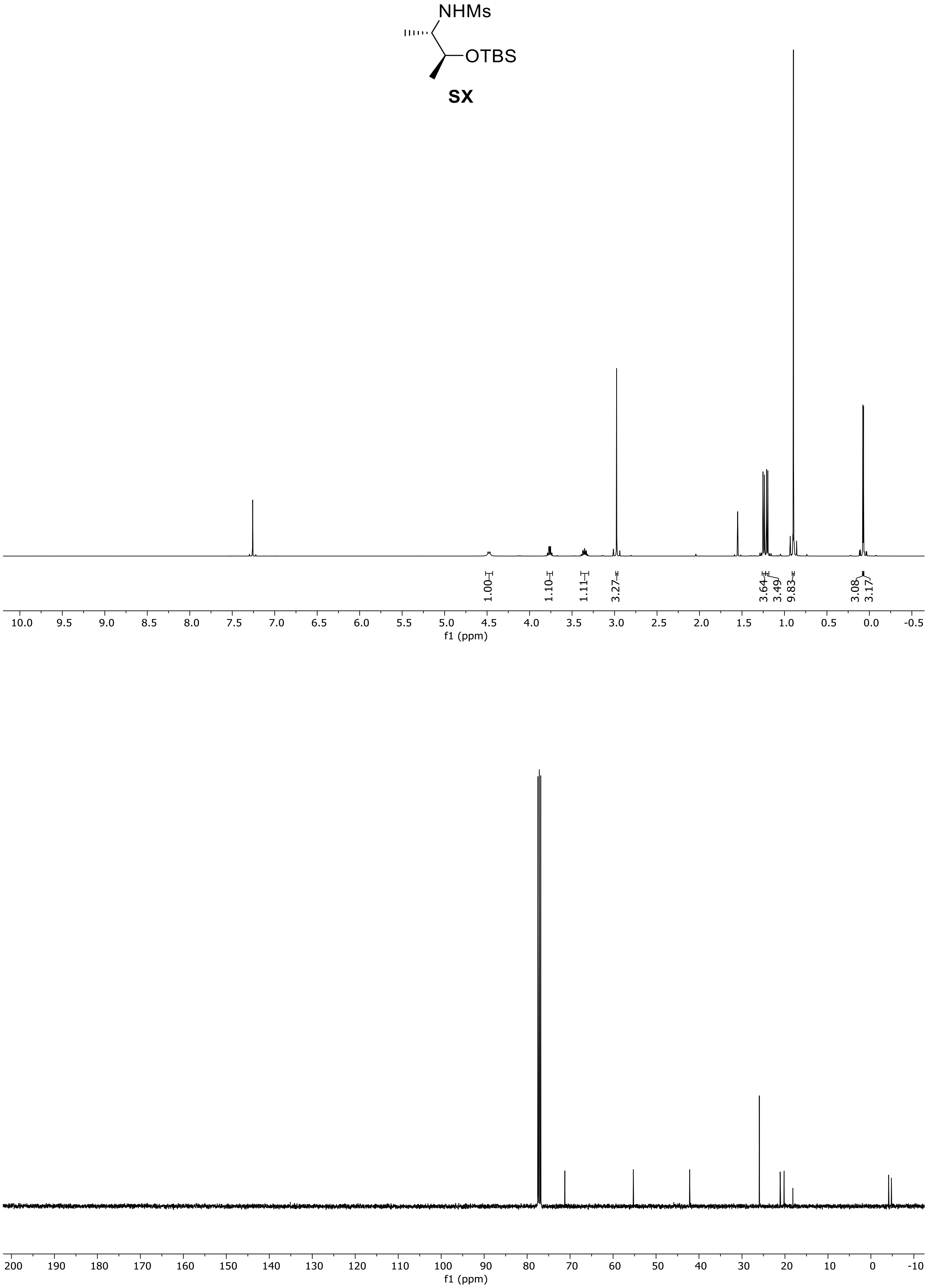

Supplementary Fig 24. ^{1}H (top) and ^{13}C (bottom) NMR spectra of compound **SX**.

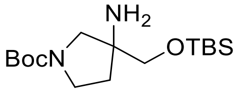

SY

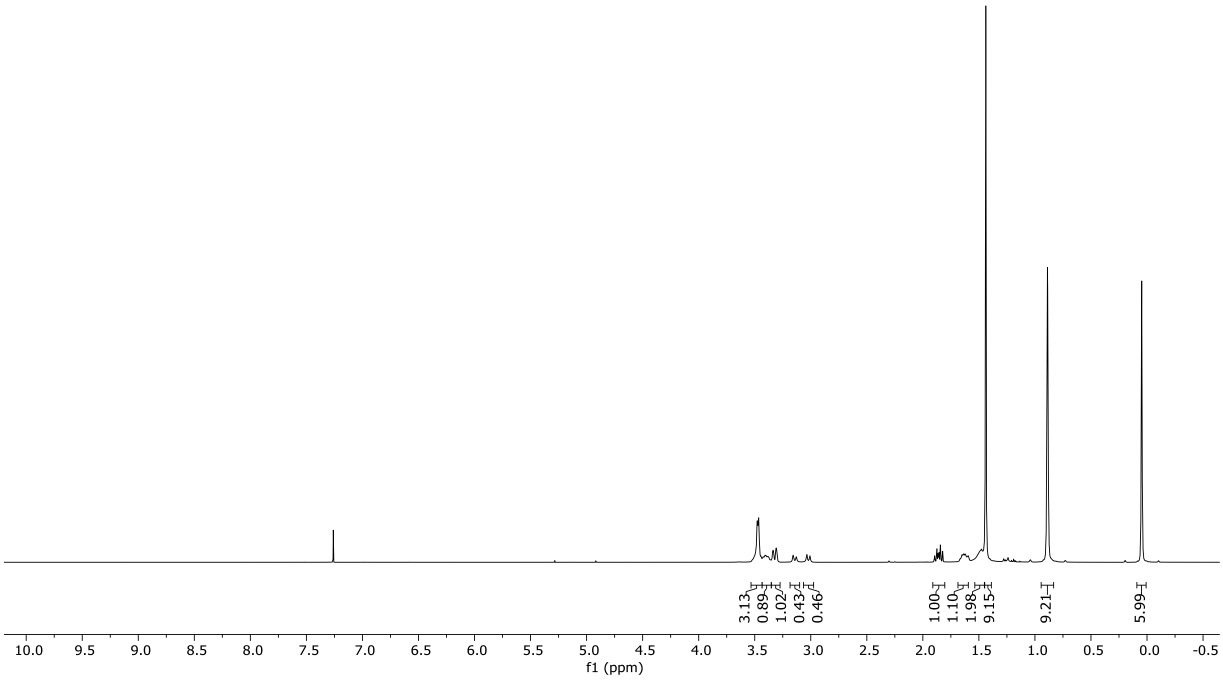

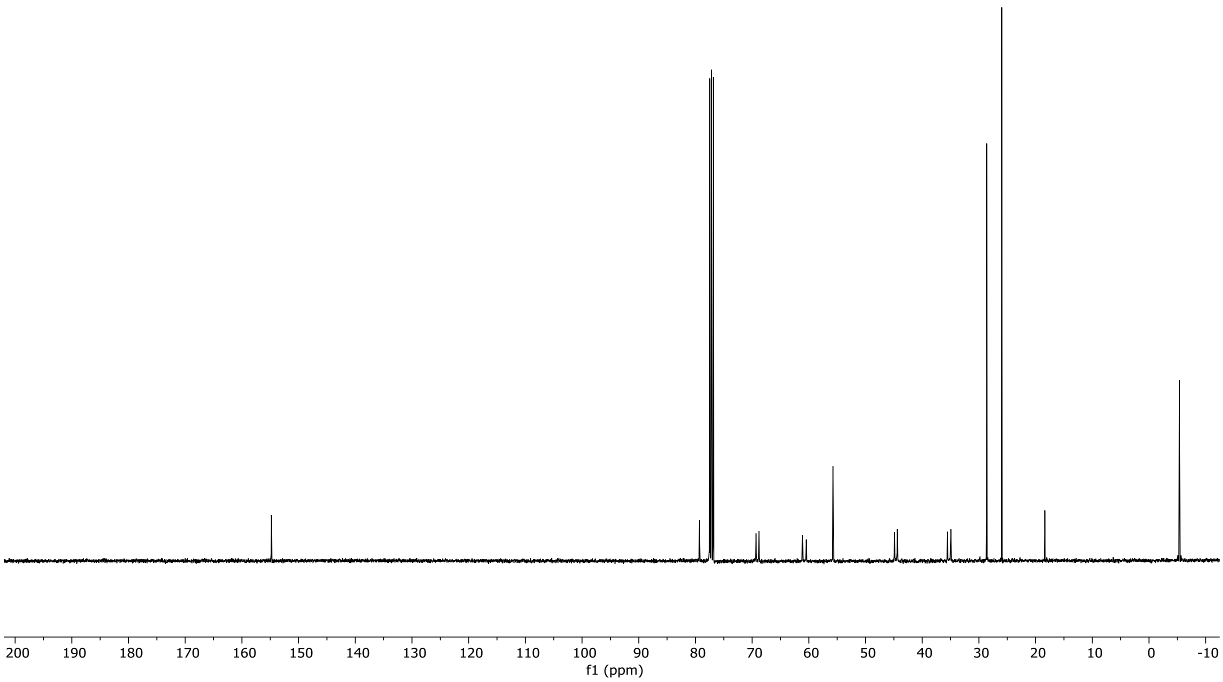

Supplementary Fig 25. ^{1}H (top) and ^{13}C (bottom) NMR spectra of compound **SY**.

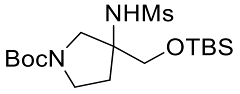

SZ

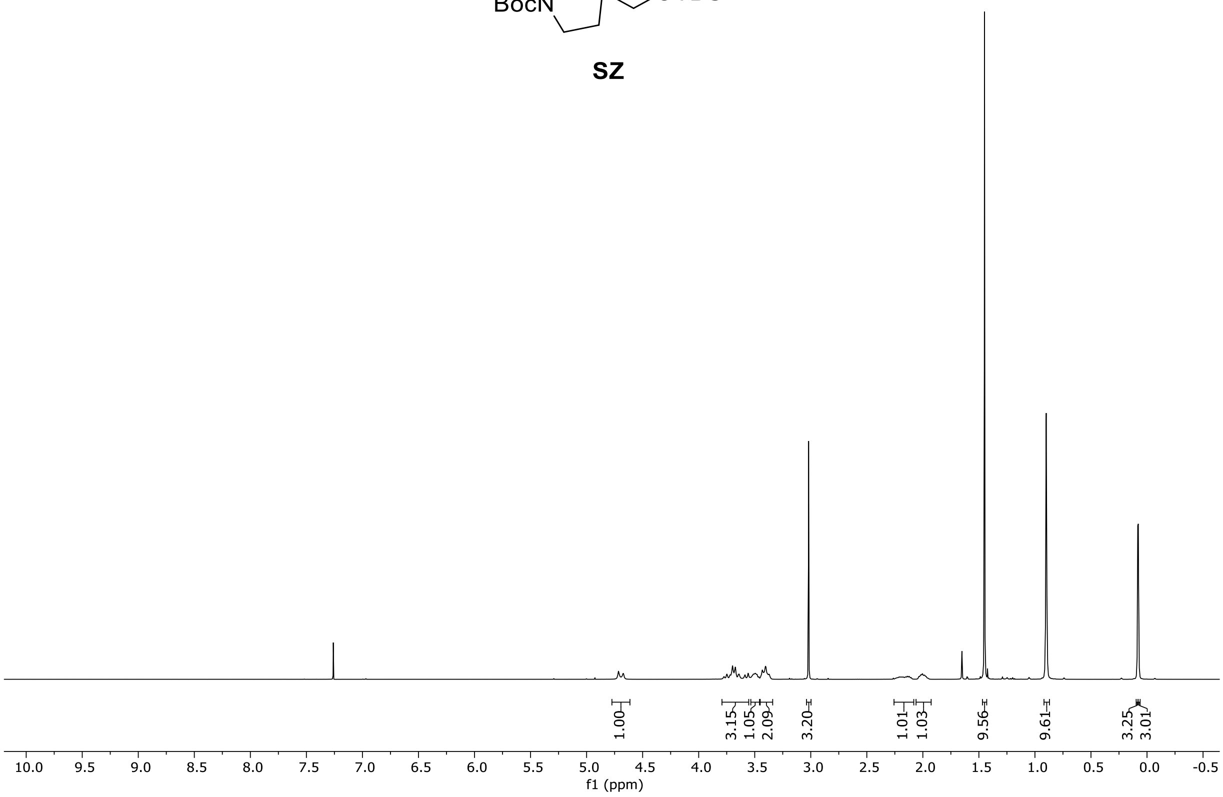

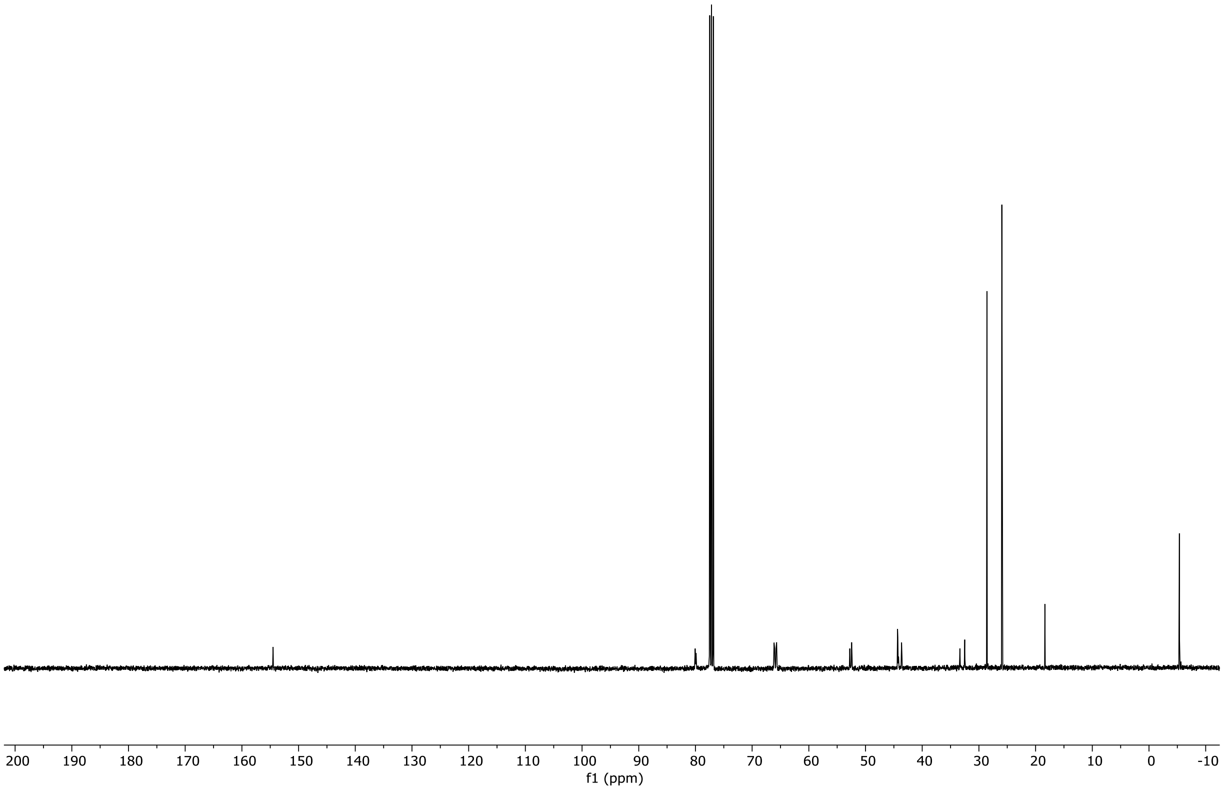

Supplementary Fig 26. ^{1}H (top) and ^{13}C (bottom) NMR spectra of compound **SZ**.

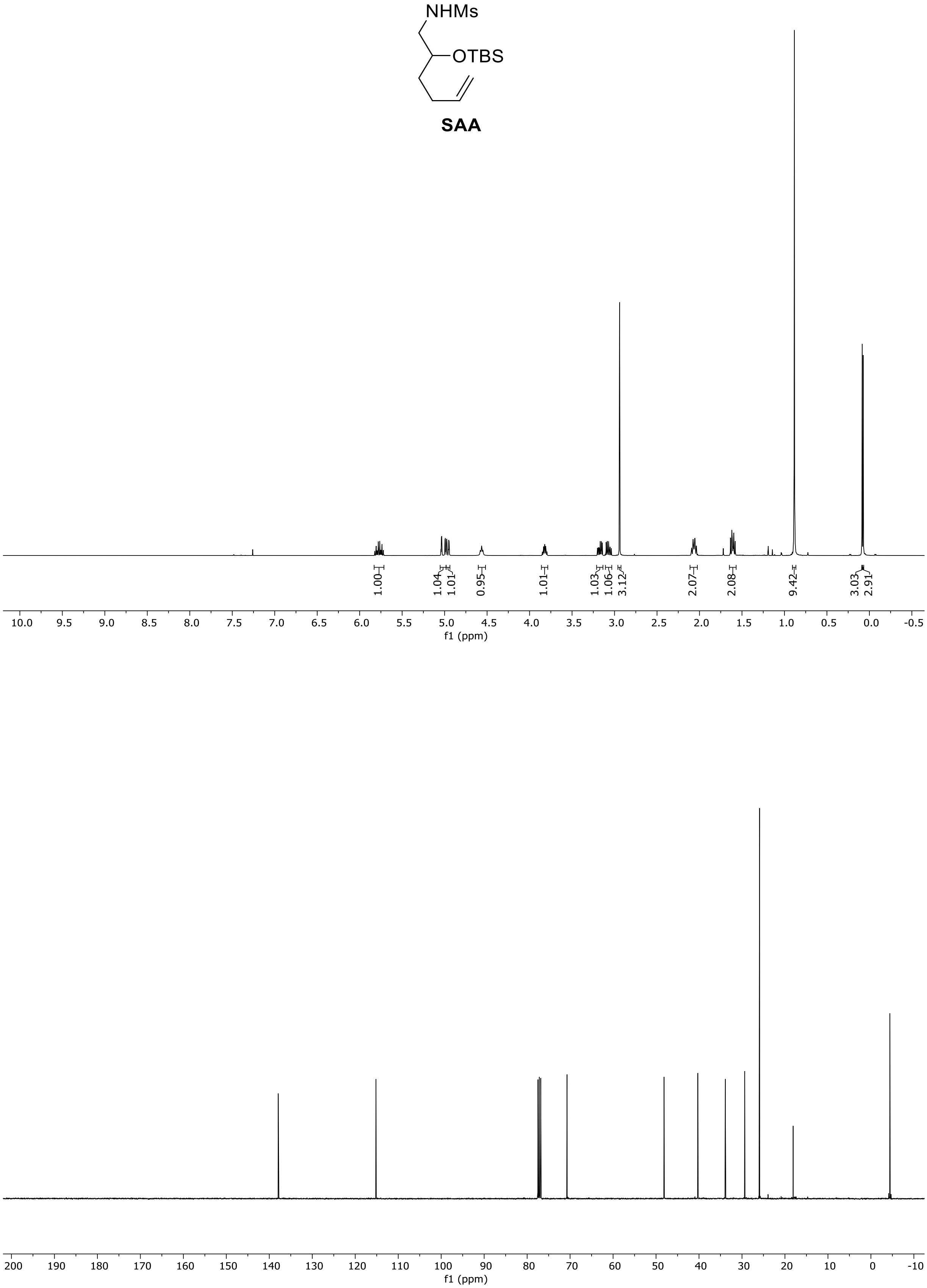

Supplementary Fig 27. ^{1}H (top) and ^{13}C (bottom) NMR spectra of compound **SAA**.

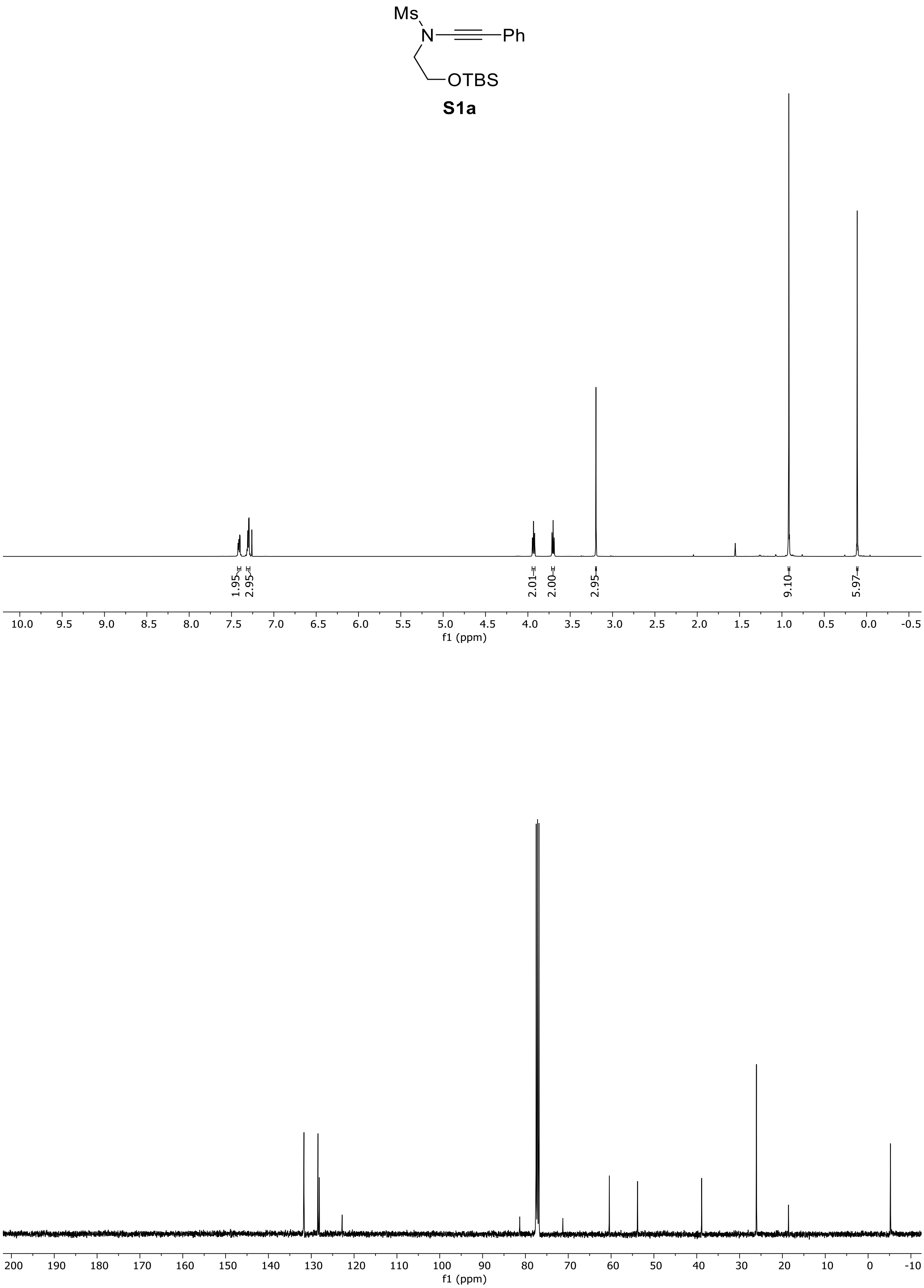

Supplementary Fig 28. ^{1}H (top) and ^{13}C (bottom) NMR spectra of compound **S1a**.

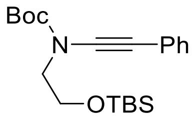

S1b

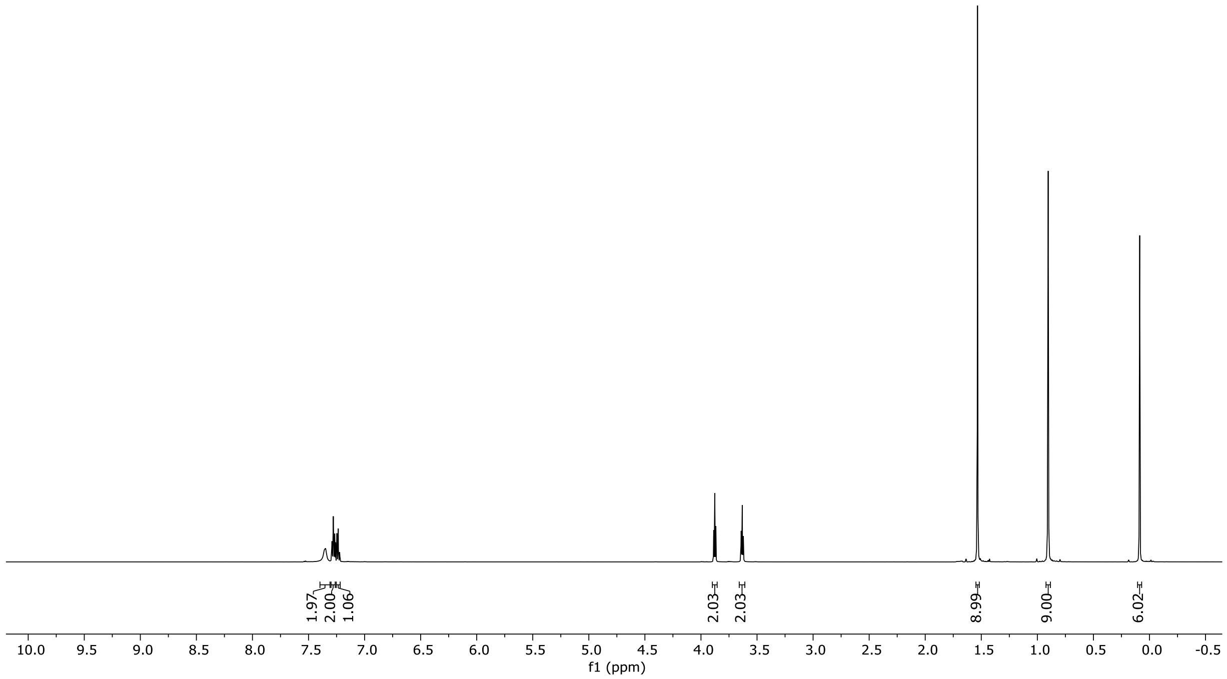

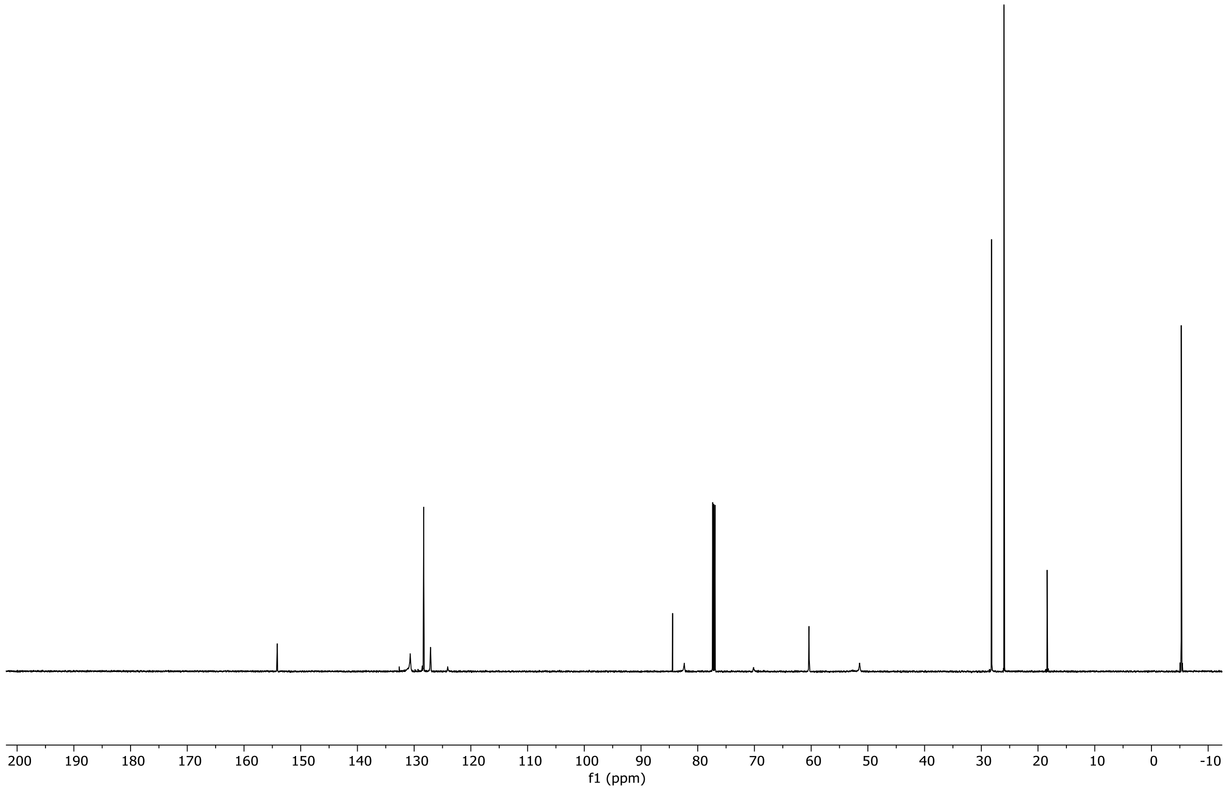

Supplementary Fig 29. ^{1}H (top) and ^{13}C (bottom) NMR spectra of compound **S1b**.

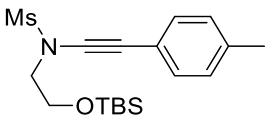

S1c

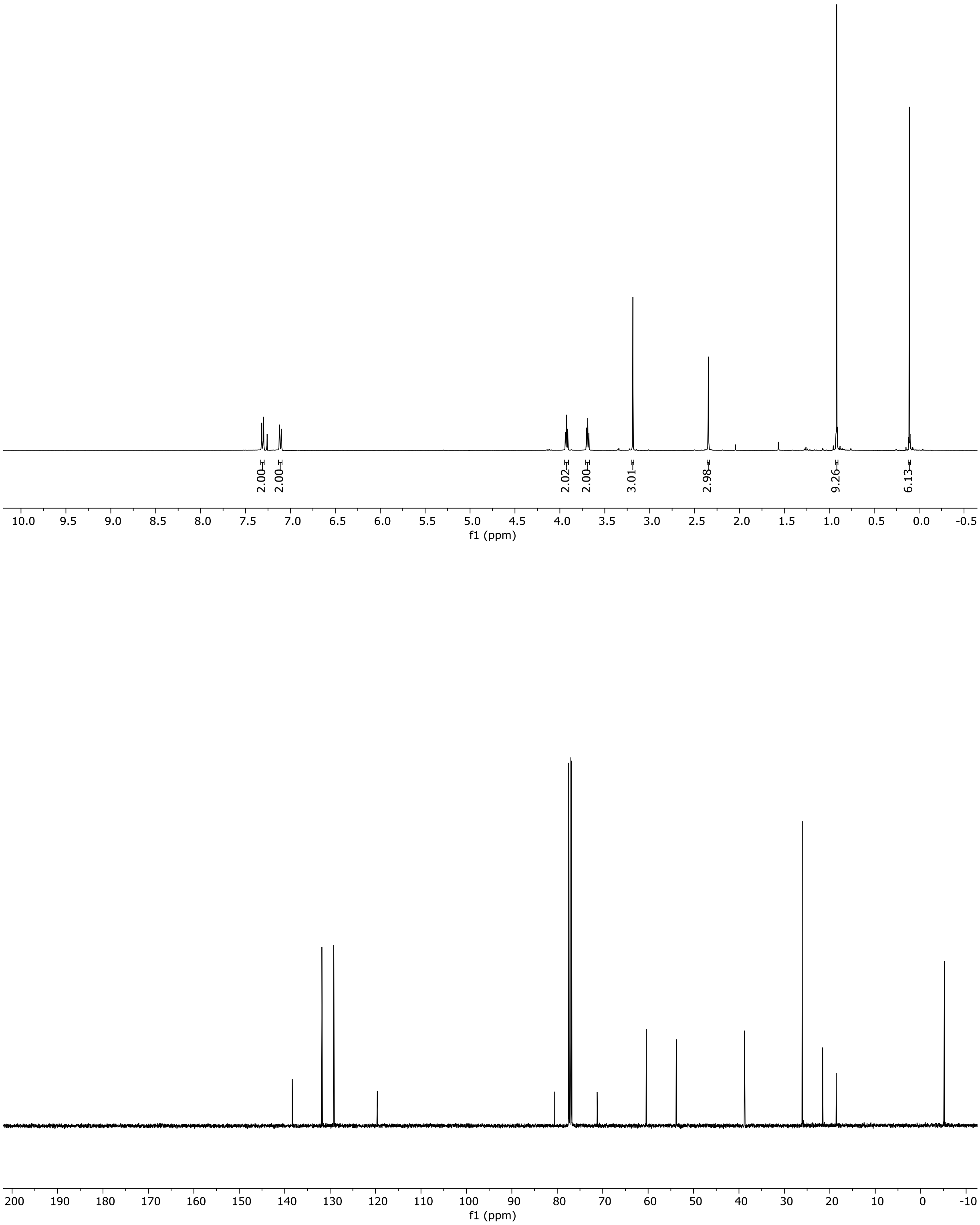

Supplementary Fig 30. ^{1}H (top) and ^{13}C (bottom) NMR spectra of compound **S1c**.

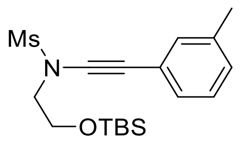

S1d

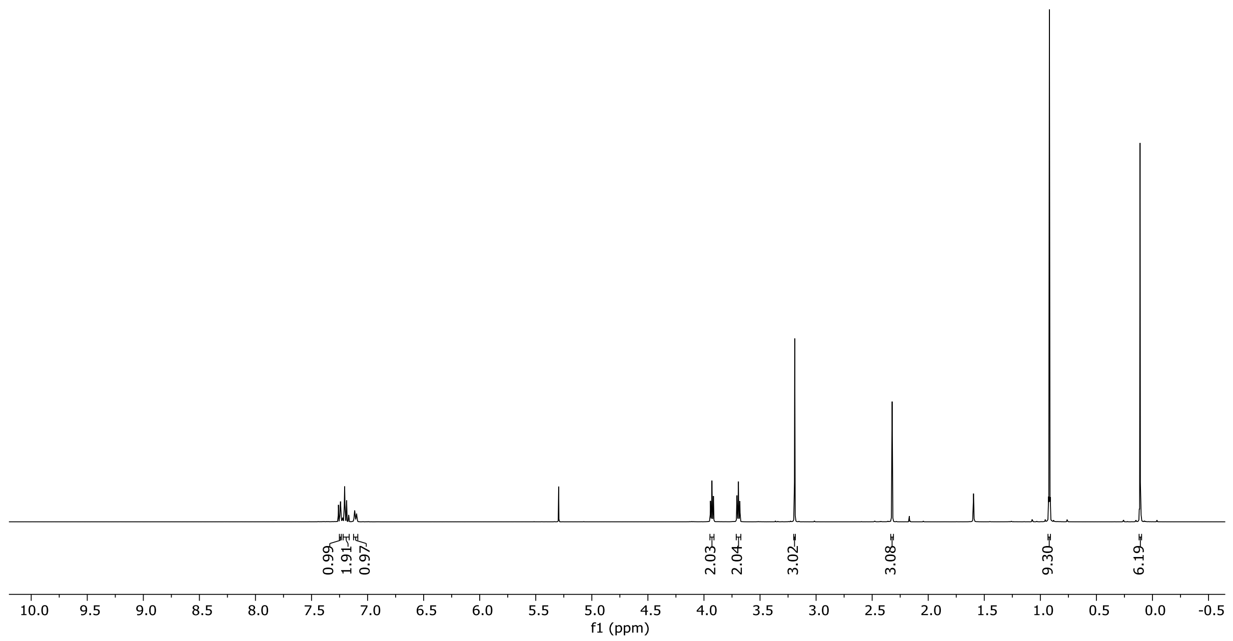

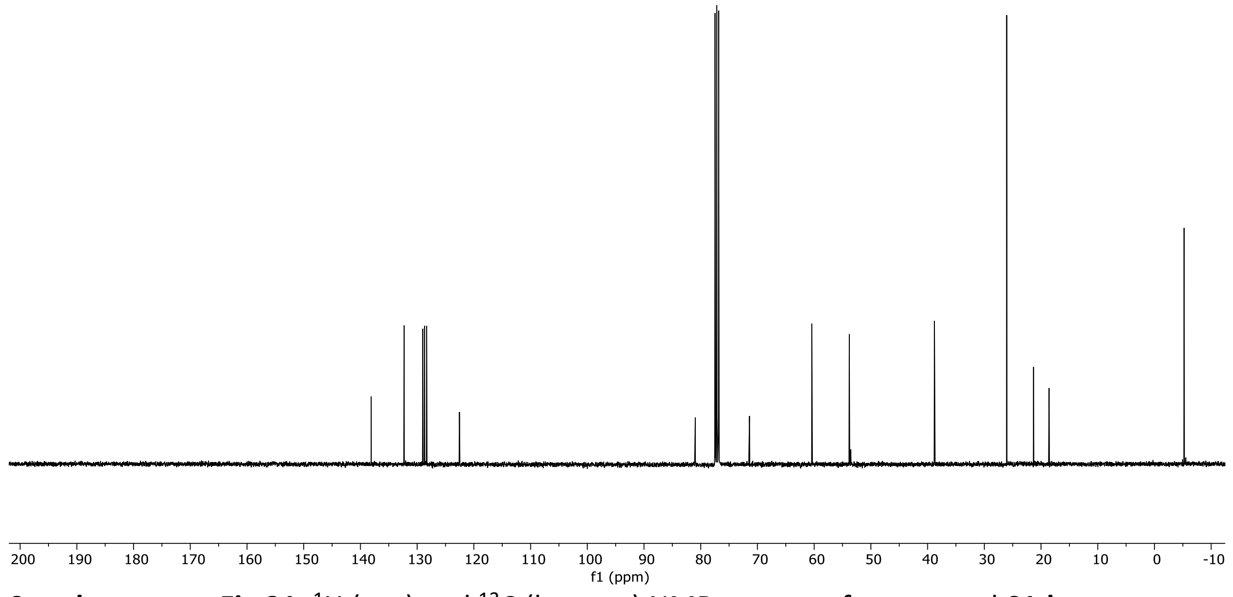

Supplementary Fig 31. ^{1}H (top) and ^{13}C (bottom) NMR spectra of compound **S1d**.

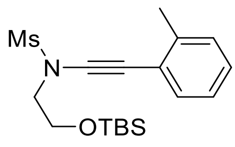

S1e

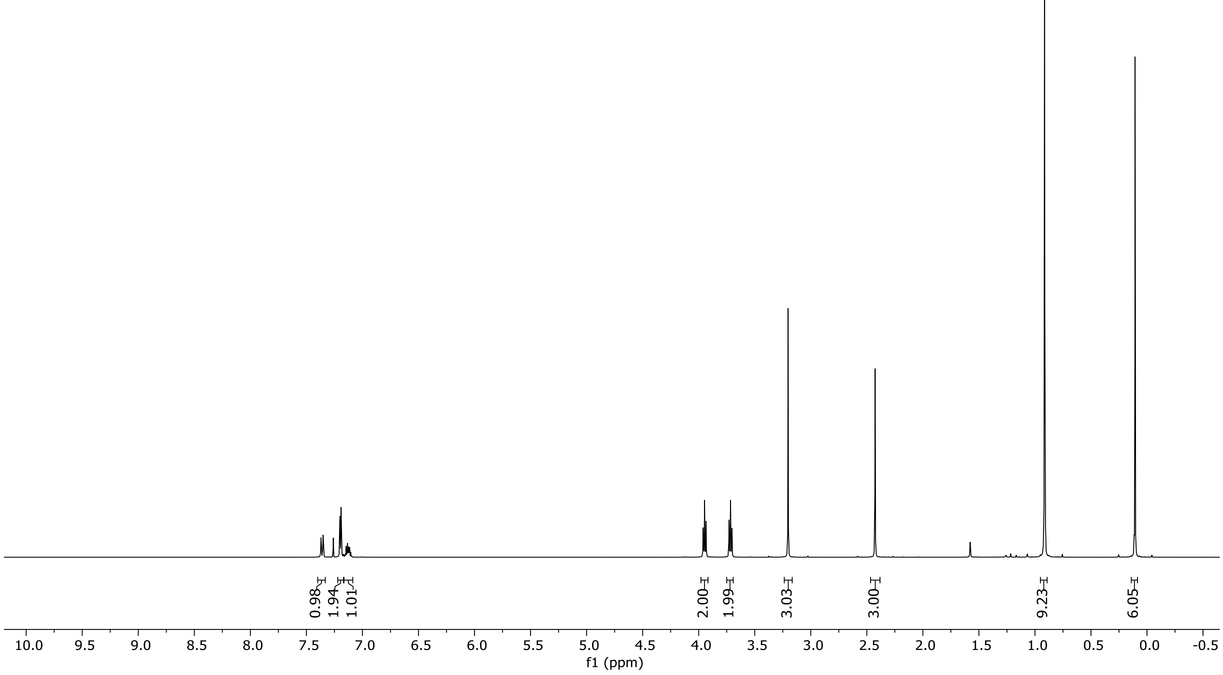

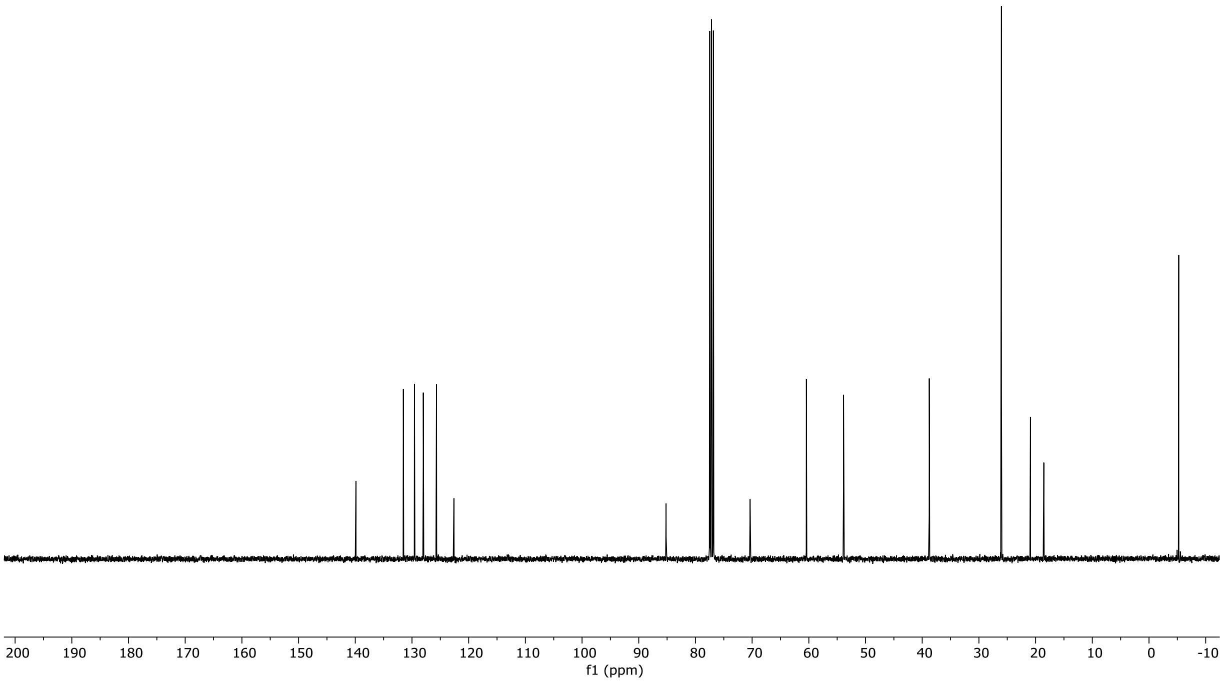

Supplementary Fig 32. ^{1}H (top) and ^{13}C (bottom) NMR spectra of compound **S1e**.

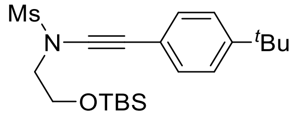

S1f

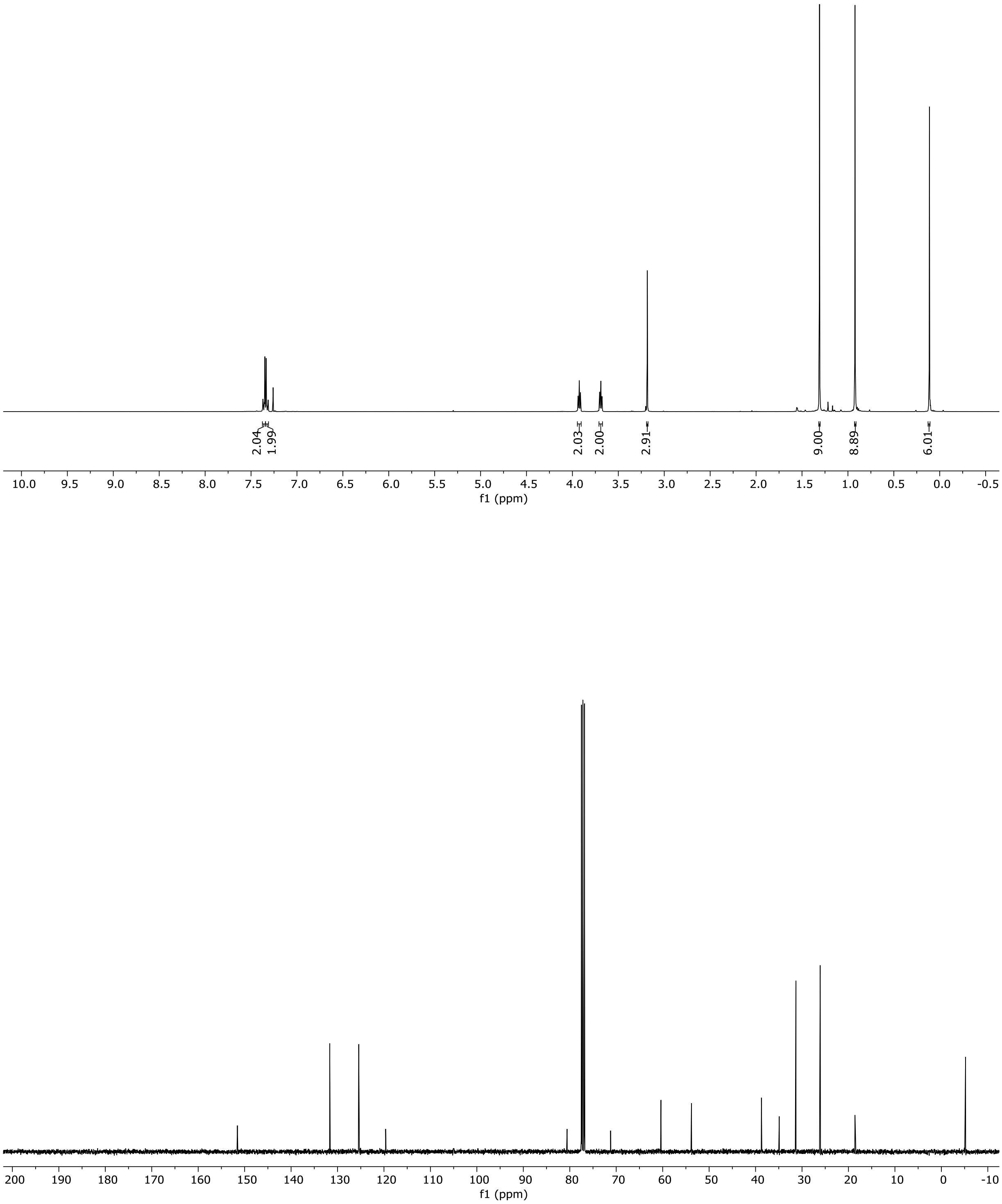

Supplementary Fig 33. ^{1}H (top) and ^{13}C (bottom) NMR spectra of compound **S1f**.

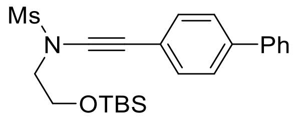

S1g

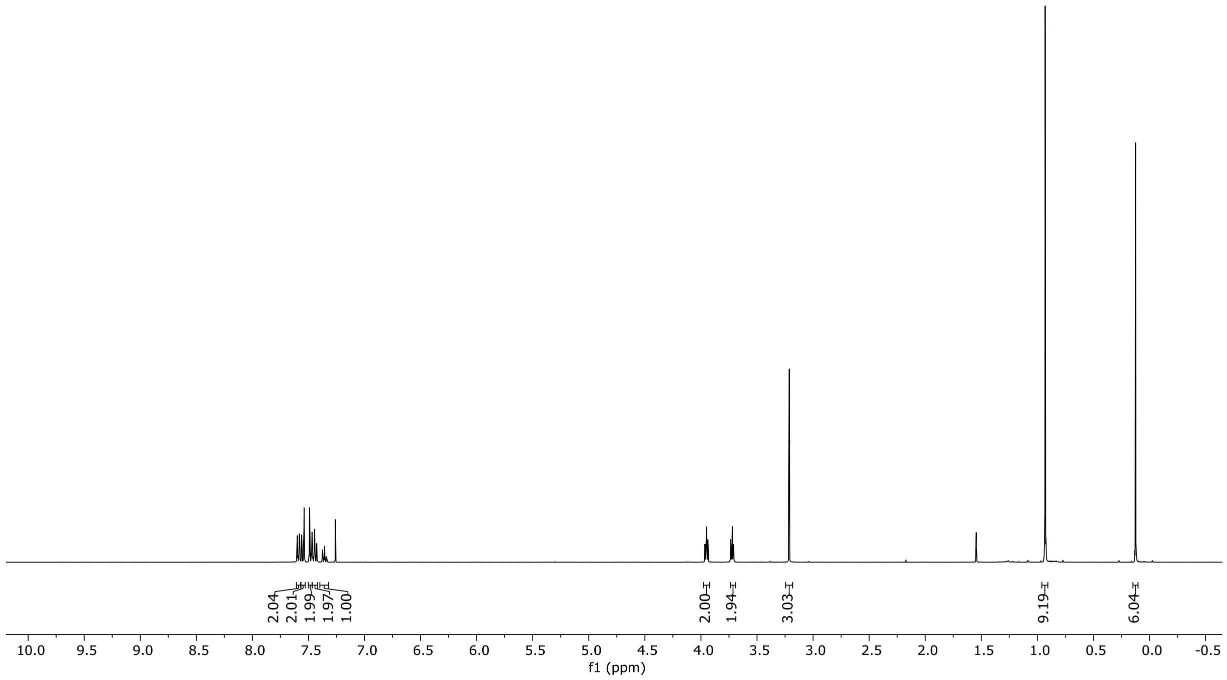

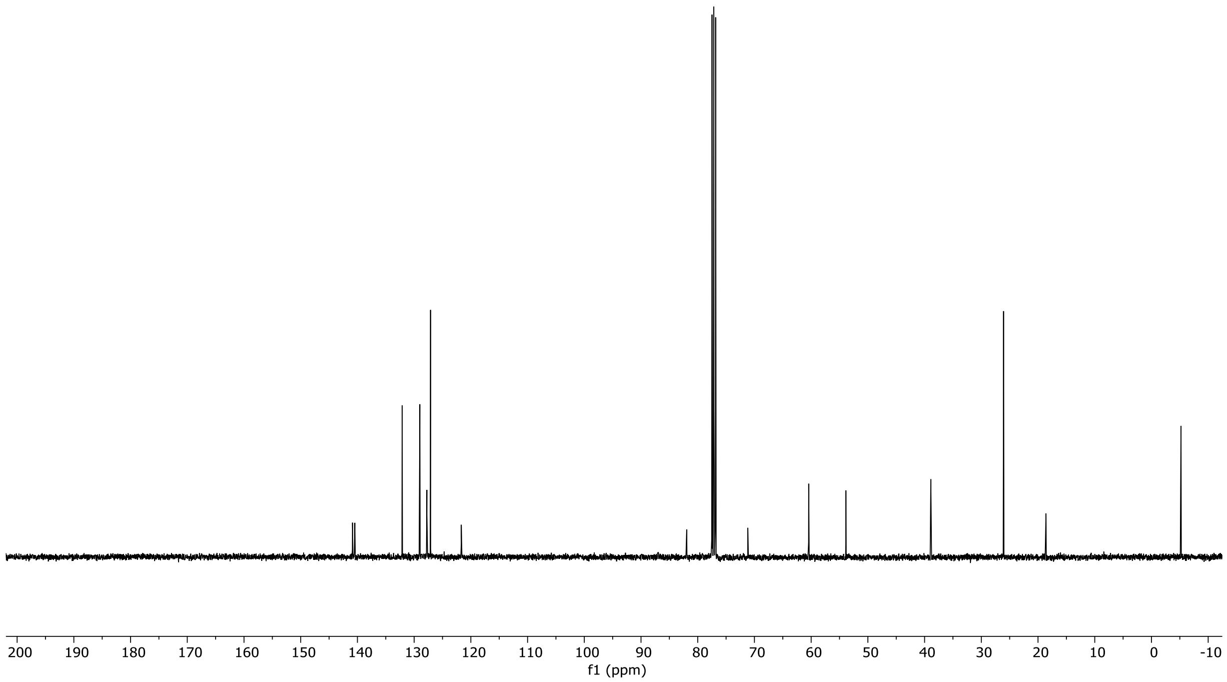

Supplementary Fig 34. ^{1}H (top) and ^{13}C (bottom) NMR spectra of compound **S1g**.

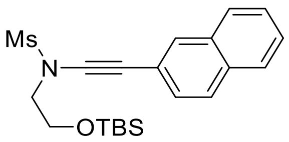

S1h

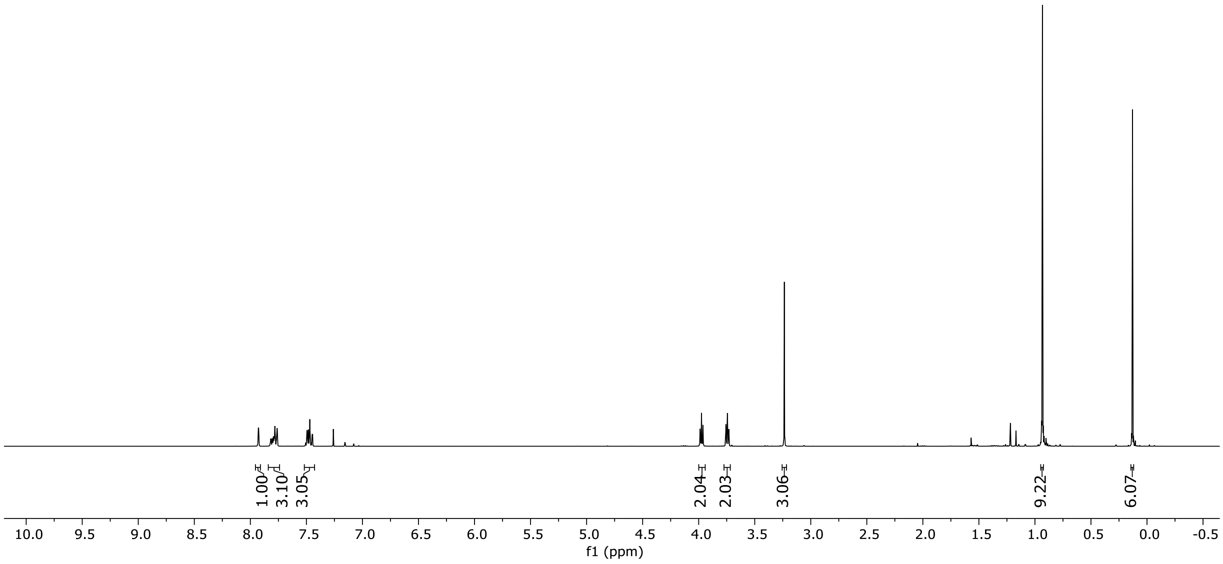

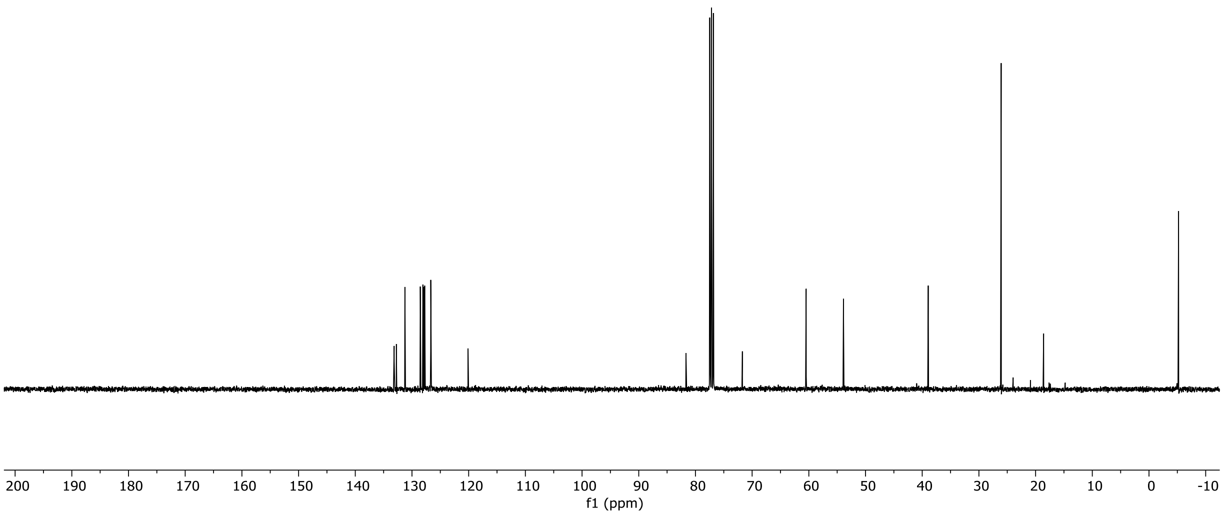

Supplementary Fig 35. ^{1}H (top) and ^{13}C (bottom) NMR spectra of compound **S1h**.

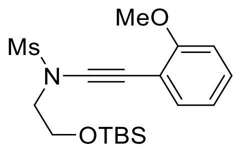

S1i

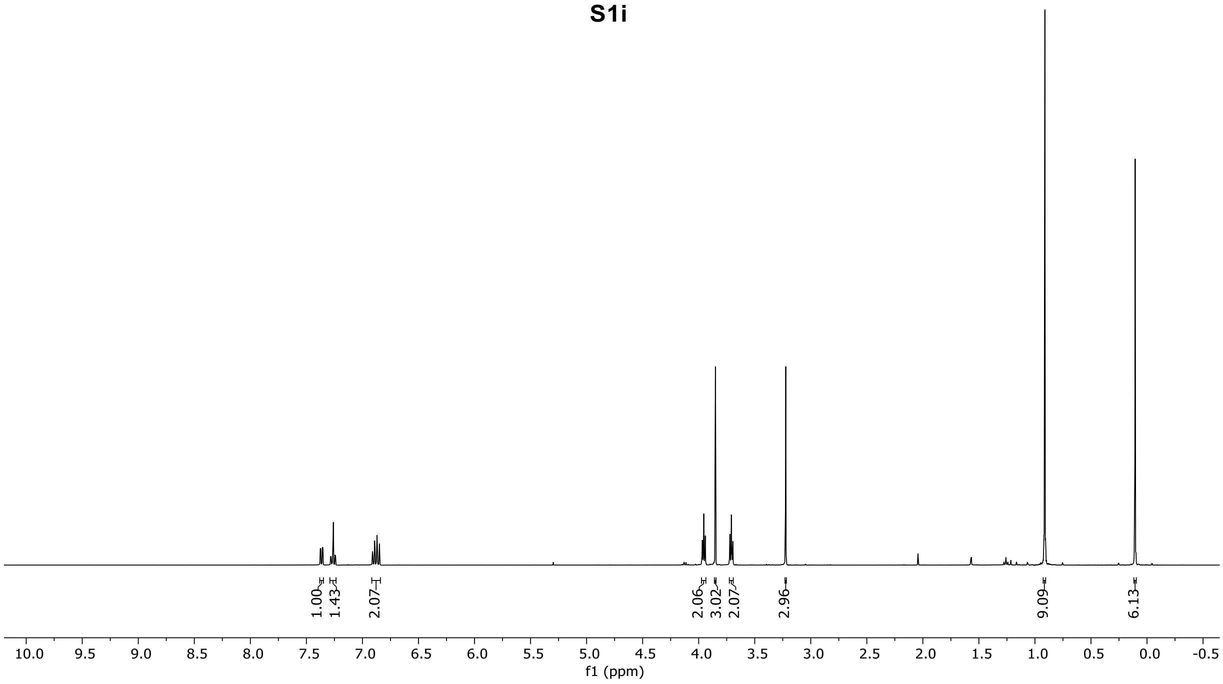

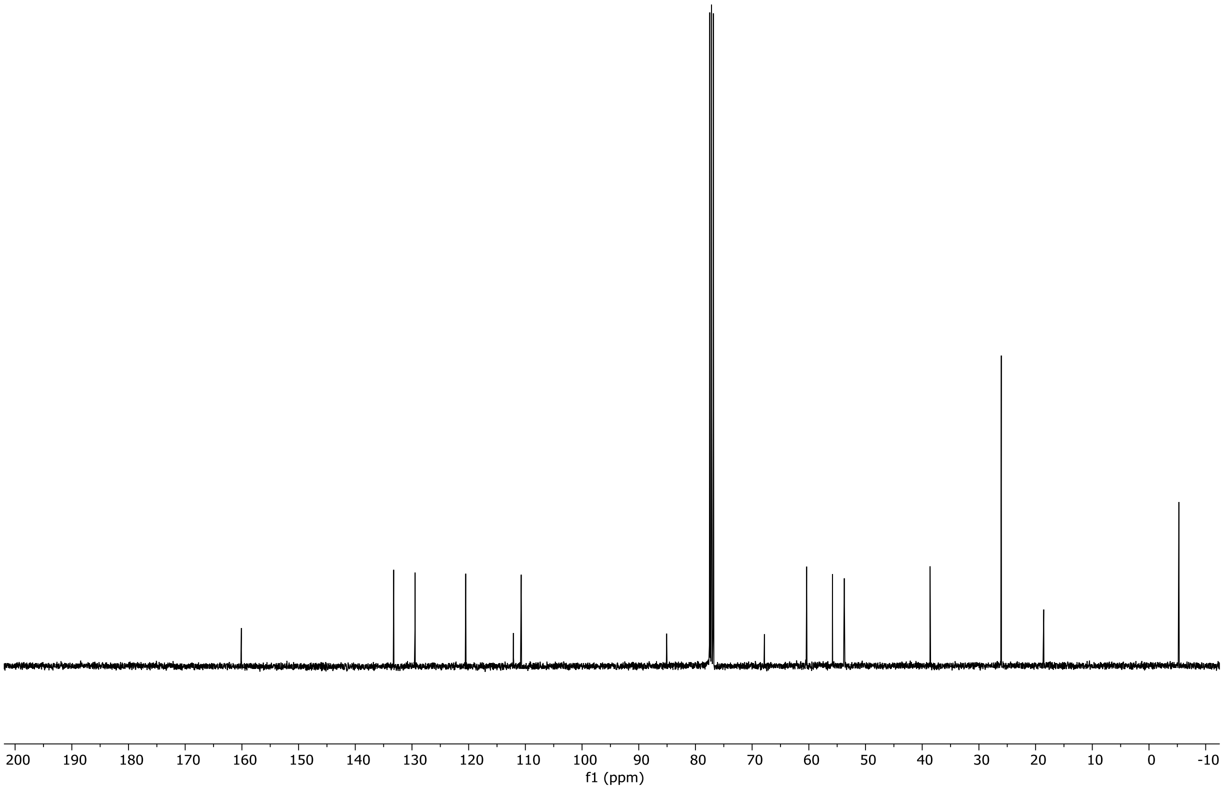

Supplementary Fig 36. ^{1}H (top) and ^{13}C (bottom) NMR spectra of compound **S1i**.

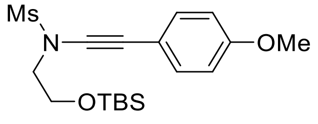

S1j

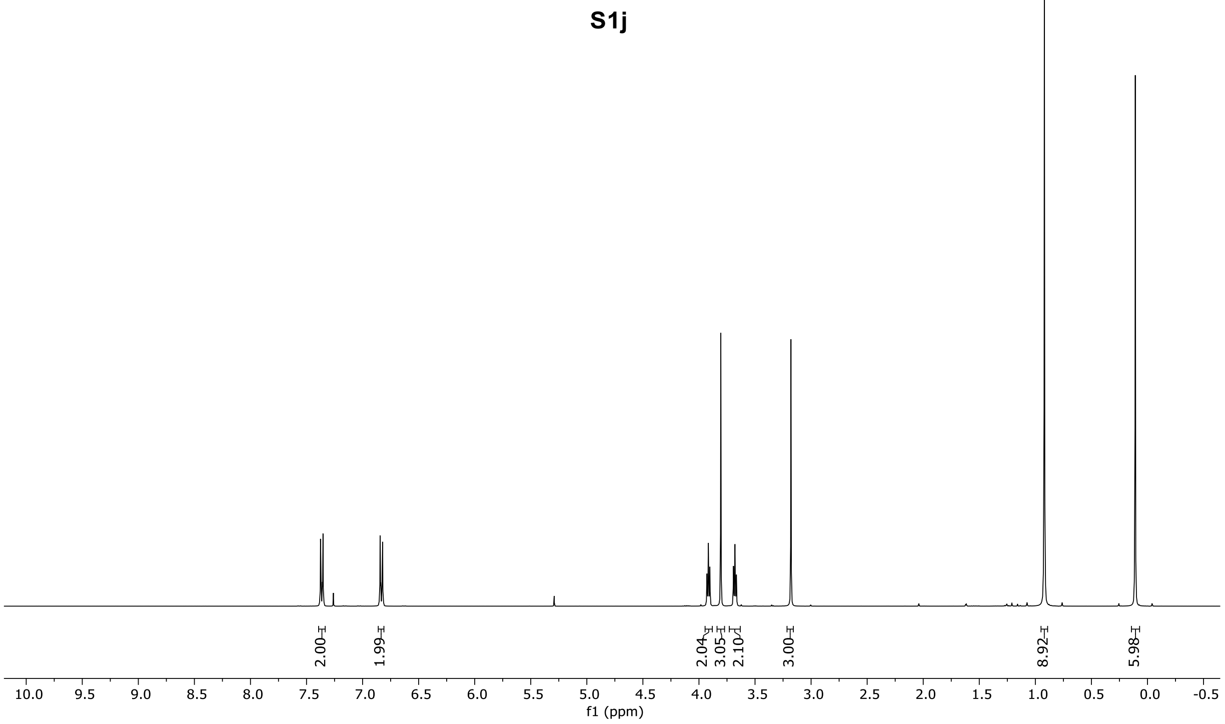

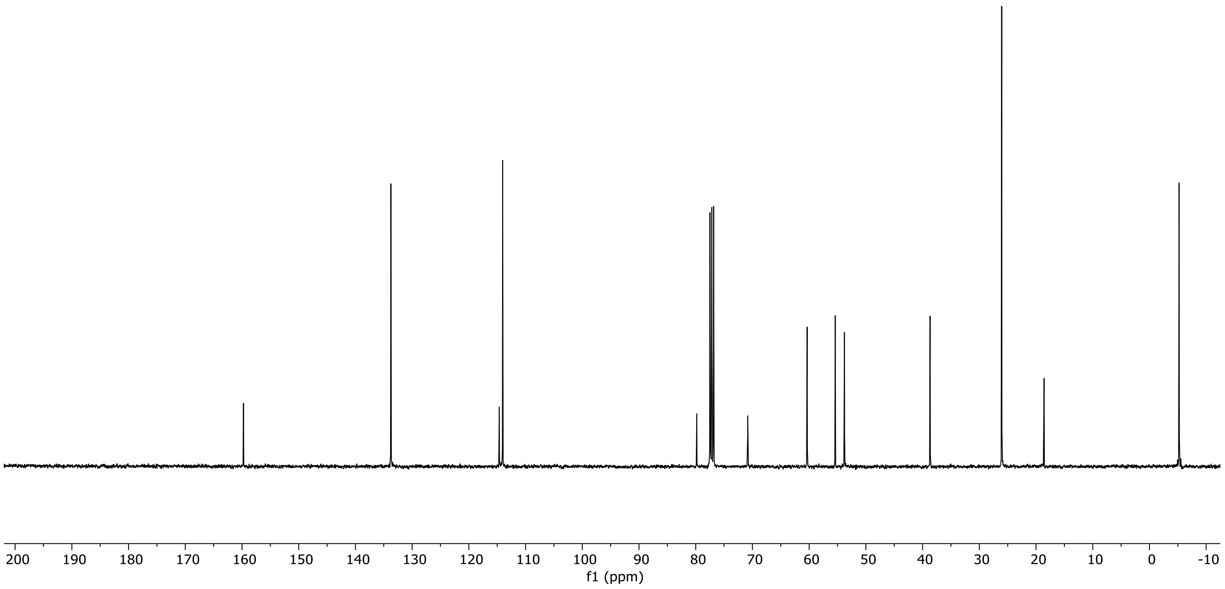

Supplementary Fig 37. ^{1}H (top) and ^{13}C (bottom) NMR spectra of compound **S1j**.

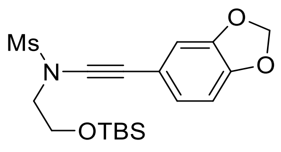

S1k

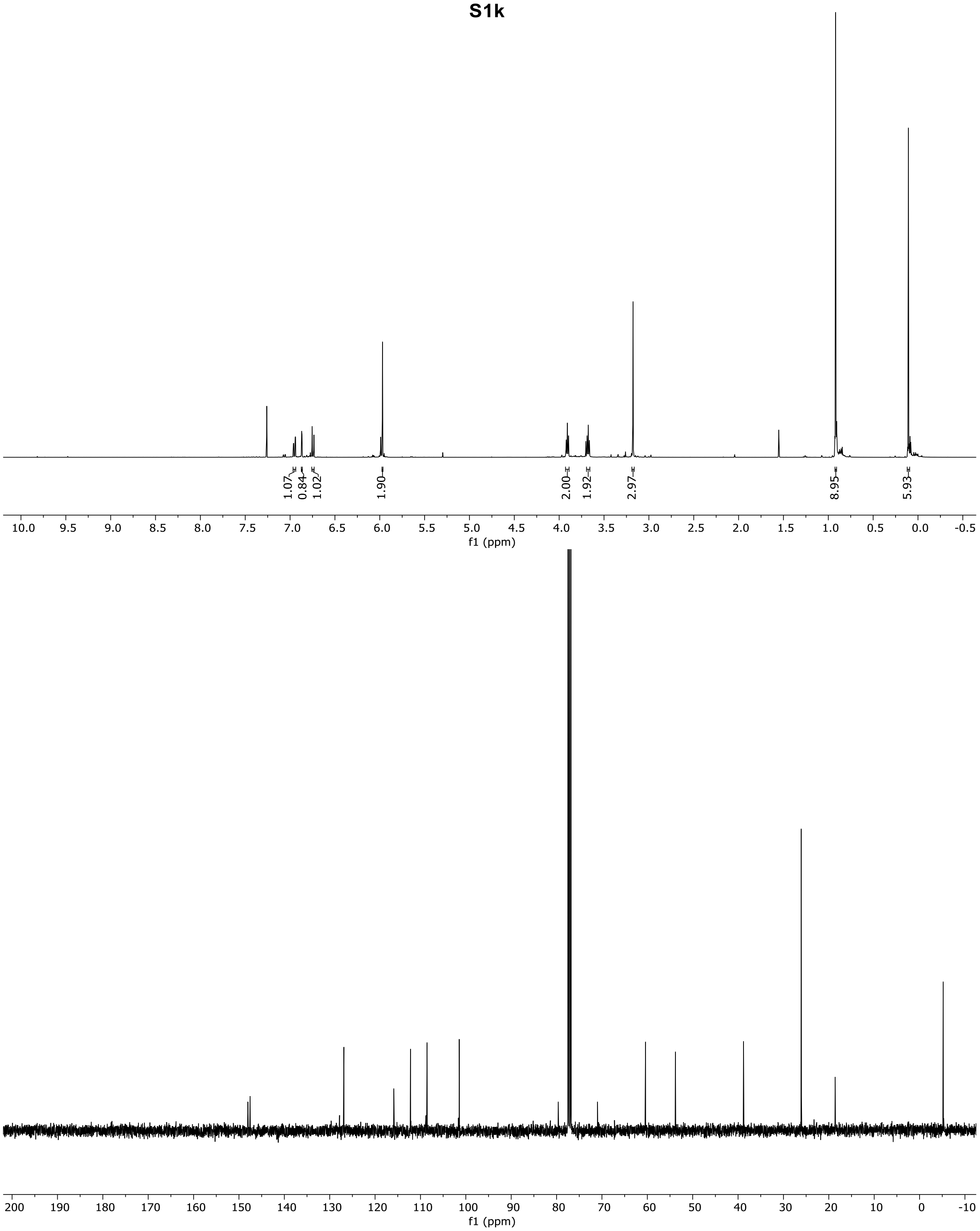

Supplementary Fig 38. ^{1}H (top) and ^{13}C (bottom) NMR spectra of compound **S1k**.

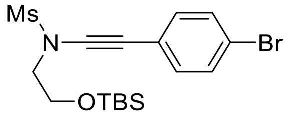

S1l

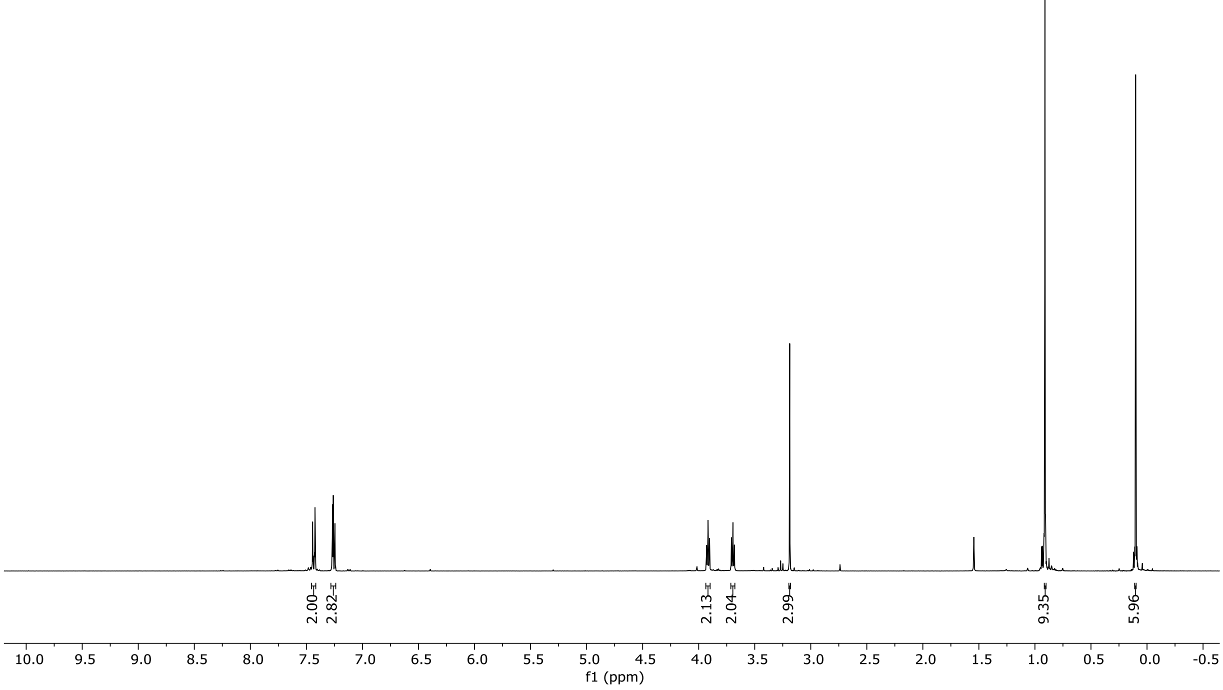

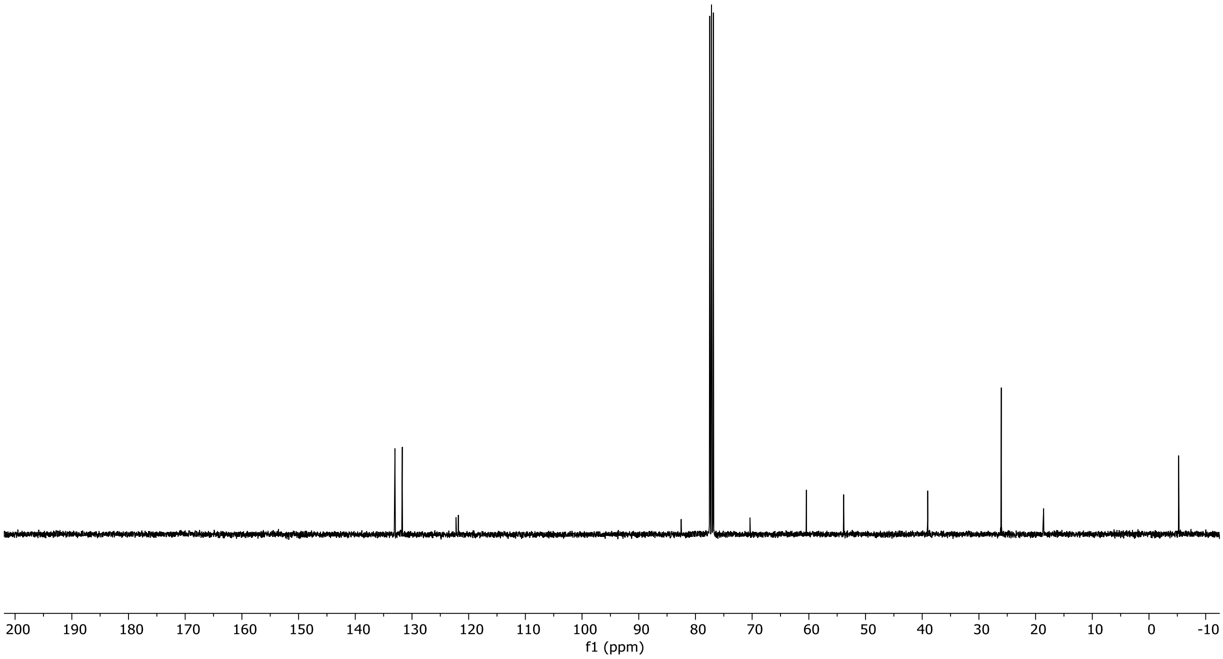

Supplementary Fig 39. ^{1}H (top) and ^{13}C (bottom) NMR spectra of compound **S1l**.

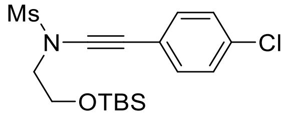

S1m

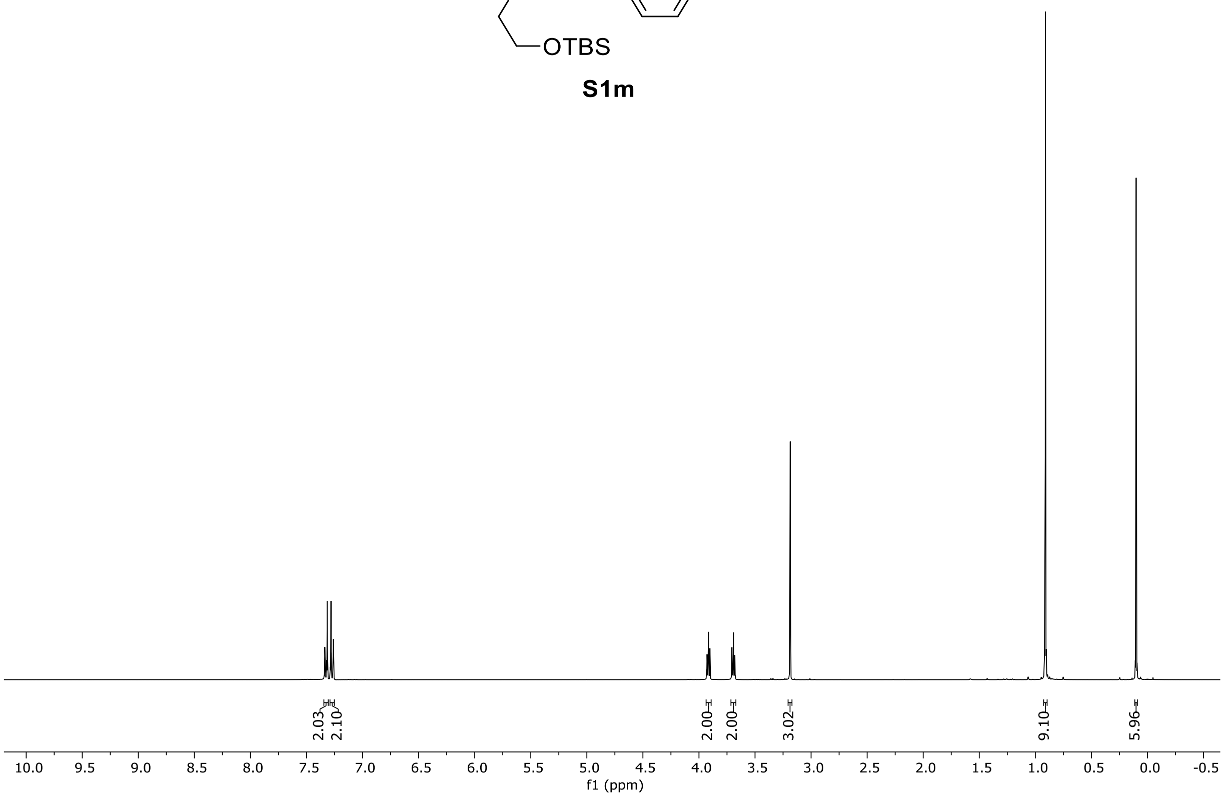

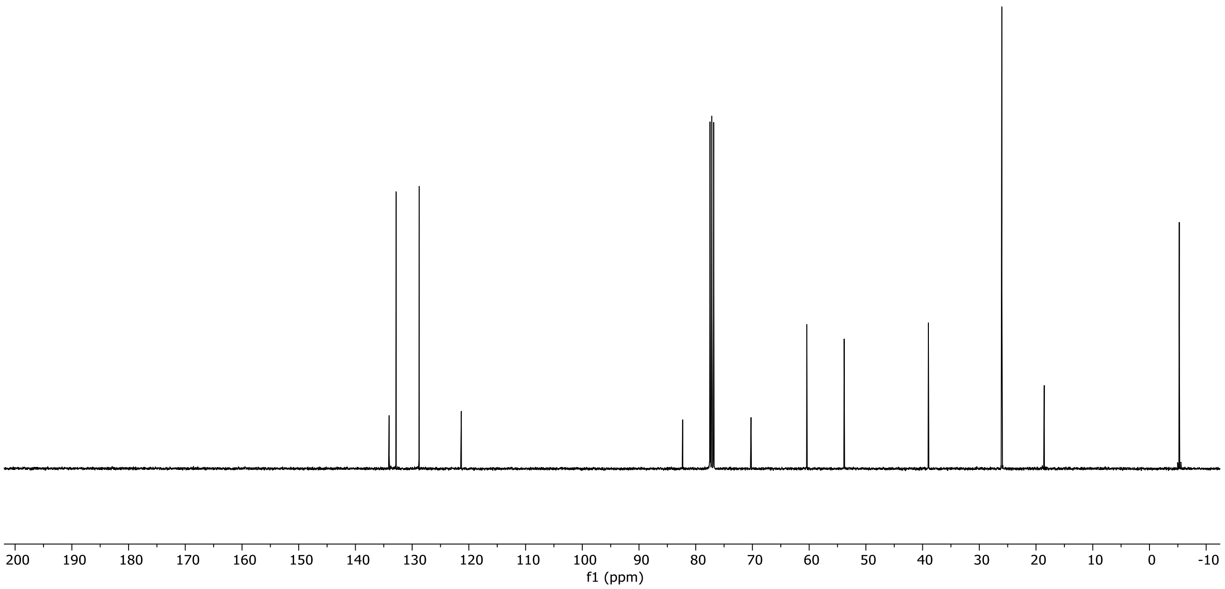

Supplementary Fig 40. ^{1}H (top) and ^{13}C (bottom) NMR spectra of compound **S1m**.

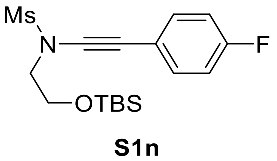

S1n

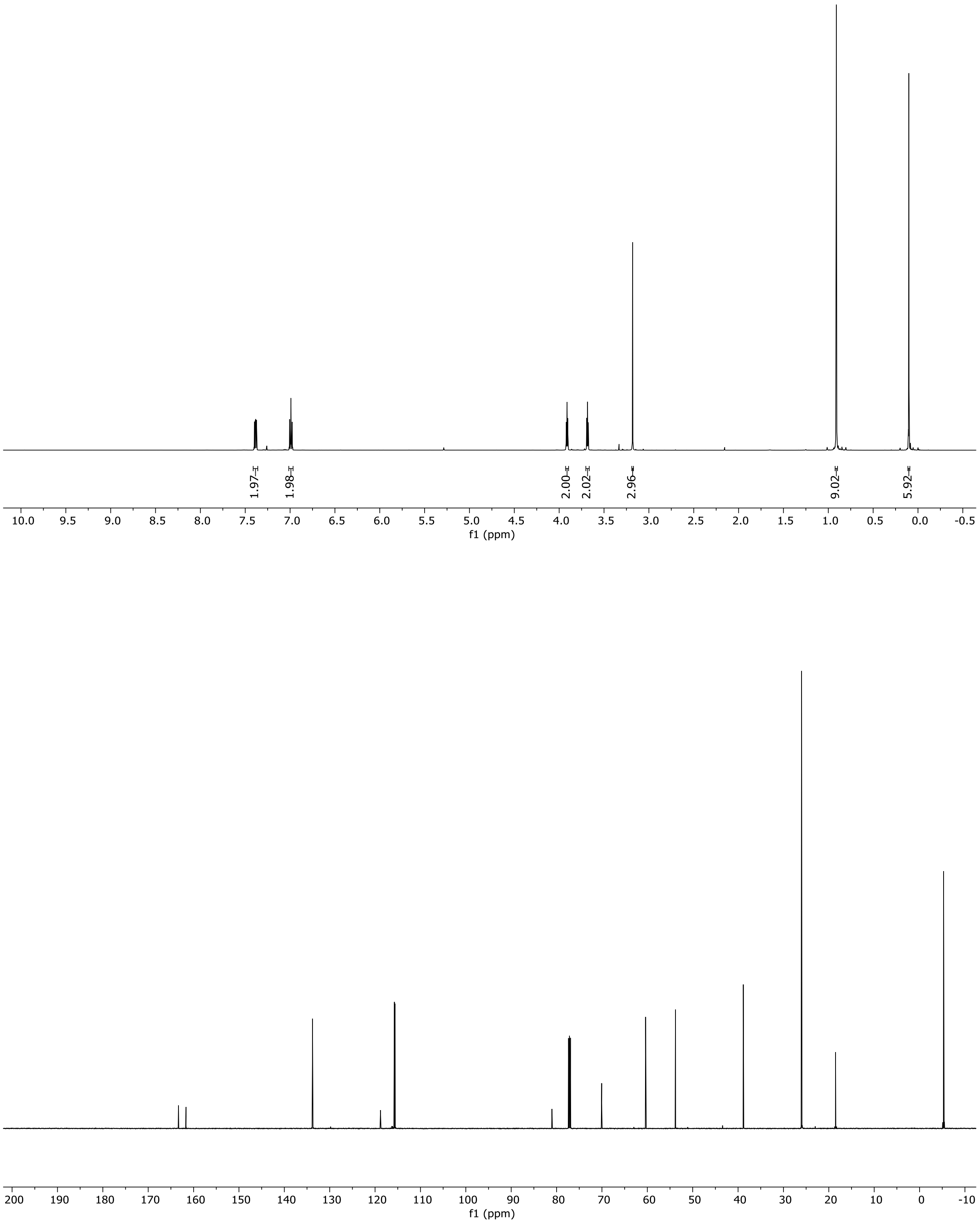

Supplementary Fig 41. ^{1}H (top) and ^{13}C (bottom) NMR spectra of compound **S1n**.

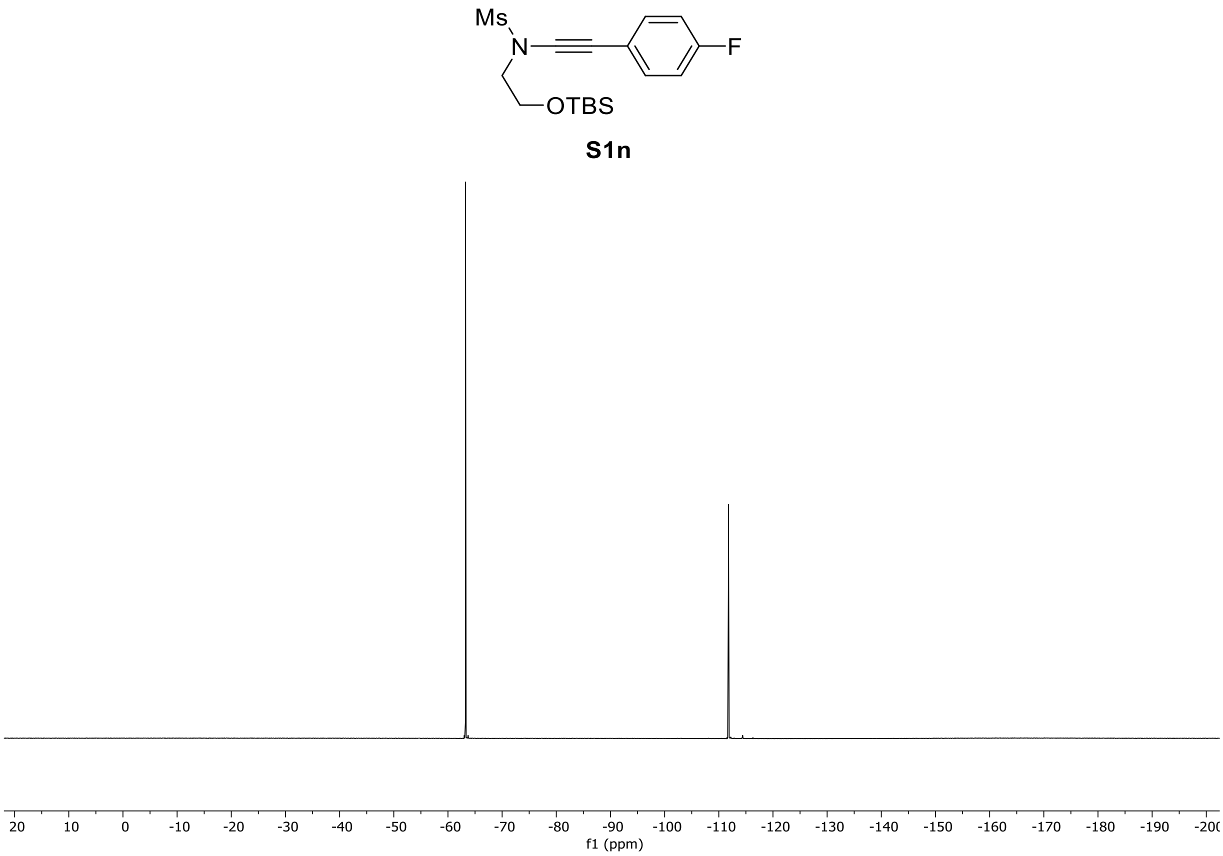

Supplementary Fig 42. ^{19}F NMR spectrum of compound **S1n**.

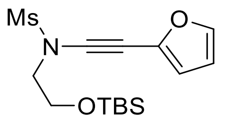

S1o

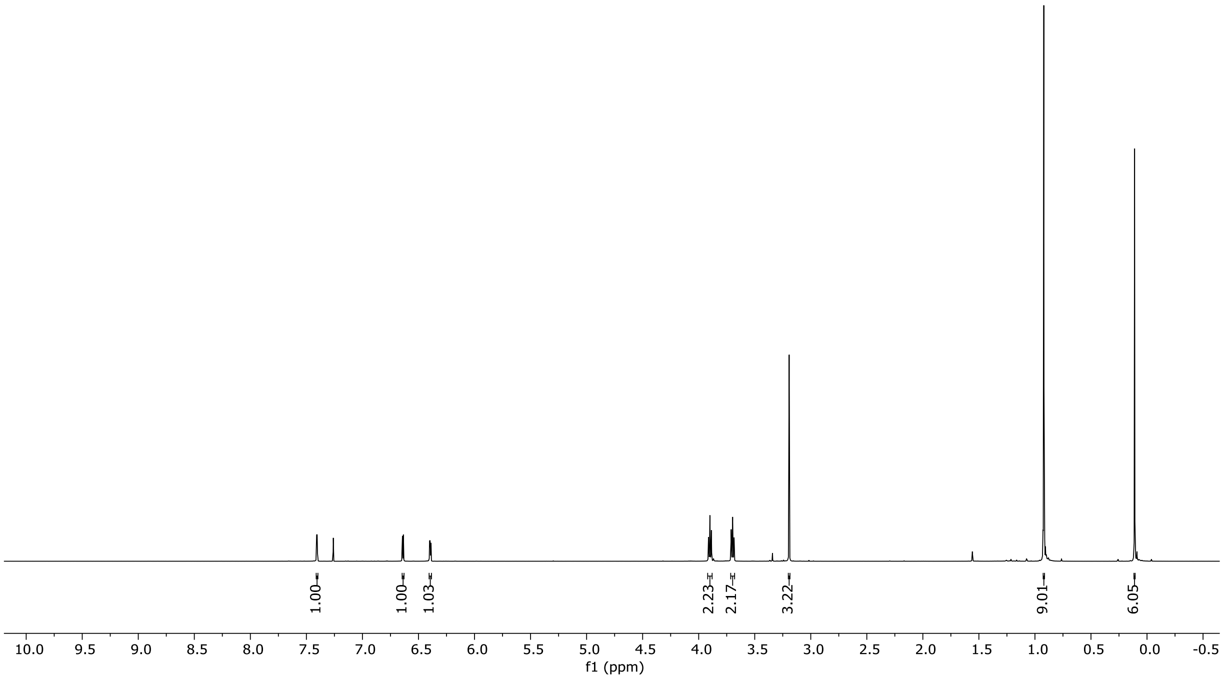

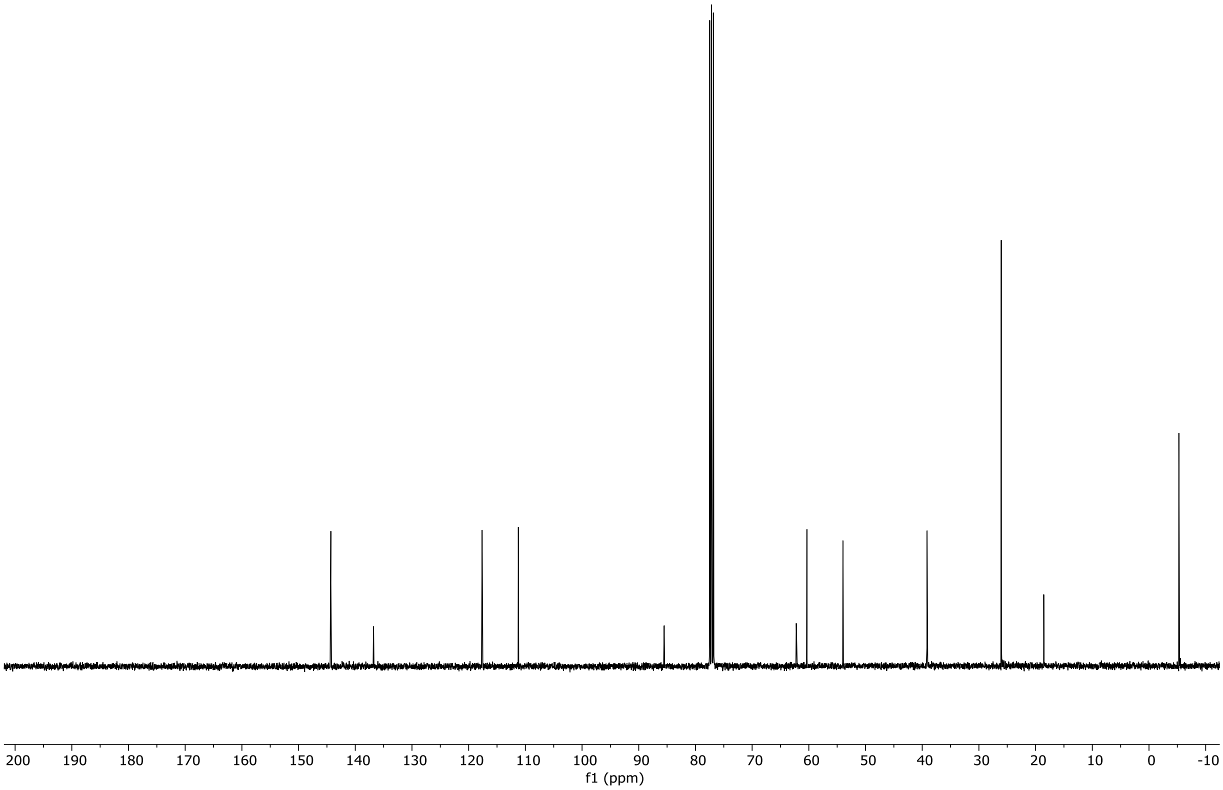

Supplementary Fig 43. ^{1}H (top) and ^{13}C (bottom) NMR spectra of compound **S1o**.

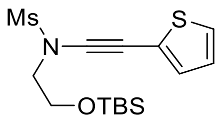

S1p

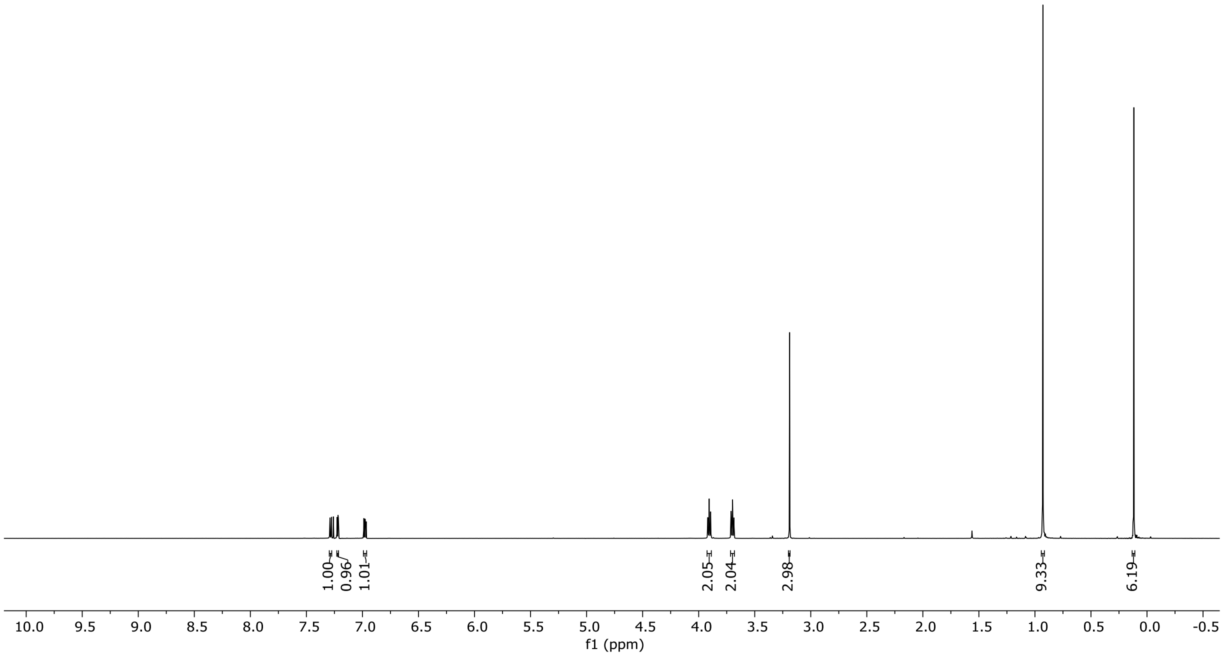

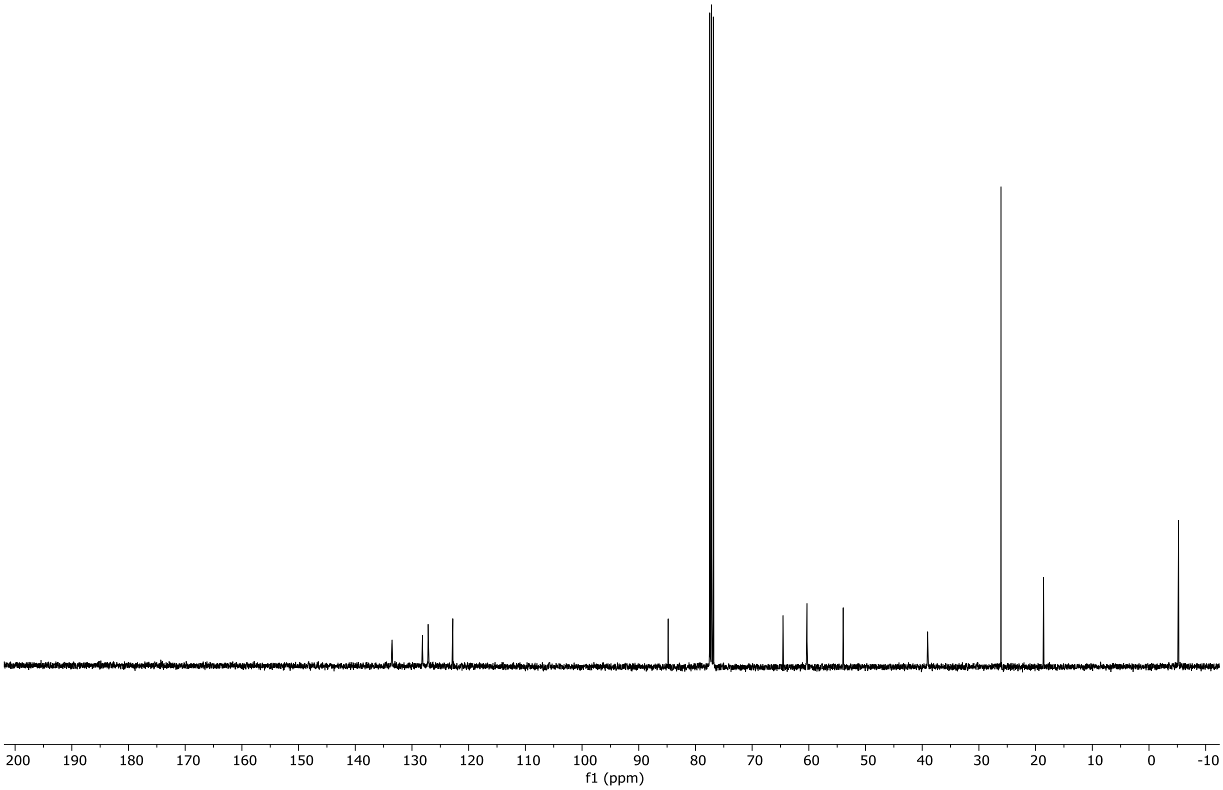

Supplementary Fig 44. ^{1}H (top) and ^{13}C (bottom) NMR spectra of compound **S1p**.

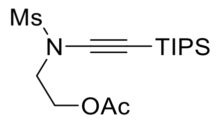

S1q

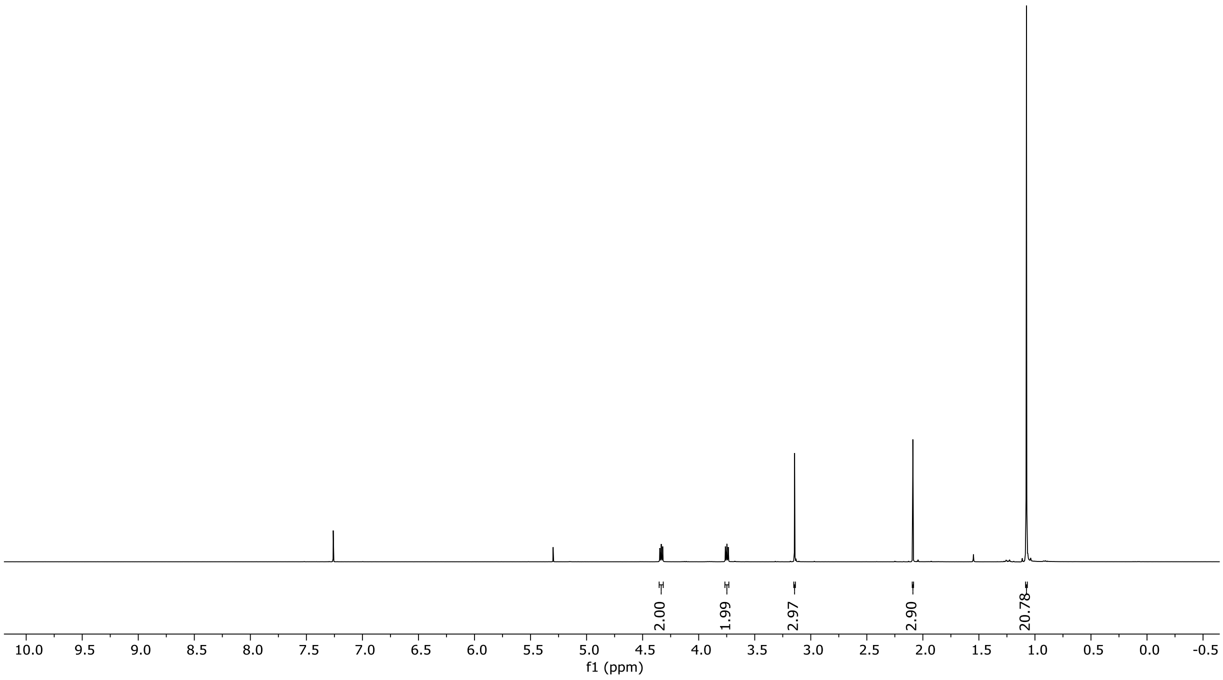

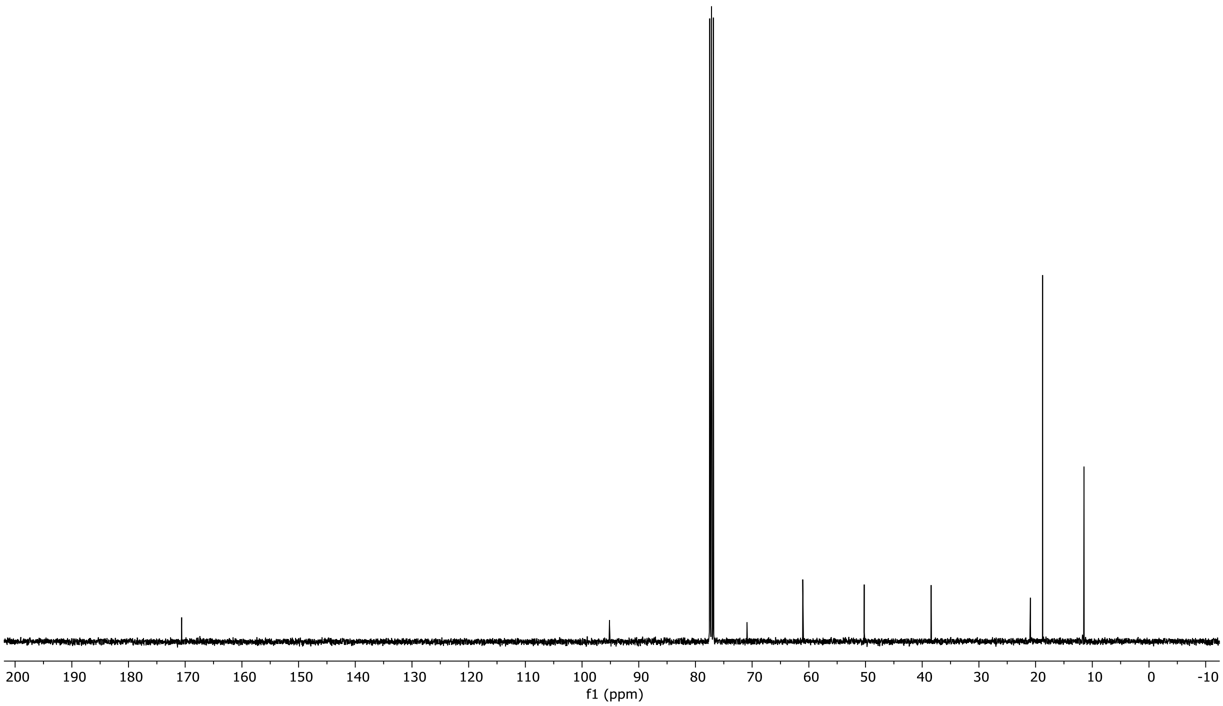

Supplementary Fig 45. ^{1}H (top) and ^{13}C (bottom) NMR spectra of compound **S1q**.

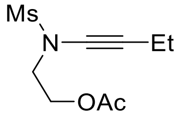

S1r

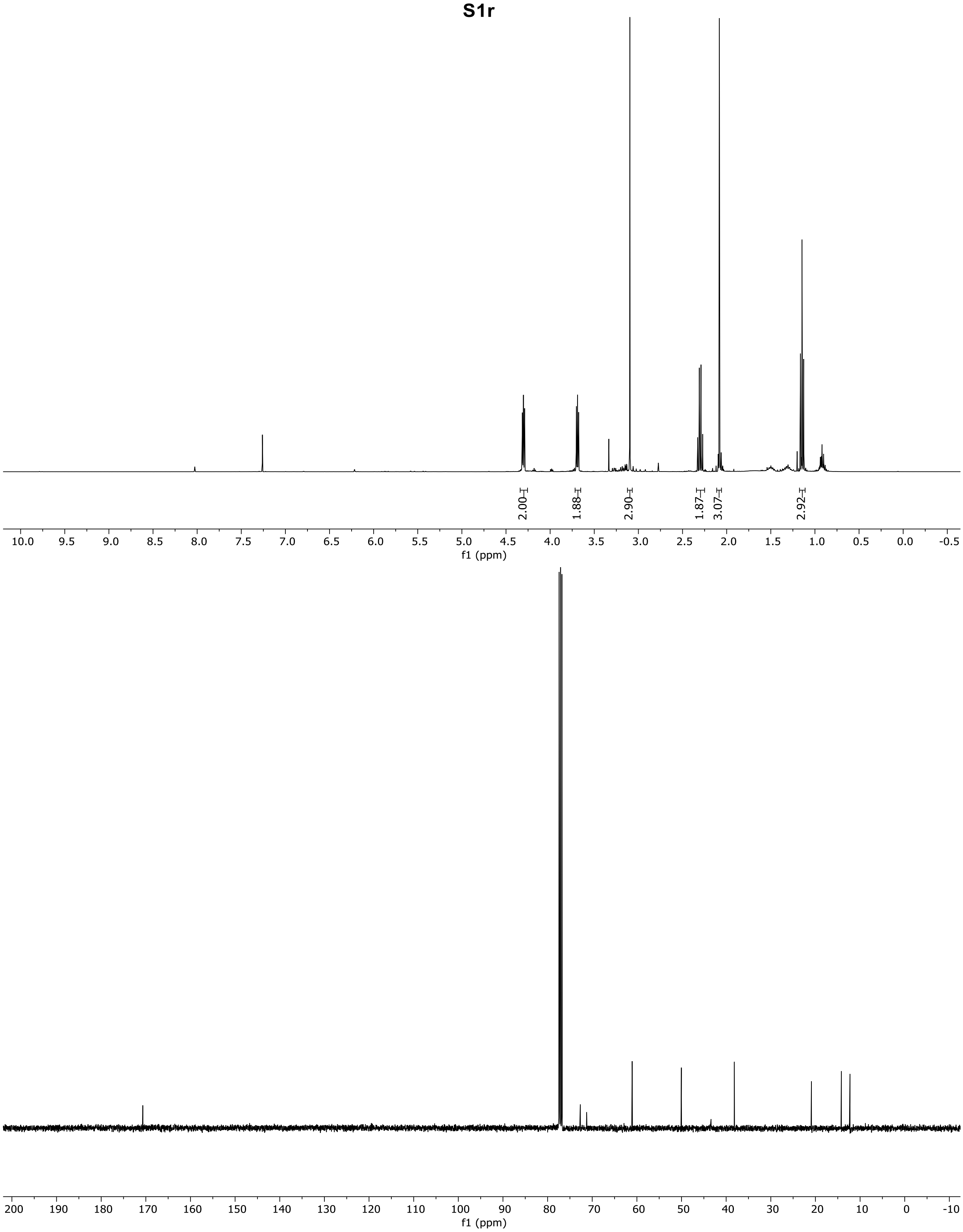

Supplementary Fig 46. ^{1}H (top) and ^{13}C (bottom) NMR spectra of compound **S1r**.

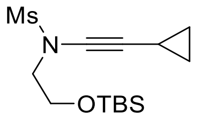

S1s

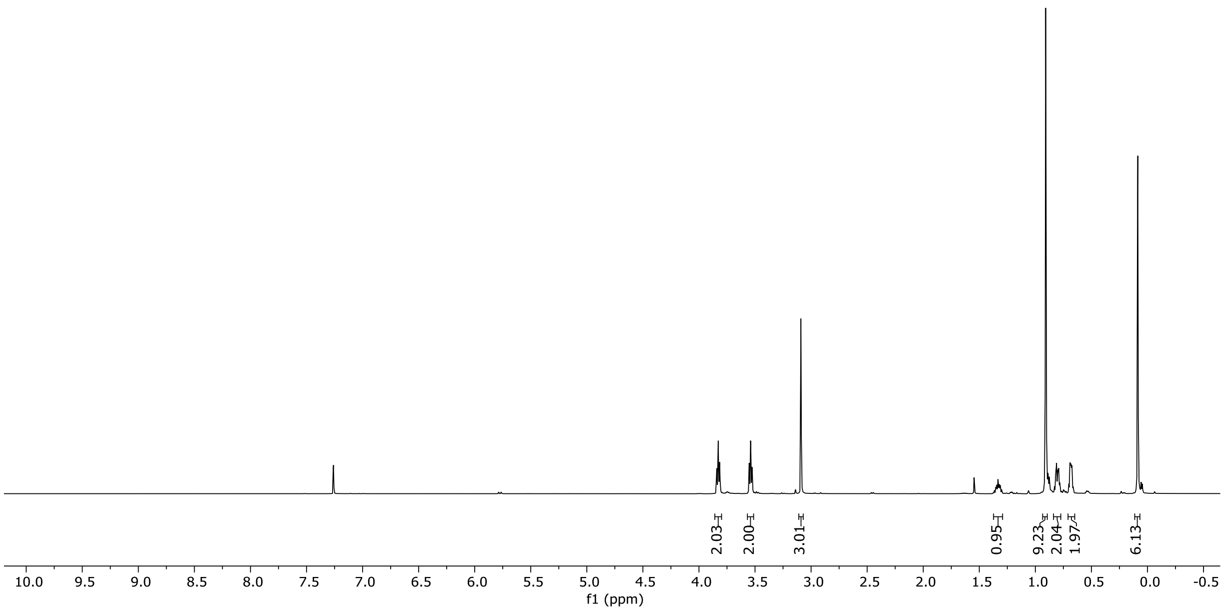

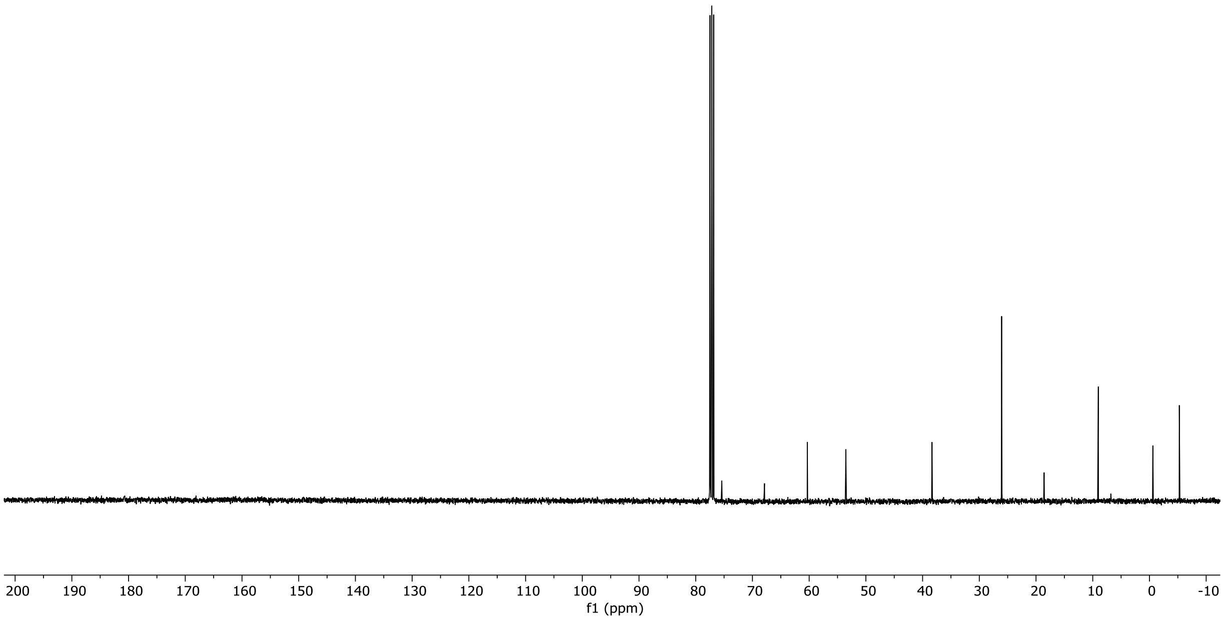

Supplementary Fig 47. ^{1}H (top) and ^{13}C (bottom) NMR spectra of compound **S1s**.

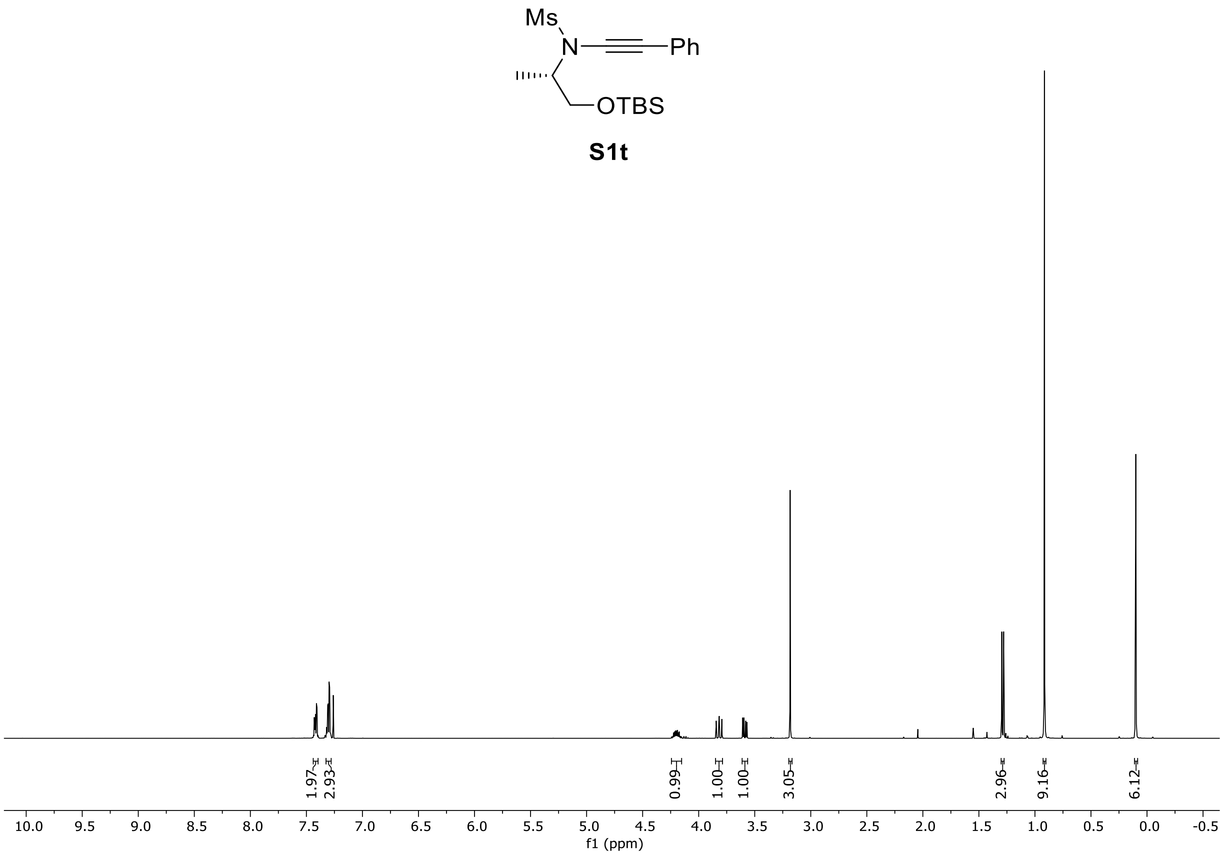

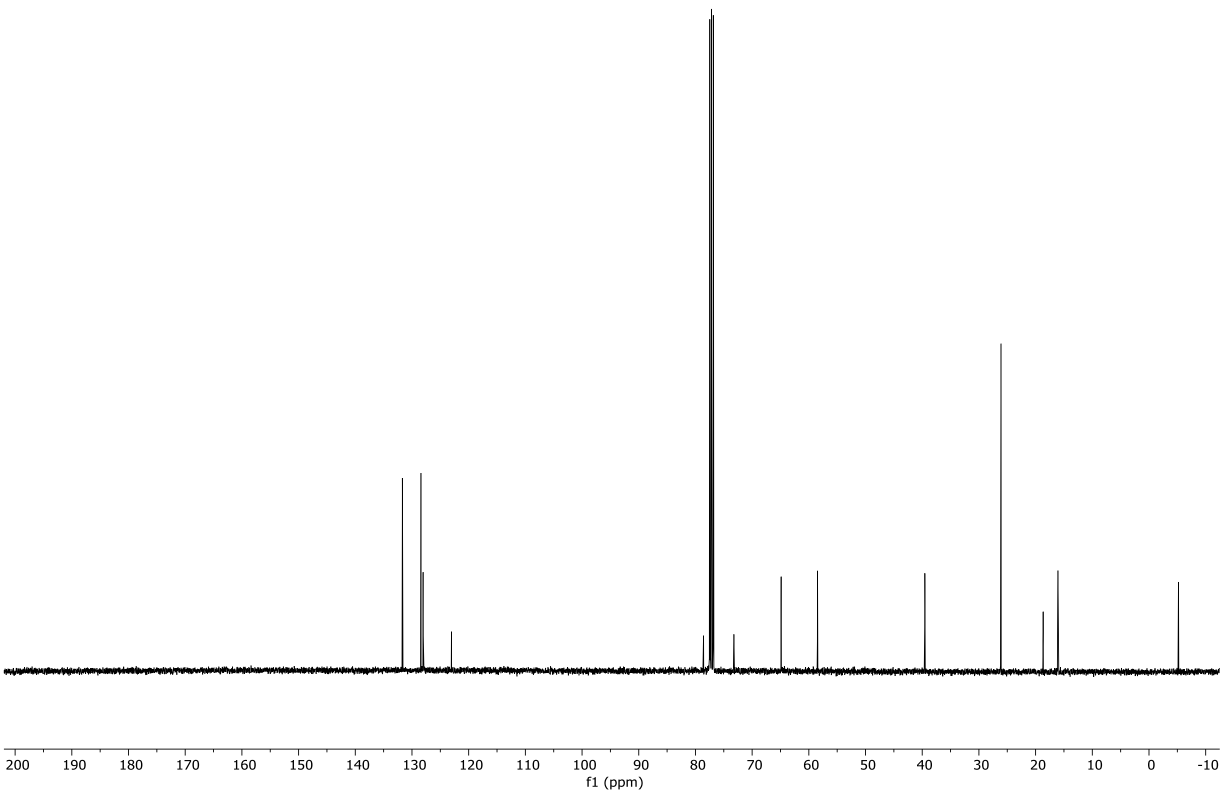

Supplementary Fig 48. ^{1}H (top) and ^{13}C (bottom) NMR spectra of compound **S1t**.

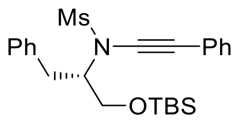

S1u

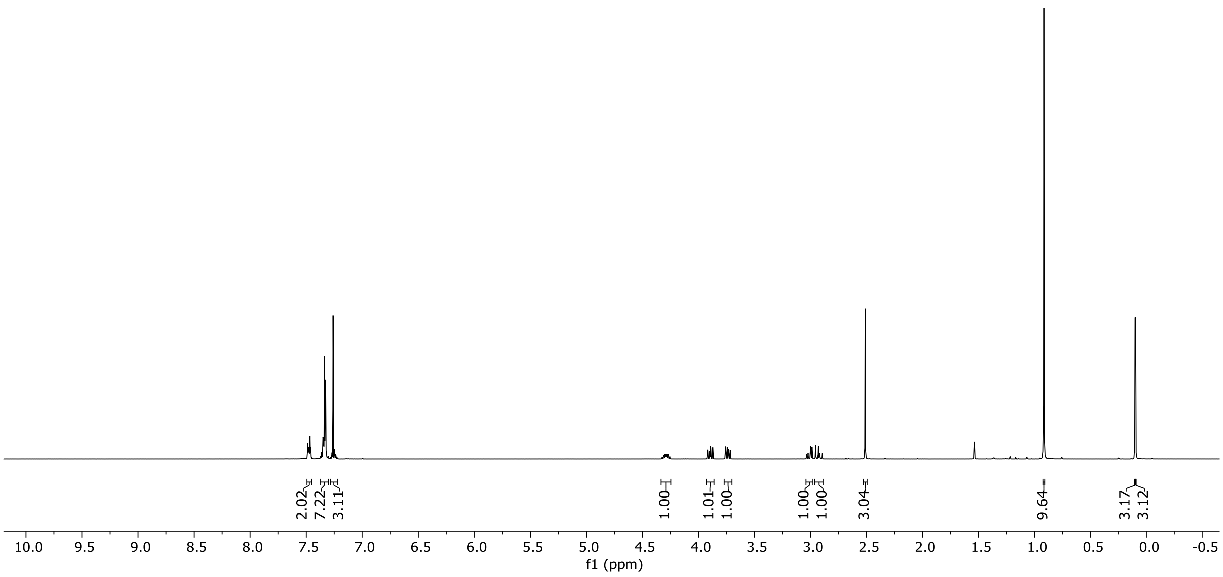

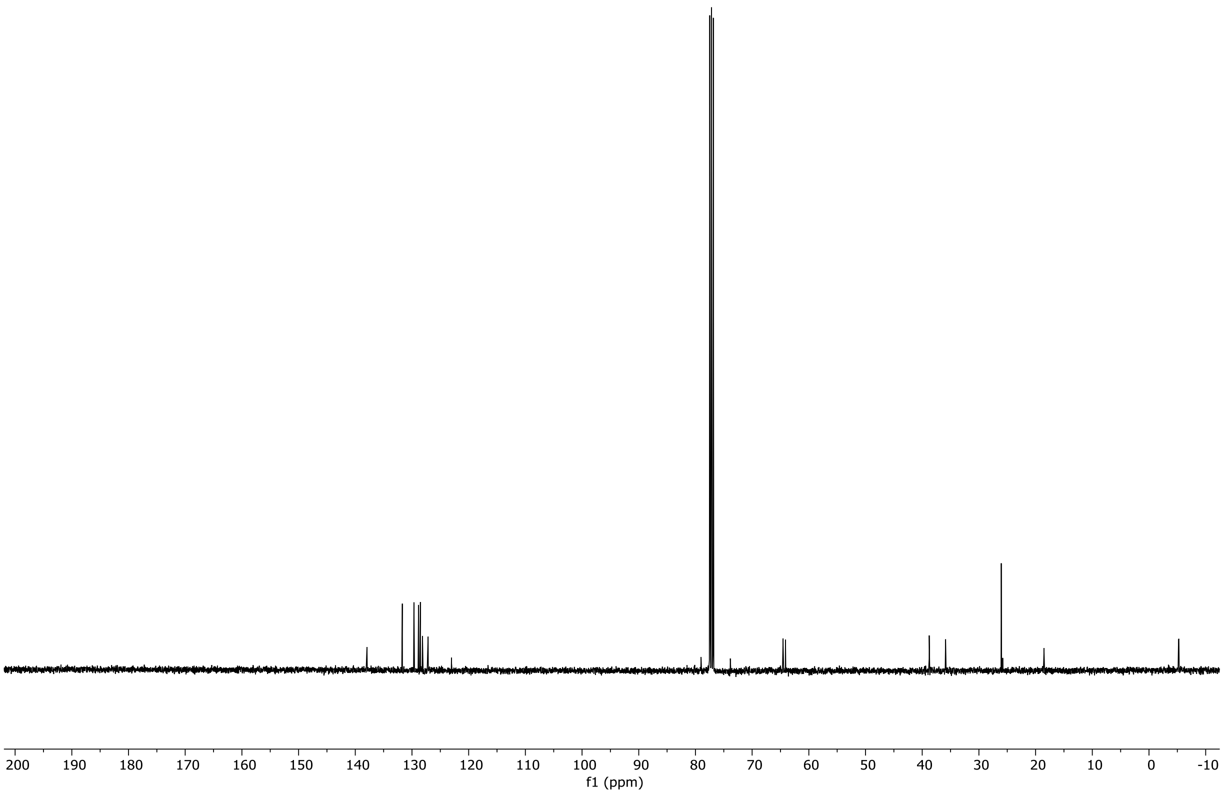

Supplementary Fig 49. ^{1}H (top) and ^{13}C (bottom) NMR spectra of compound **S1u**.

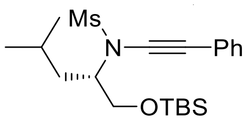

S1v

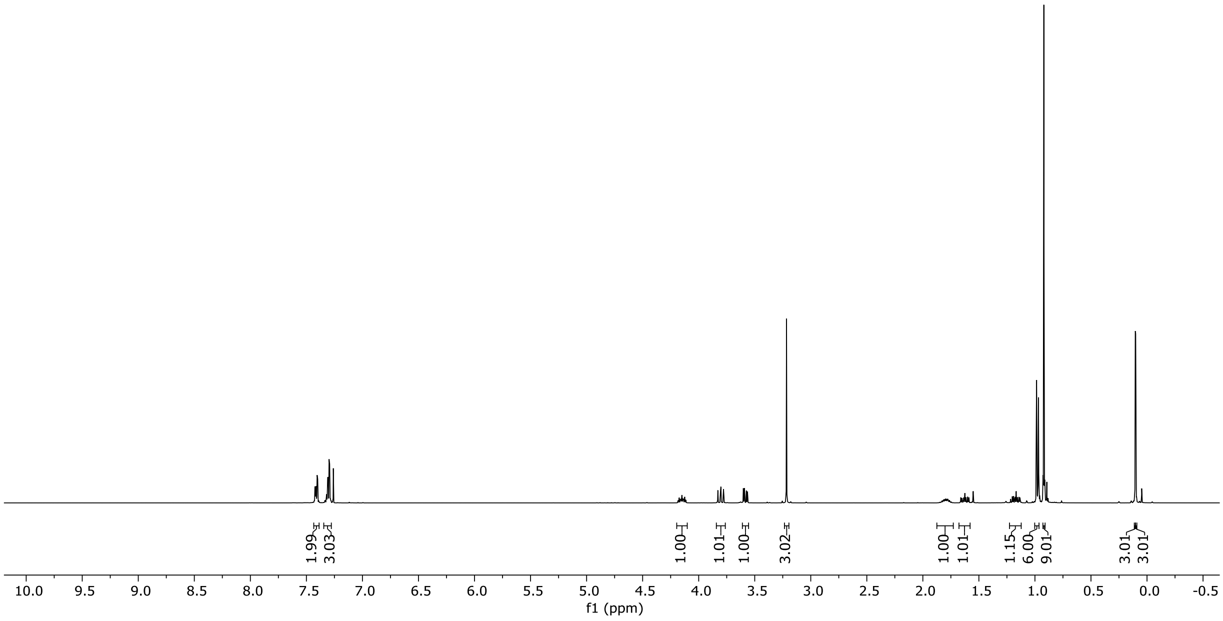

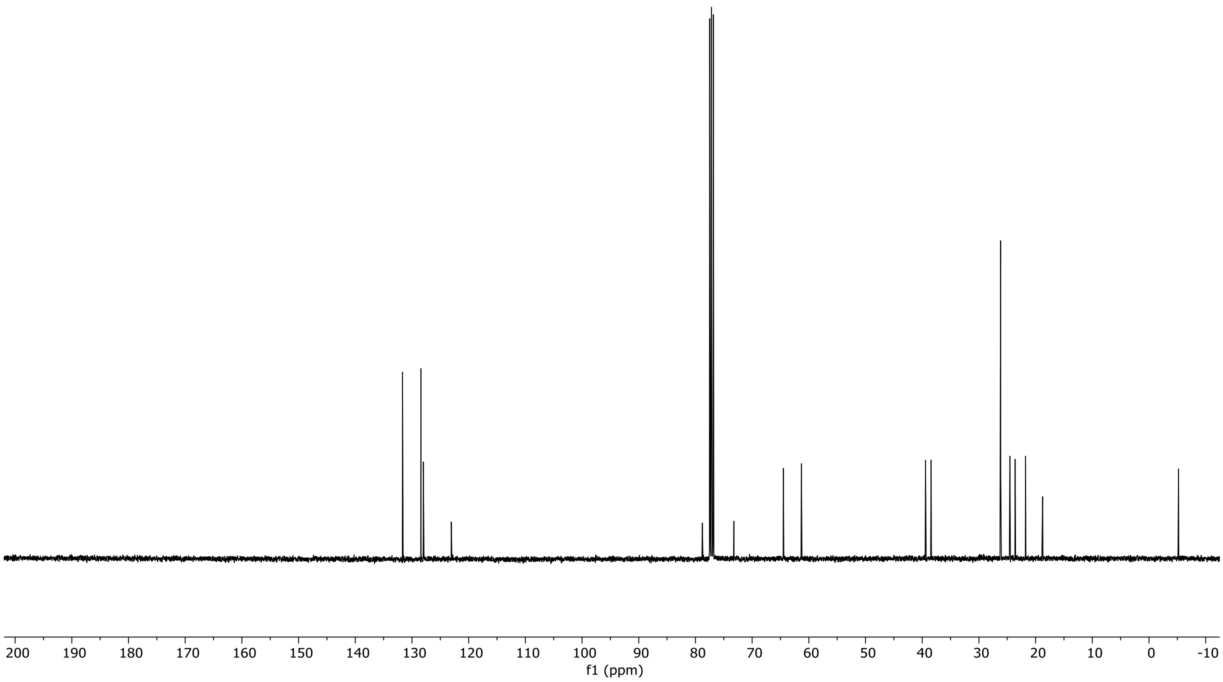

Supplementary Fig 50. ^{1}H (top) and ^{13}C (bottom) NMR spectra of compound **S1v**.

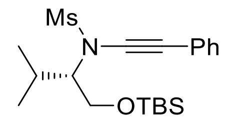

S1w

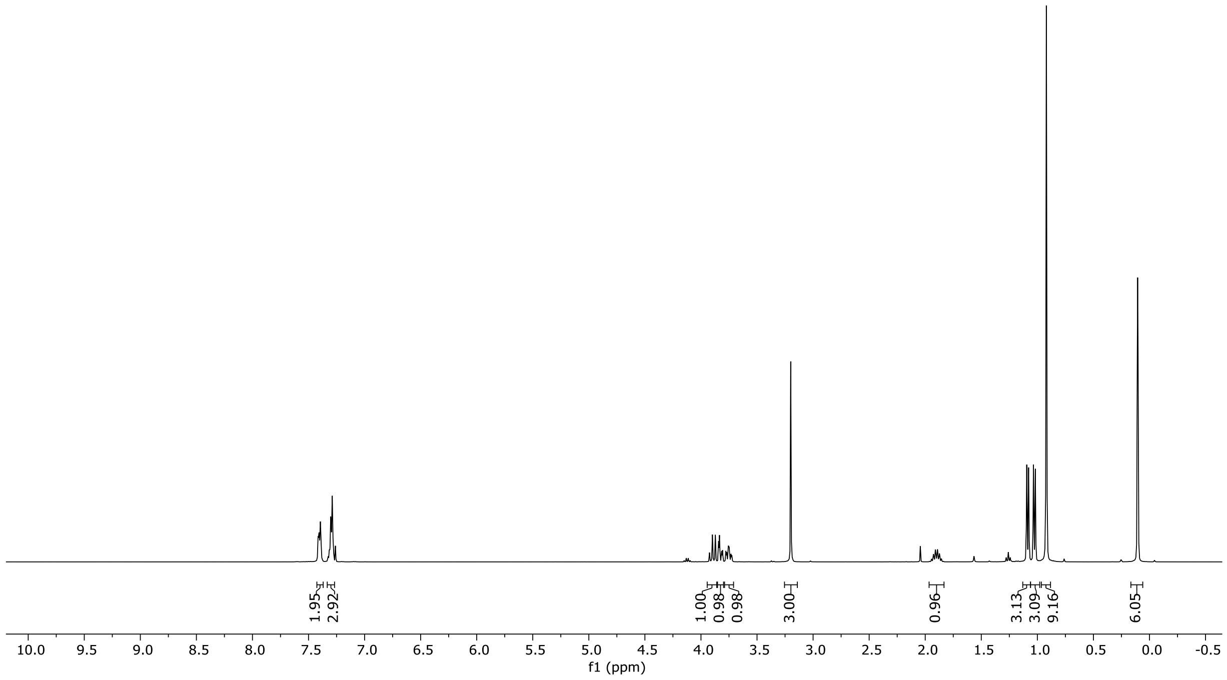

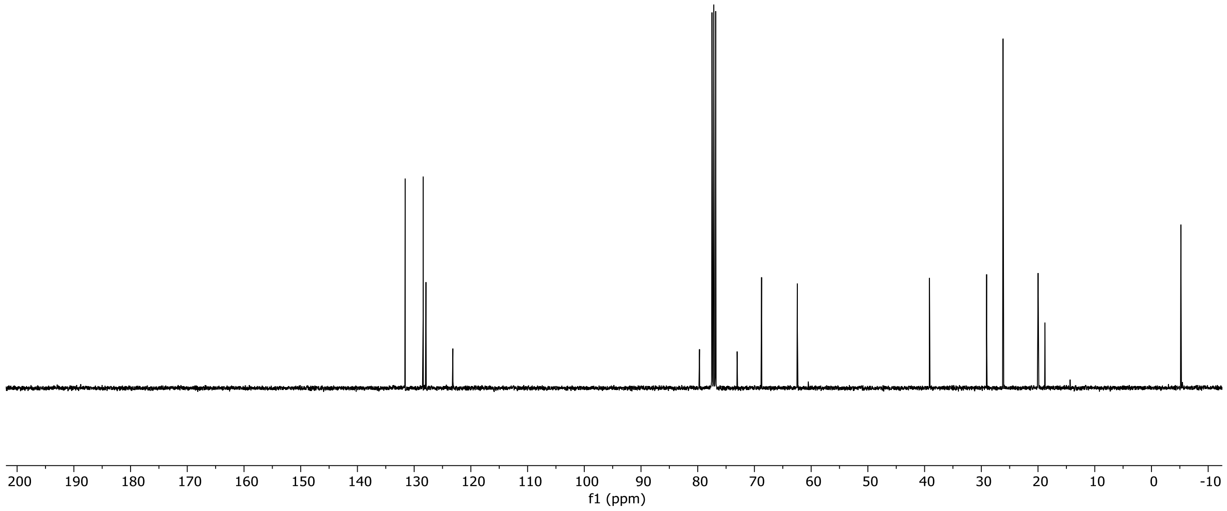

Supplementary Fig 51. ^{1}H (top) and ^{13}C (bottom) NMR spectra of compound **S1w**.

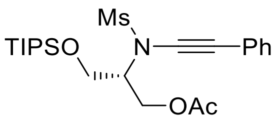

S1x

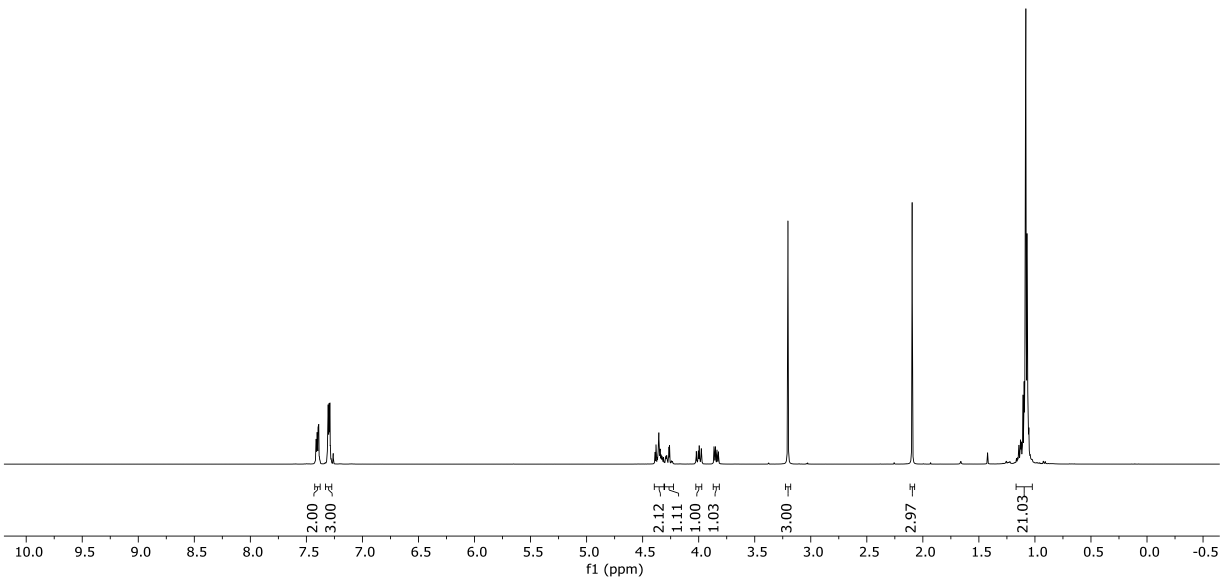

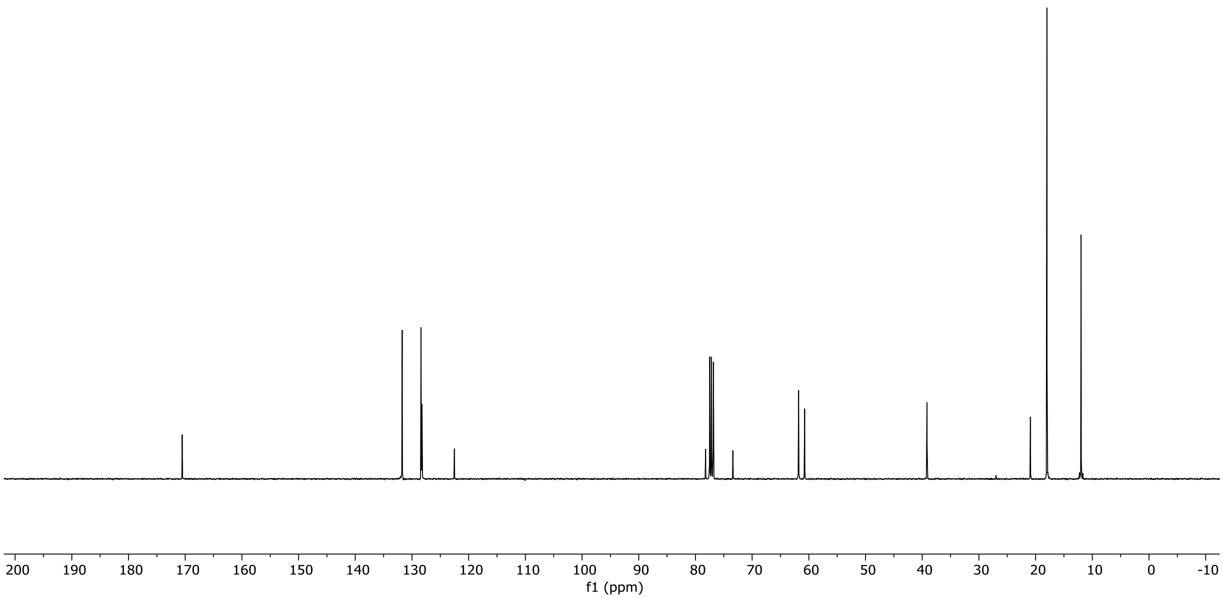

Supplementary Fig 52. ^{1}H (top) and ^{13}C (bottom) NMR spectra of compound **S1x**.

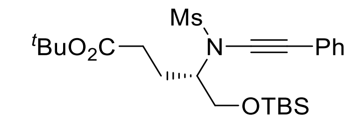

S1y

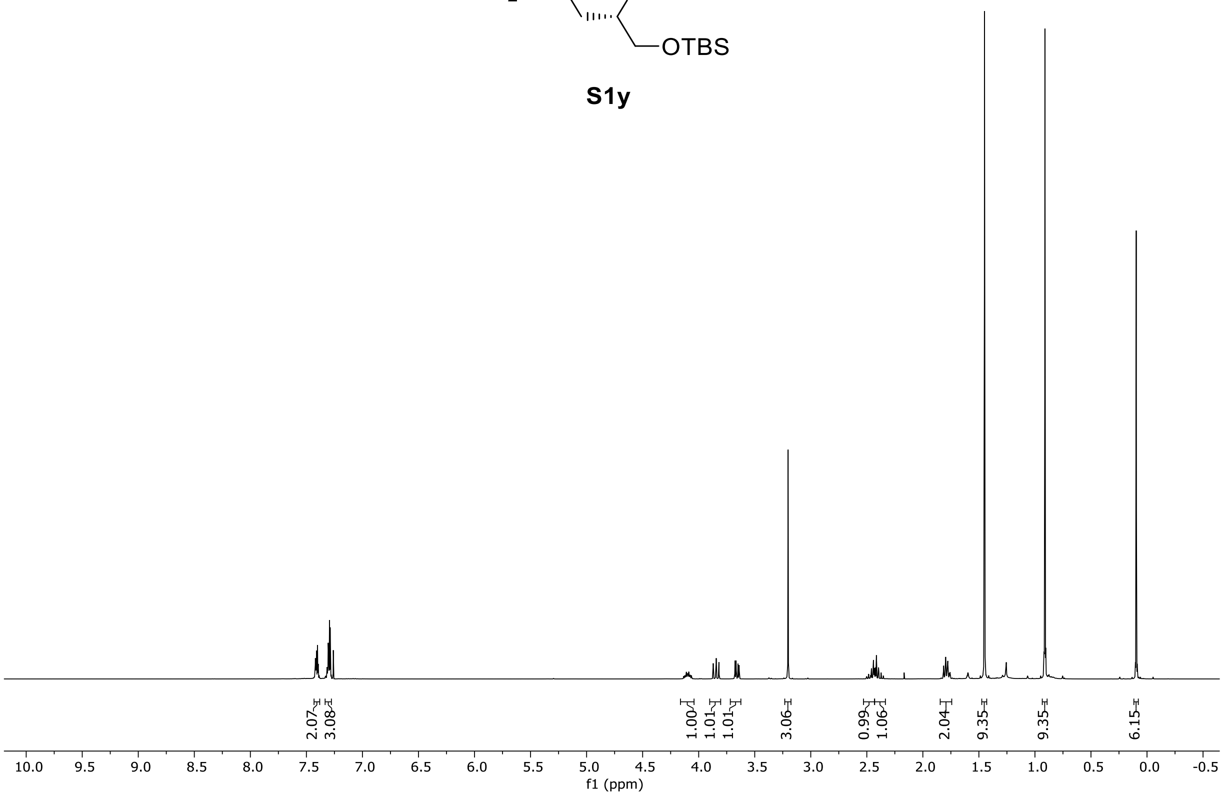

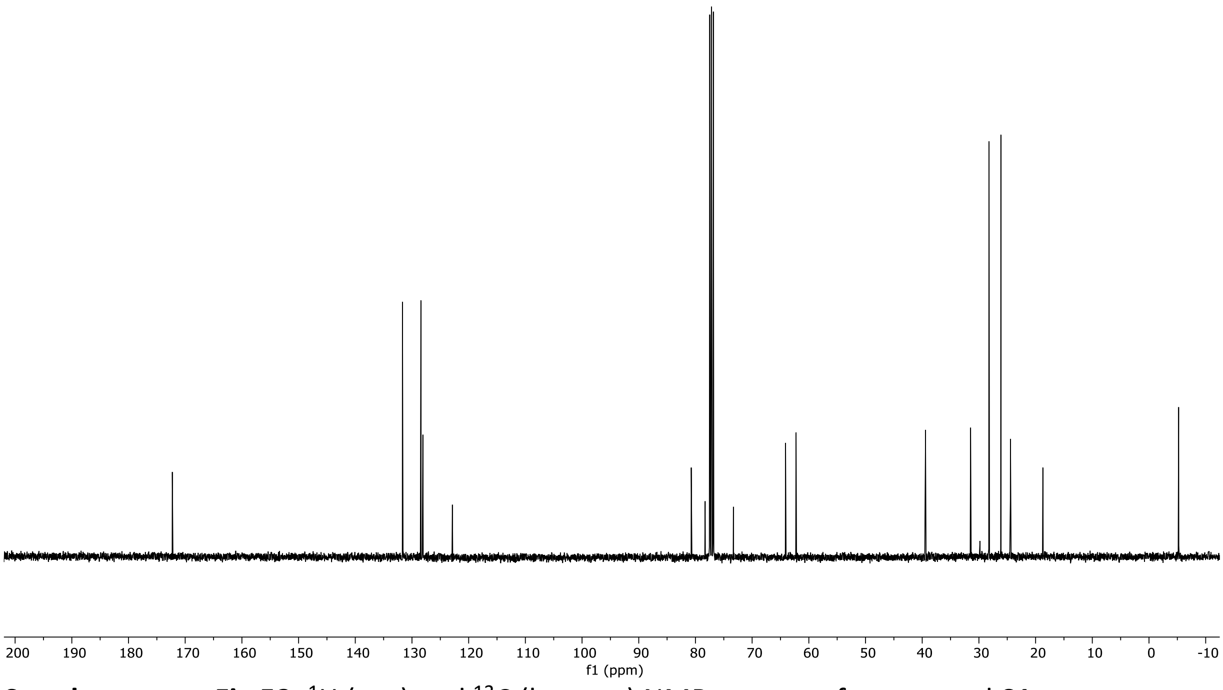

Supplementary Fig 53. ^{1}H (top) and ^{13}C (bottom) NMR spectra of compound **S1y**.

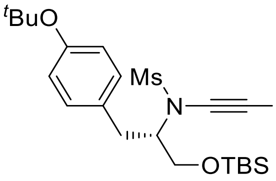

S1z

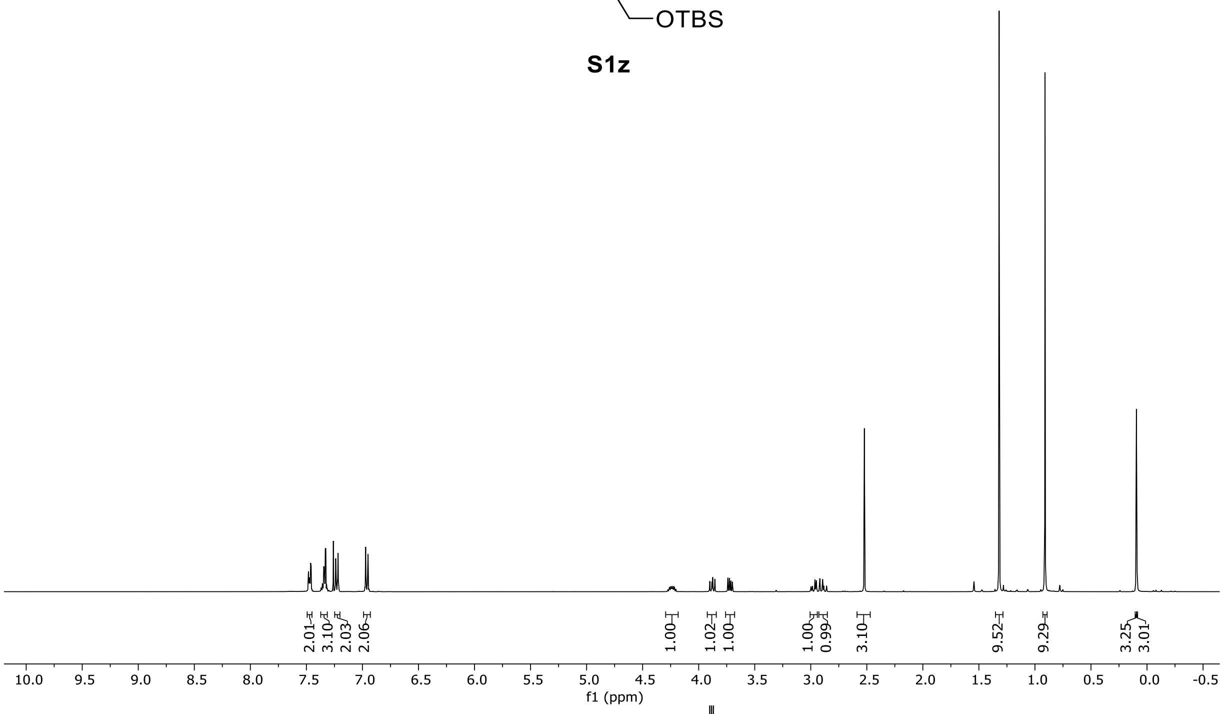

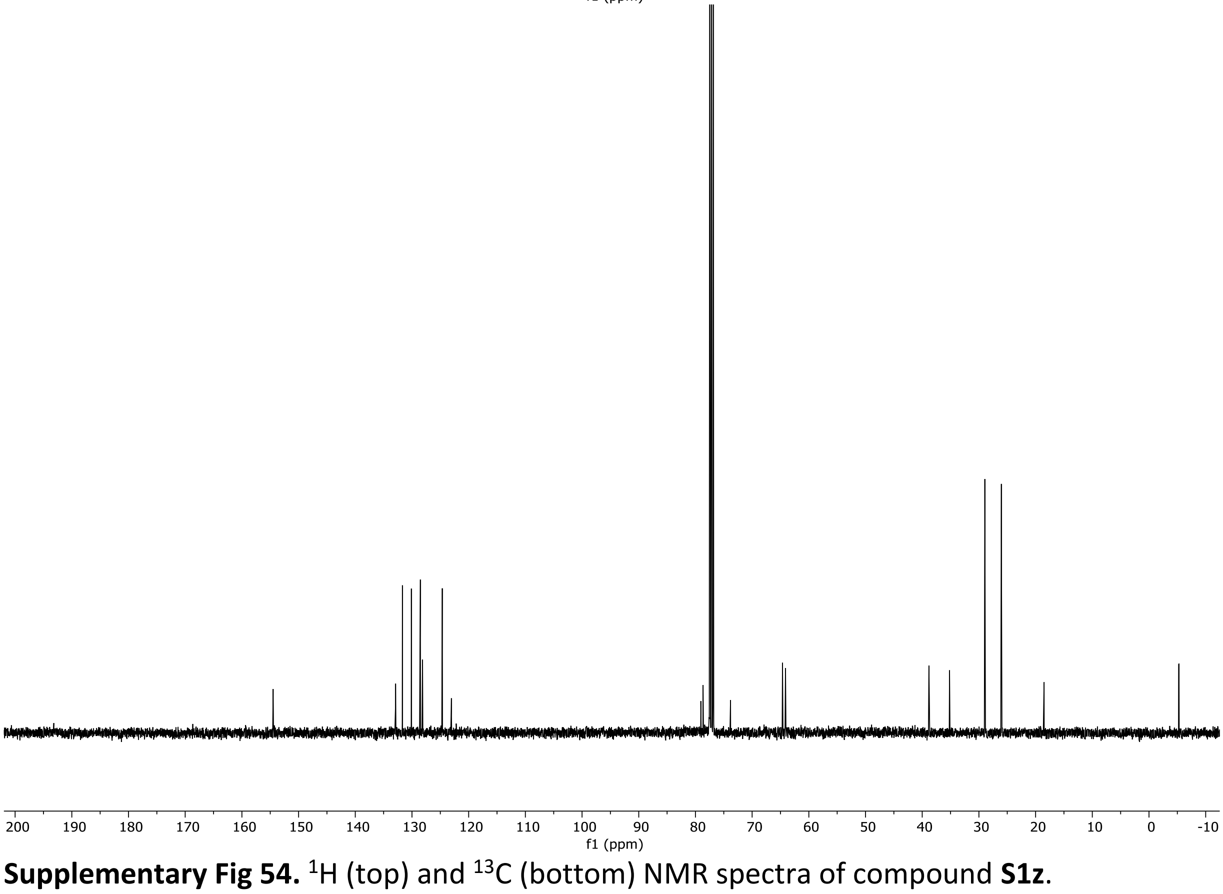

Supplementary Fig 54. ^{1}H (top) and ^{13}C (bottom) NMR spectra of compound **S1z**.

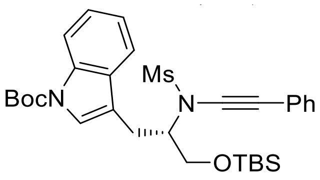

S1aa

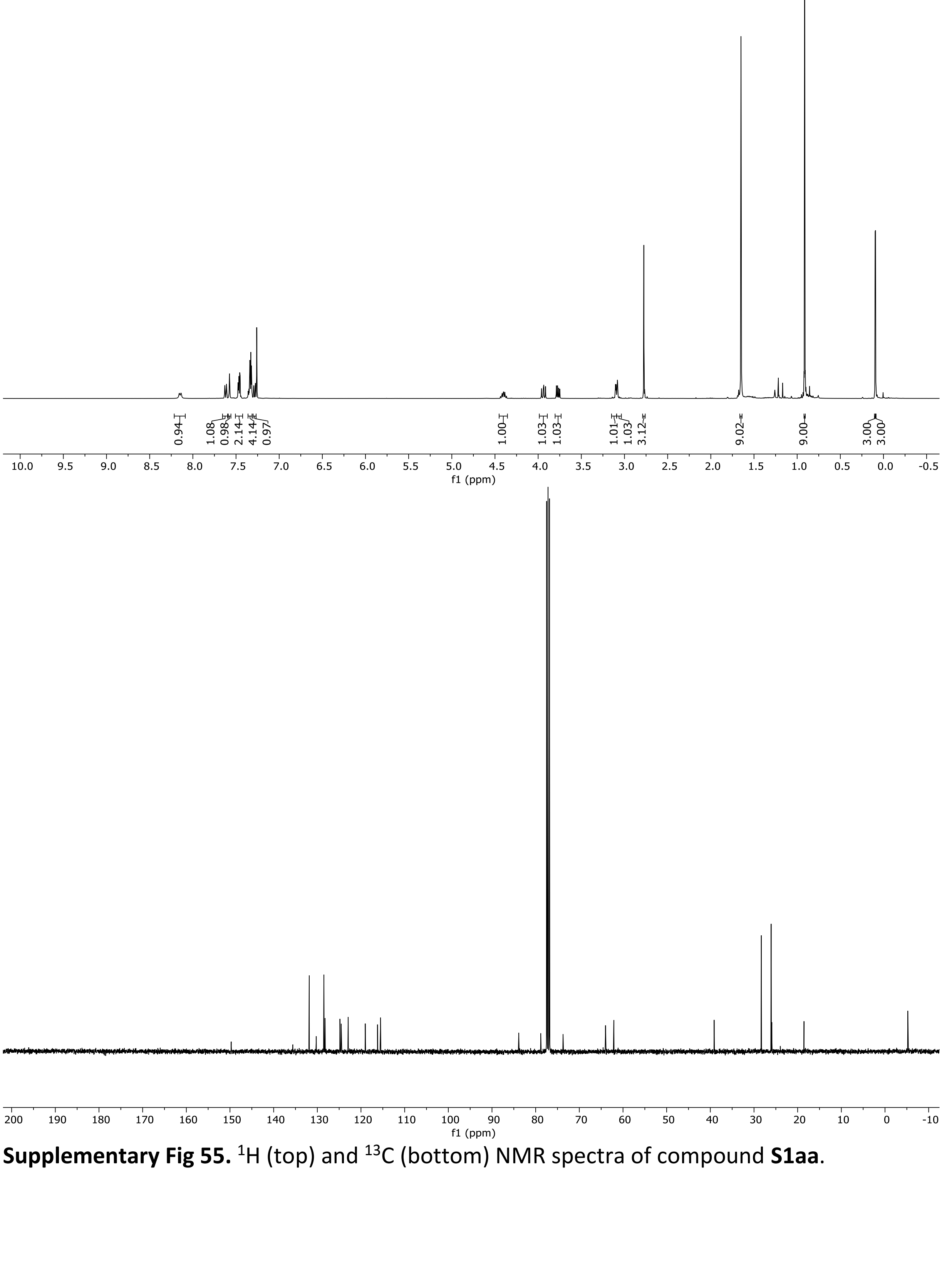

Supplementary Fig 55. ^{1}H (top) and ^{13}C (bottom) NMR spectra of compound **S1aa**.

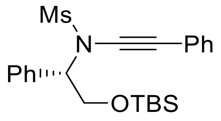

S1ab

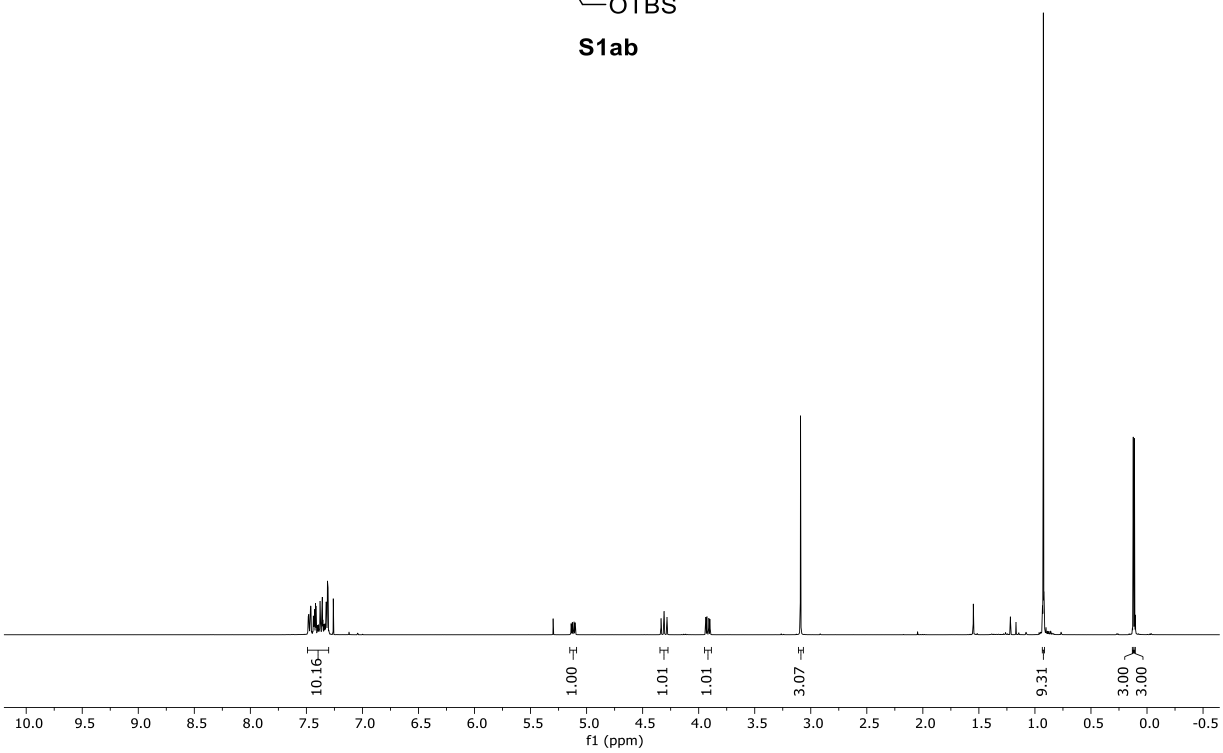

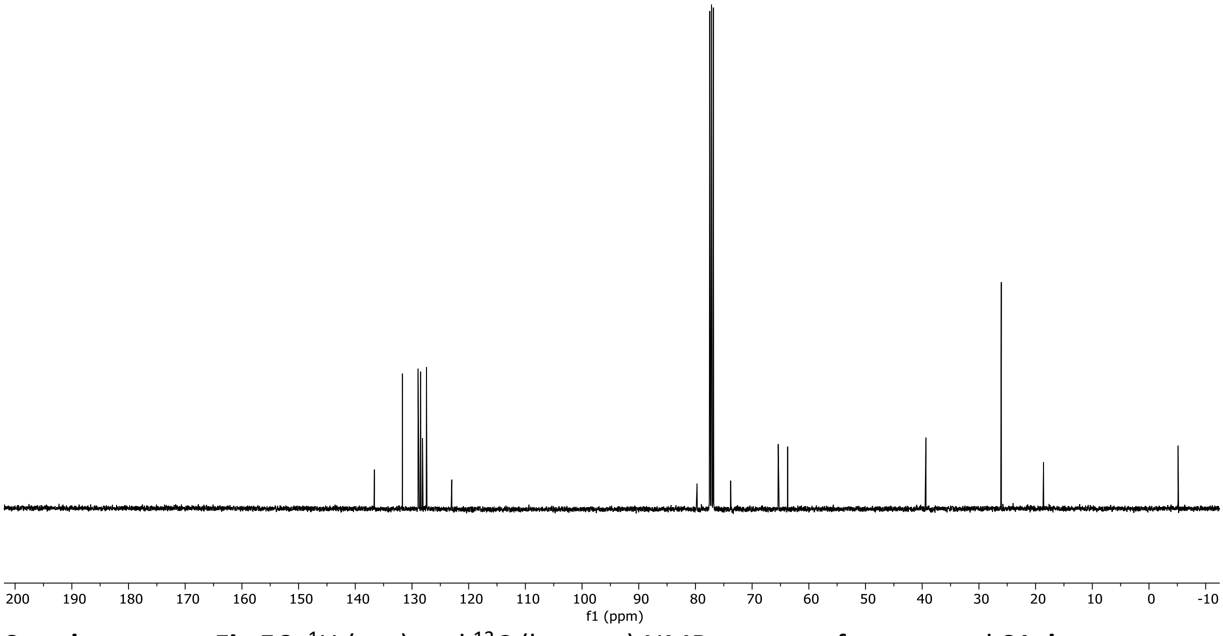

Supplementary Fig 56. ^{1}H (top) and ^{13}C (bottom) NMR spectra of compound **S1ab**.

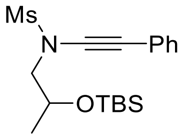

S1ac

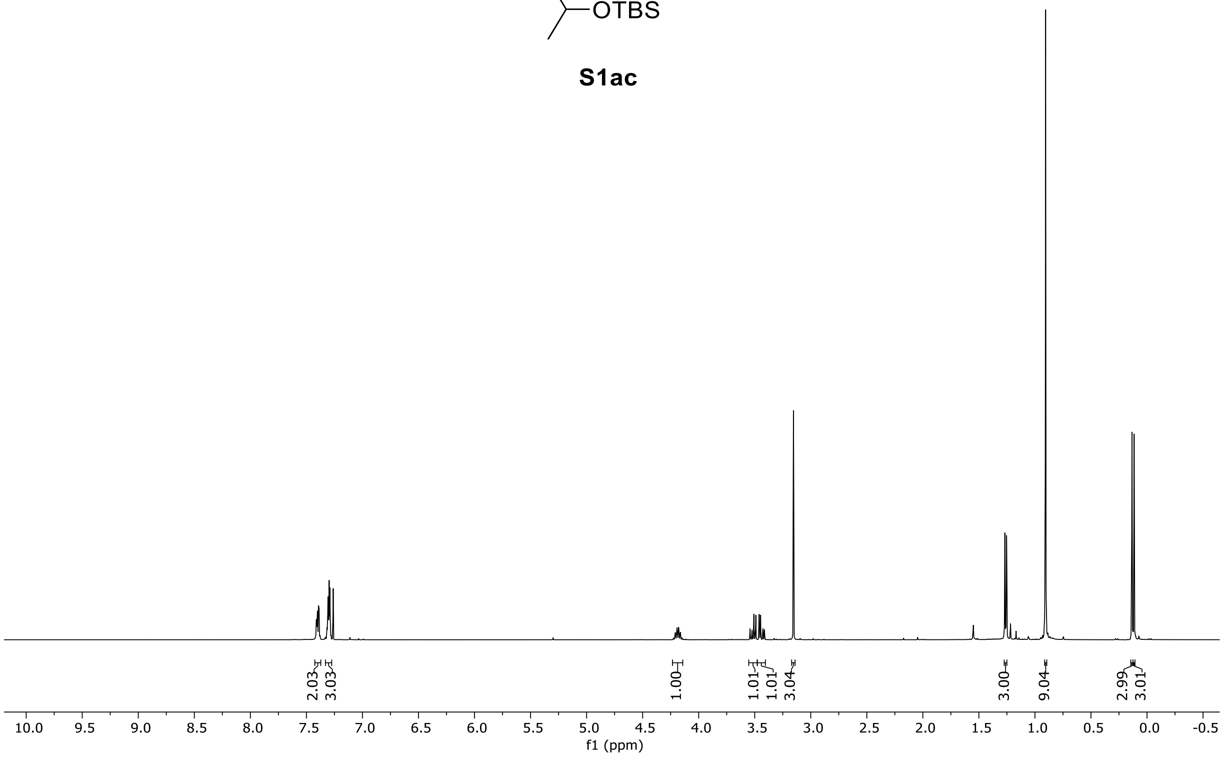

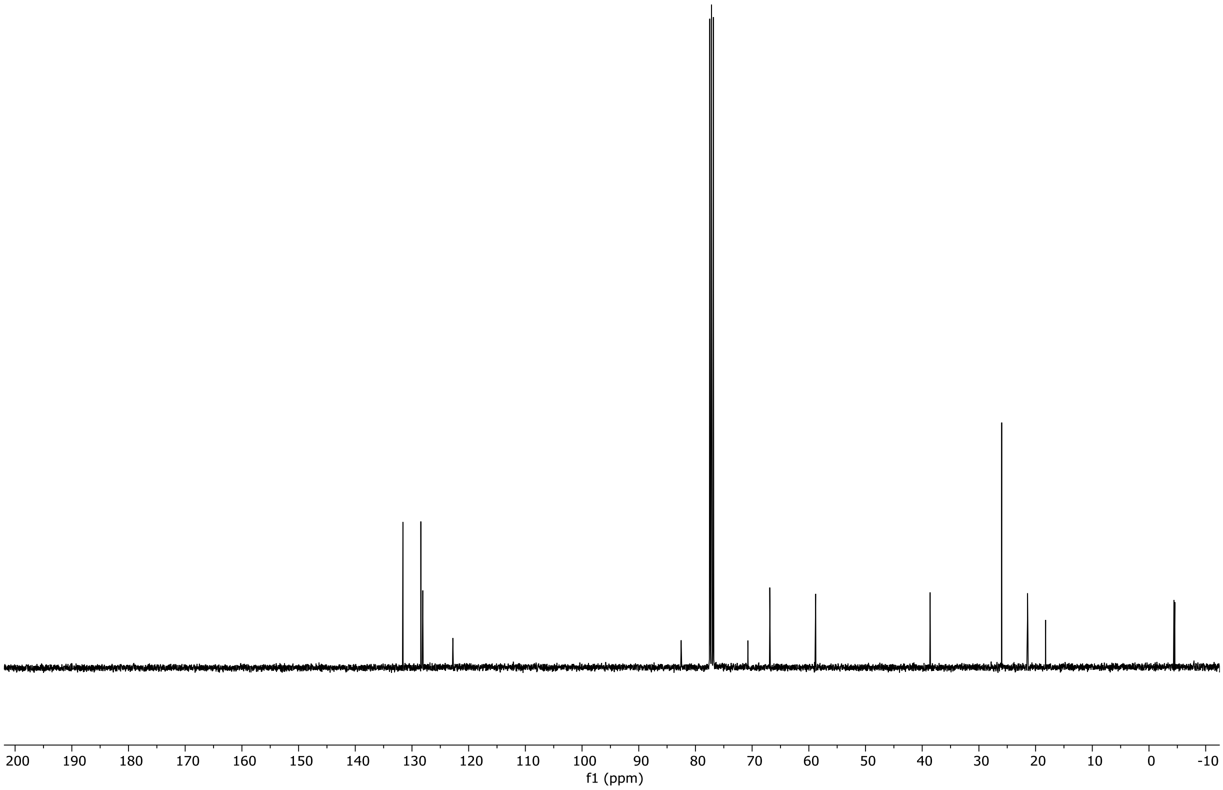

Supplementary Fig 57. ^{1}H (top) and ^{13}C (bottom) NMR spectra of compound **S1ac**.

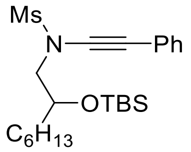

S1ad

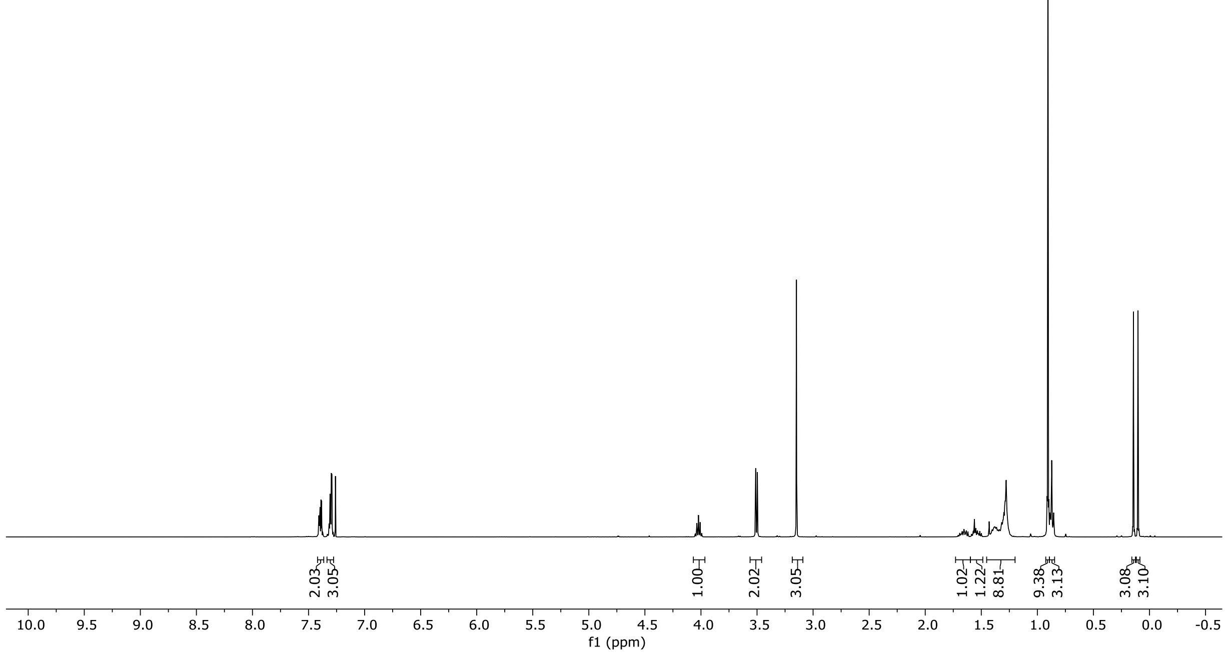

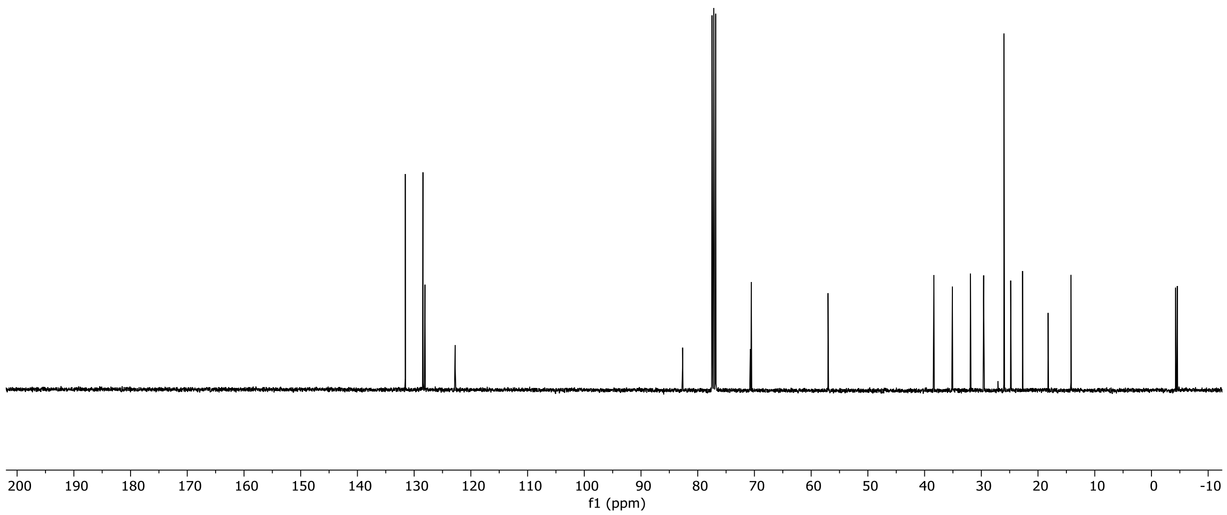

Supplementary Fig 58. ^{1}H (top) and ^{13}C (bottom) NMR spectra of compound **S1ad**.

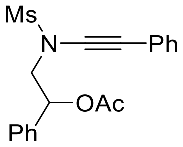

S1ae

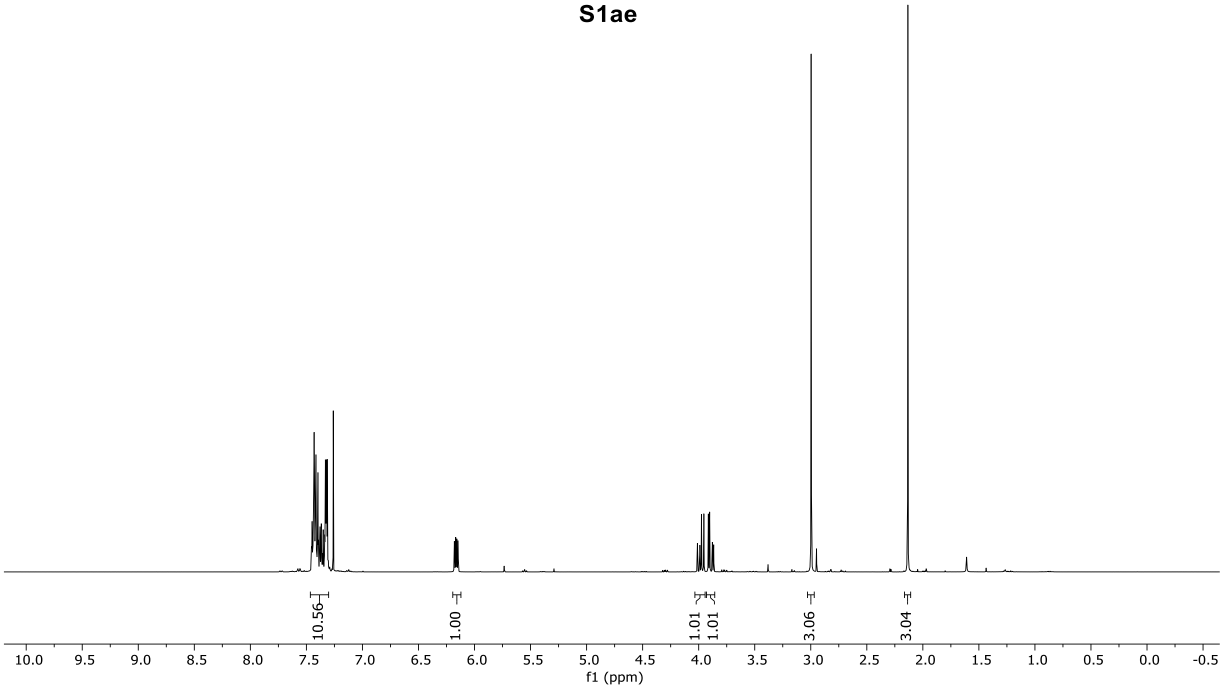

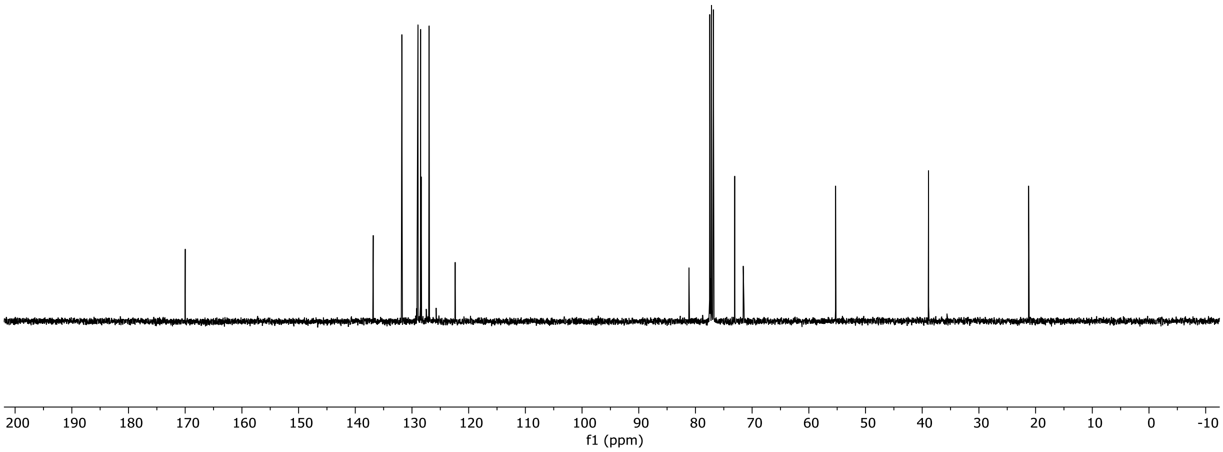

Supplementary Fig 59. ^{1}H (top) and ^{13}C (bottom) NMR spectra of compound **S1ae**.

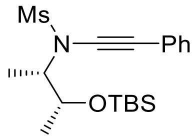

S1af

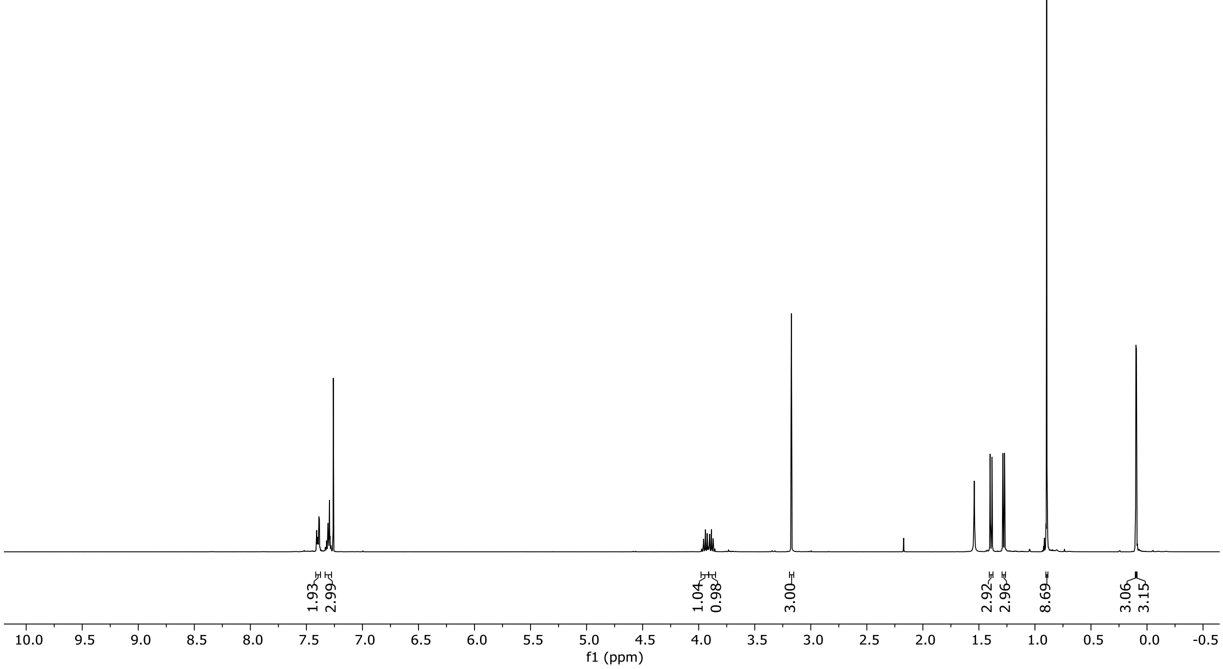

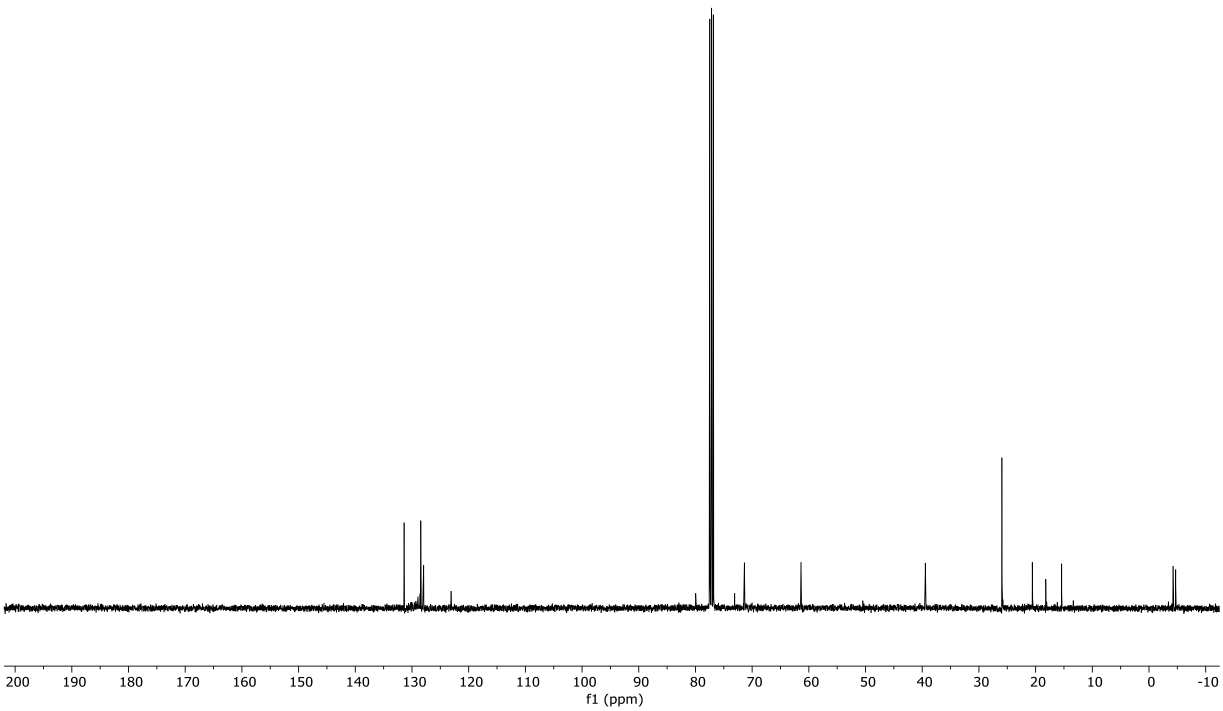

Supplementary Fig 60. ^{1}H (top) and ^{13}C (bottom) NMR spectra of compound **S1af**.

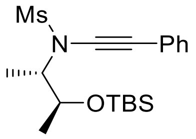

S1af'

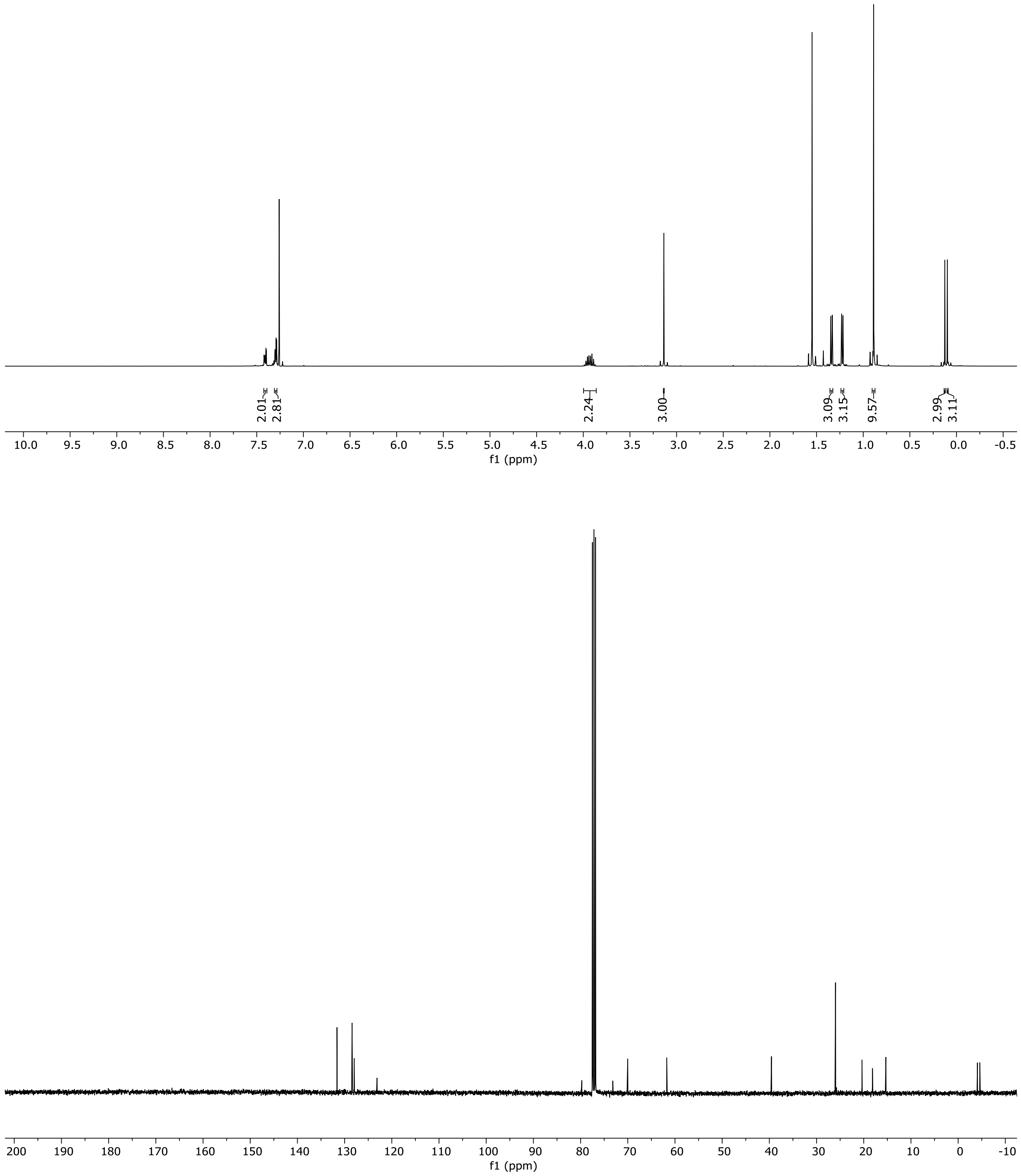

Supplementary Fig 61. ^{1}H (top) and ^{13}C (bottom) NMR spectra of compound **S1af'**.

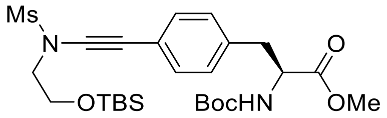

S1ag

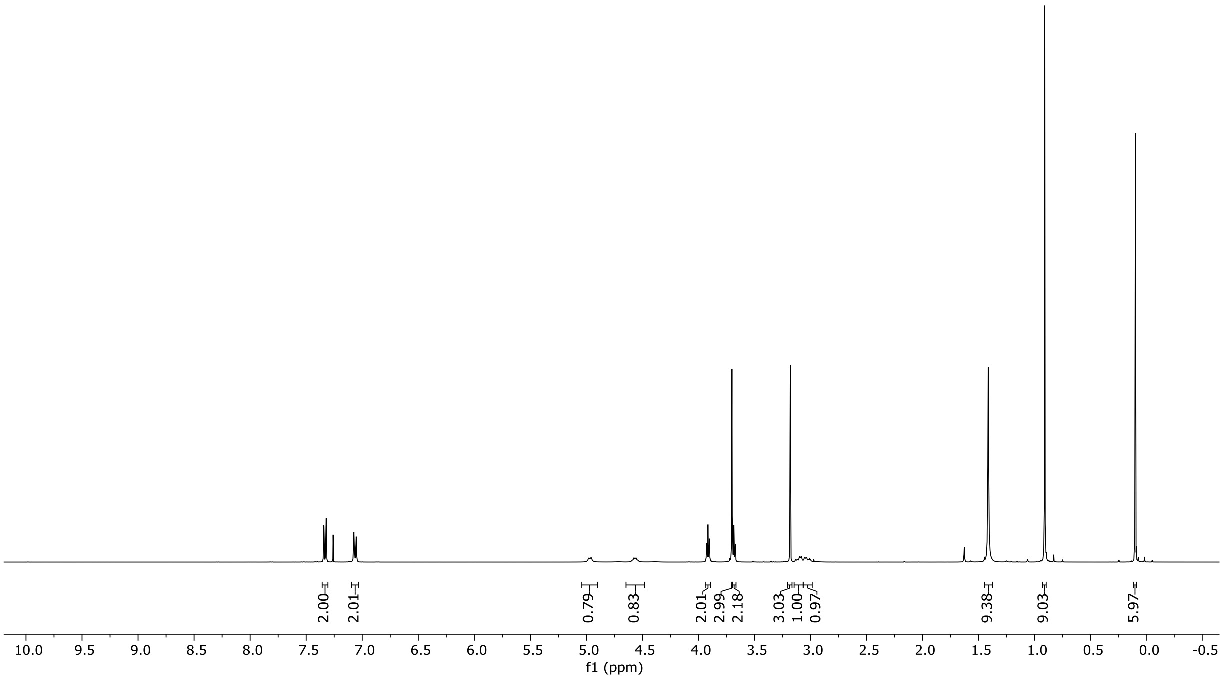

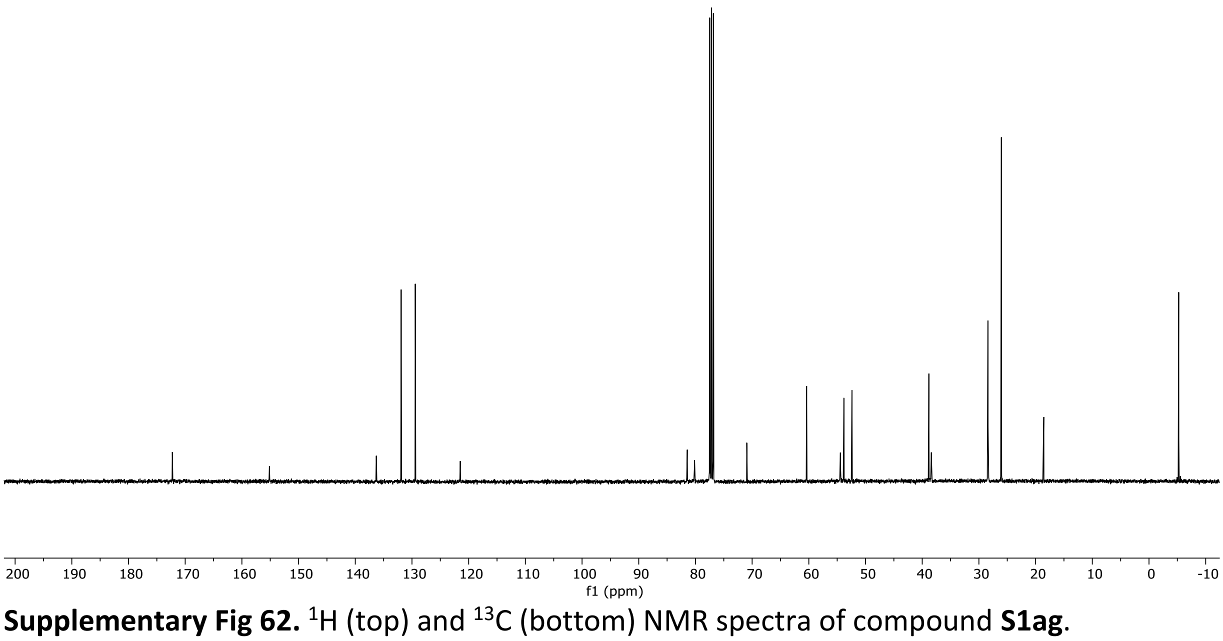

Supplementary Fig 62. ^{1}H (top) and ^{13}C (bottom) NMR spectra of compound **S1ag**.

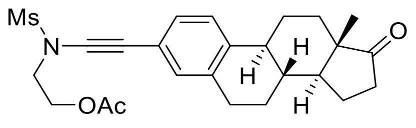

S1ah

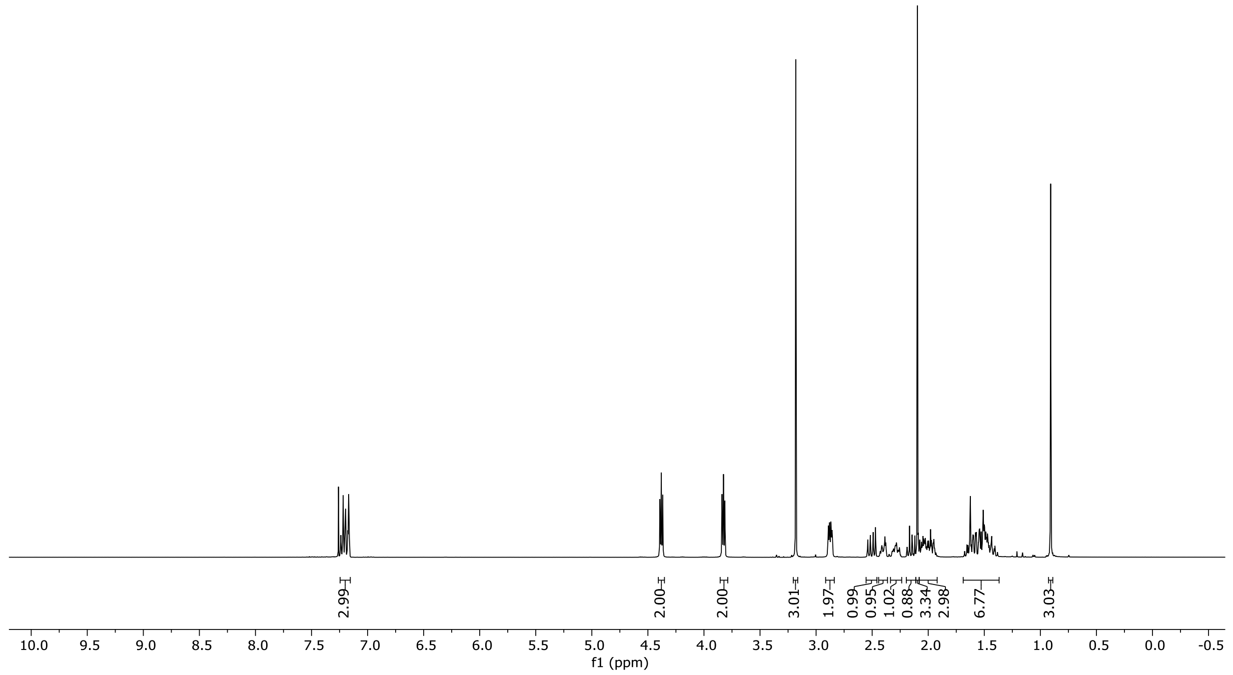

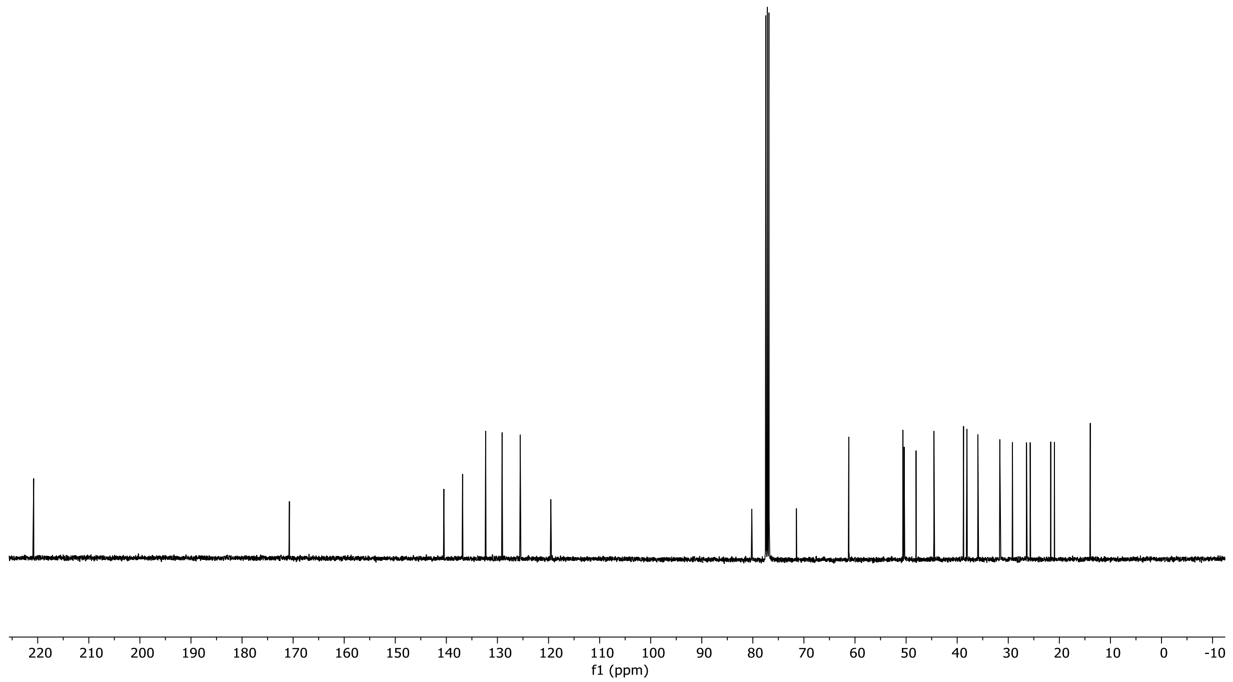

Supplementary Fig 63. ^{1}H (top) and ^{13}C (bottom) NMR spectra of compound **S1ah**.

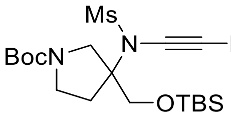

S1ai

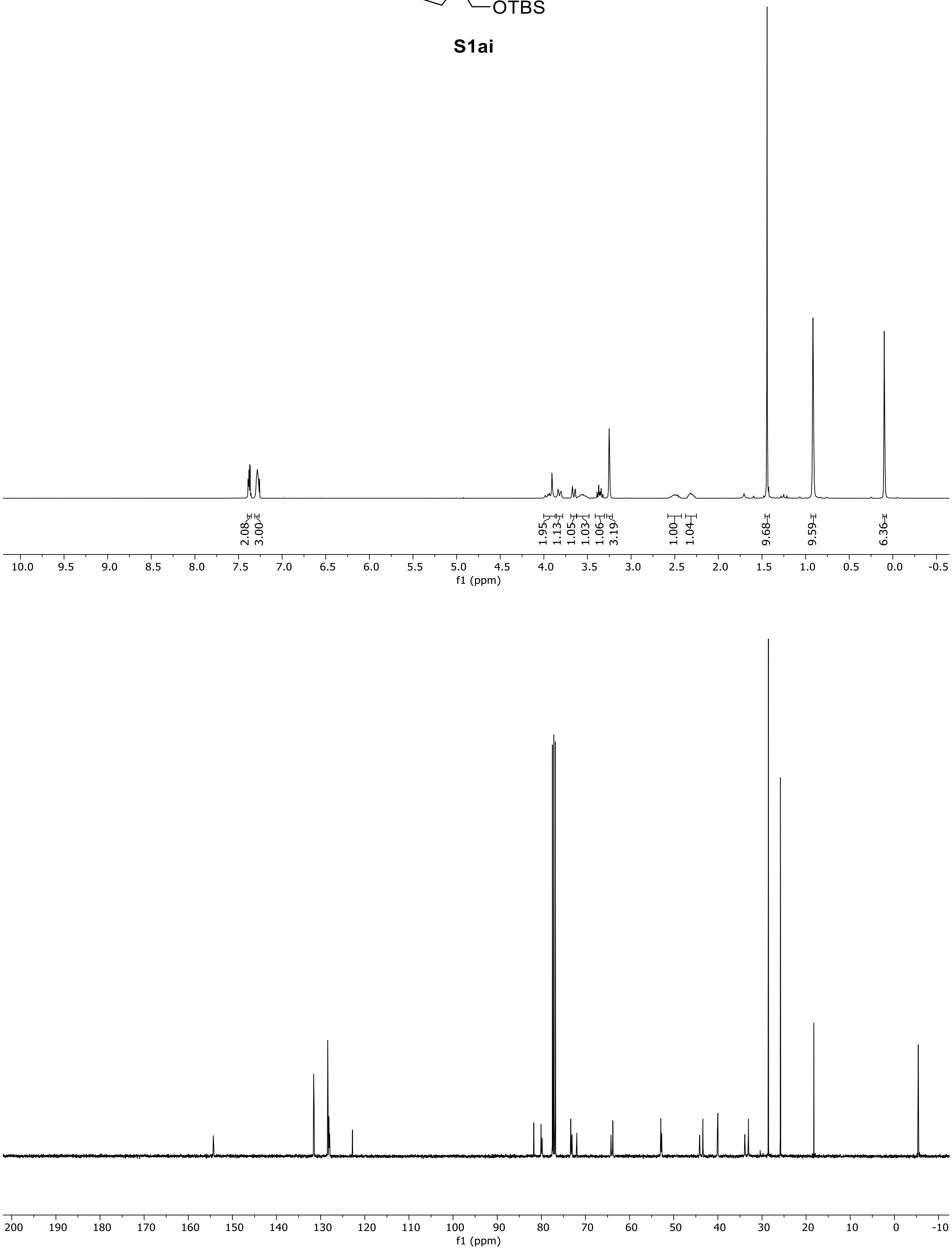

Supplementary Fig 64. ^{1}H (top) and ^{13}C (bottom) NMR spectra of compound **S1ai**.

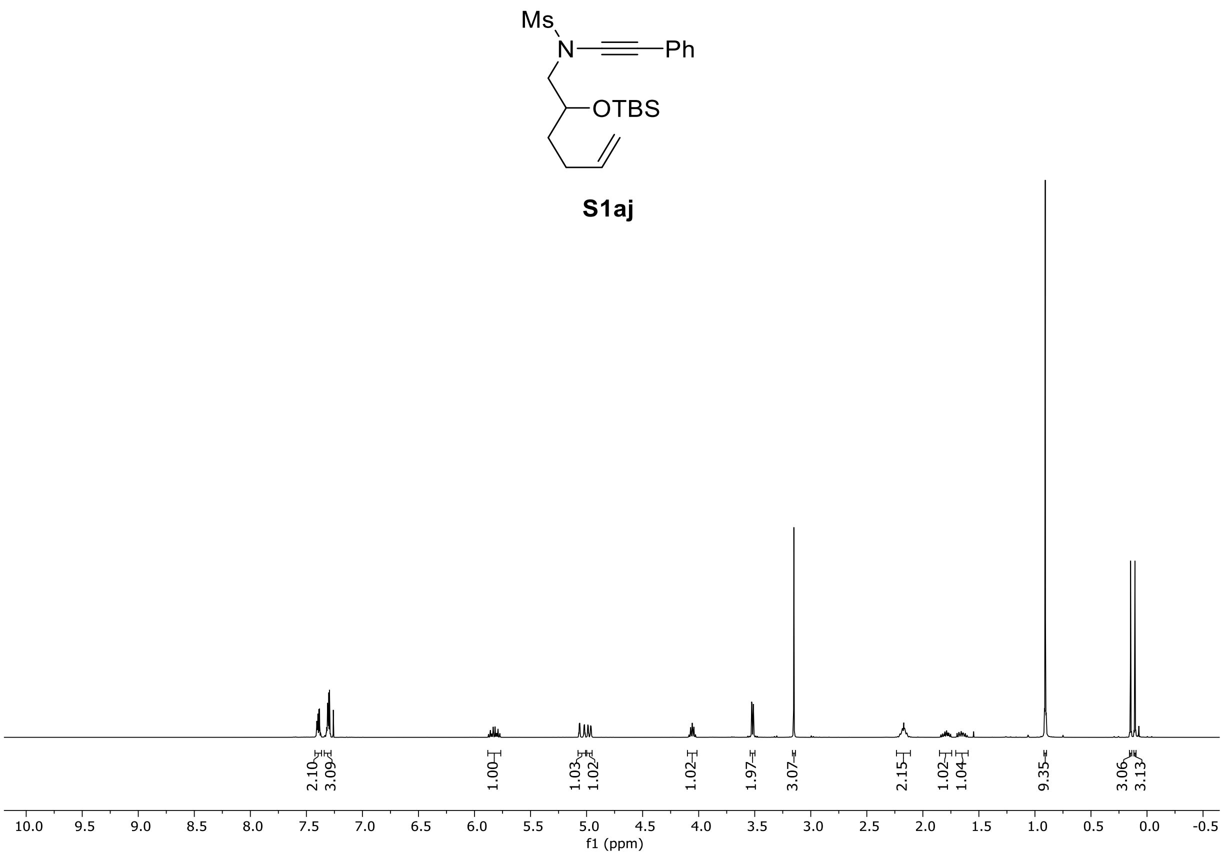

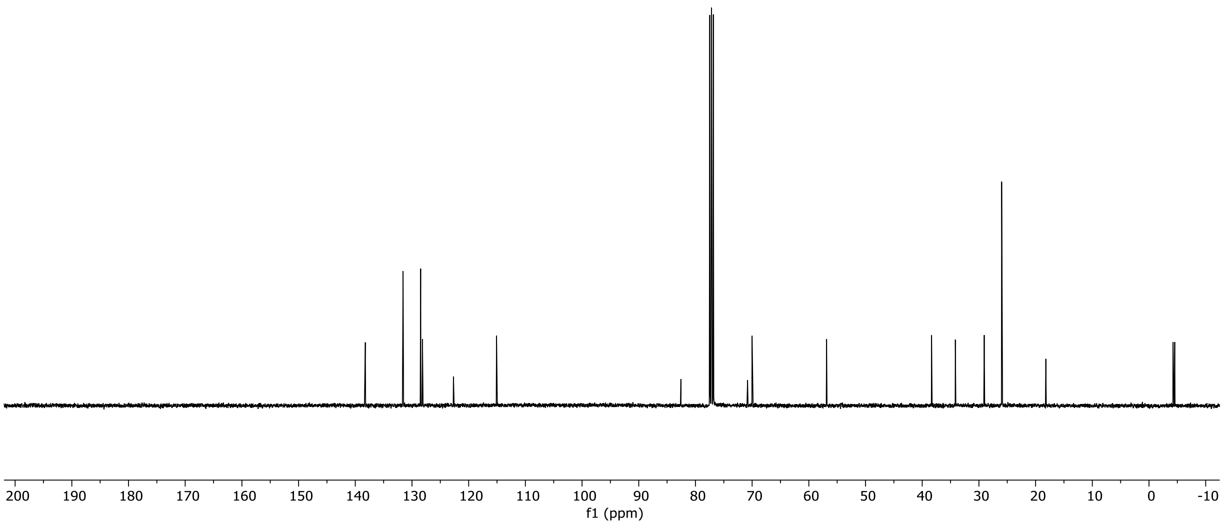

Supplementary Fig 65. ^{1}H (top) and ^{13}C (bottom) NMR spectra of compound **S1aj**.

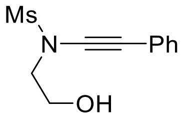

S2a

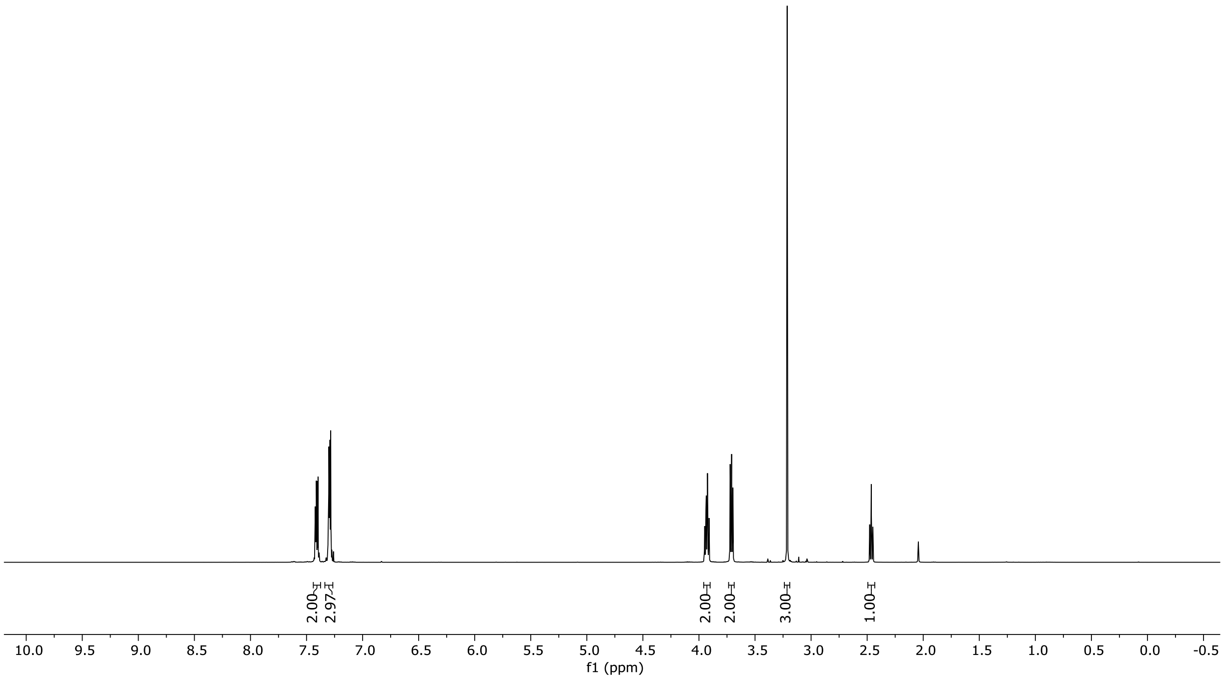

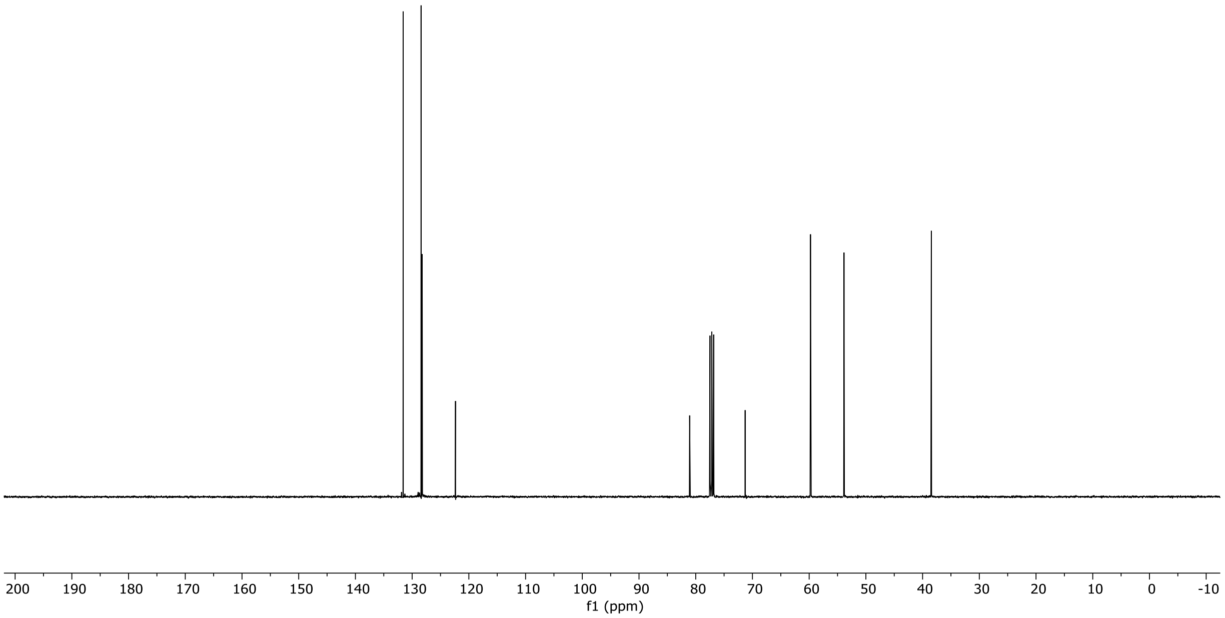

Supplementary Fig 66. ^{1}H (top) and ^{13}C (bottom) NMR spectra of compound **S2a**.

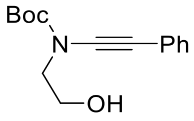

S2b

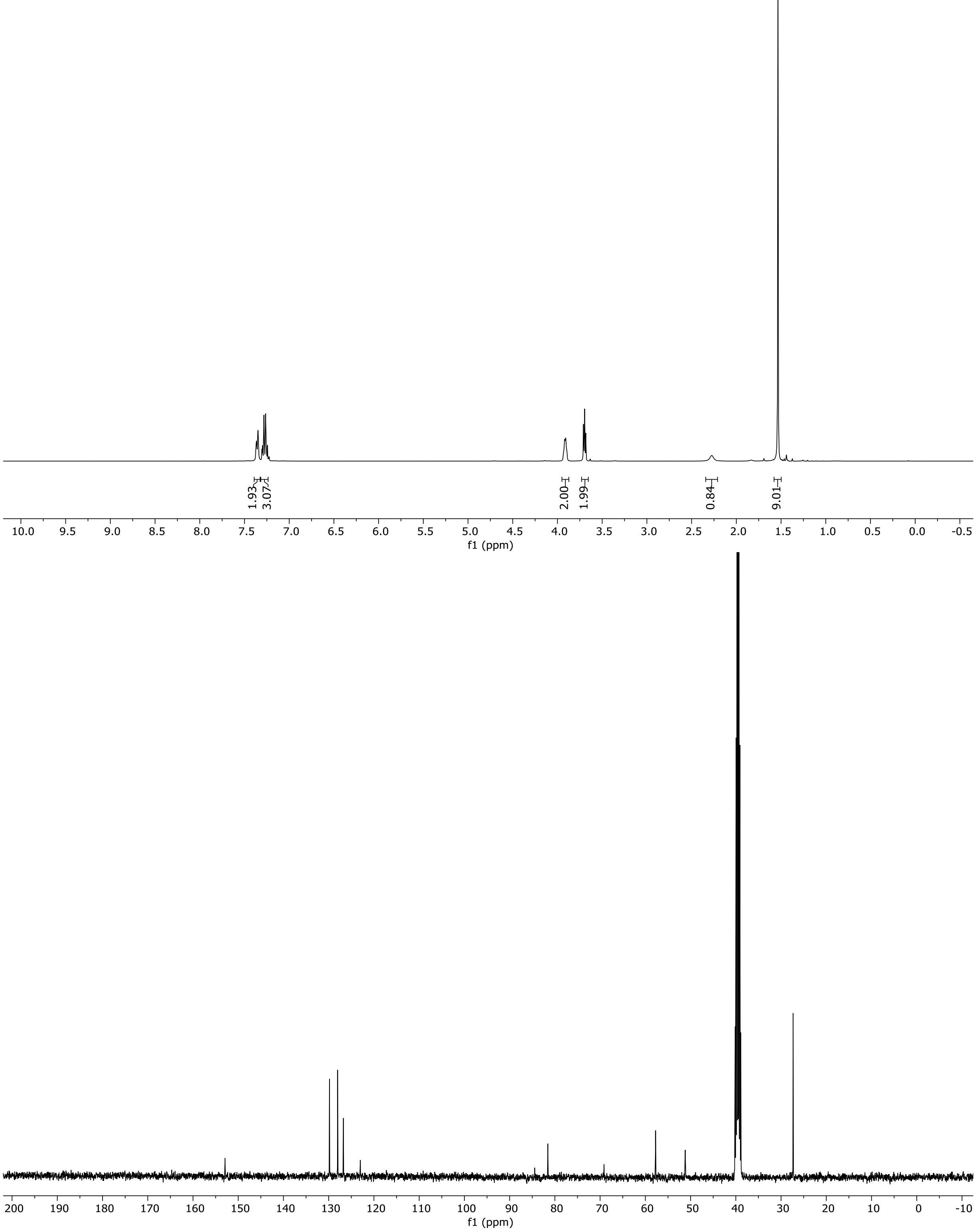

Supplementary Fig 67. ^{1}H (top) and ^{13}C (bottom) NMR spectra of compound **S2b**.

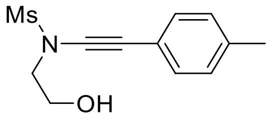

S2c

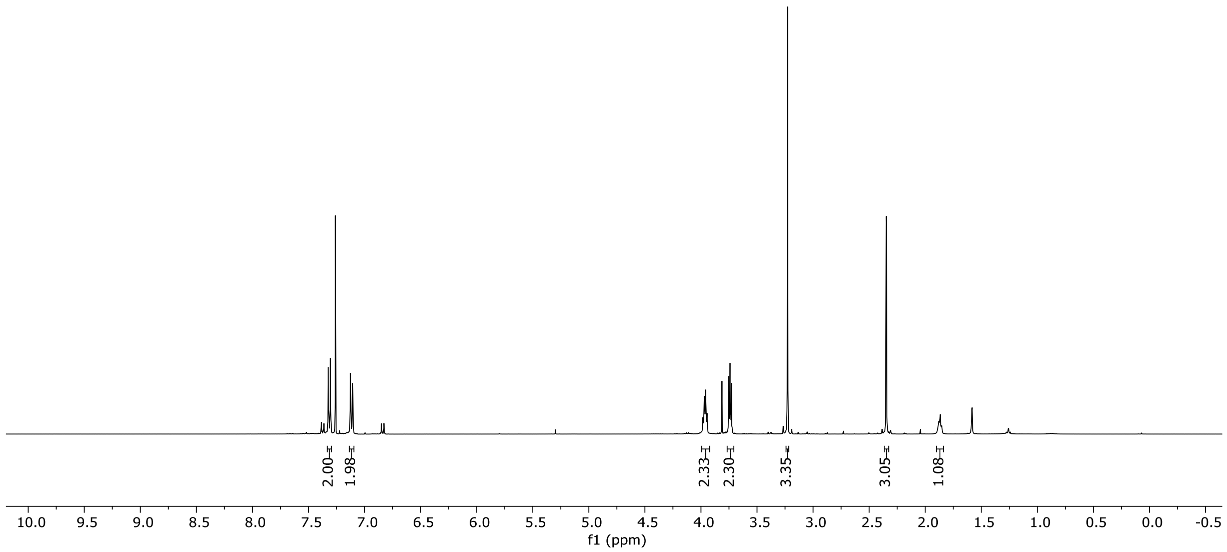

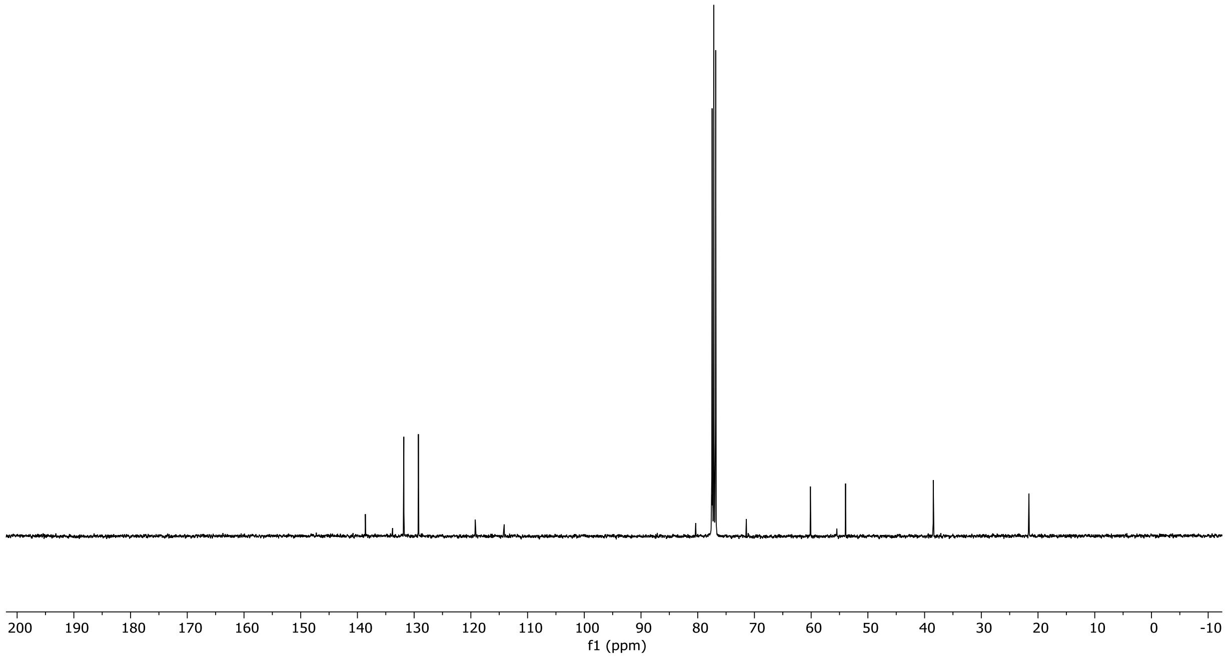

Supplementary Fig 68. ^{1}H (top) and ^{13}C (bottom) NMR spectra of compound **S2c**.

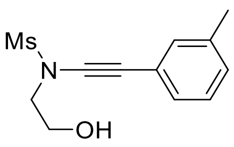

S2d

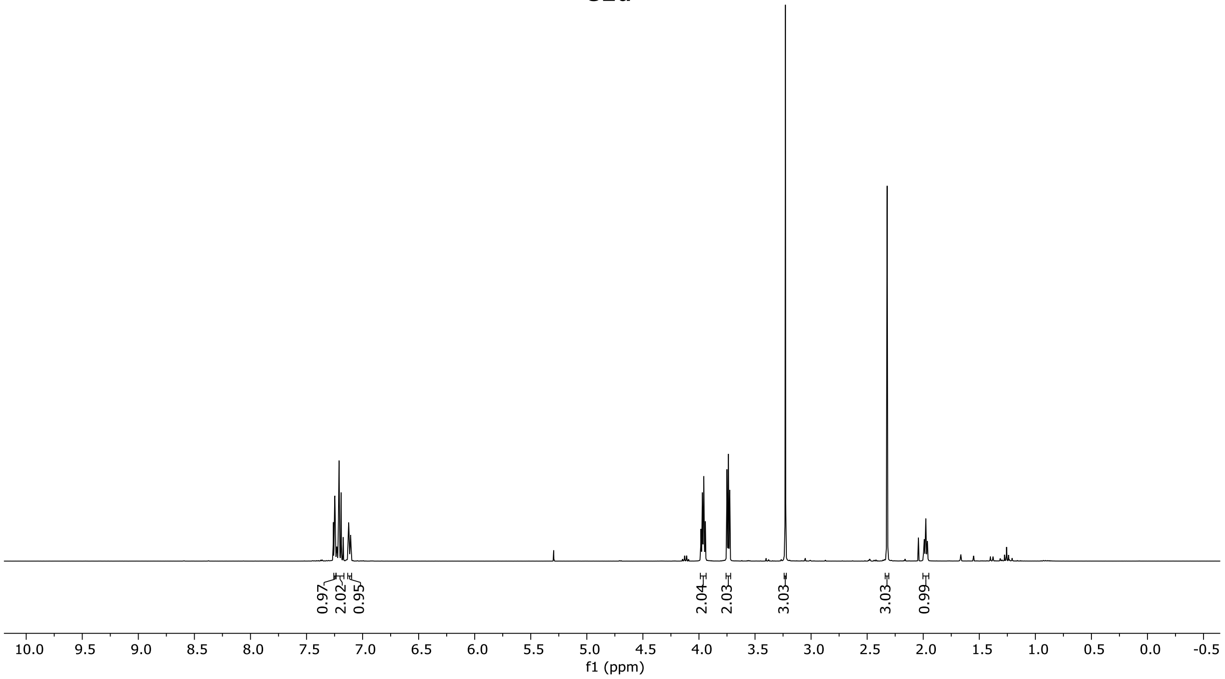

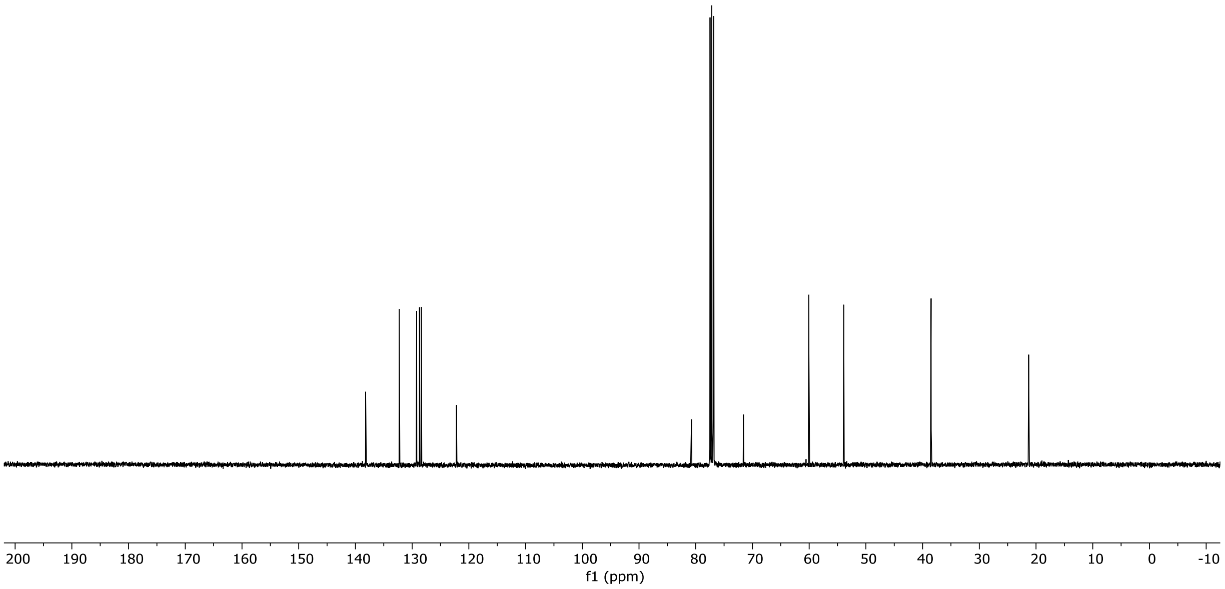

Supplementary Fig 69. ^{1}H (top) and ^{13}C (bottom) NMR spectra of compound **S2d**.

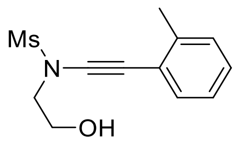

S2e

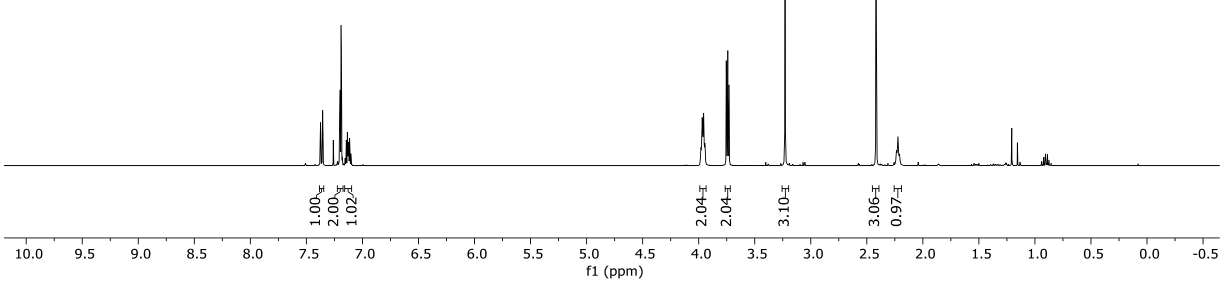

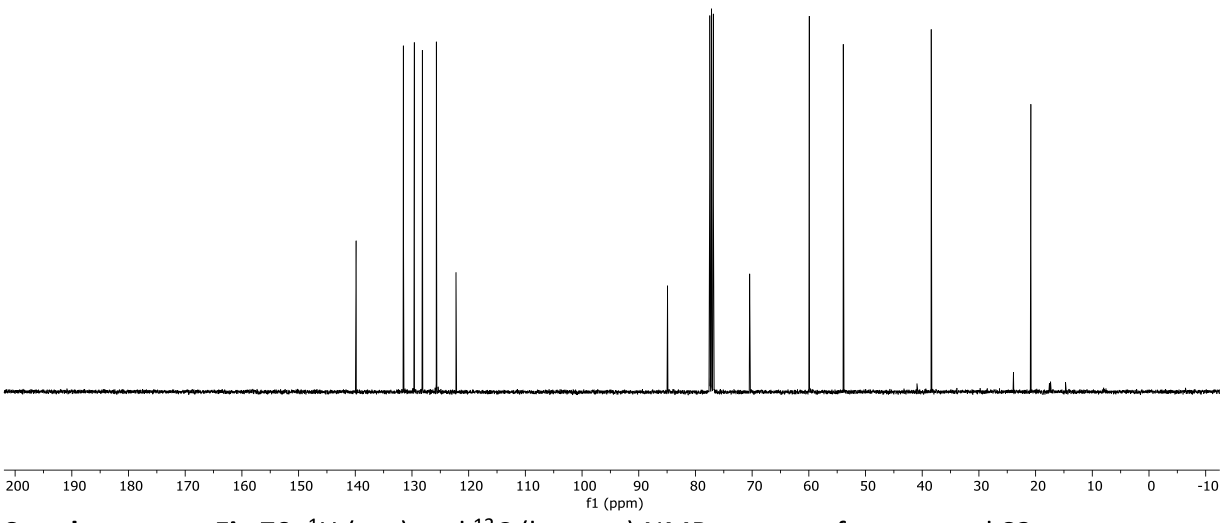

Supplementary Fig 70. ^{1}H (top) and ^{13}C (bottom) NMR spectra of compound **S2e**.

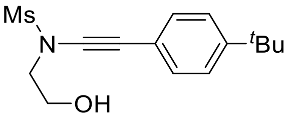

S2f

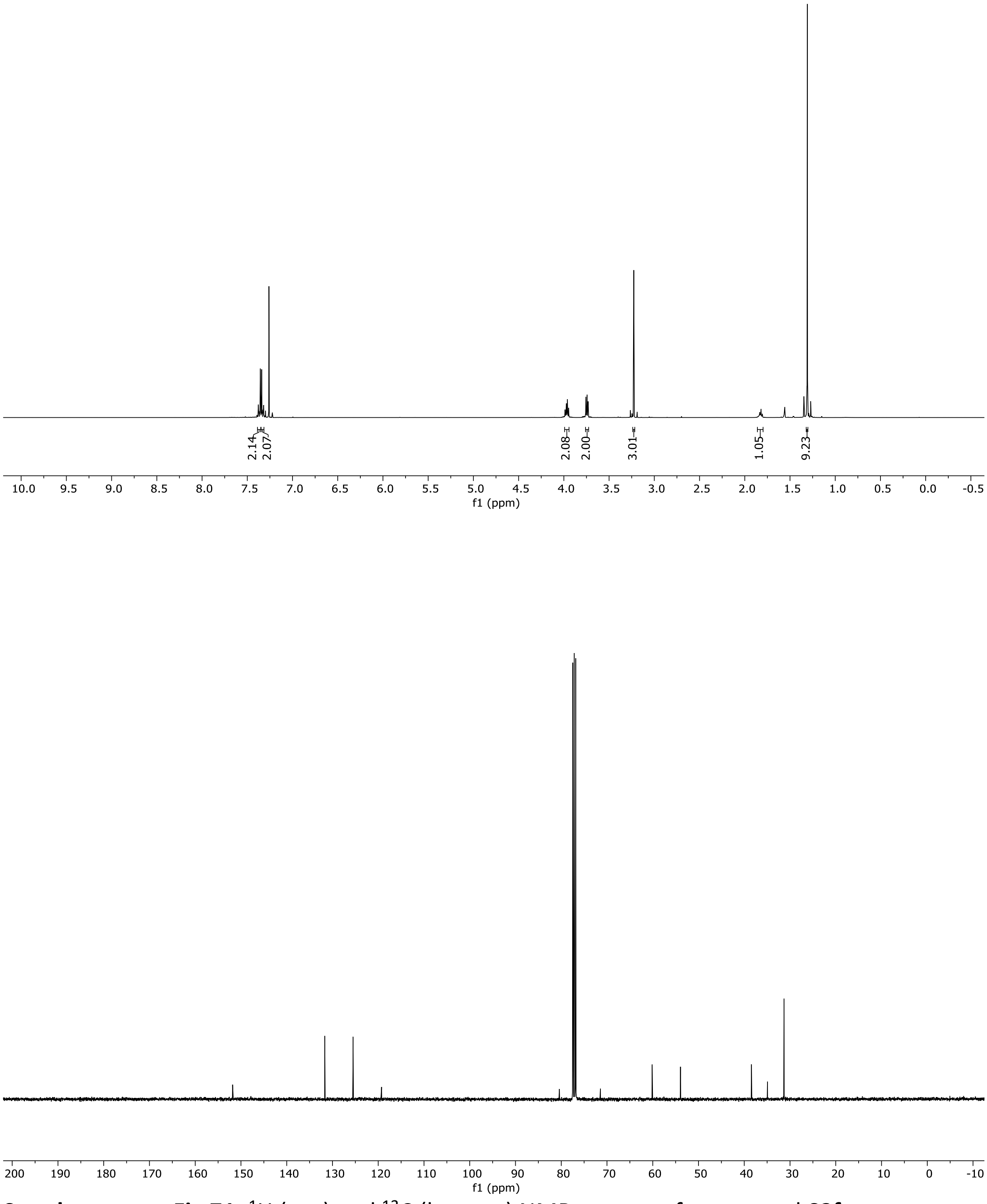

Supplementary Fig 71. ^{1}H (top) and ^{13}C (bottom) NMR spectra of compound **S2f**.

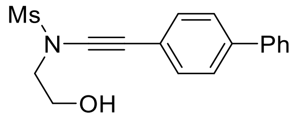

S2g

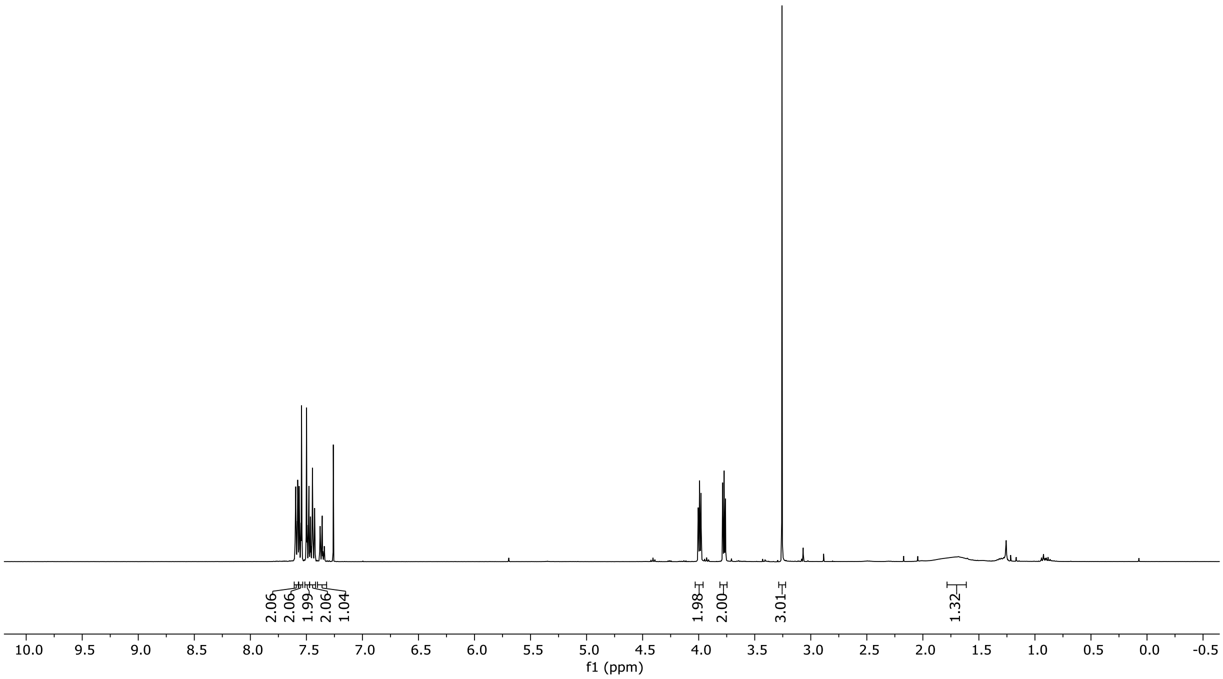

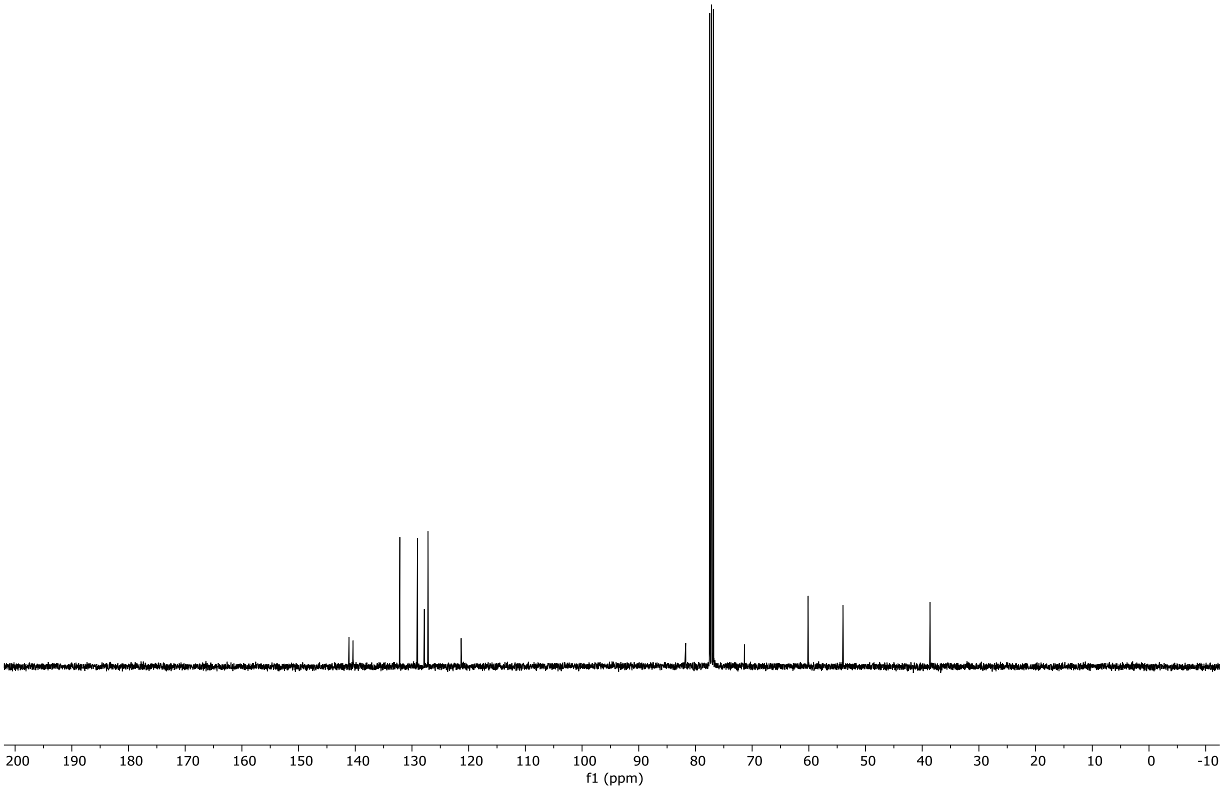

Supplementary Fig 72. ^{1}H (top) and ^{13}C (bottom) NMR spectra of compound **S2g**.

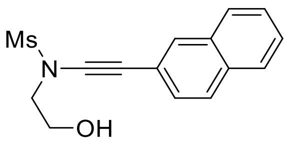

S2h

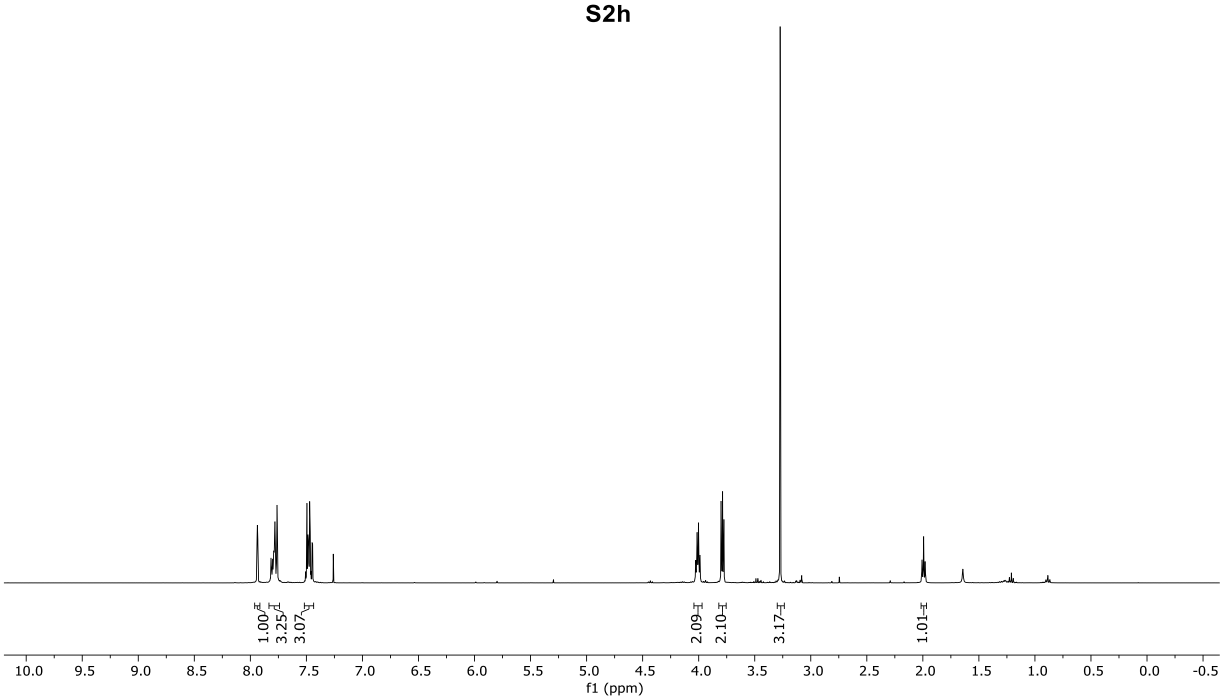

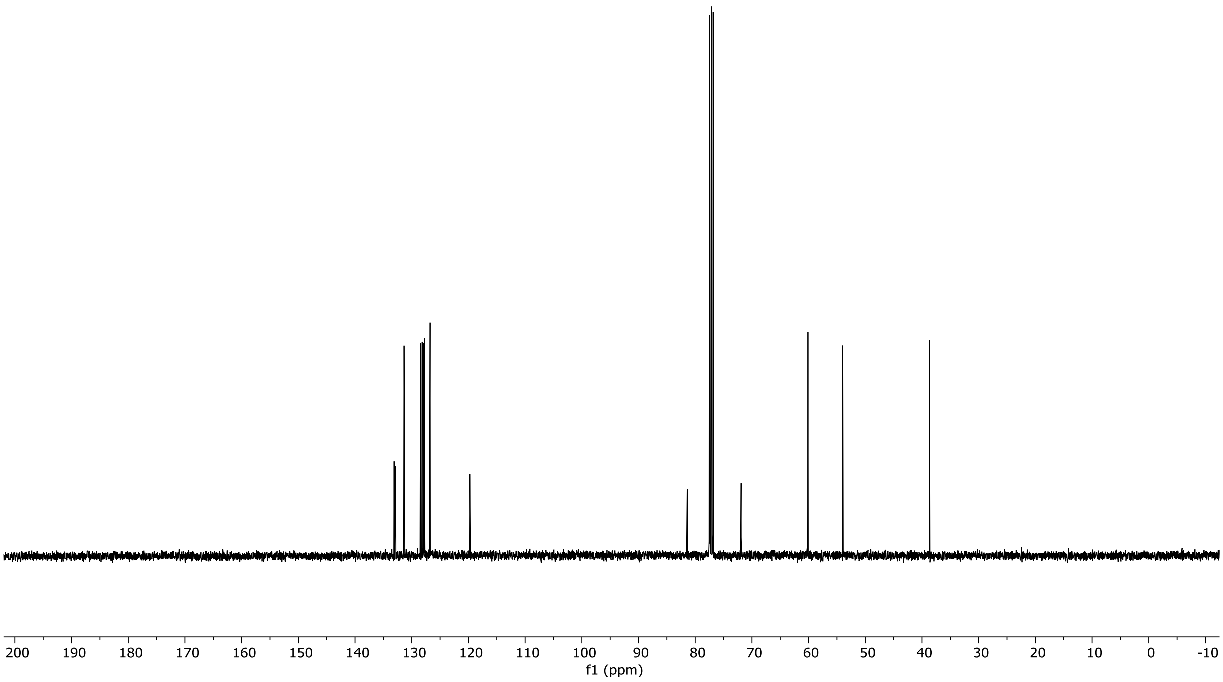

Supplementary Fig 73. ^{1}H (top) and ^{13}C (bottom) NMR spectra of compound **S2h**.

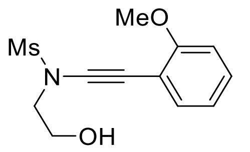

S2i

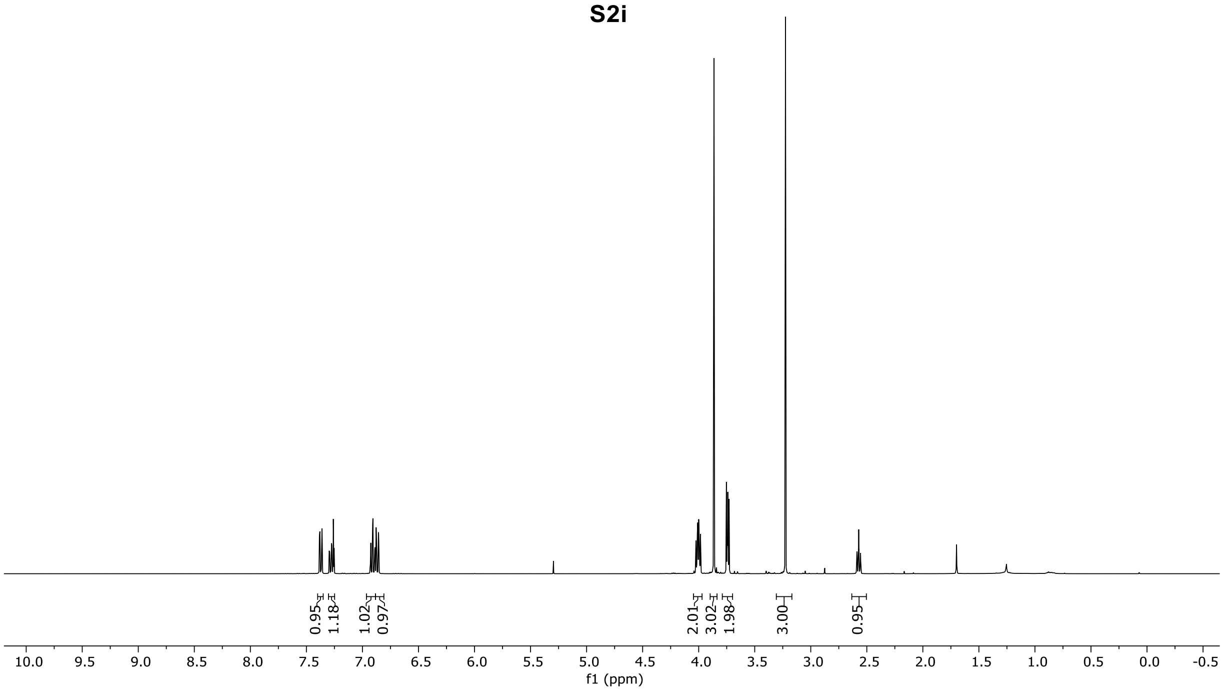

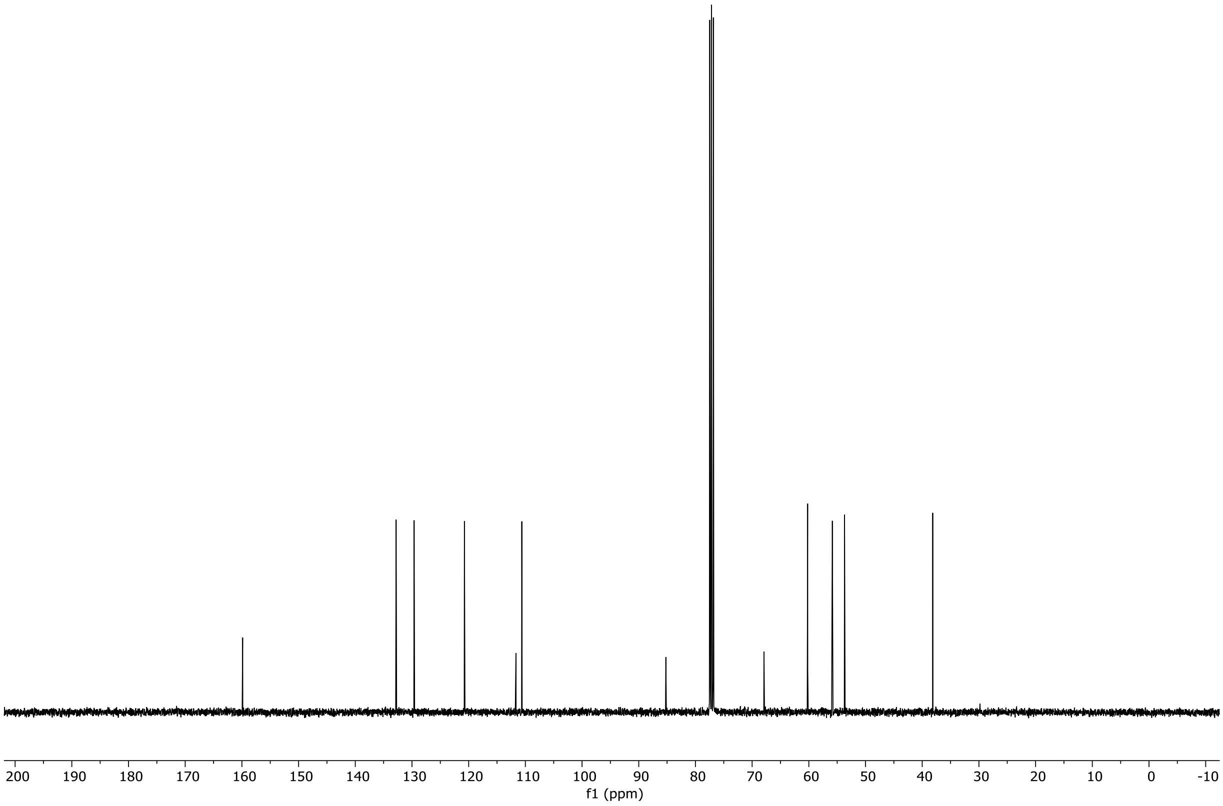

Supplementary Fig 74. ^{1}H (top) and ^{13}C (bottom) NMR spectra of compound **S2i**.

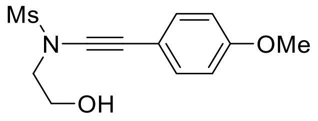

S2j

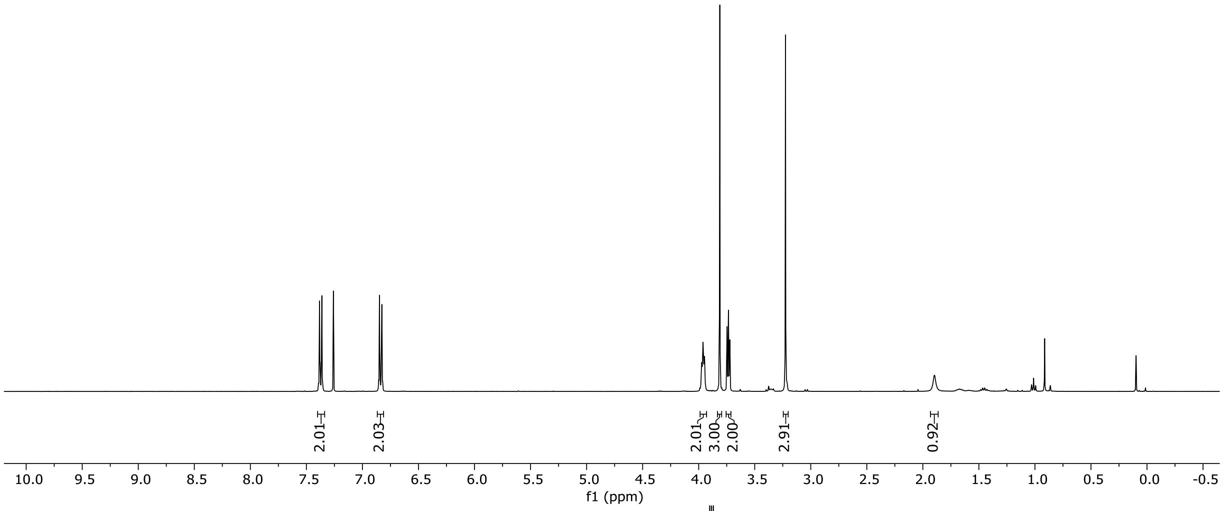

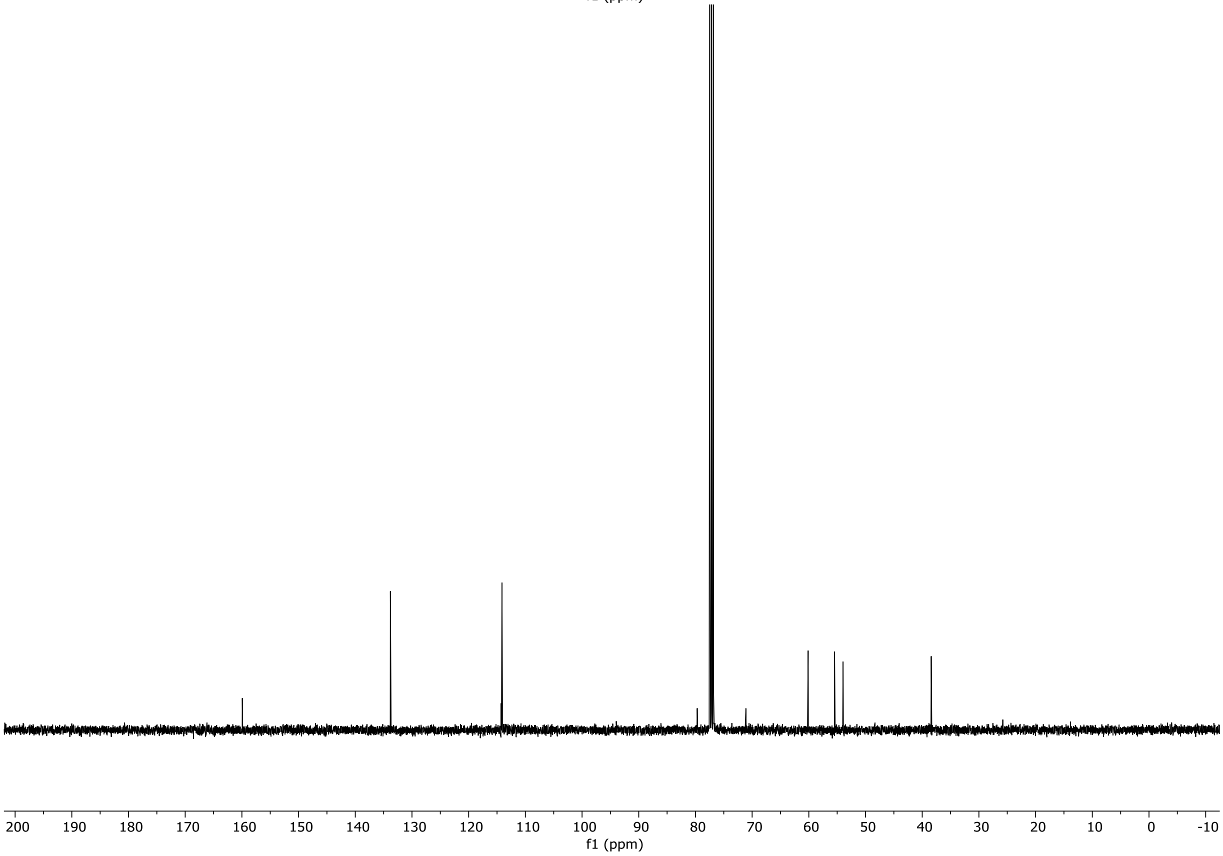

Supplementary Fig 75. ^{1}H (top) and ^{13}C (bottom) NMR spectra of compound **S2j**.

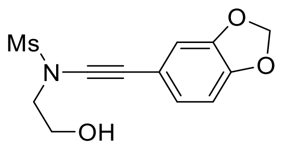

S2k

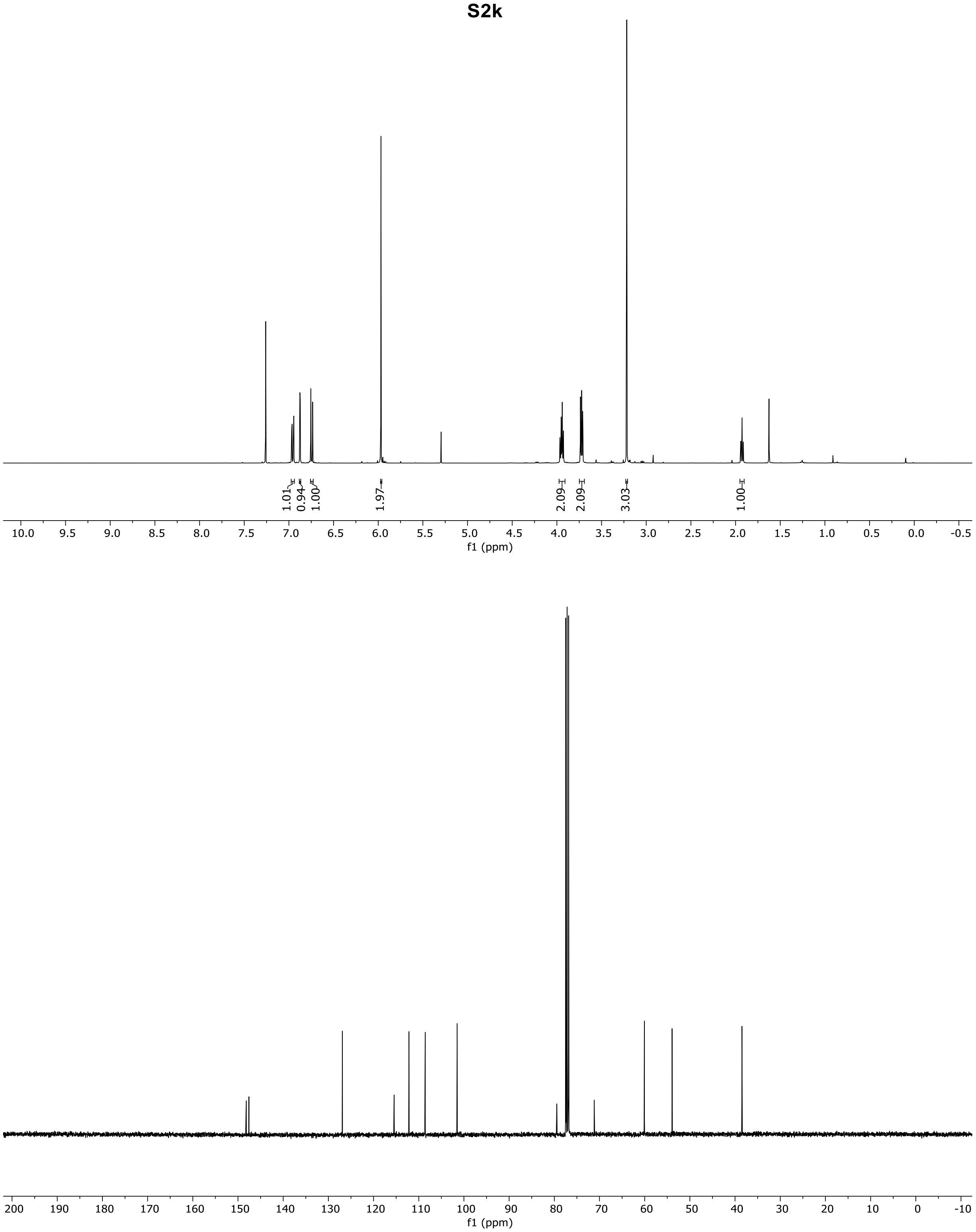

Supplementary Fig 76. ^{1}H (top) and ^{13}C (bottom) NMR spectra of compound **S2k**.

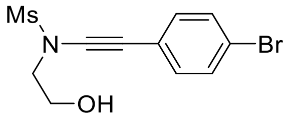

S2l

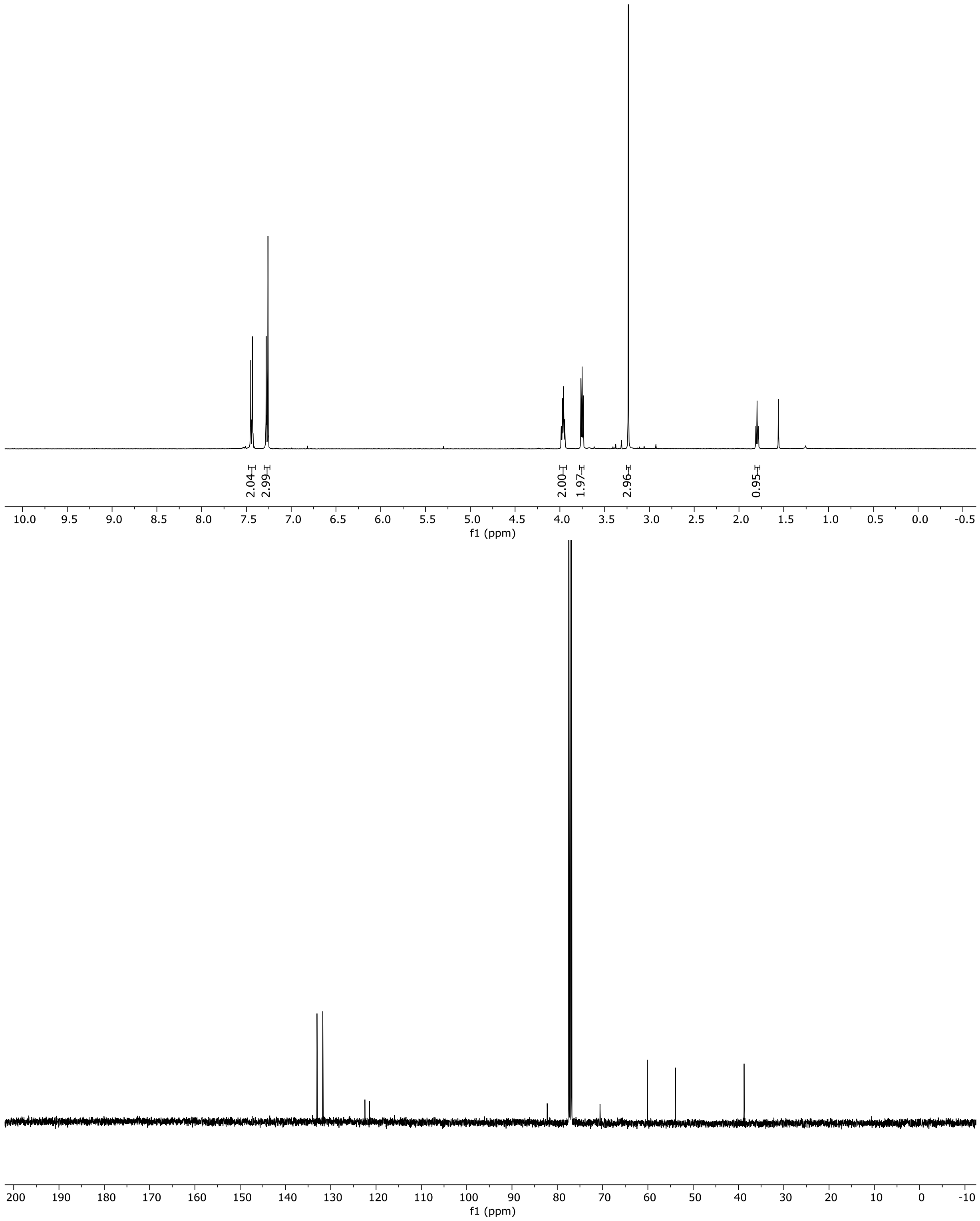

Supplementary Fig 77. ^{1}H (top) and ^{13}C (bottom) NMR spectra of compound **S2l**.

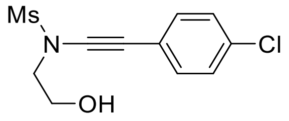

S2m

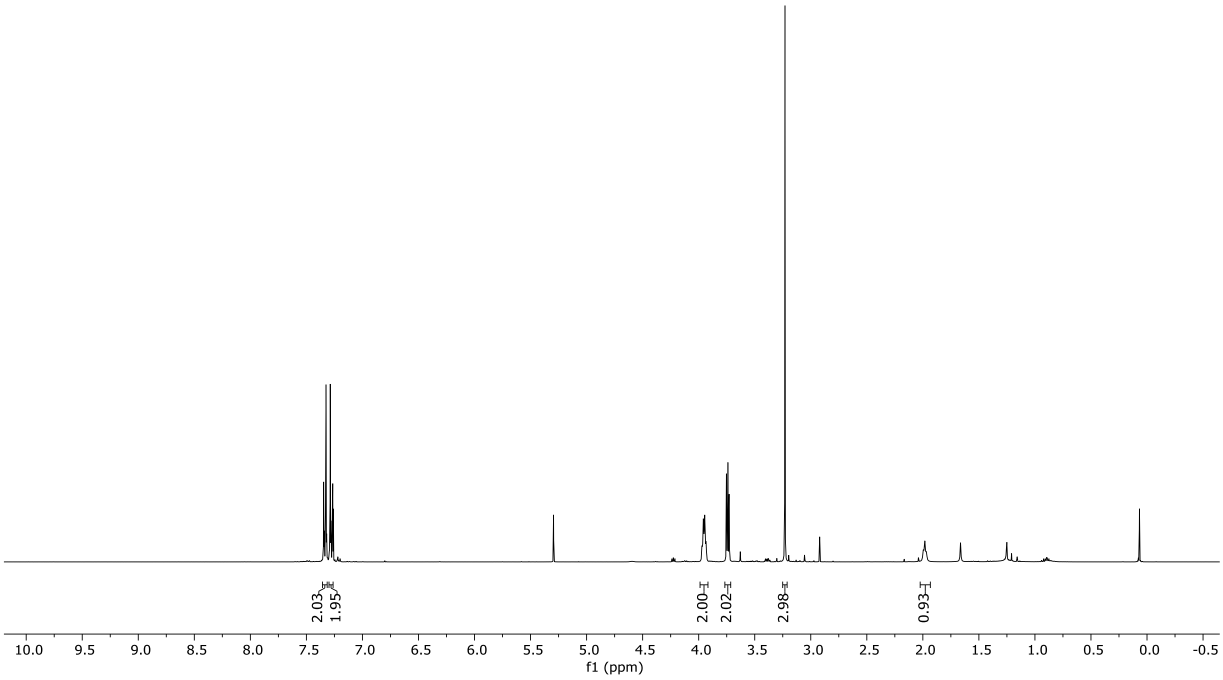

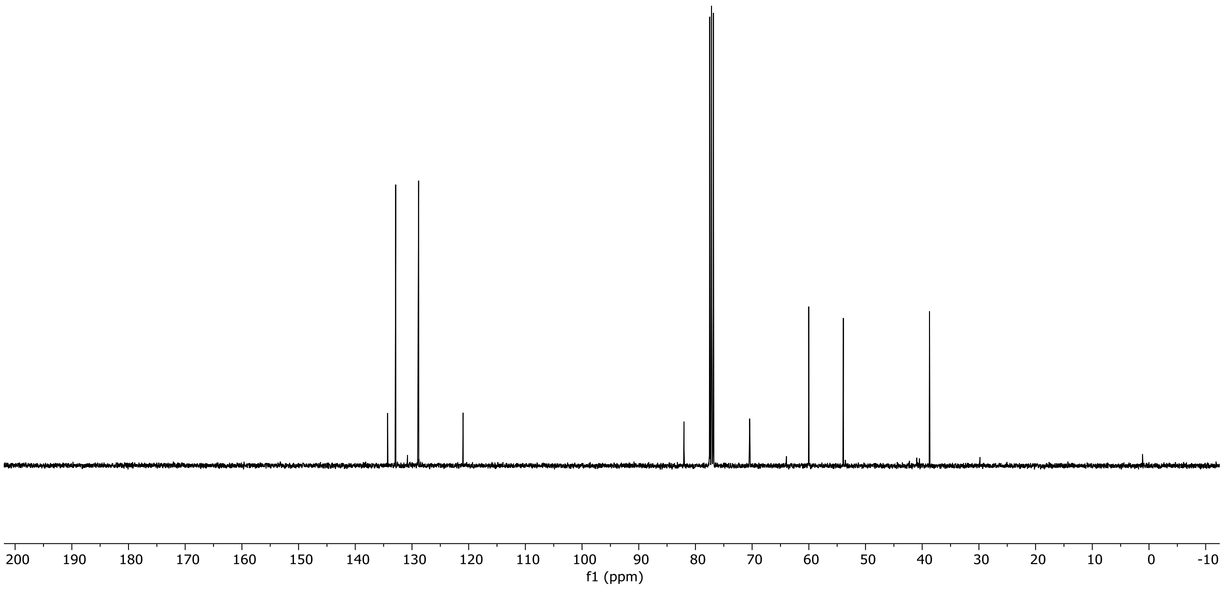

Supplementary Fig 78. ^{1}H (top) and ^{13}C (bottom) NMR spectra of compound **S2m**.

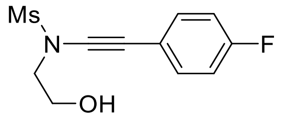

S2n

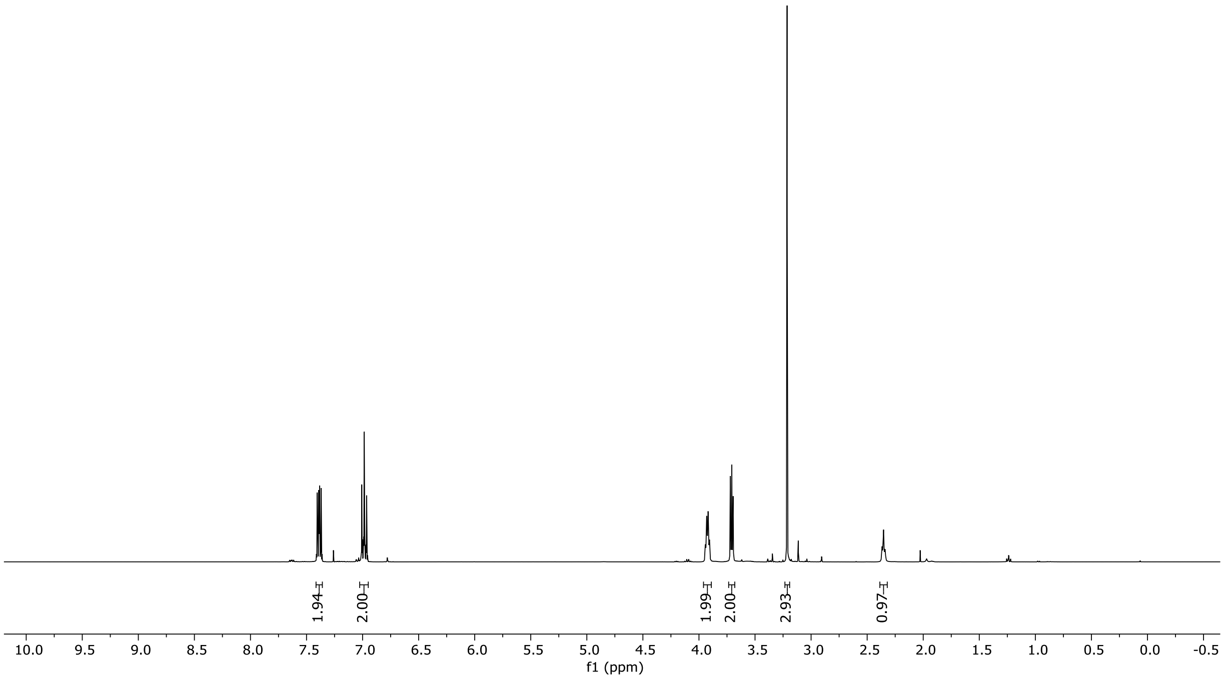

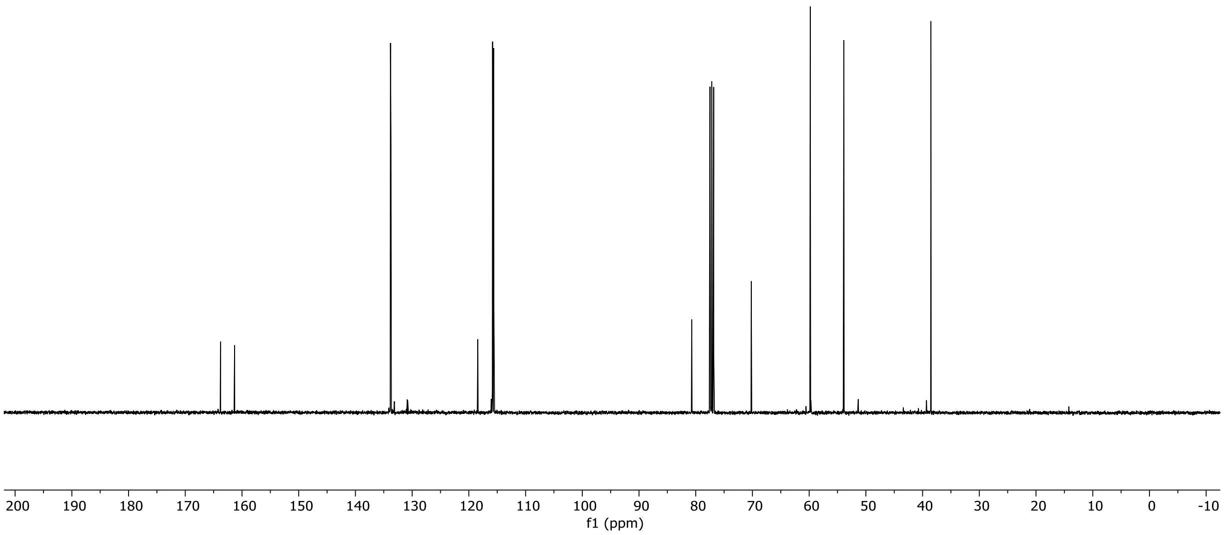

Supplementary Fig 79. ^{1}H (top) and ^{13}C (bottom) NMR spectra of compound **S2n**.

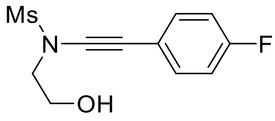

S2n

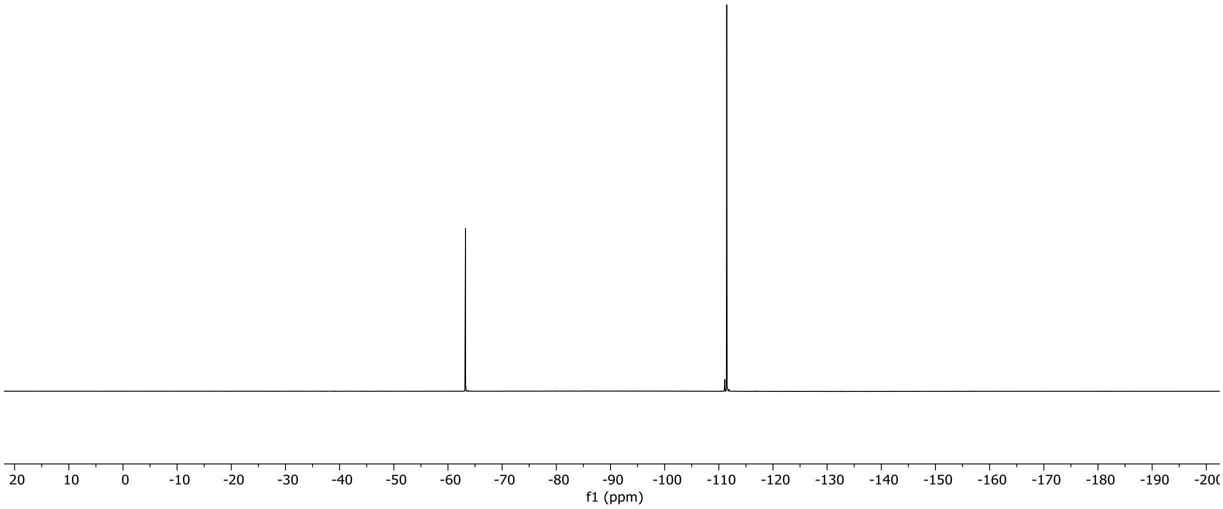

Supplementary Fig 80. ^{19}F NMR spectrum of compound **S2n**.

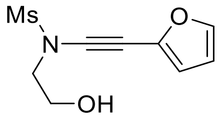

S2o

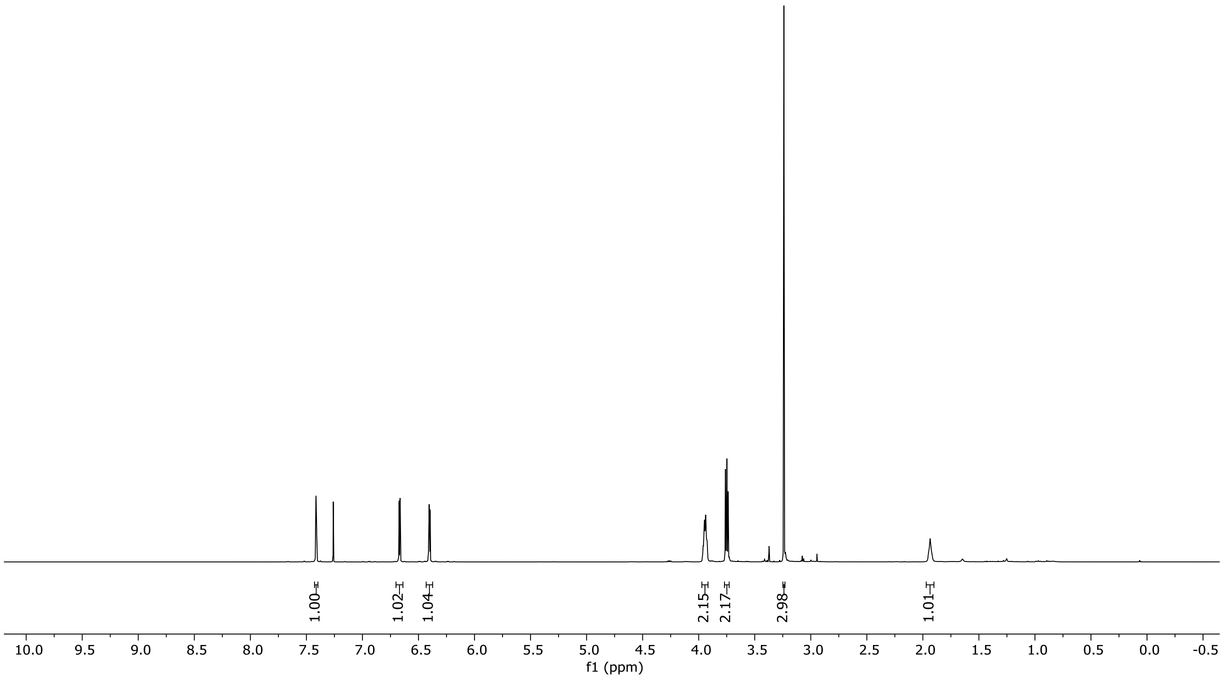

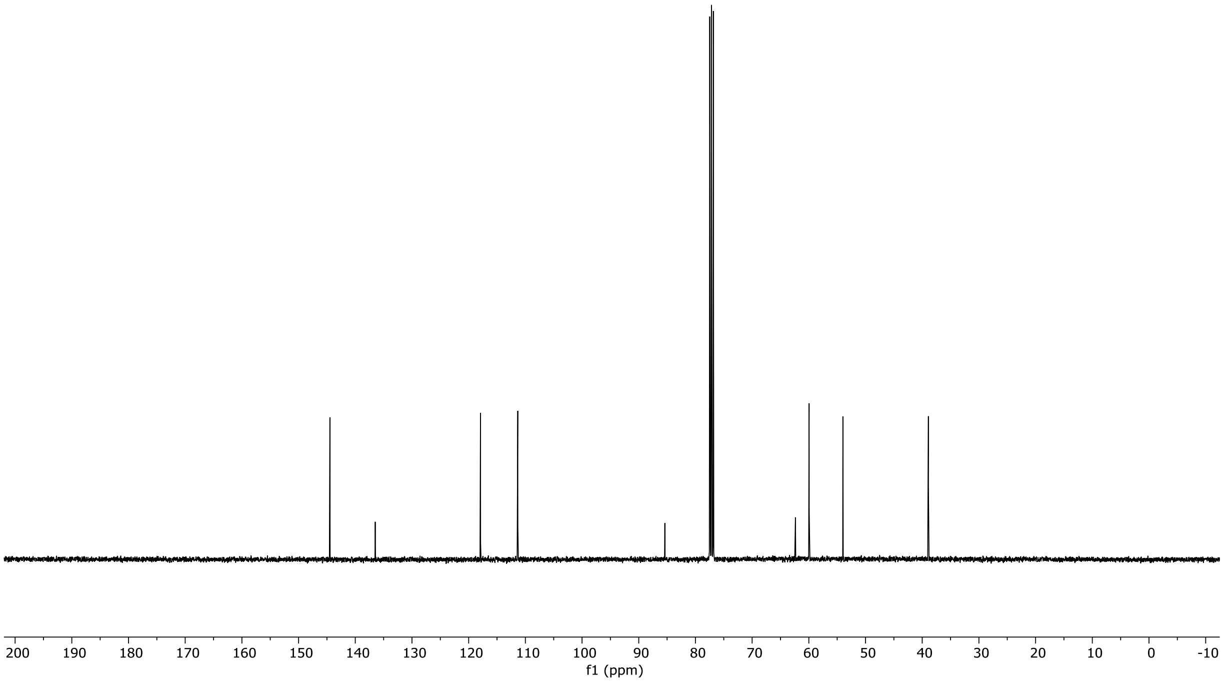

Supplementary Fig 81. ^{1}H (top) and ^{13}C (bottom) NMR spectra of compound **S2o**.

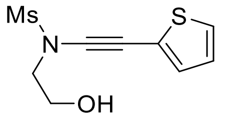

S2p

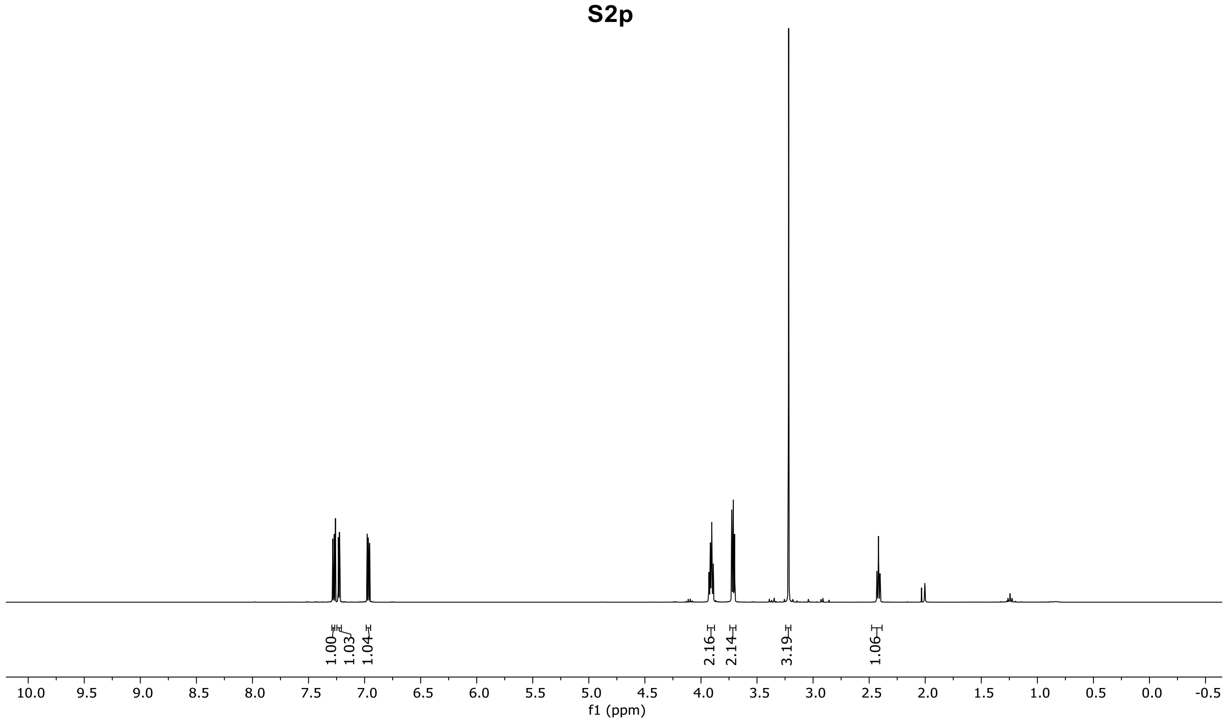

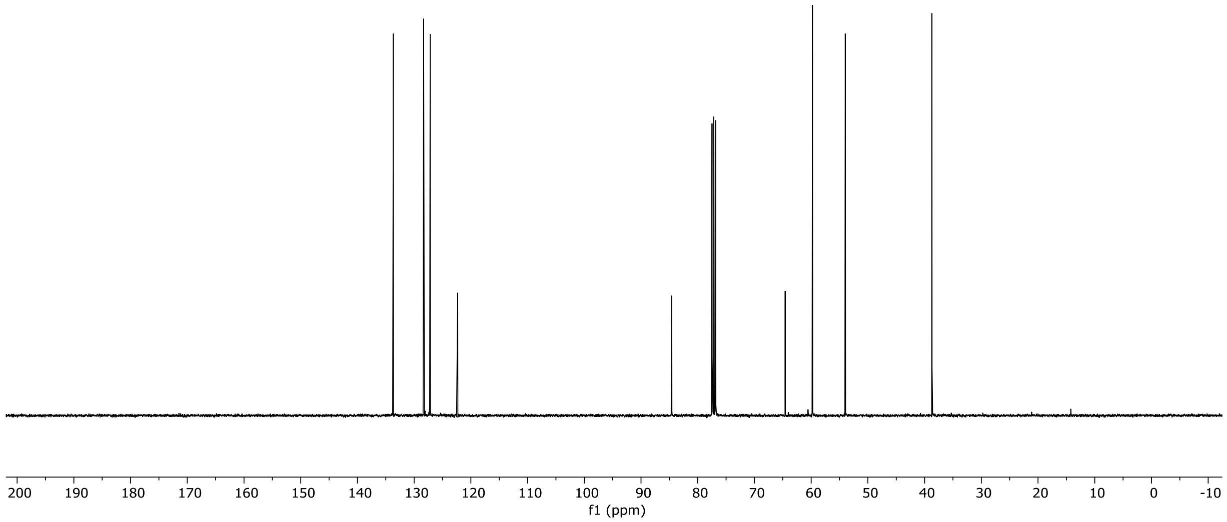

Supplementary Fig 82. ^{1}H (top) and ^{13}C (bottom) NMR spectra of compound **S2p**.

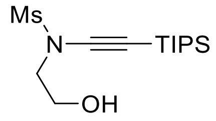

S2q

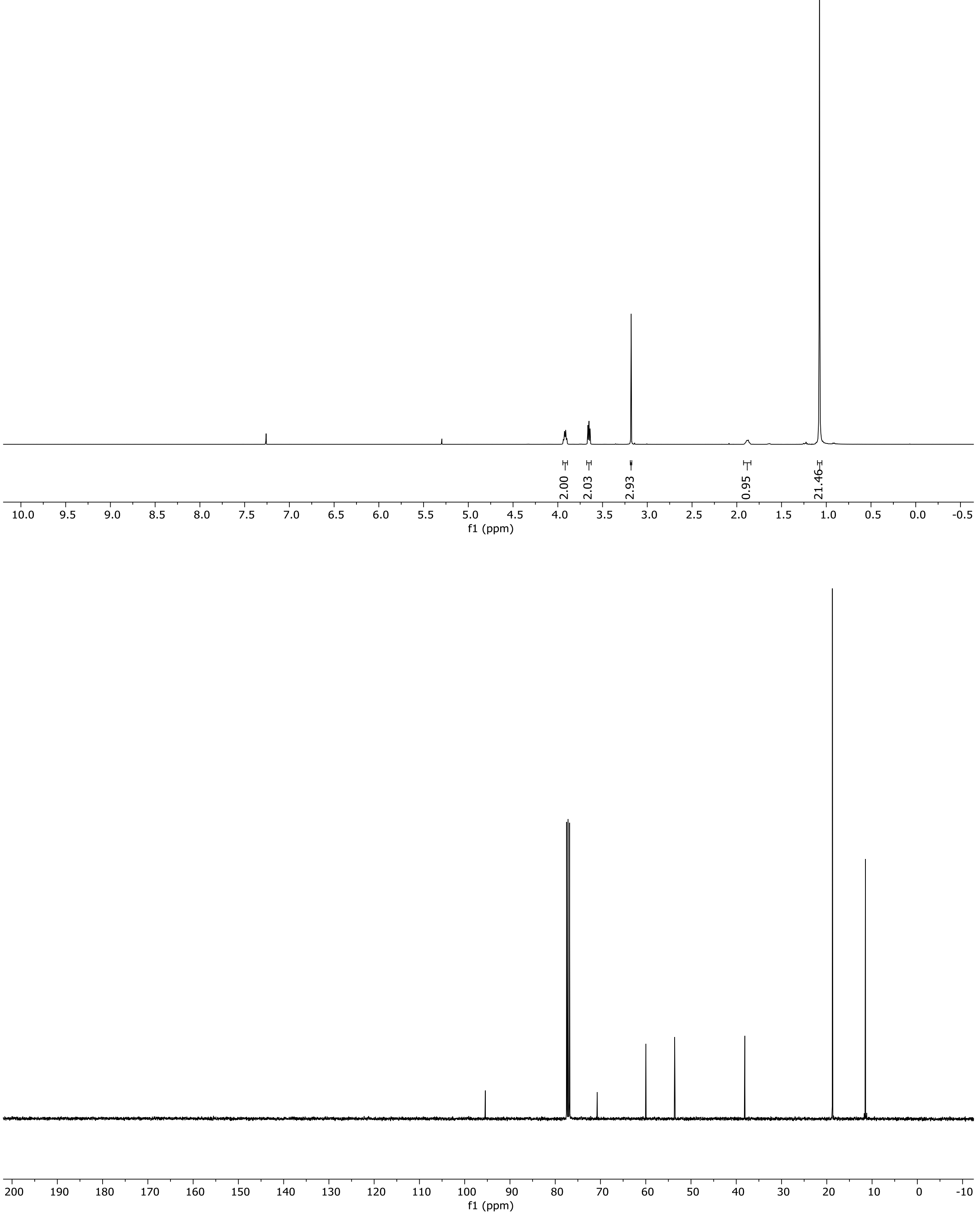

Supplementary Fig 83. ^{1}H (top) and ^{13}C (bottom) NMR spectra of compound **S2q**.

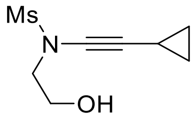

S2s

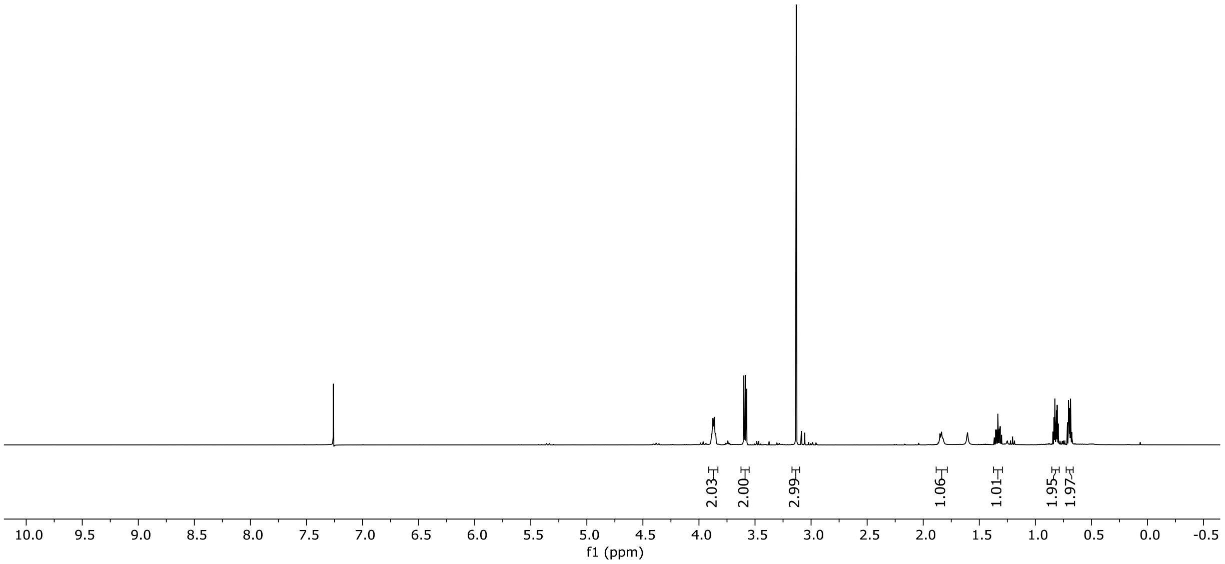

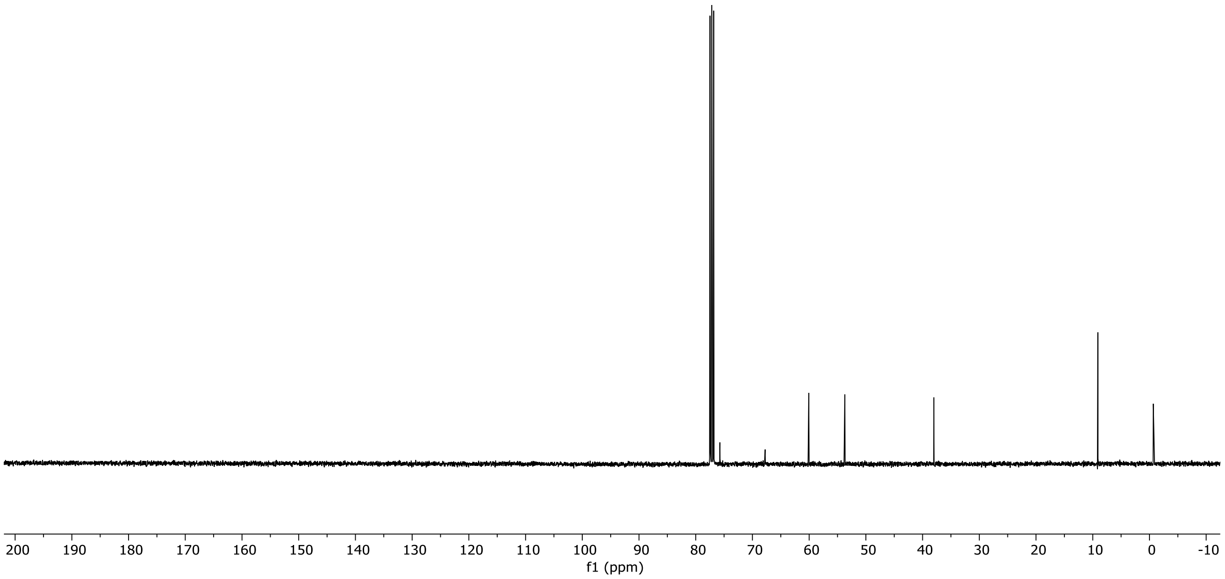

Supplementary Fig 84. ^{1}H (top) and ^{13}C (bottom) NMR spectra of compound **S2s**.

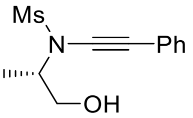

S2t

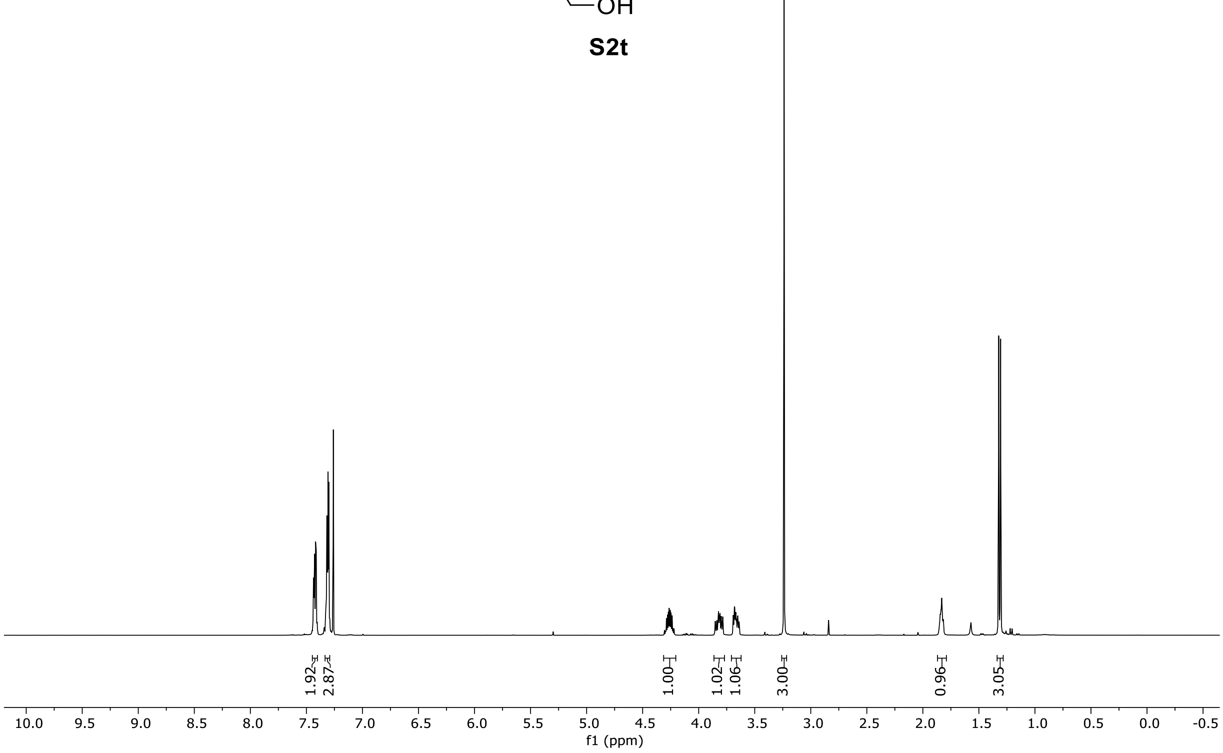

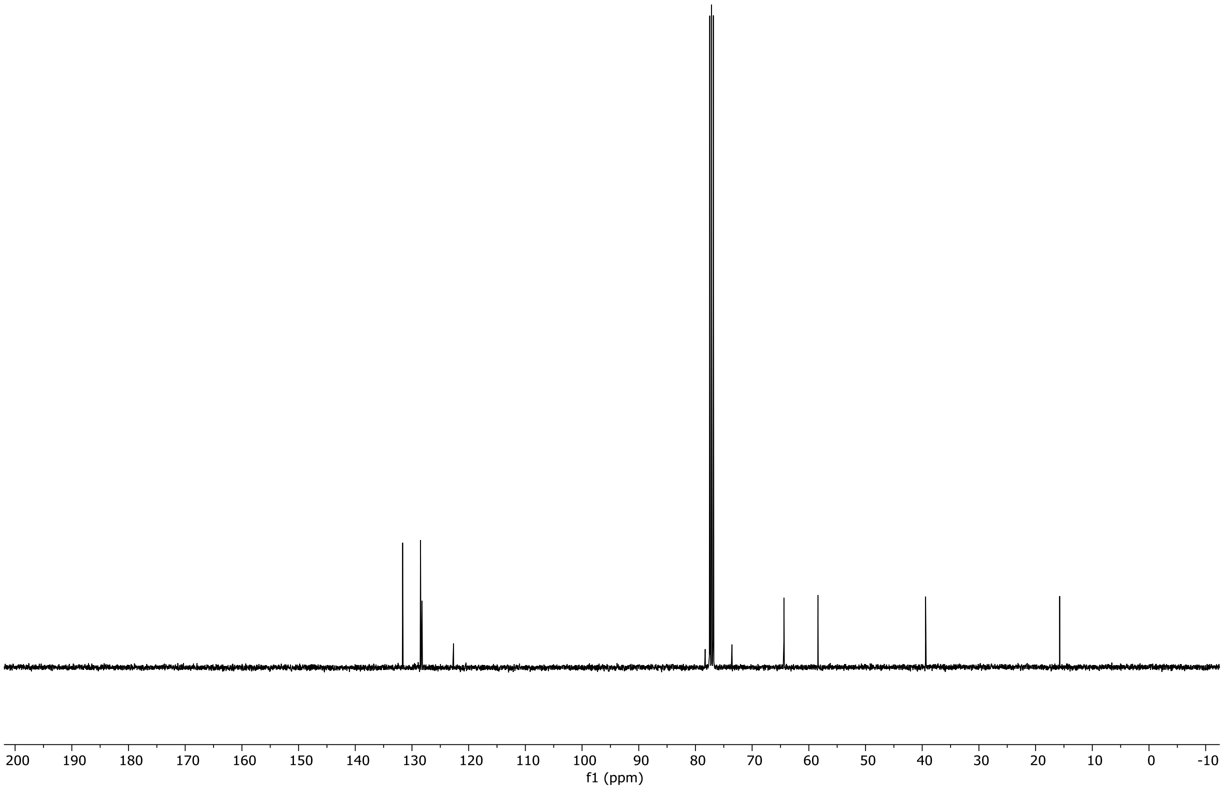

Supplementary Fig 85. ^{1}H (top) and ^{13}C (bottom) NMR spectra of compound **S2t**.

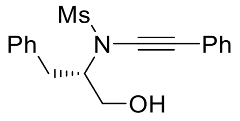

S2u

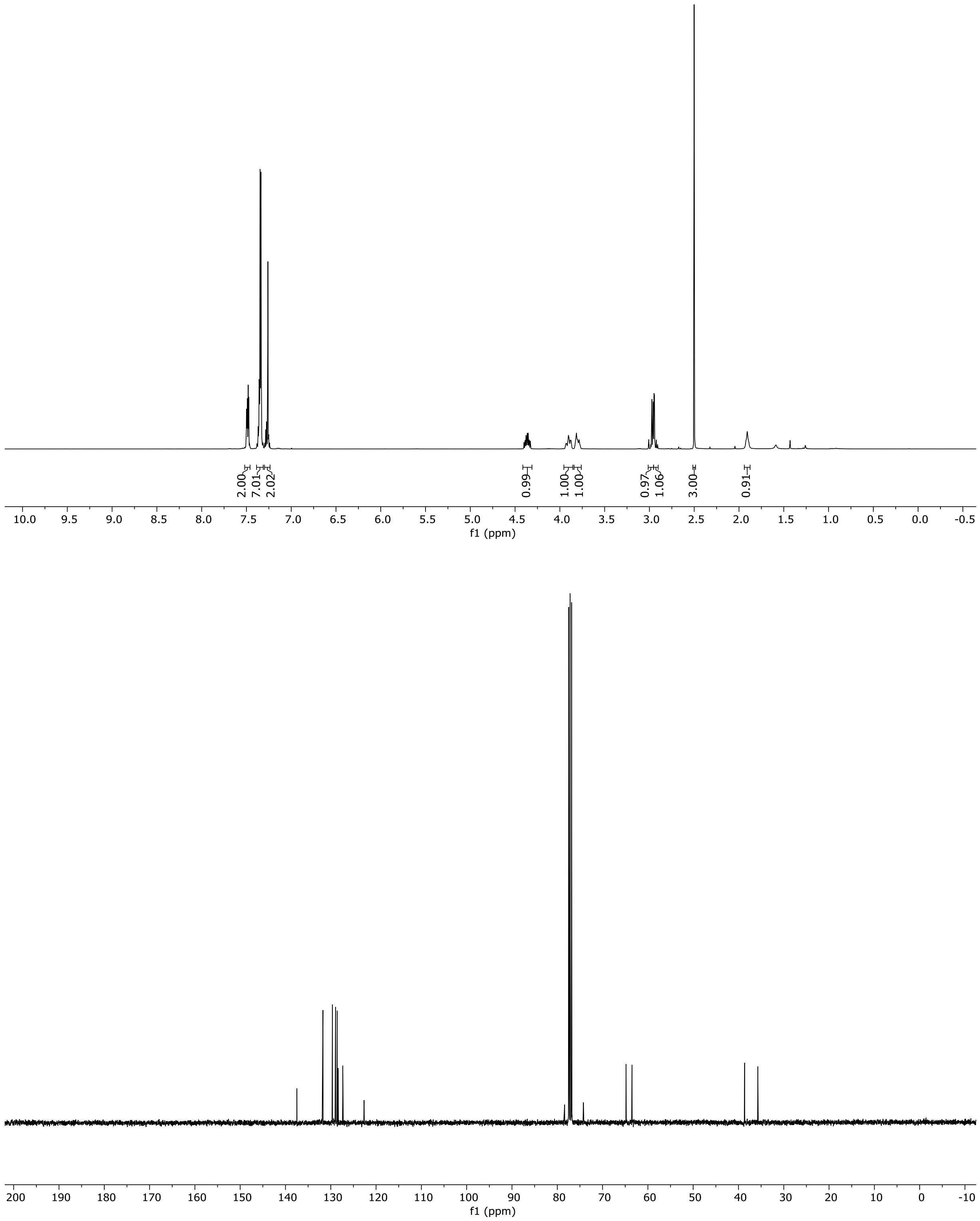

Supplementary Fig 86. ^{1}H (top) and ^{13}C (bottom) NMR spectra of compound **S2u**.

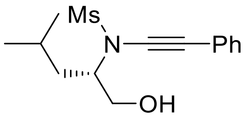

S2v

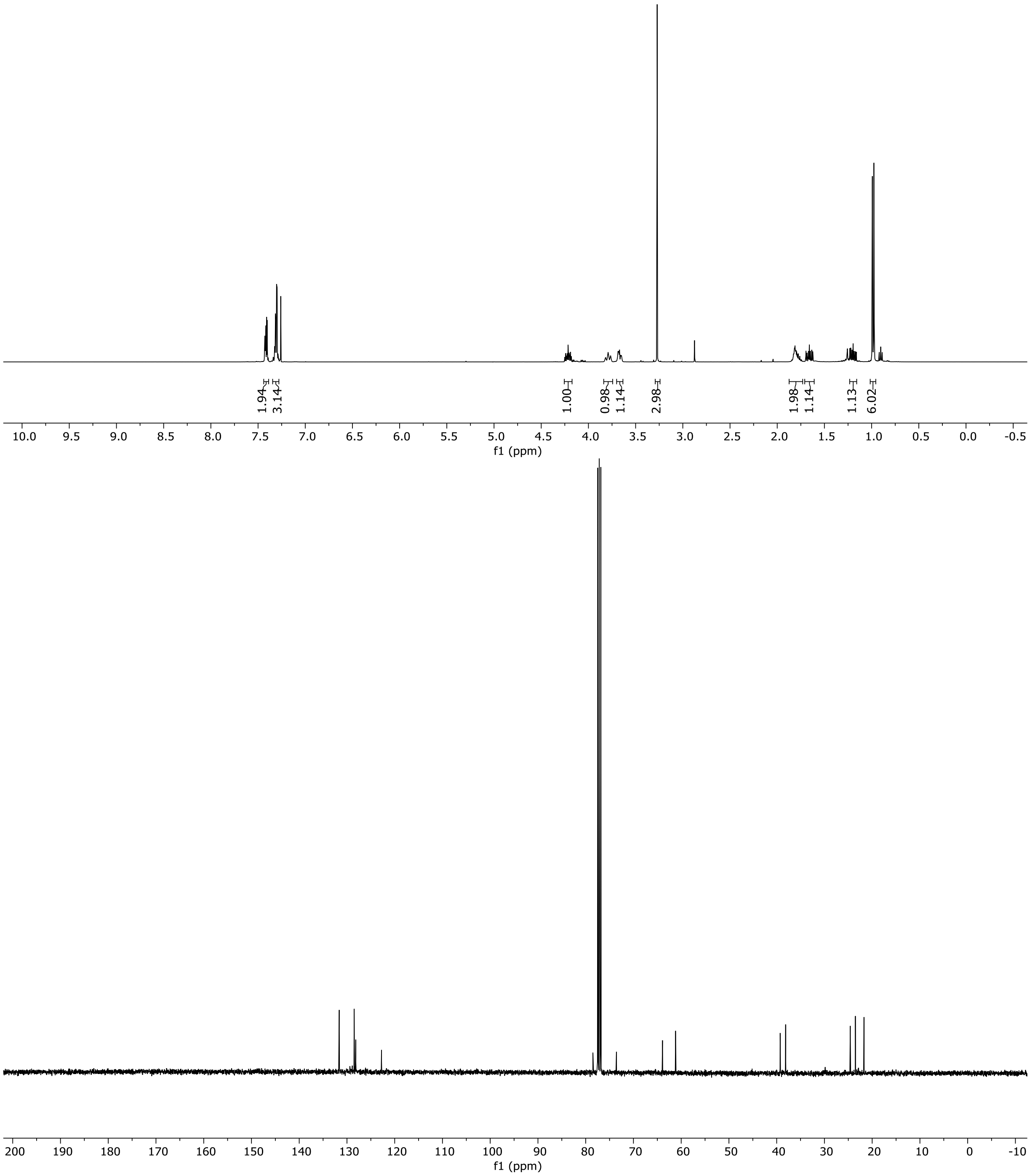

Supplementary Fig 87. ^{1}H (top) and ^{13}C (bottom) NMR spectra of compound **S2v**.

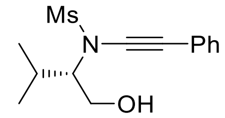

S2w

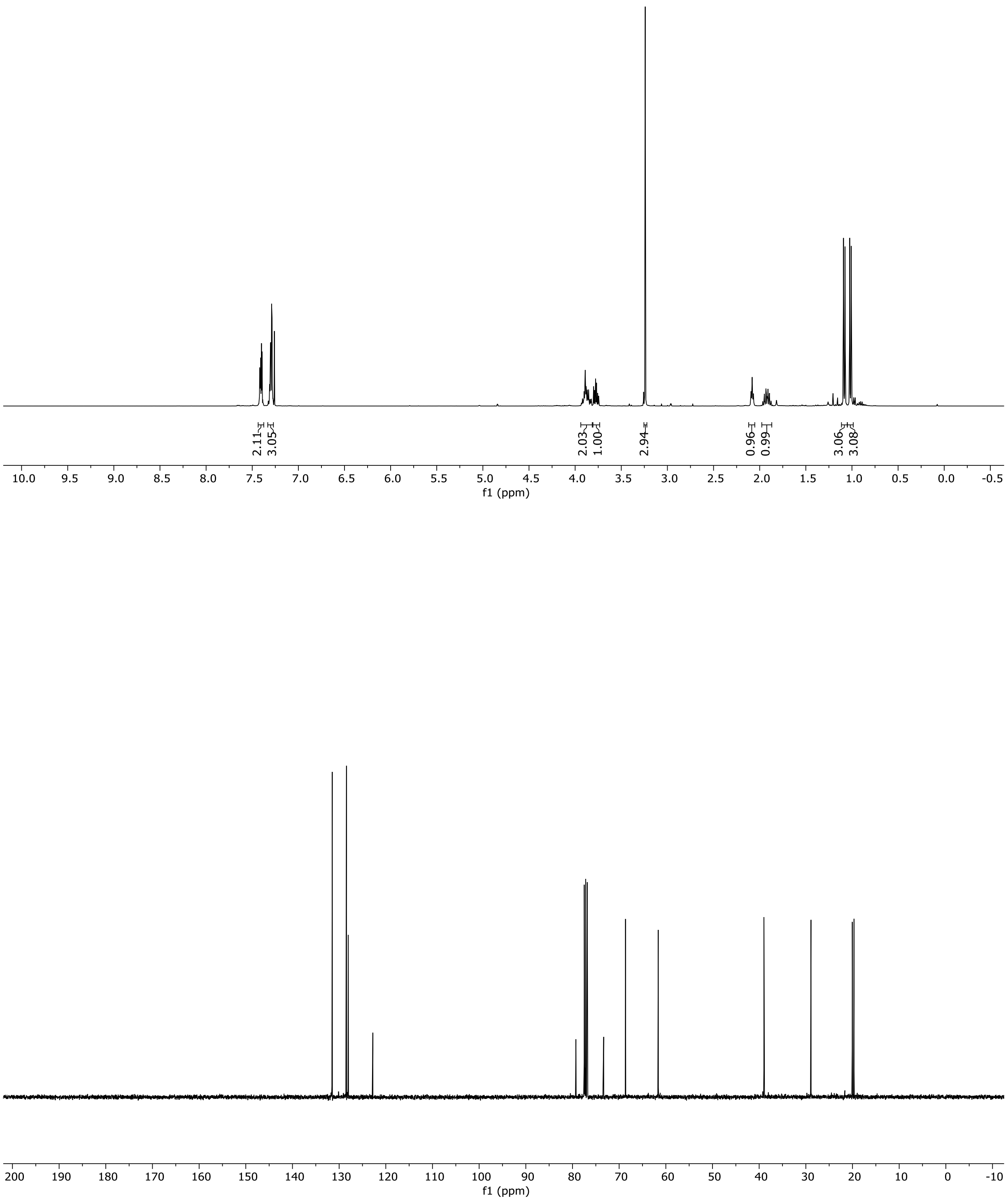

Supplementary Fig 88. ^{1}H (top) and ^{13}C (bottom) NMR spectra of compound **S2w**.

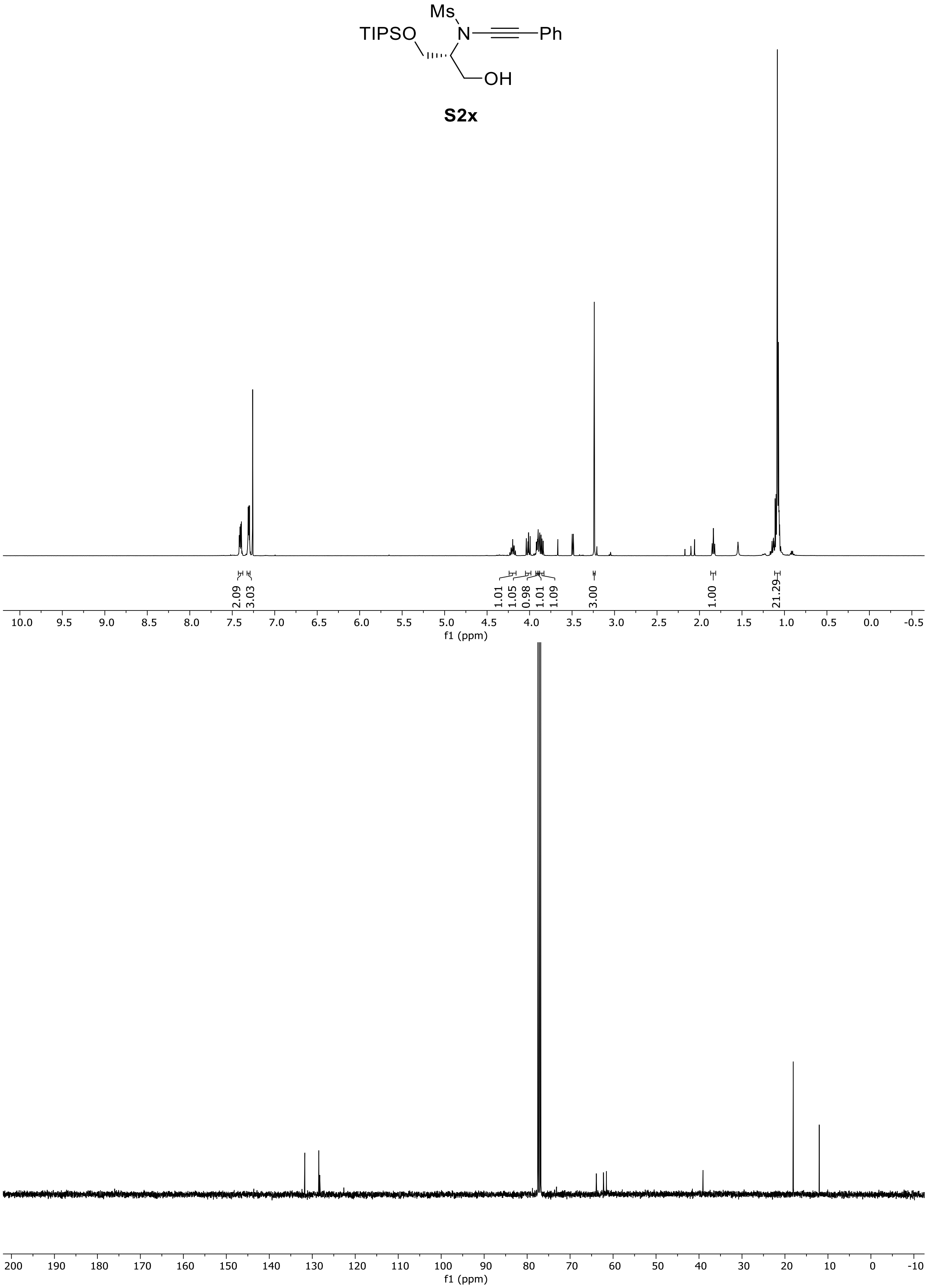

Supplementary Fig 89. ^{1}H (top) and ^{13}C (bottom) NMR spectra of compound **S2x**.

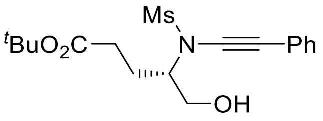

S2y

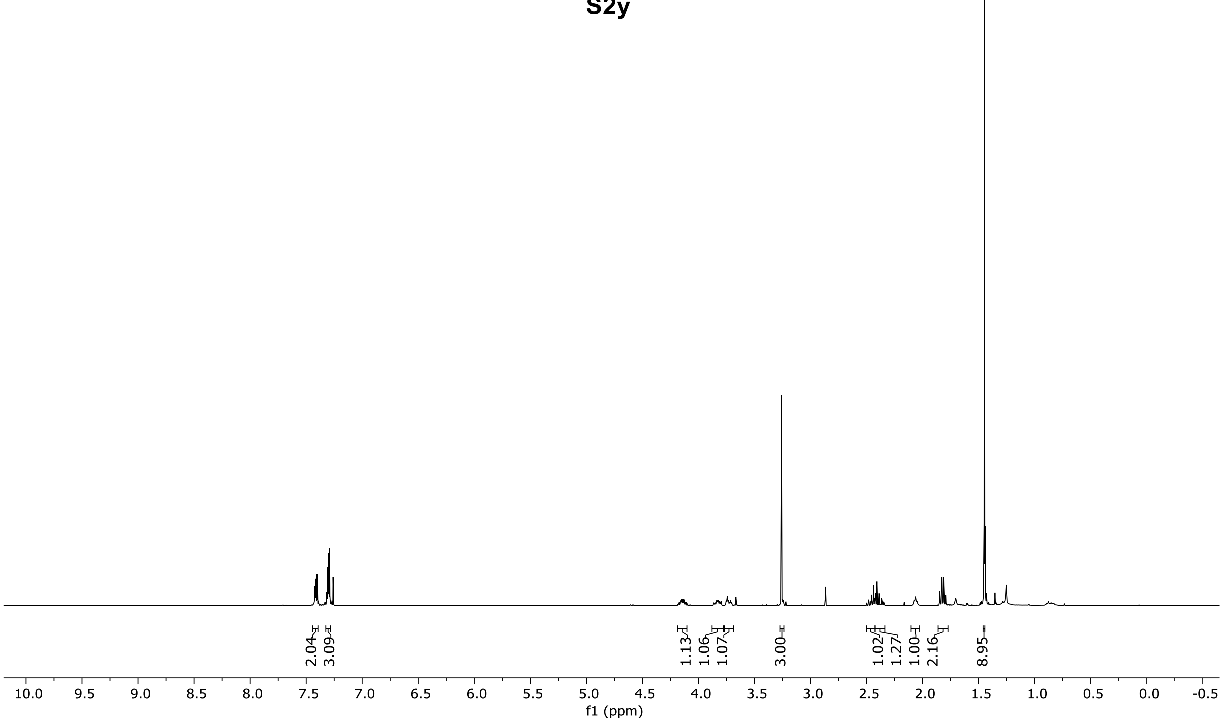

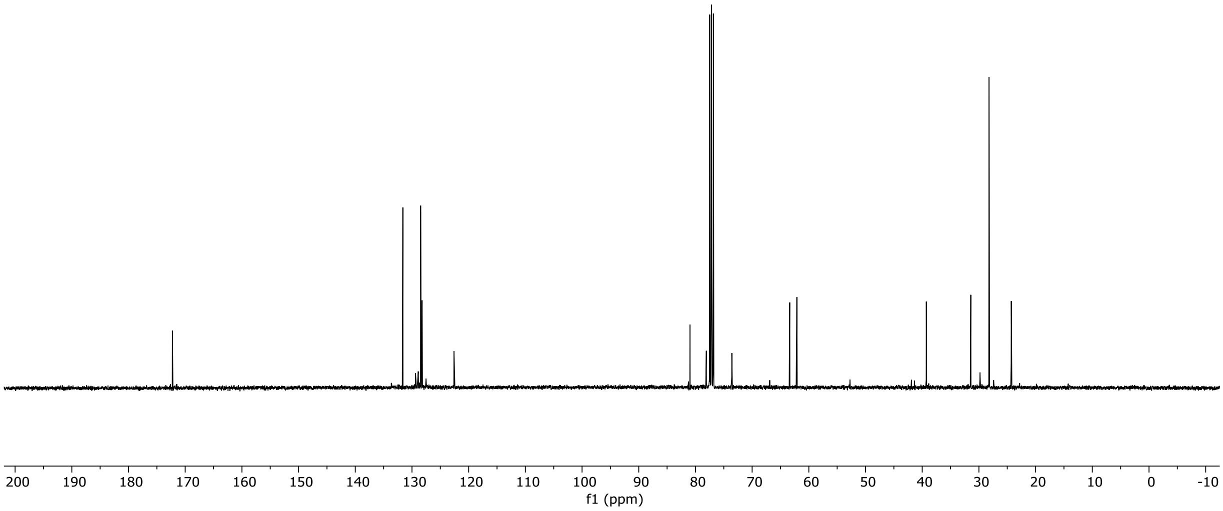

Supplementary Fig 90. ^{1}H (top) and ^{13}C (bottom) NMR spectra of compound **S2y**.

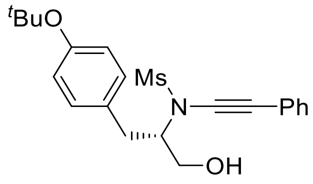

S2z

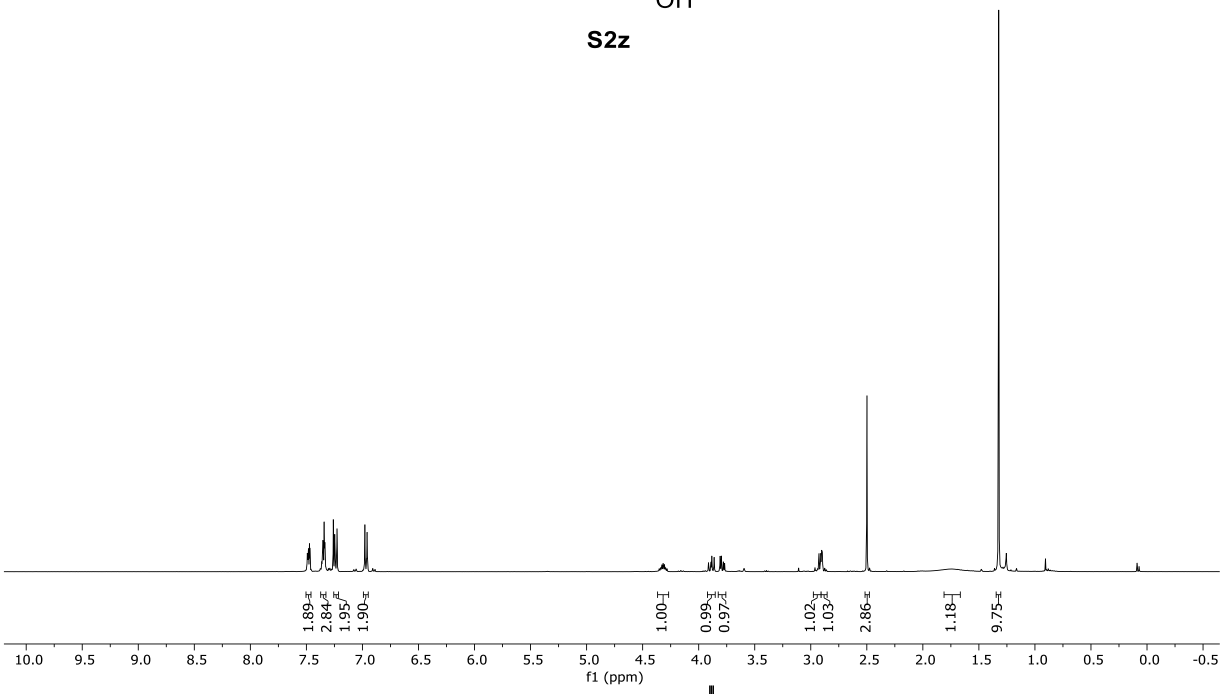

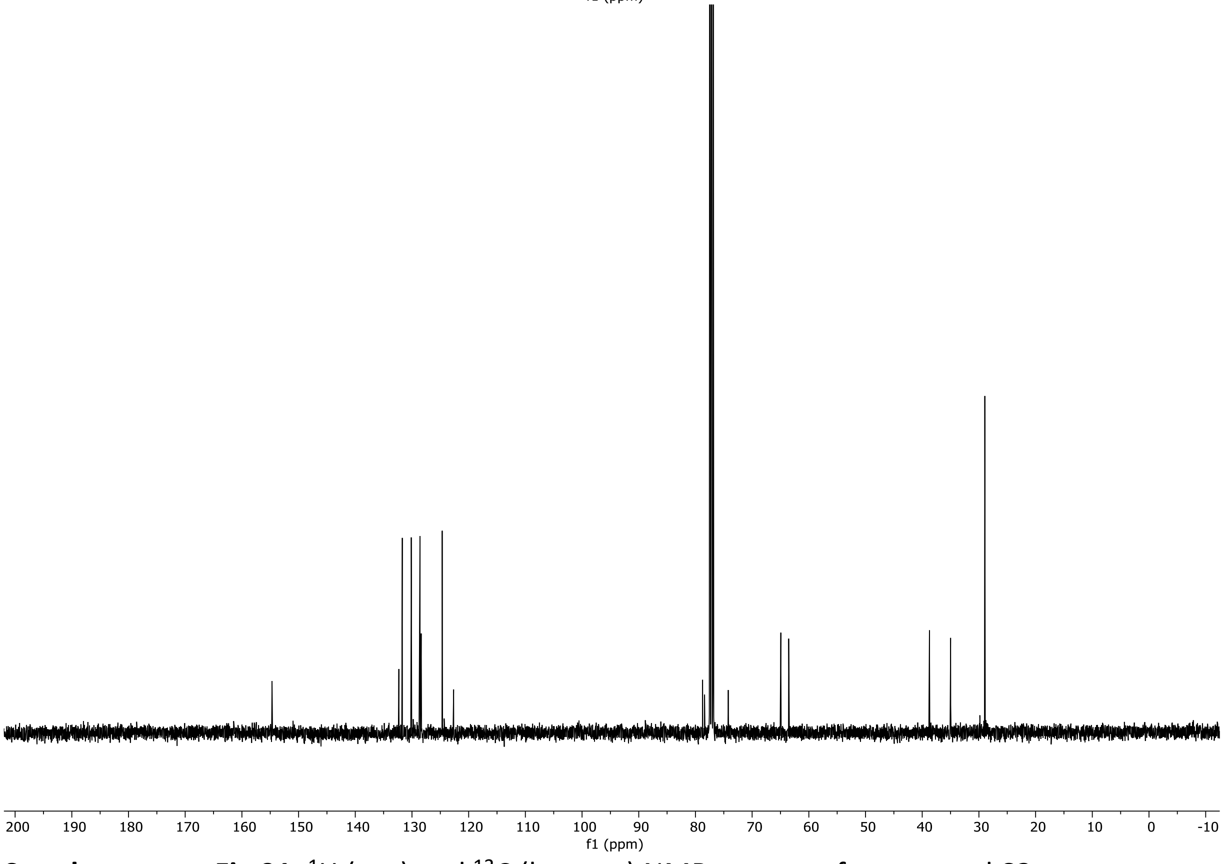

Supplementary Fig 91. ^{1}H (top) and ^{13}C (bottom) NMR spectra of compound **S2z**.

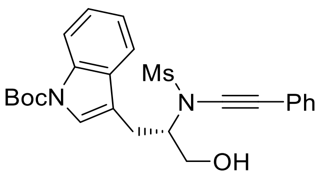

S2aa

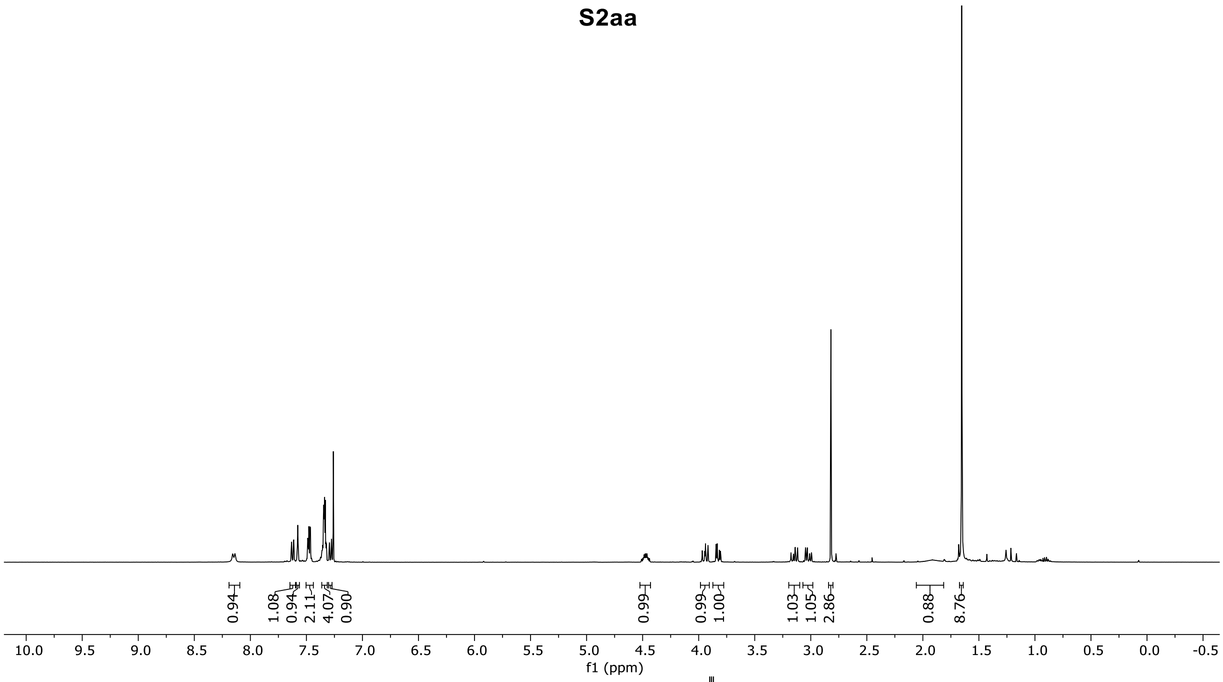

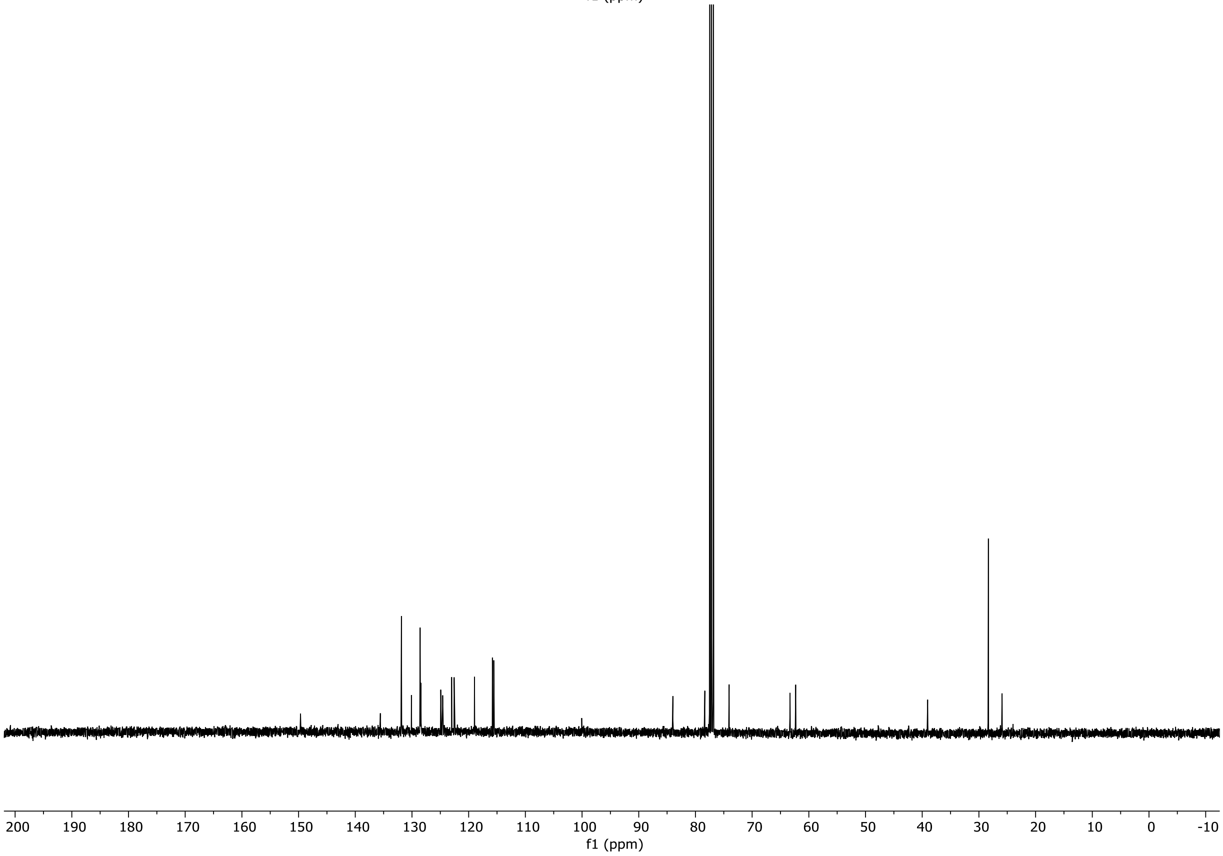

Supplementary Fig 92. ^{1}H (top) and ^{13}C (bottom) NMR spectra of compound **S2aa**.

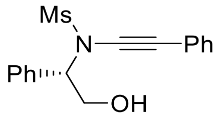

S2ab

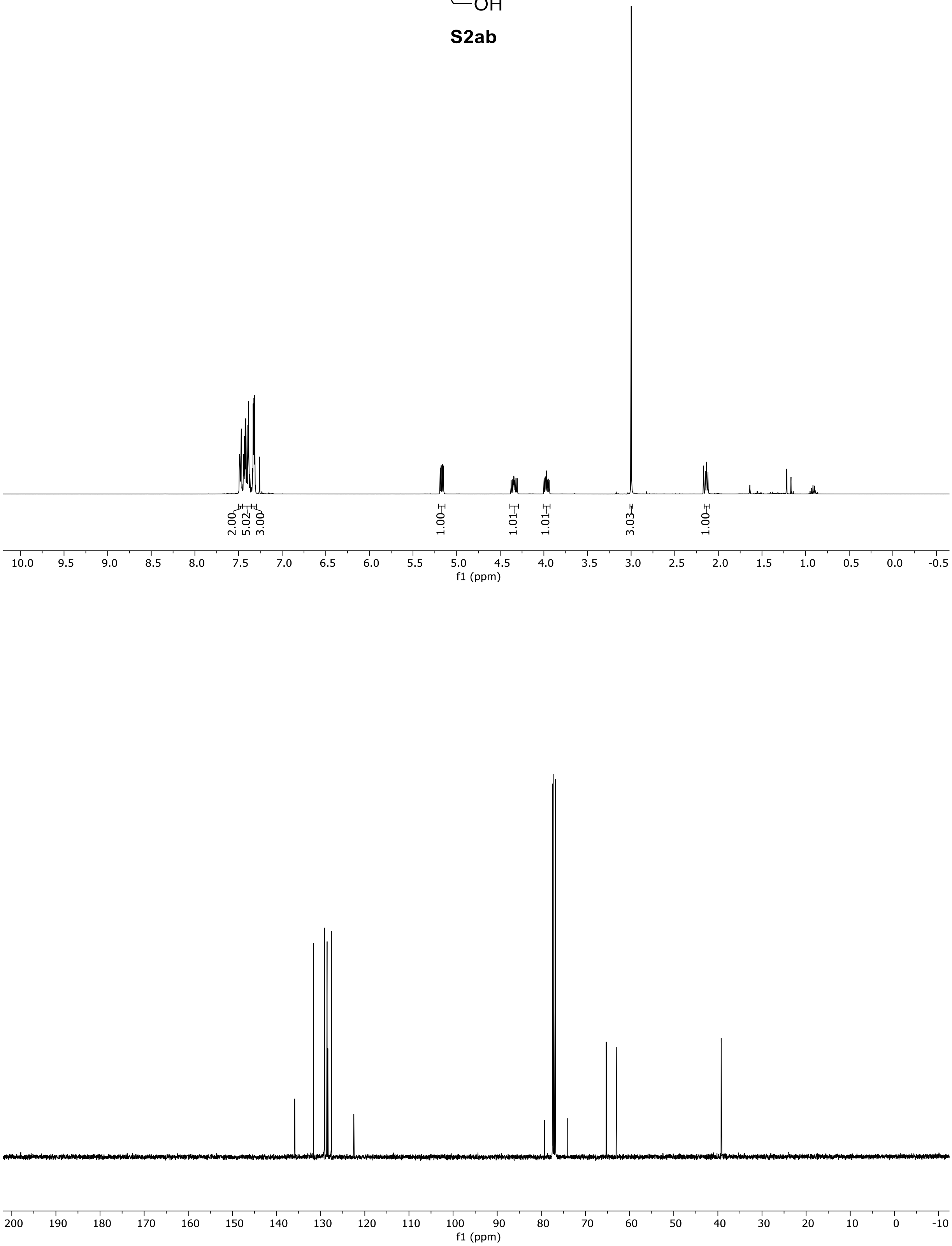

Supplementary Fig 93. ^{1}H (top) and ^{13}C (bottom) NMR spectra of compound **S2ab**.

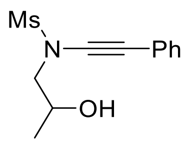

S2ac

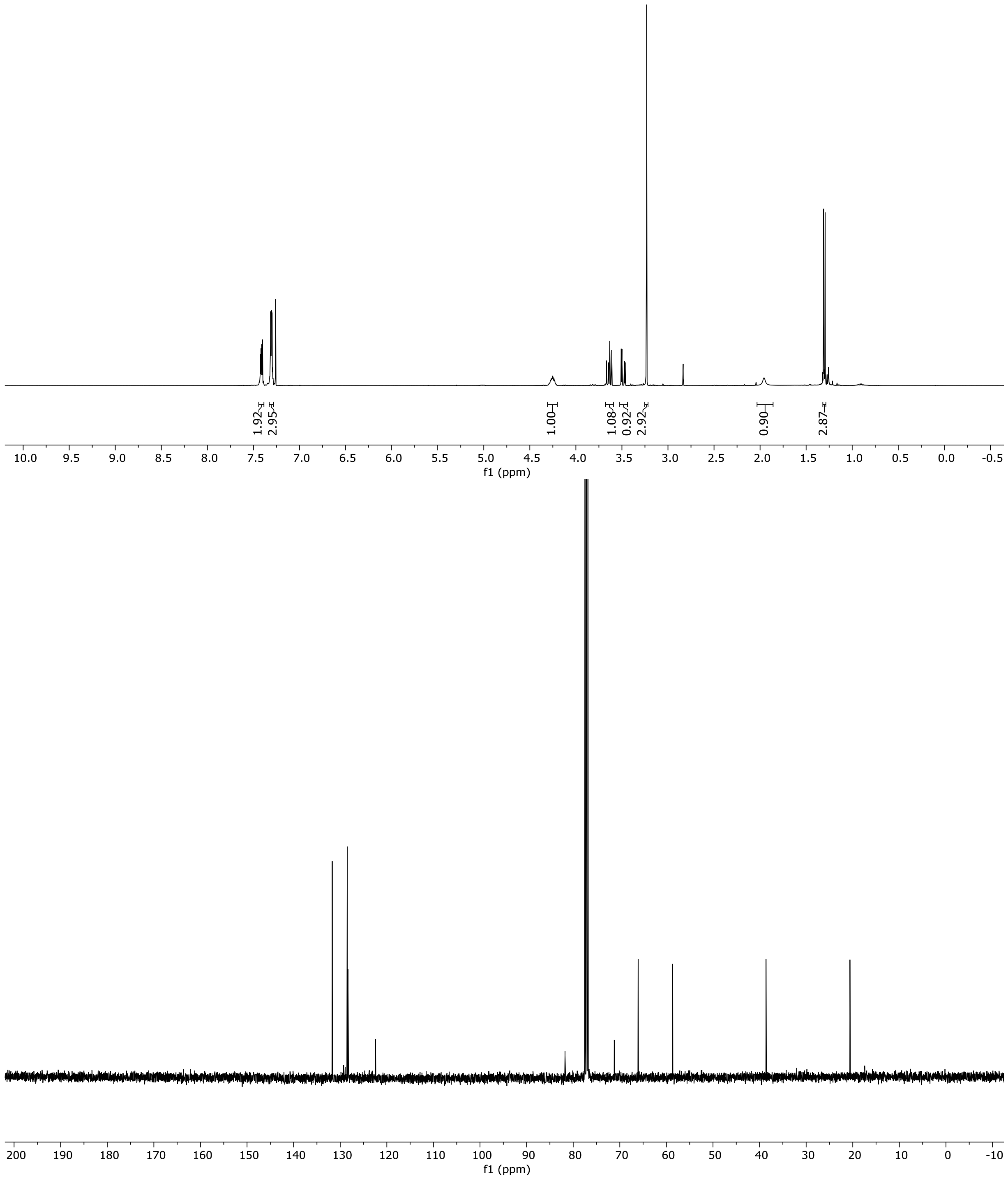

Supplementary Fig 94. ^{1}H (top) and ^{13}C (bottom) NMR spectra of compound **S2ac**.

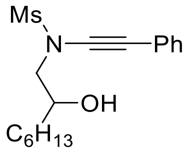

S2ad

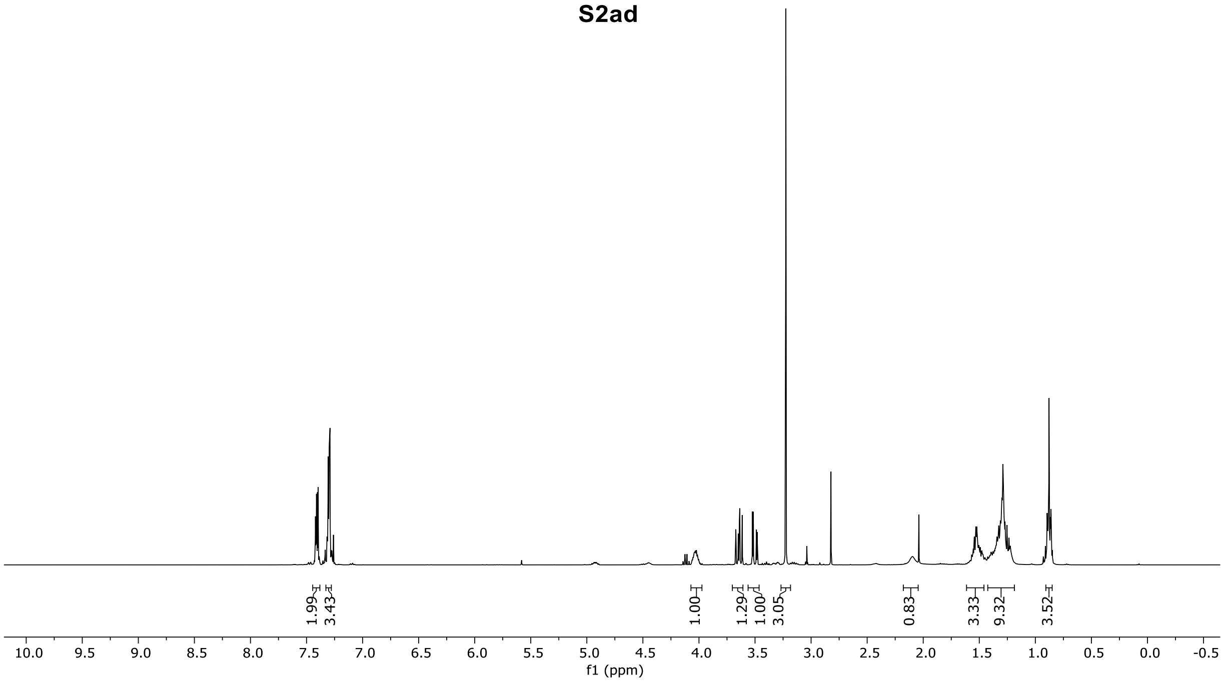

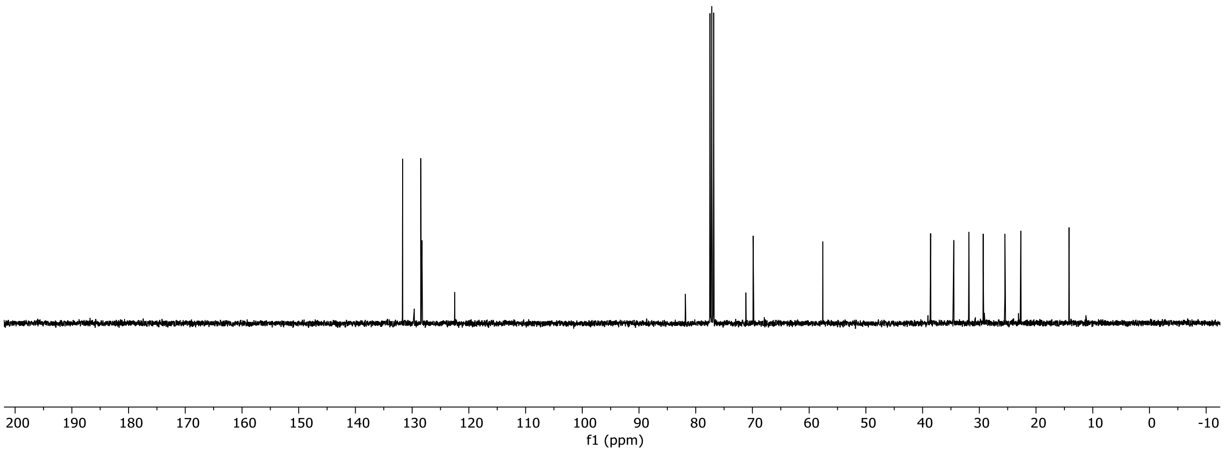

Supplementary Fig 95. ^{1}H (top) and ^{13}C (bottom) NMR spectra of compound **S2ad**.

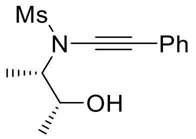

S2af

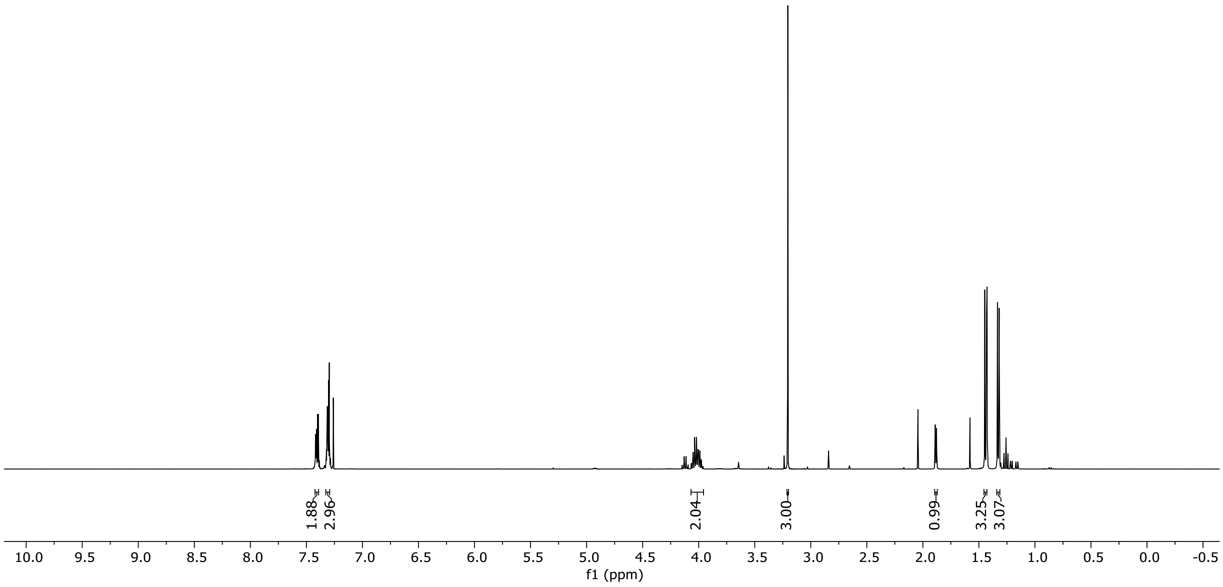

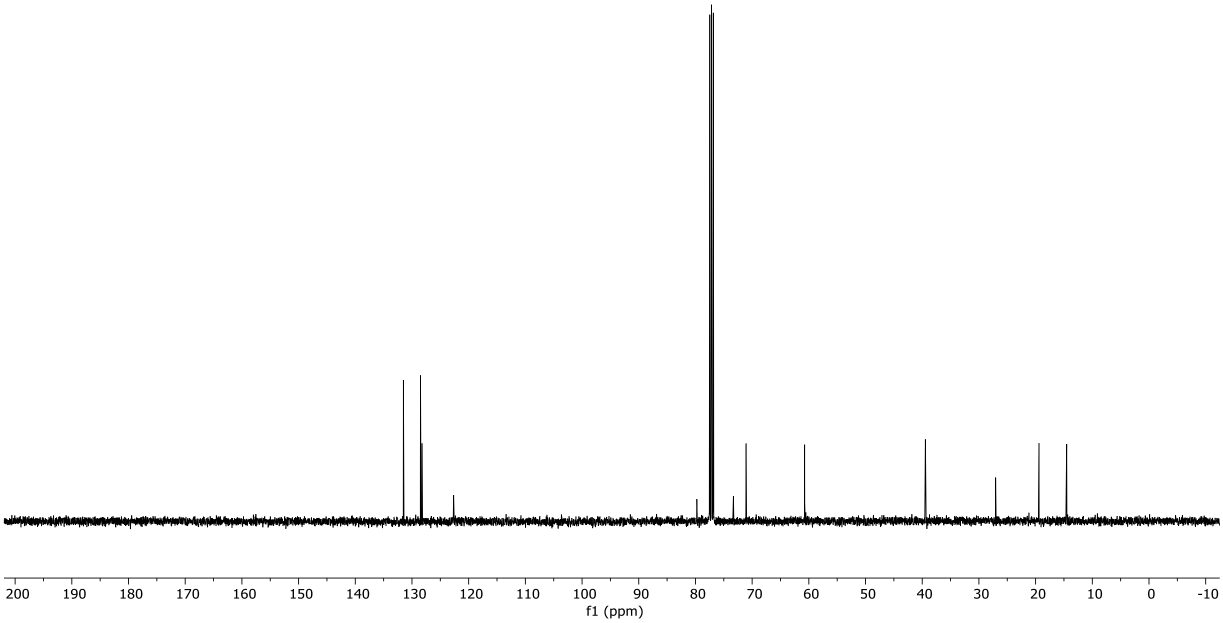

Supplementary Fig 96. ^{1}H (top) and ^{13}C (bottom) NMR spectra of compound **S2af**.

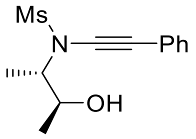

S2af'

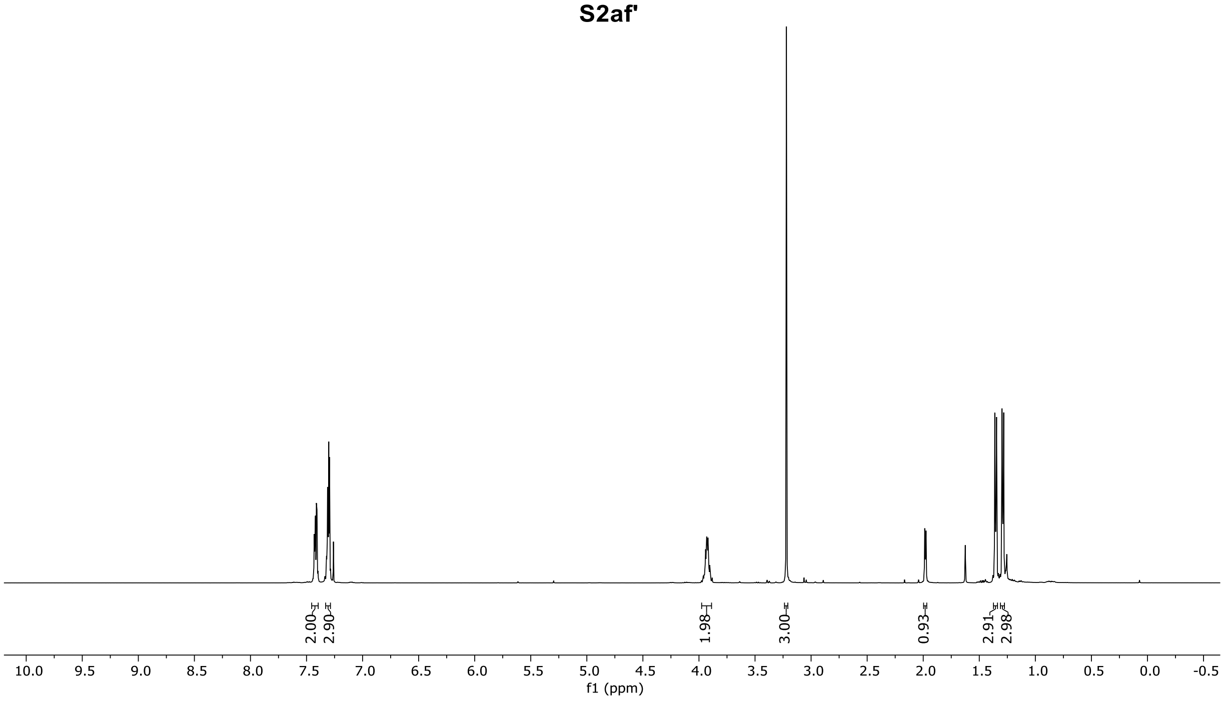

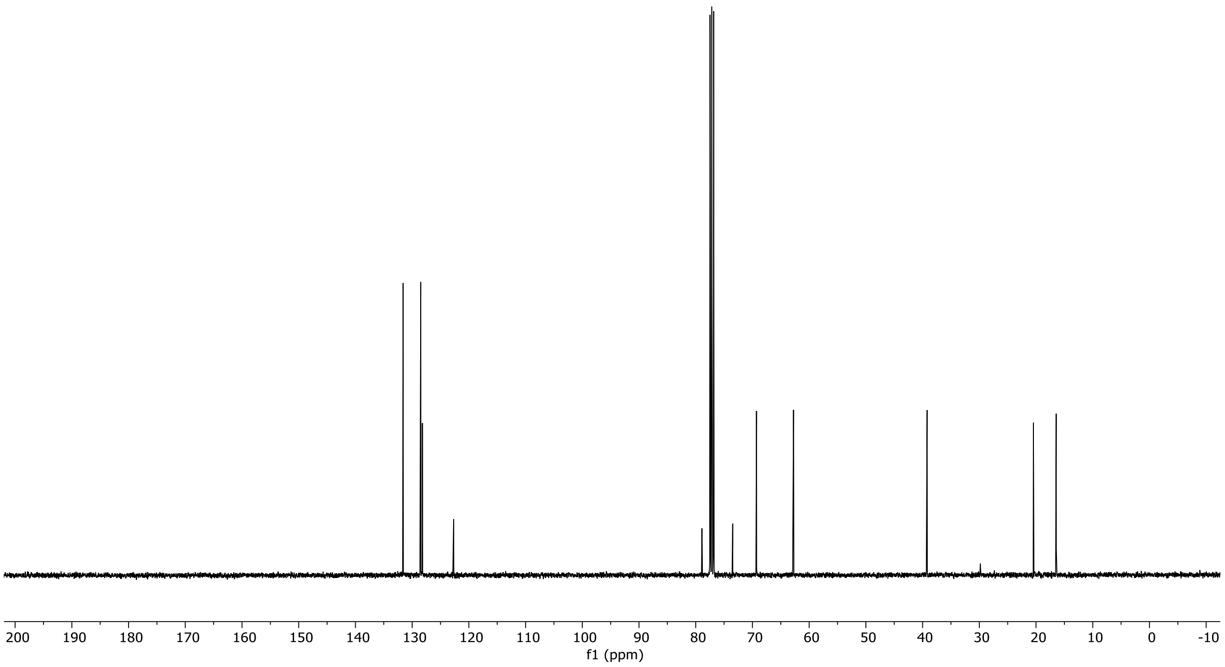

Supplementary Fig 97. ^{1}H (top) and ^{13}C (bottom) NMR spectra of compound **S2af'**.

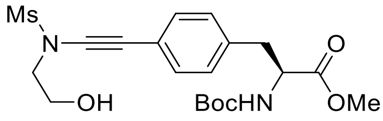

S2ag

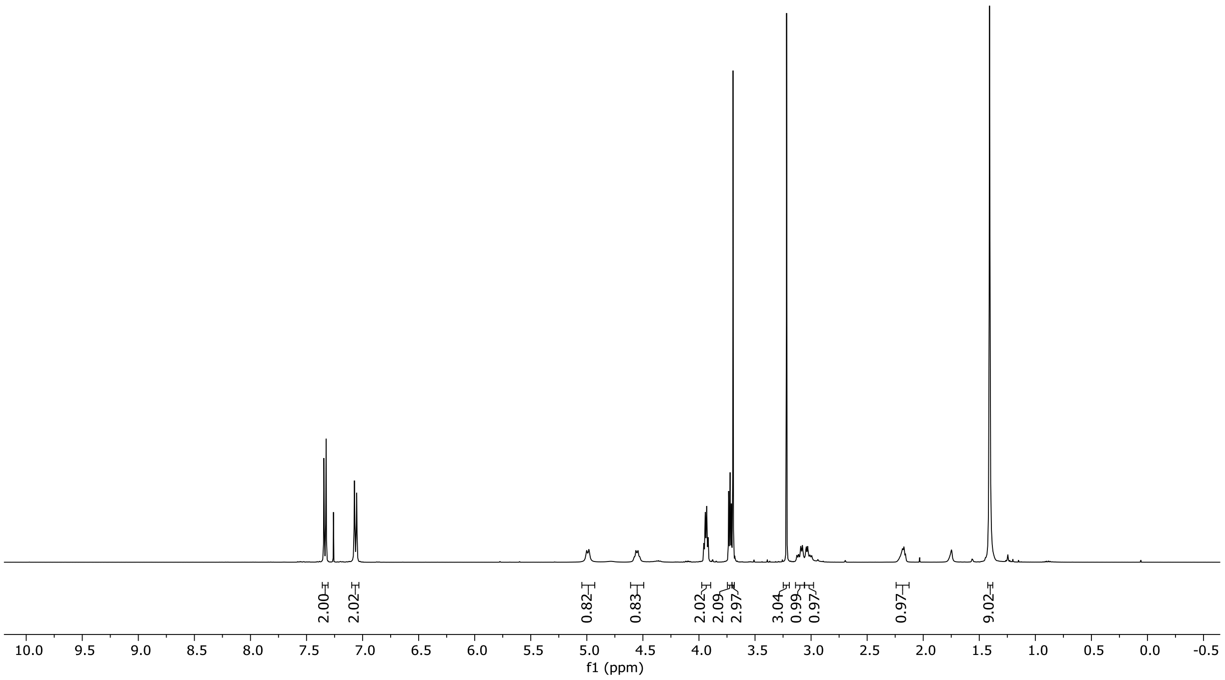

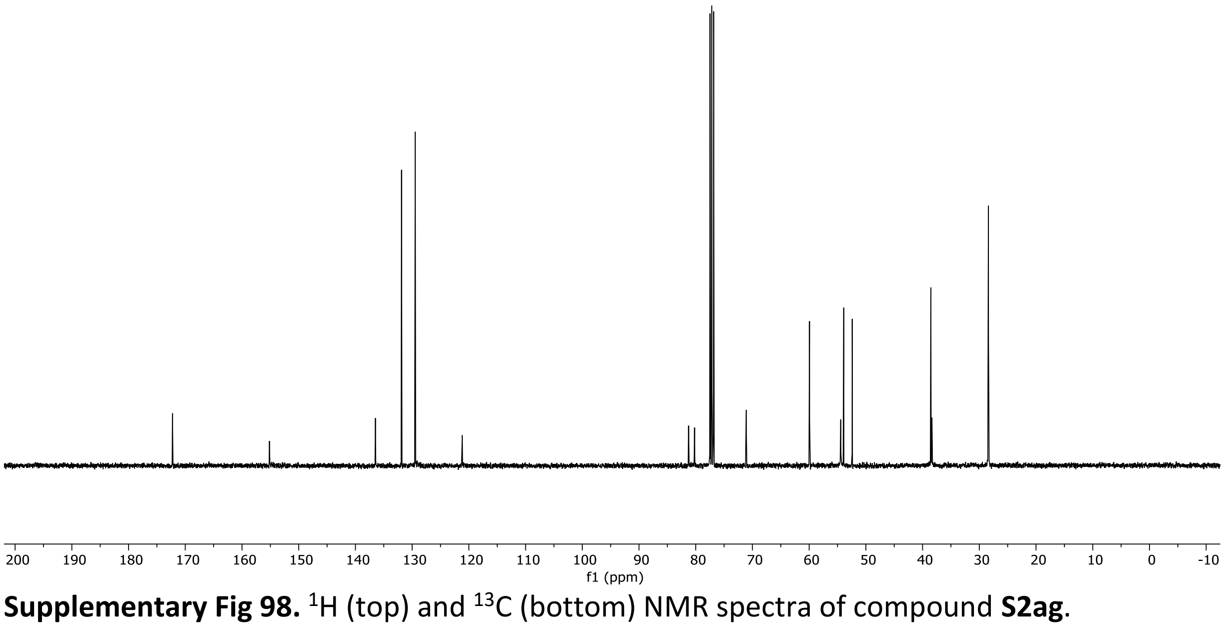

Supplementary Fig 98. ^{1}H (top) and ^{13}C (bottom) NMR spectra of compound **S2ag**.

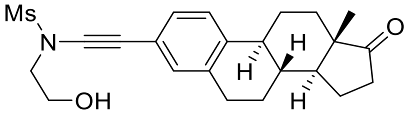

S2ah

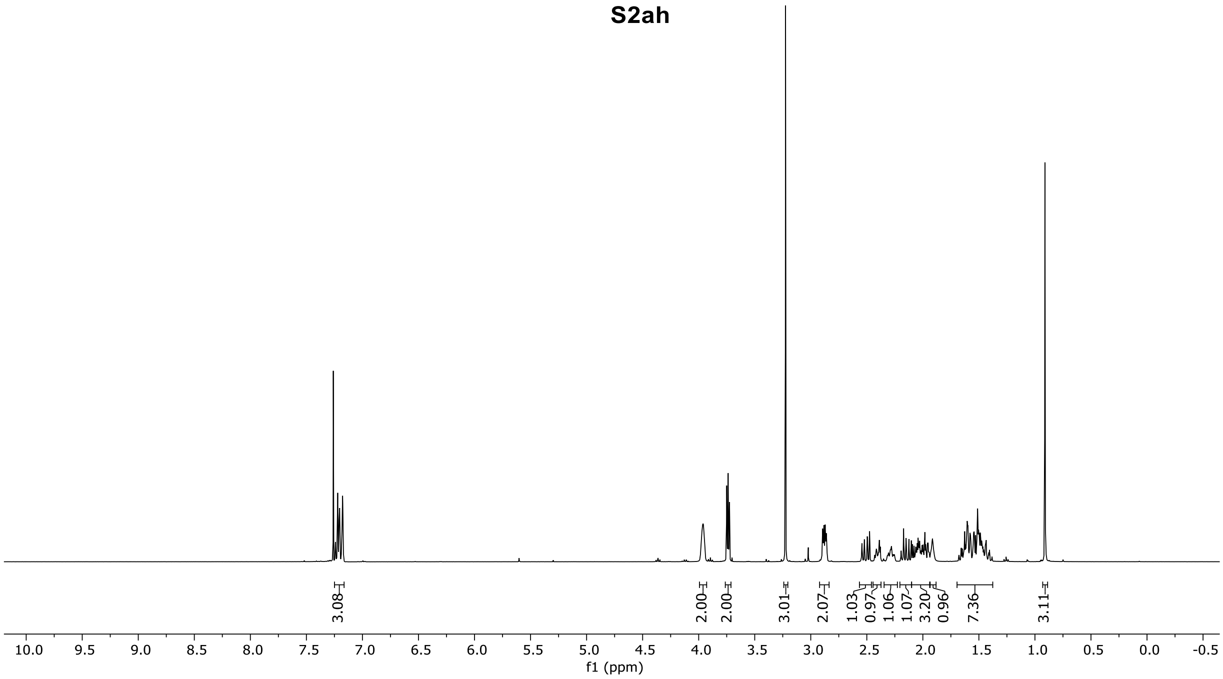

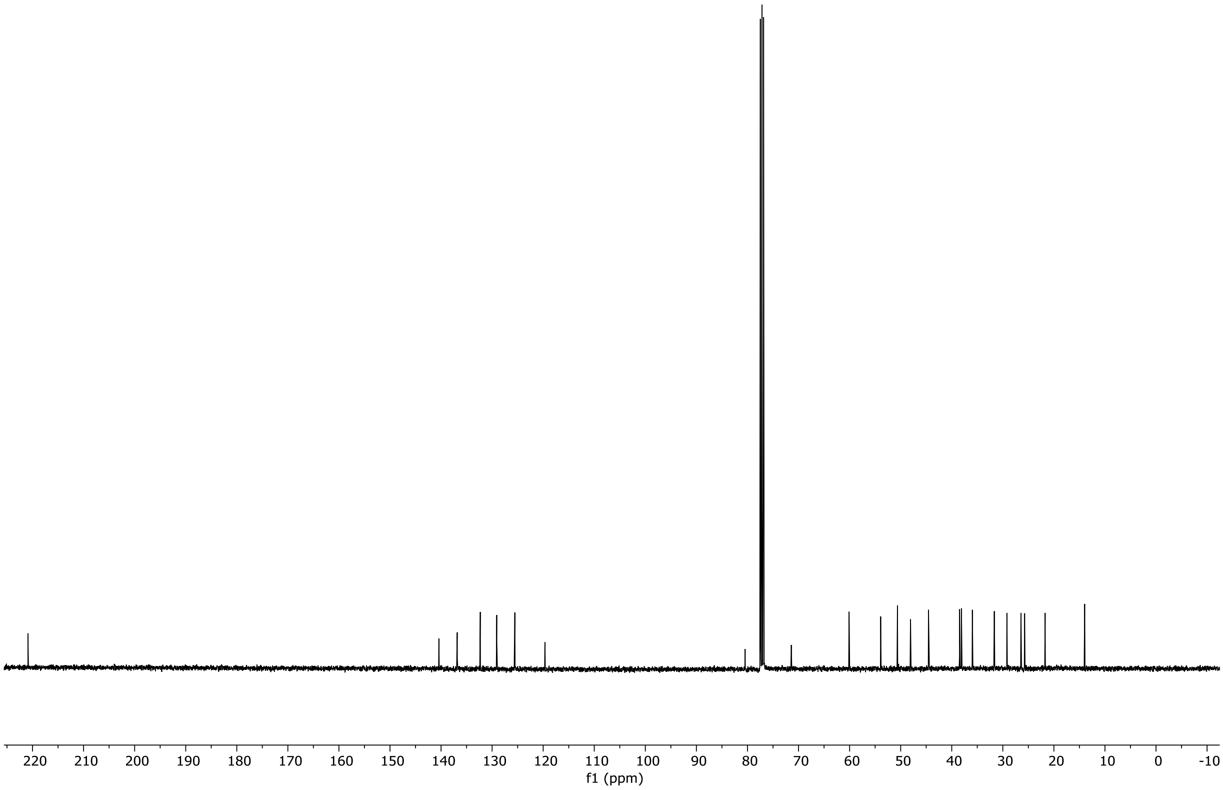

Supplementary Fig 99. ^{1}H (top) and ^{13}C (bottom) NMR spectra of compound **S2ah**.

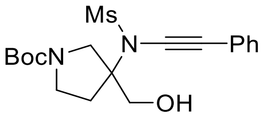

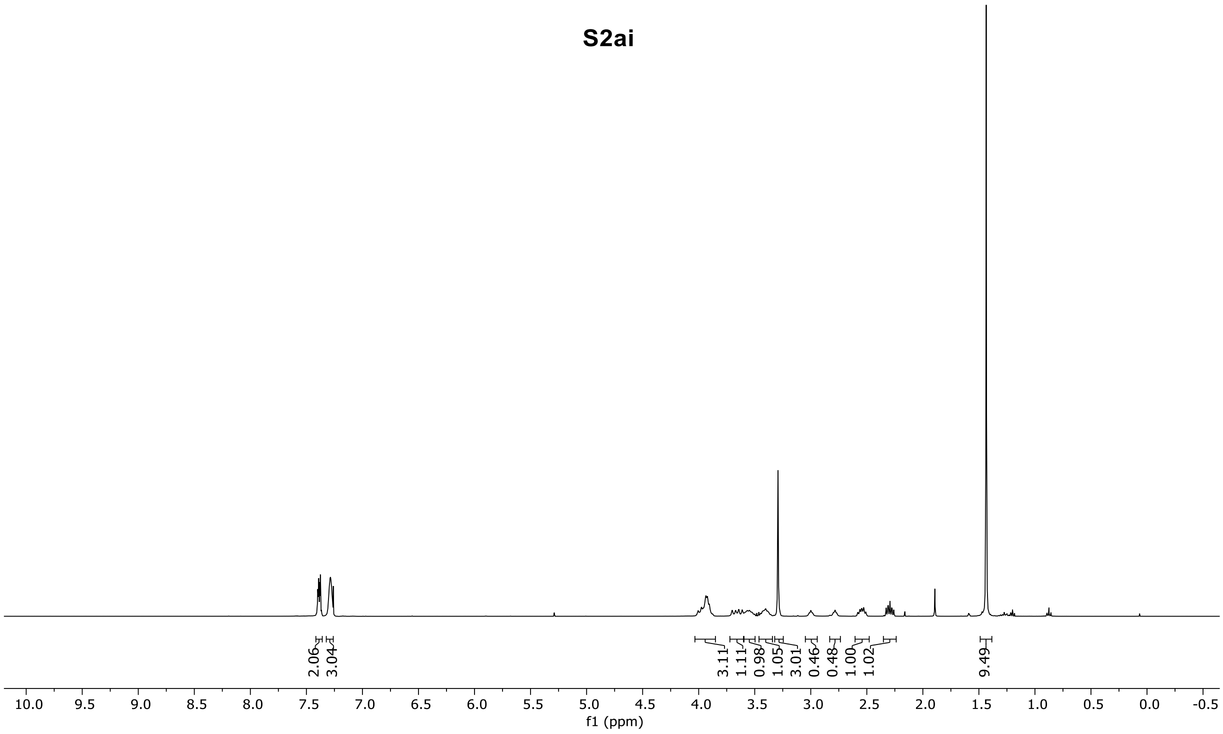

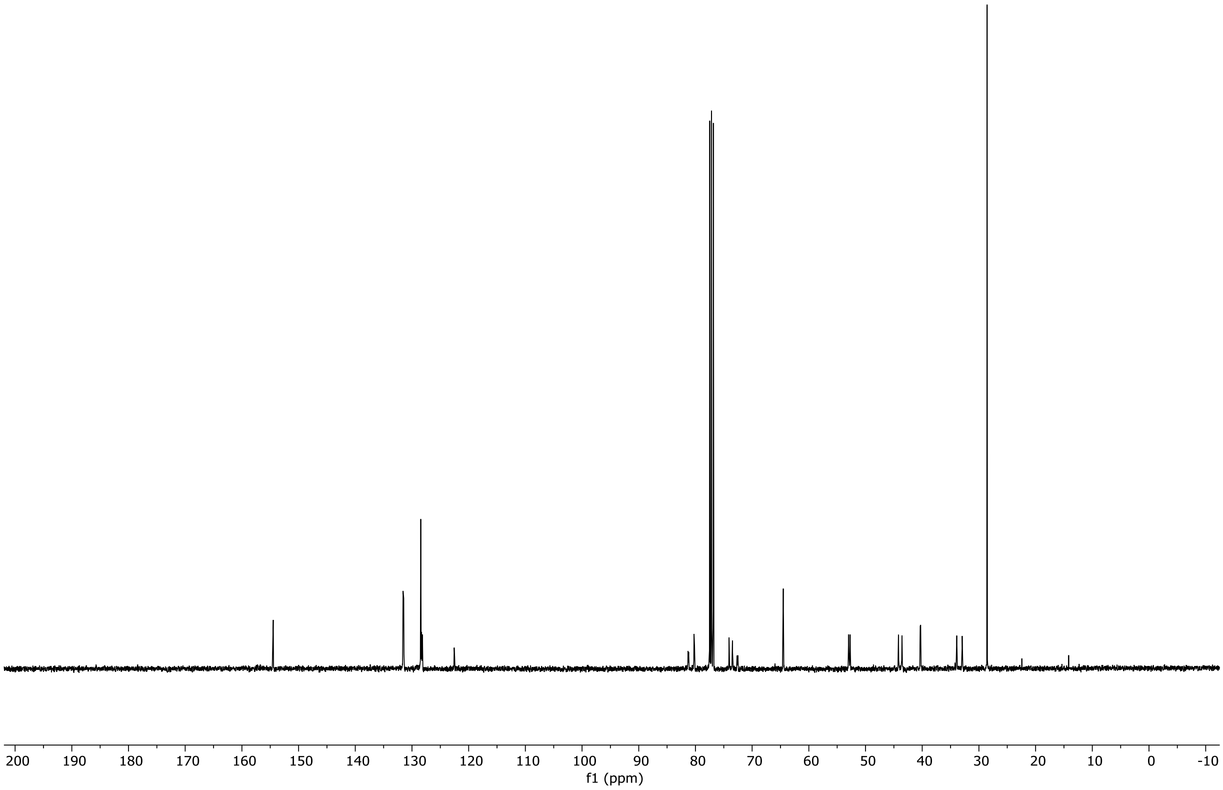

Supplementary Fig 100. ^{1}H (top) and ^{13}C (bottom) NMR spectra of compound **S2ai**.

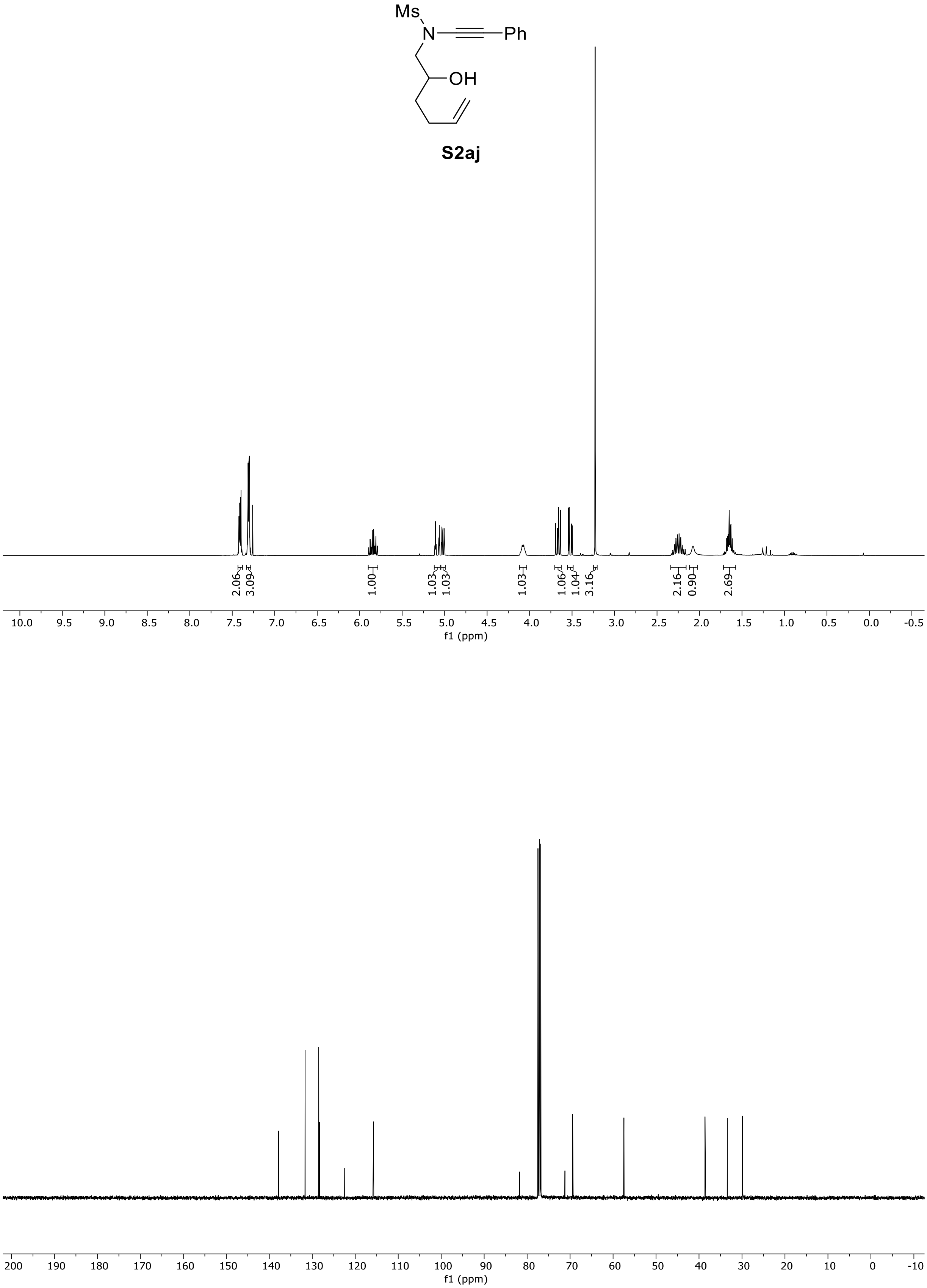

Supplementary Fig 101. ^{1}H (top) and ^{13}C (bottom) NMR spectra of compound **S2aj**.

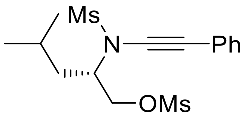

S3v

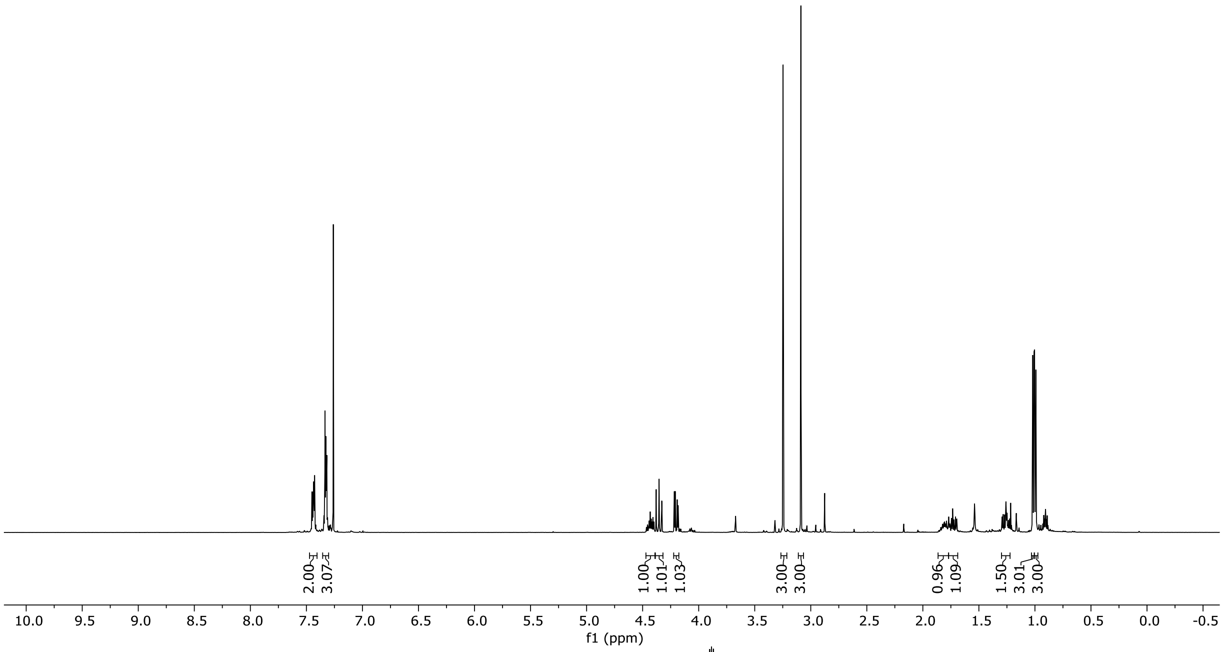

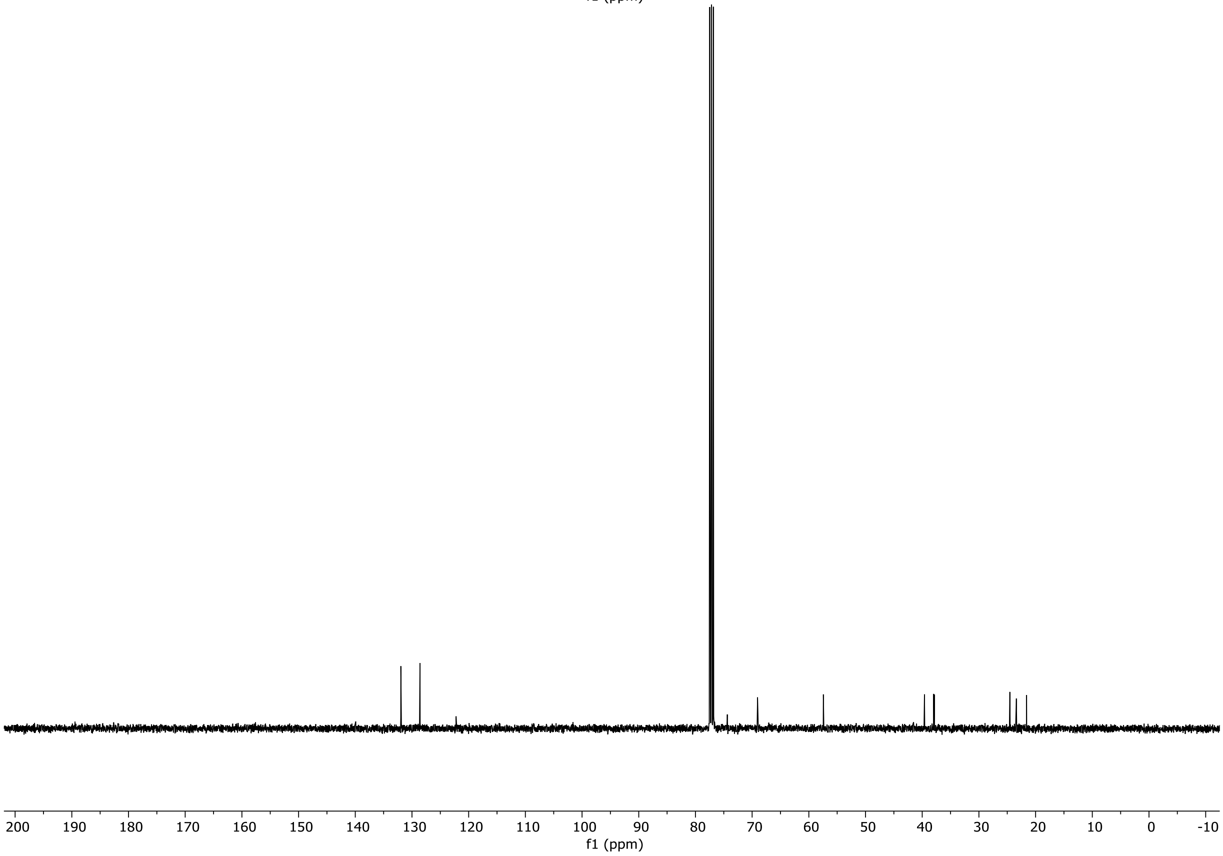

Supplementary Fig 102. ^{1}H (top) and ^{13}C (bottom) NMR spectra of compound **S3v**.

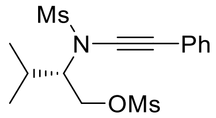

S3w

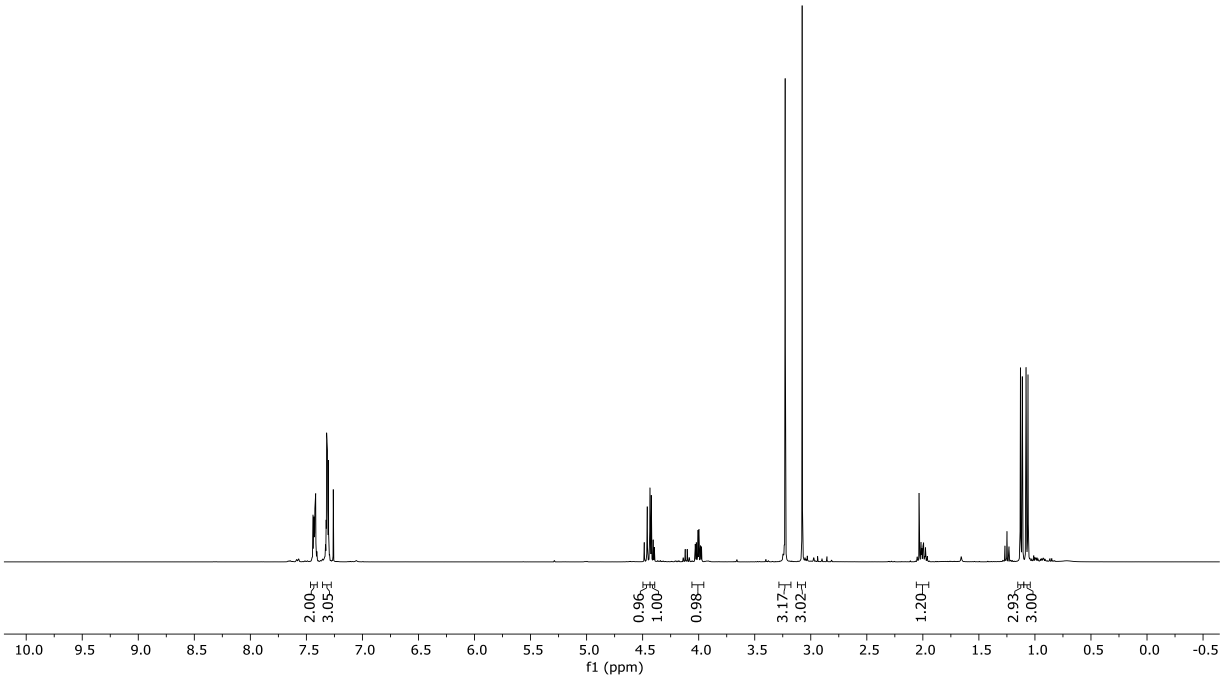

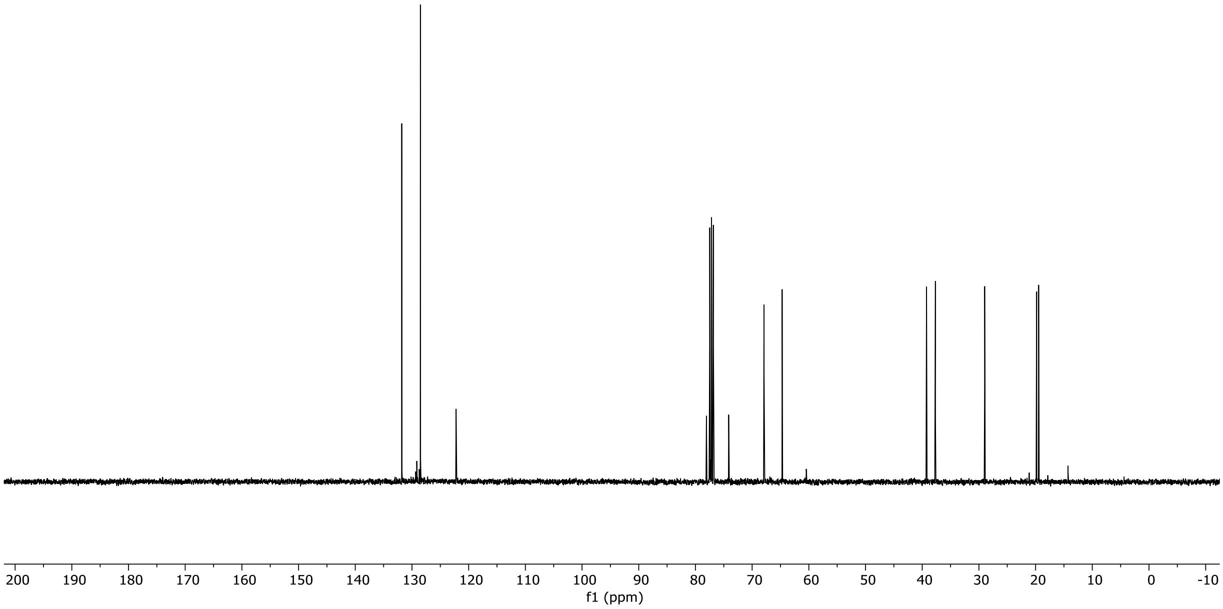

Supplementary Fig 103. ^{1}H (top) and ^{13}C (bottom) NMR spectra of compound **S3w**.

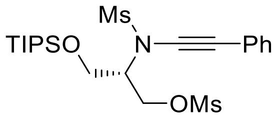

S3x

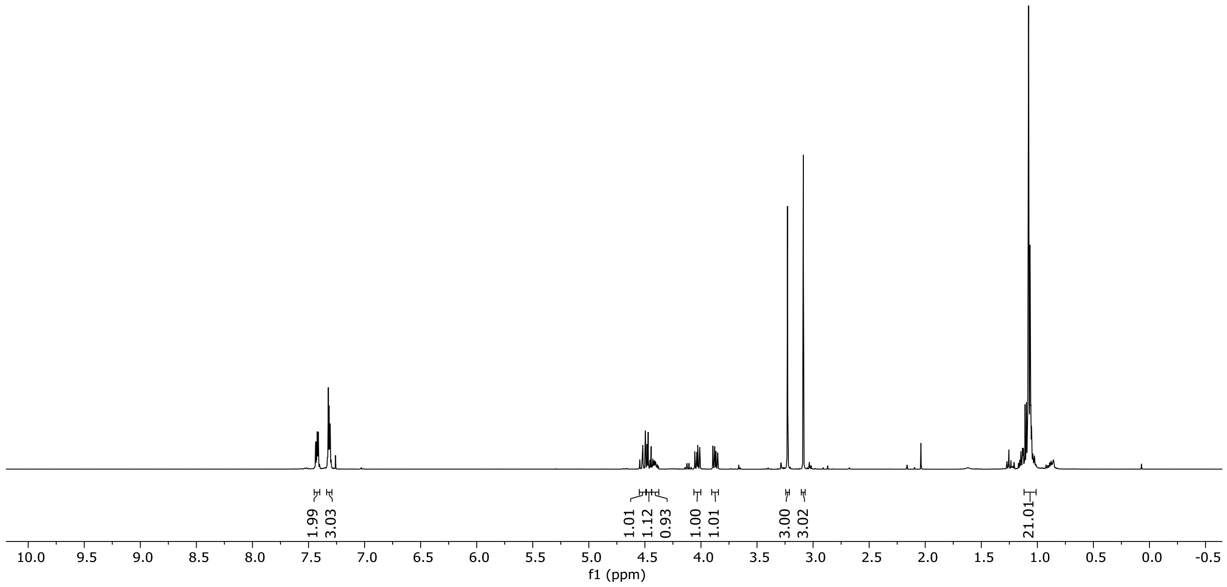

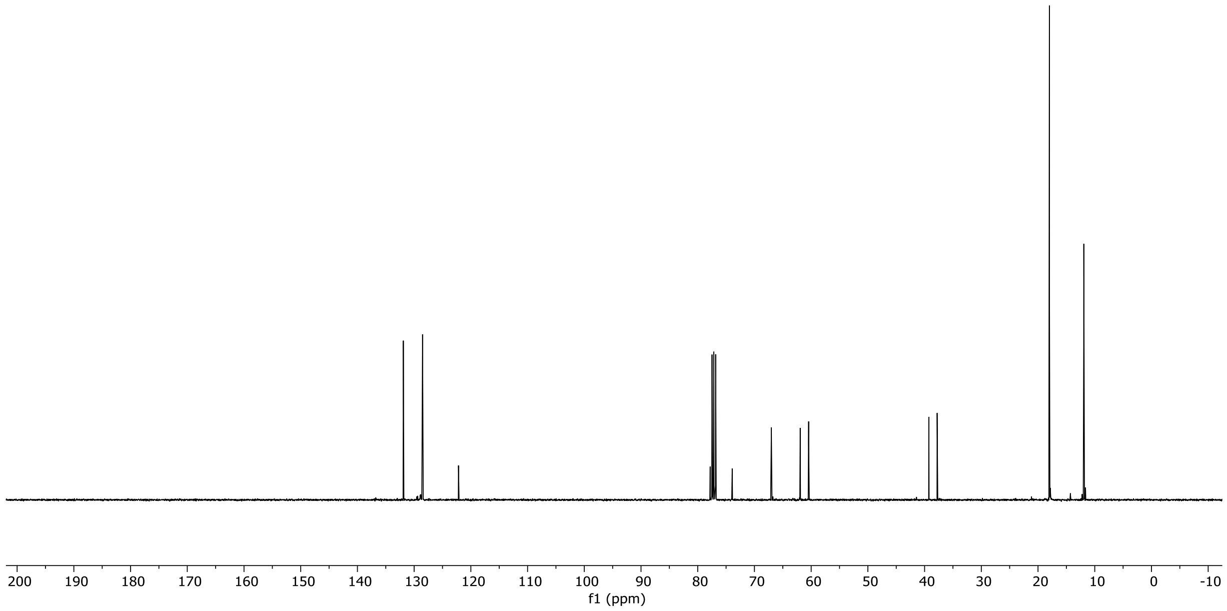

Supplementary Fig 104. ^{1}H (top) and ^{13}C (bottom) NMR spectra of compound **S3x**.

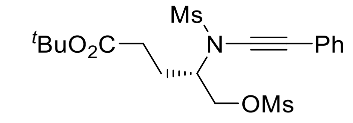

S3y

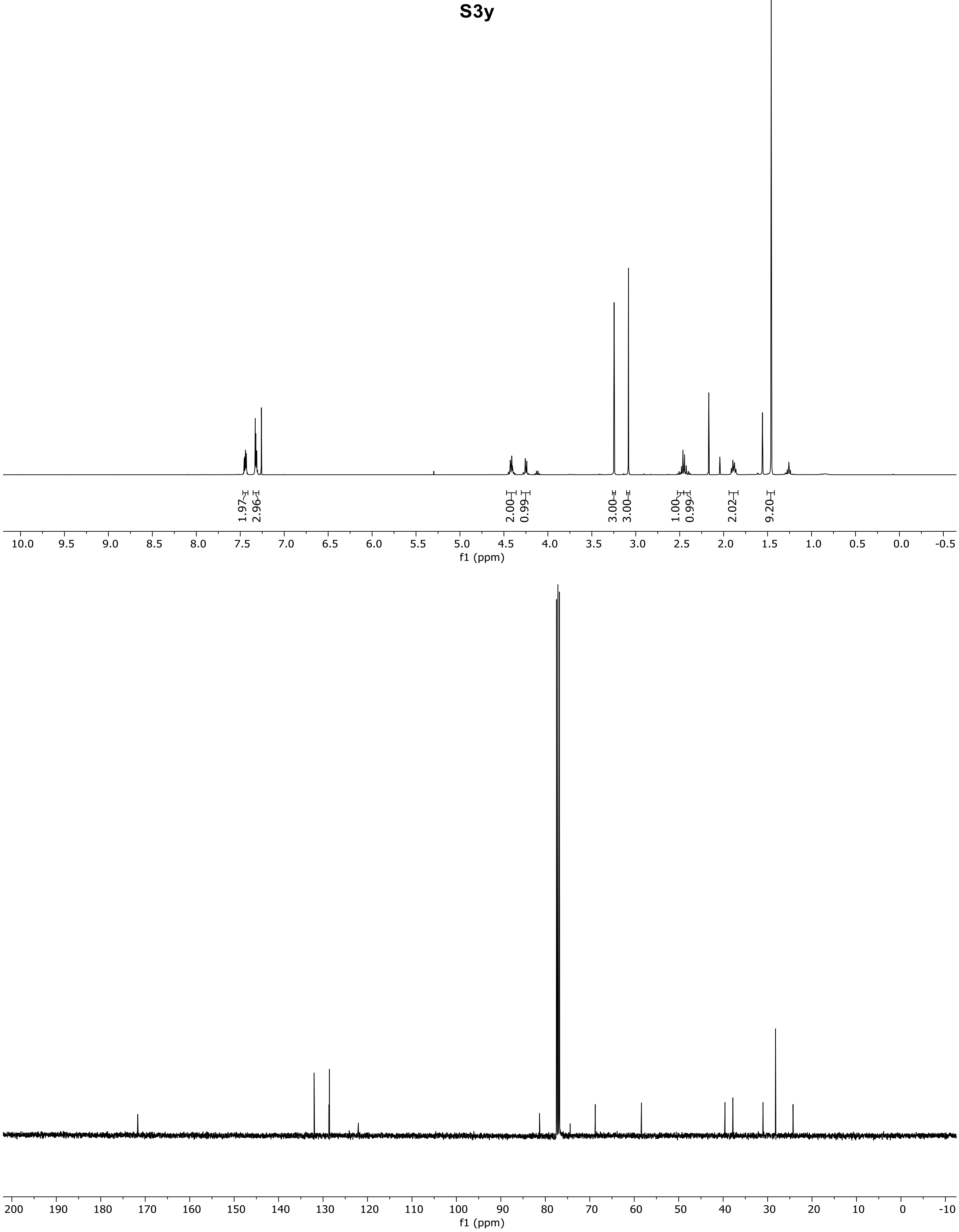

Supplementary Fig 105. ^{1}H (top) and ^{13}C (bottom) NMR spectra of compound **S3y**.

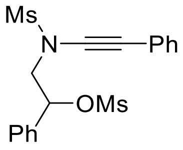

S3ae

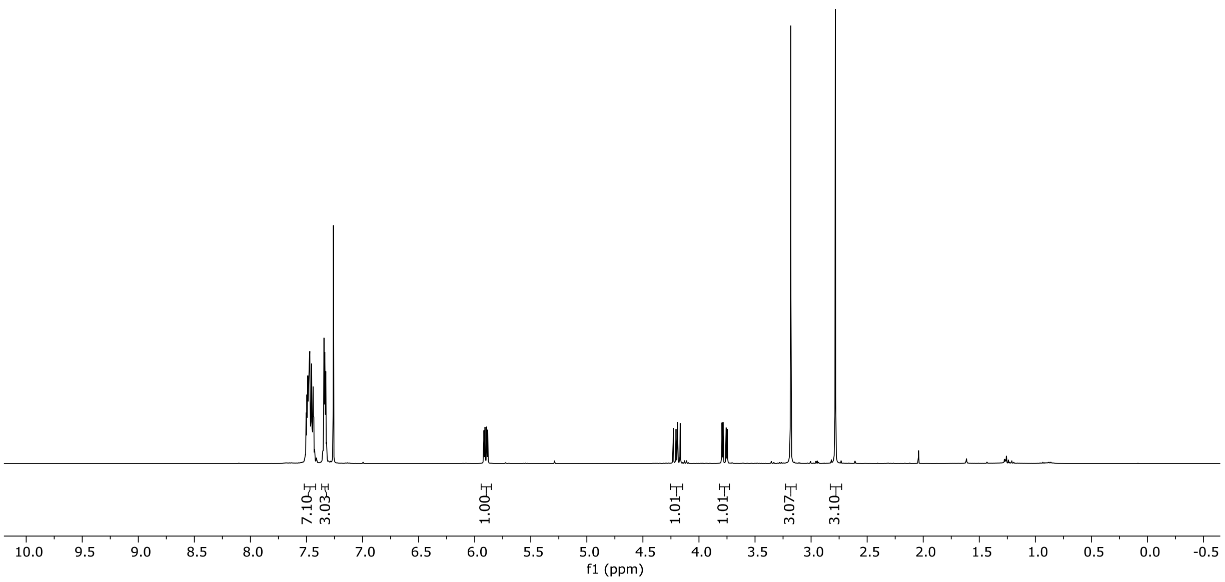

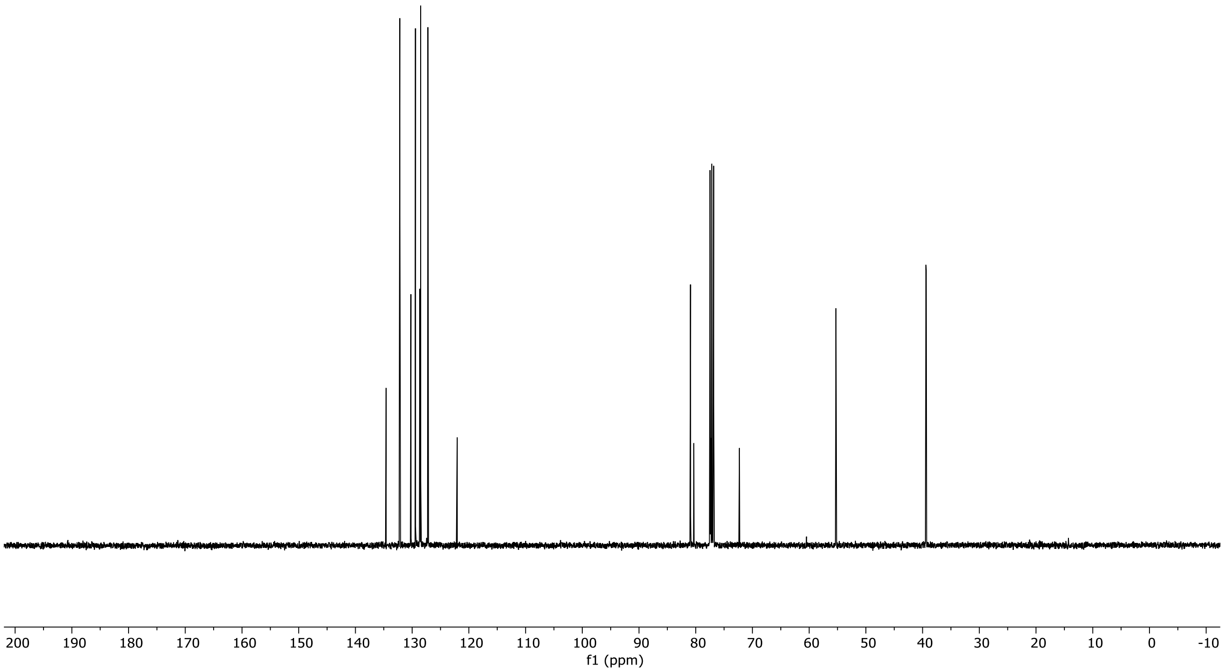

Supplementary Fig 106. ^{1}H (top) and ^{13}C (bottom) NMR spectra of compound **S3ae**.

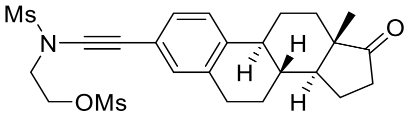

S3ah

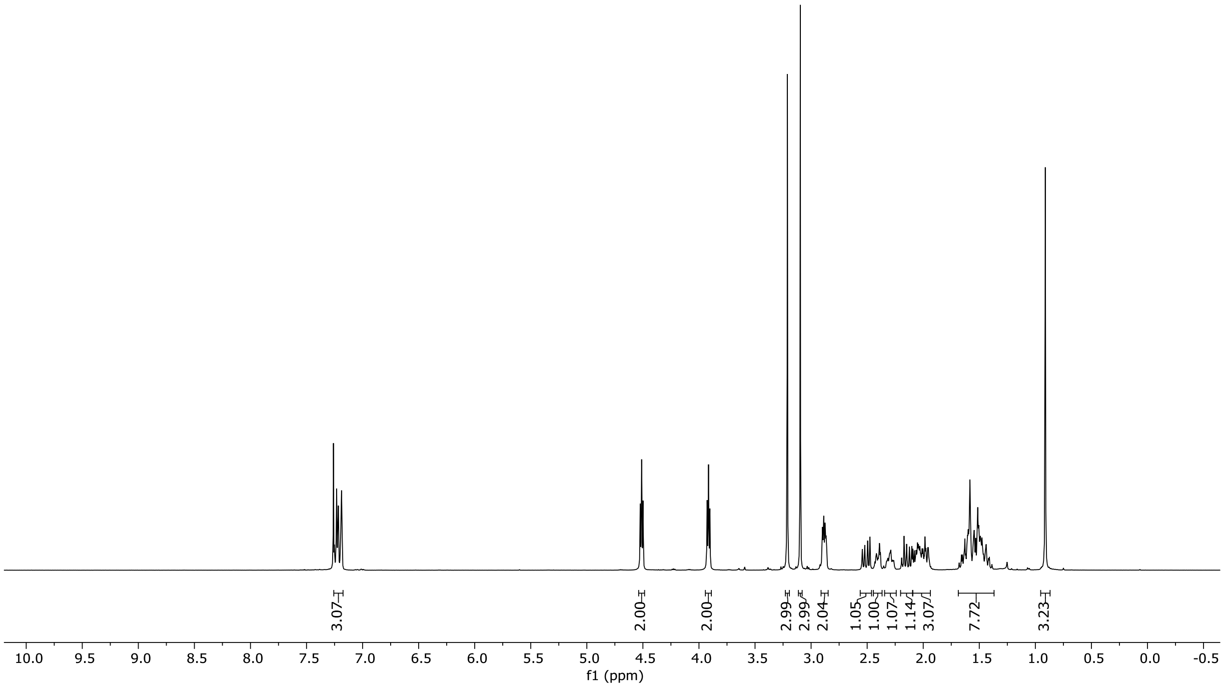

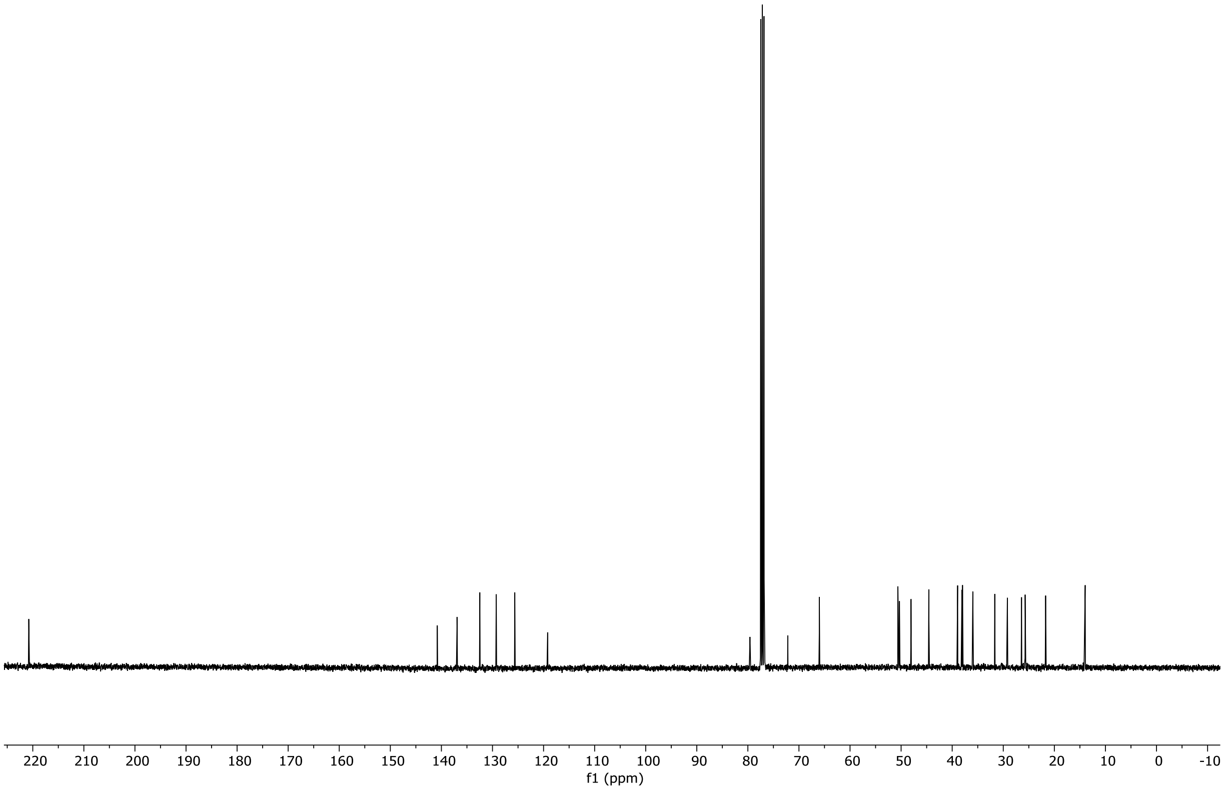

Supplementary Fig 107. ^{1}H (top) and ^{13}C (bottom) NMR spectra of compound **S3ah**.

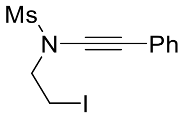

1a

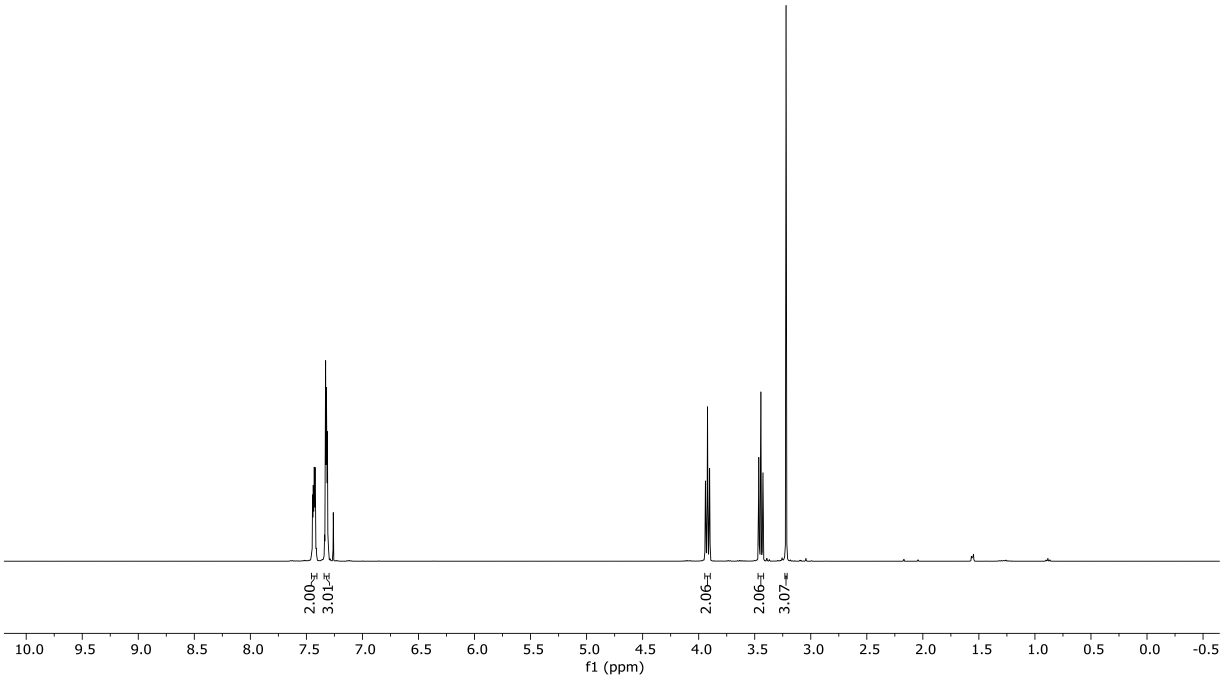

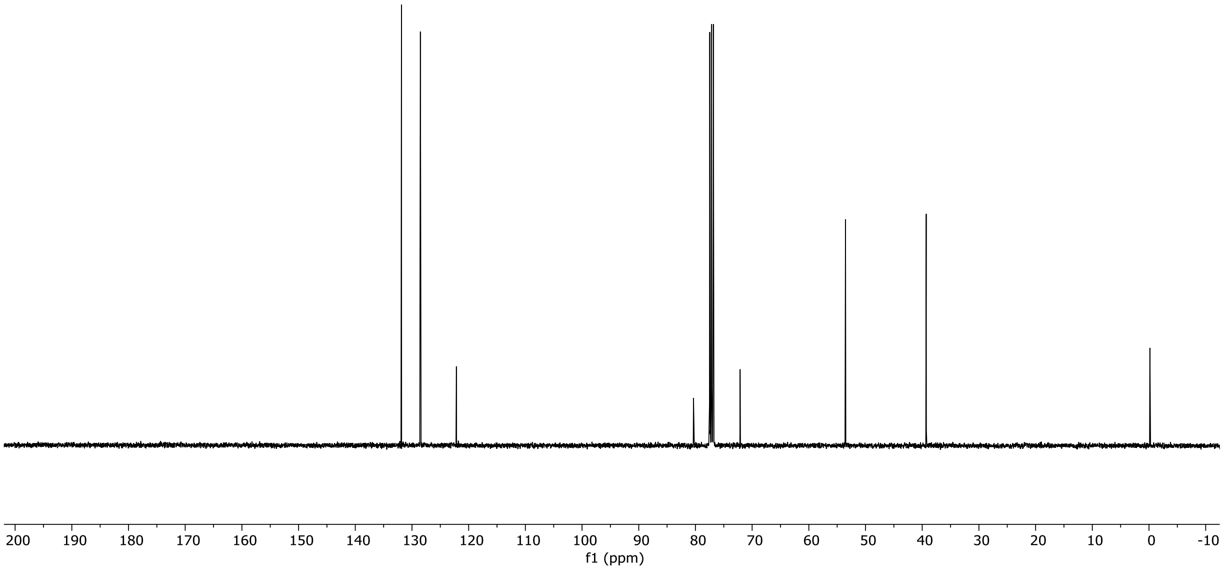

Supplementary Fig 108. ^{1}H (top) and ^{13}C (bottom) NMR spectra of compound **1a**.

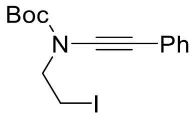

1b

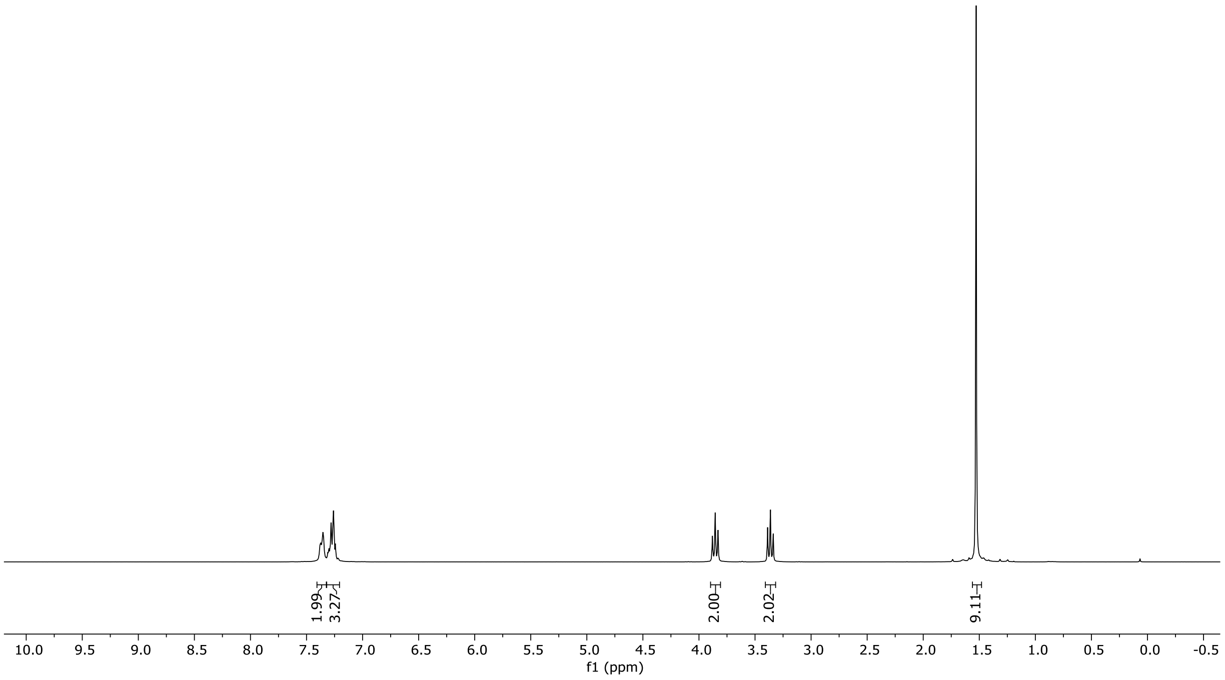

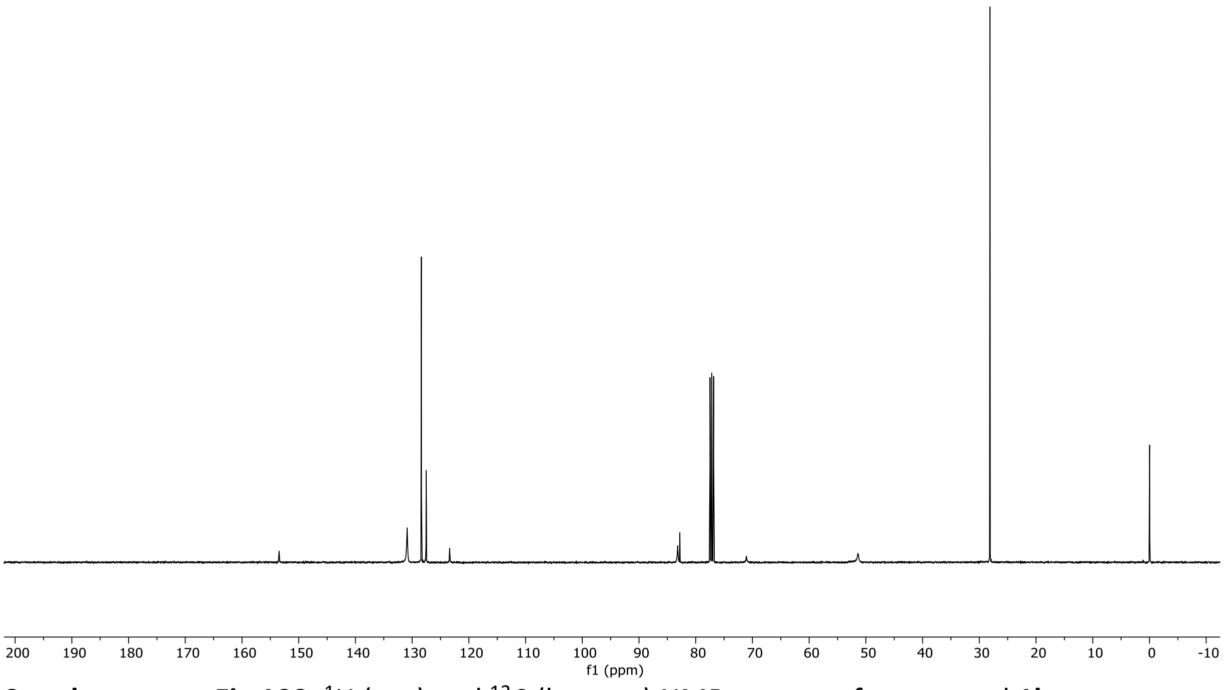

Supplementary Fig 109. ^{1}H (top) and ^{13}C (bottom) NMR spectra of compound **1b**.

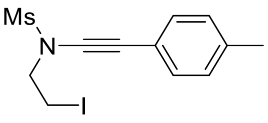

1c

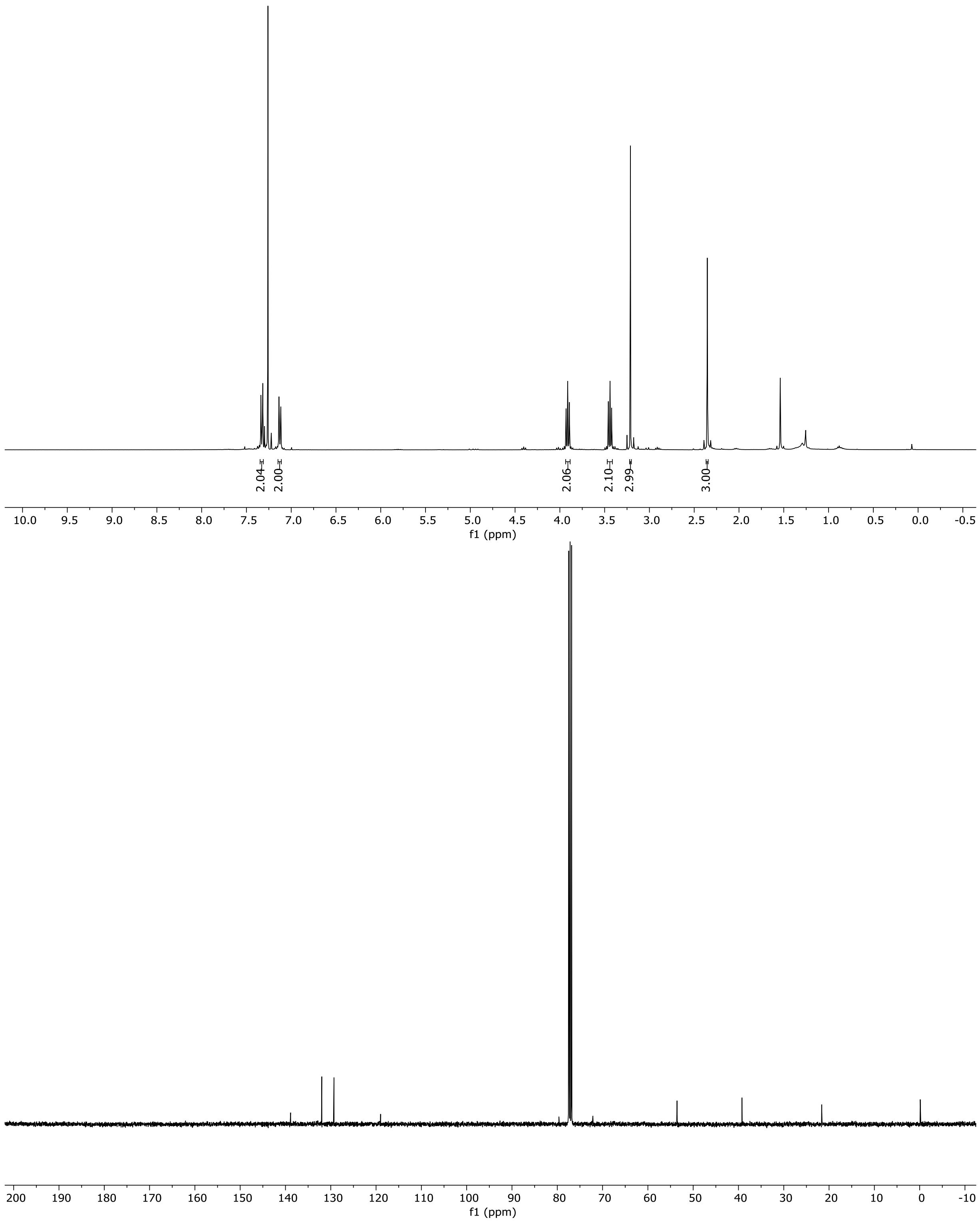

Supplementary Fig 110. ^{1}H (top) and ^{13}C (bottom) NMR spectra of compound **1c**.

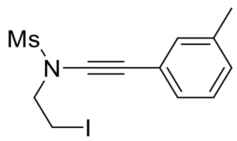

1d

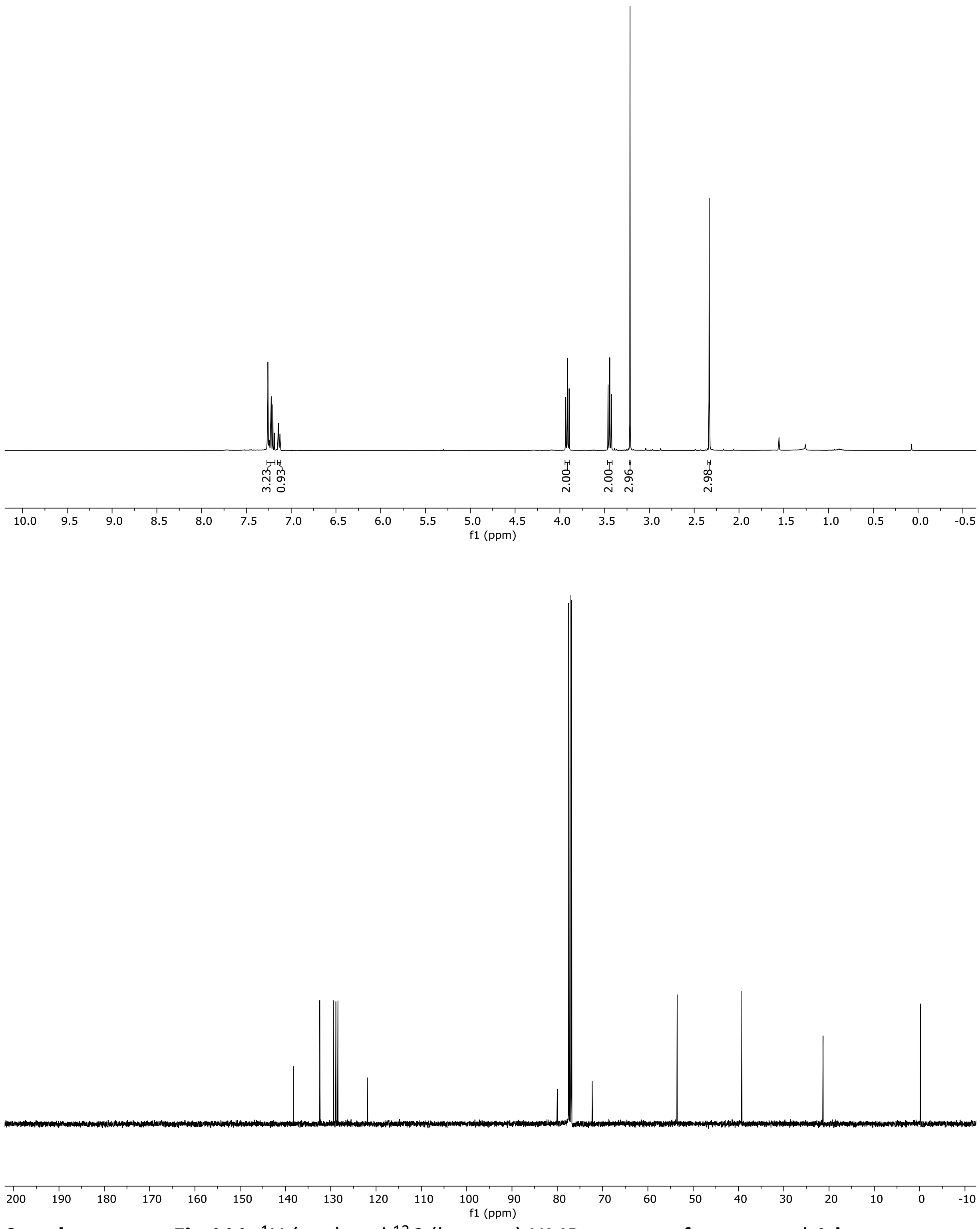

Supplementary Fig 111. ^{1}H (top) and ^{13}C (bottom) NMR spectra of compound **1d**.

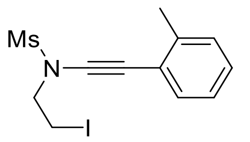

1e

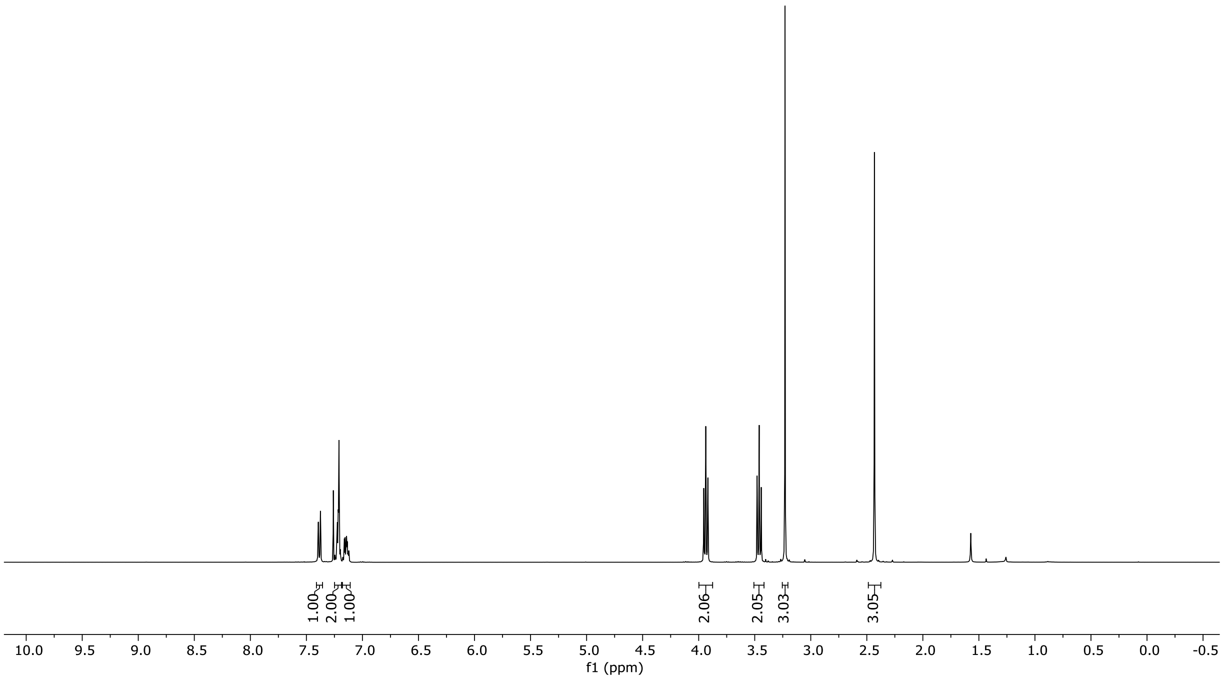

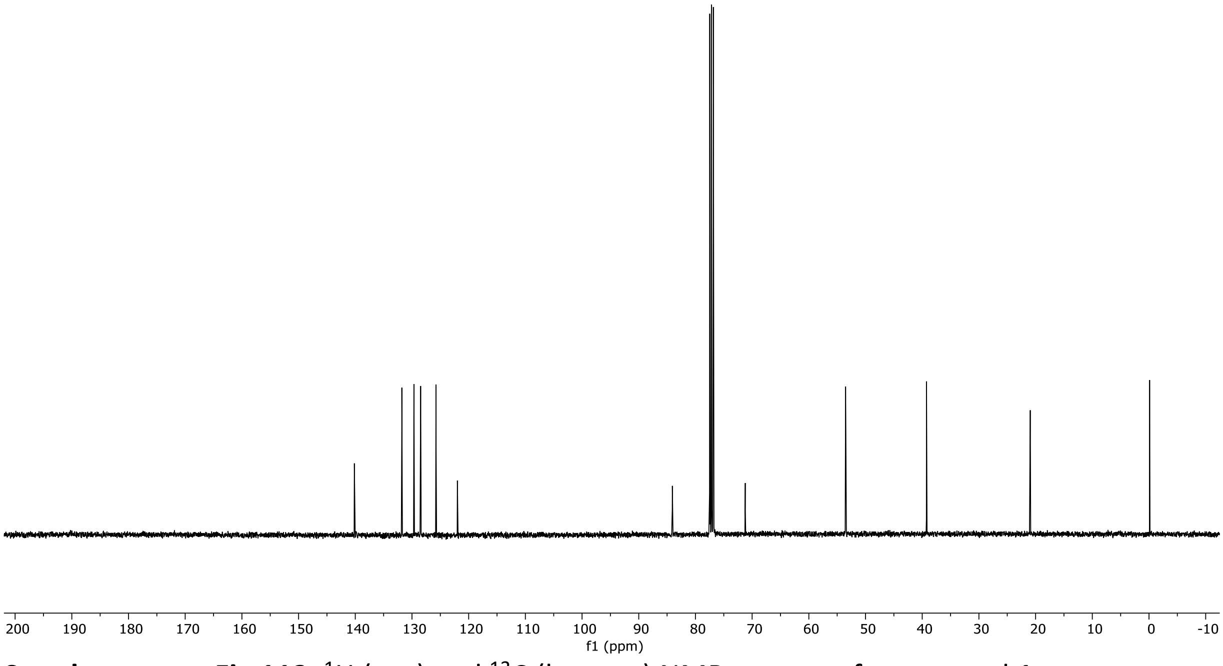

Supplementary Fig 112. ^{1}H (top) and ^{13}C (bottom) NMR spectra of compound **1e**.

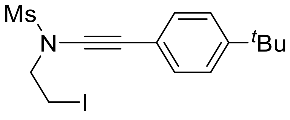

1f

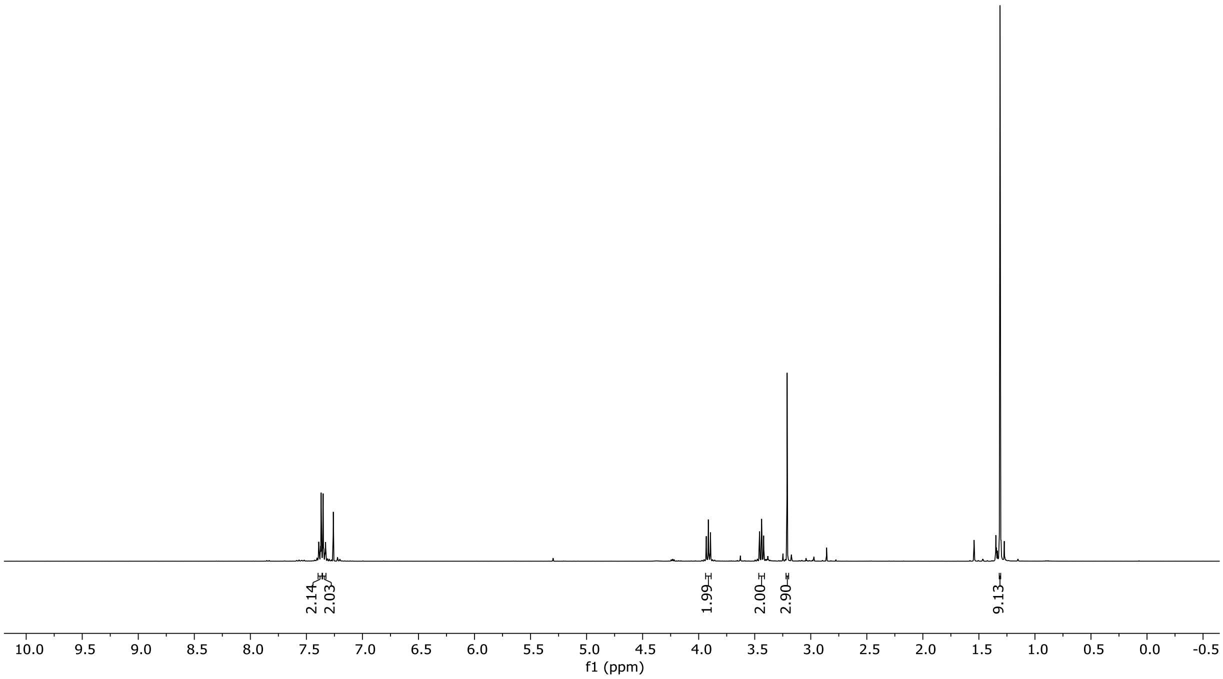

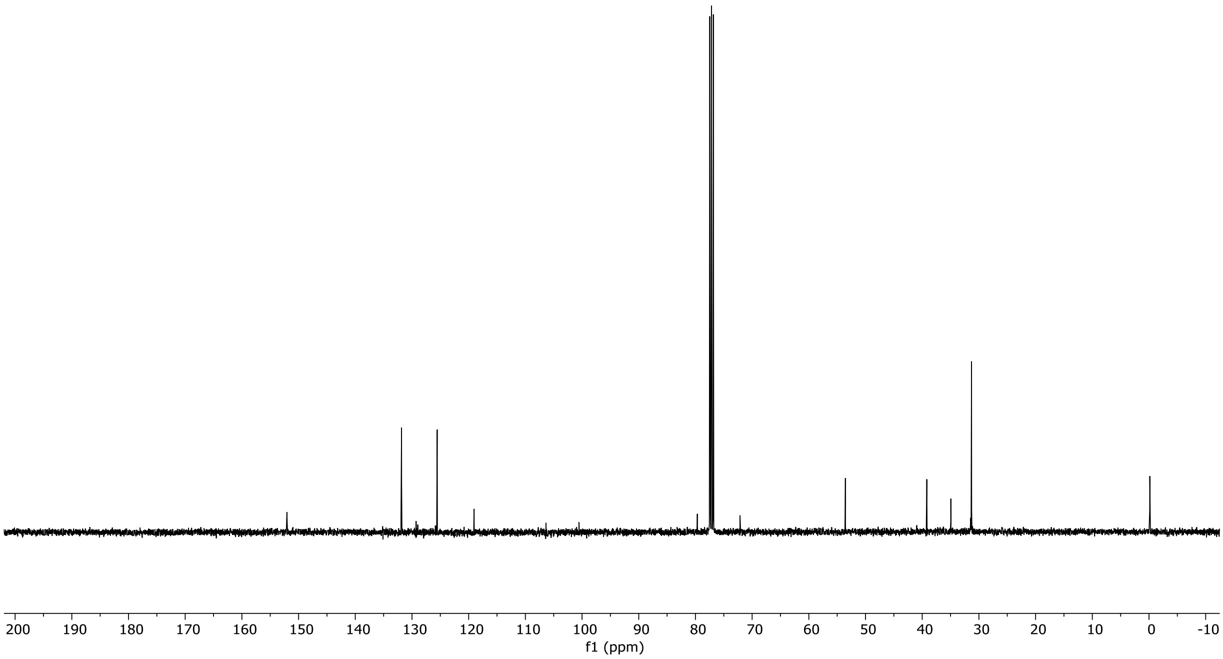

Supplementary Fig 113. ^{1}H (top) and ^{13}C (bottom) NMR spectra of compound **1f**.

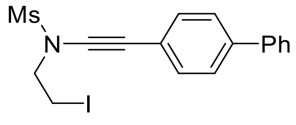

1g

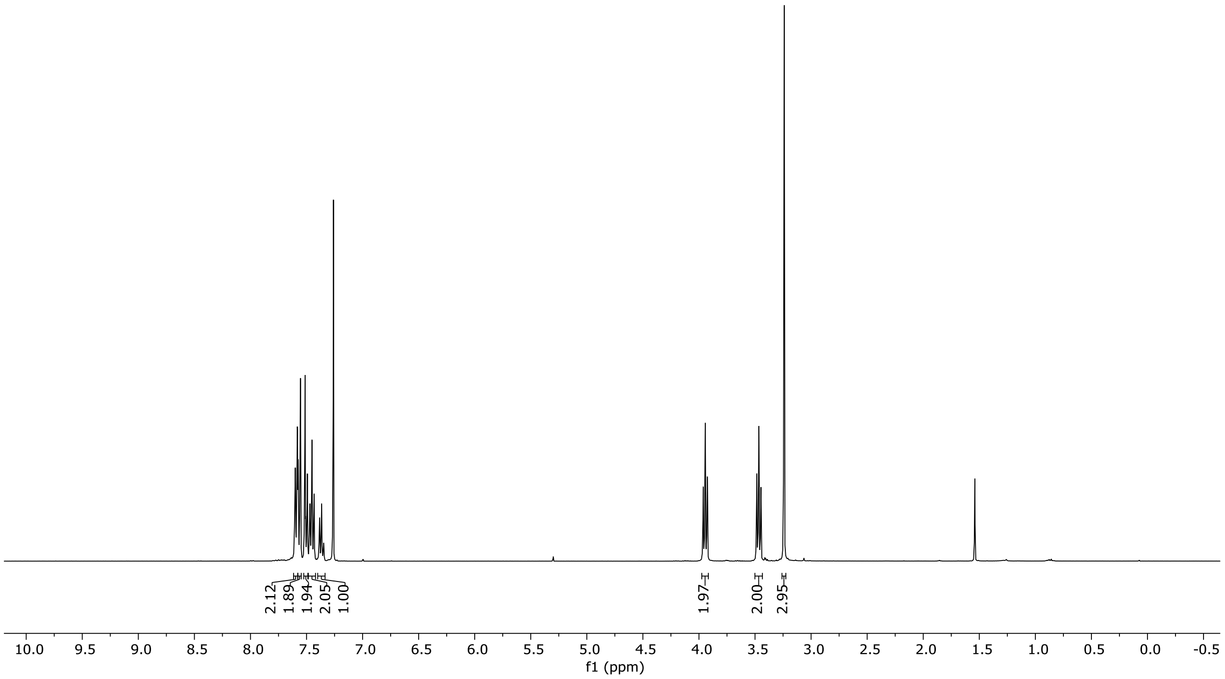

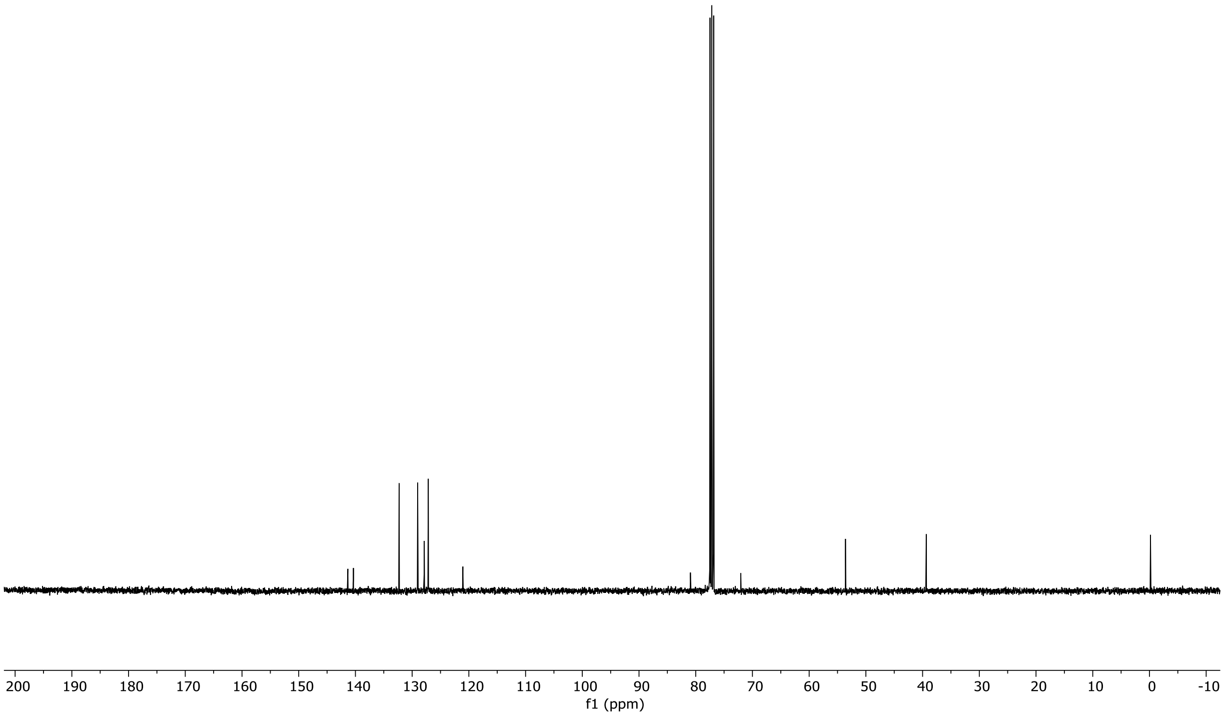

Supplementary Fig 114. ^{1}H (top) and ^{13}C (bottom) NMR spectra of compound **1g**.

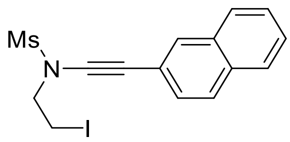

1h

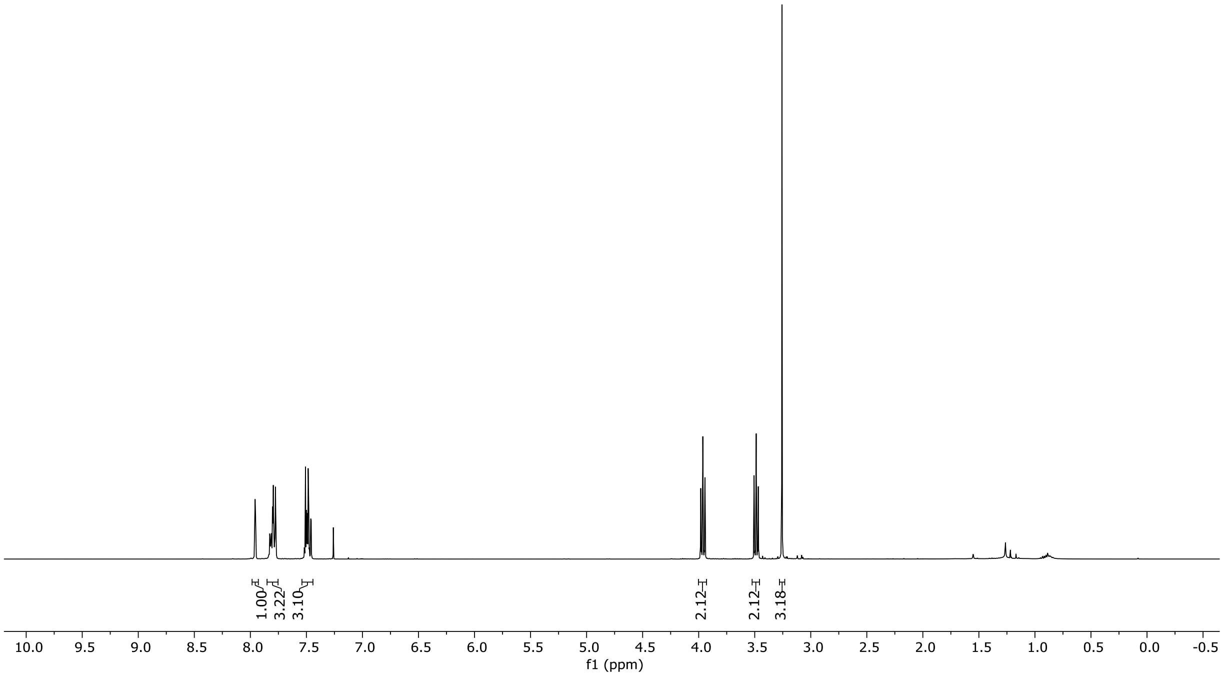

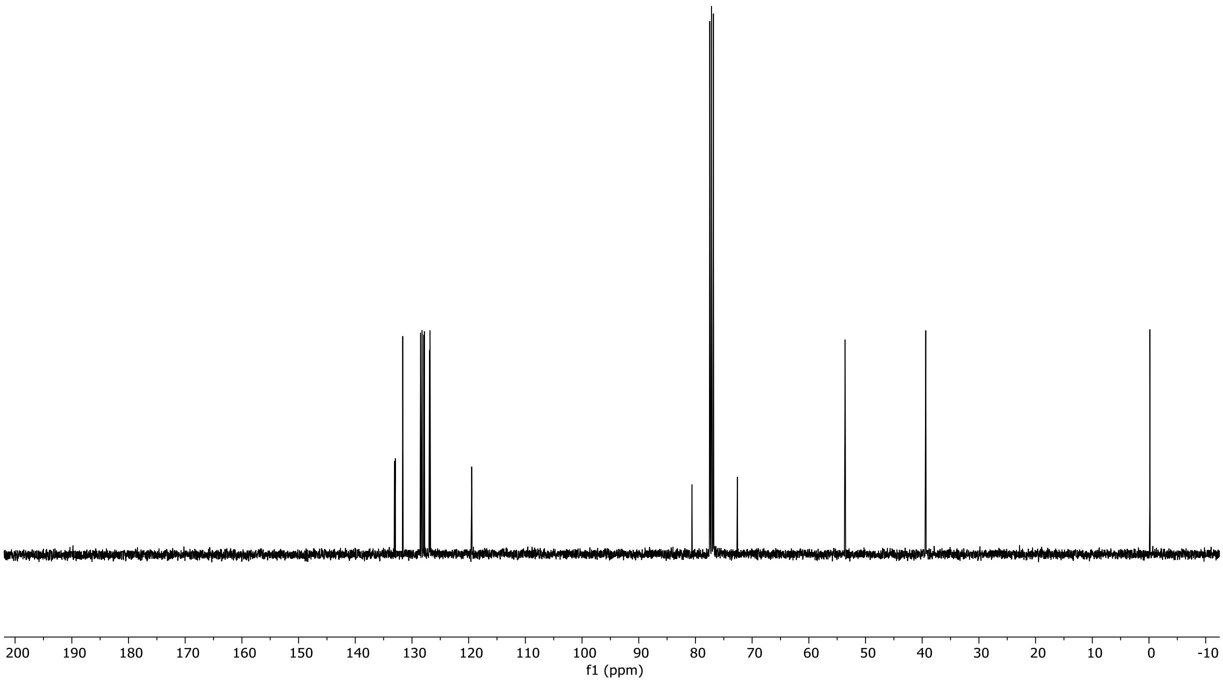

Supplementary Fig 115. ^{1}H (top) and ^{13}C (bottom) NMR spectra of compound **1h**.

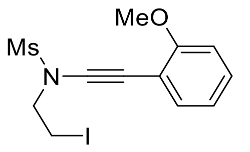

1i

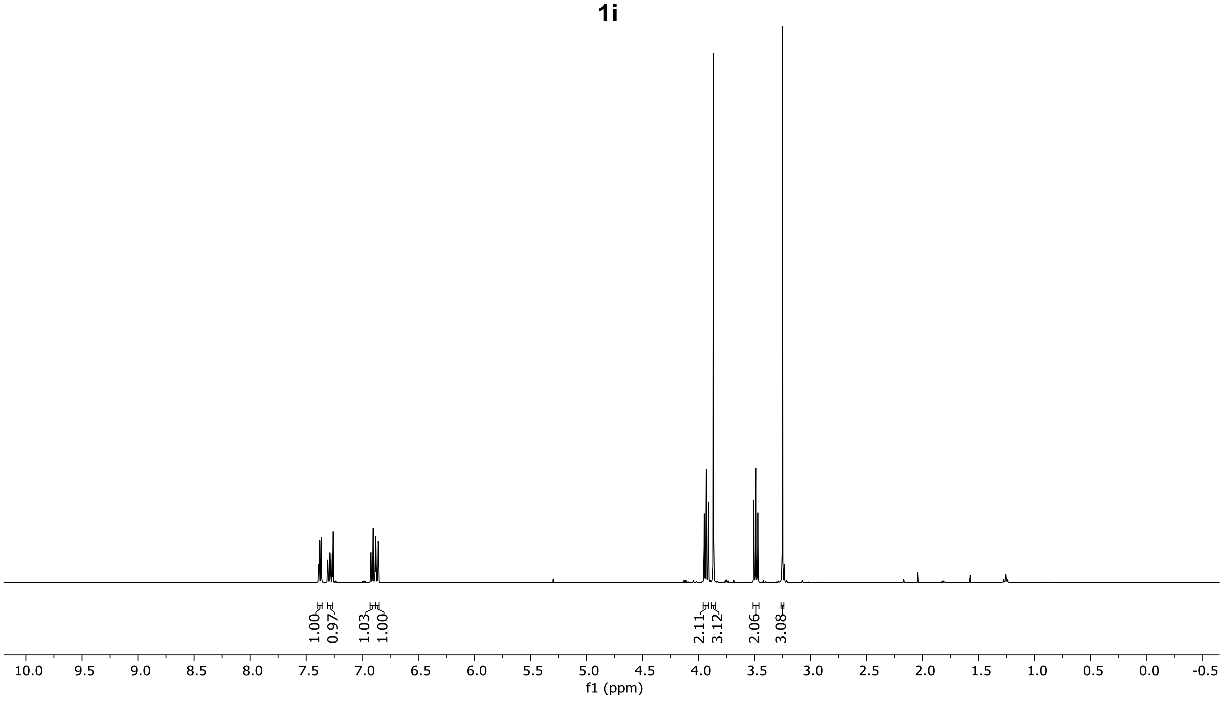

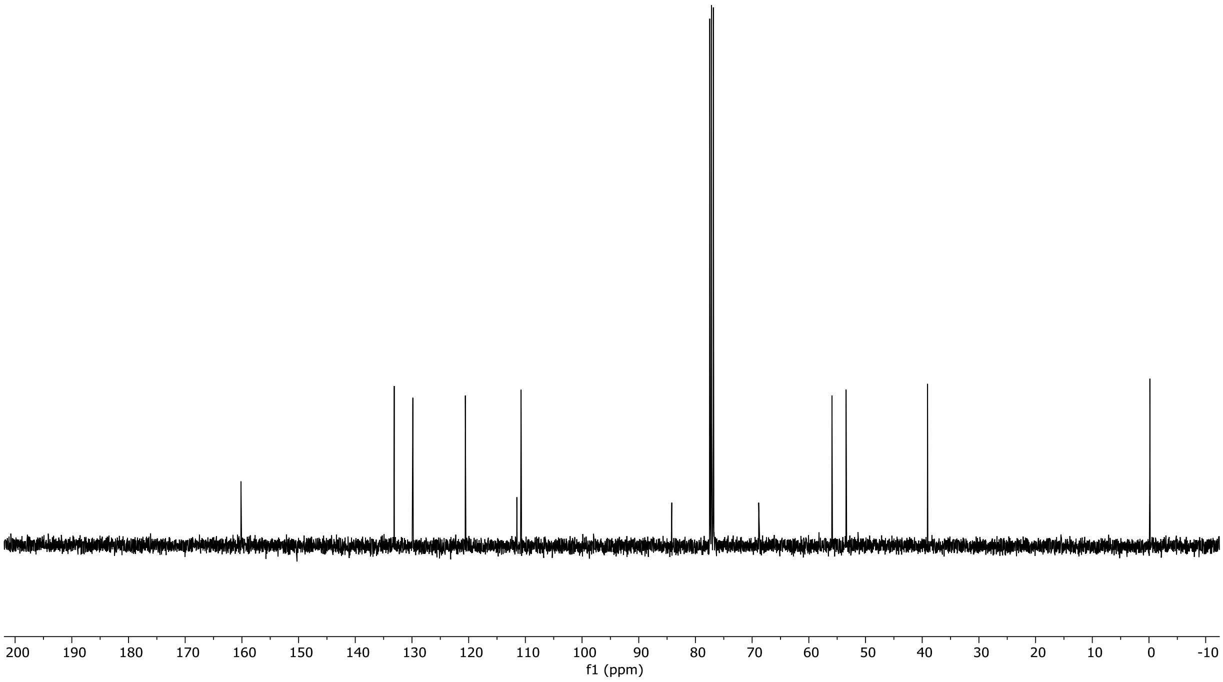

Supplementary Fig 116. ^{1}H (top) and ^{13}C (bottom) NMR spectra of compound **1i**.

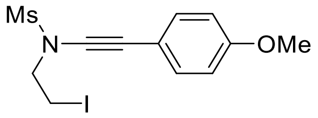

1j

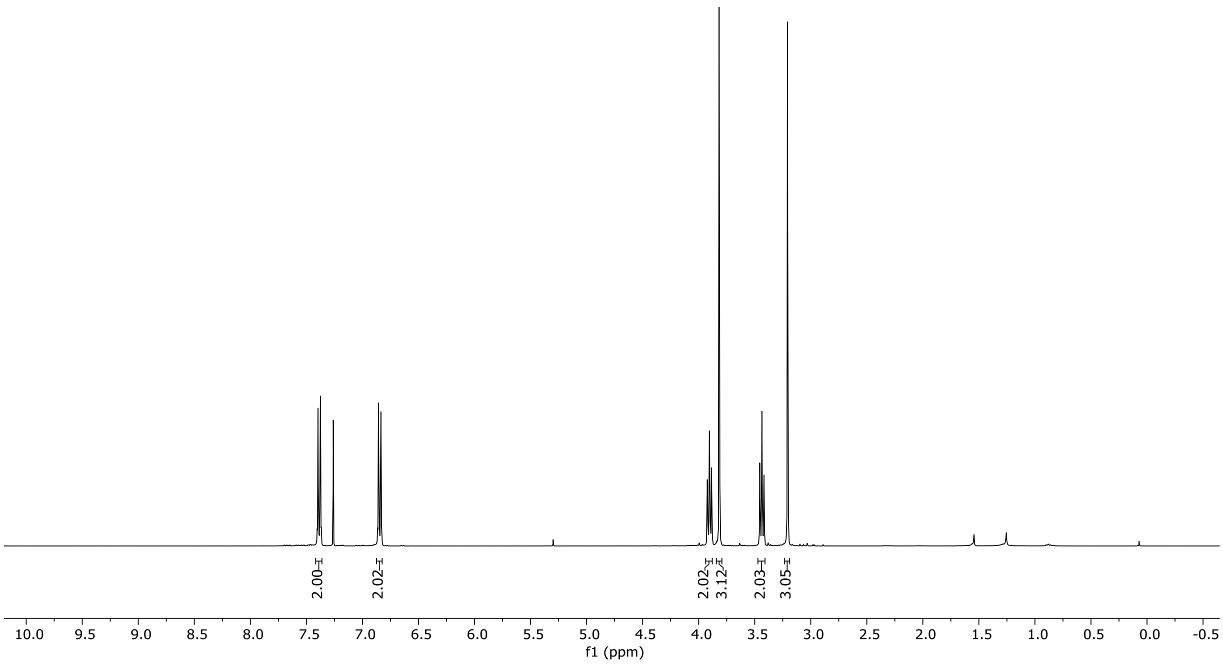

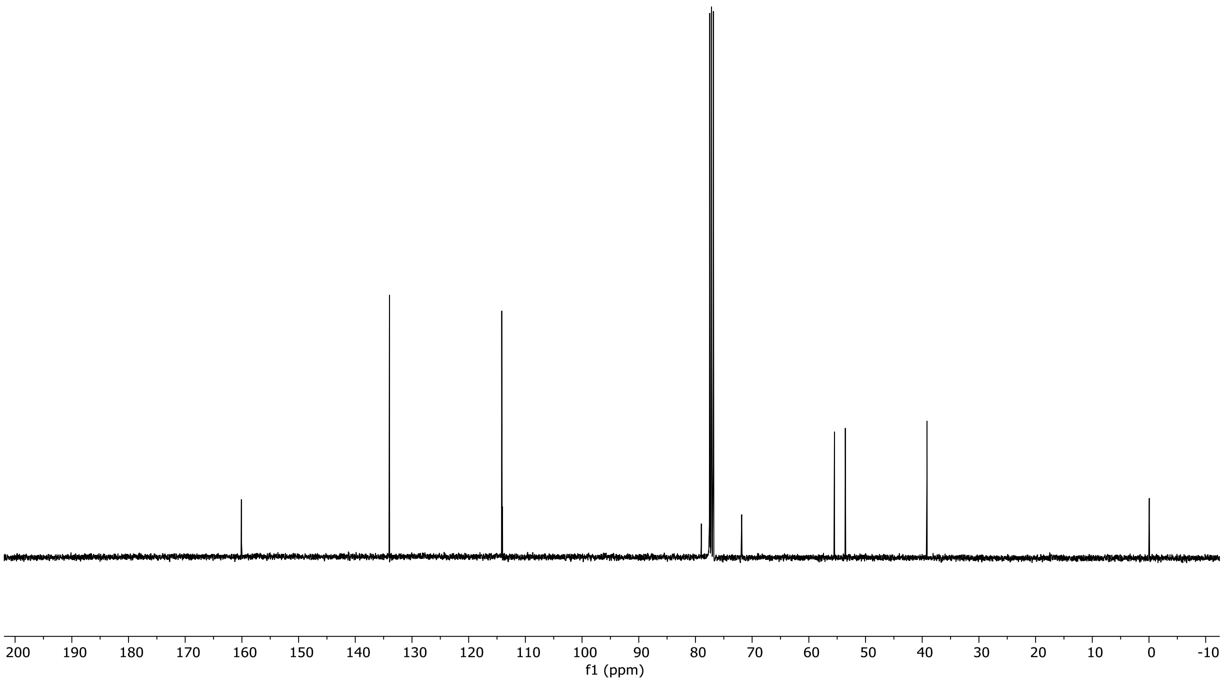

Supplementary Fig 117. ^{1}H (top) and ^{13}C (bottom) NMR spectra of compound **1j**.

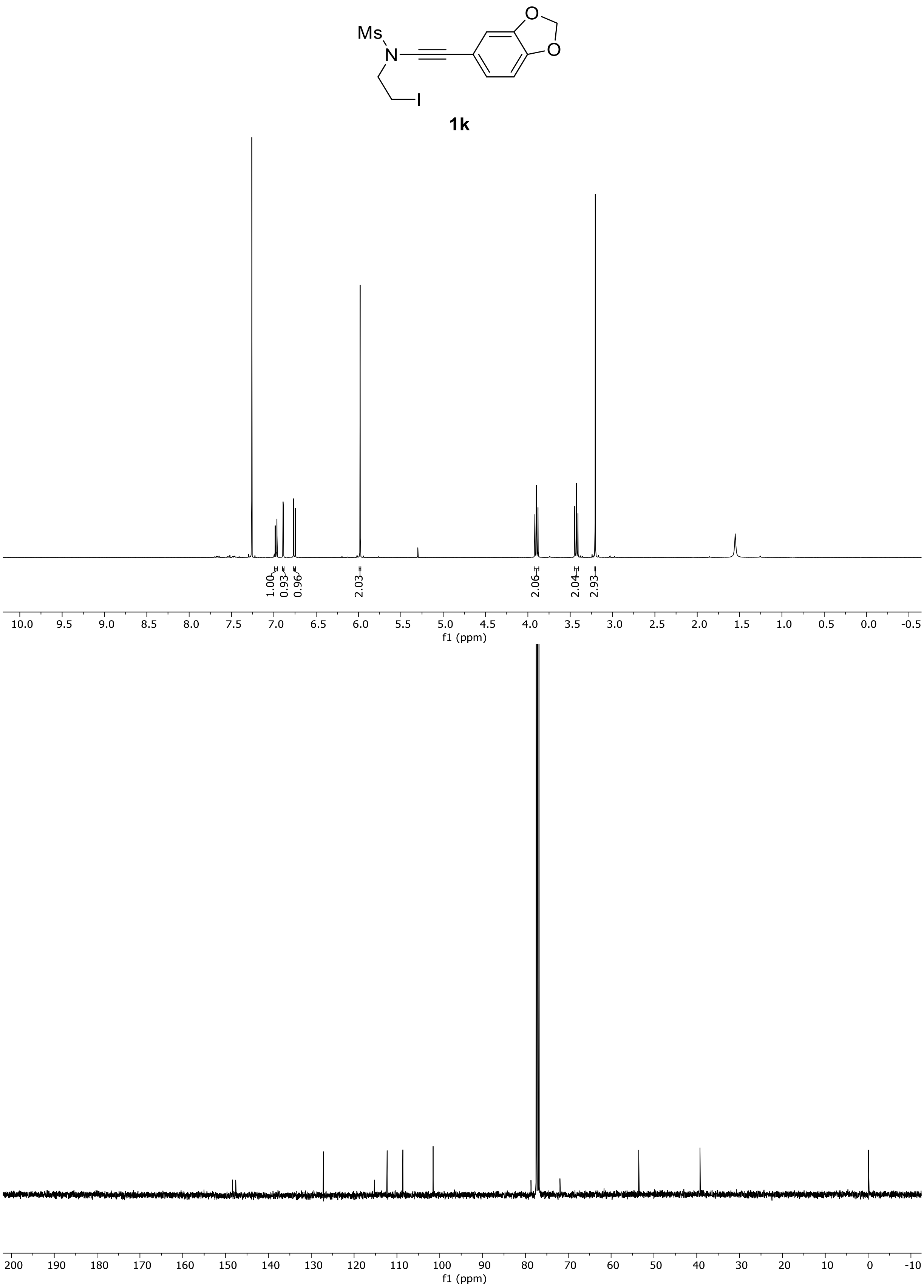

Supplementary Fig 118. ^{1}H (top) and ^{13}C (bottom) NMR spectra of compound **1k**.

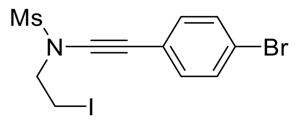

1l

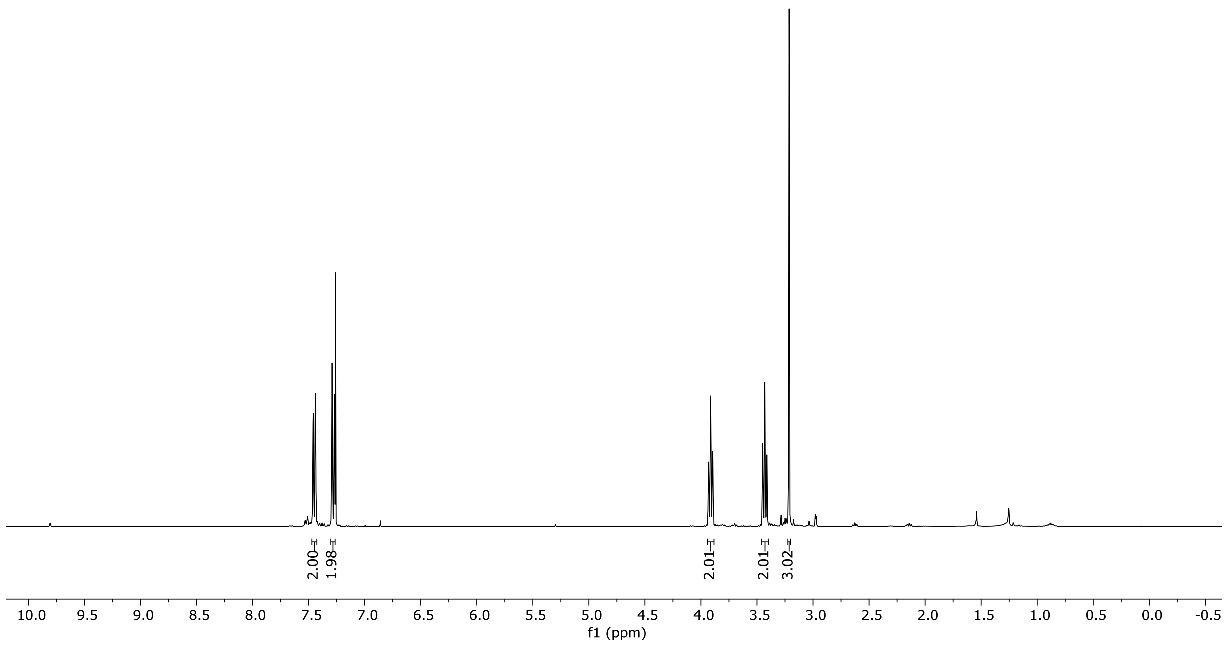

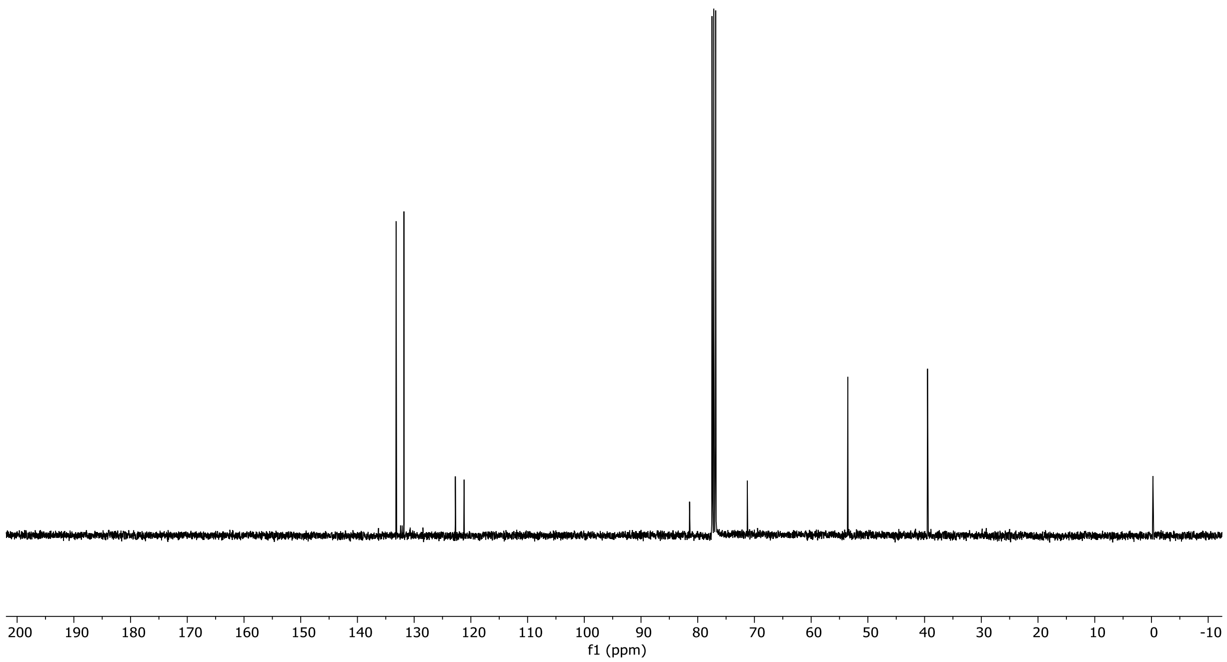

Supplementary Fig 119. ^{1}H (top) and ^{13}C (bottom) NMR spectra of compound **1l**.

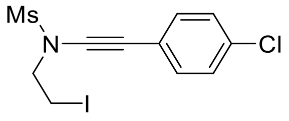

1m

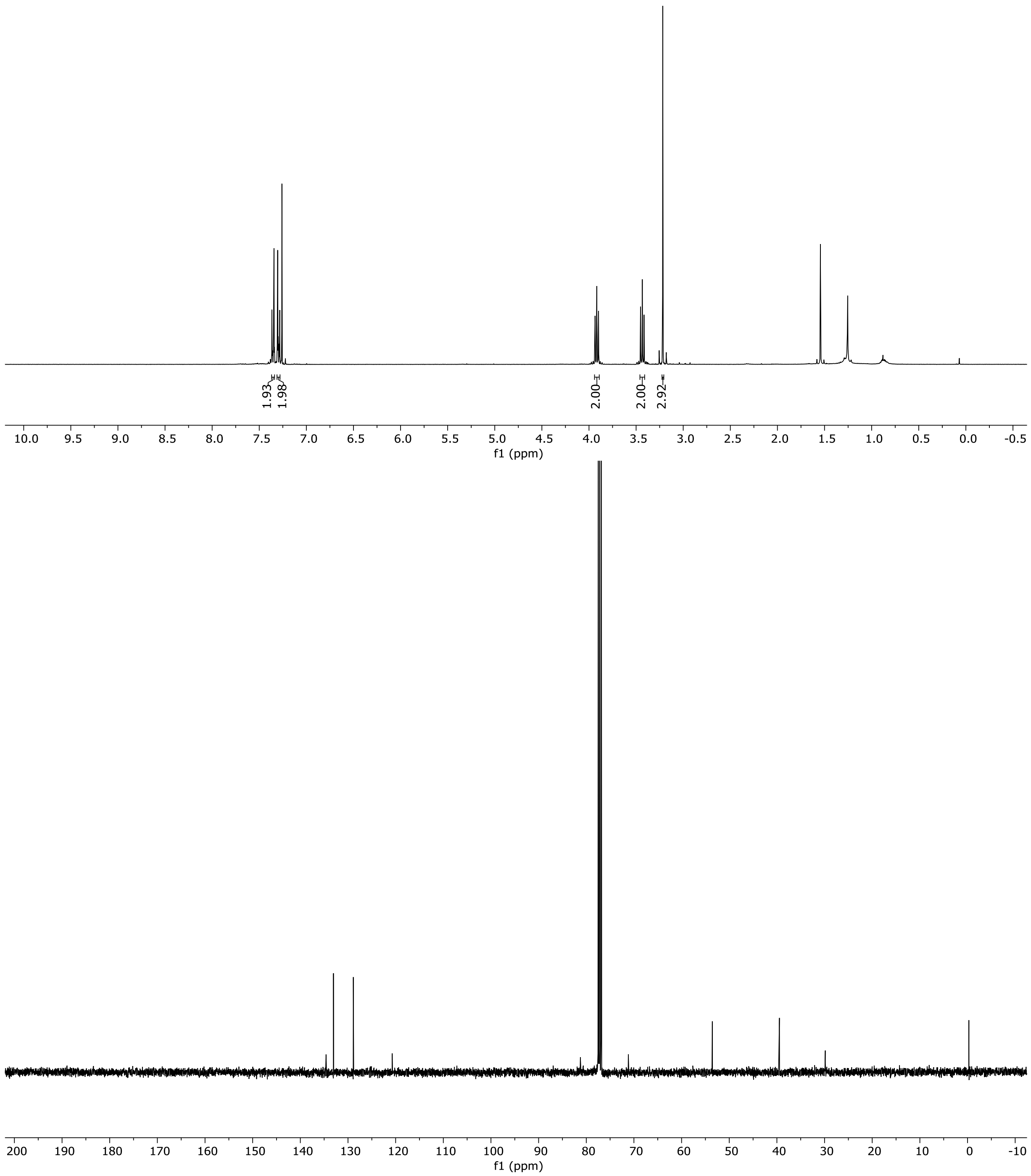

Supplementary Fig 120. ^{1}H (top) and ^{13}C (bottom) NMR spectra of compound **1m**.

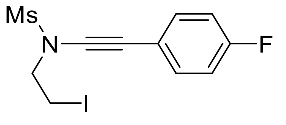

1n

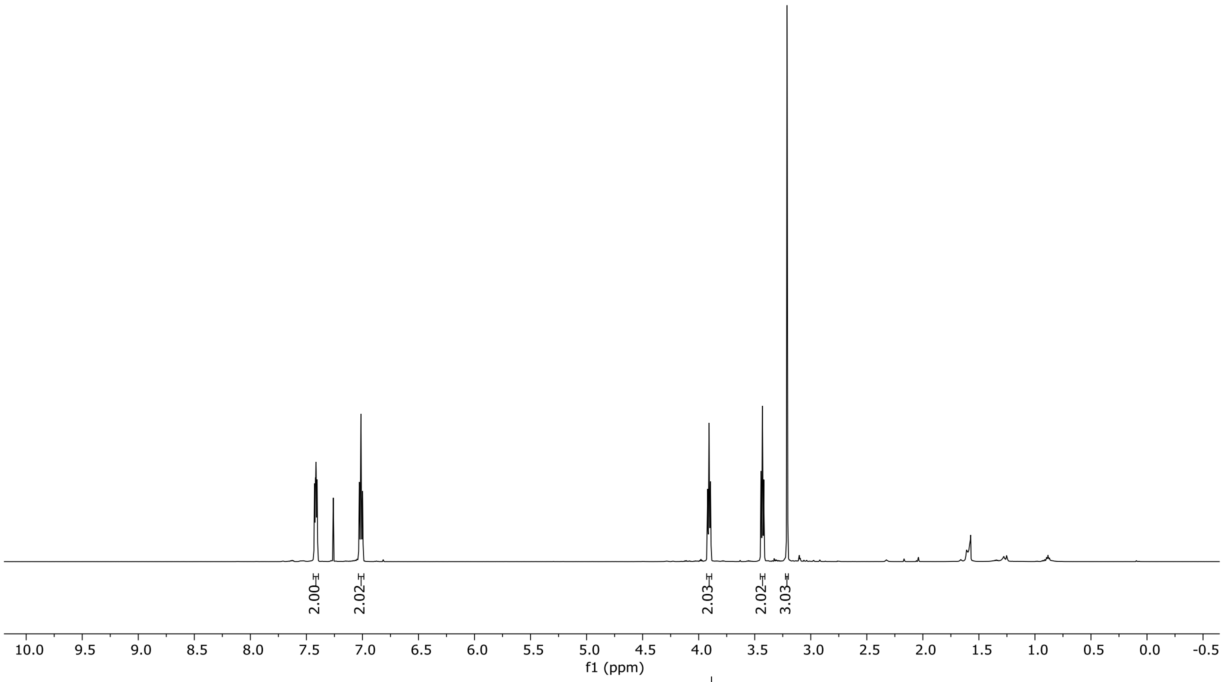

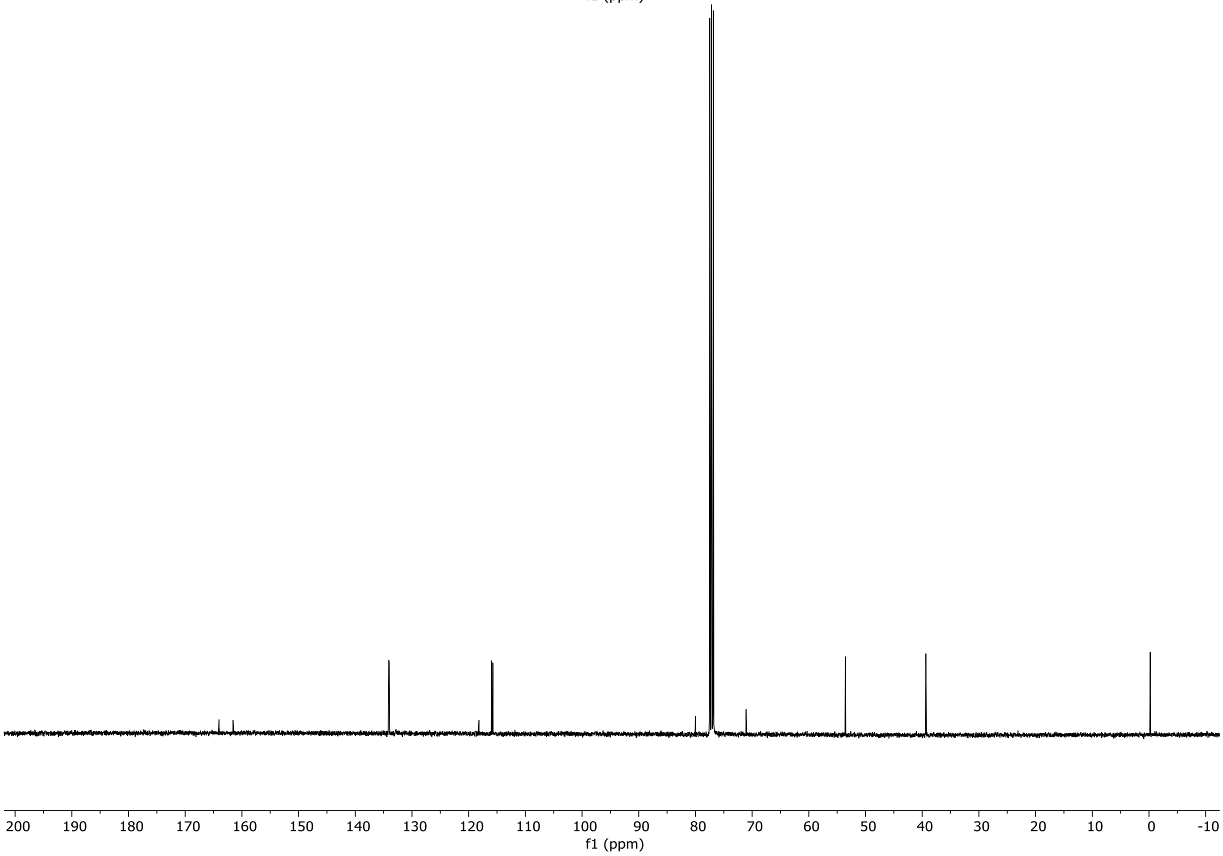

Supplementary Fig 121. ^{1}H (top) and ^{13}C (bottom) NMR spectra of compound **1n**.

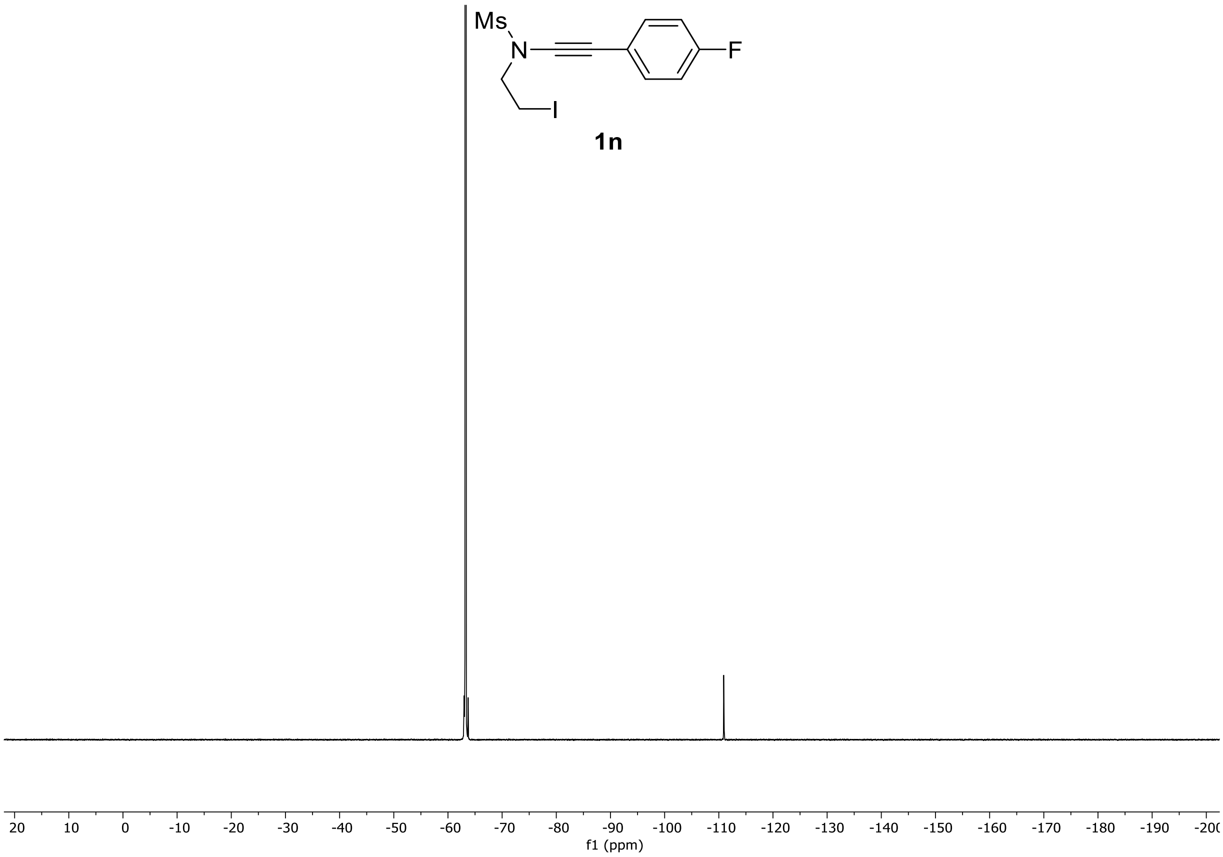

Supplementary Fig 122. ^{19}F NMR spectrum of compound **1n**.

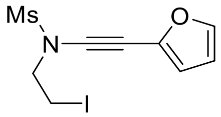

1o

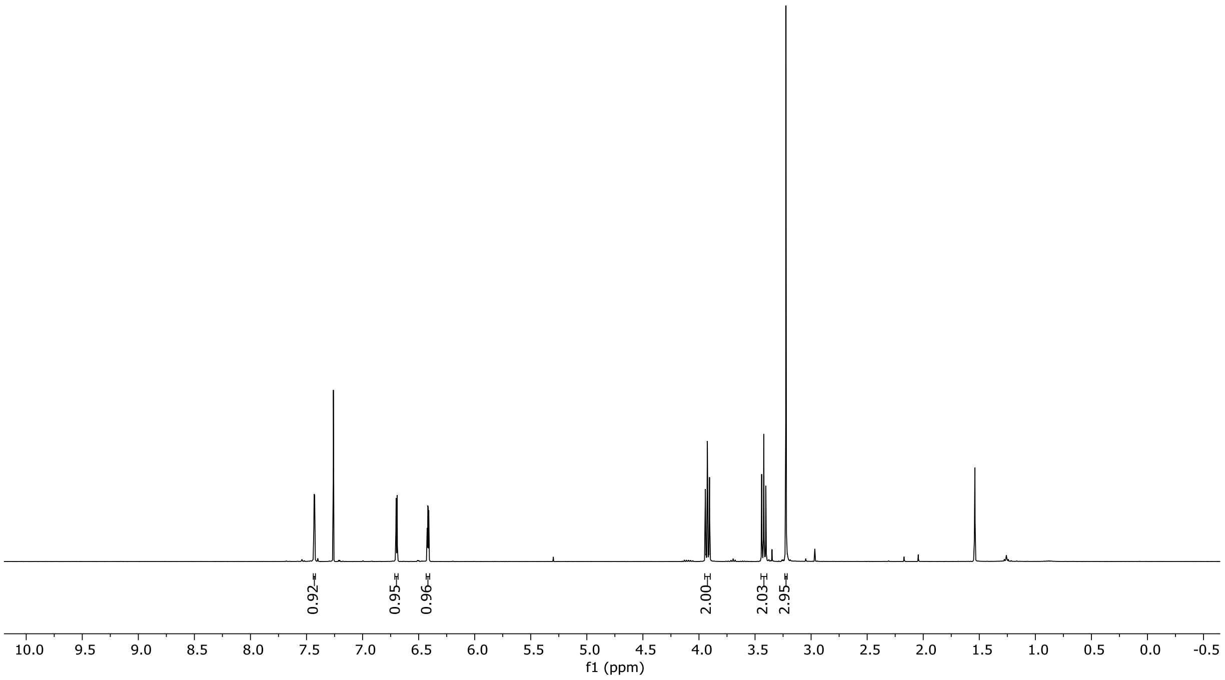

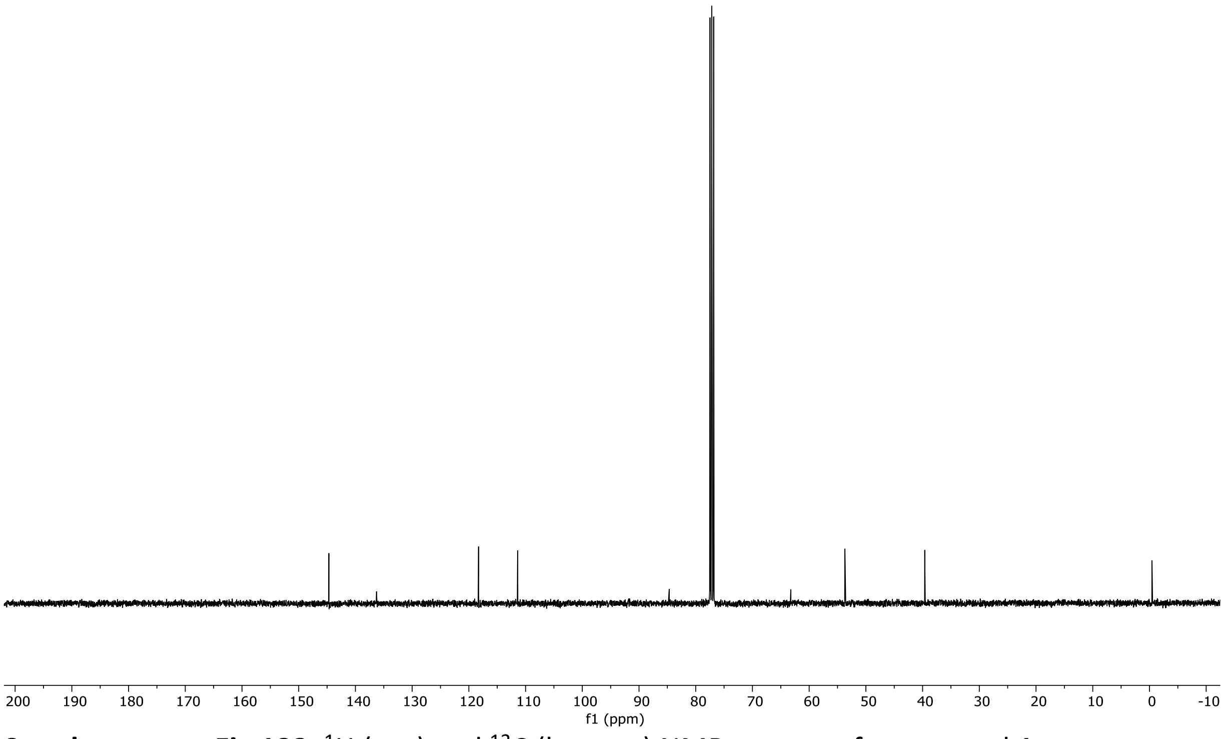

Supplementary Fig 123. ^{1}H (top) and ^{13}C (bottom) NMR spectra of compound **1o**.

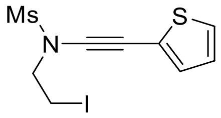

1p

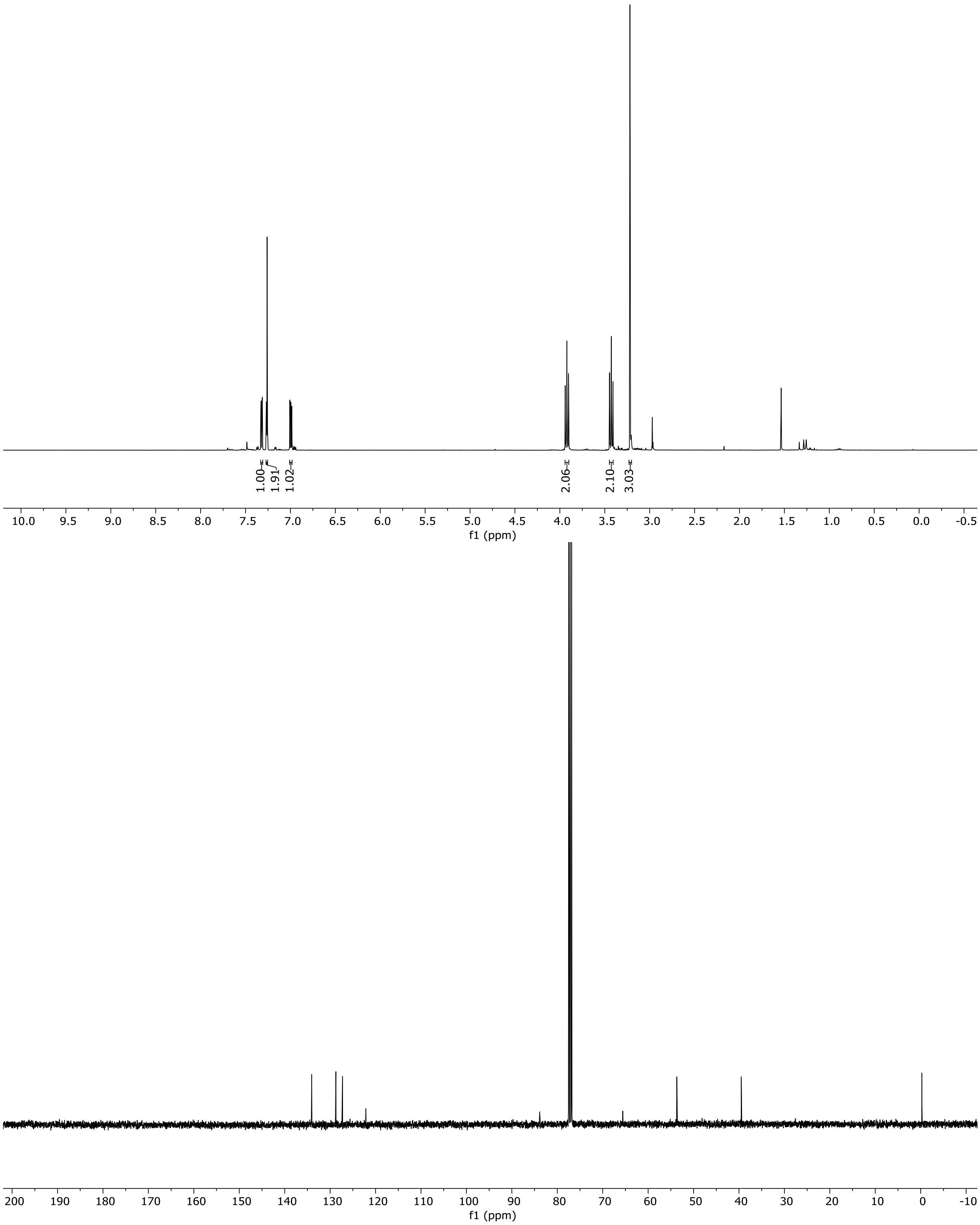

Supplementary Fig 124. ^{1}H (top) and ^{13}C (bottom) NMR spectra of compound **1p**.

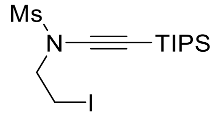

1q

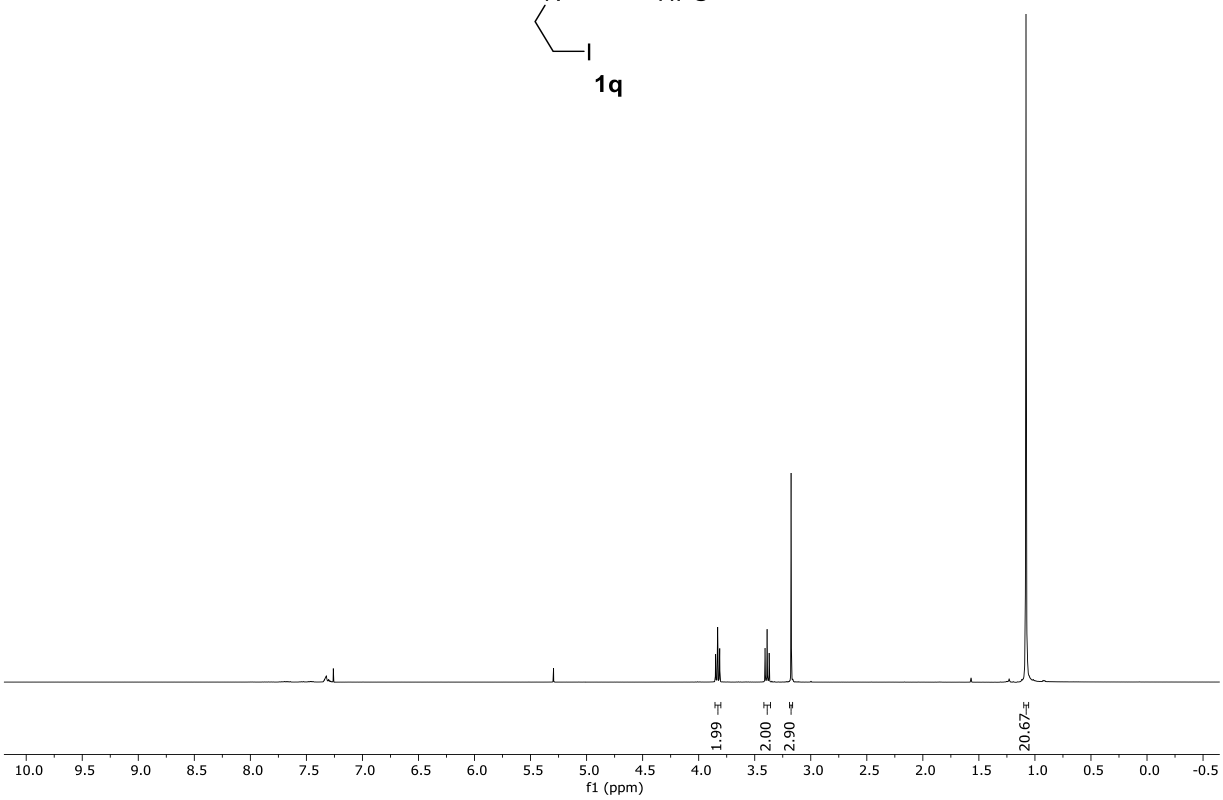

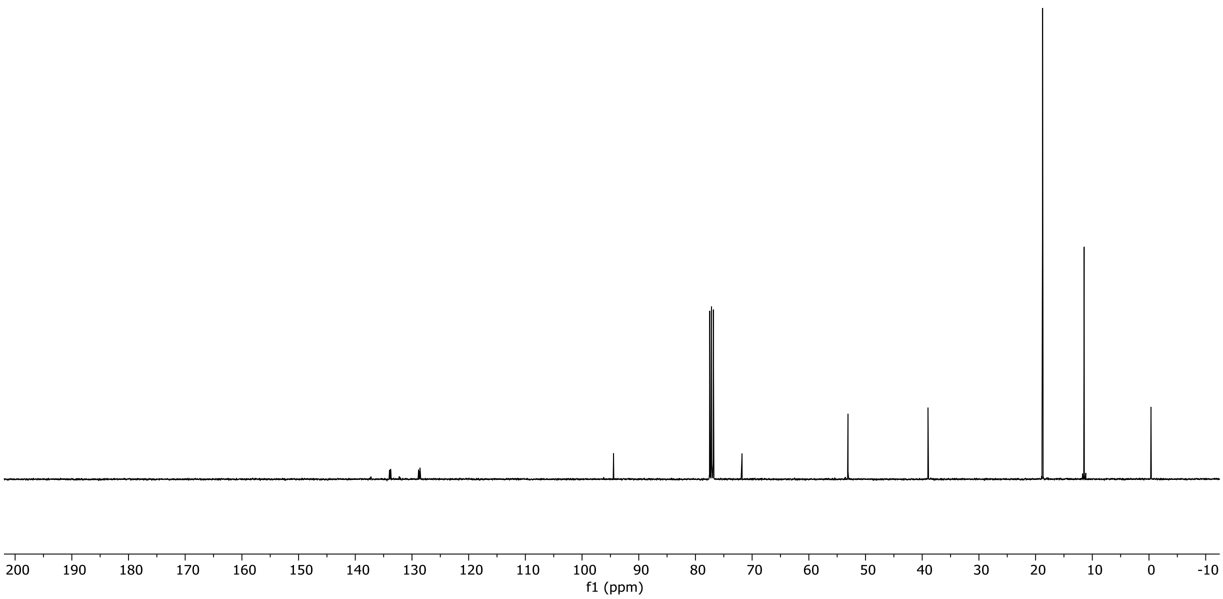

Supplementary Fig 125. ^{1}H (top) and ^{13}C (bottom) NMR spectra of compound **1q**.

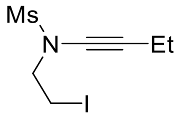

1r

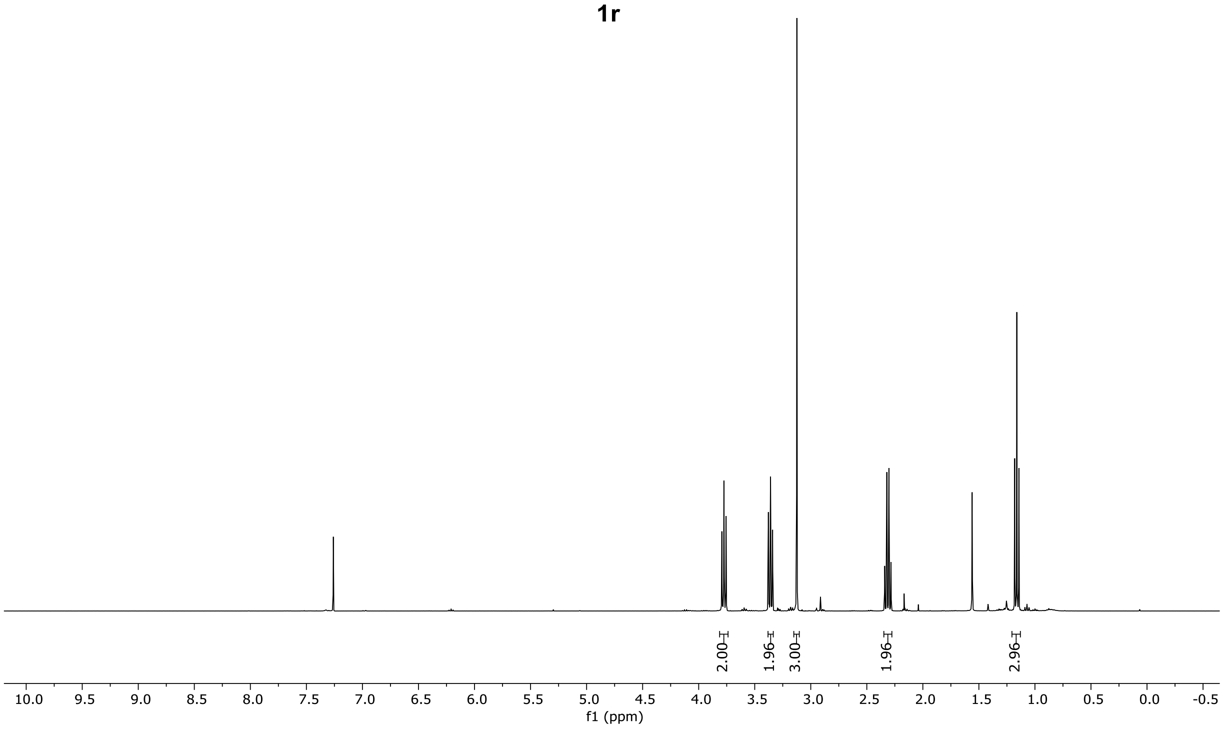

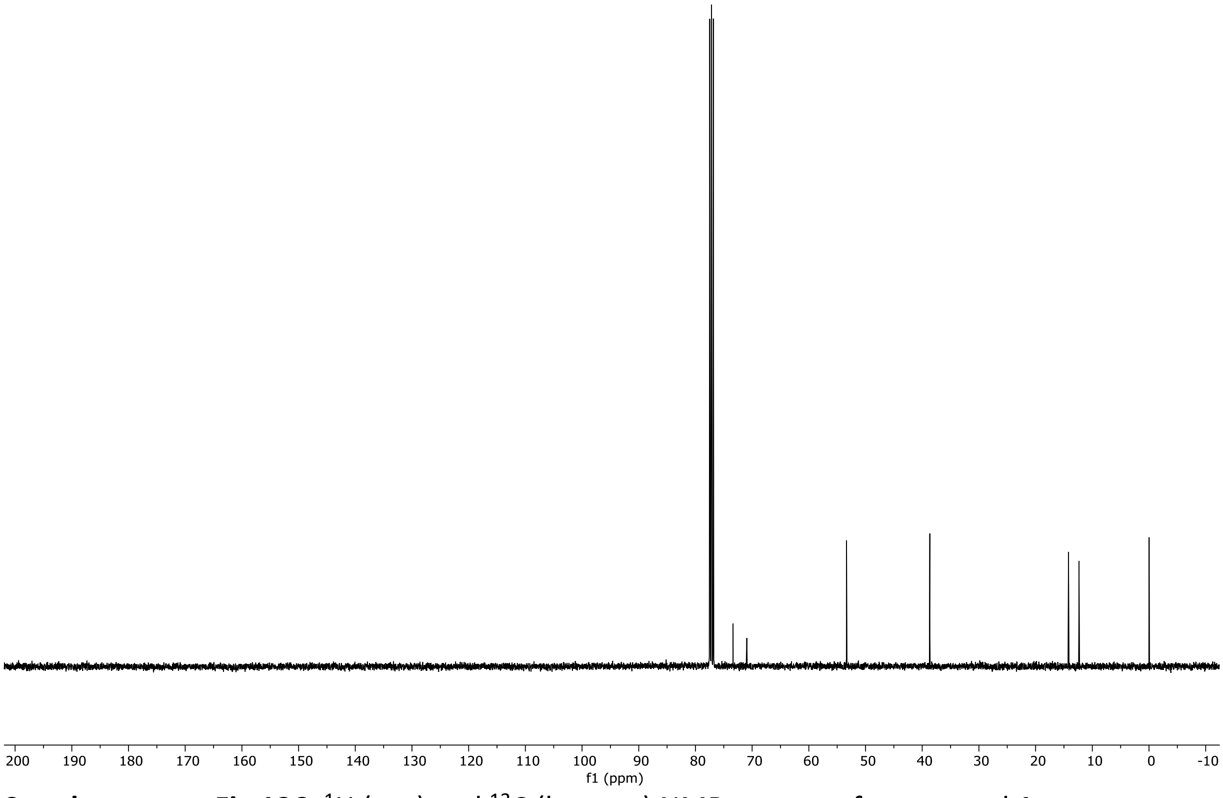

Supplementary Fig 126. ^{1}H (top) and ^{13}C (bottom) NMR spectra of compound **1r**.

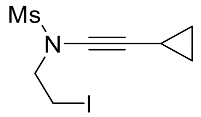

1s

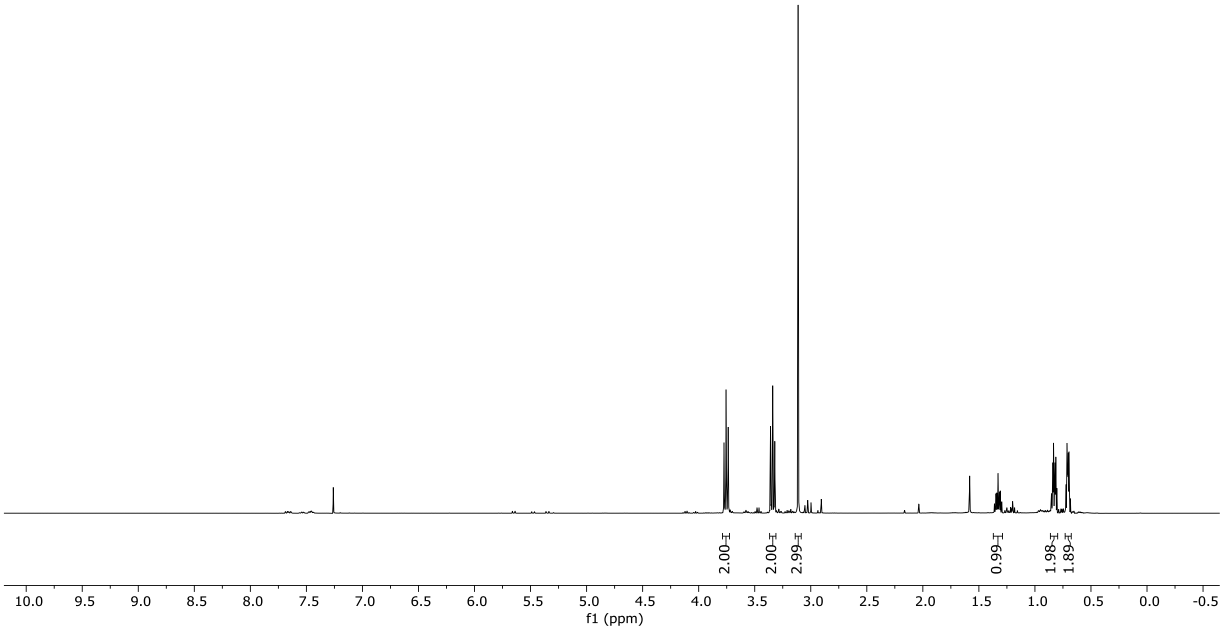

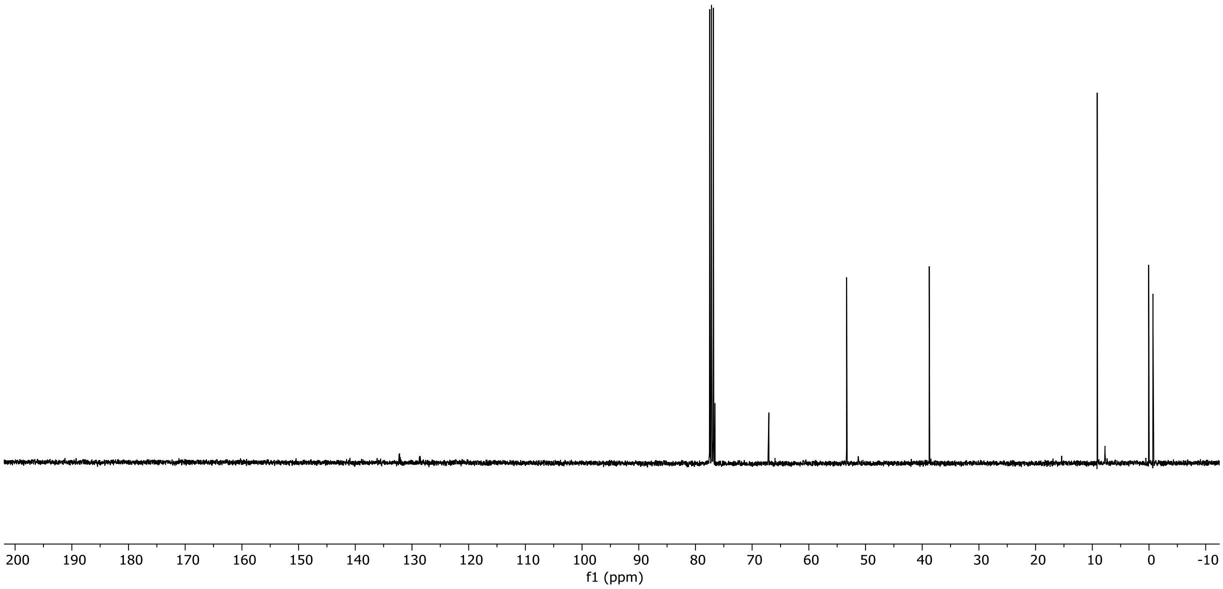

Supplementary Fig 127. ^{1}H (top) and ^{13}C (bottom) NMR spectra of compound **1s**.

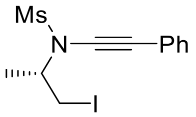

1t

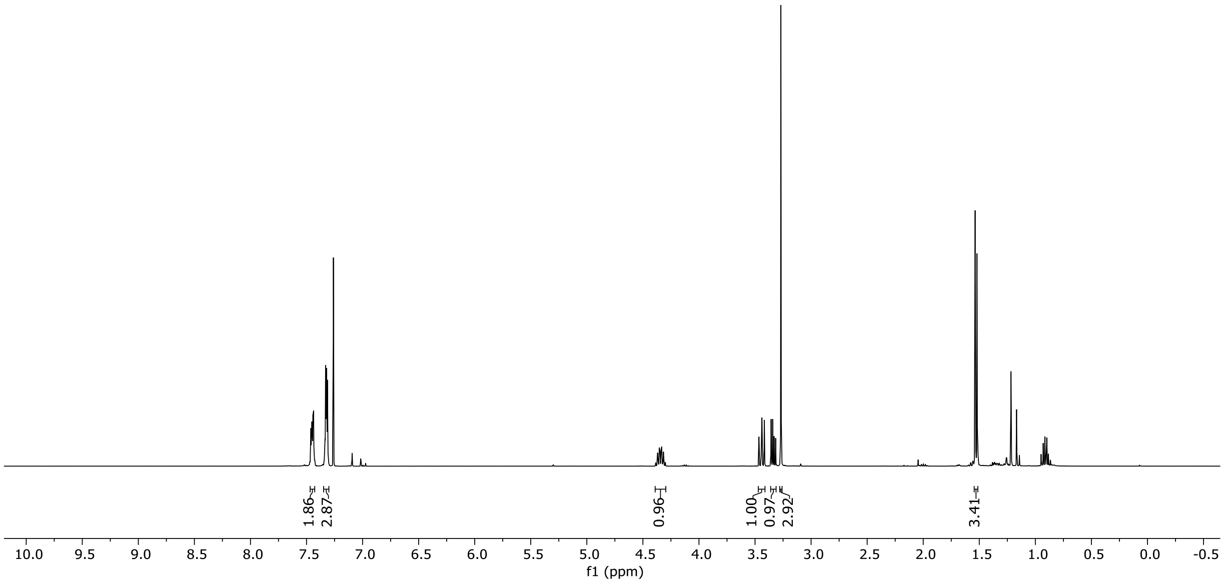

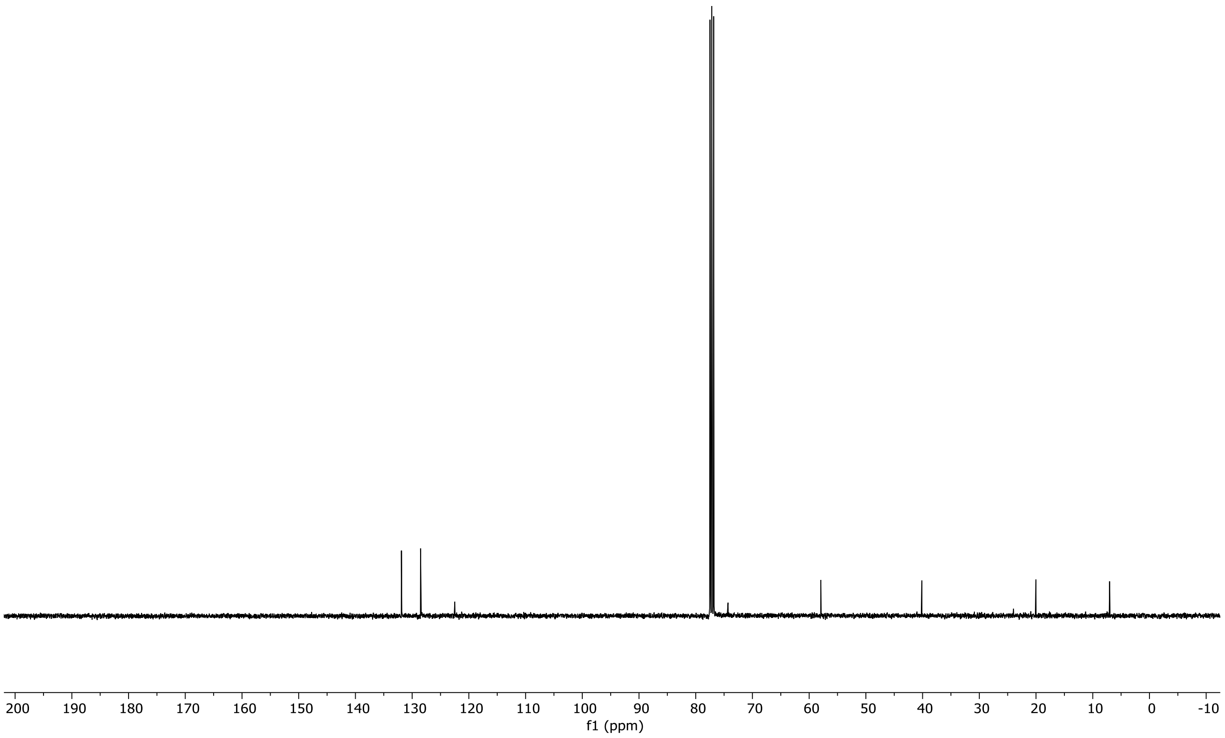

Supplementary Fig 128. ^{1}H (top) and ^{13}C (bottom) NMR spectra of compound **1t**.

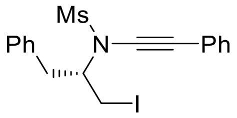

1u

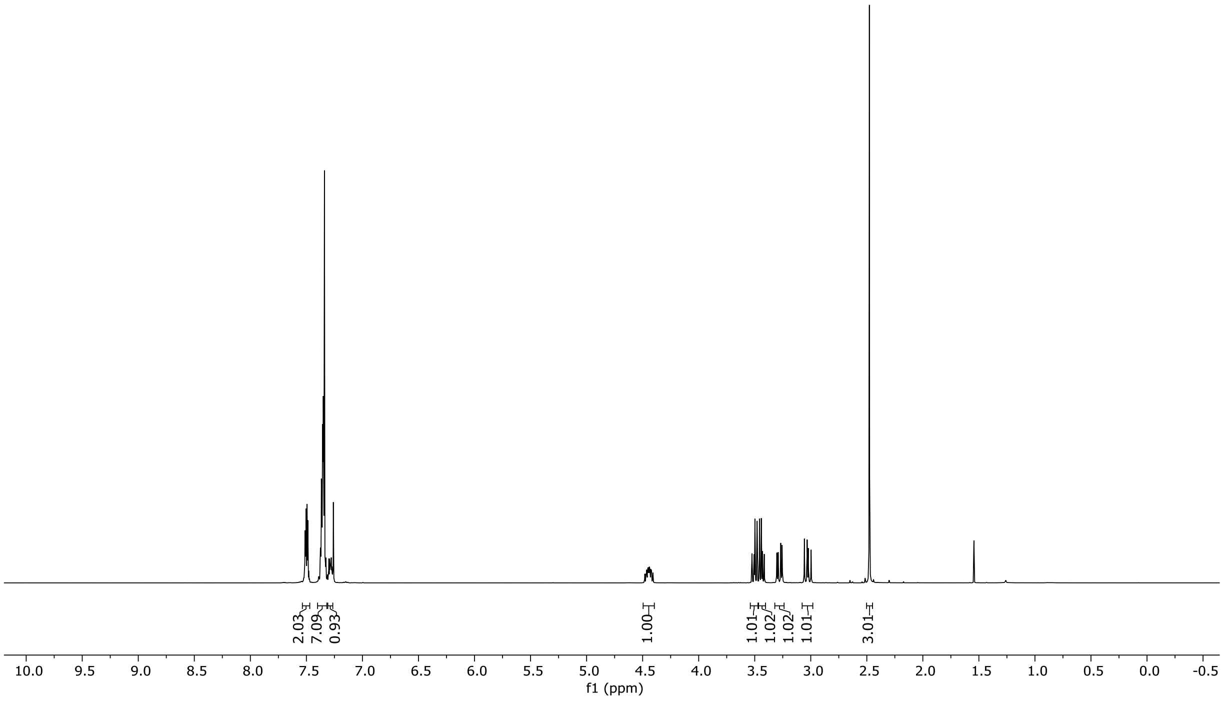

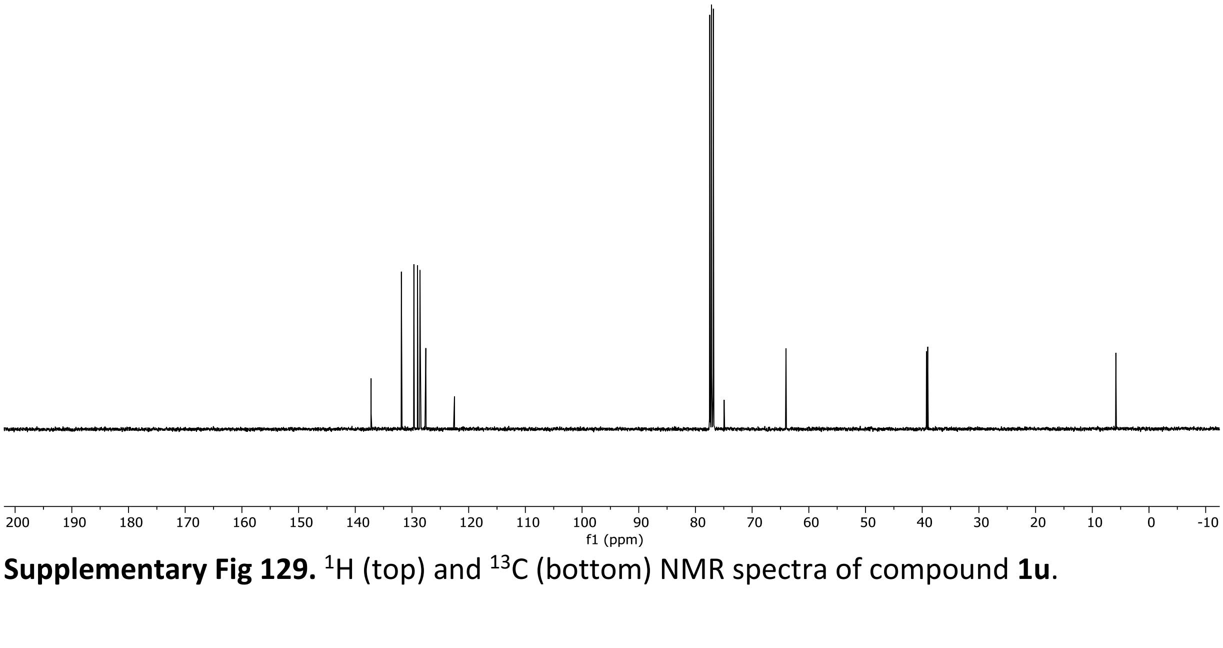

Supplementary Fig 129. ^{1}H (top) and ^{13}C (bottom) NMR spectra of compound **1u**.

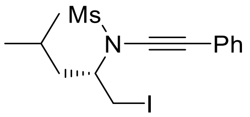

1v

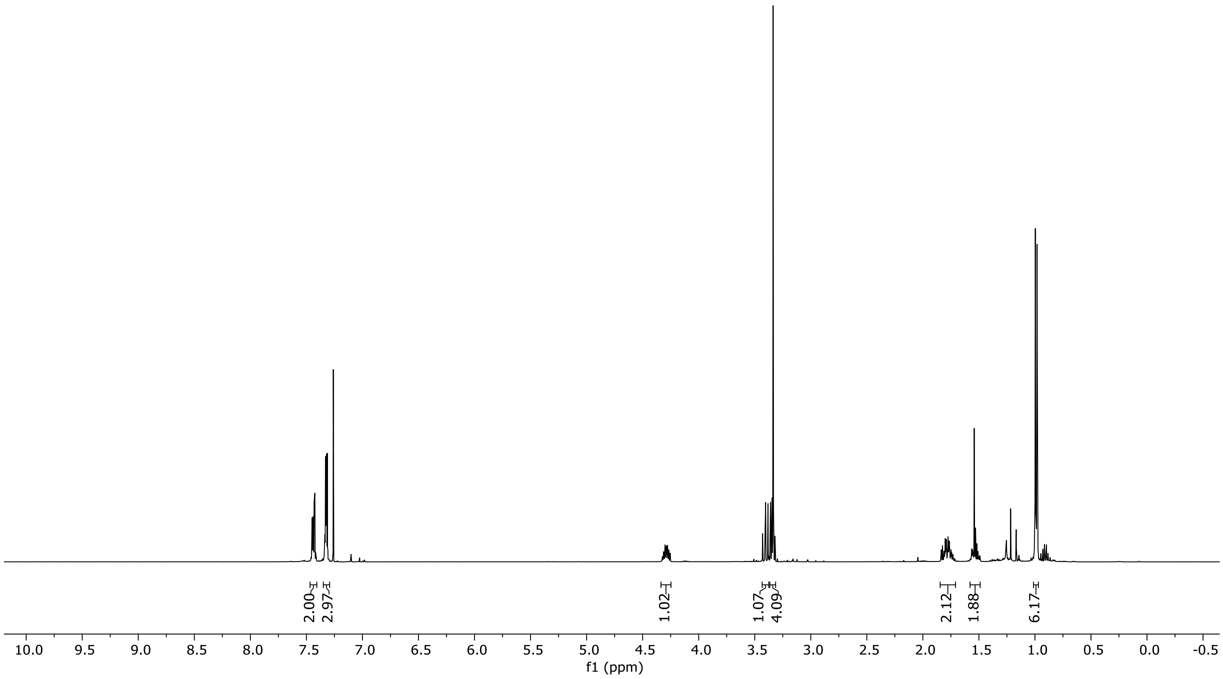

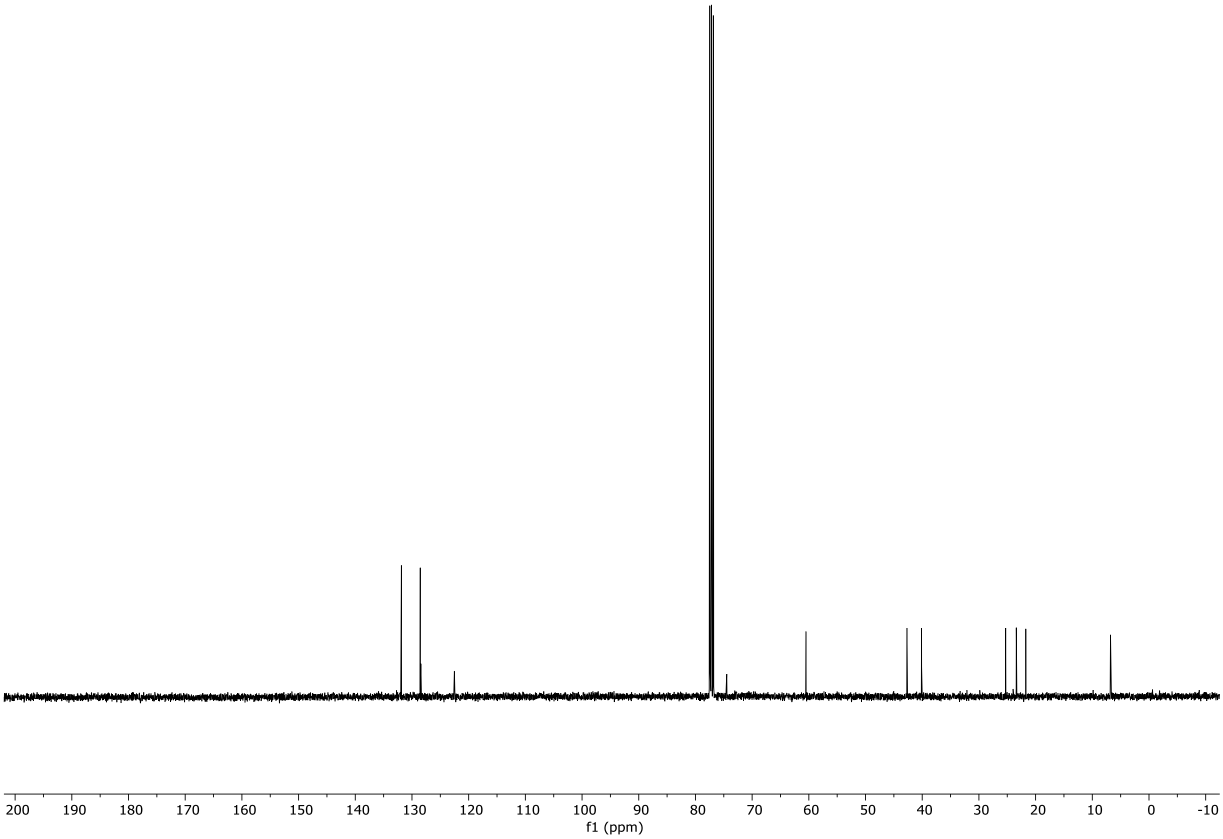

Supplementary Fig 130. ^{1}H (top) and ^{13}C (bottom) NMR spectra of compound **1v**.

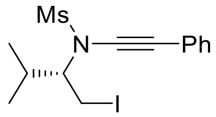

1w

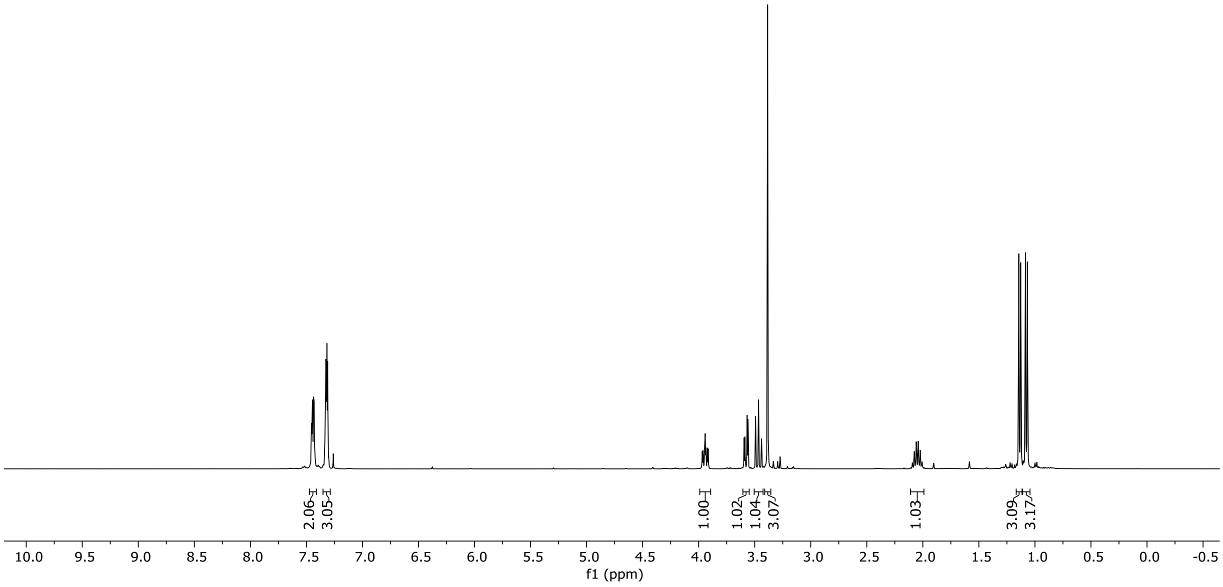

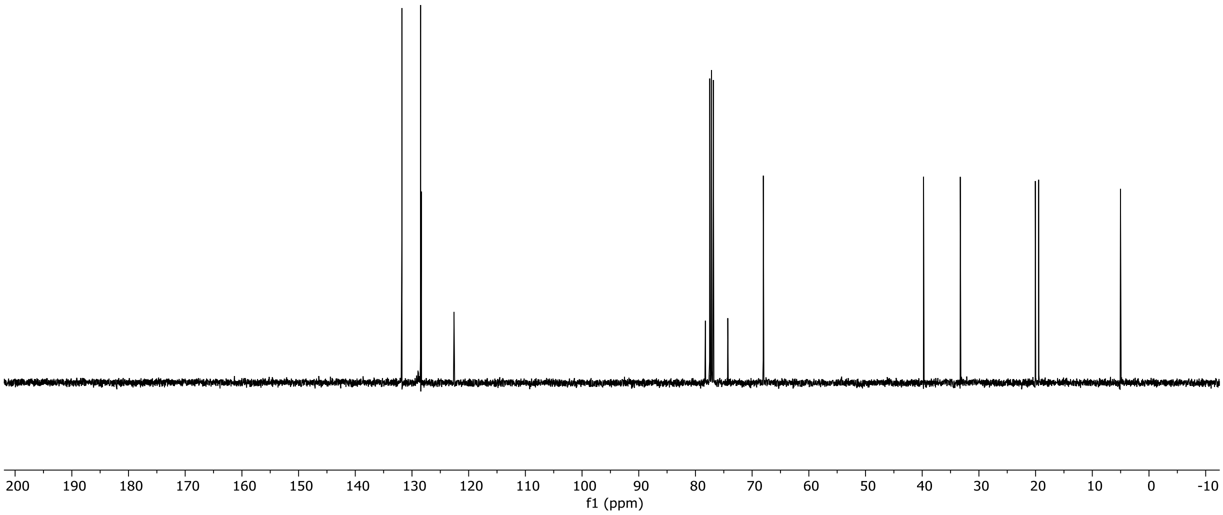

Supplementary Fig 131. ^{1}H (top) and ^{13}C (bottom) NMR spectra of compound **1w**.

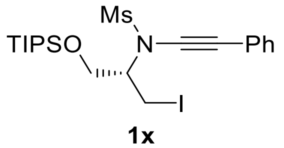

1x

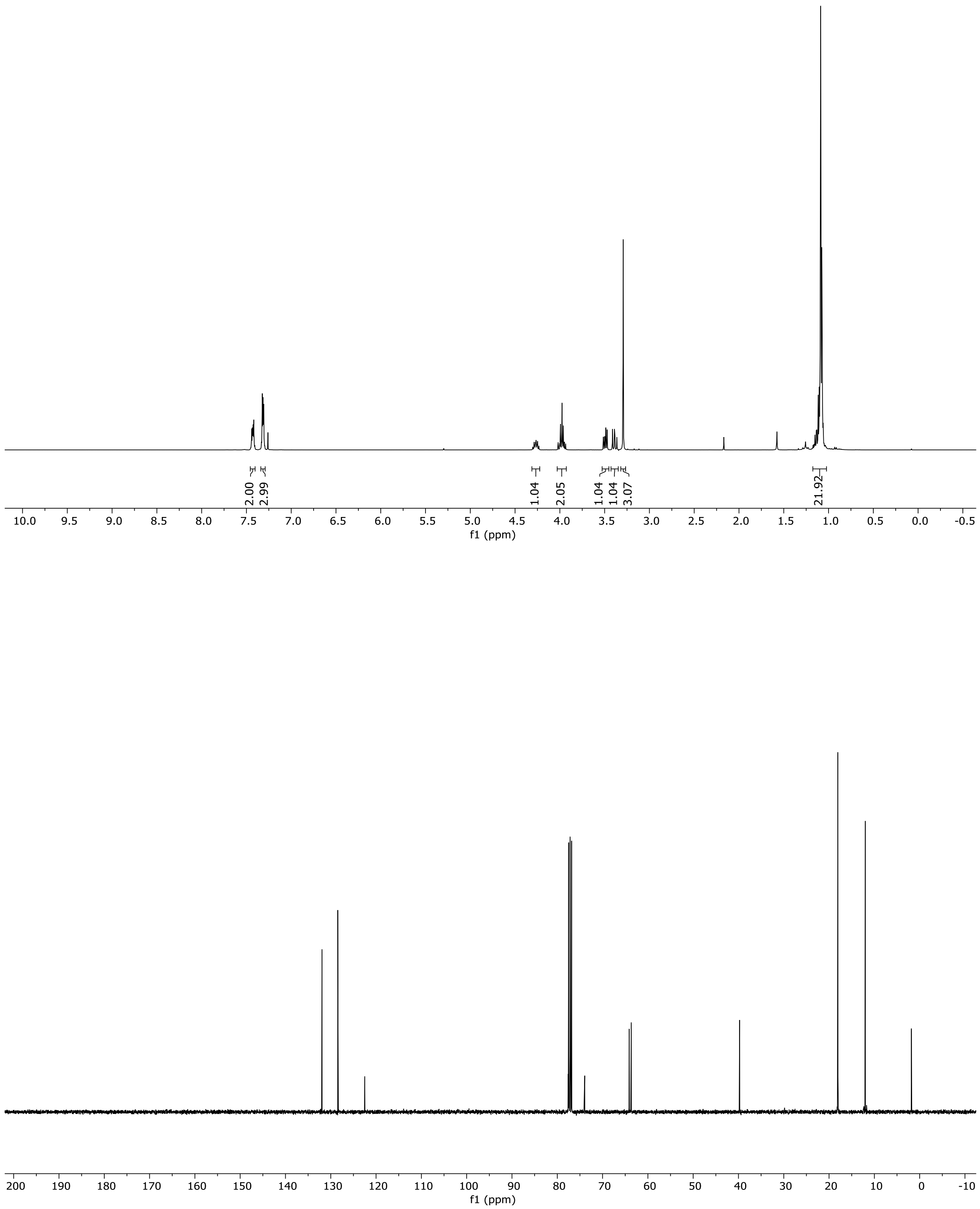

Supplementary Fig 132. ^{1}H (top) and ^{13}C (bottom) NMR spectra of compound **1x**.

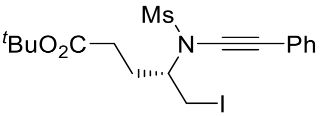

1y

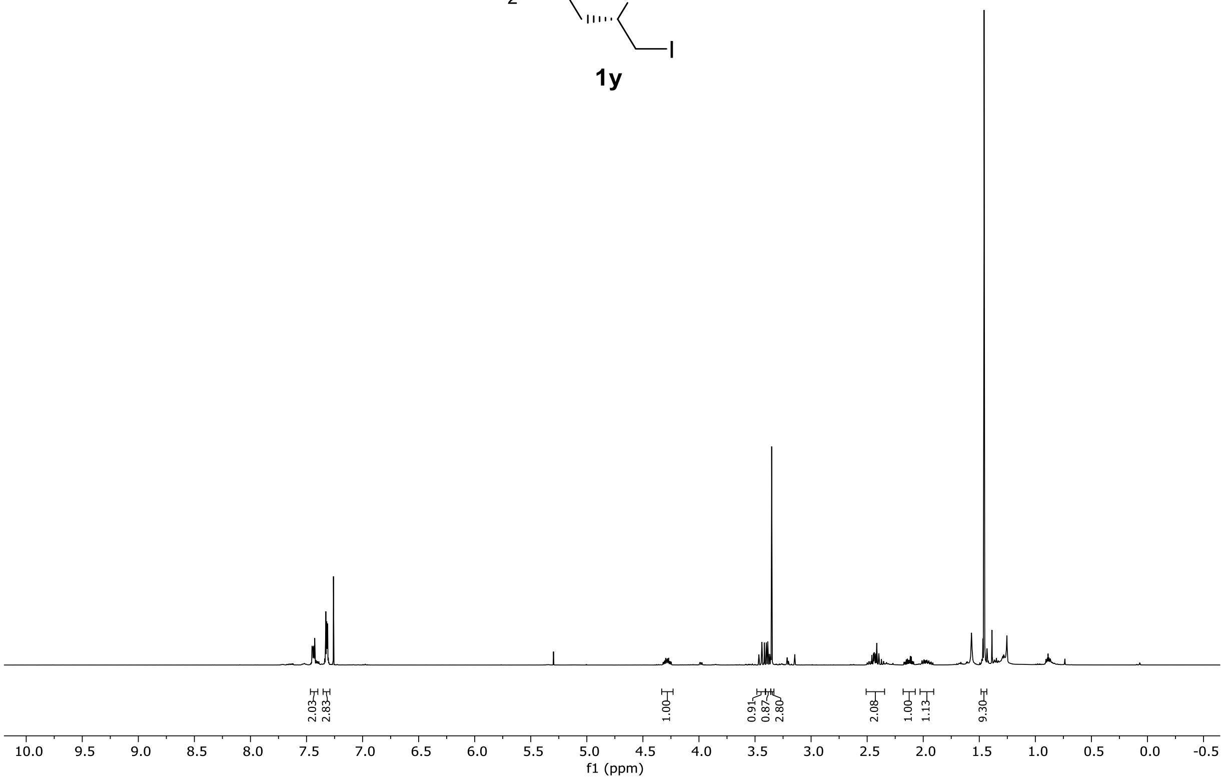

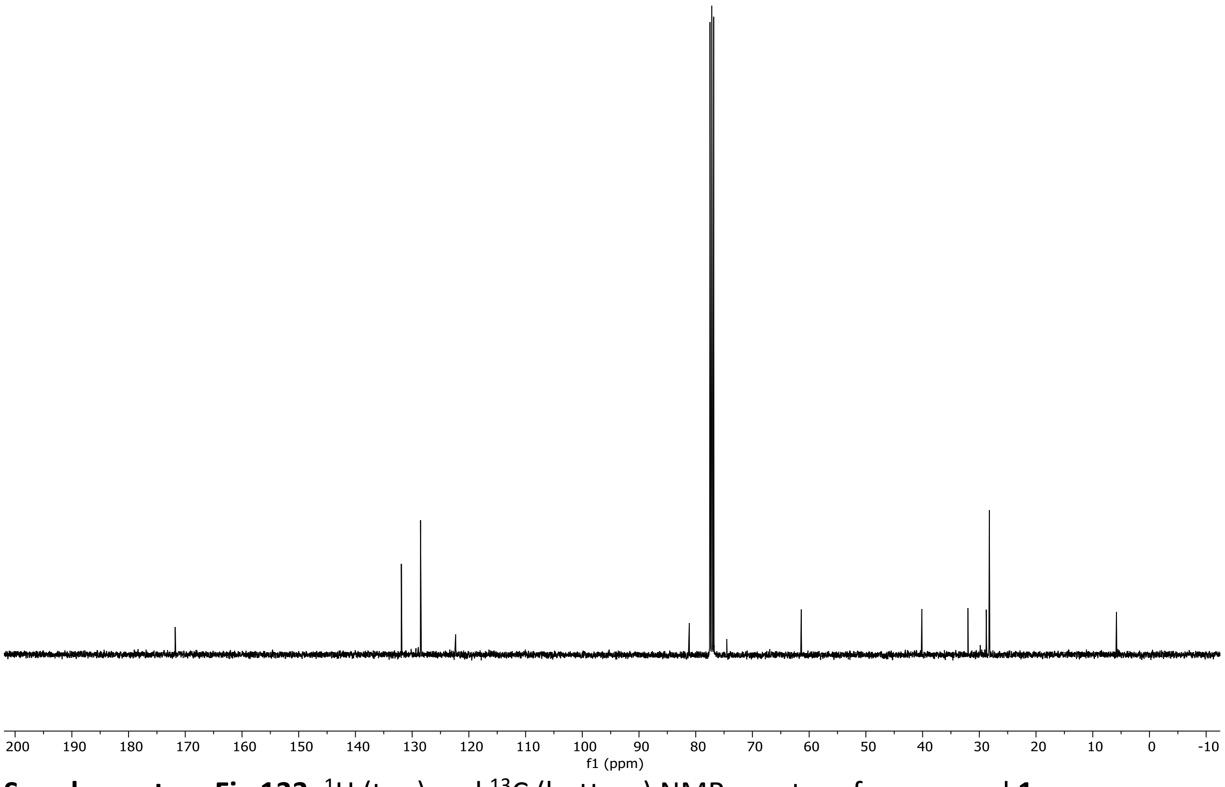

Supplementary Fig 133. ^{1}H (top) and ^{13}C (bottom) NMR spectra of compound **1y**.

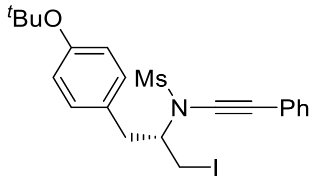

1z

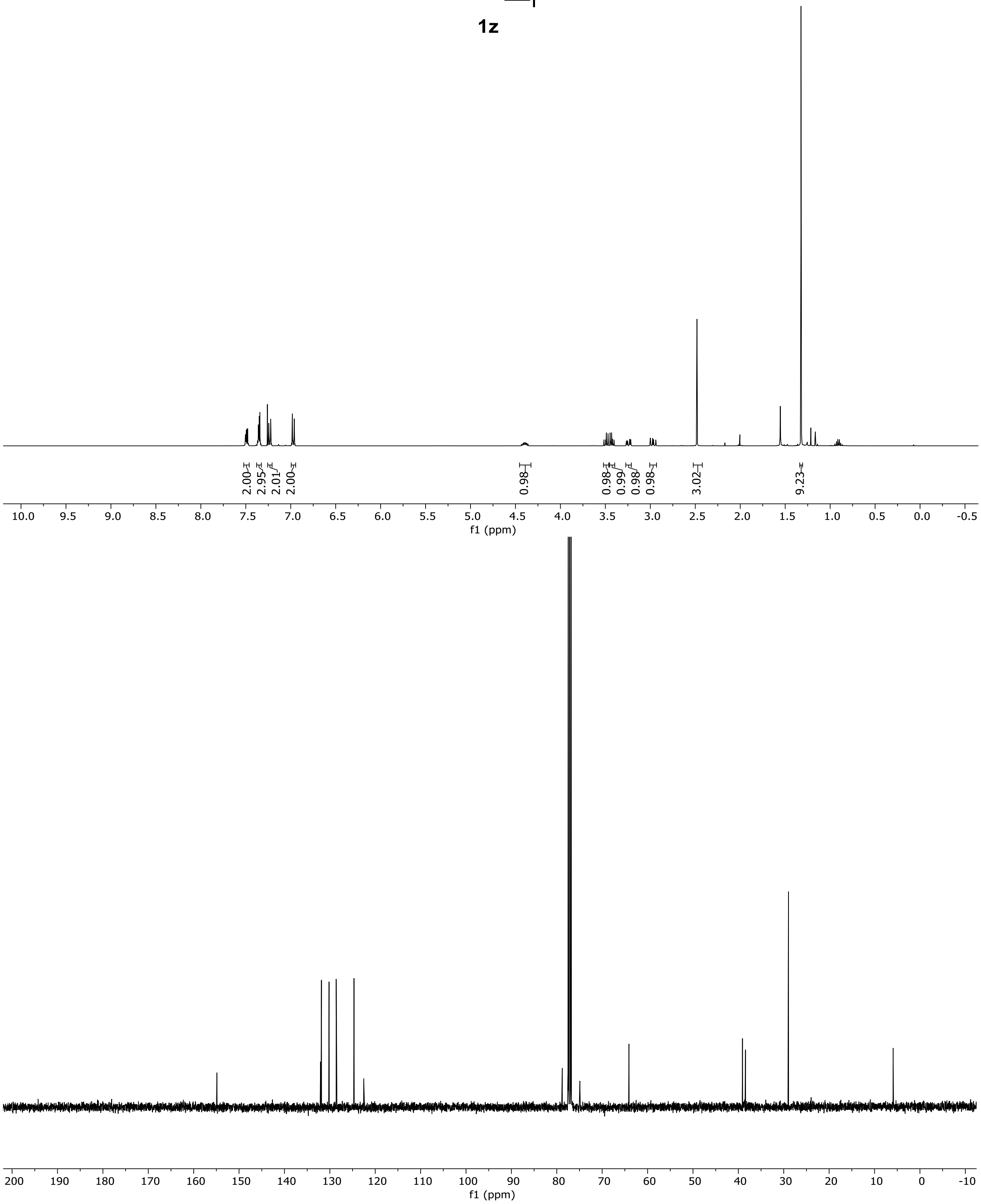

Supplementary Fig 134. ^{1}H (top) and ^{13}C (bottom) NMR spectra of compound **1z**.

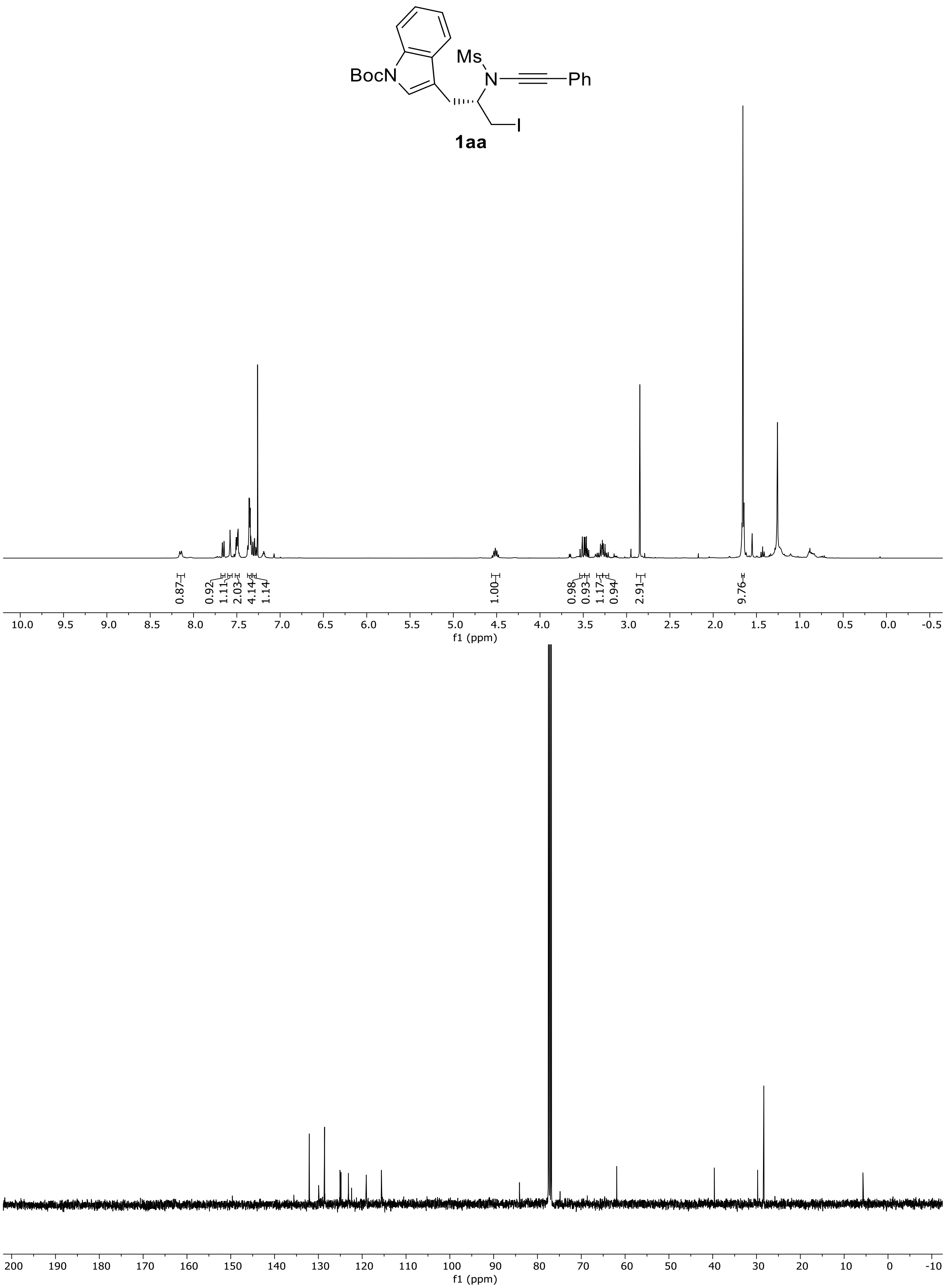

Supplementary Fig 135. ^{1}H (top) and ^{13}C (bottom) NMR spectra of compound **1aa**.

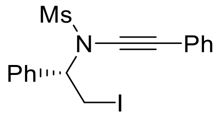

1ab

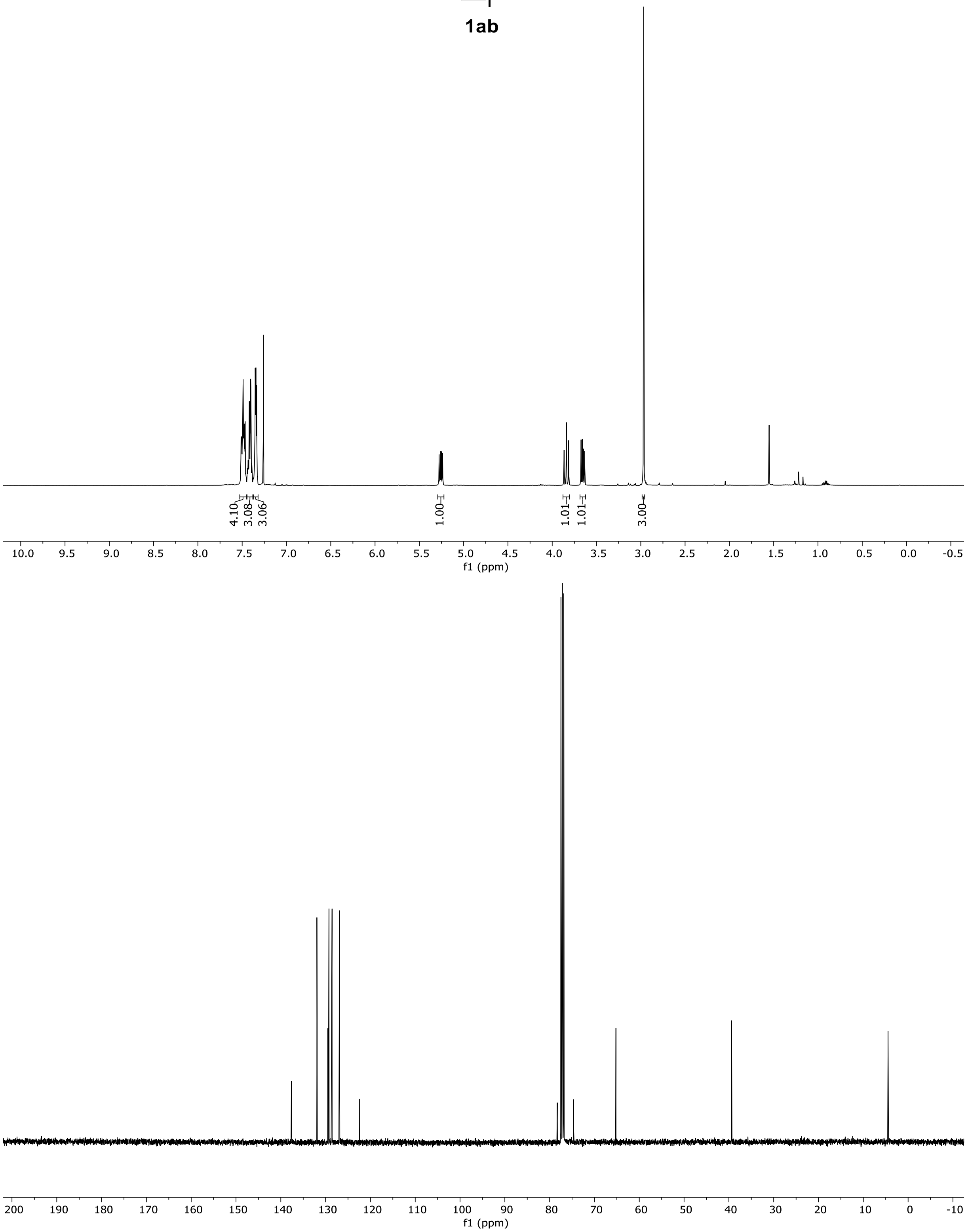

Supplementary Fig 136. ^{1}H (top) and ^{13}C (bottom) NMR spectra of compound **1ab**.

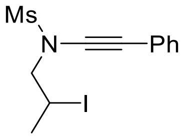

1ac

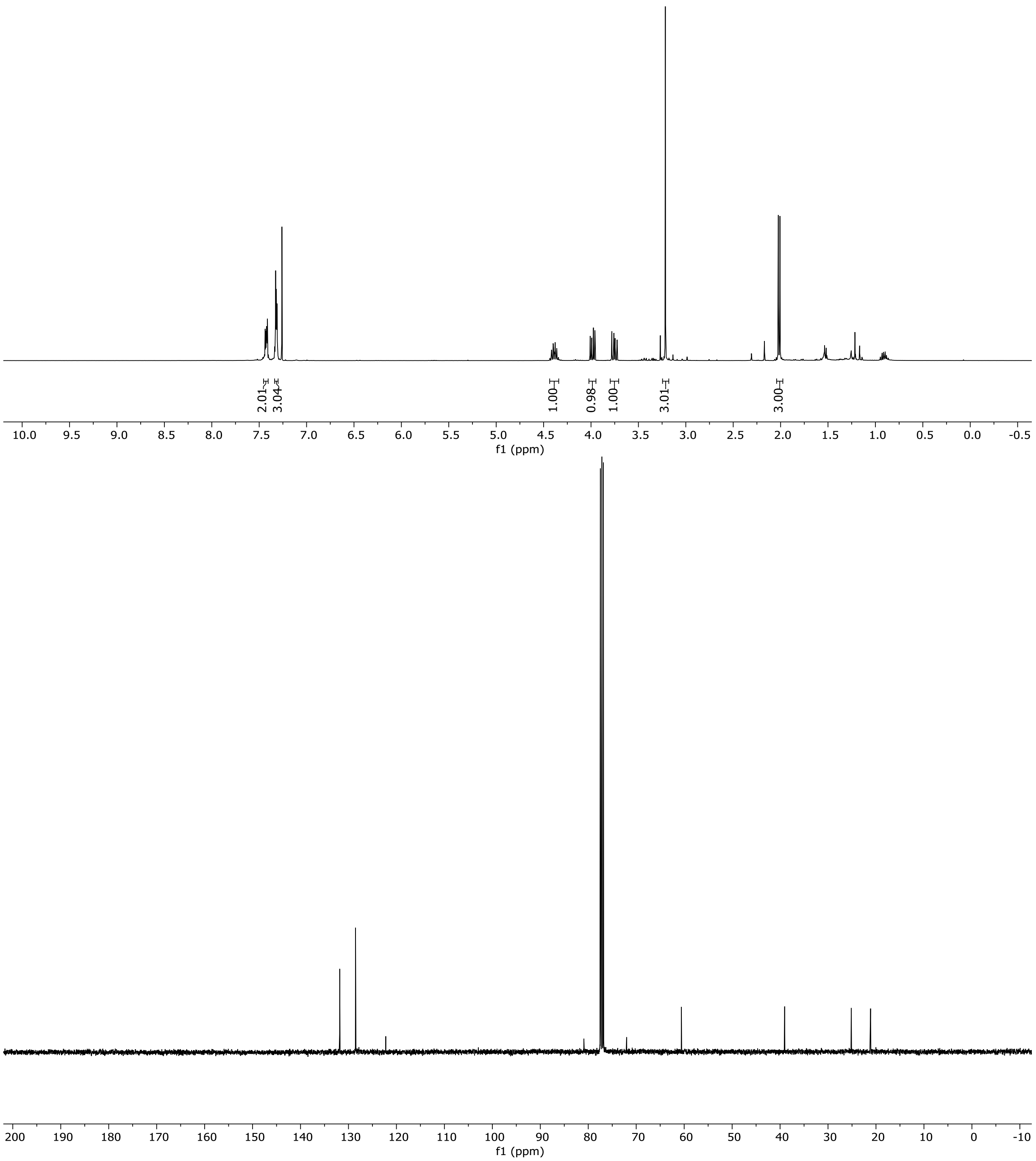

Supplementary Fig 137. ^{1}H (top) and ^{13}C (bottom) NMR spectra of compound **1ac**.

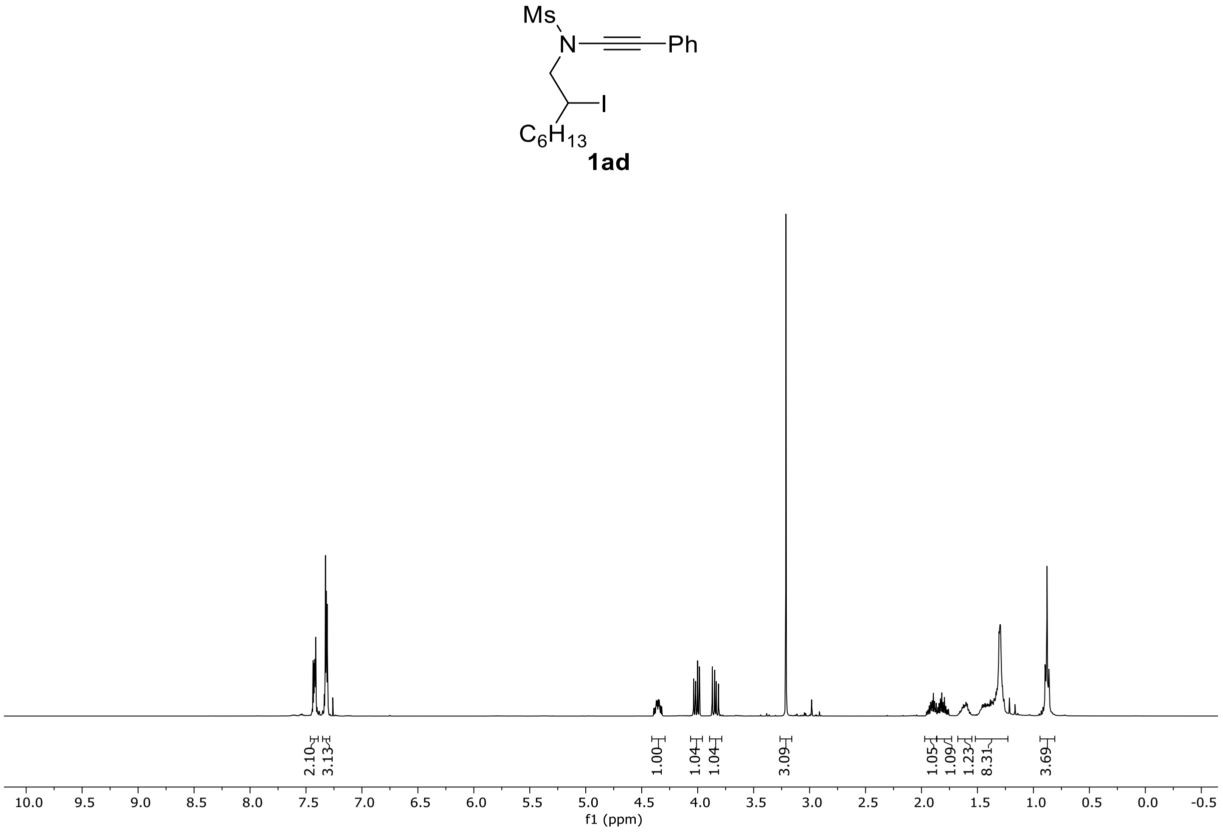

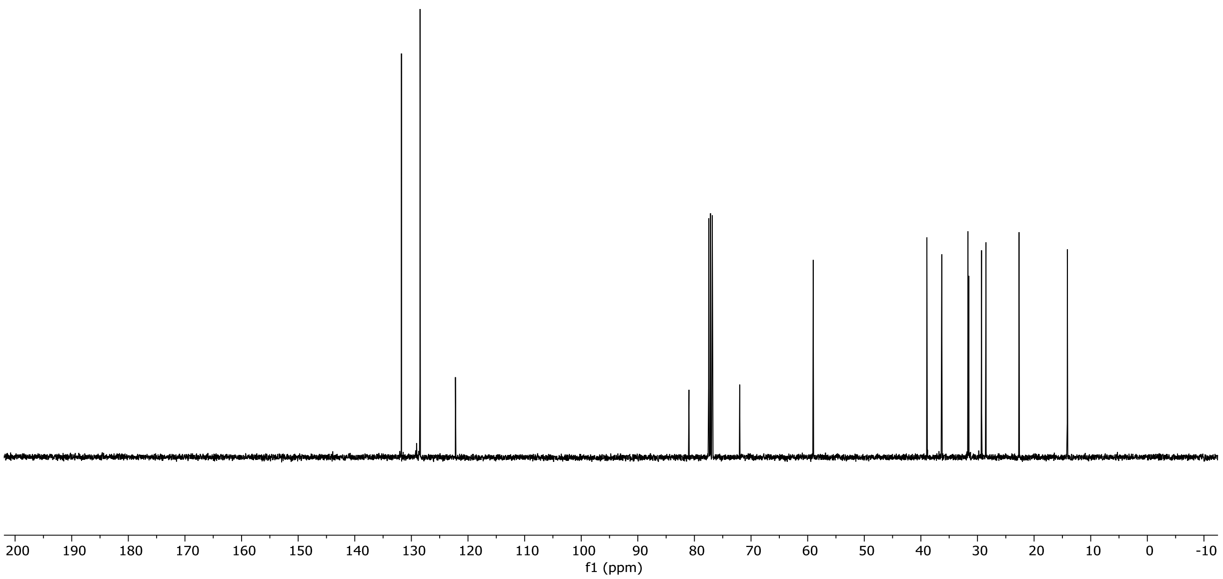

Supplementary Fig 138. ^{1}H (top) and ^{13}C (bottom) NMR spectra of compound **1ad**.

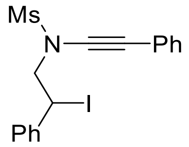

1ae

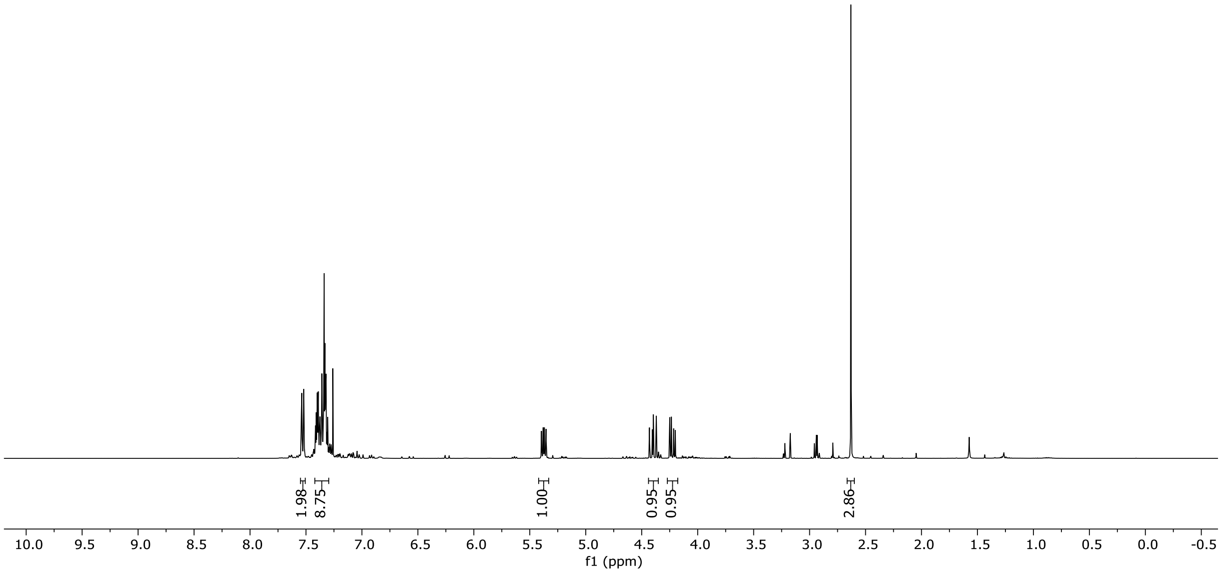

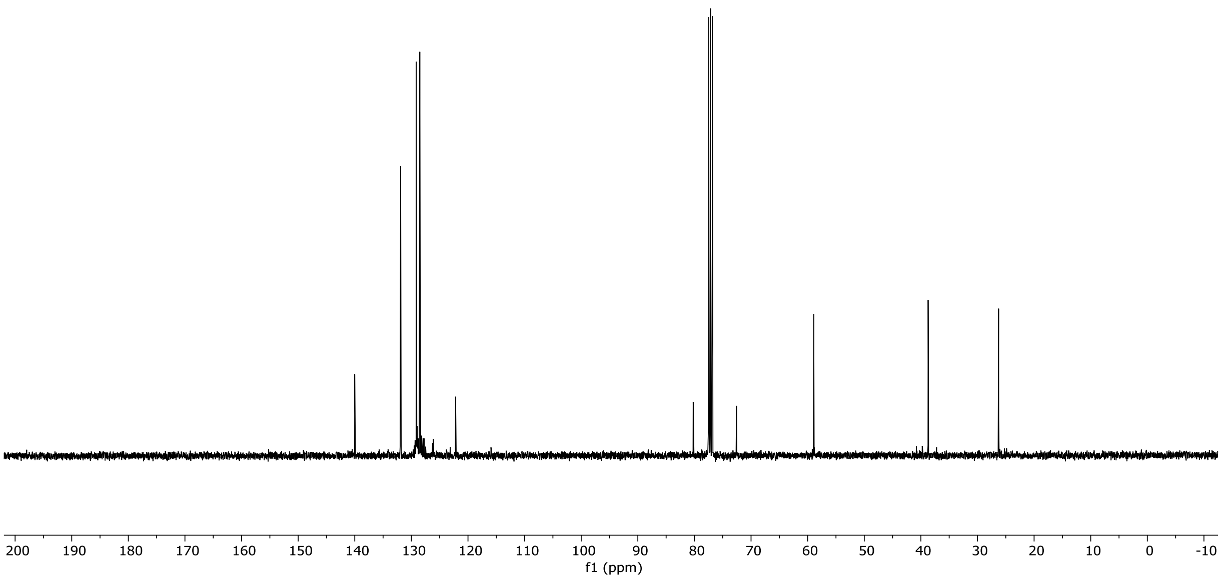

Supplementary Fig 139. ^{1}H (top) and ^{13}C (bottom) NMR spectra of compound **1ae**.

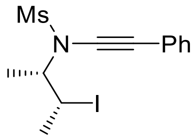

1af

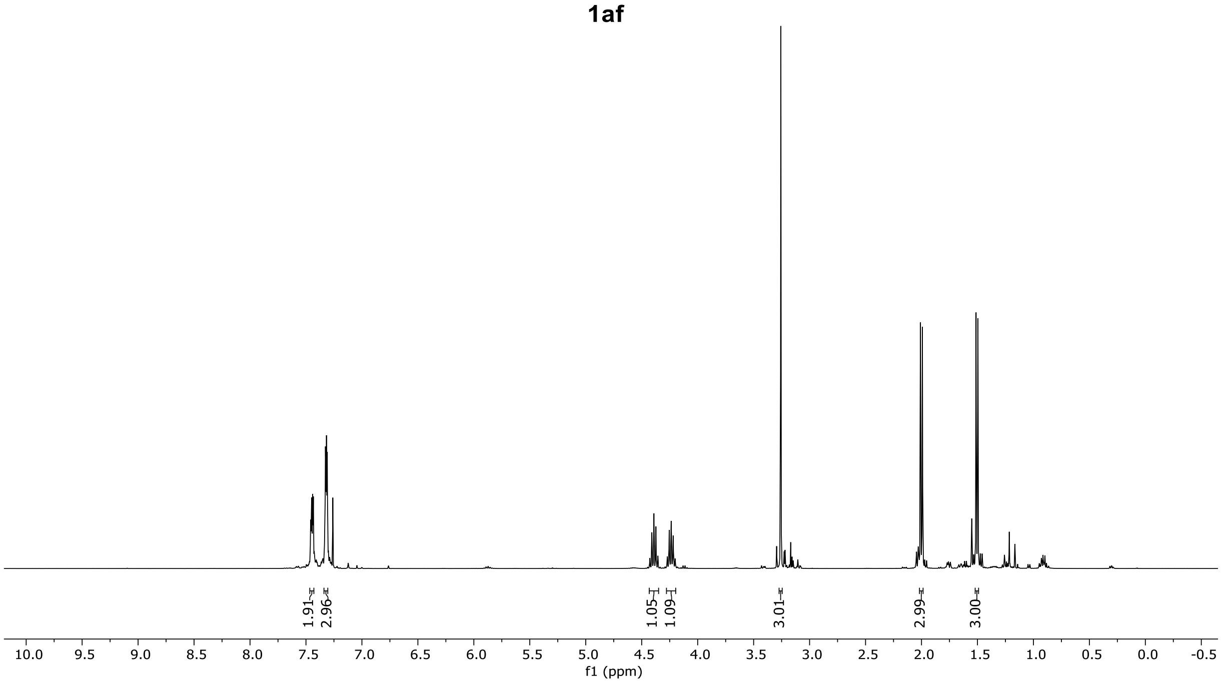

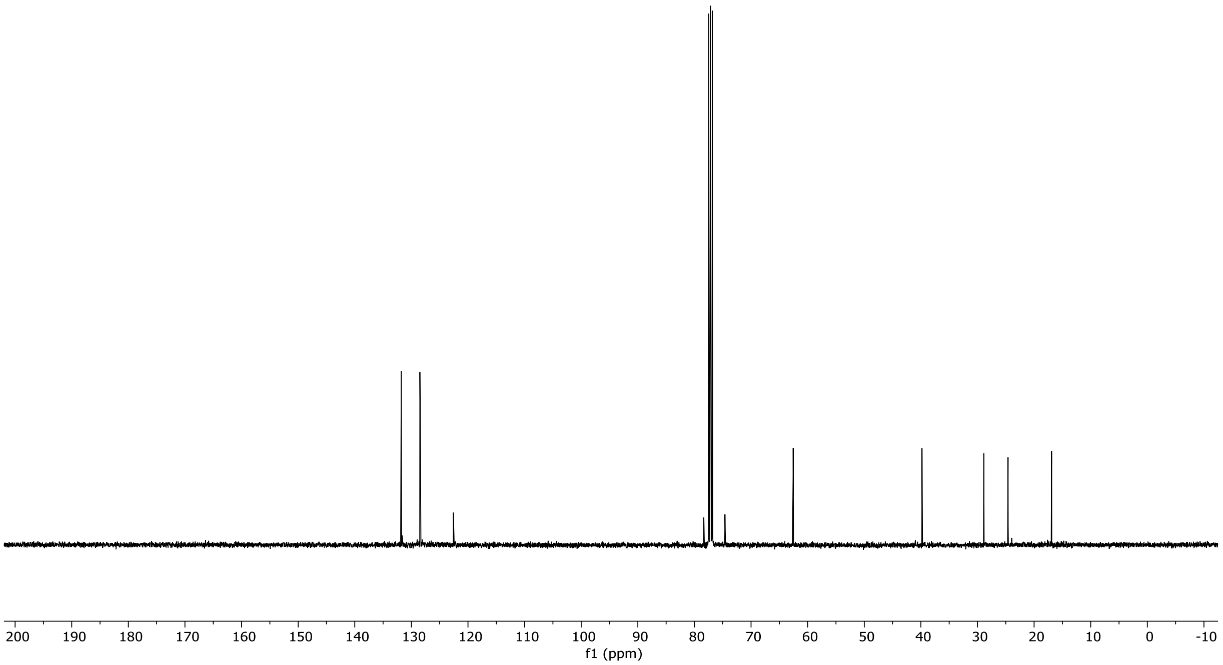

Supplementary Fig 140. ^{1}H (top) and ^{13}C (bottom) NMR spectra of compound **1af**.

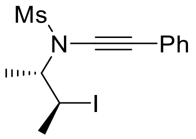

1af'

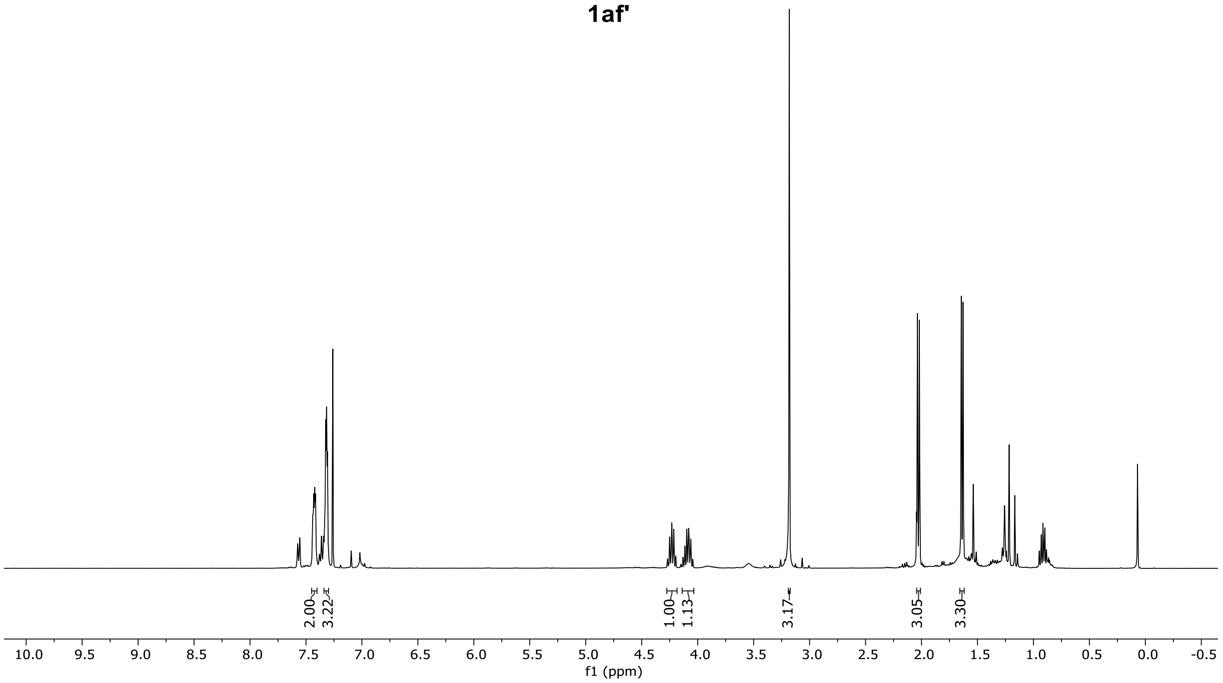

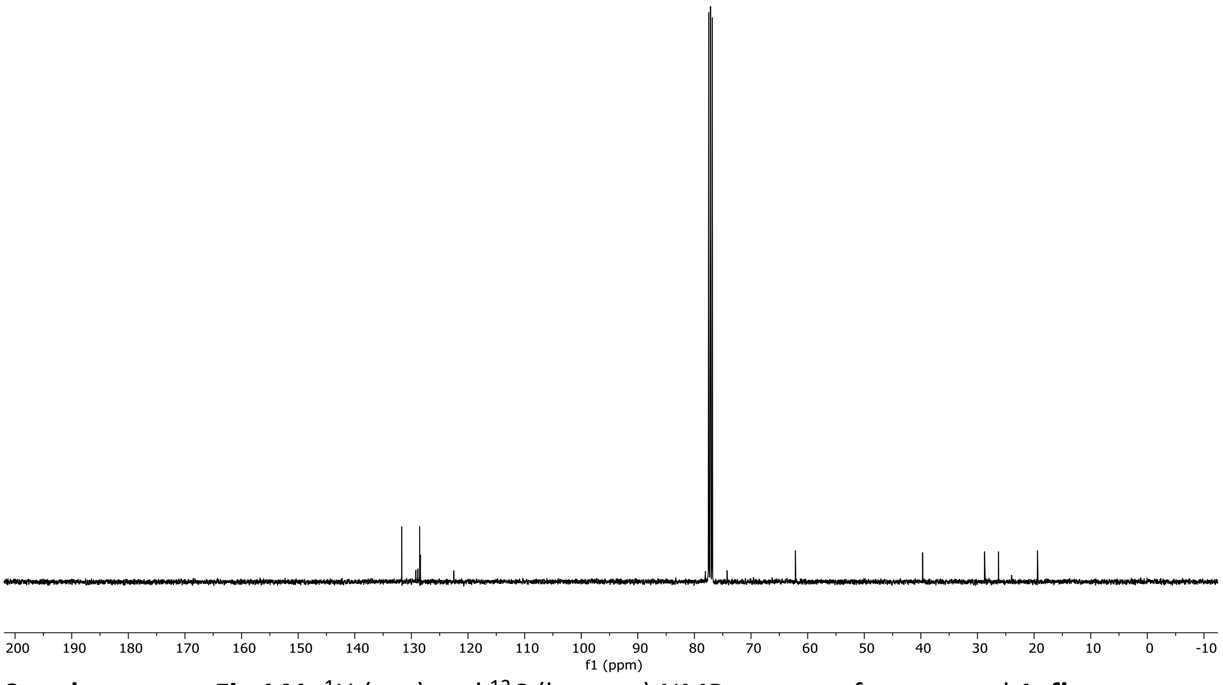

Supplementary Fig 141. ^{1}H (top) and ^{13}C (bottom) NMR spectra of compound **1af’**.

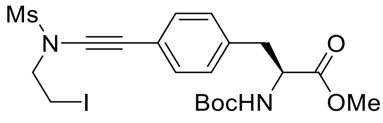

1ag

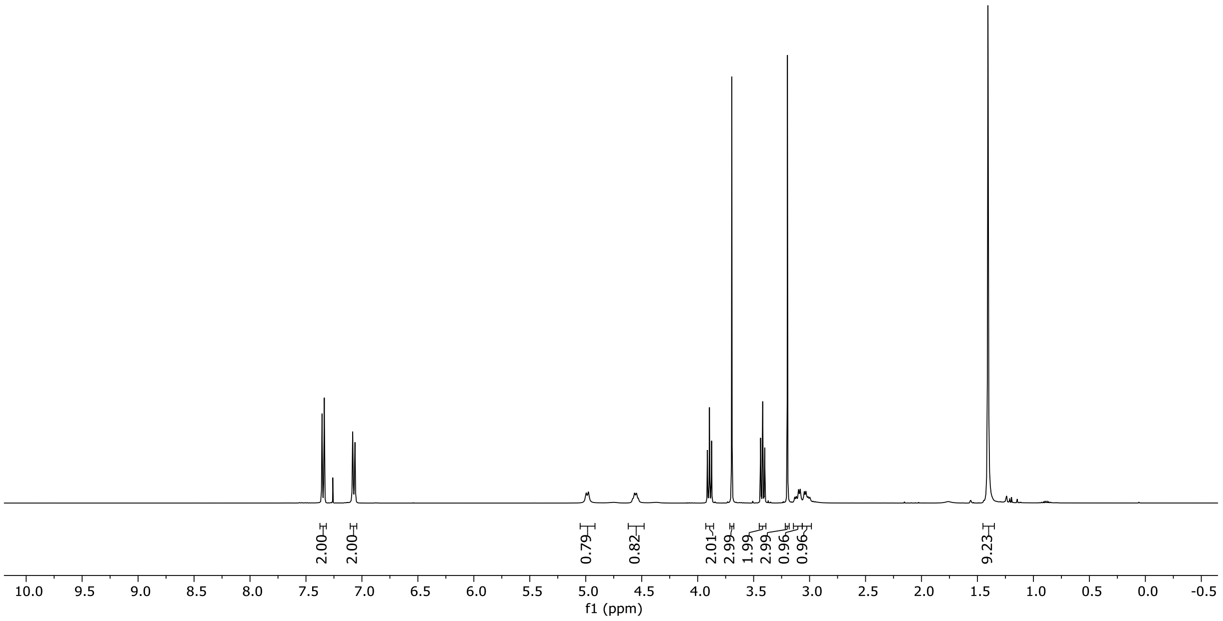

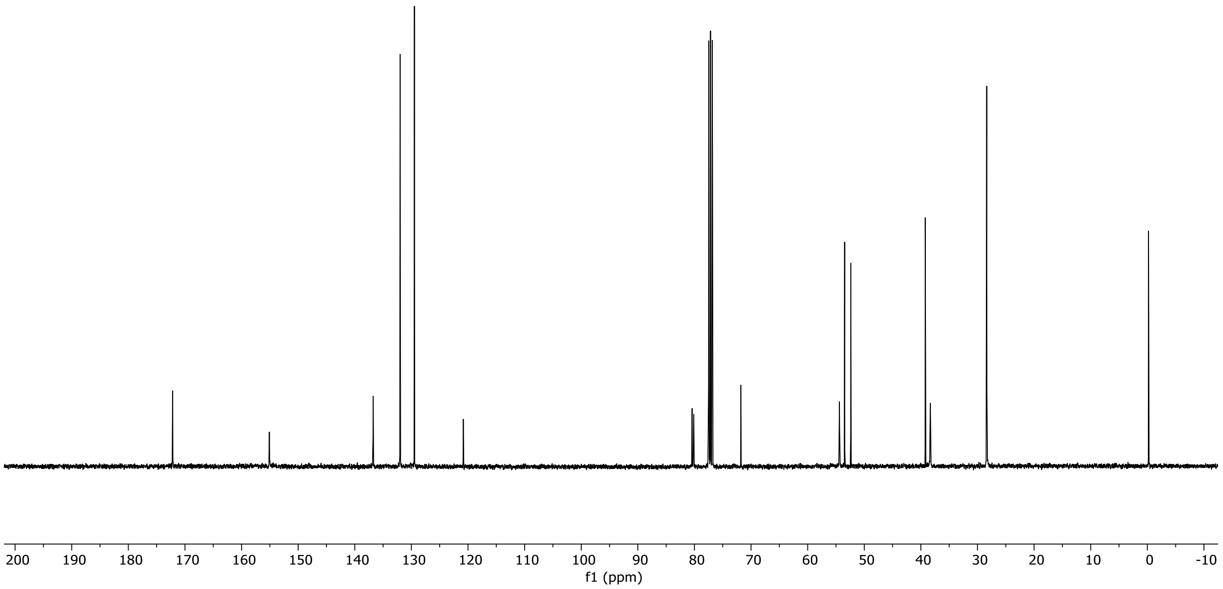

Supplementary Fig 142. ^{1}H (top) and ^{13}C (bottom) NMR spectra of compound **1ag**.

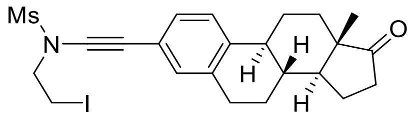

1ah

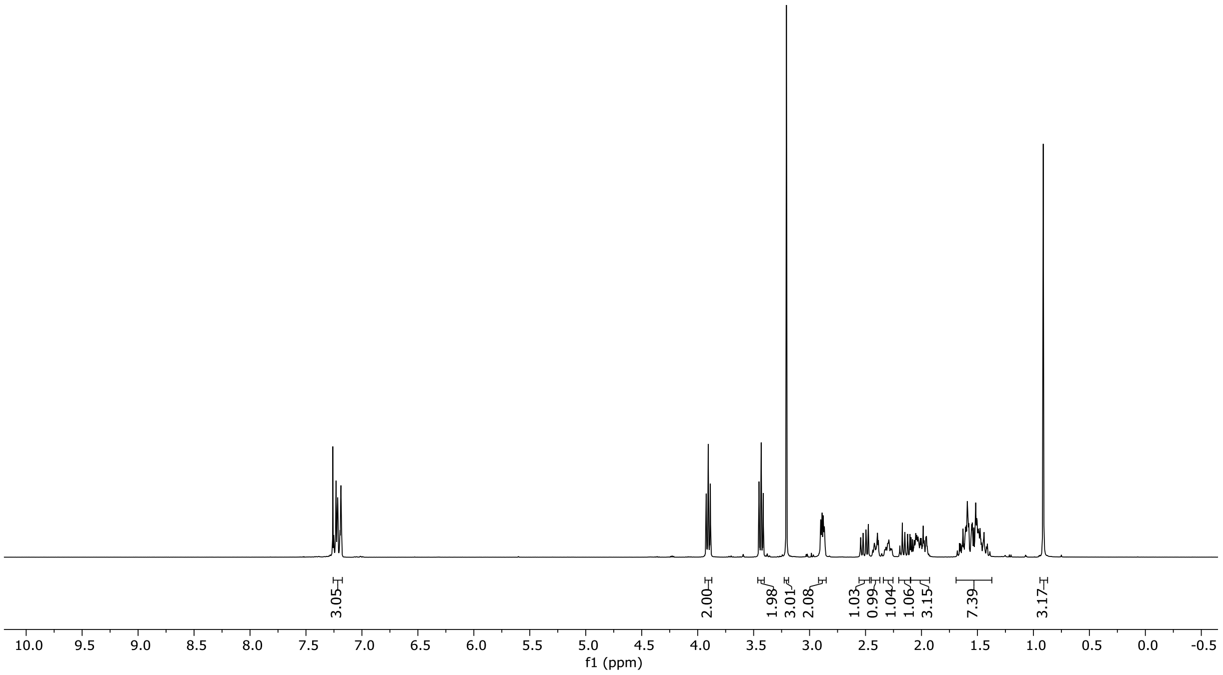

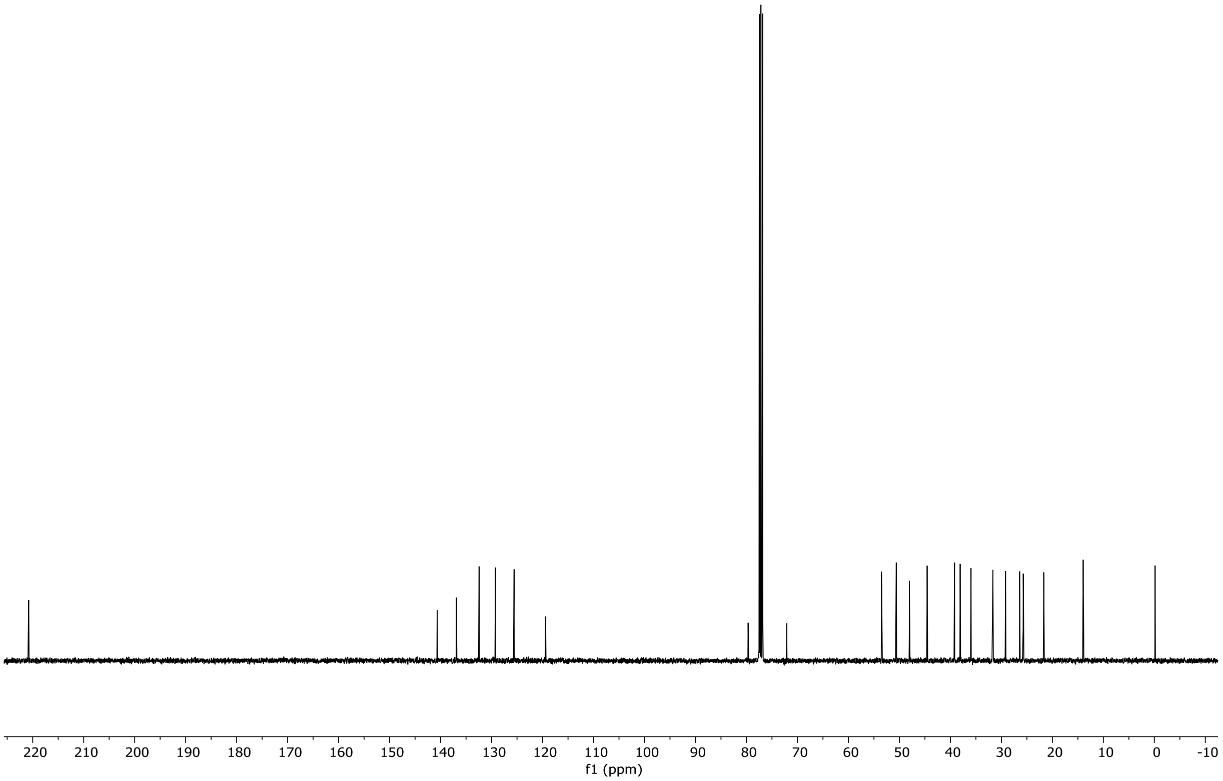

Supplementary Fig 143. ^{1}H (top) and ^{13}C (bottom) NMR spectra of compound **1ah**.

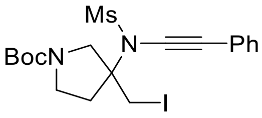

1ai

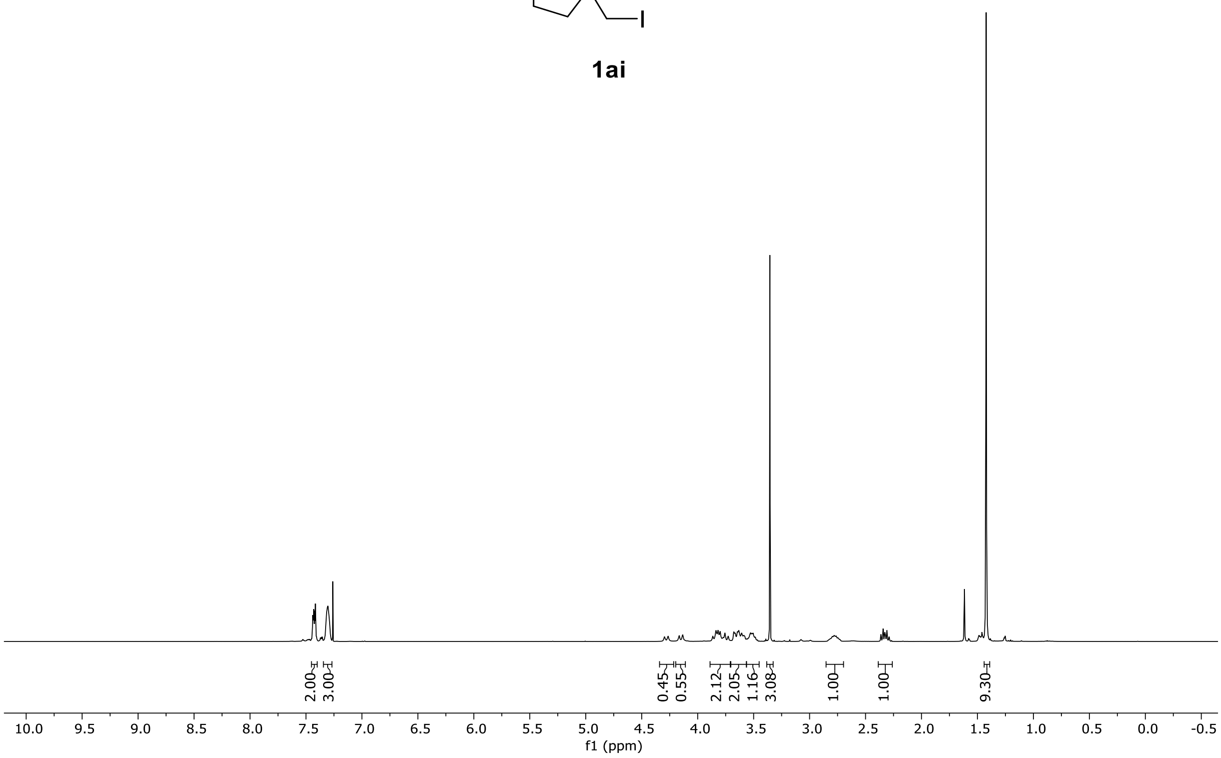

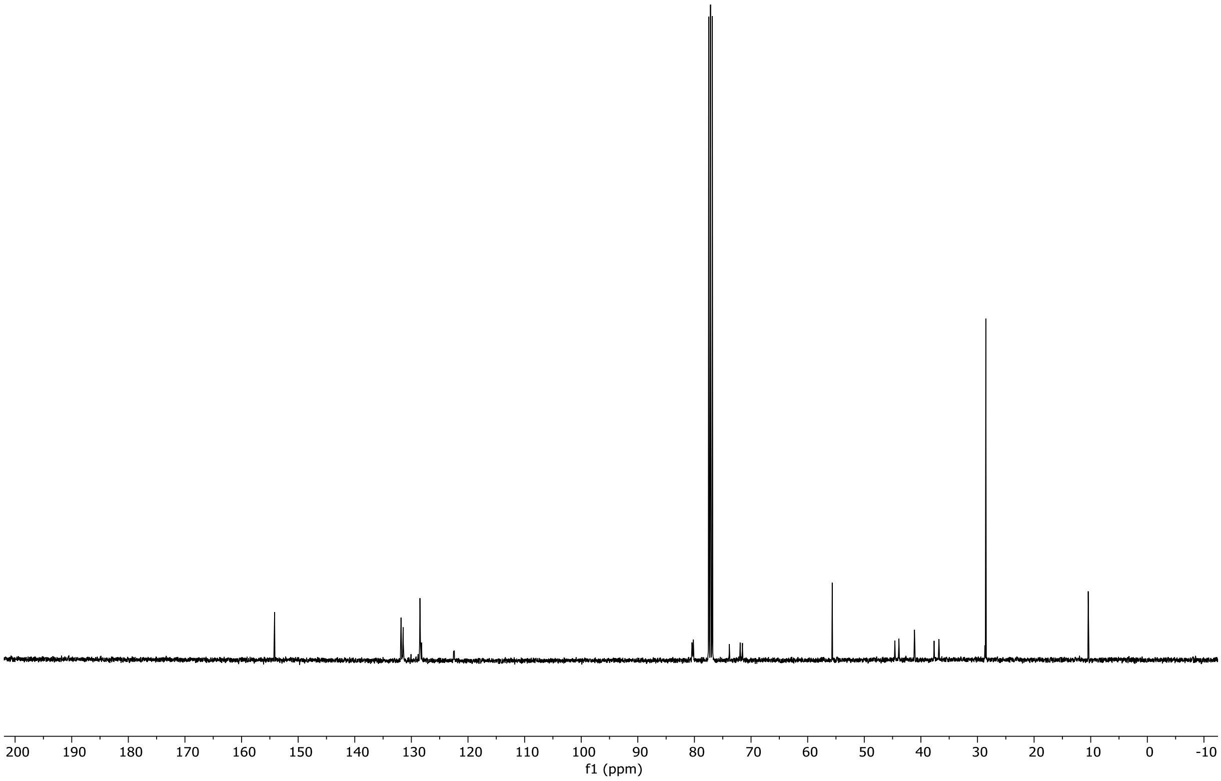

Supplementary Fig 144. ^{1}H (top) and ^{13}C (bottom) NMR spectra of compound **1ai**.

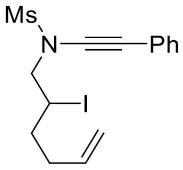

1aj

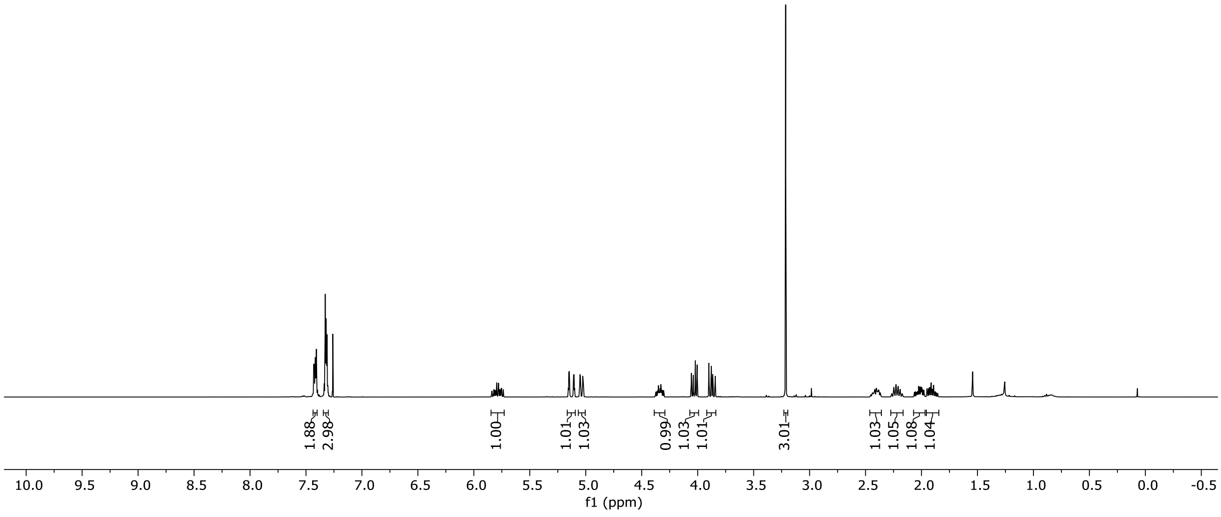

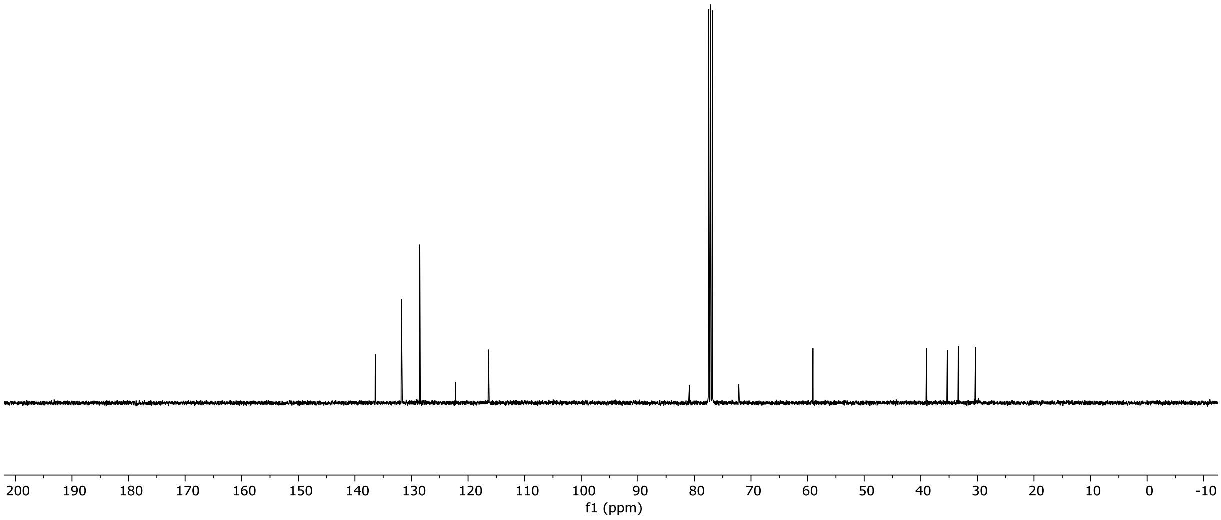

Supplementary Fig 145. ^{1}H (top) and ^{13}C (bottom) NMR spectra of compound **1aj**.

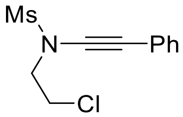

$1a_{Cl}$

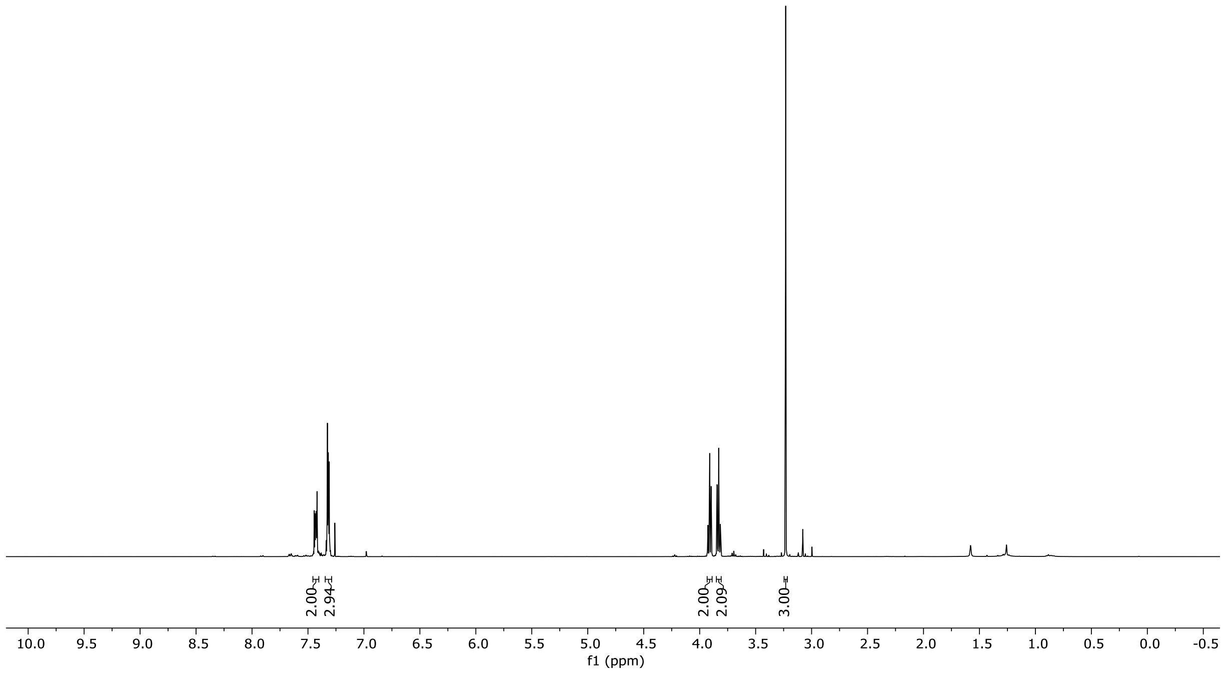

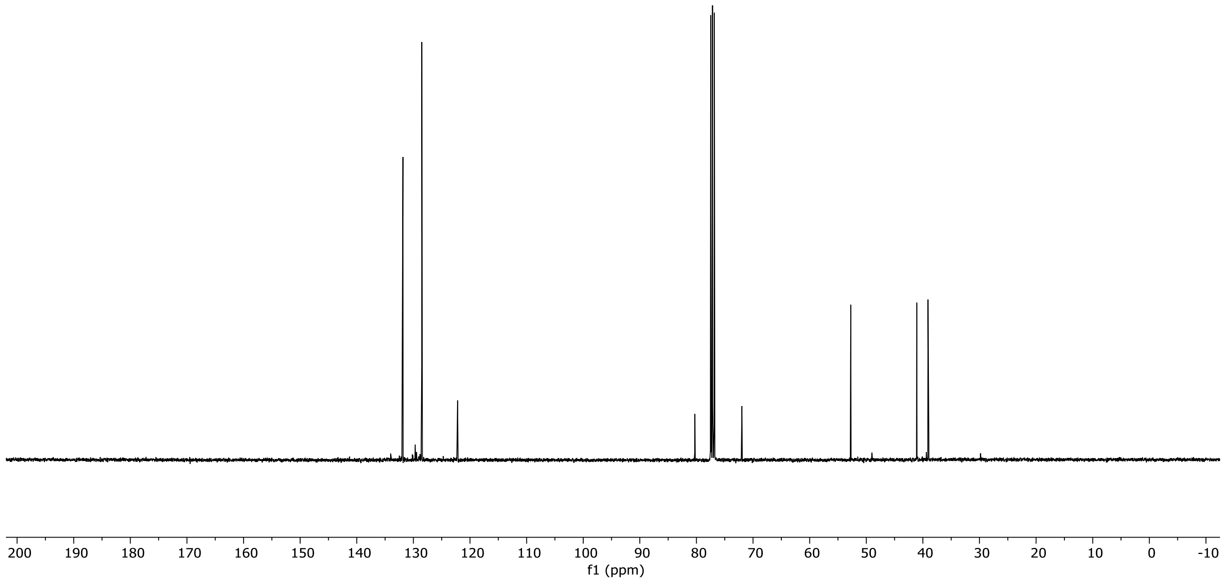

Supplementary Fig 146. ^{1}H (top) and ^{13}C (bottom) NMR spectra of compound **$1a_{Cl}$**.

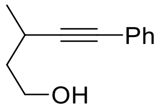

SAB

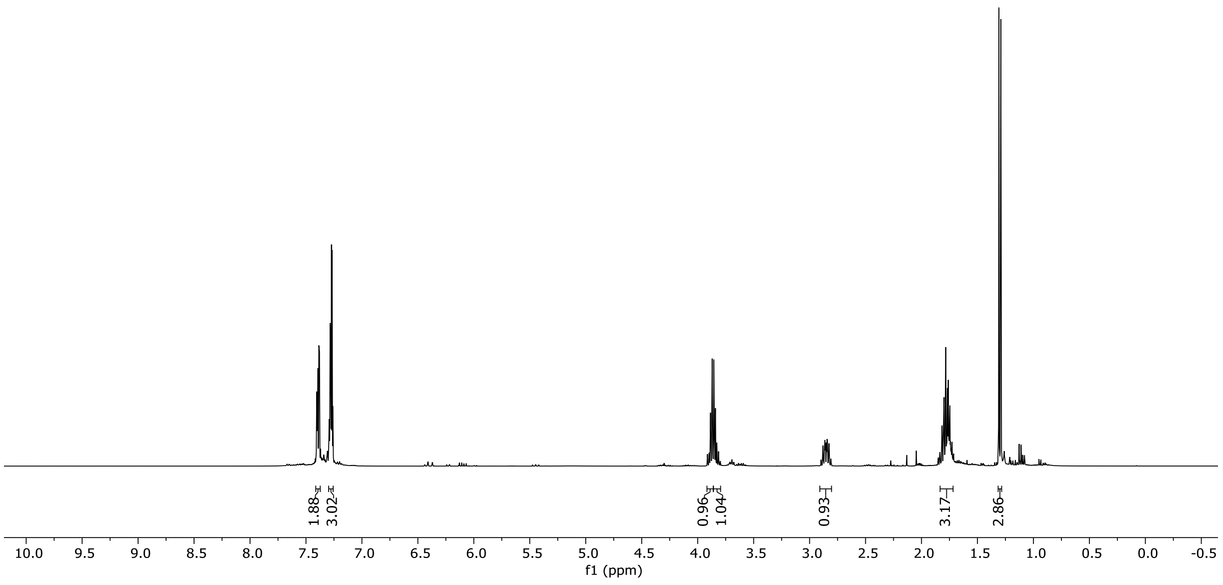

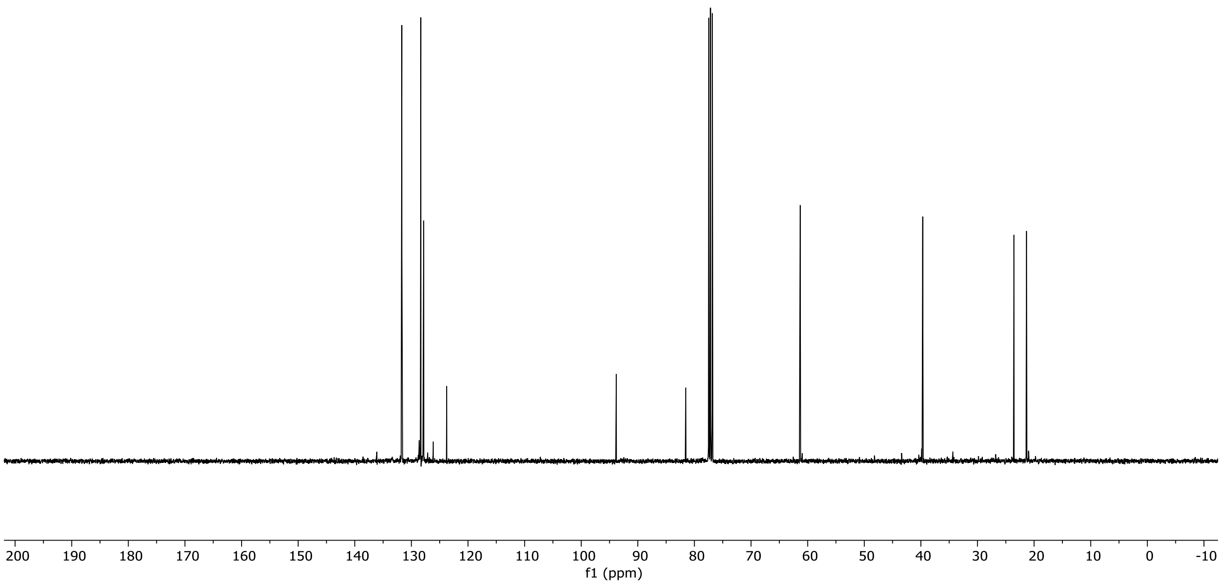

Supplementary Fig 147. ^{1}H (top) and ^{13}C (bottom) NMR spectra of compound **SAB**.

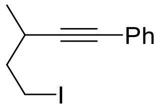

7

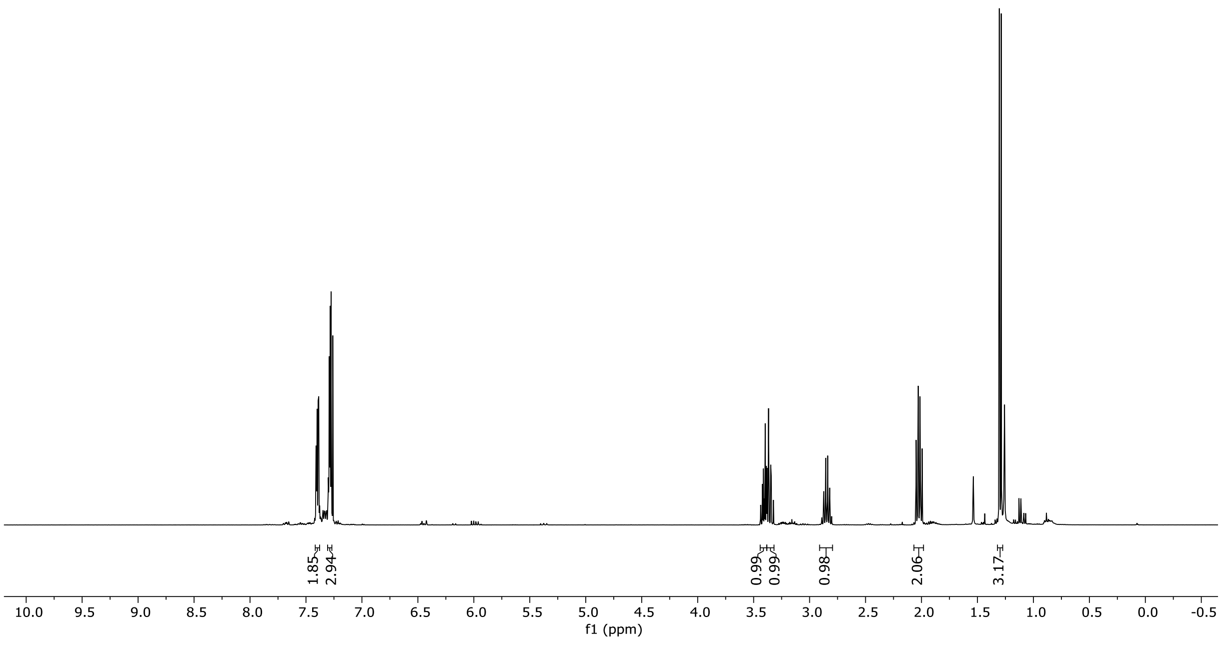

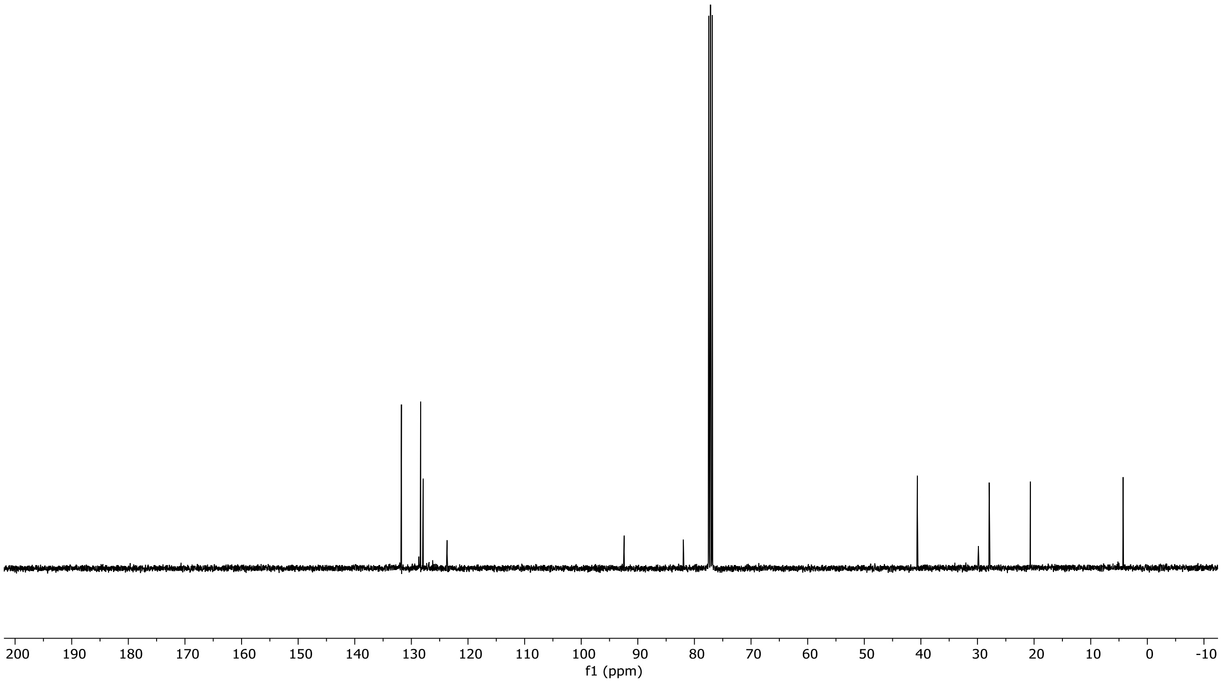

Supplementary Fig 148. ^{1}H (top) and ^{13}C (bottom) NMR spectra of compound **7**.

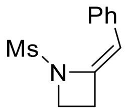

(*Z*)-2a

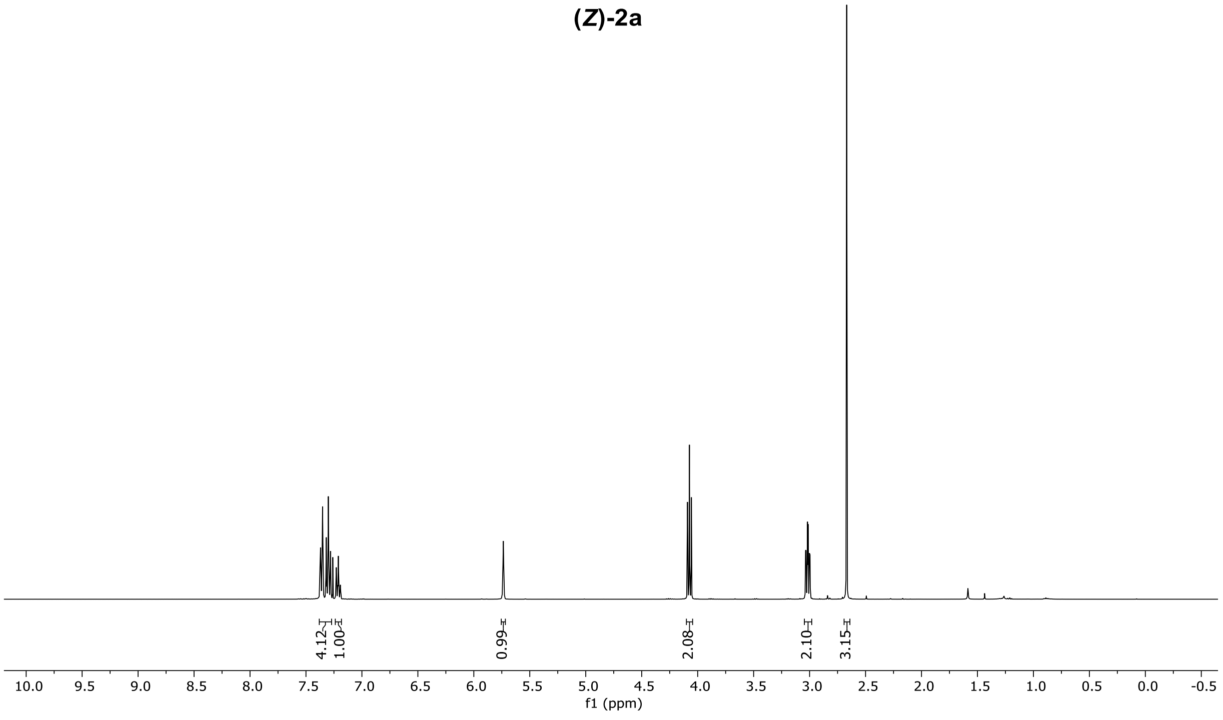

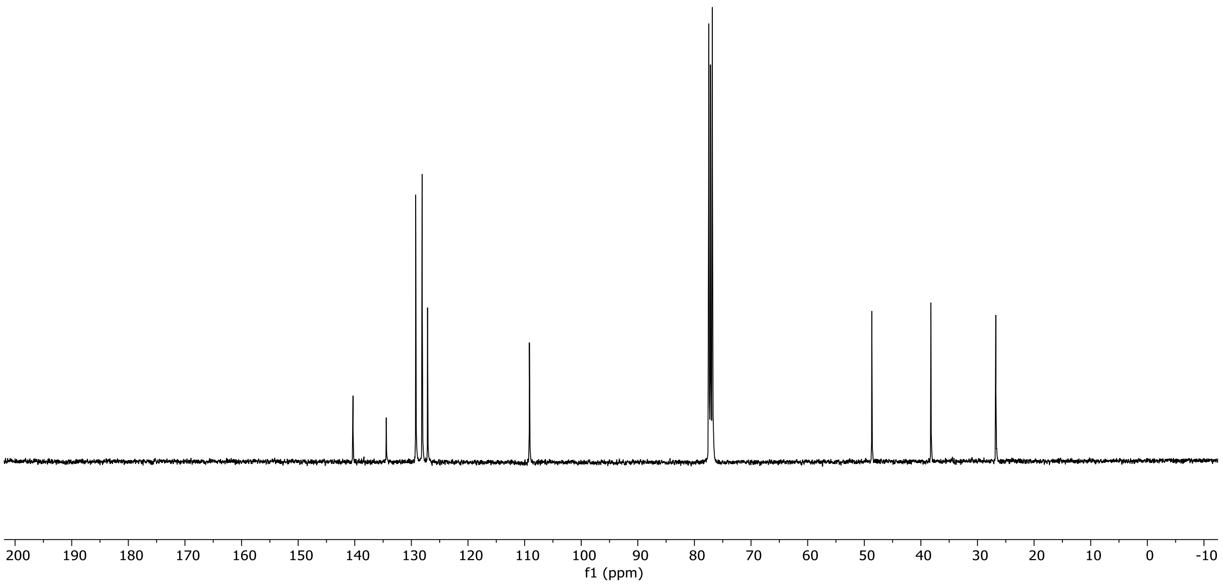

Supplementary Fig 149. ^{1}H (top) and ^{13}C (bottom) NMR spectra of compound **(*Z*)-2a**.

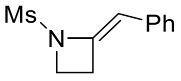

(*E*)-2a

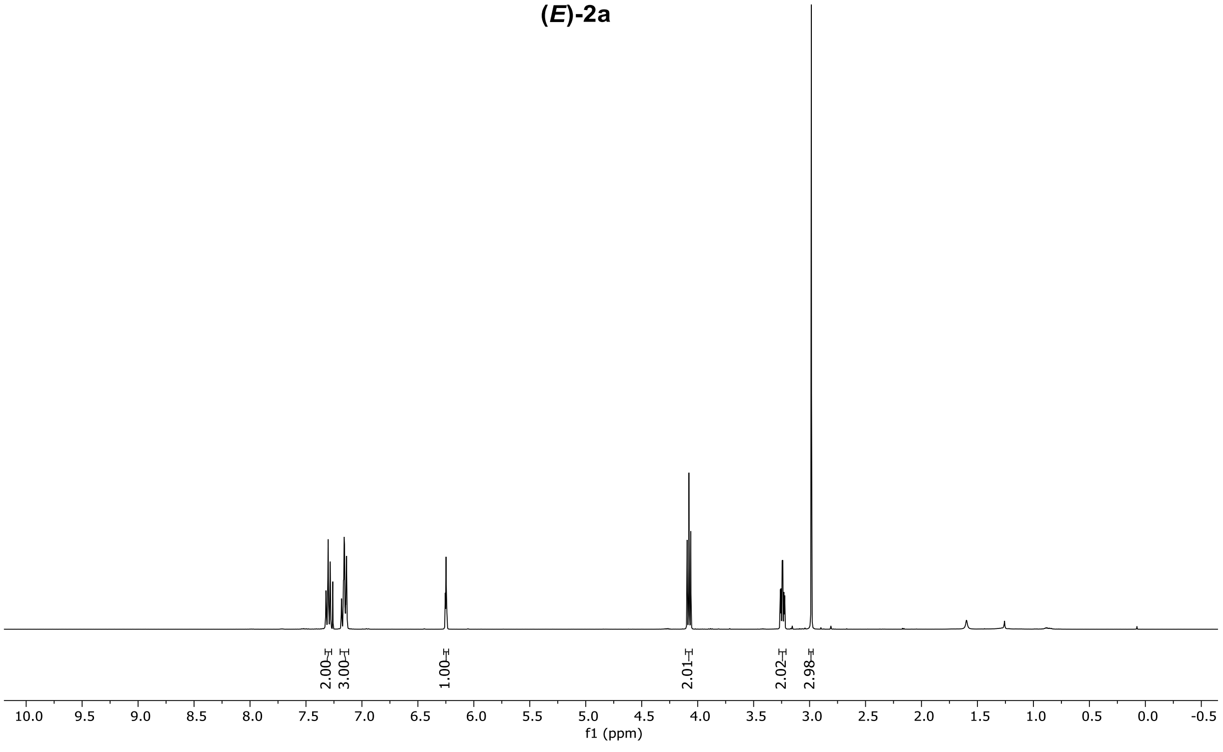

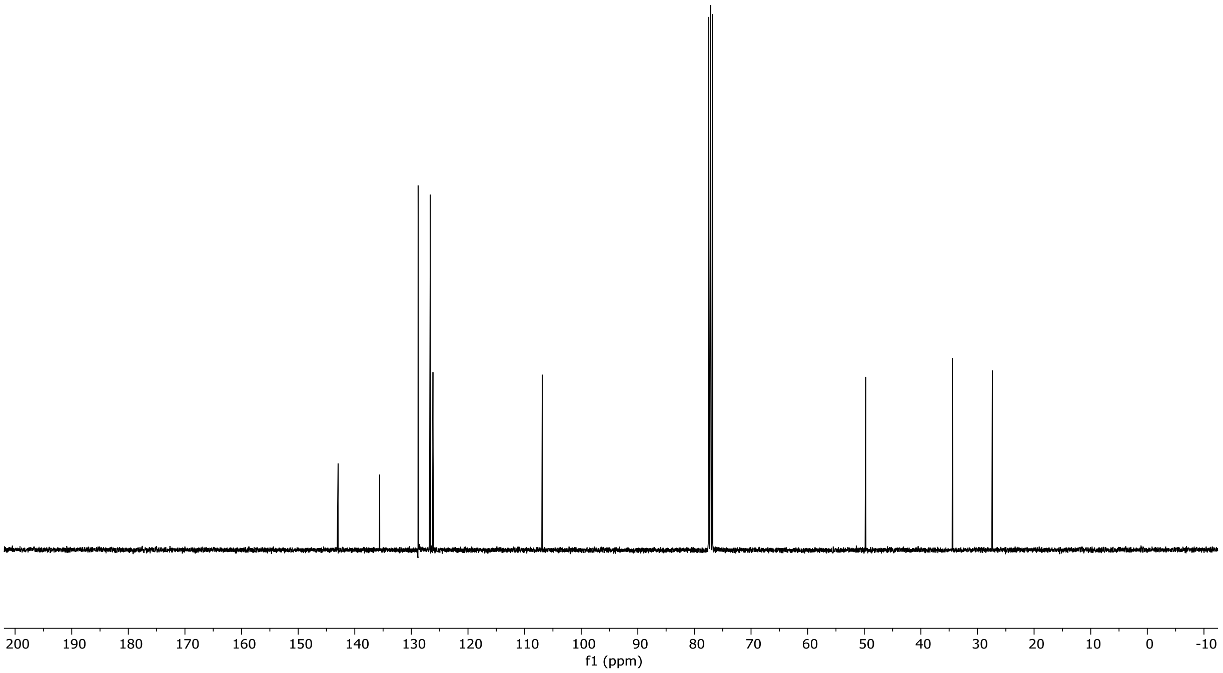

Supplementary Fig 150. ^{1}H (top) and ^{13}C (bottom) NMR spectra of compound **(*E*)-2a**.

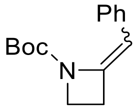

2b

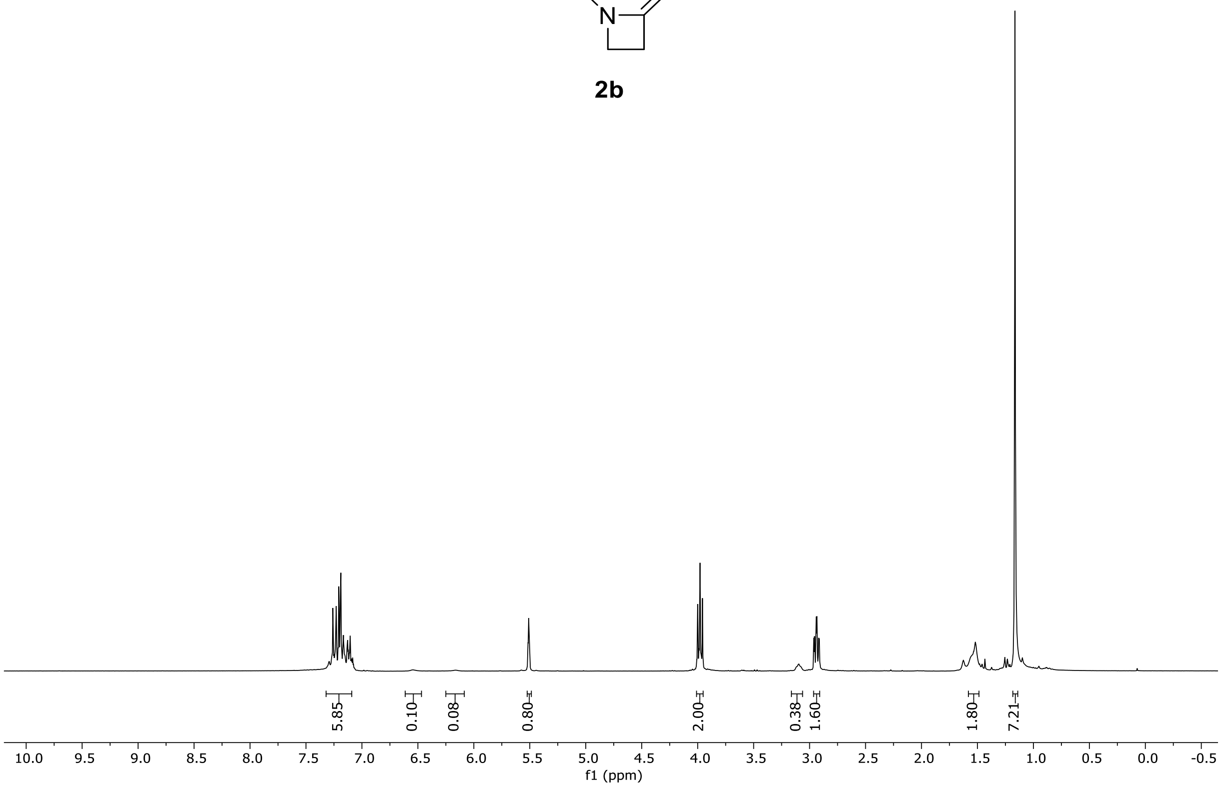

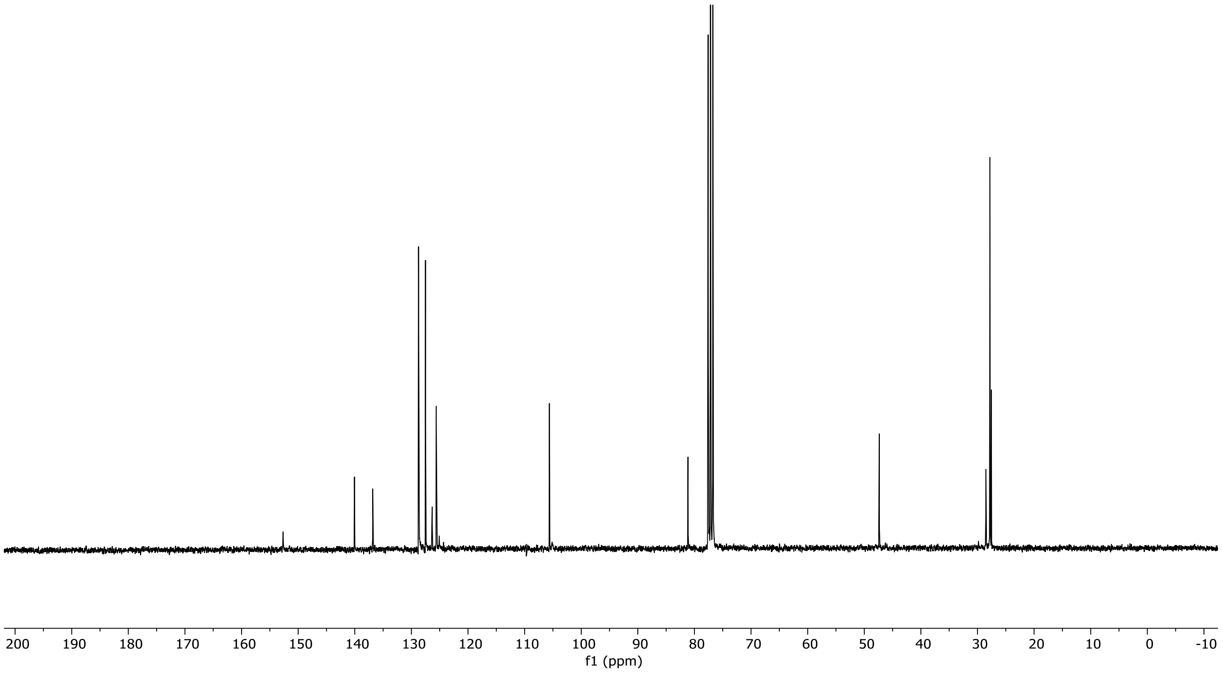

Supplementary Fig 151. ^{1}H (top) and ^{13}C (bottom) NMR spectra of compound **2b**.

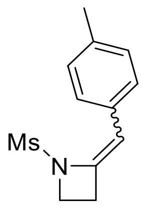

2c

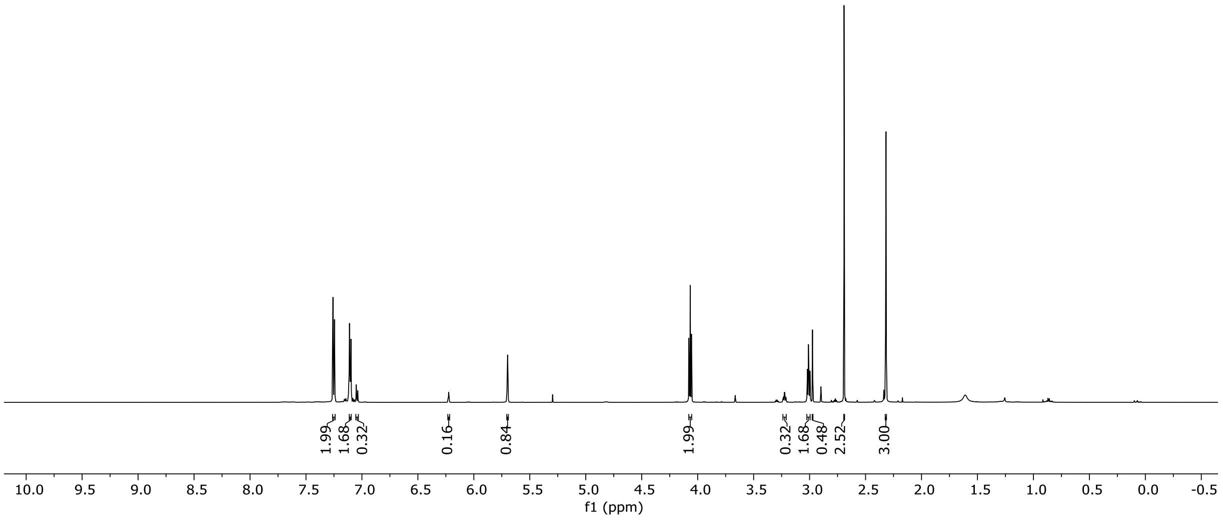

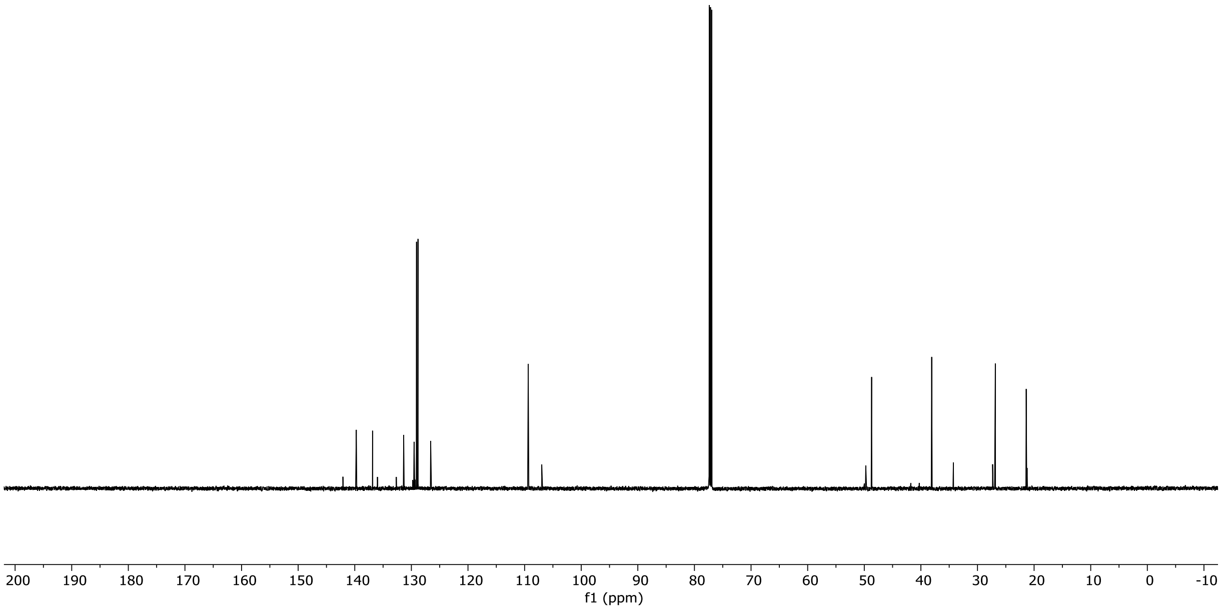

Supplementary Fig 152. ^{1}H (top) and ^{13}C (bottom) NMR spectra of compound **2c**.

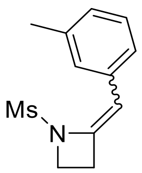

2d

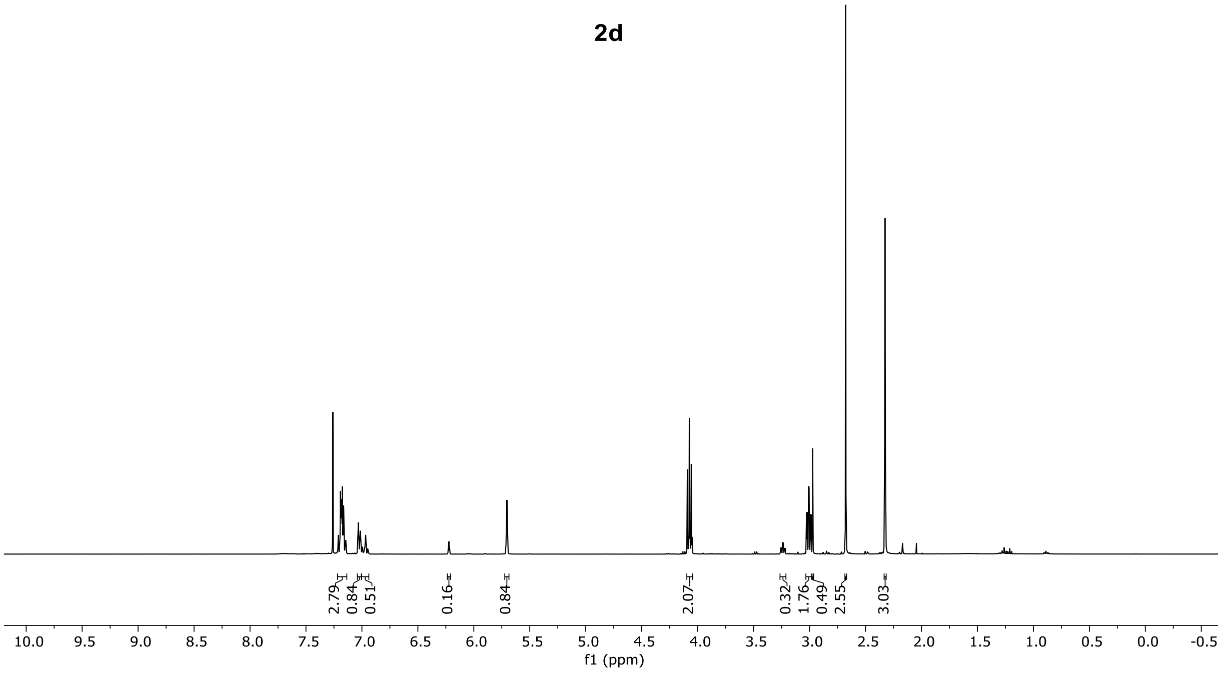

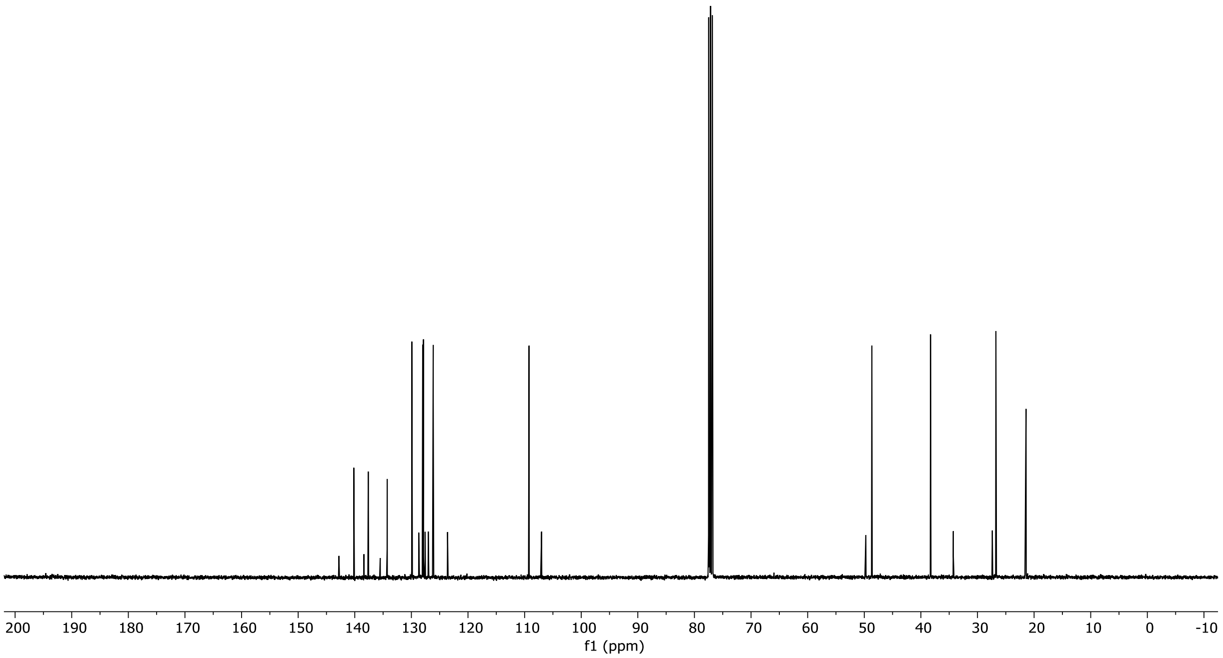

Supplementary Fig 153. ^{1}H (top) and ^{13}C (bottom) NMR spectra of compound **2d**.

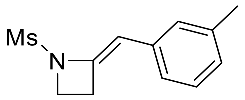

(*E*)-2d

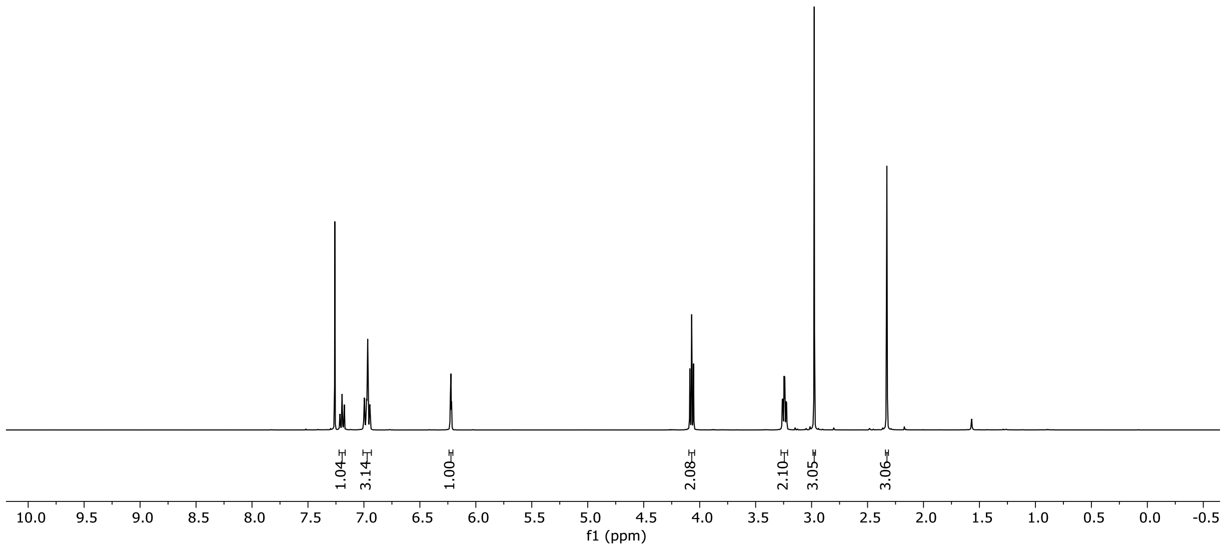

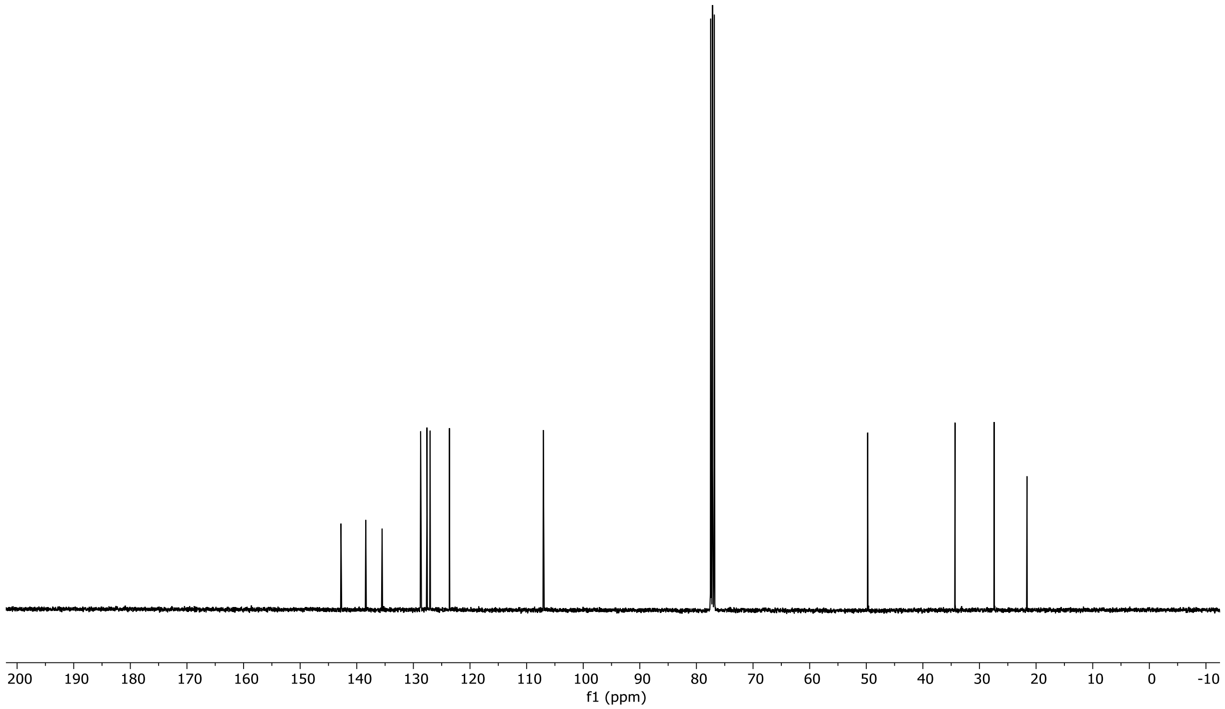

Supplementary Fig 154. ^{1}H (top) and ^{13}C (bottom) NMR spectra of compound **(*E*)-2d**.

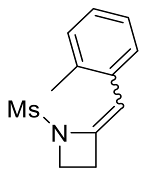

2e

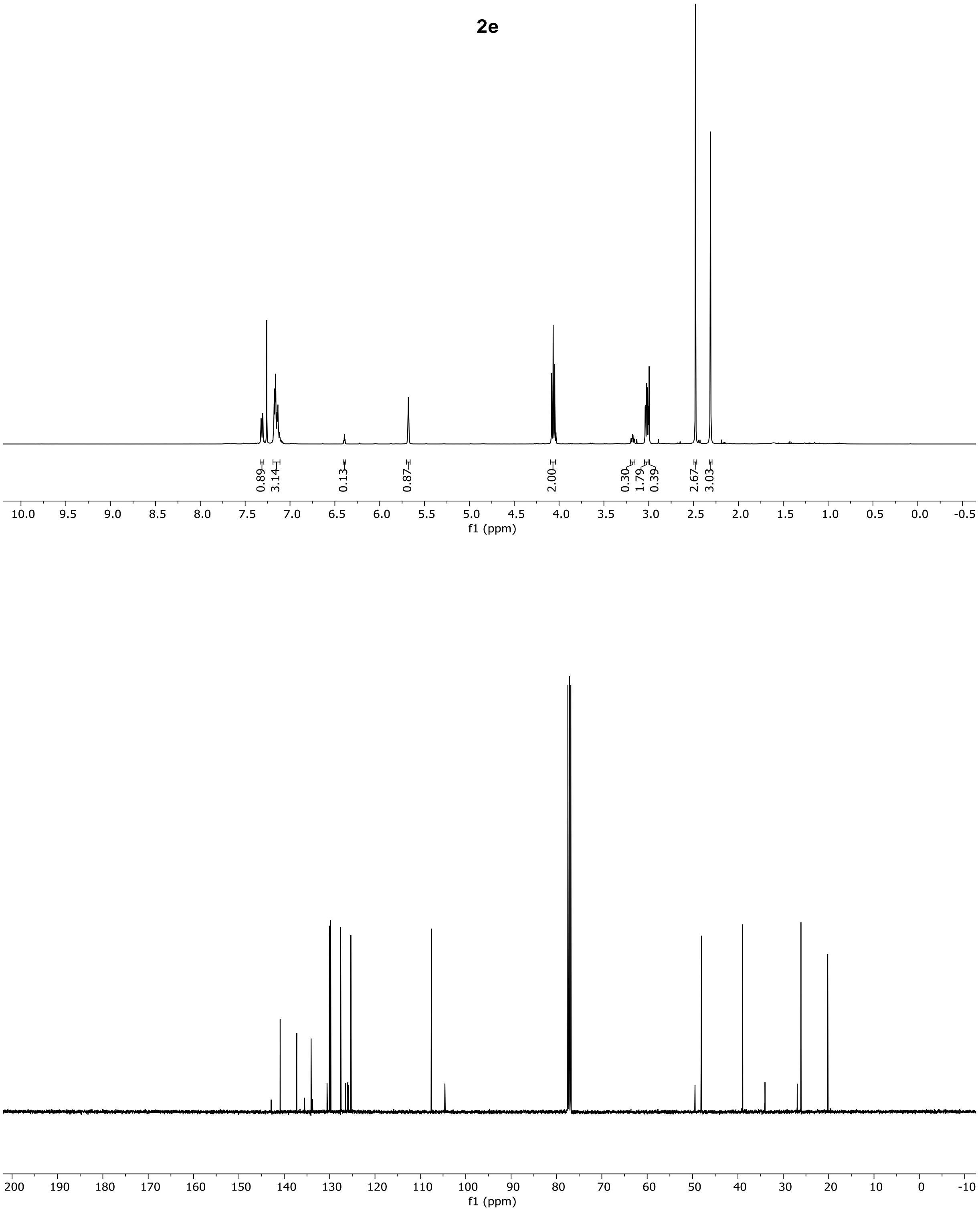

Supplementary Fig 155. ^{1}H (top) and ^{13}C (bottom) NMR spectra of compound **2e**.

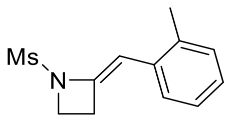

(*E*)-2e

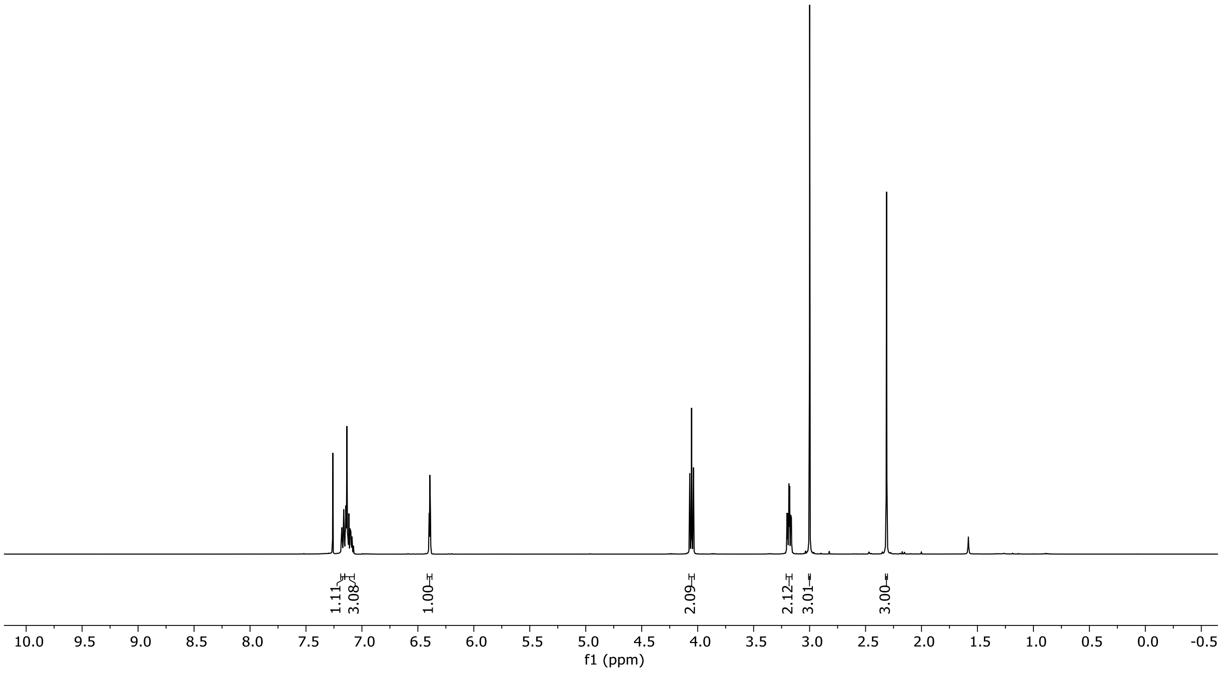

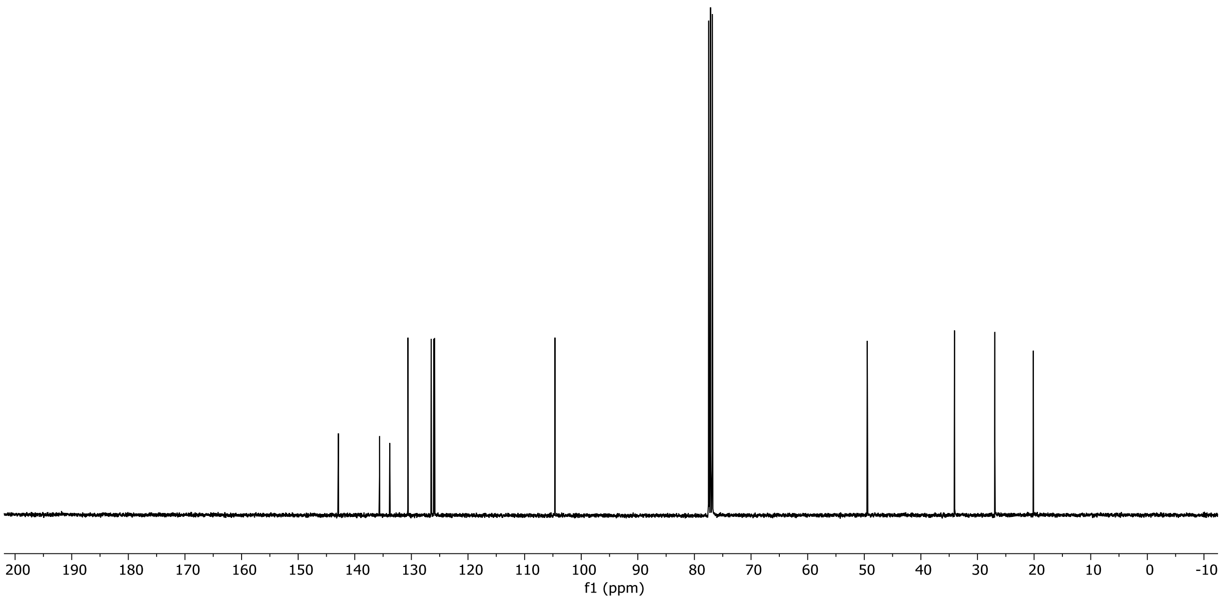

Supplementary Fig 156. ^{1}H (top) and ^{13}C (bottom) NMR spectra of compound **(*E*)-2e**.

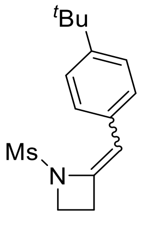

2f

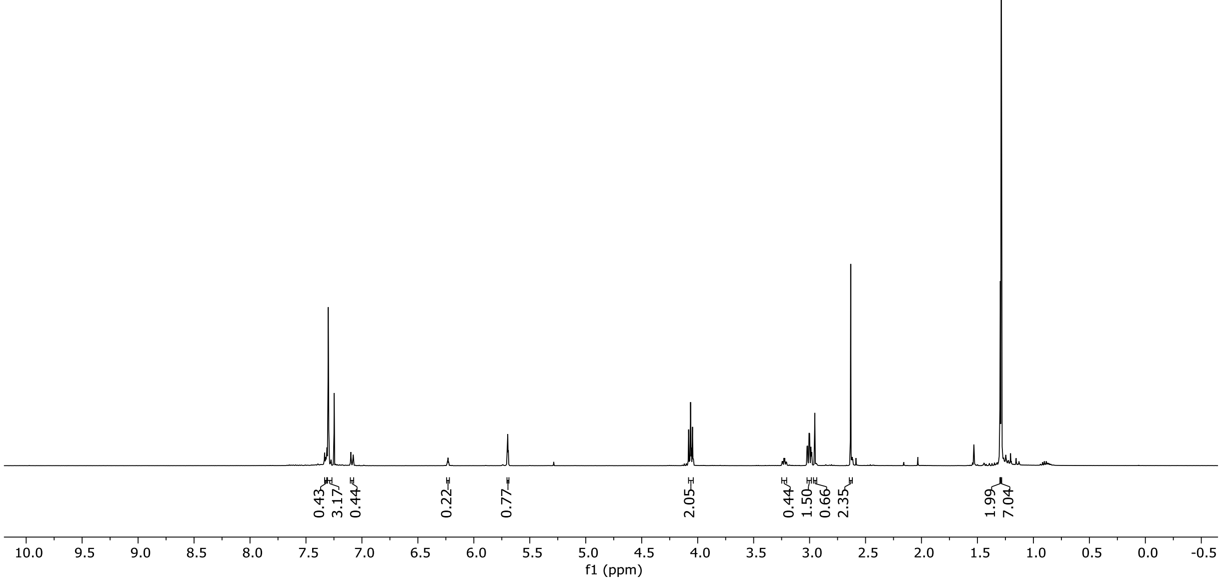

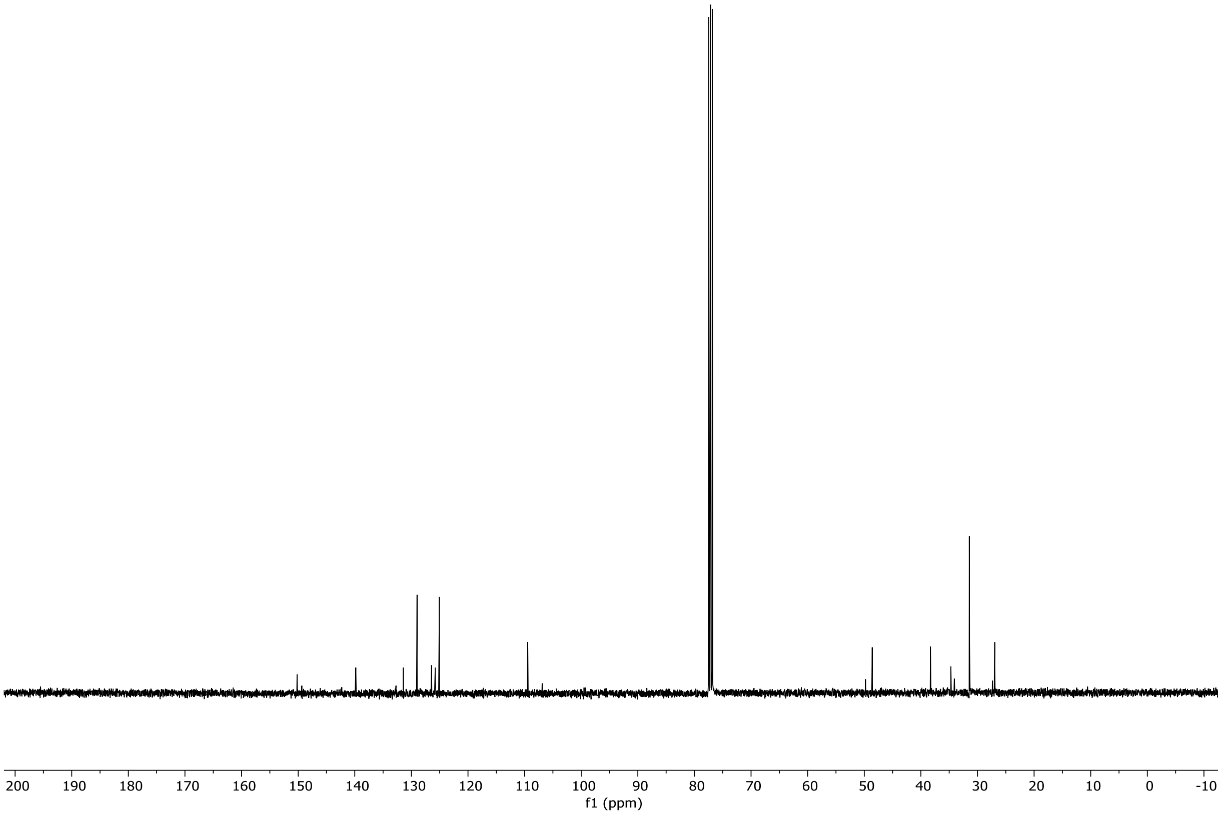

Supplementary Fig 157. ^{1}H (top) and ^{13}C (bottom) NMR spectra of compound **2f**.

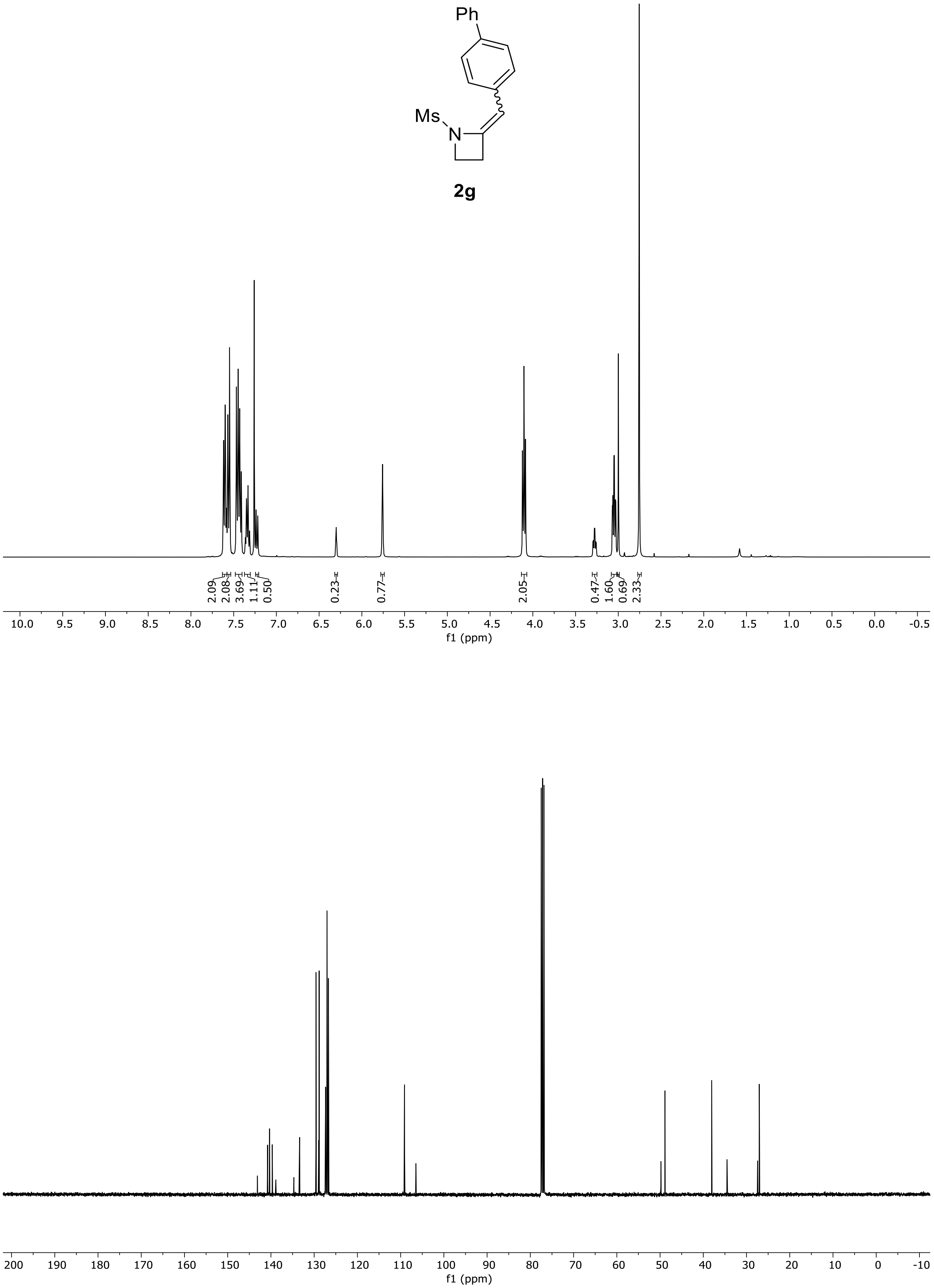

Supplementary Fig 158. ^{1}H (top) and ^{13}C (bottom) NMR spectra of compound **2g**.

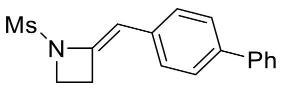

(*E*)-2g

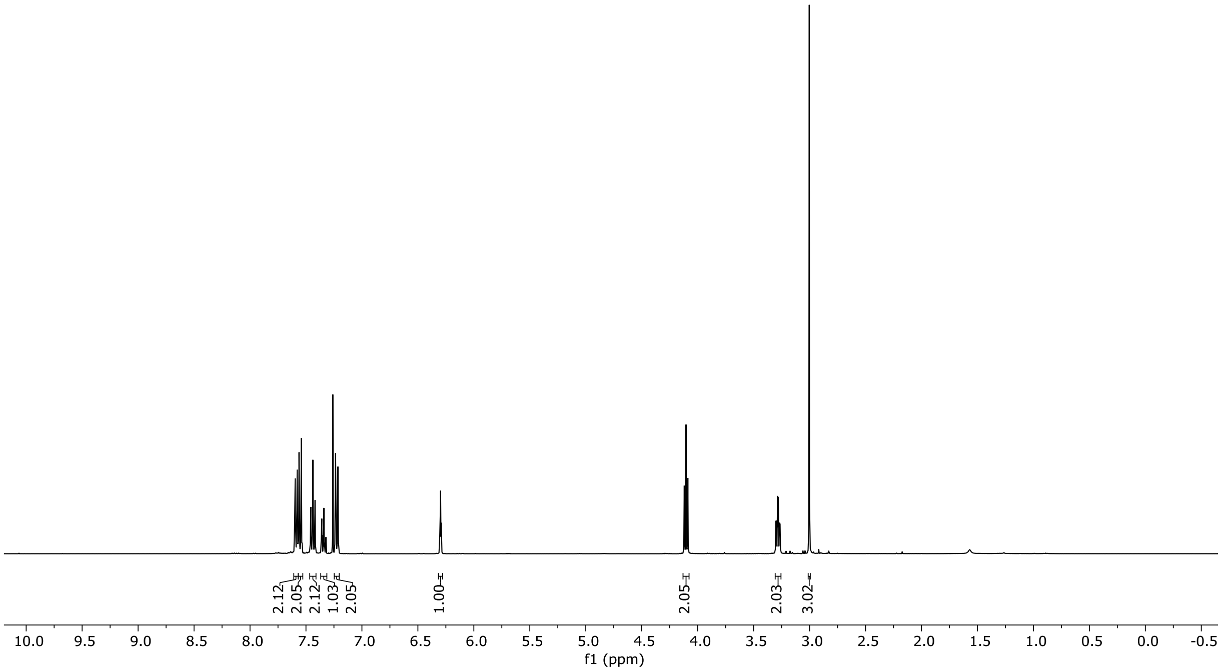

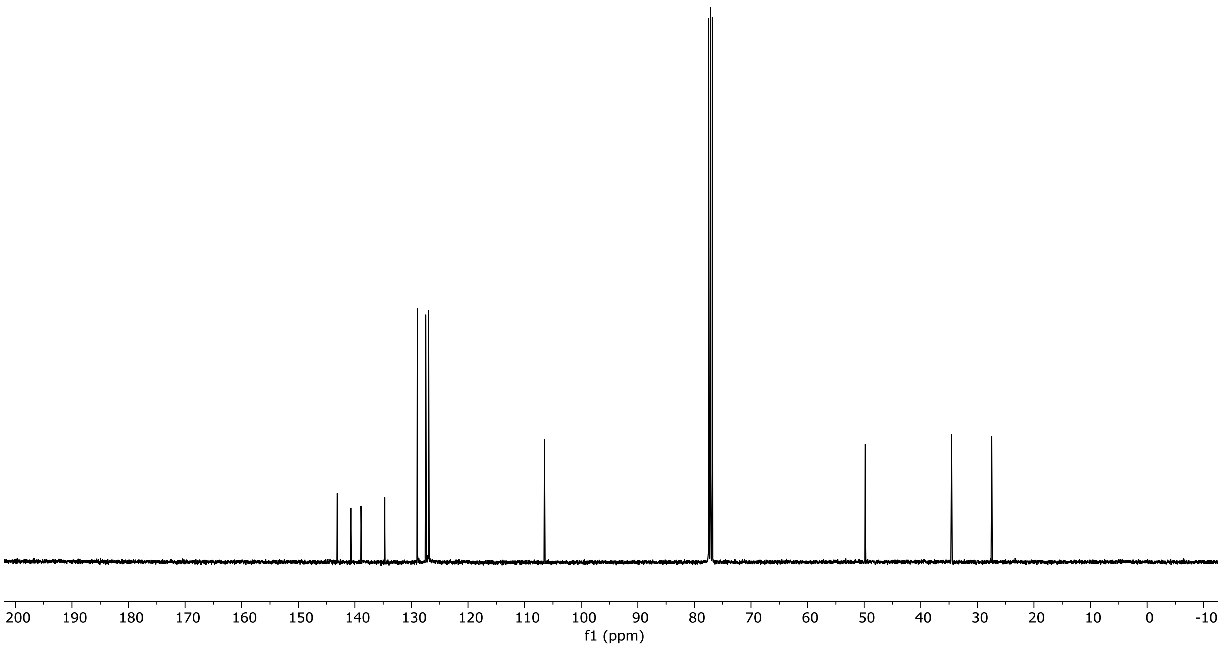

Supplementary Fig 159. ^{1}H (top) and ^{13}C (bottom) NMR spectra of compound **(*E*)-2g**.

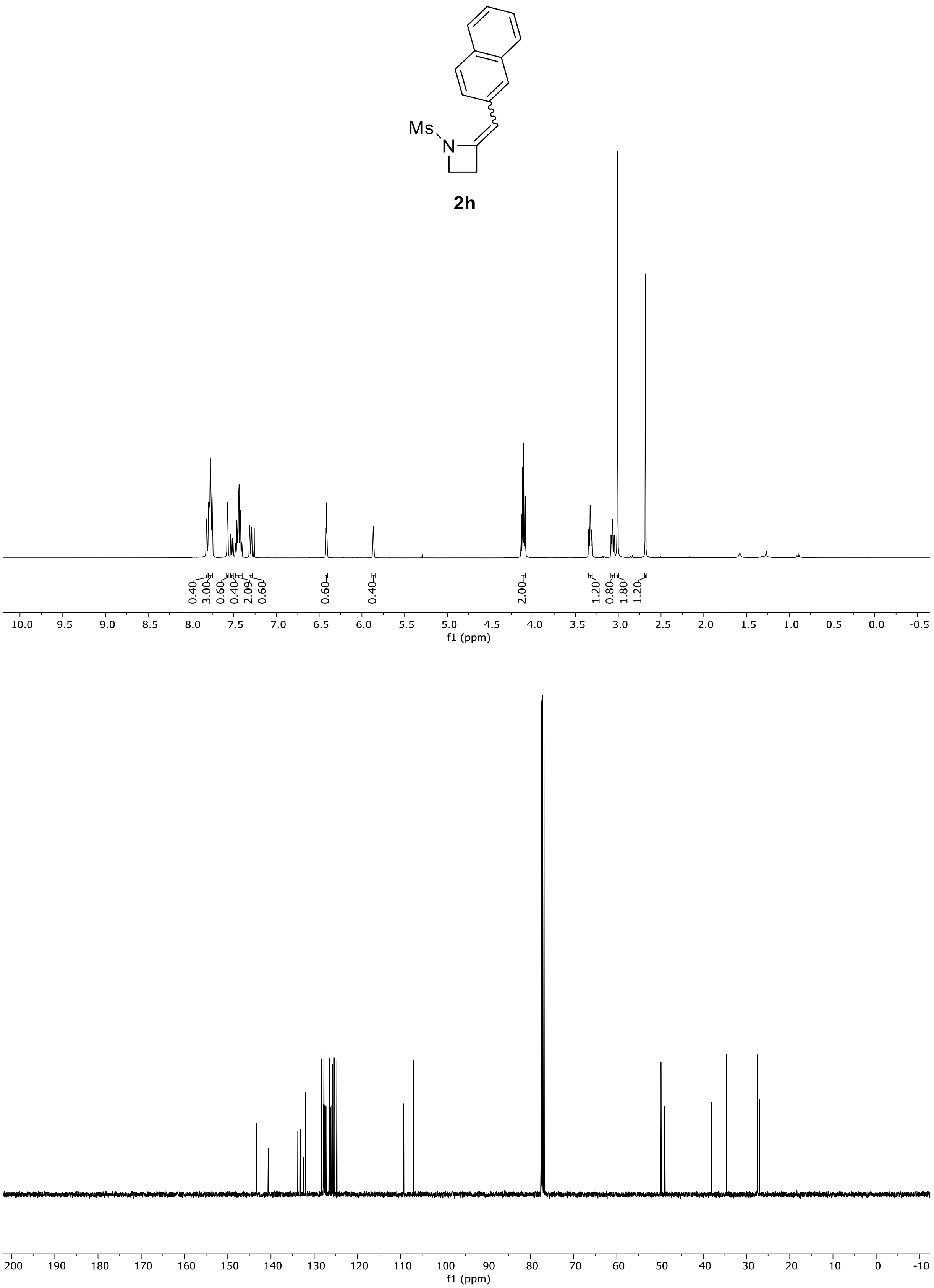

Supplementary Fig 160. ^{1}H (top) and ^{13}C (bottom) NMR spectra of compound **2h**.

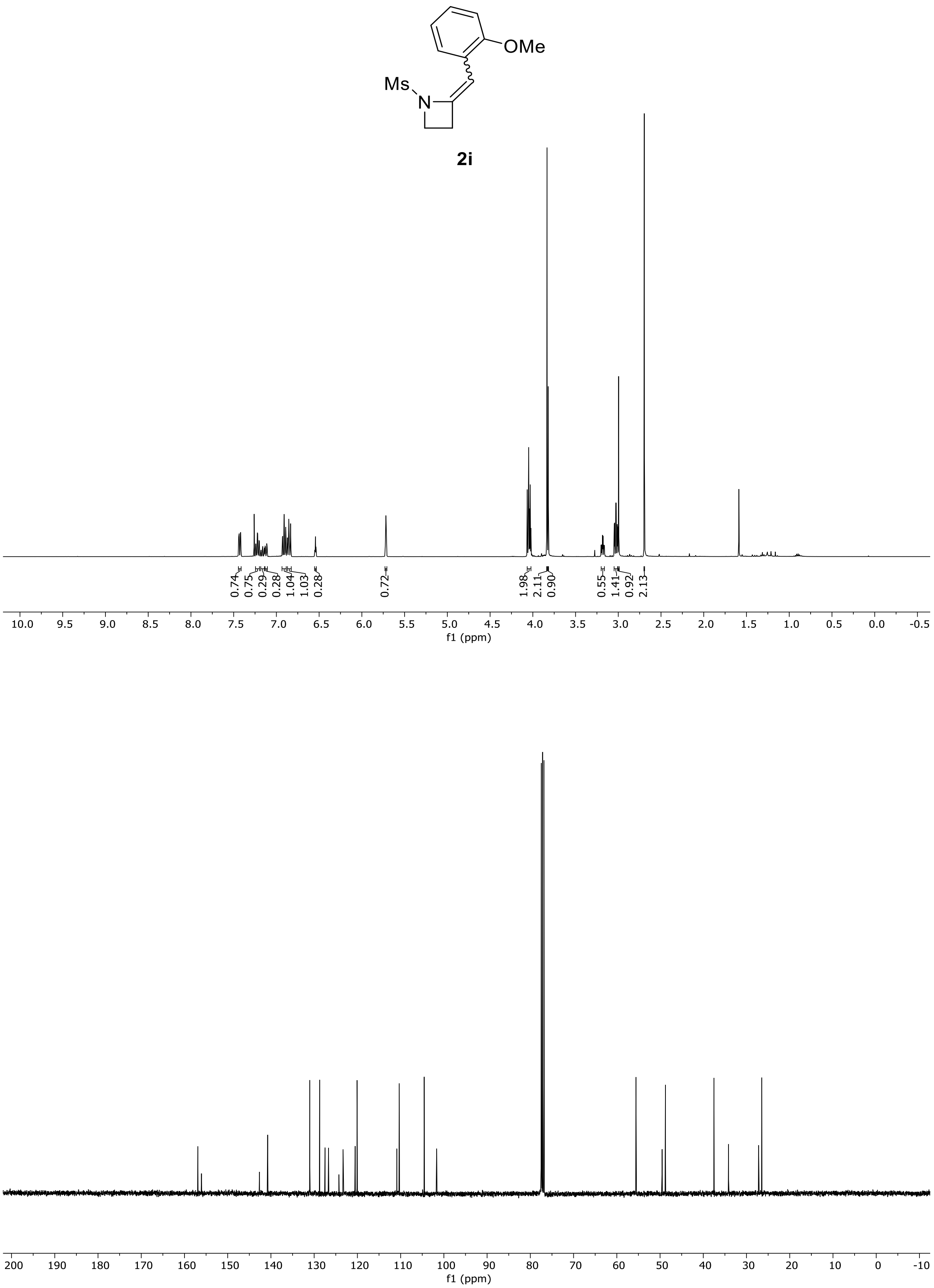

Supplementary Fig 161. ^{1}H (top) and ^{13}C (bottom) NMR spectra of compound **2i**.

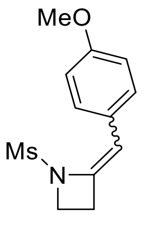

2j

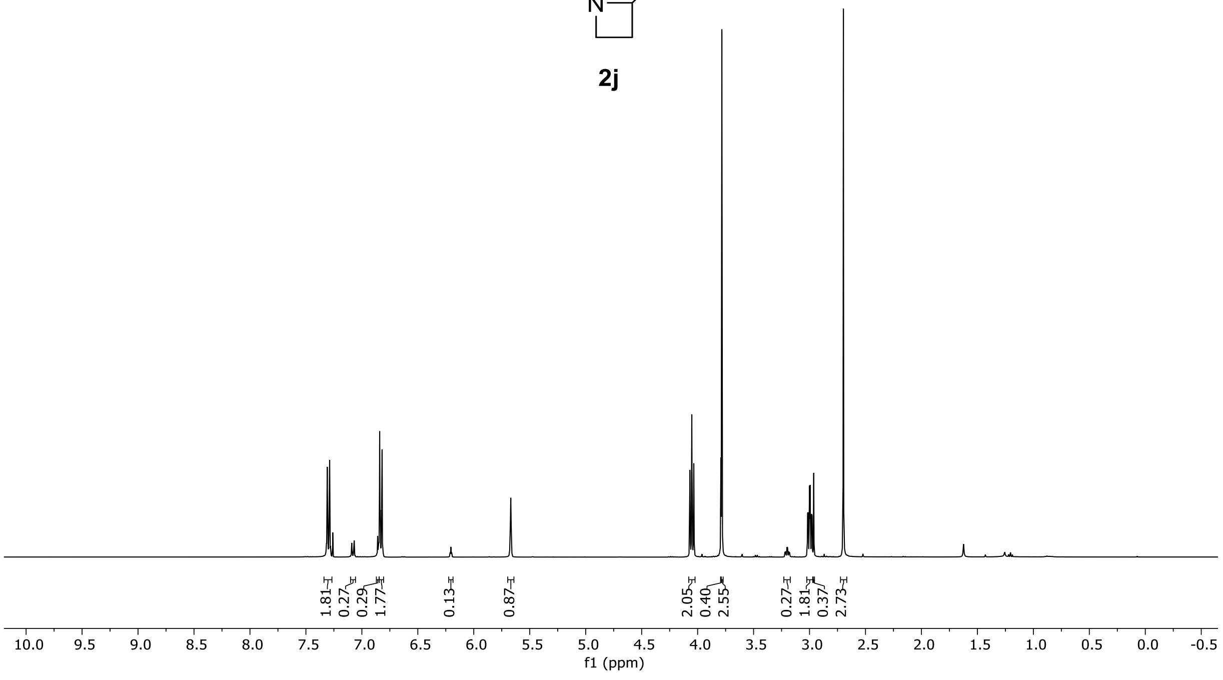

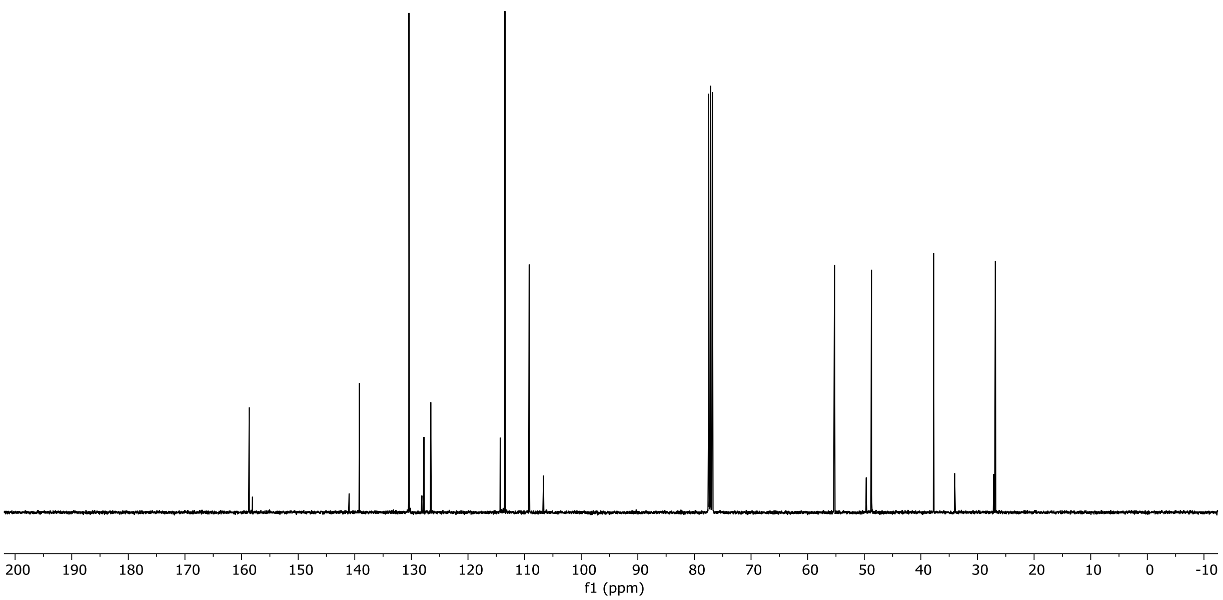

Supplementary Fig 162. ^{1}H (top) and ^{13}C (bottom) NMR spectra of compound **2j**.

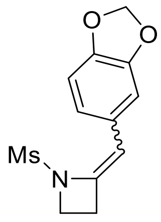

2k

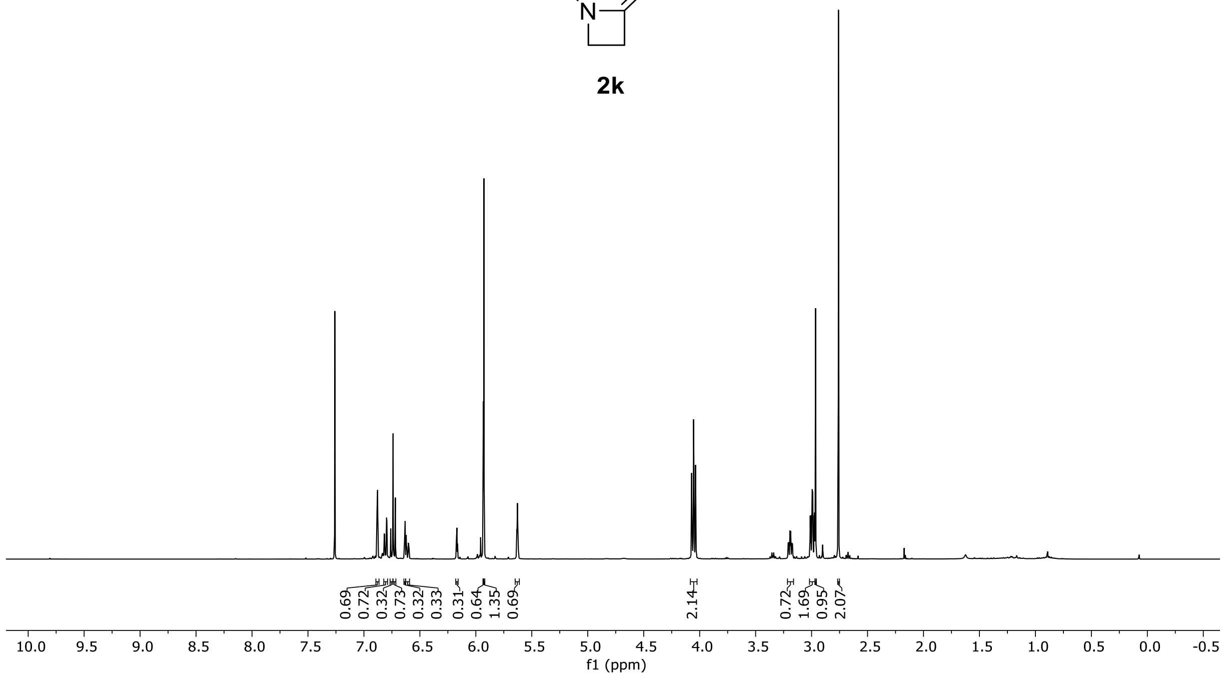

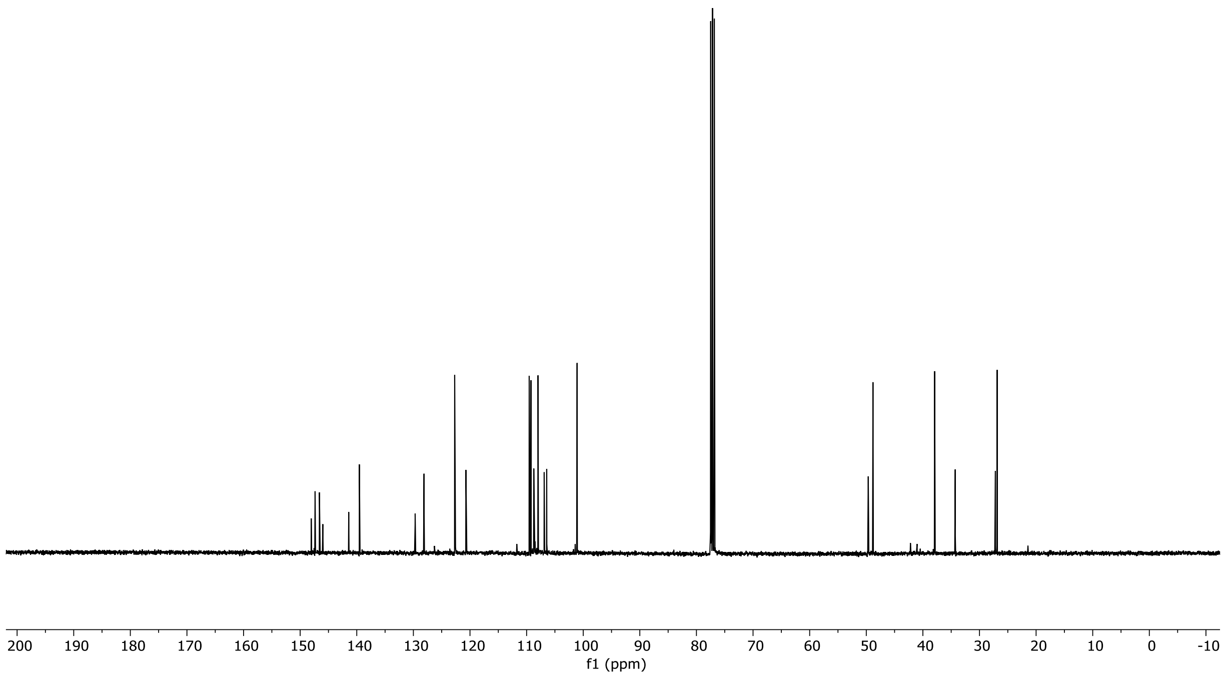

Supplementary Fig 163. ^{1}H (top) and ^{13}C (bottom) NMR spectra of compound **2k**.

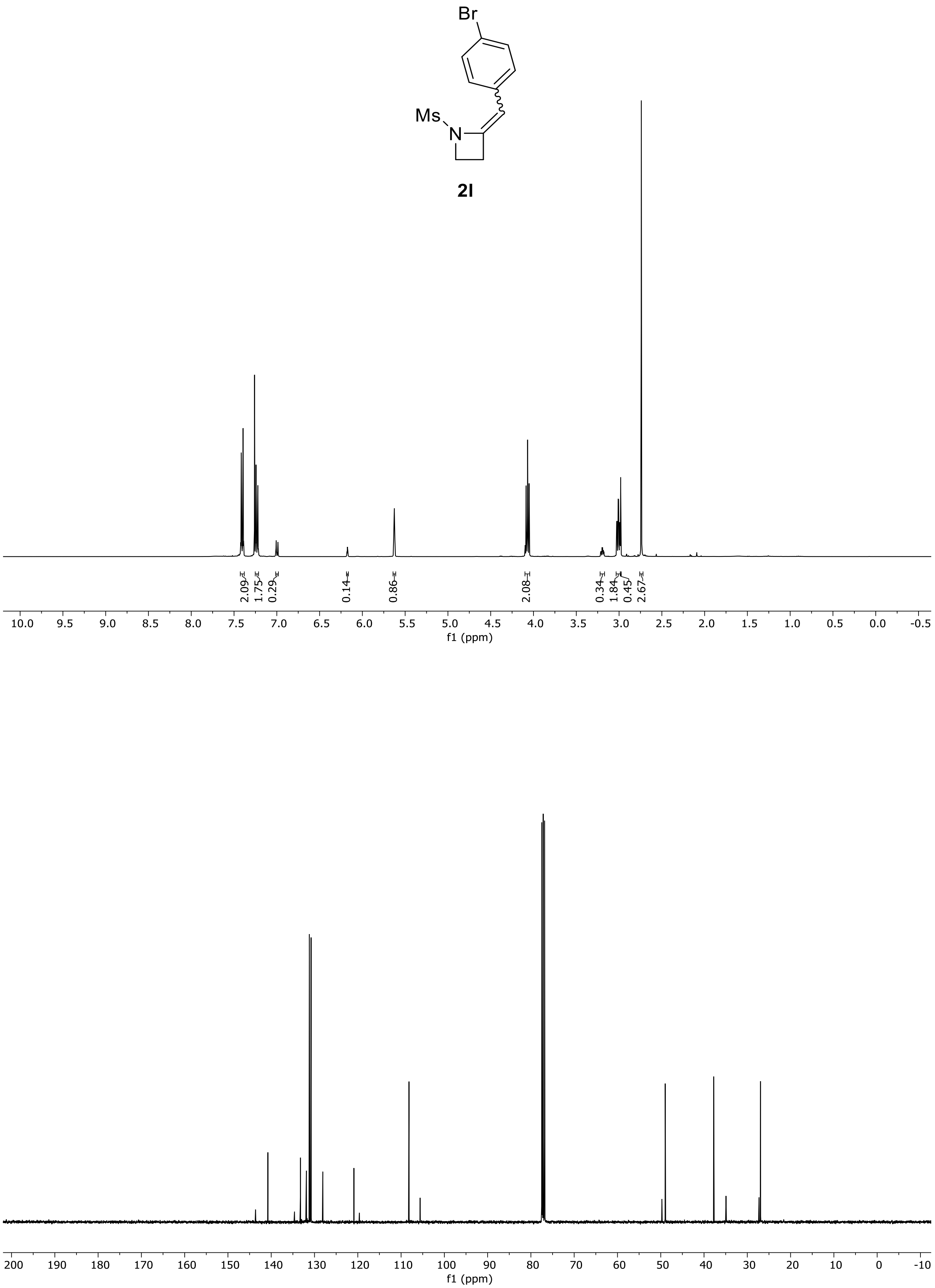

Supplementary Fig 164. ^{1}H (top) and ^{13}C (bottom) NMR spectra of compound **2l**.

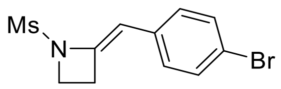

(*E*)-2l

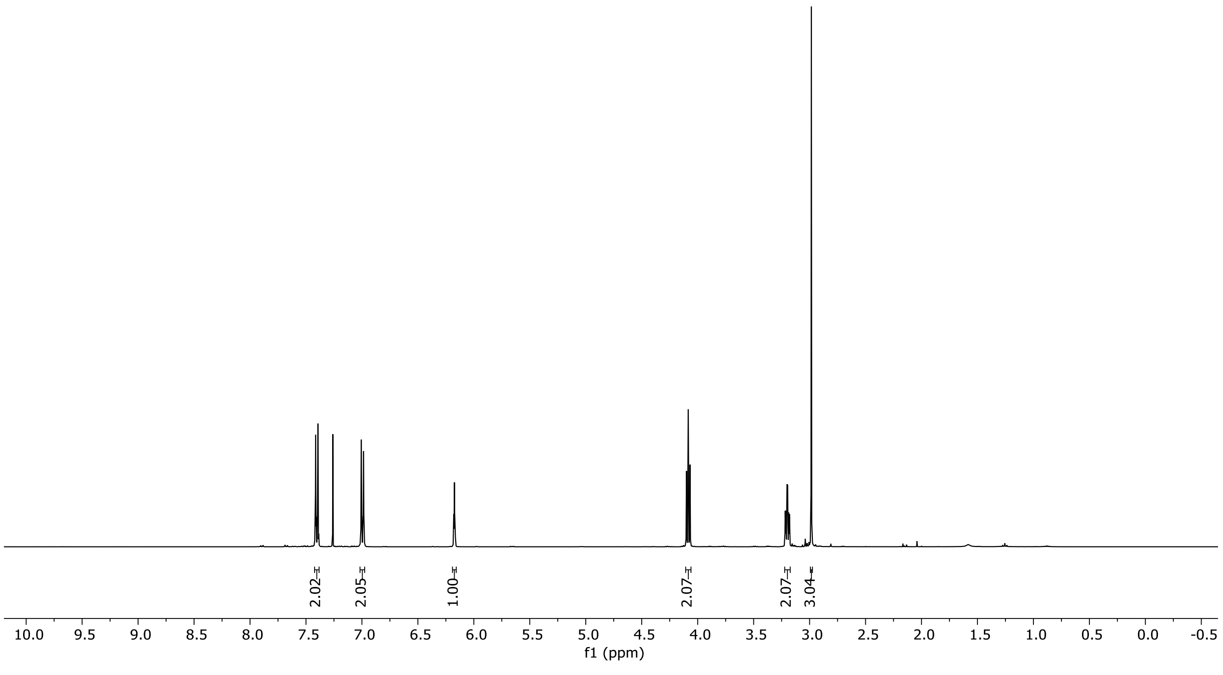

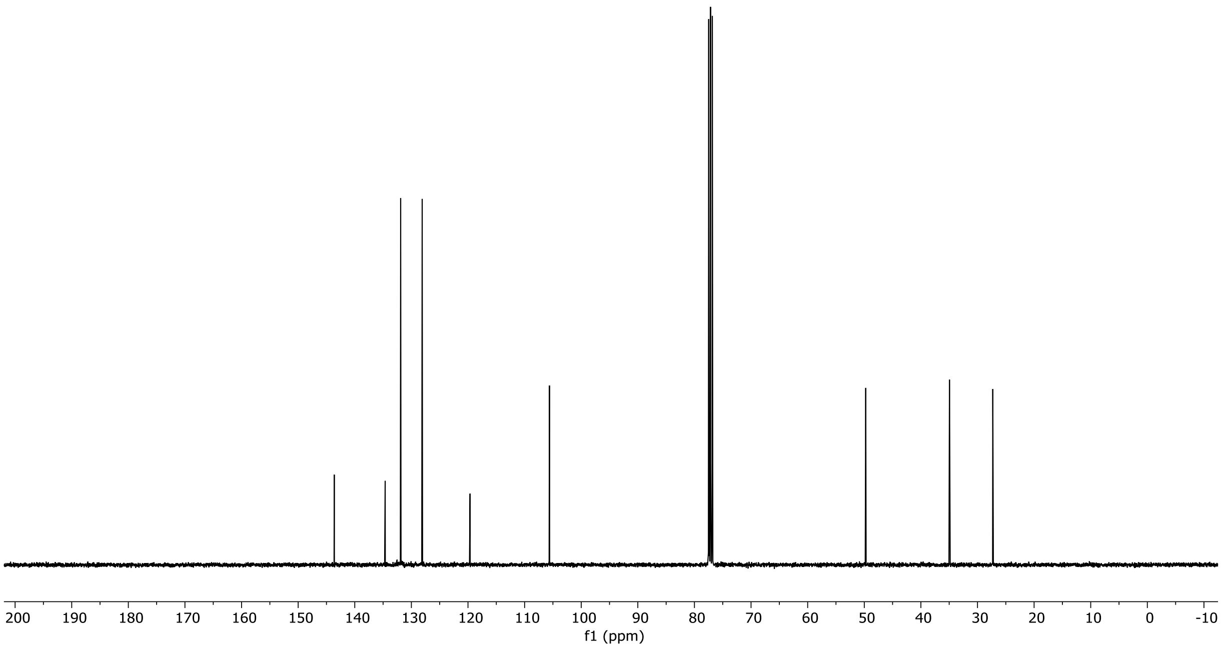

Supplementary Fig 165. ^{1}H (top) and ^{13}C (bottom) NMR spectra of compound **(*E*)-2l**.

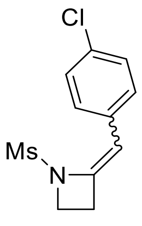

2m

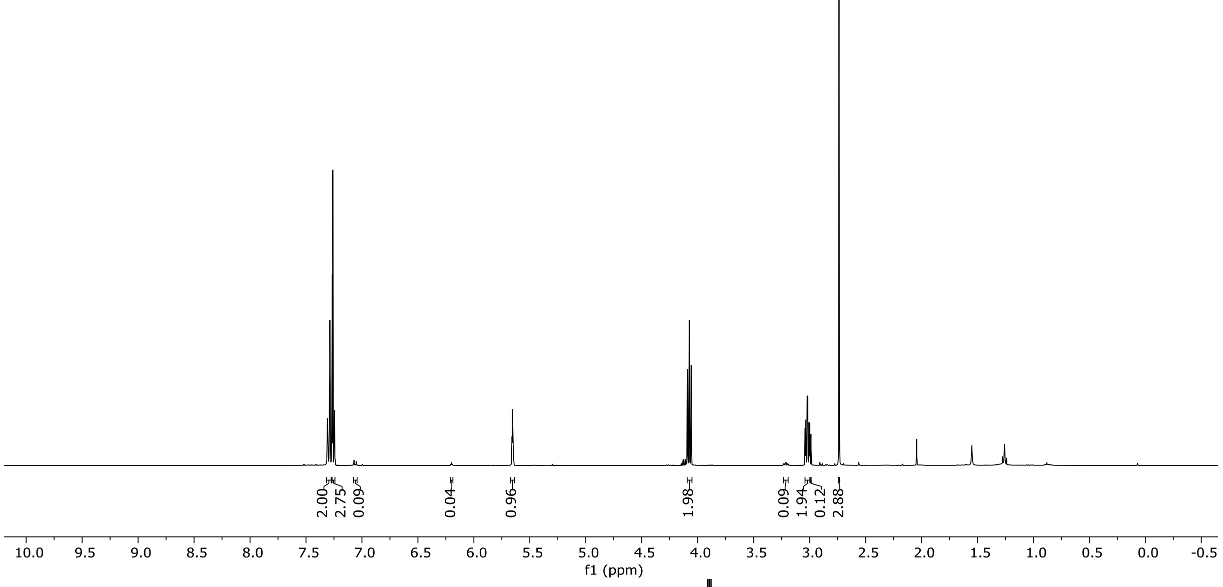

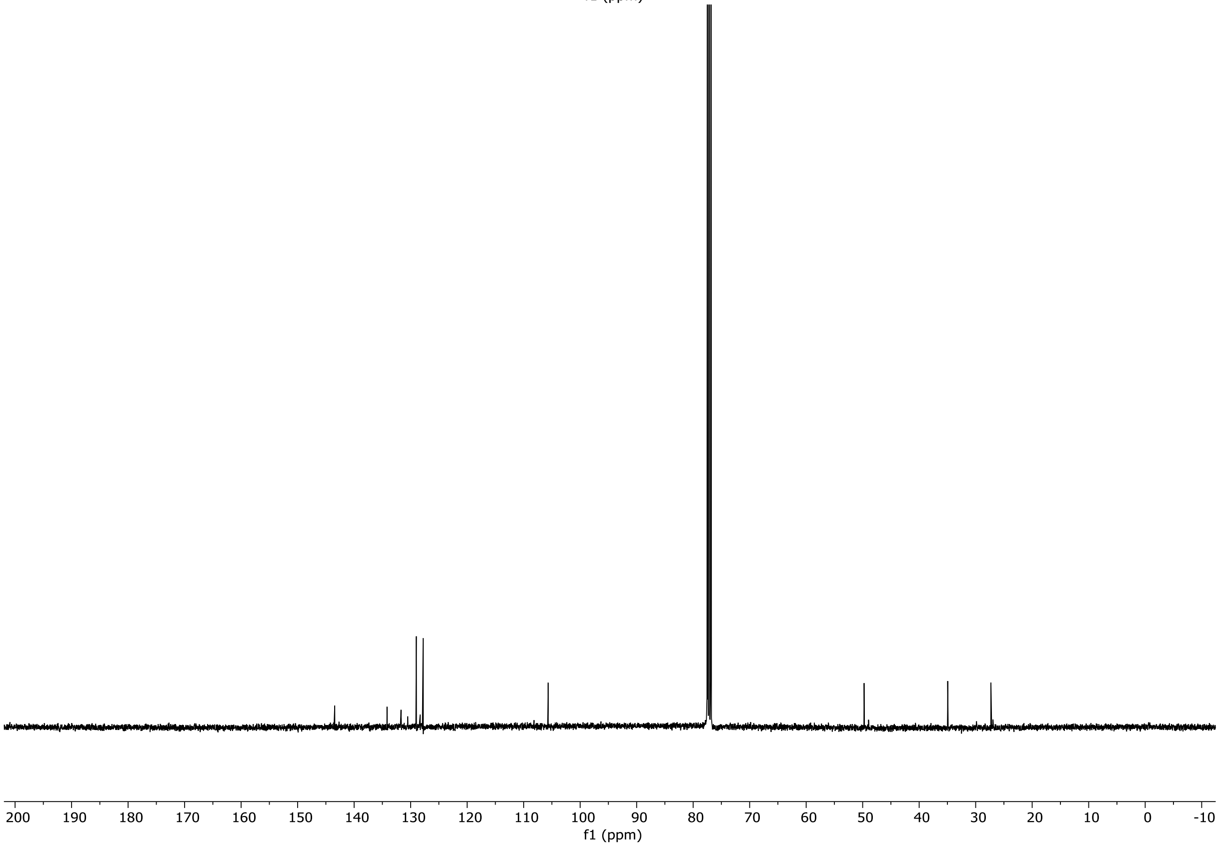

Supplementary Fig 166. ^{1}H (top) and ^{13}C (bottom) NMR spectra of compound **2m**.

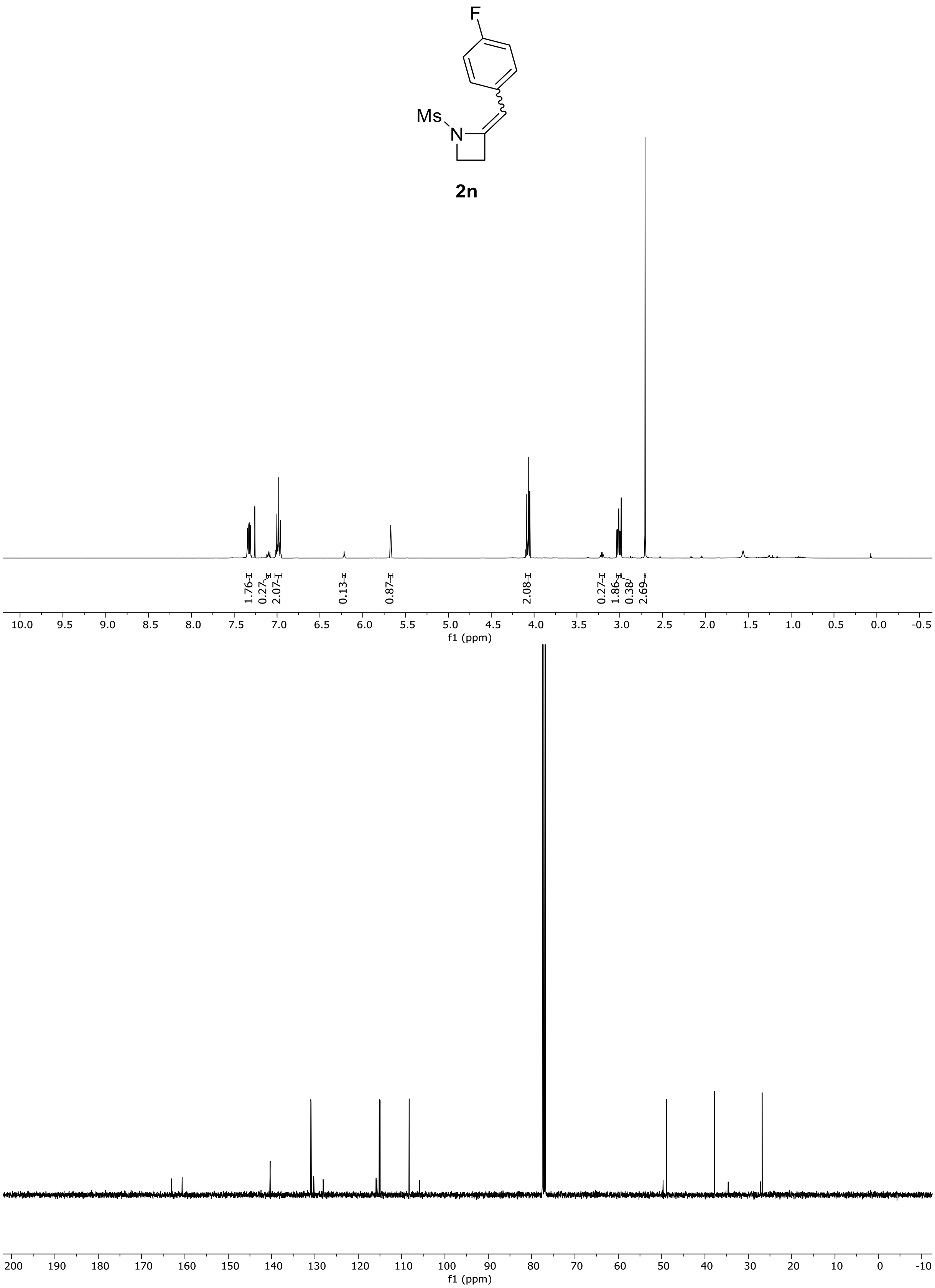

Supplementary Fig 167. ^{1}H (top) and ^{13}C (bottom) NMR spectra of compound **2n**.

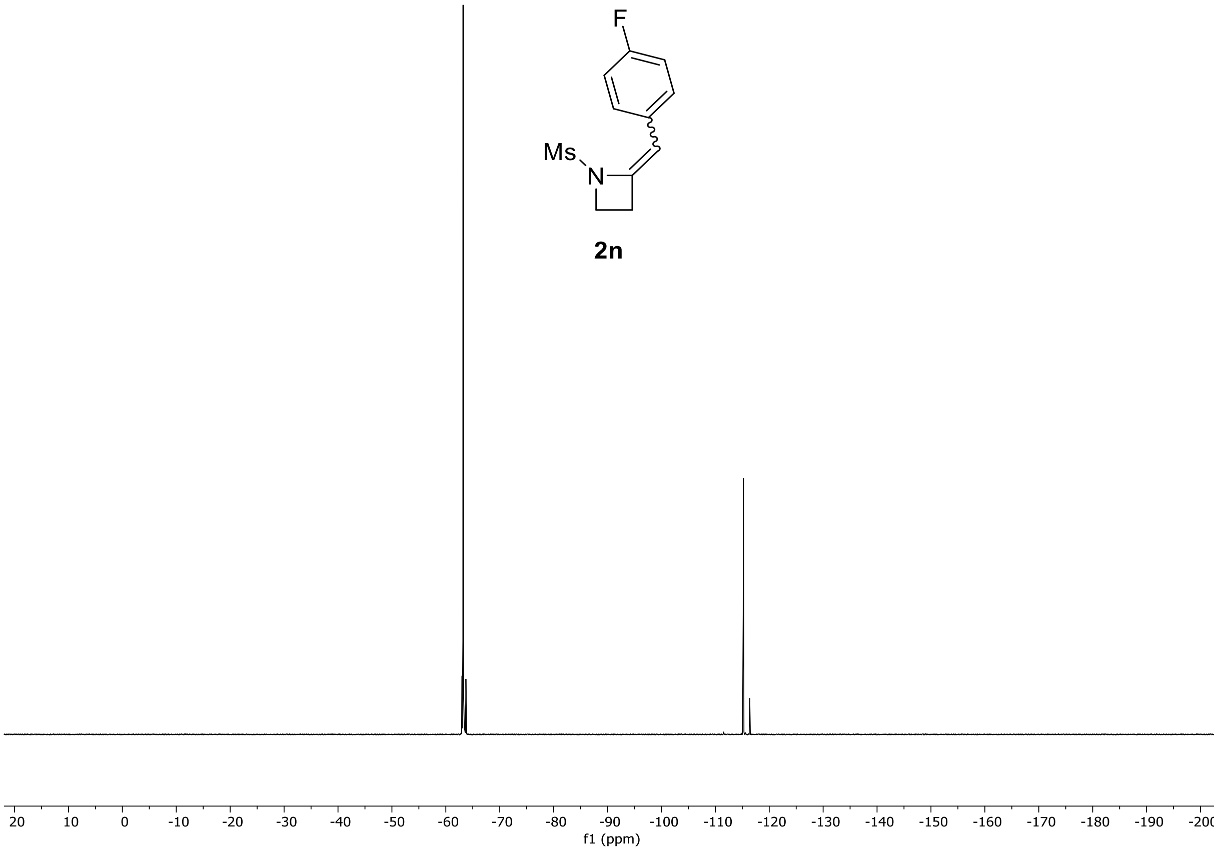

Supplementary Fig 168. ^{19}F NMR spectrum of compound **2n**.

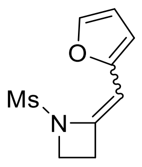

2o

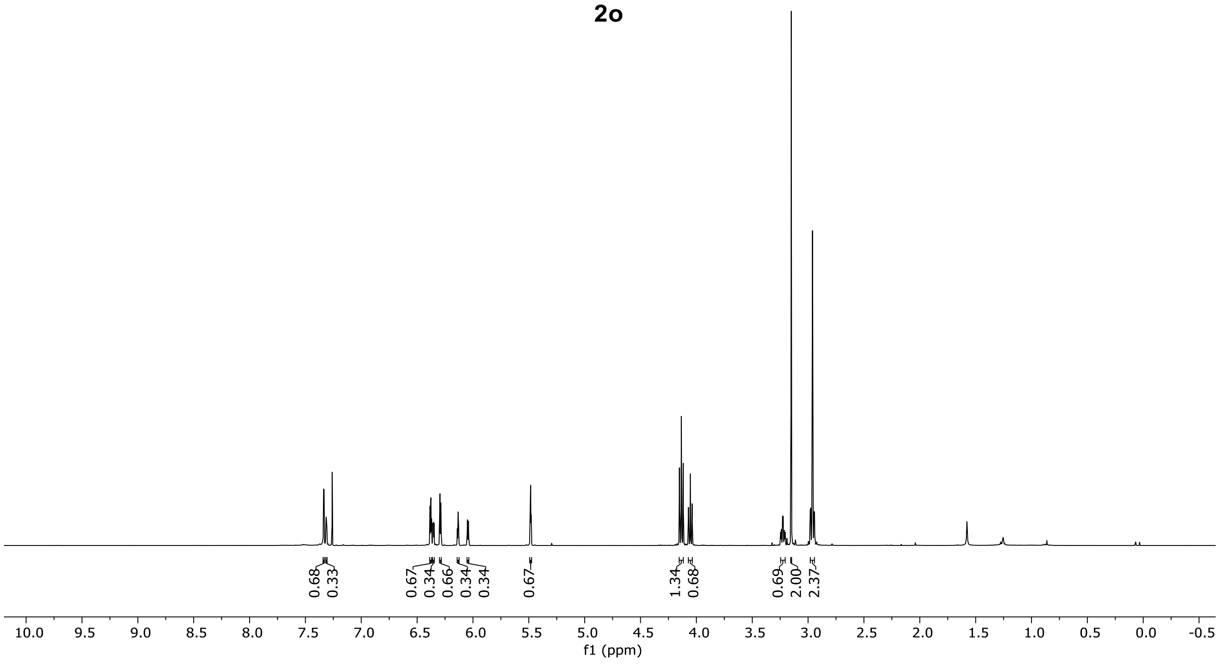

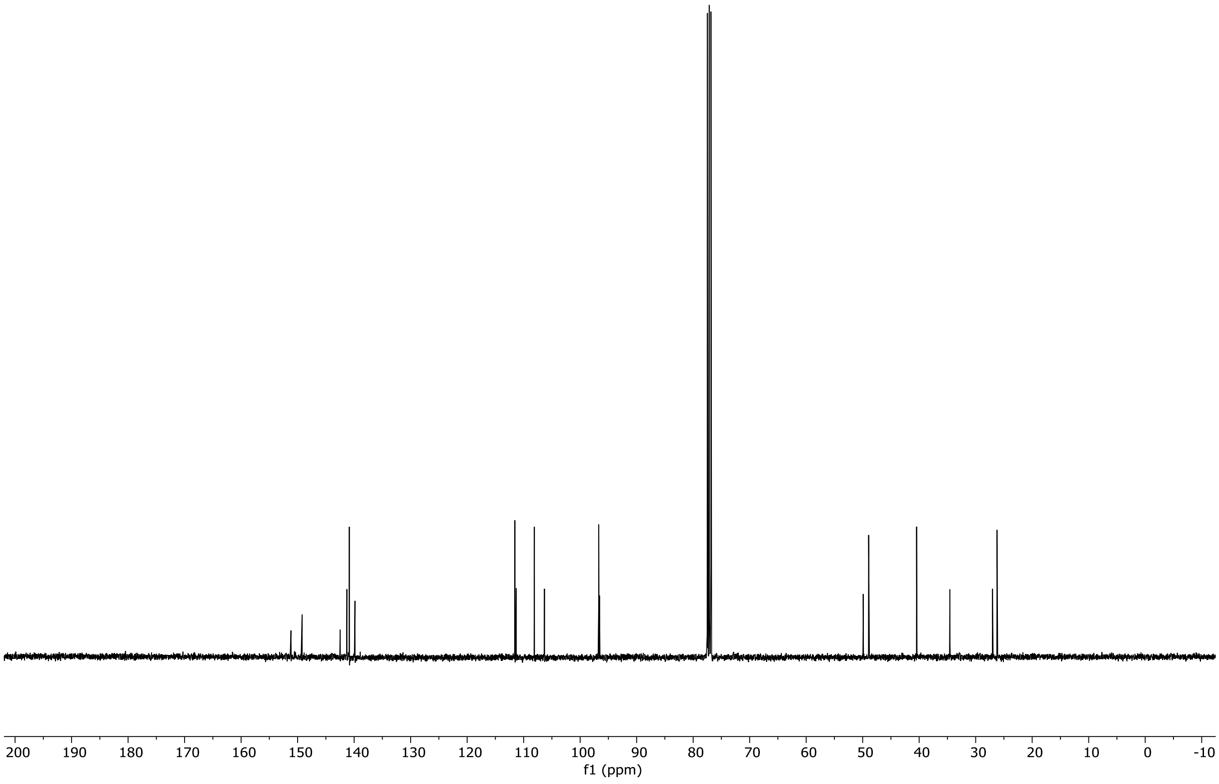

Supplementary Fig 169. ^{1}H (top) and ^{13}C (bottom) NMR spectra of compound **2o**.

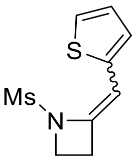

2p

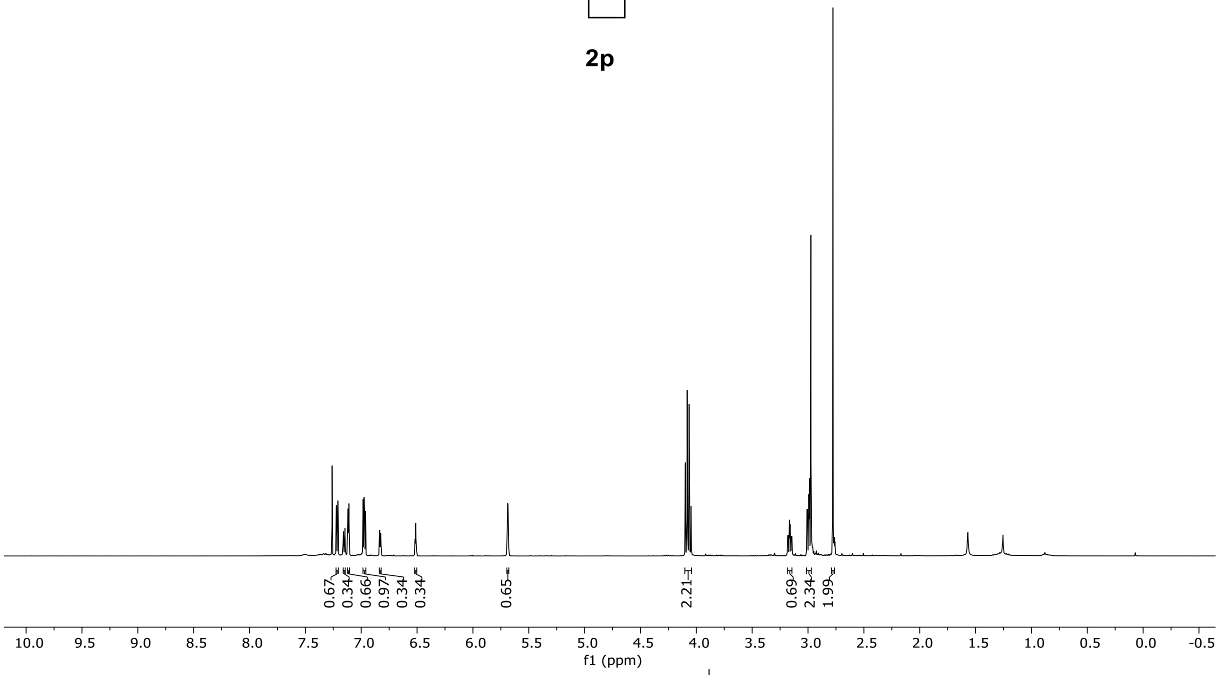

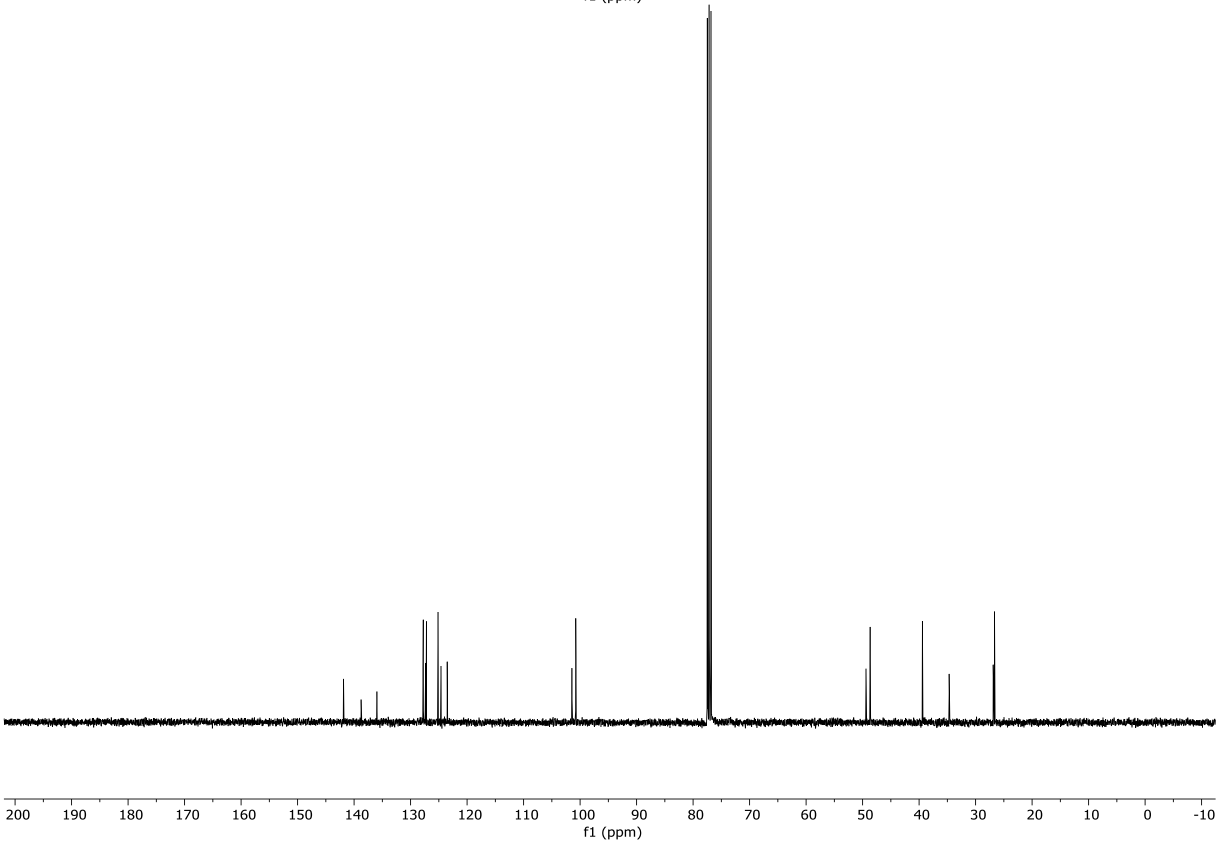

Supplementary Fig 170. ^{1}H (top) and ^{13}C (bottom) NMR spectra of compound **2p**.

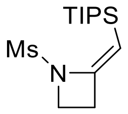

2q

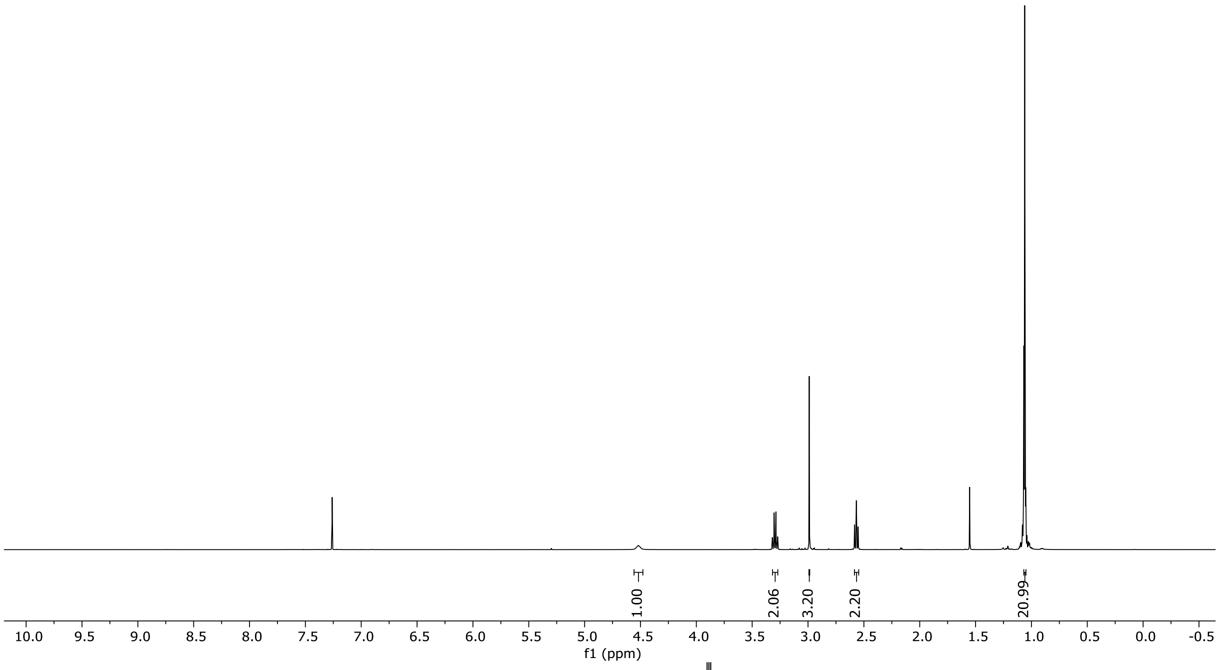

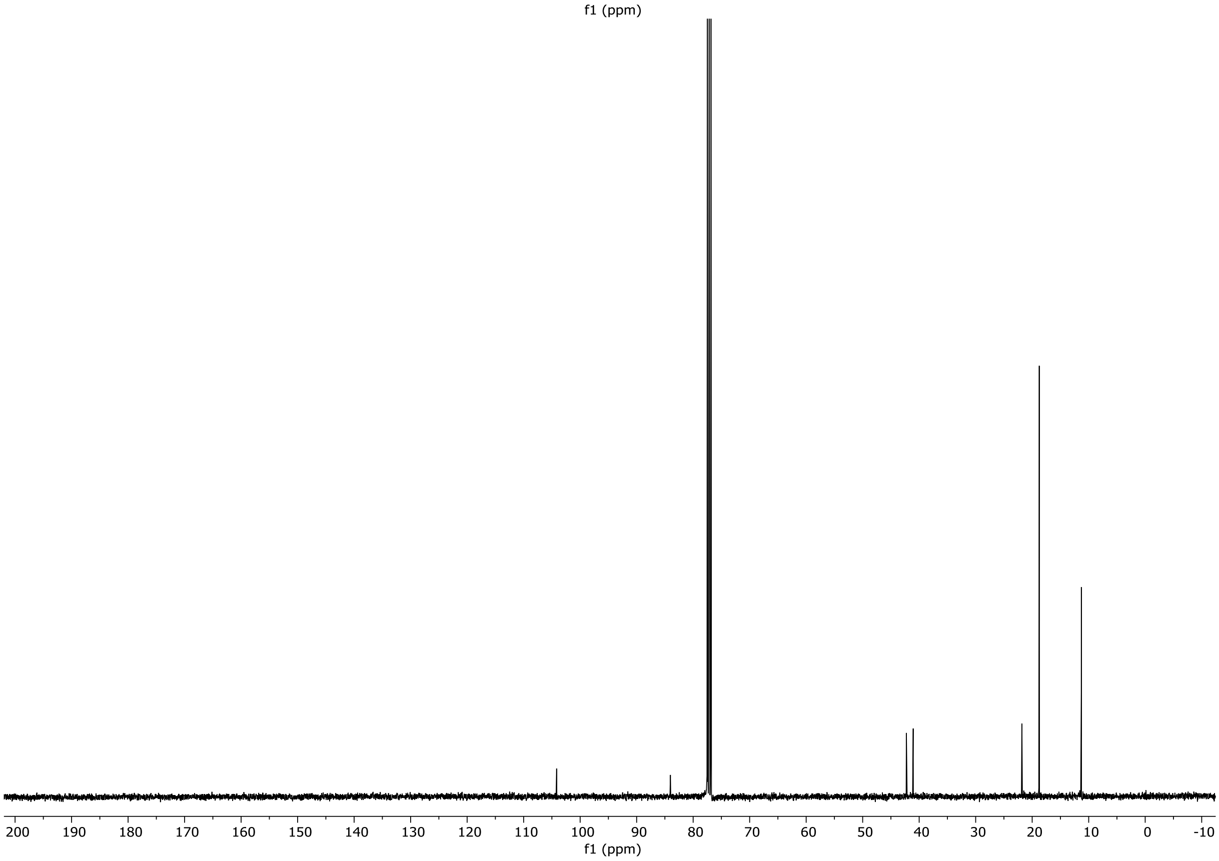

Supplementary Fig 171. ^{1}H (top) and ^{13}C (bottom) NMR spectra of compound **2q**.

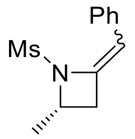

2t

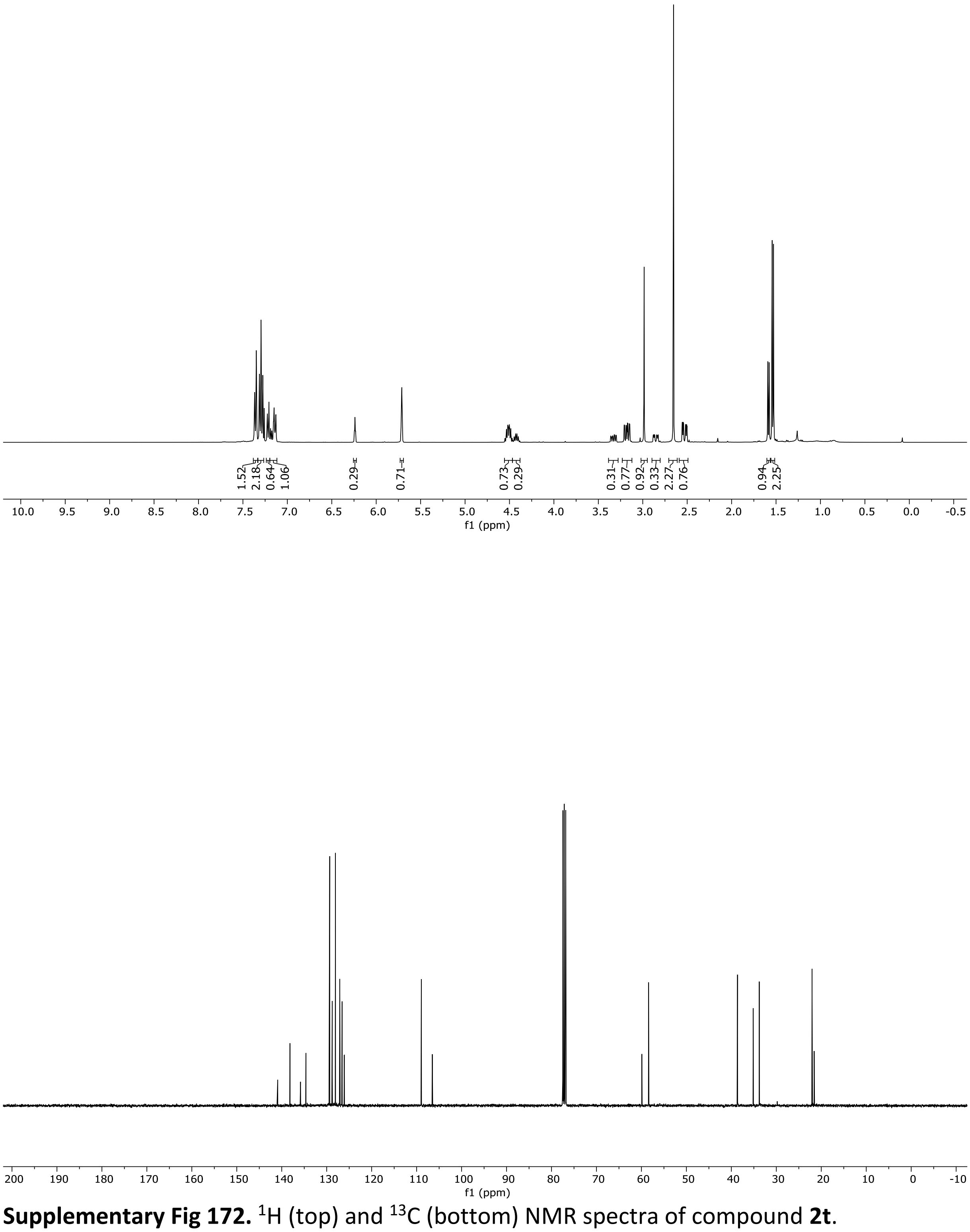

Supplementary Fig 172. ^{1}H (top) and ^{13}C (bottom) NMR spectra of compound **2t**.

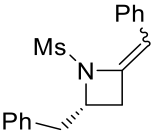

2u

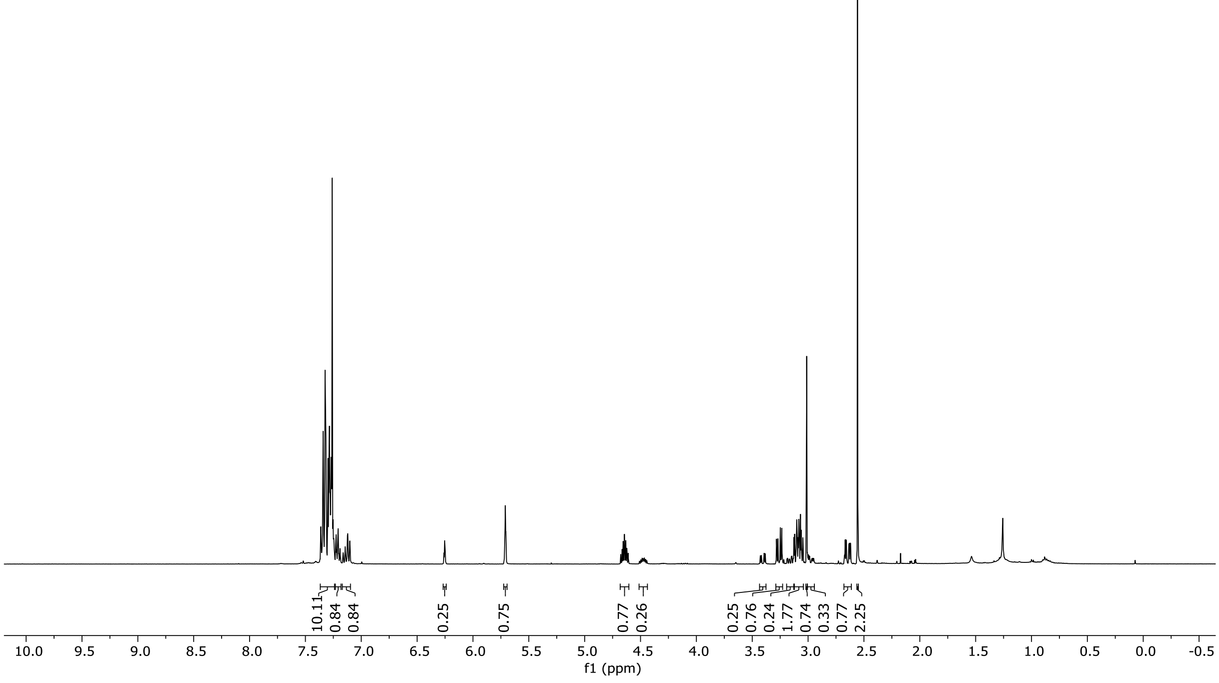

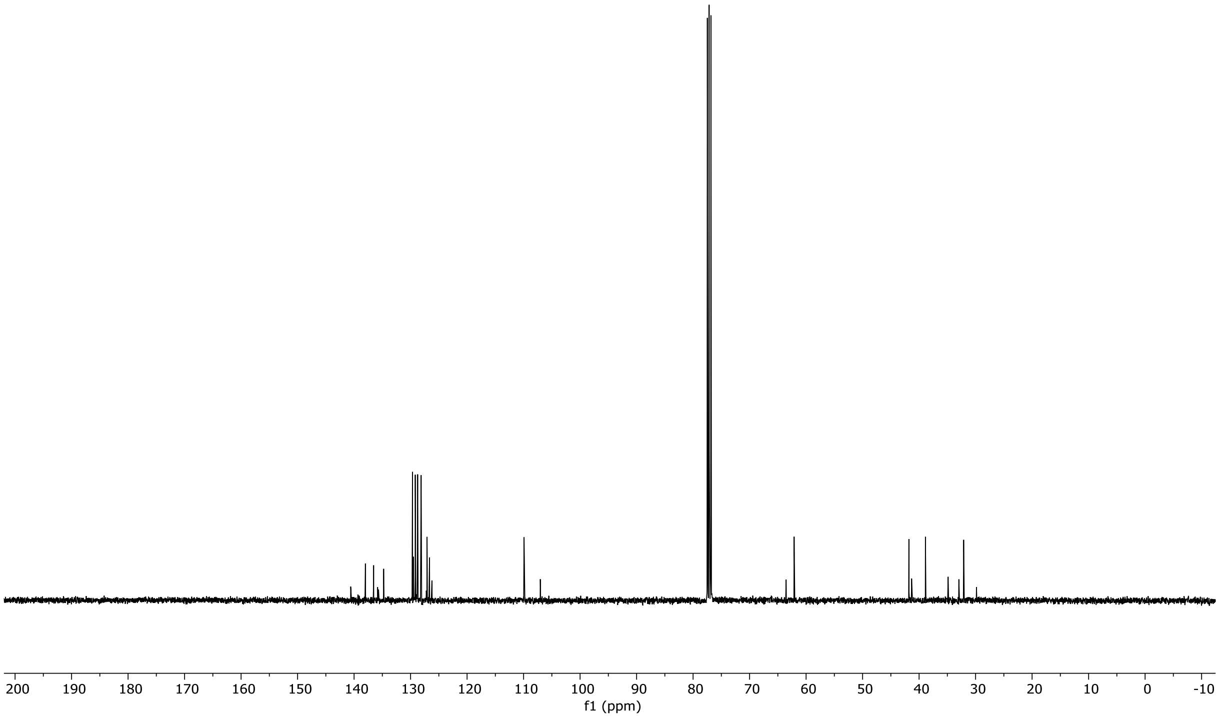

Supplementary Fig 173. ^{1}H (top) and ^{13}C (bottom) NMR spectra of compound **2u**.

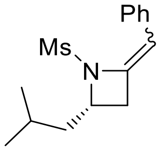

2v

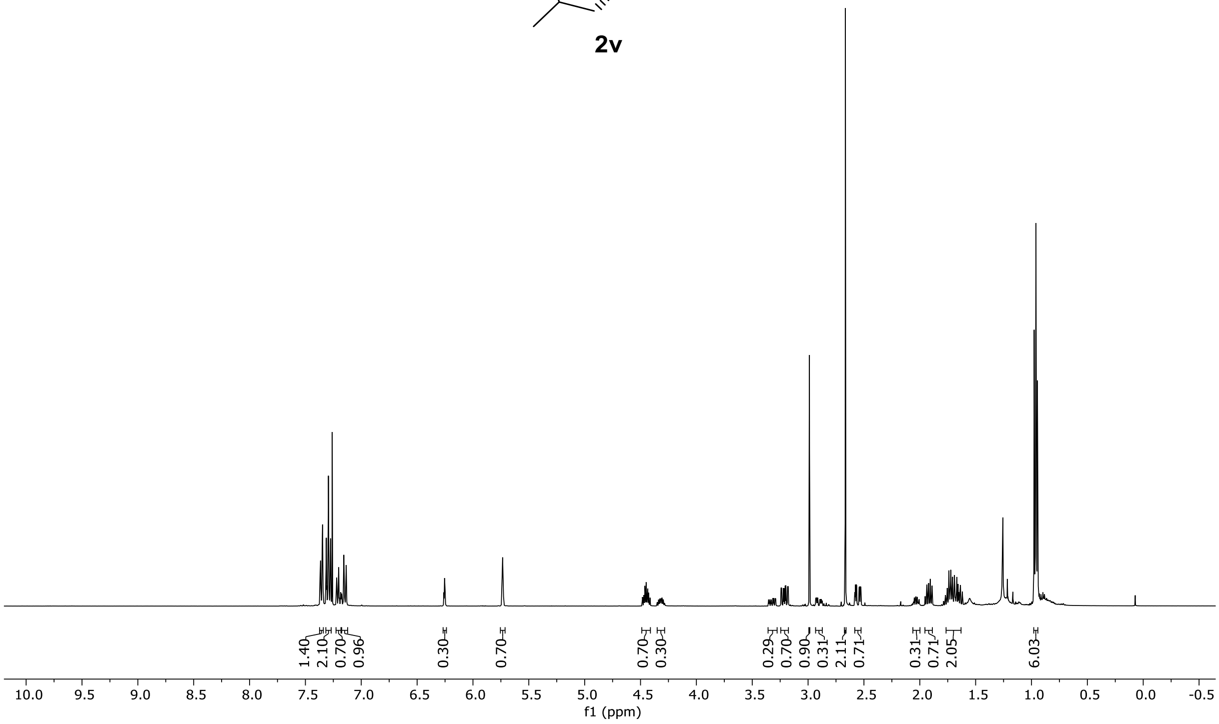

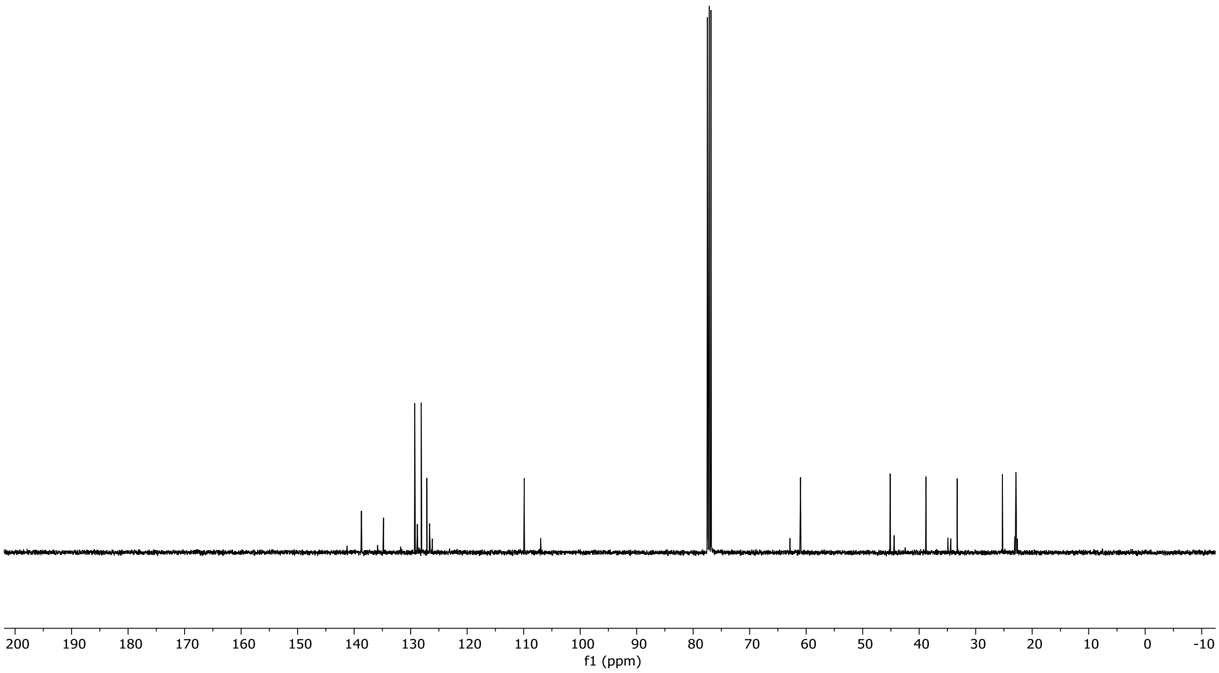

Supplementary Fig 174. ^{1}H (top) and ^{13}C (bottom) NMR spectra of compound **2v**.

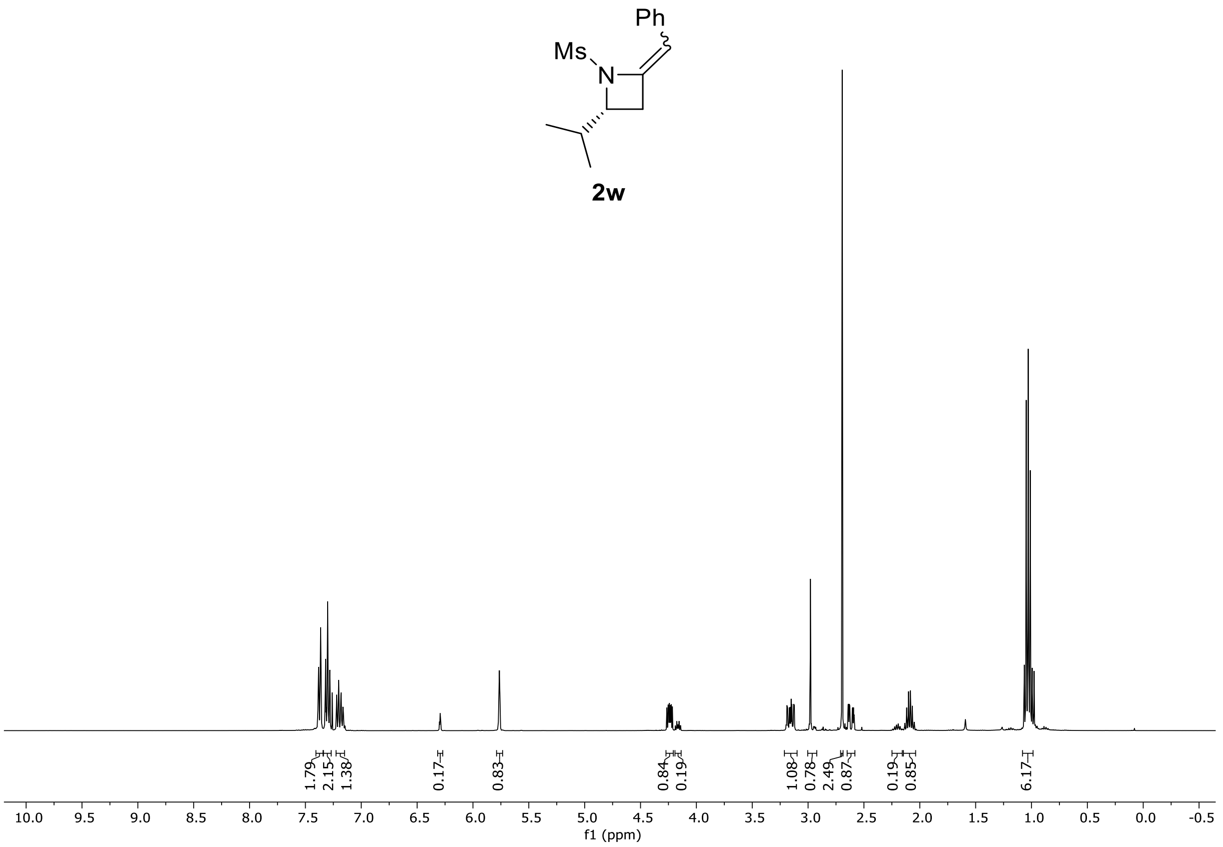

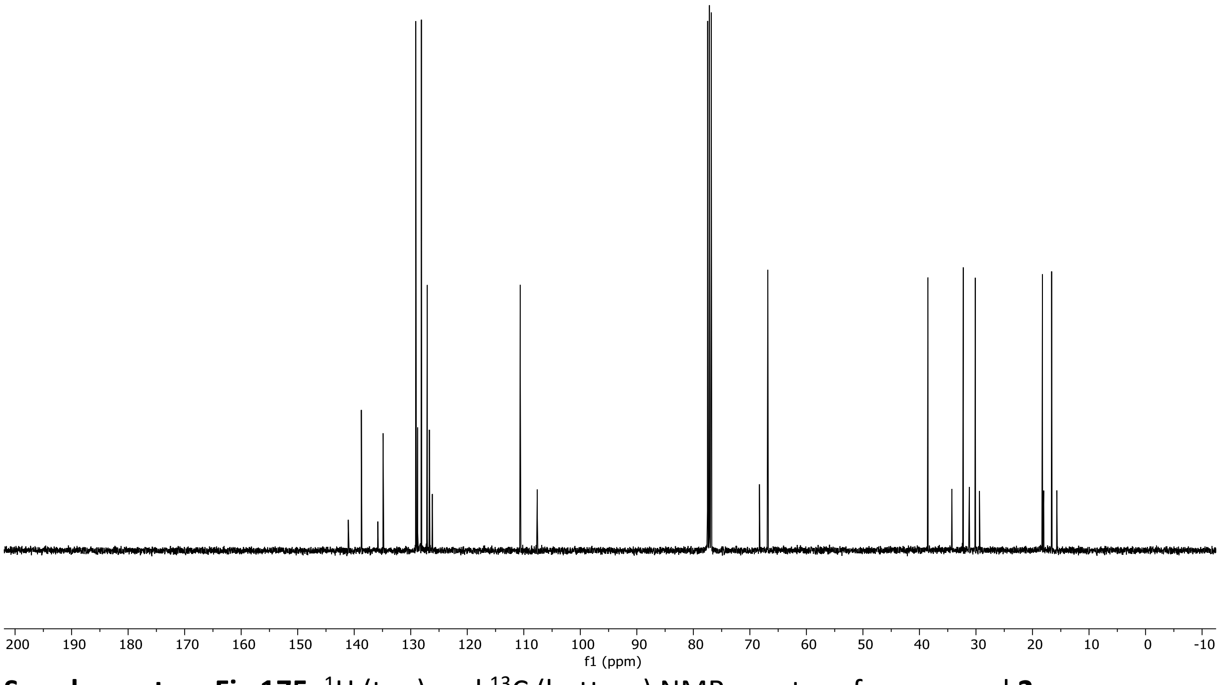

Supplementary Fig 175. ^{1}H (top) and ^{13}C (bottom) NMR spectra of compound **2w**.

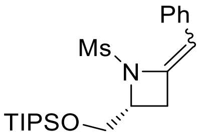

2x

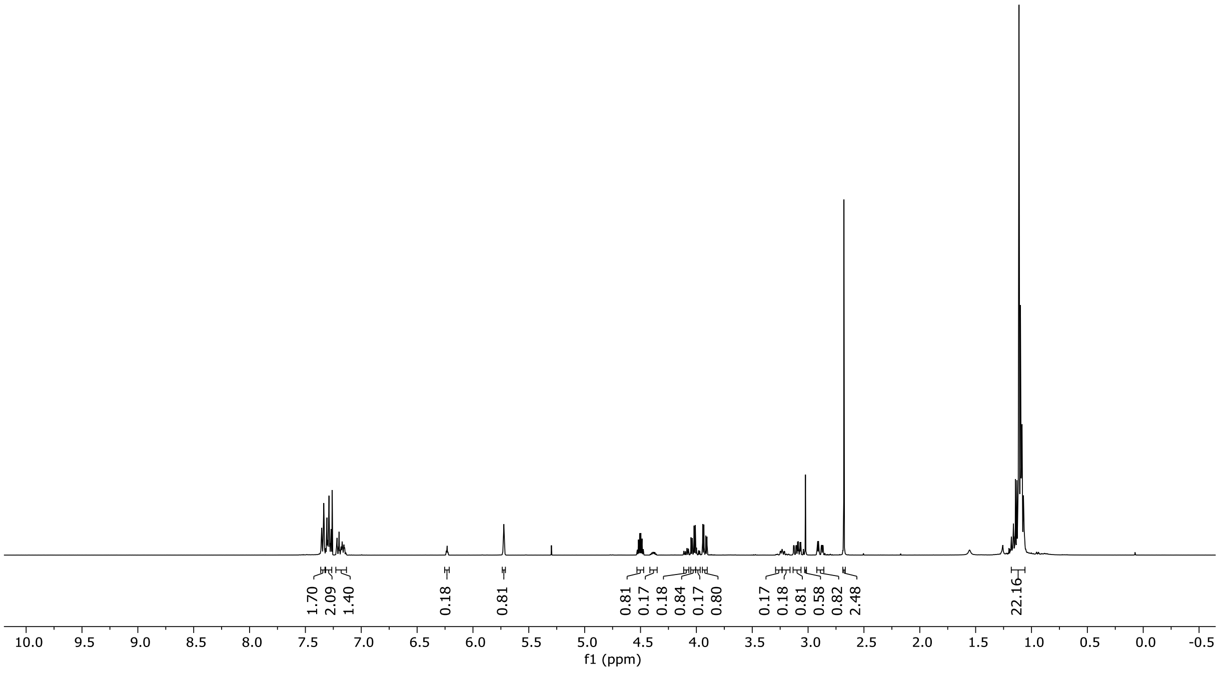

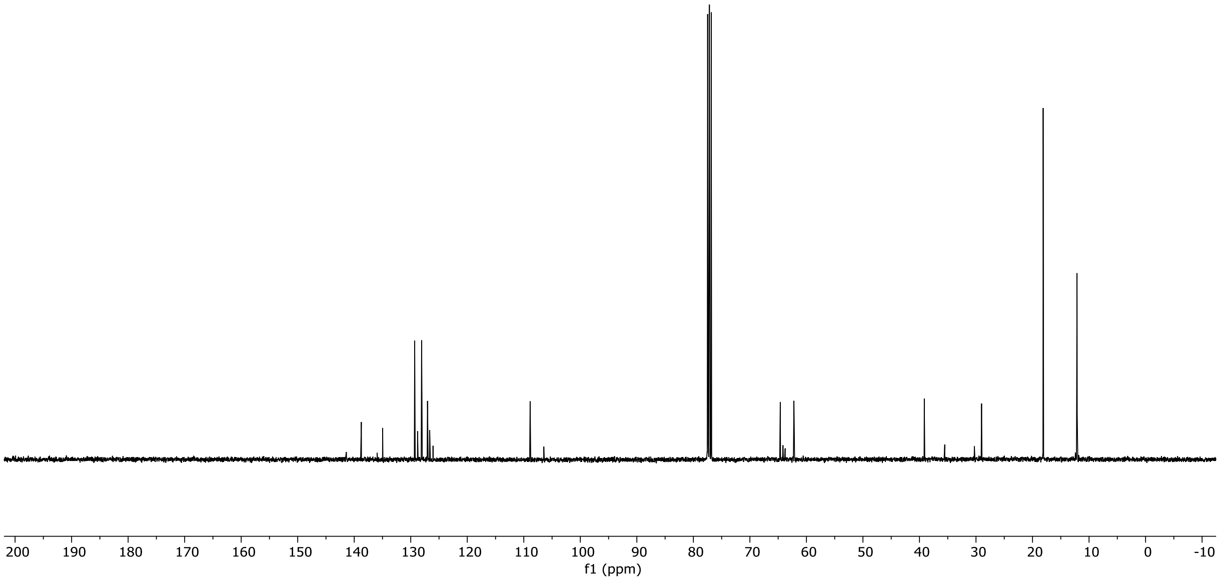

Supplementary Fig 176. ^{1}H (top) and ^{13}C (bottom) NMR spectra of compound **2x**.

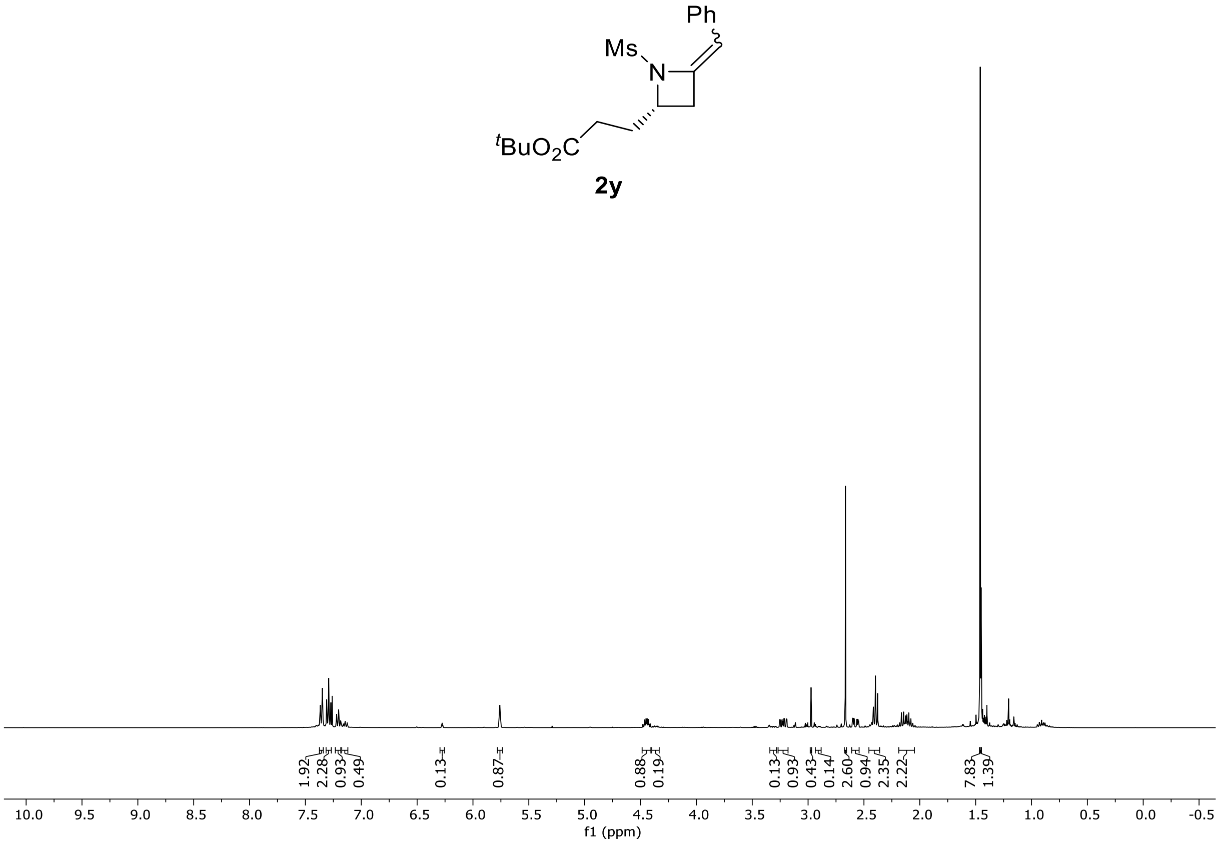

2y

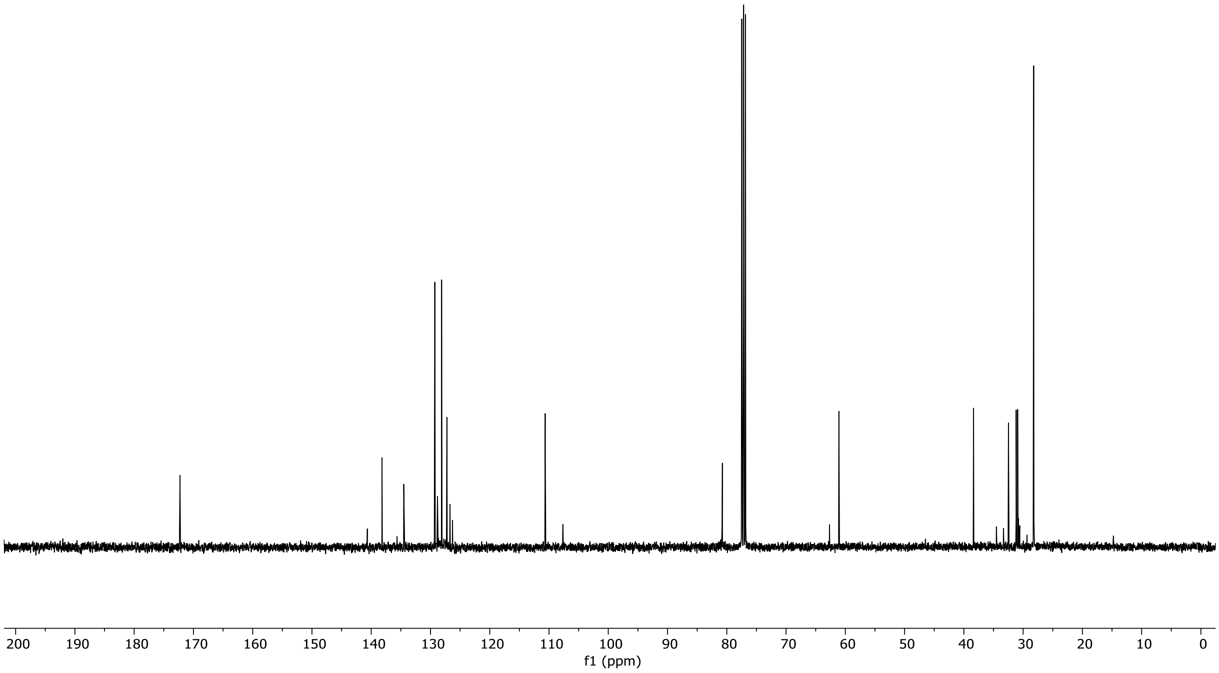

Supplementary Fig 177. ^{1}H (top) and ^{13}C (bottom) NMR spectra of compound **2y**.

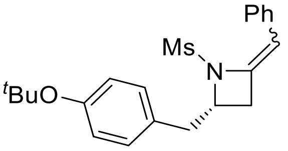

2z

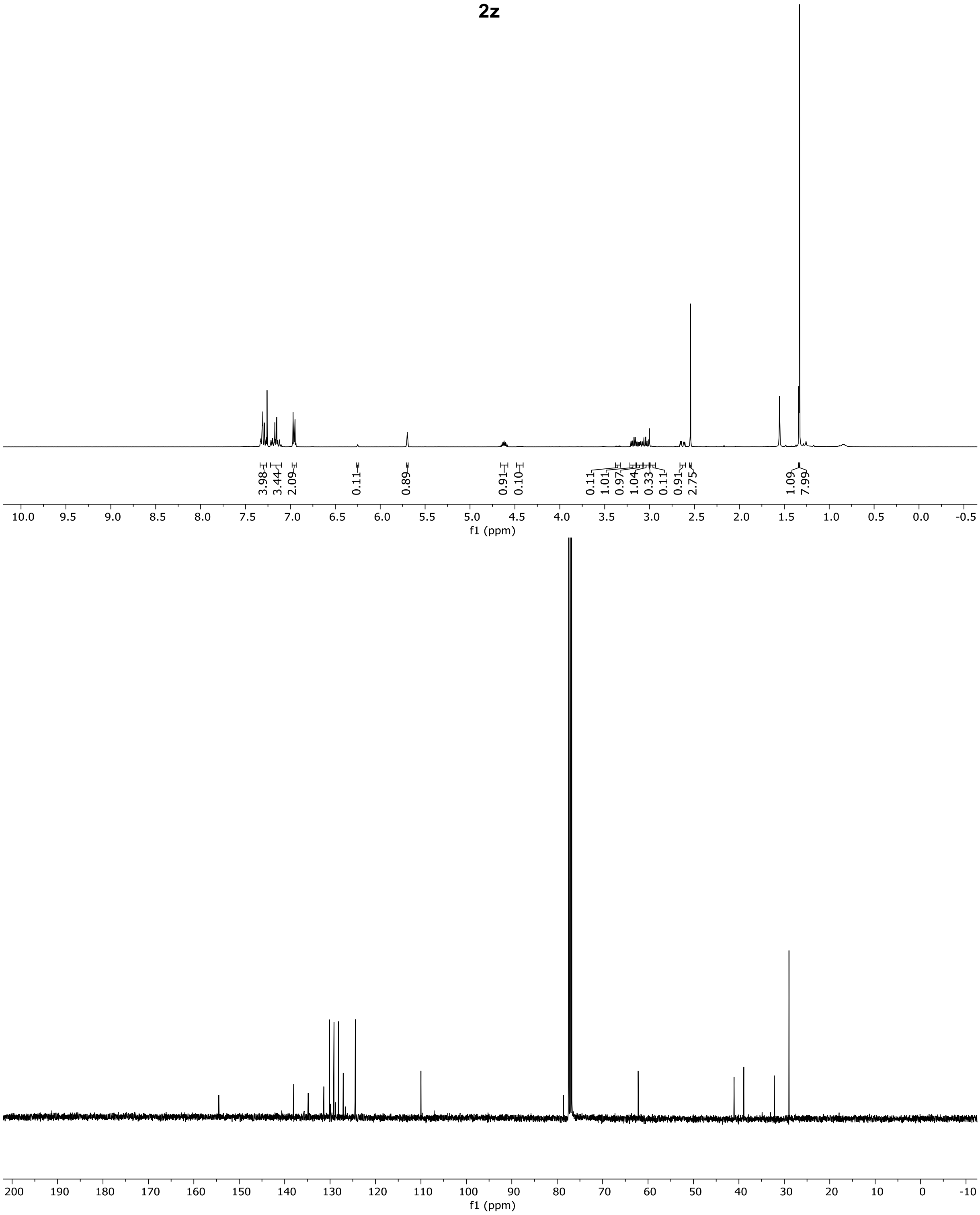

Supplementary Fig 178. ^{1}H (top) and ^{13}C (bottom) NMR spectra of compound **2z**.

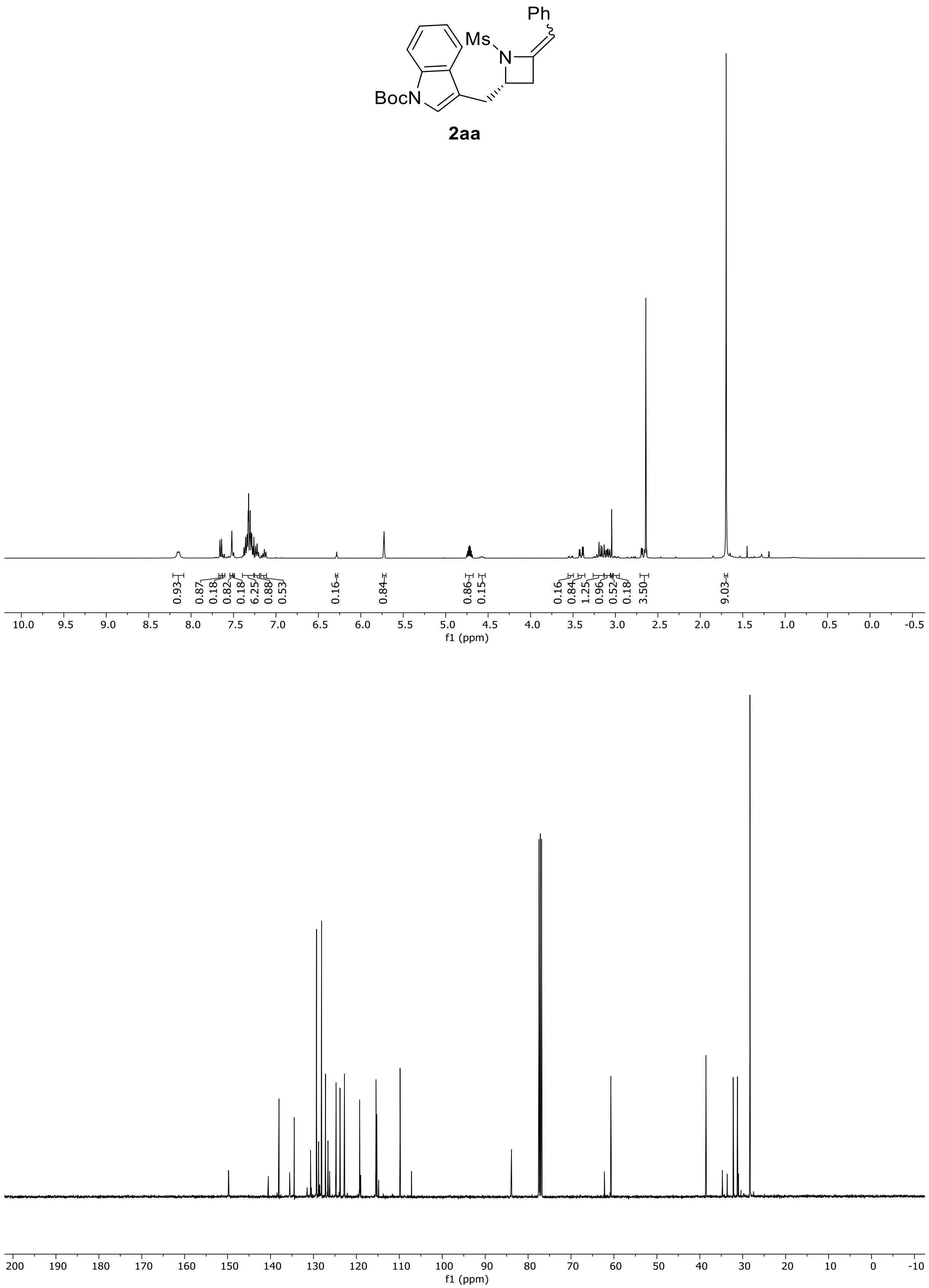

Supplementary Fig 179. ^{1}H (top) and ^{13}C (bottom) NMR spectra of compound **2aa**.

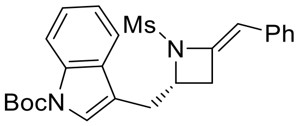

(*E*)-2aa

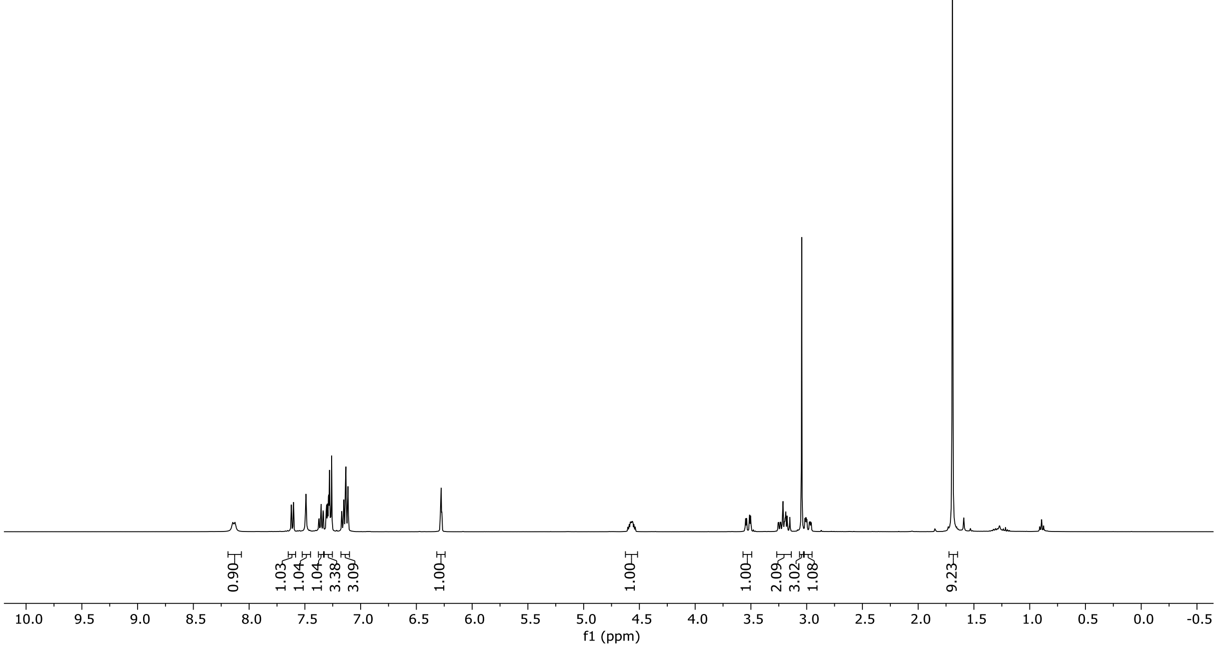

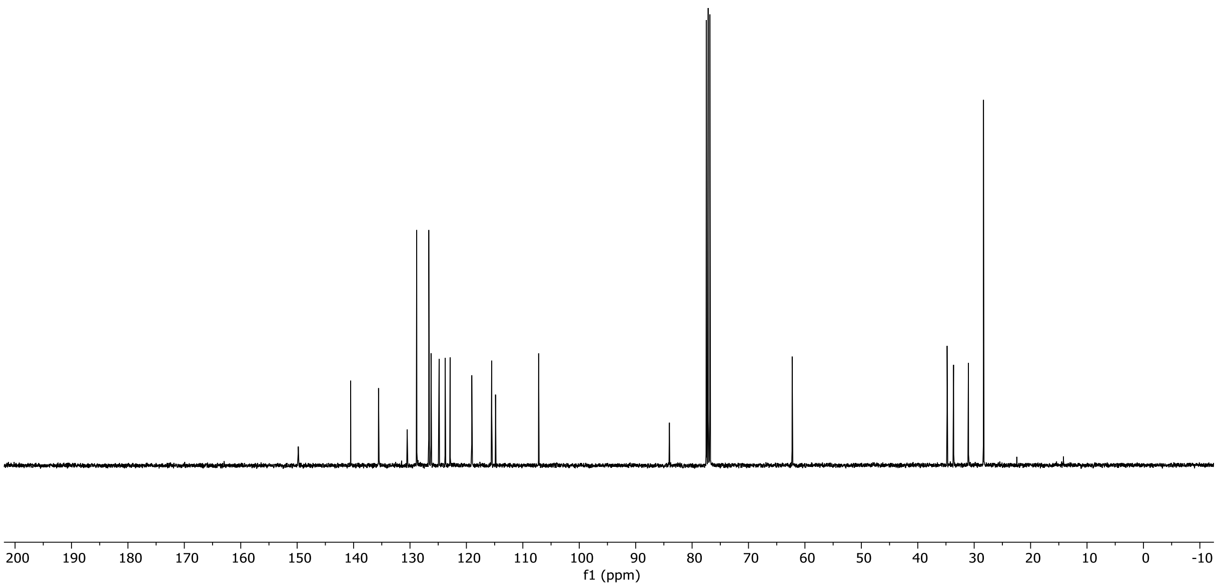

Supplementary Fig 180. ^{1}H (top) and ^{13}C (bottom) NMR spectra of compound **(*E*)-2aa**.

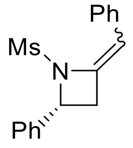

2ab

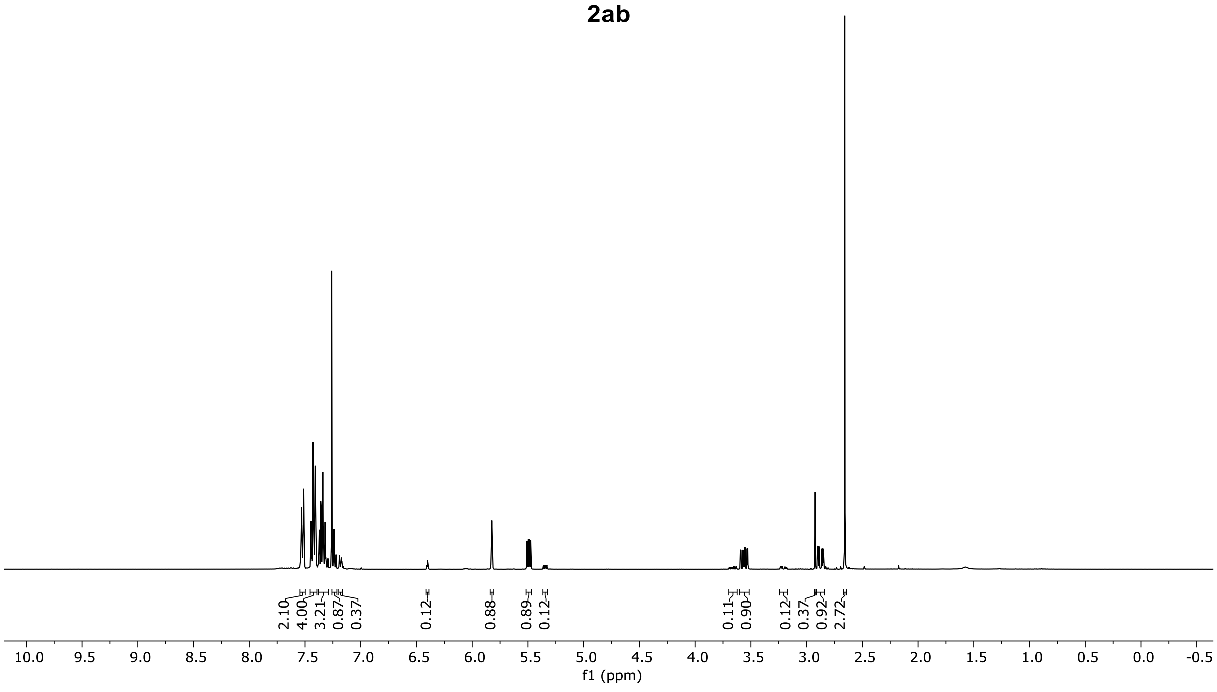

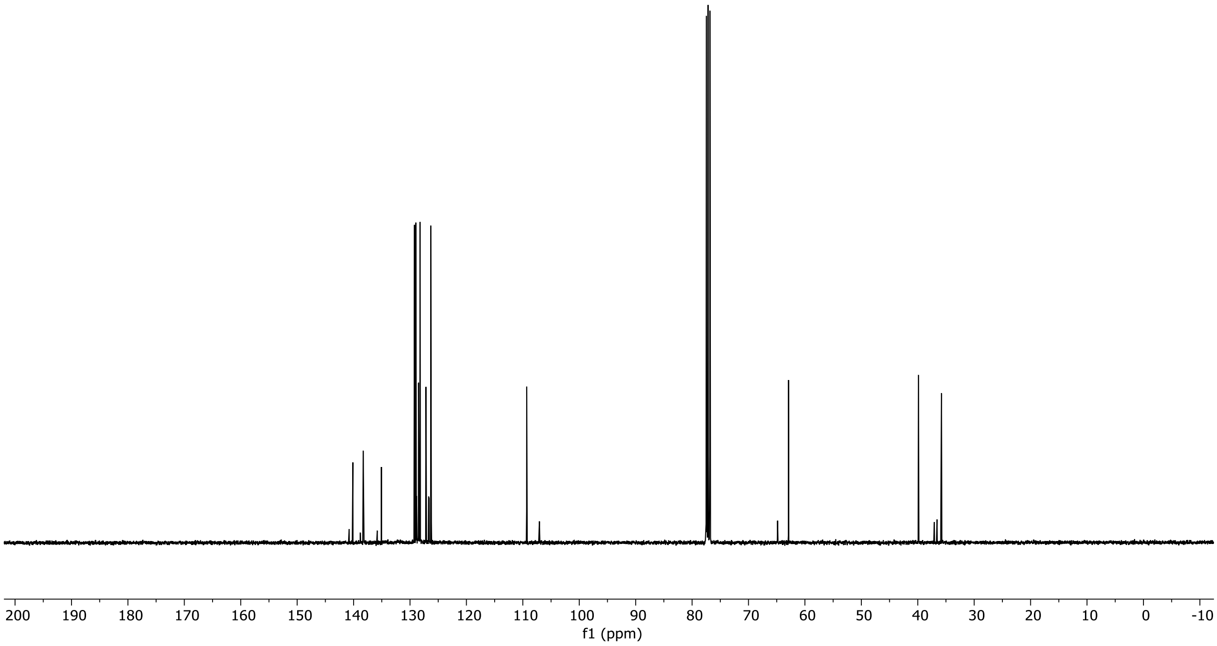

Supplementary Fig 181. ^{1}H (top) and ^{13}C (bottom) NMR spectra of compound **2ab**.

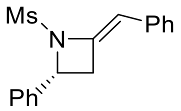

(*E*)-2ab

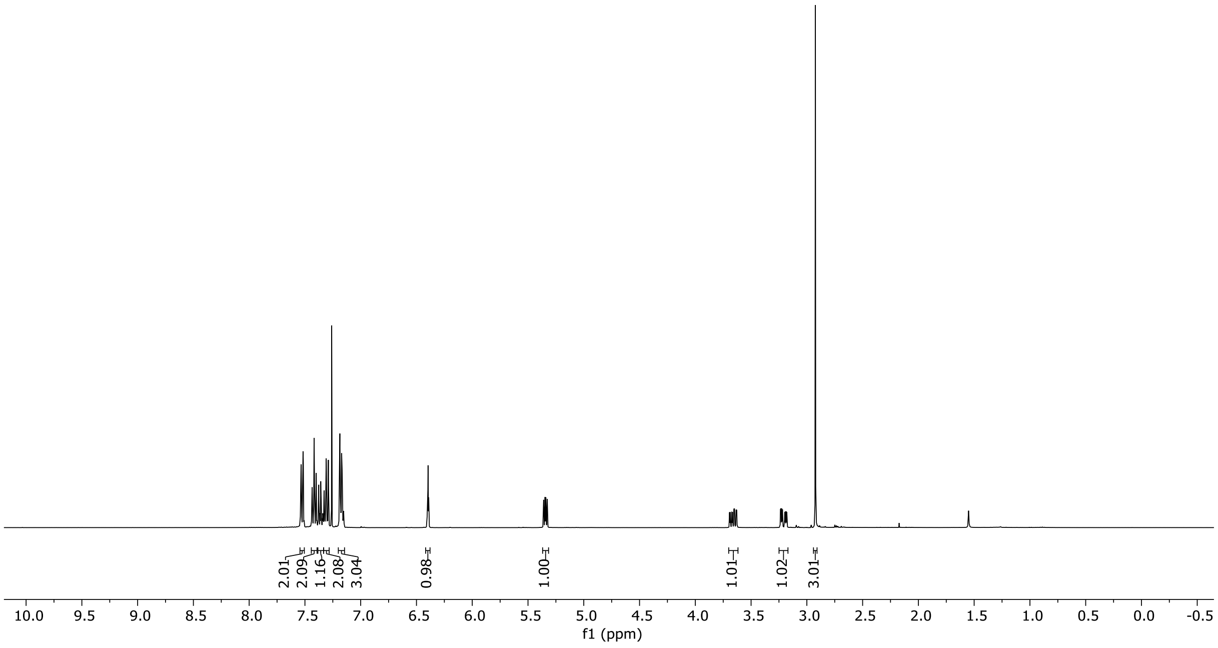

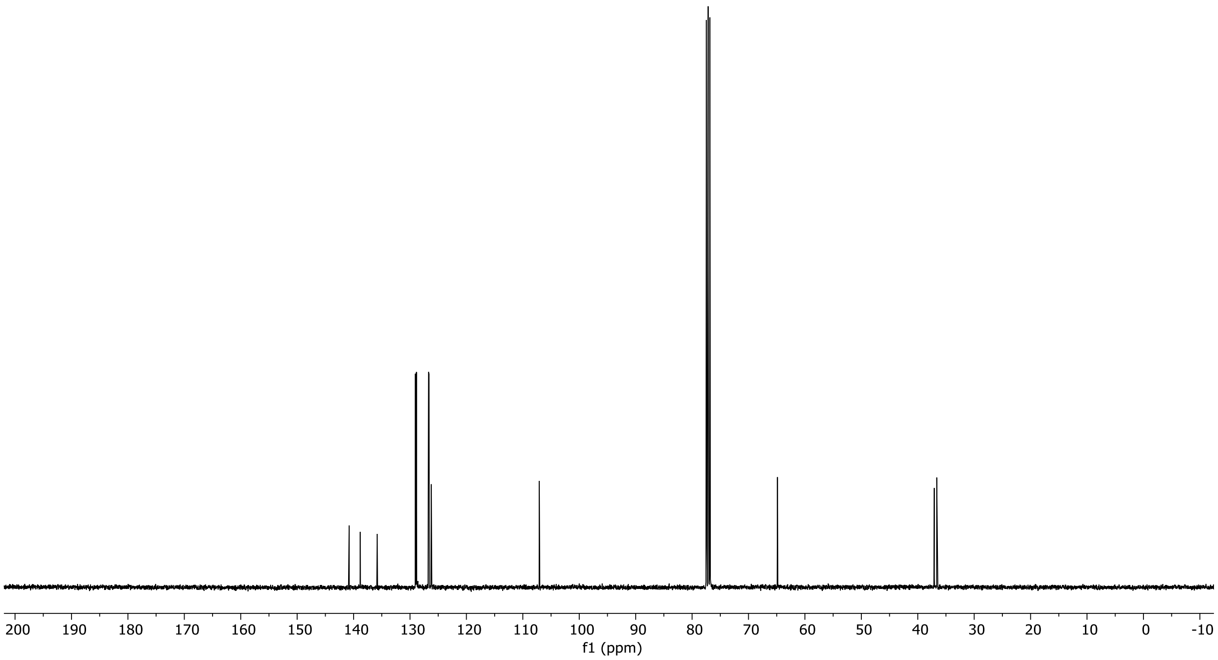

Supplementary Fig 182. ^{1}H (top) and ^{13}C (bottom) NMR spectra of compound **(*E*)-2ab**.

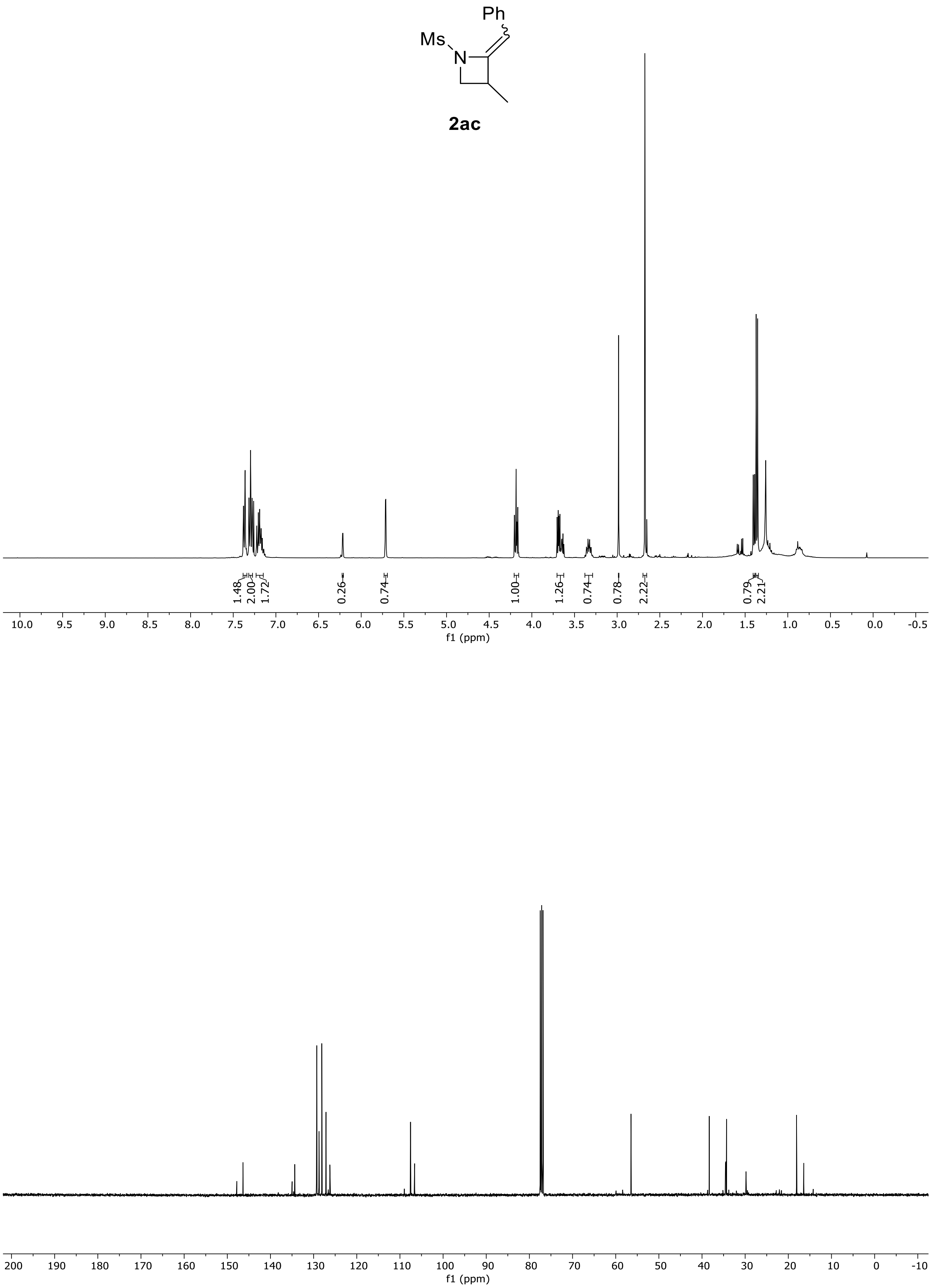

Supplementary Fig 183. ^{1}H (top) and ^{13}C (bottom) NMR spectra of compound **2ac**.

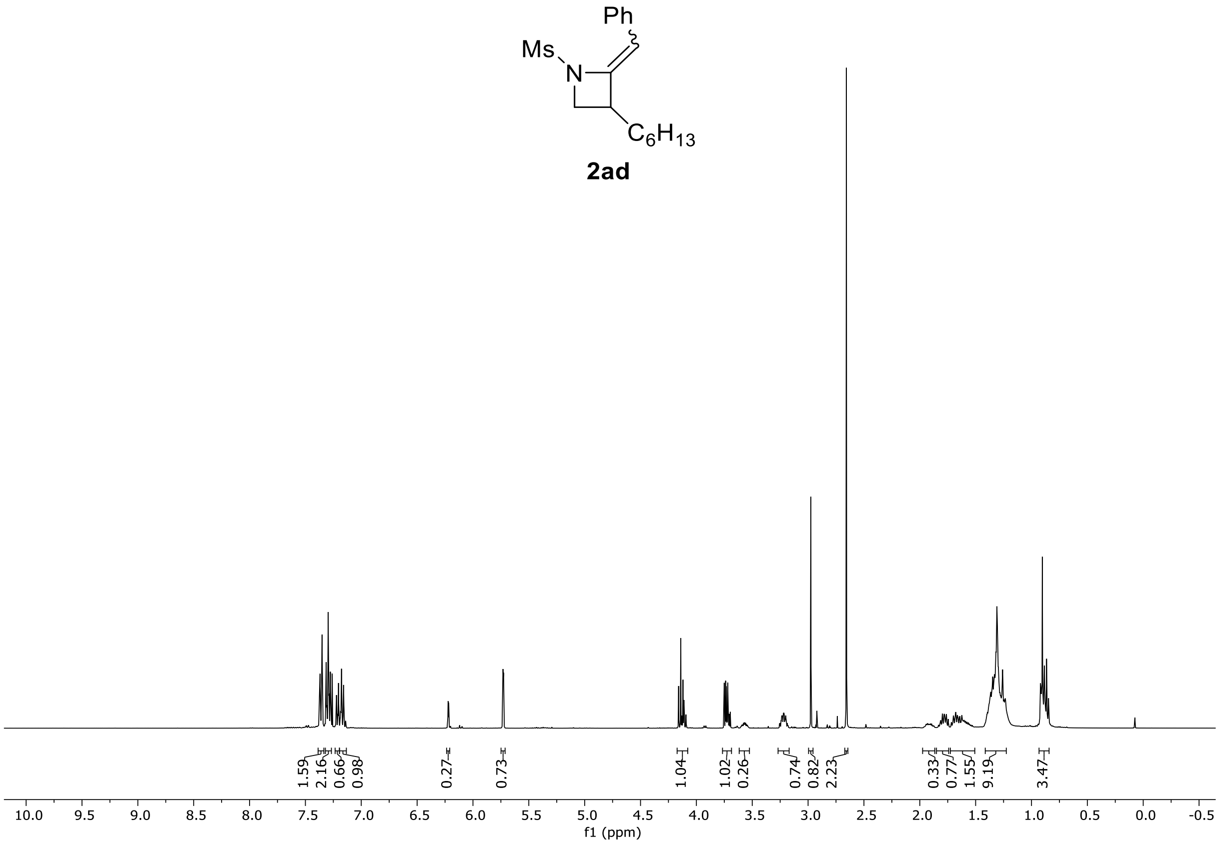

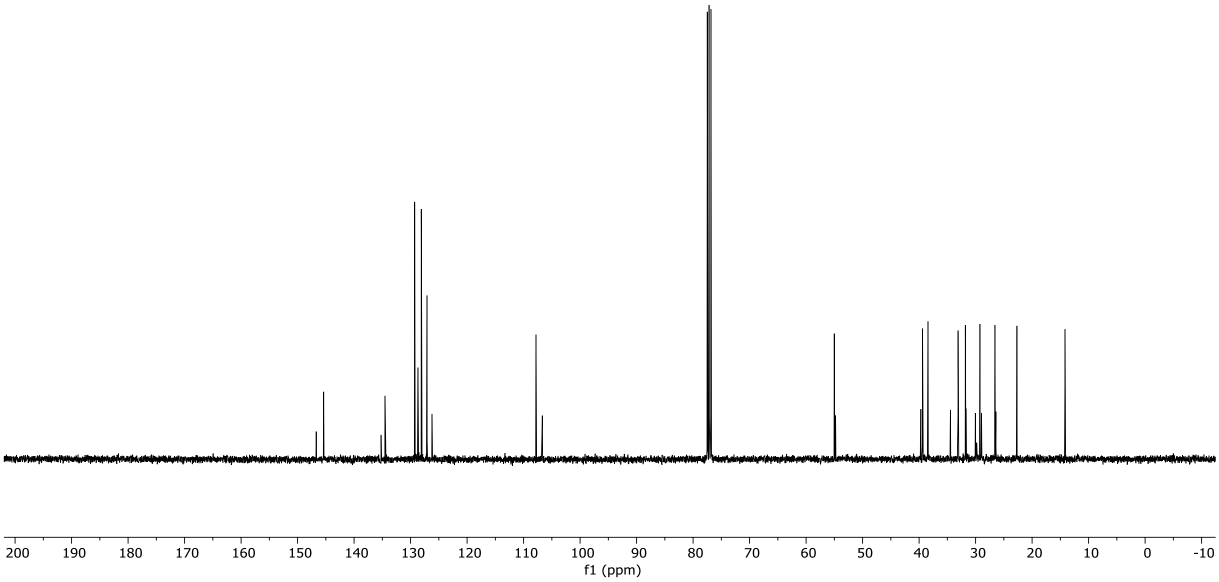

Supplementary Fig 184. ^{1}H (top) and ^{13}C (bottom) NMR spectra of compound **2ad**.

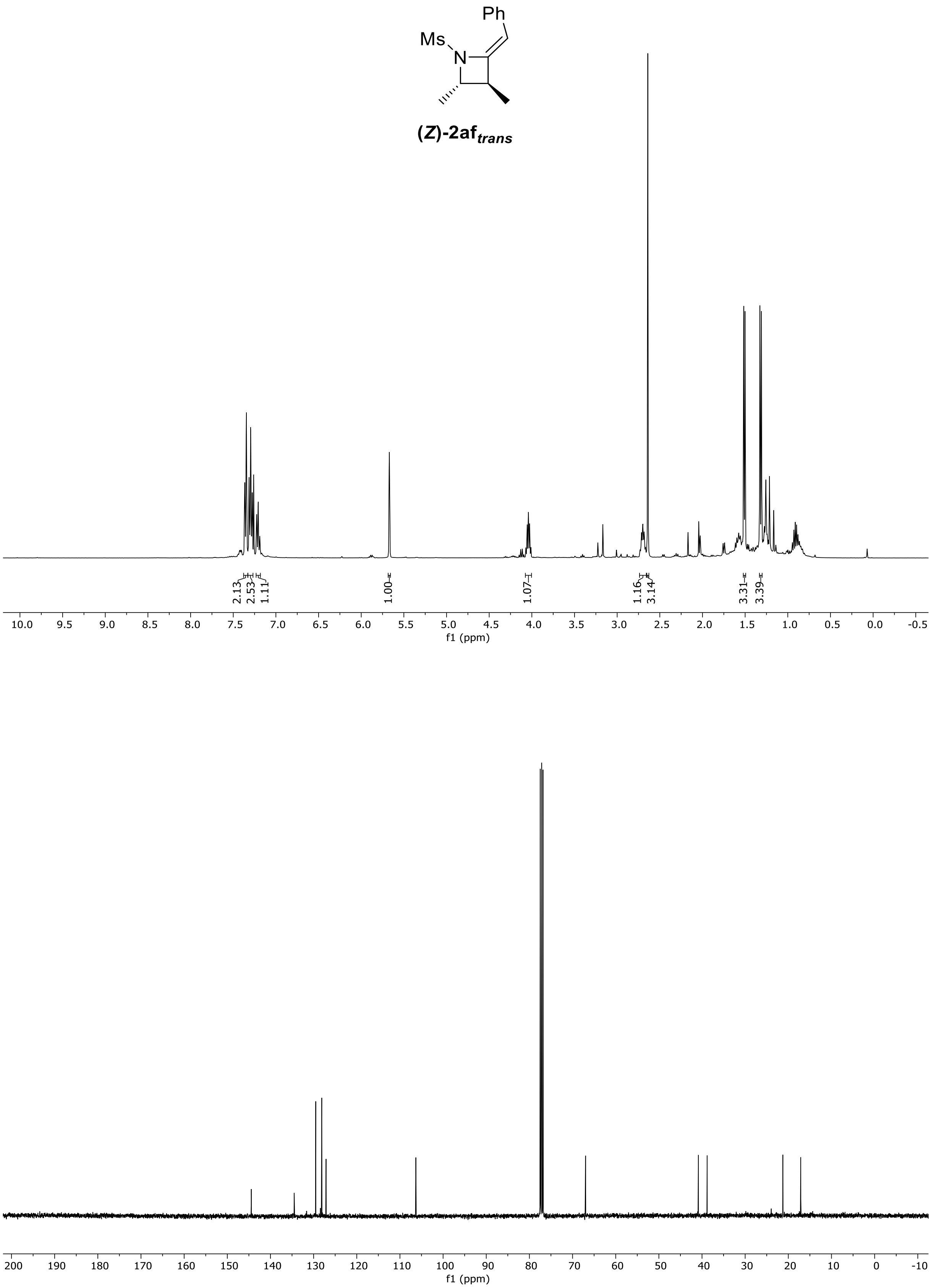

Supplementary Fig 185. ^{1}H (top) and ^{13}C (bottom) NMR spectra of compound **(*Z*)-2af$_{trans}$**.

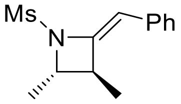

(*E*)-2af*trans*

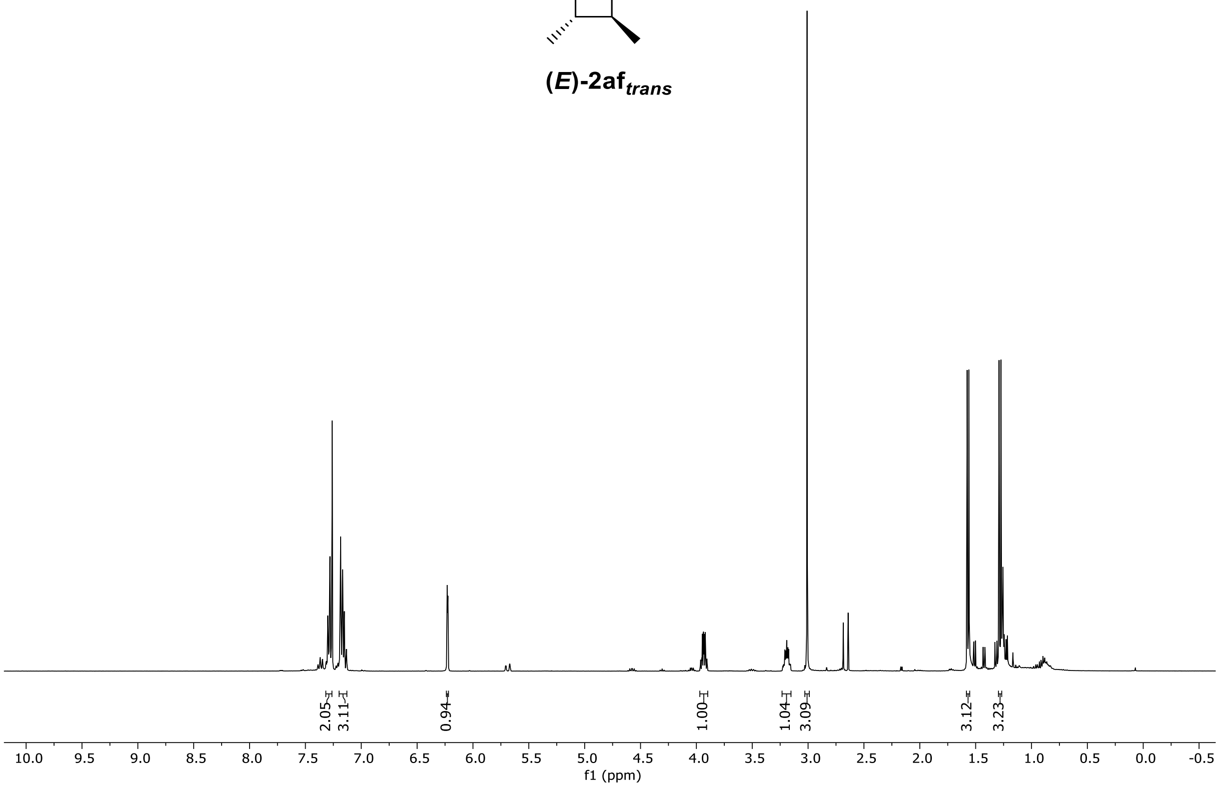

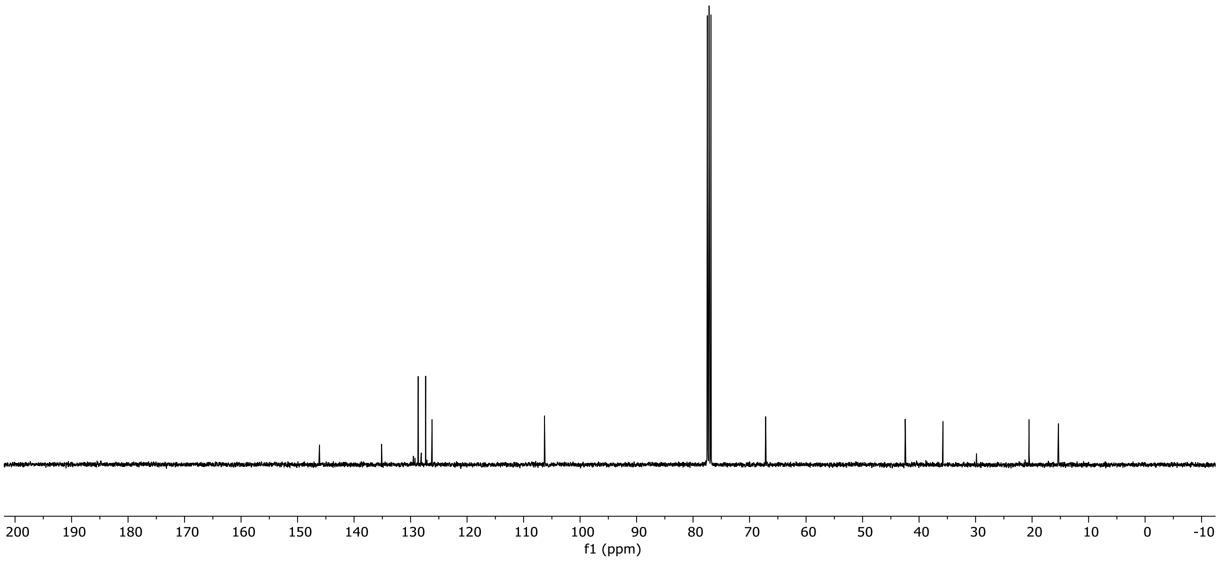

Supplementary Fig 186. ^{1}H (top) and ^{13}C (bottom) NMR spectra of compound **(*E*)-2af$_{trans}$**.

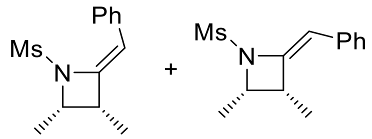

(*Z*)-2af*cis* **(*E*)-2af*cis***

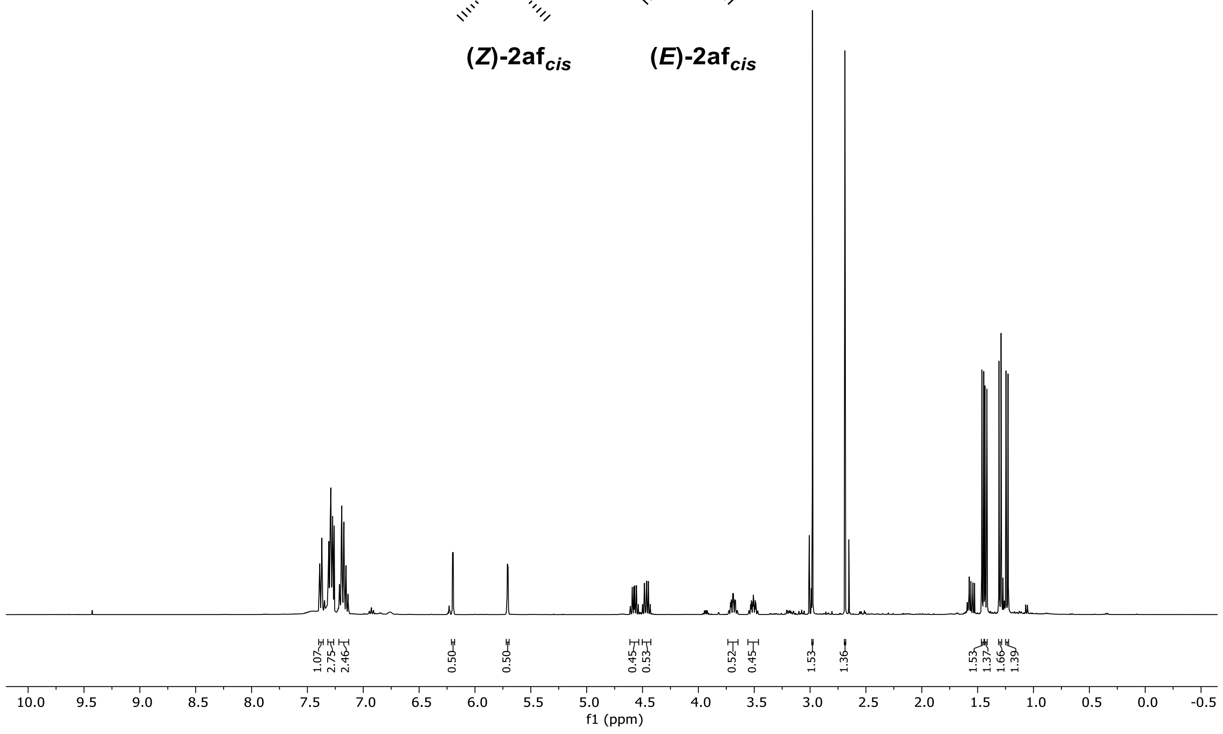

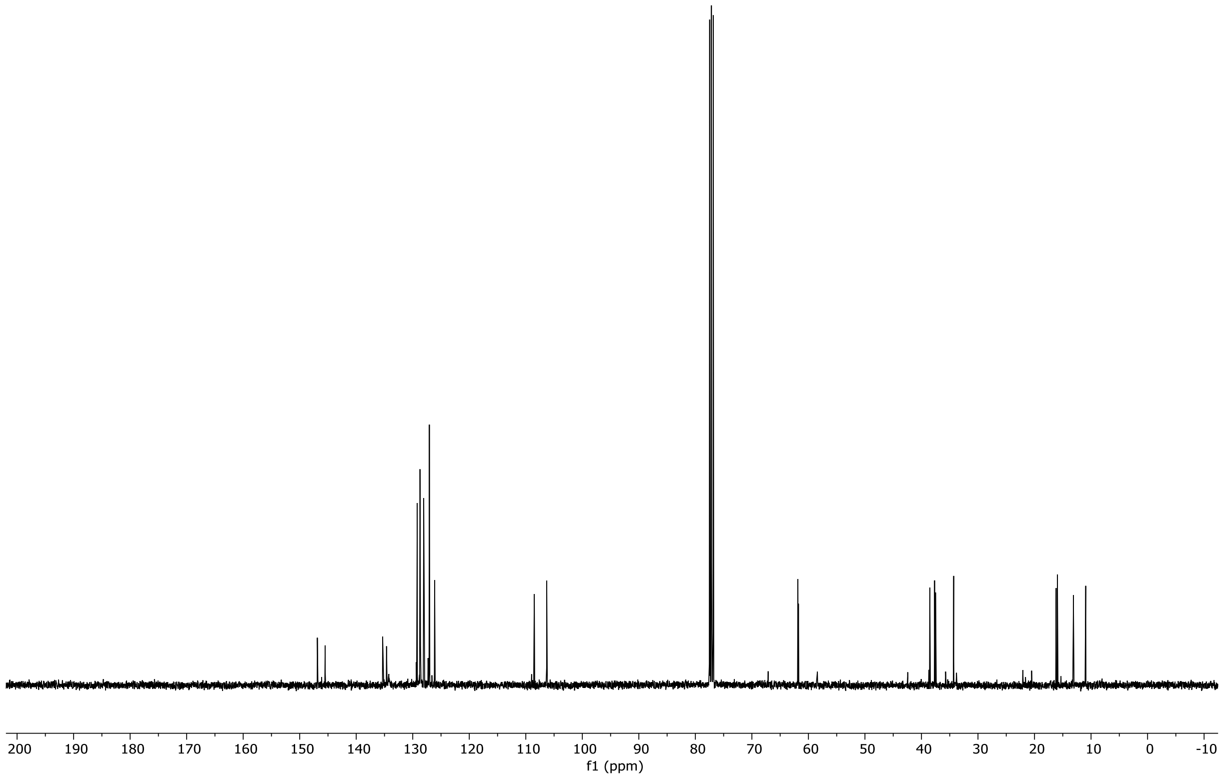

Supplementary Fig 187. ^{1}H (top) and ^{13}C (bottom) NMR spectra of compounds **(*Z*)-2af$_{cis}$** and **(*E*)-2af$_{cis}$**.

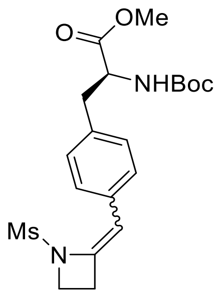

2ag

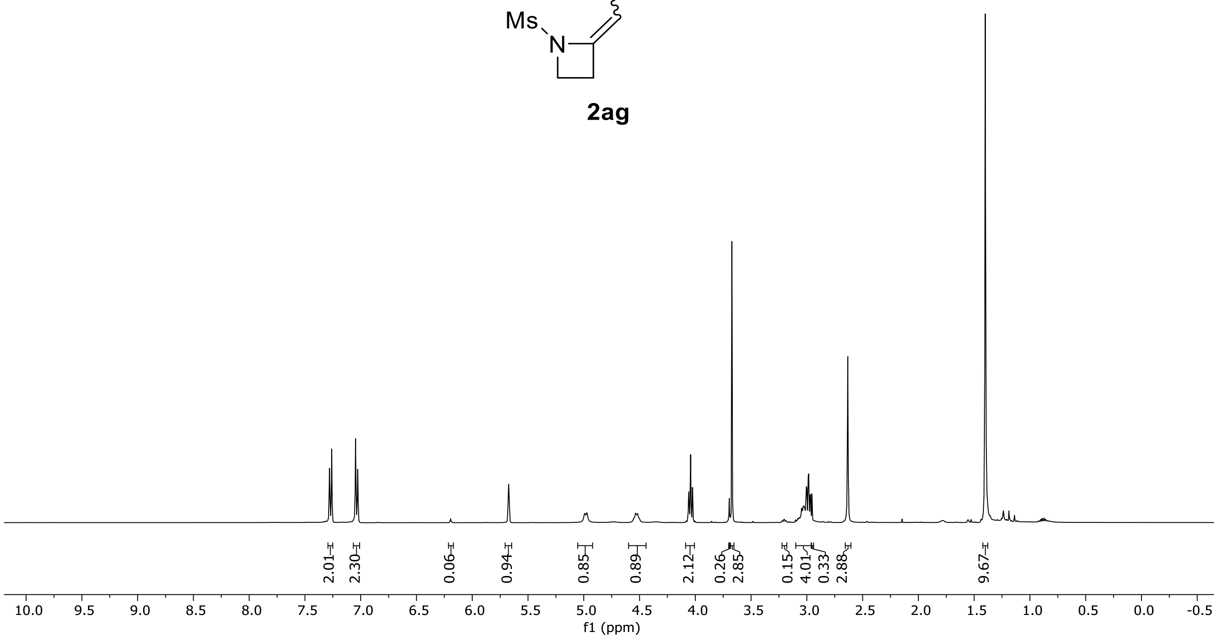

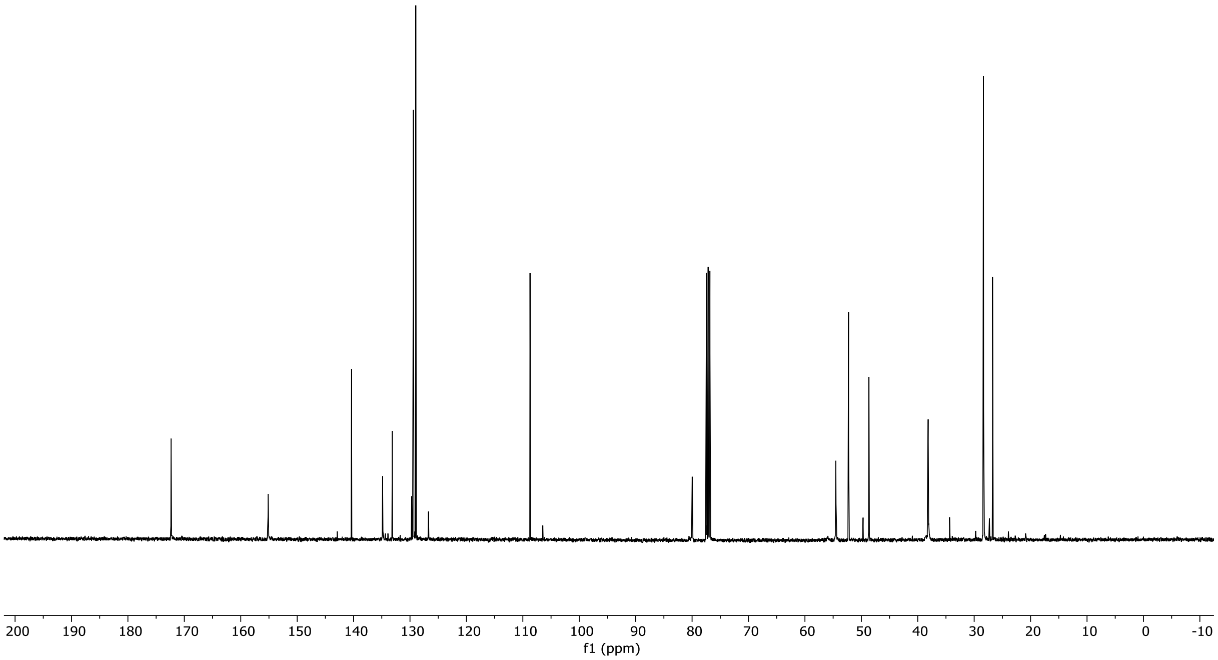

Supplementary Fig 188. ^{1}H (top) and ^{13}C (bottom) NMR spectra of compound **2ag**.

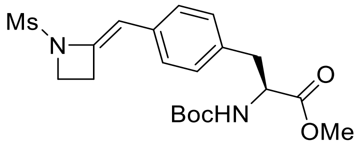

(*E*)-2ag

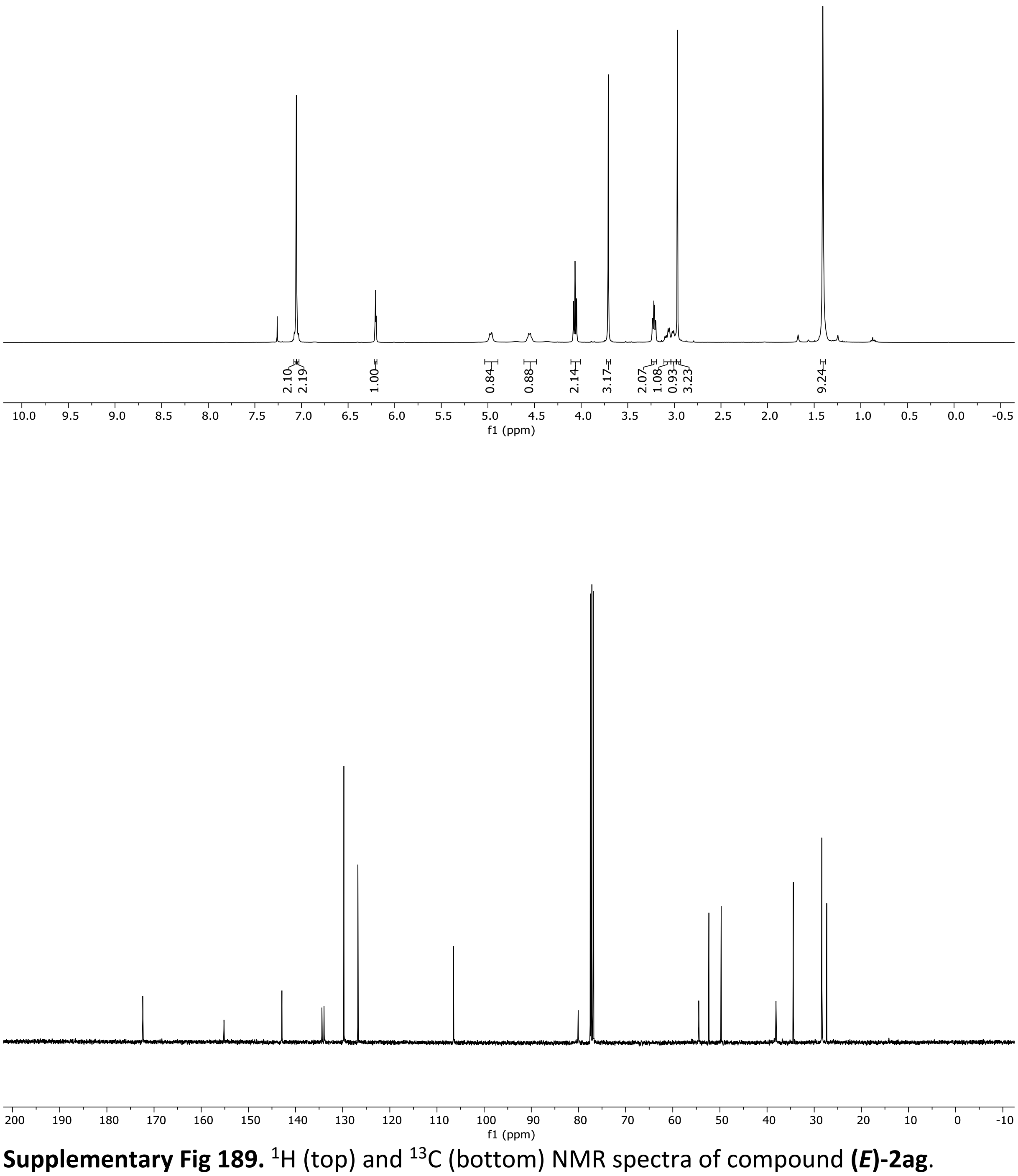

Supplementary Fig 189. ^{1}H (top) and ^{13}C (bottom) NMR spectra of compound **(*E*)-2ag**.

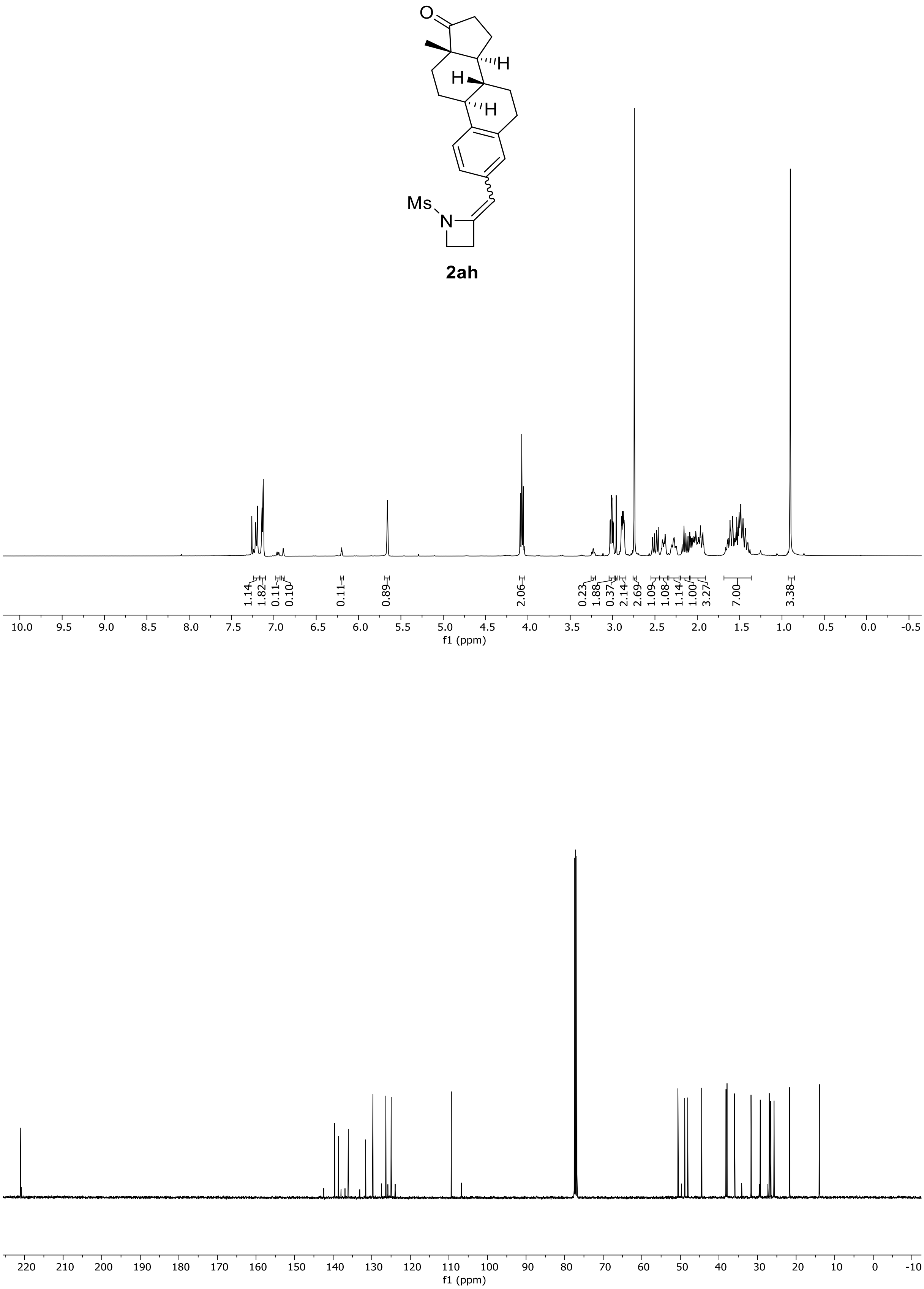

Supplementary Fig 190. ^{1}H (top) and ^{13}C (bottom) NMR spectra of compound **2ah**.

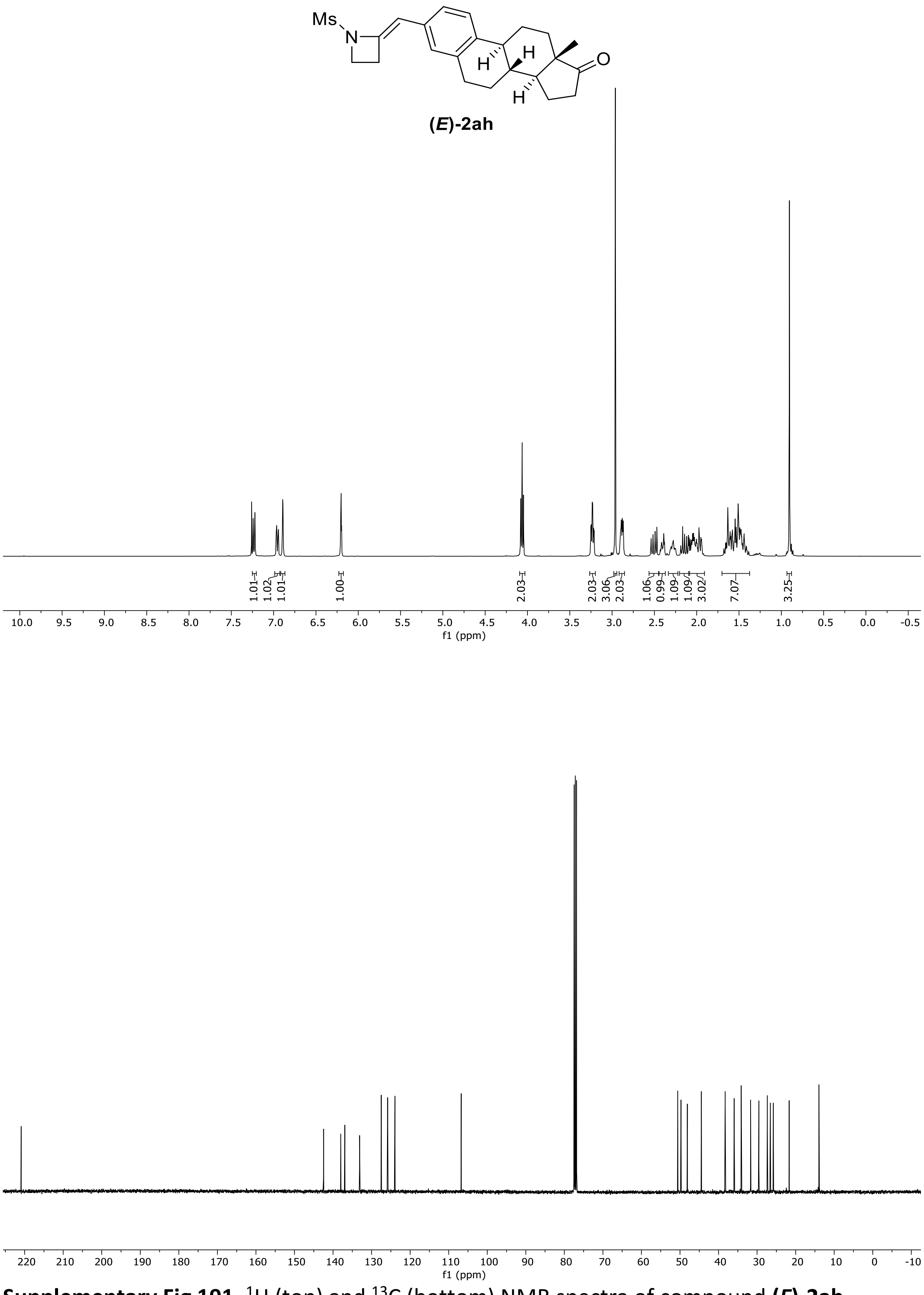

Supplementary Fig 191. ^{1}H (top) and ^{13}C (bottom) NMR spectra of compound **(*E*)-2ah**.

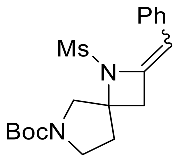

2ai

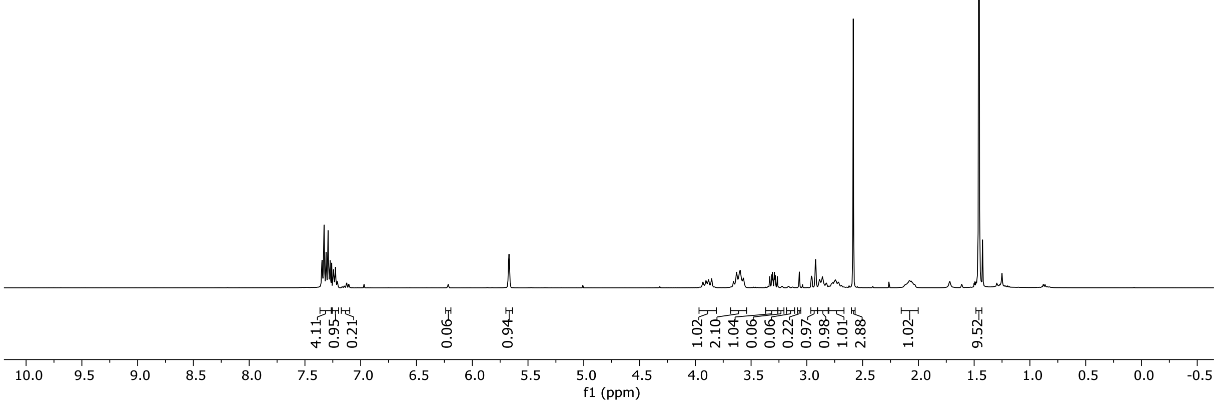

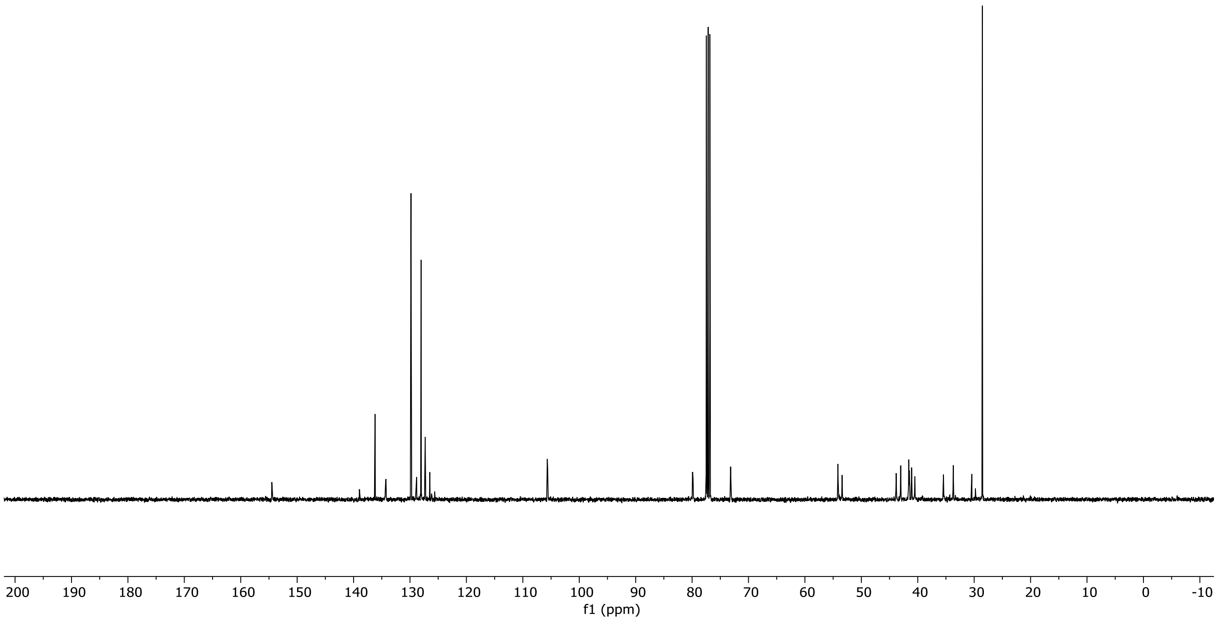

Supplementary Fig 192. ^{1}H (top) and ^{13}C (bottom) NMR spectra of compound **2ai**.

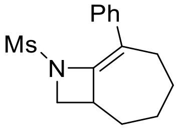

2aj

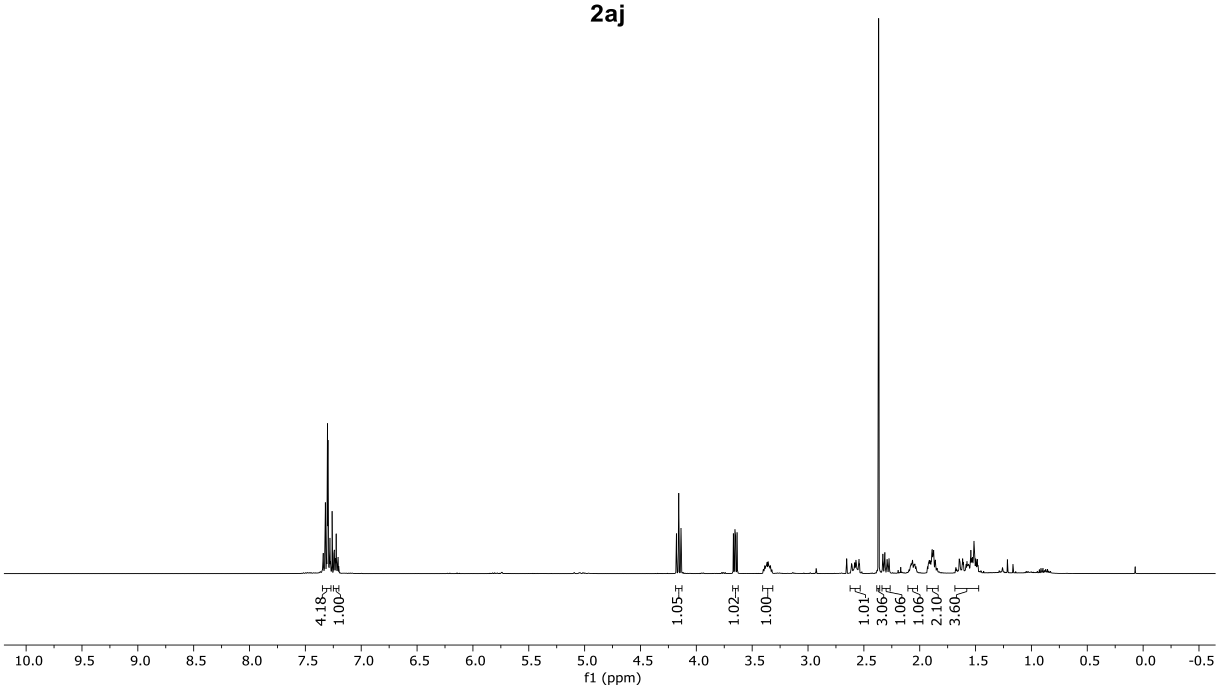

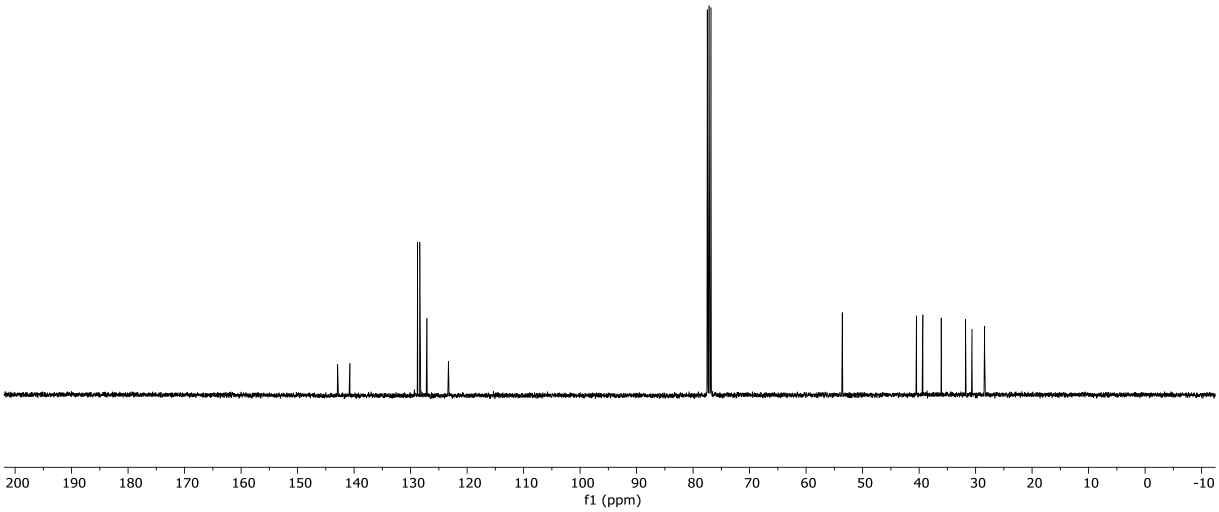

Supplementary Fig 193. ^{1}H (top) and ^{13}C (bottom) NMR spectra of compound **2aj**.

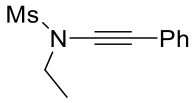

4a

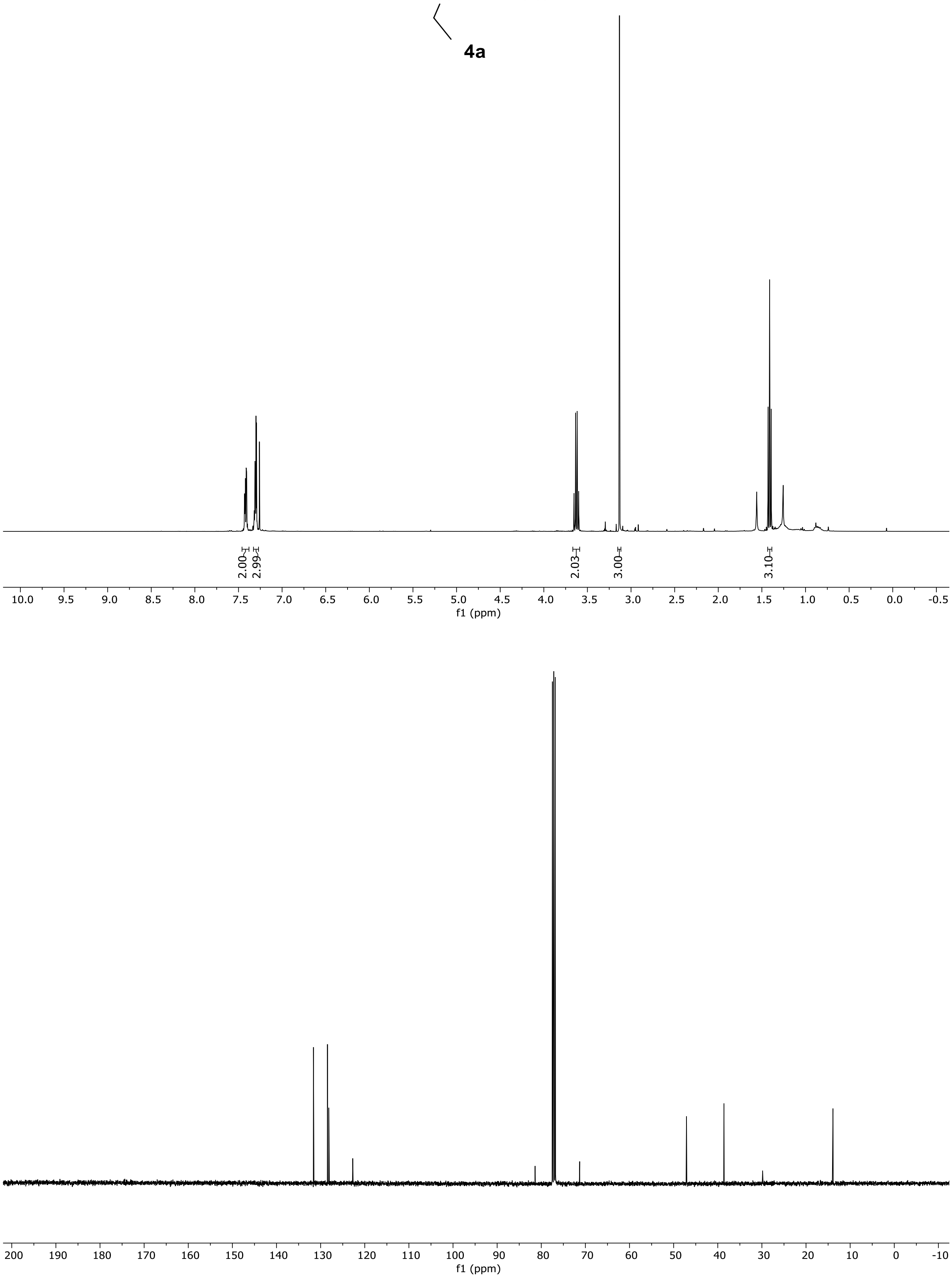

Supplementary Fig 194. ^{1}H (top) and ^{13}C (bottom) NMR spectra of compound **4a**.

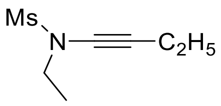

4r

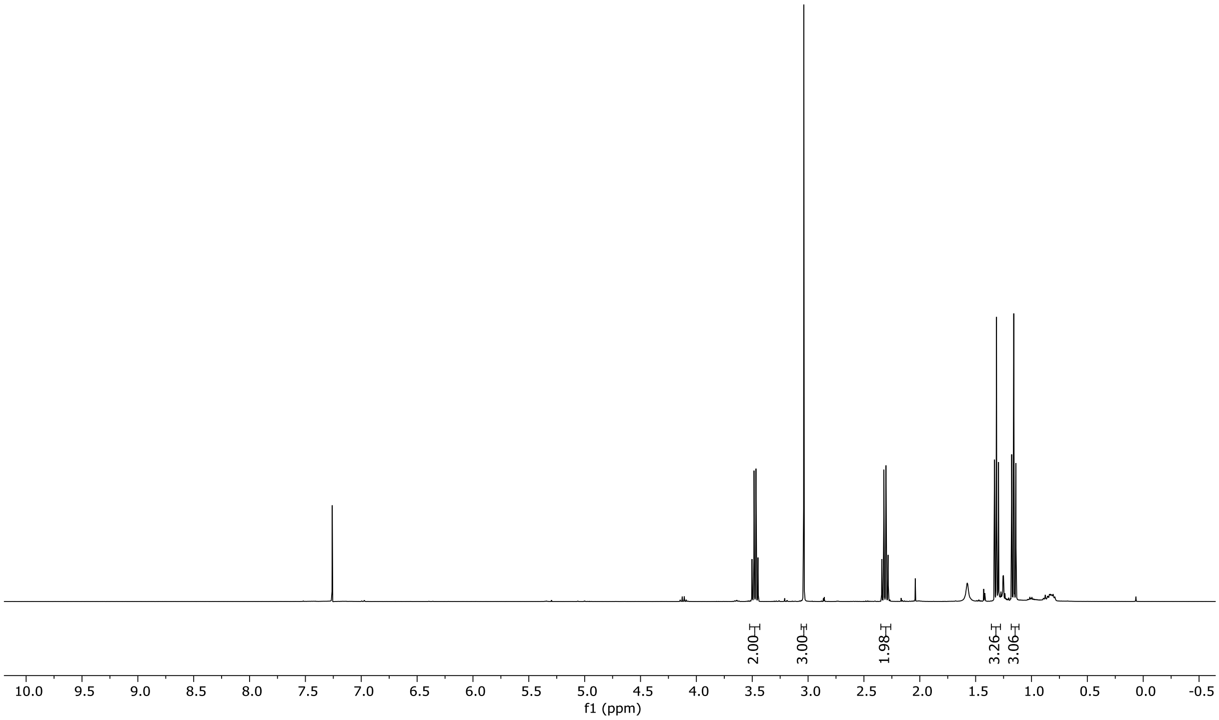

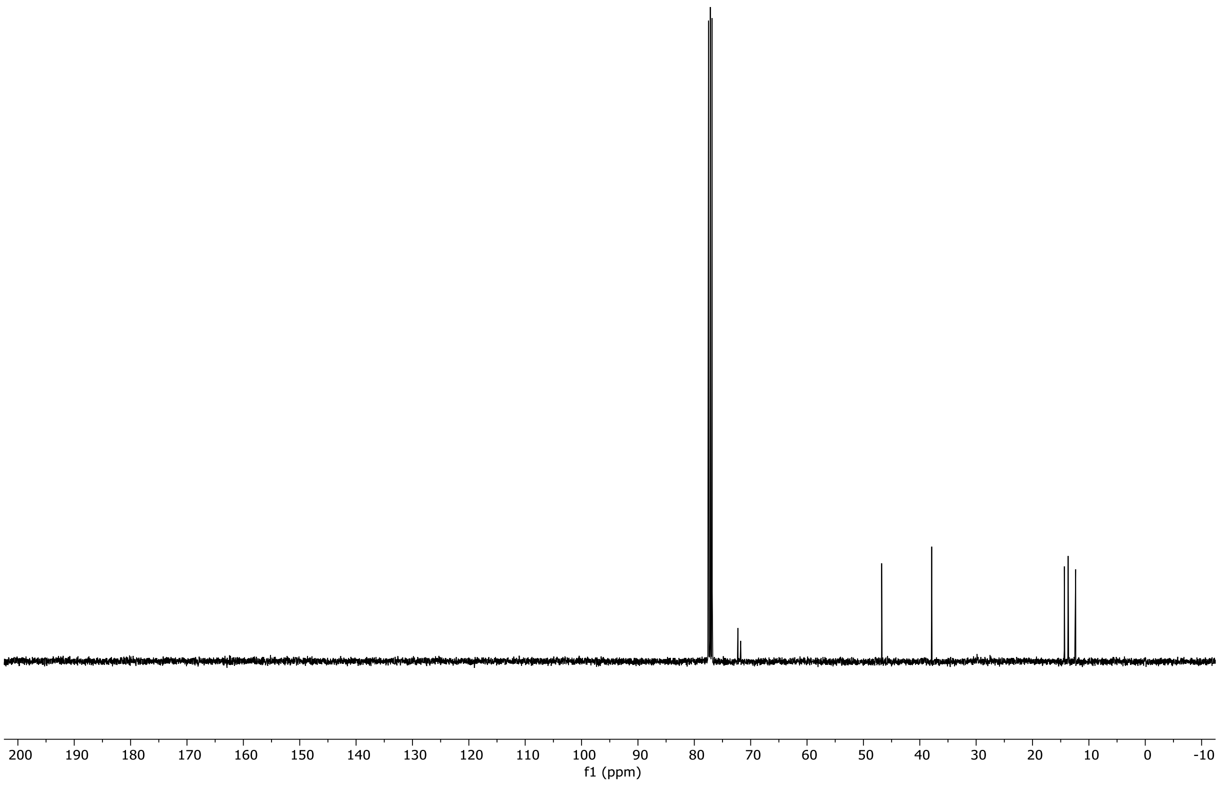

Supplementary Fig 195. ^{1}H (top) and ^{13}C (bottom) NMR spectra of compound **4r**.

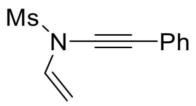

5a

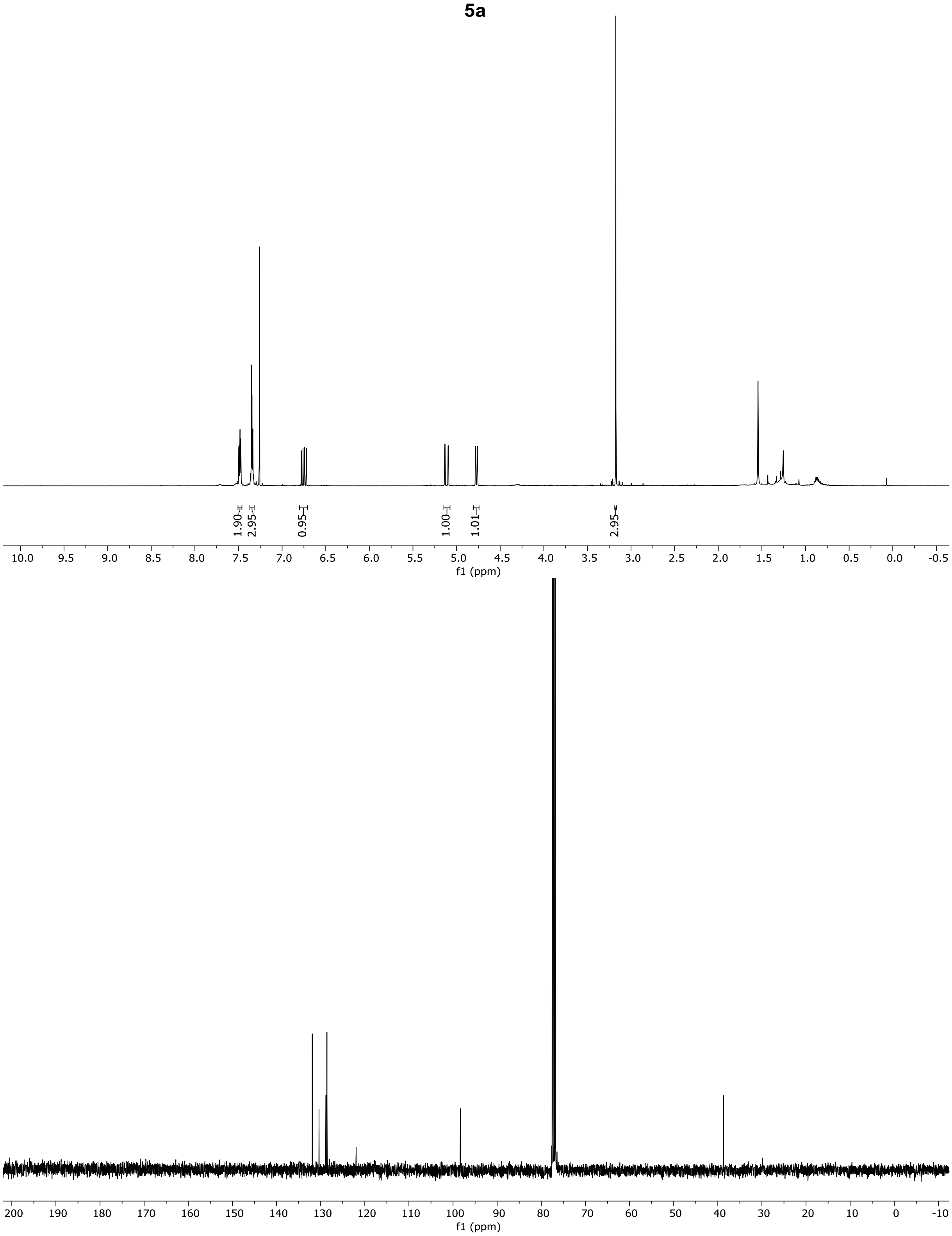

Supplementary Fig 196. ^{1}H (top) and ^{13}C (bottom) NMR spectra of compound **5a**.

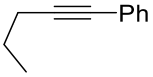

9

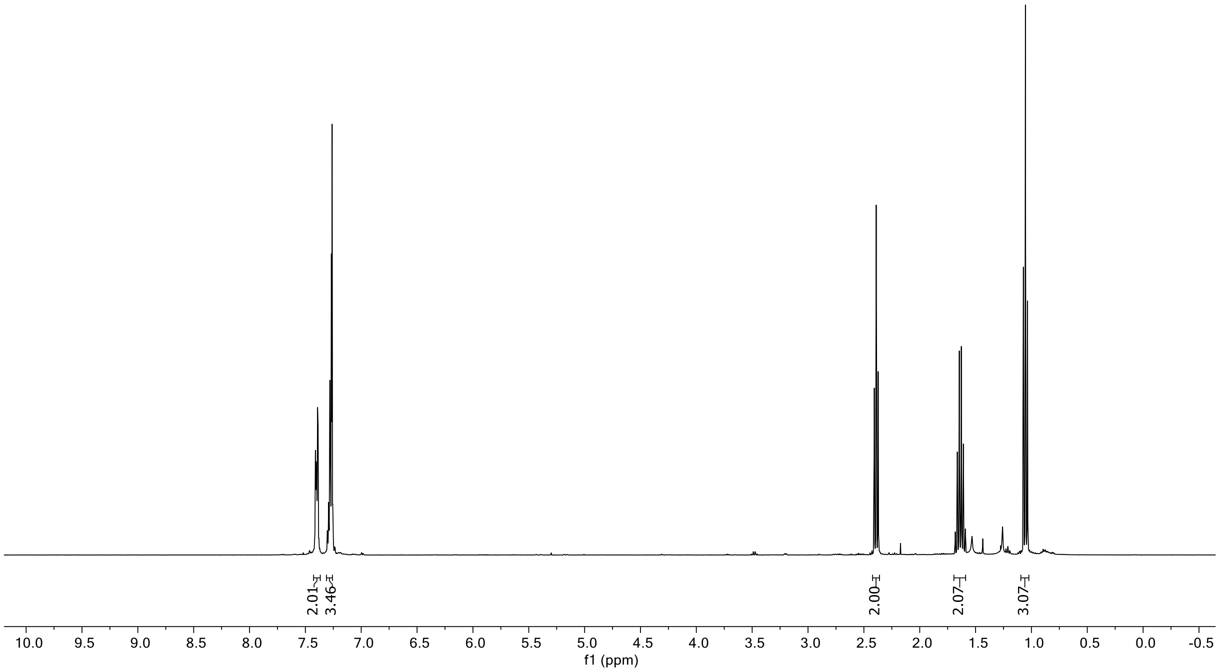

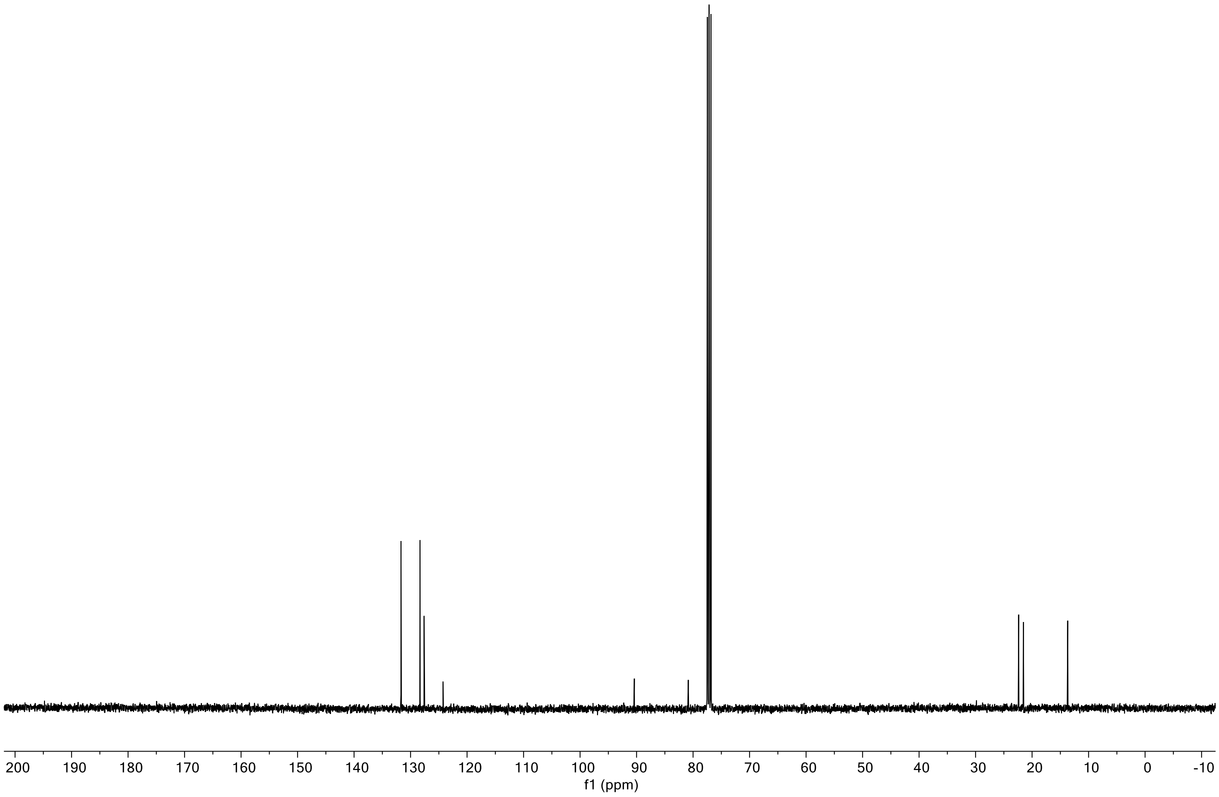

Supplementary Fig 197. ^{1}H (top) and ^{13}C (bottom) NMR spectra of compound **9**.

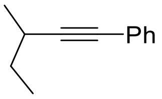

10

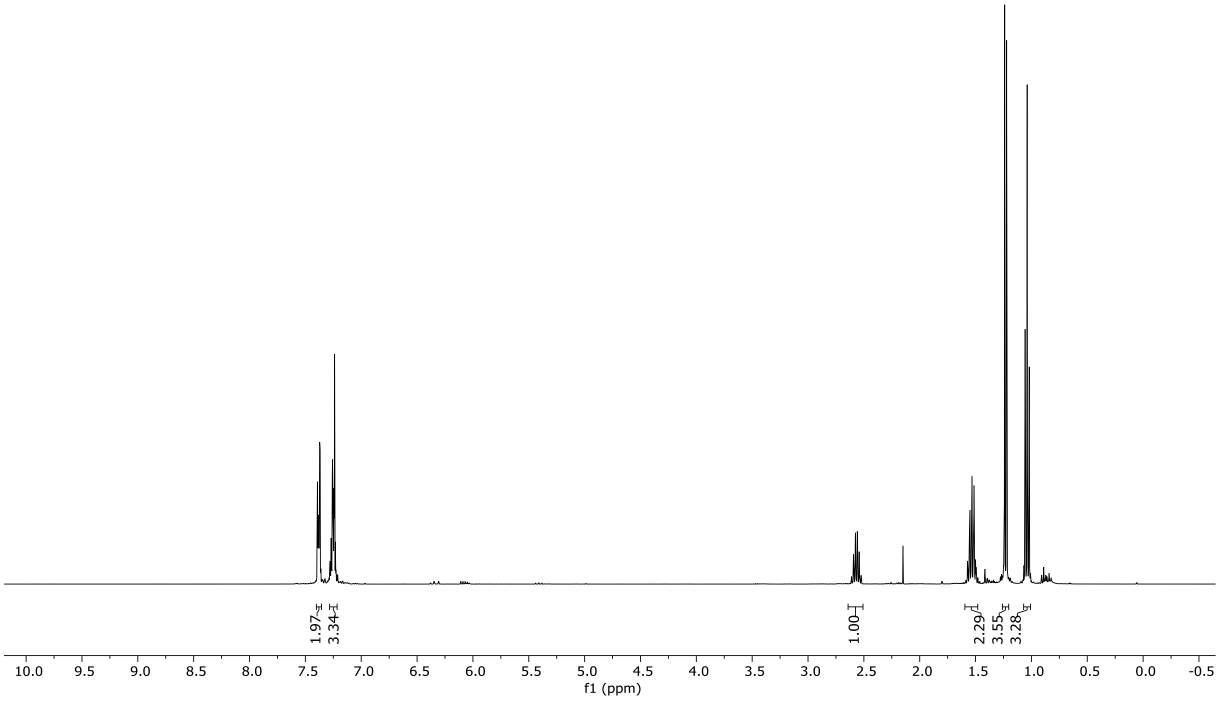

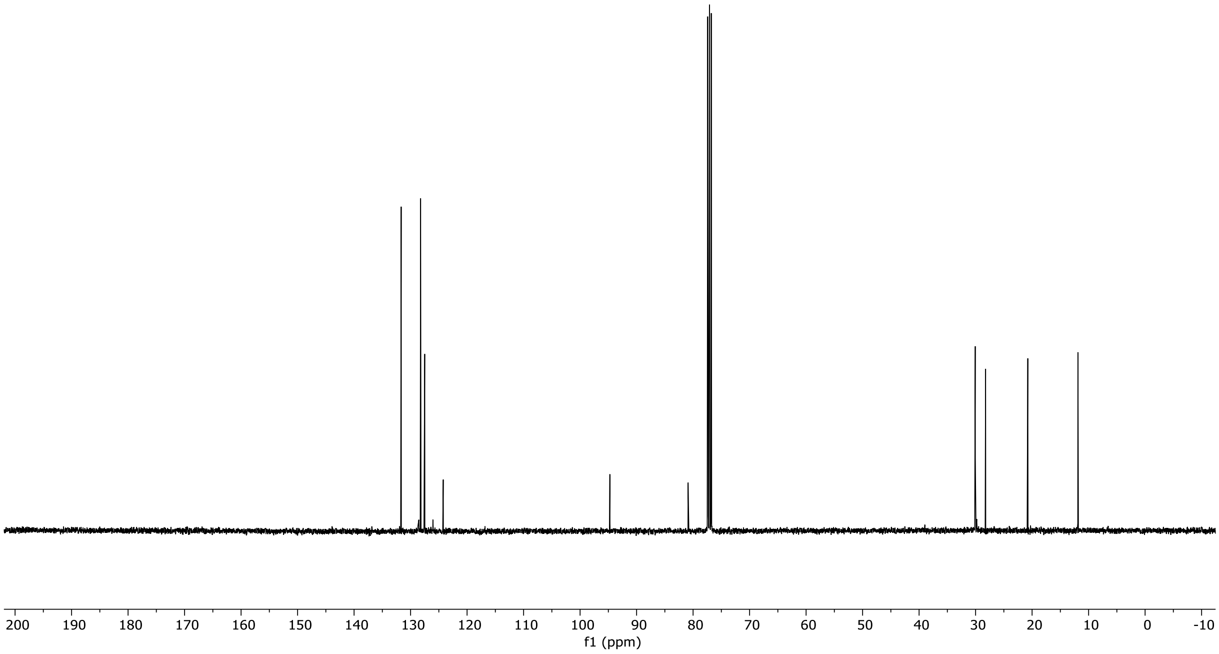

Supplementary Fig 198. ^{1}H (top) and ^{13}C (bottom) NMR spectra of compound **10**.

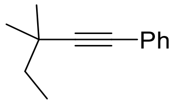

11

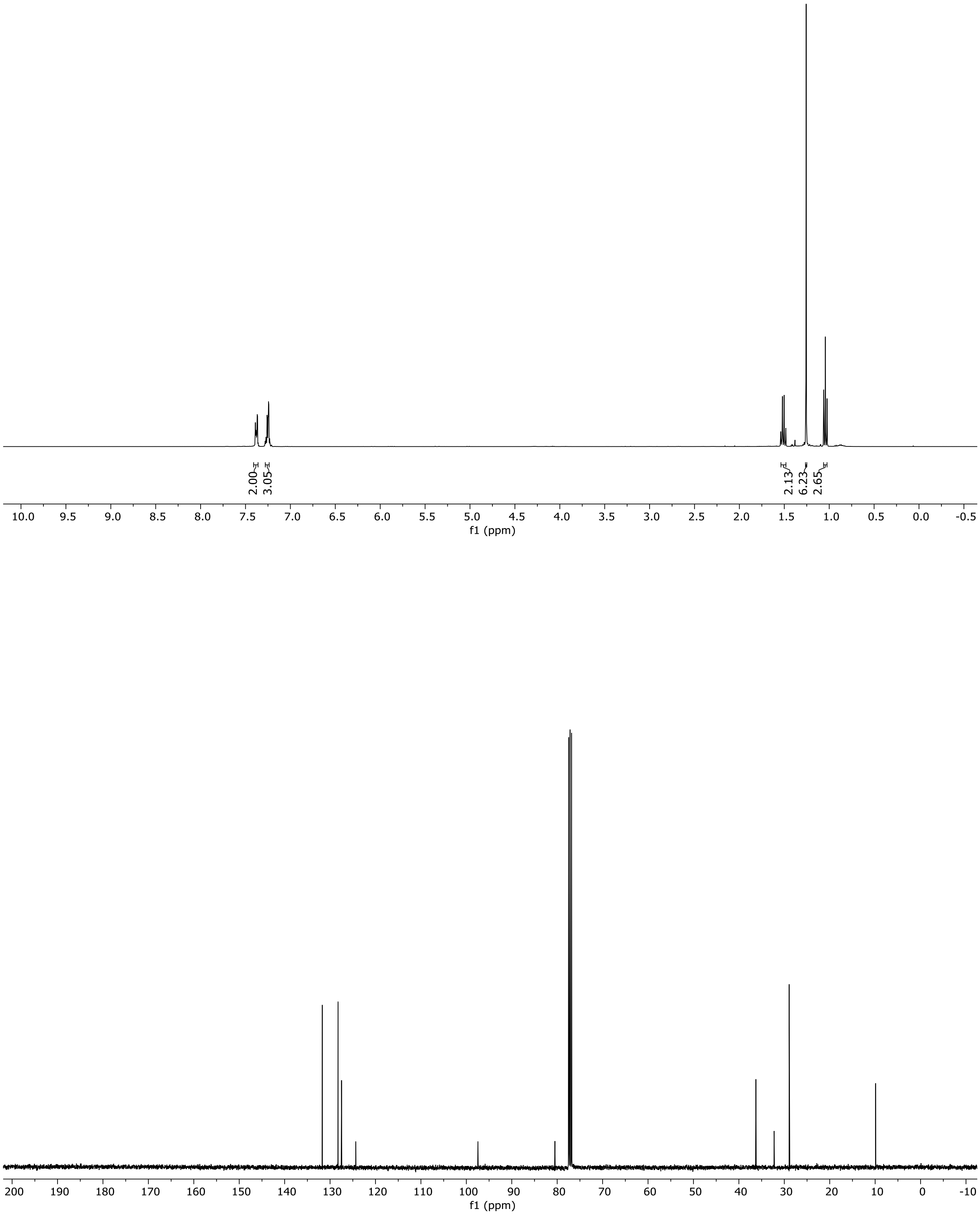

Supplementary Fig 199. ^{1}H (top) and ^{13}C (bottom) NMR spectra of compound **11**.

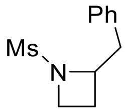

15a

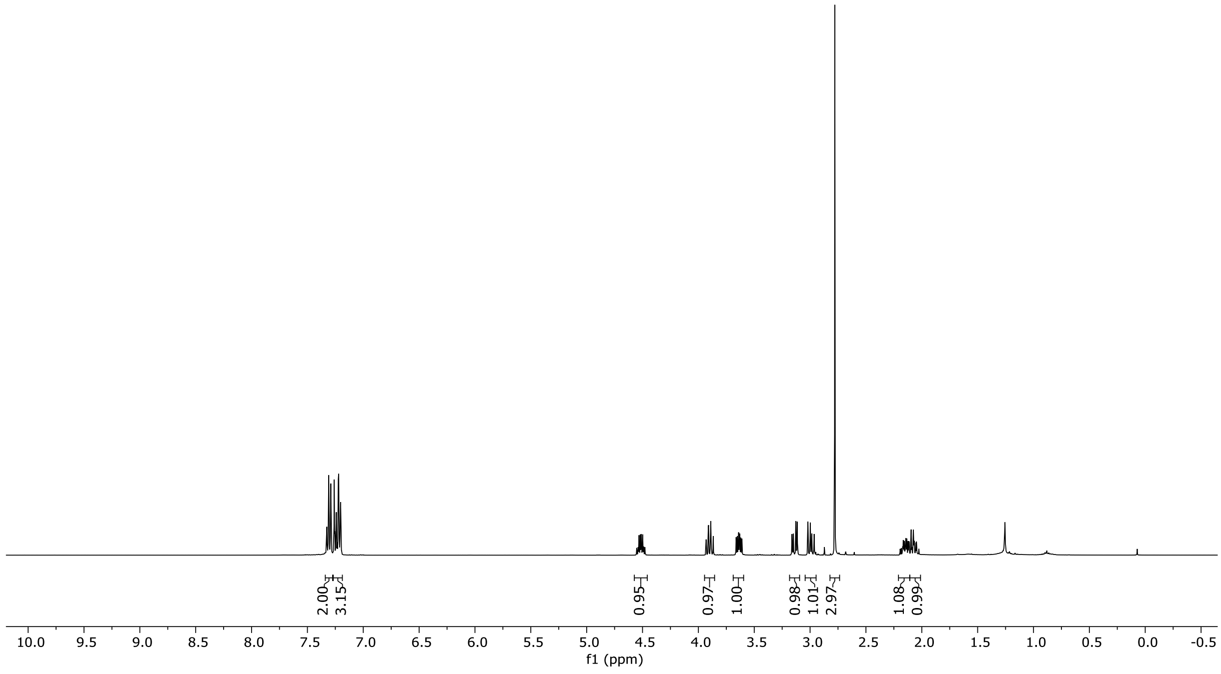

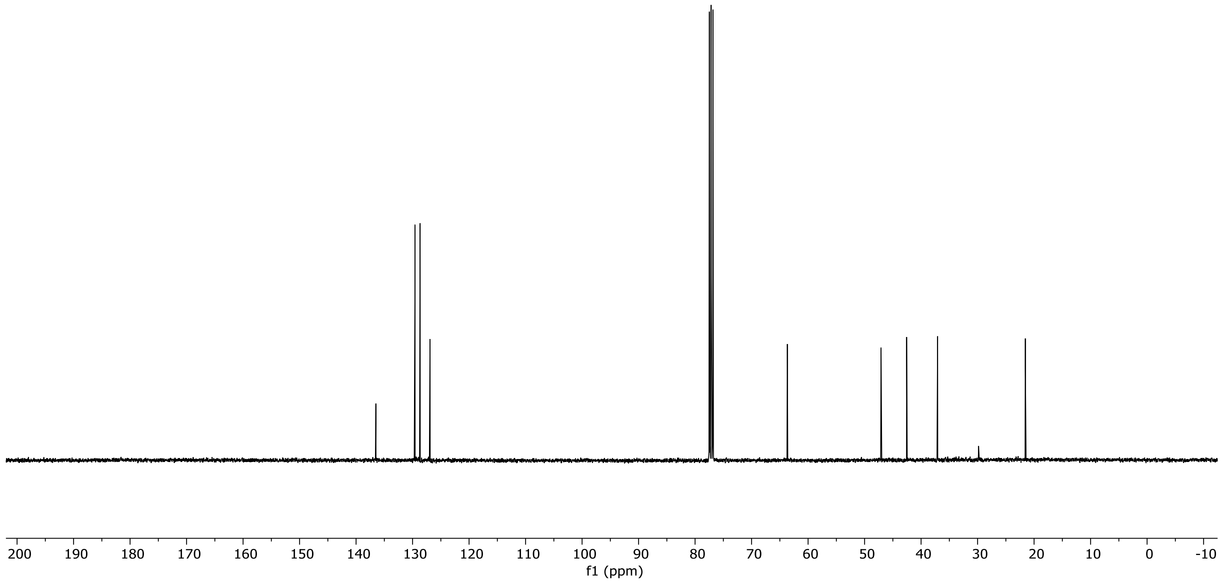

Supplementary Fig 200. ^{1}H (top) and ^{13}C (bottom) NMR spectra of compound **15a**.

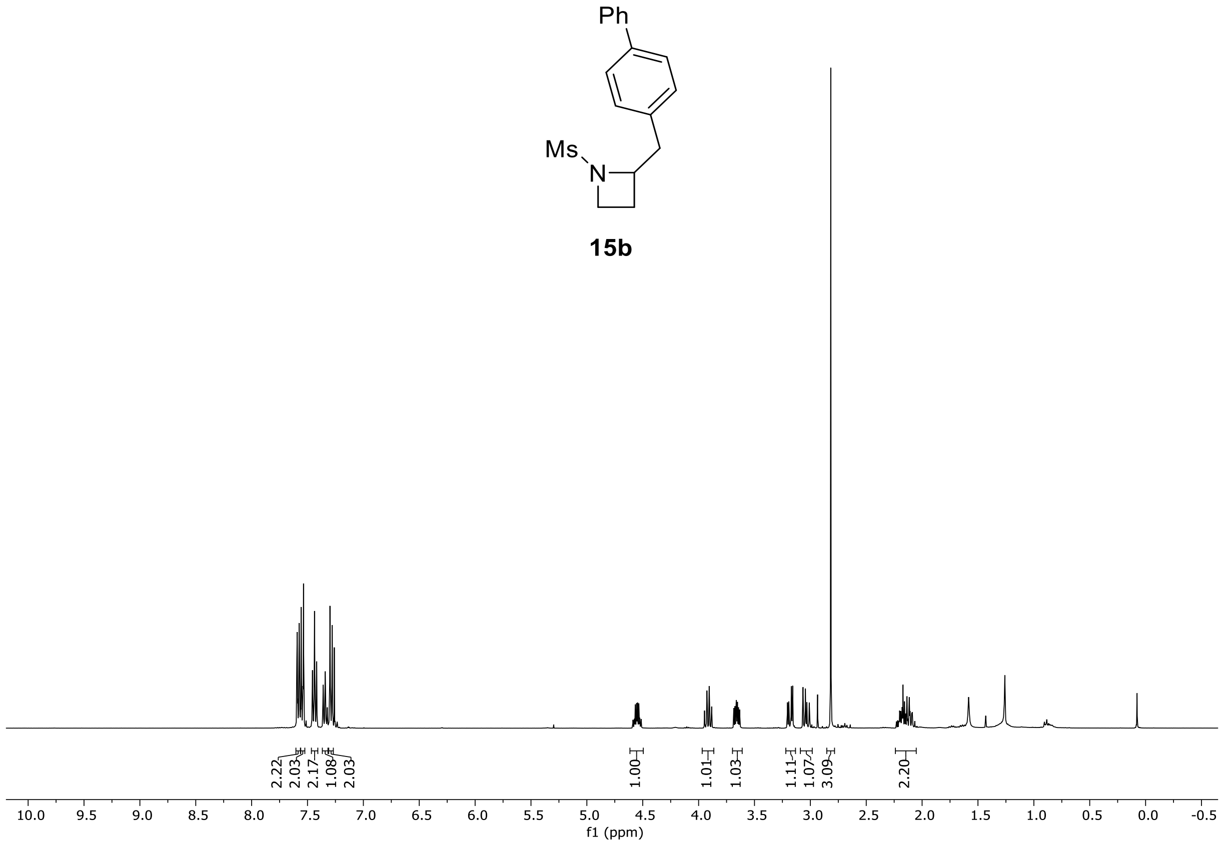

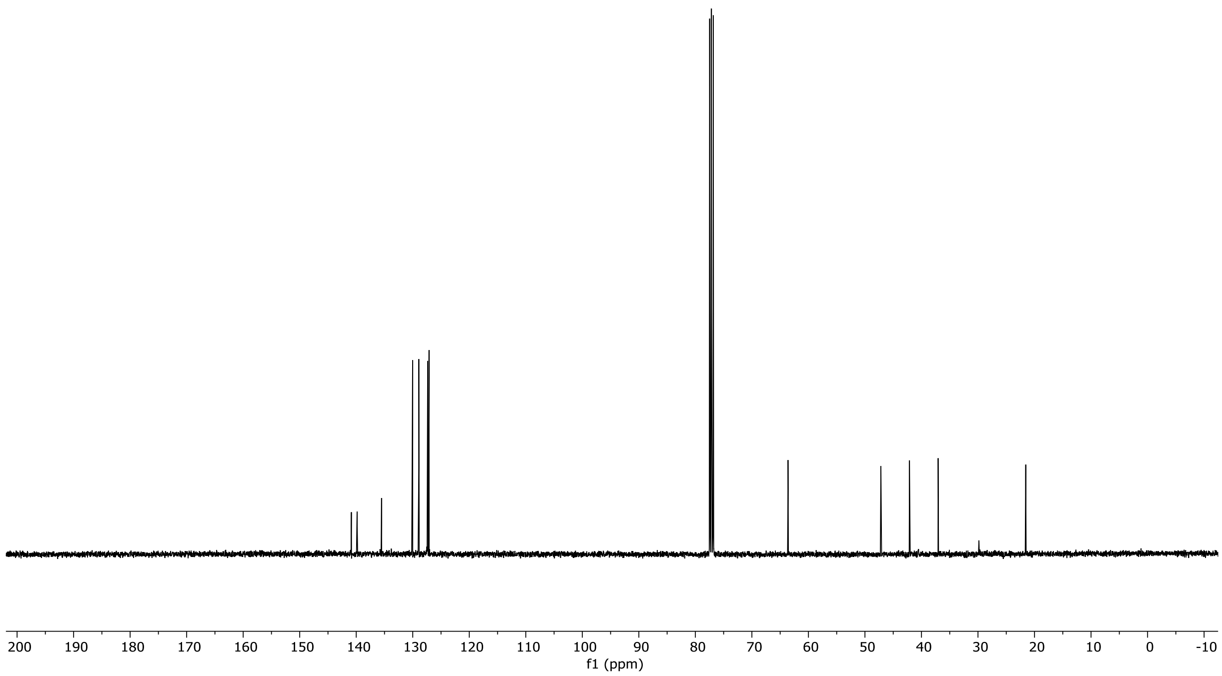

Supplementary Fig 201. ^{1}H (top) and ^{13}C (bottom) NMR spectra of compound **15b**.

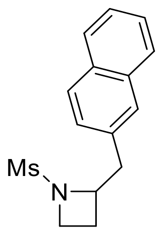

15c

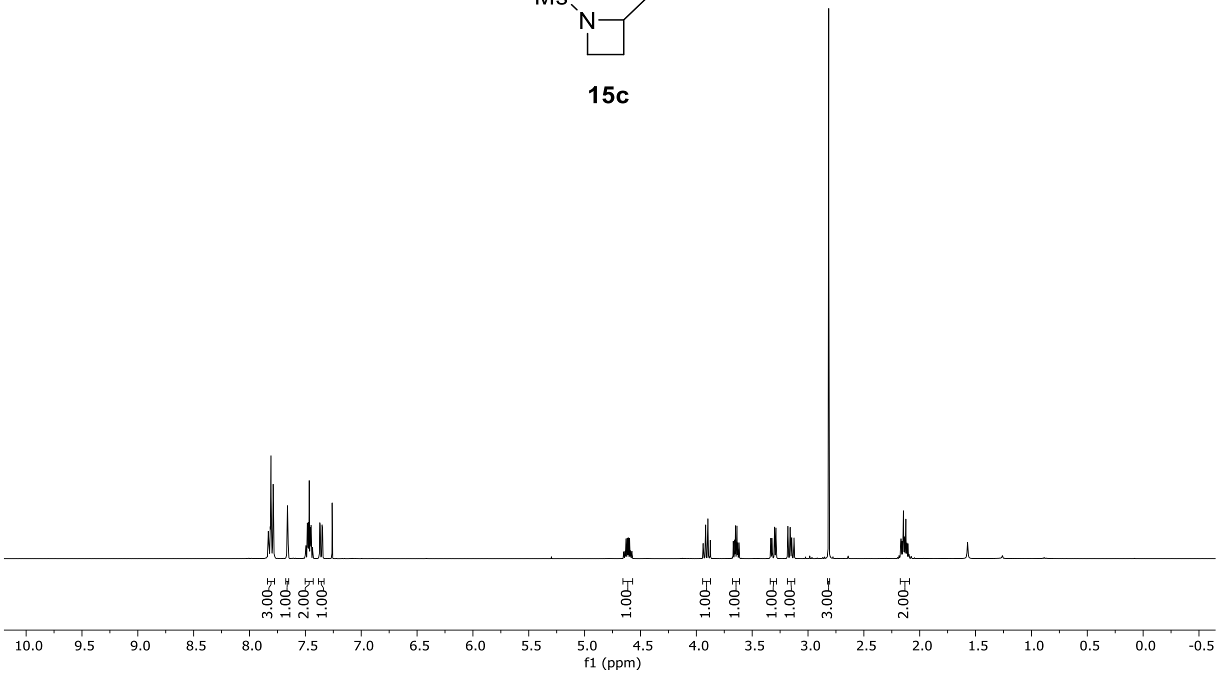

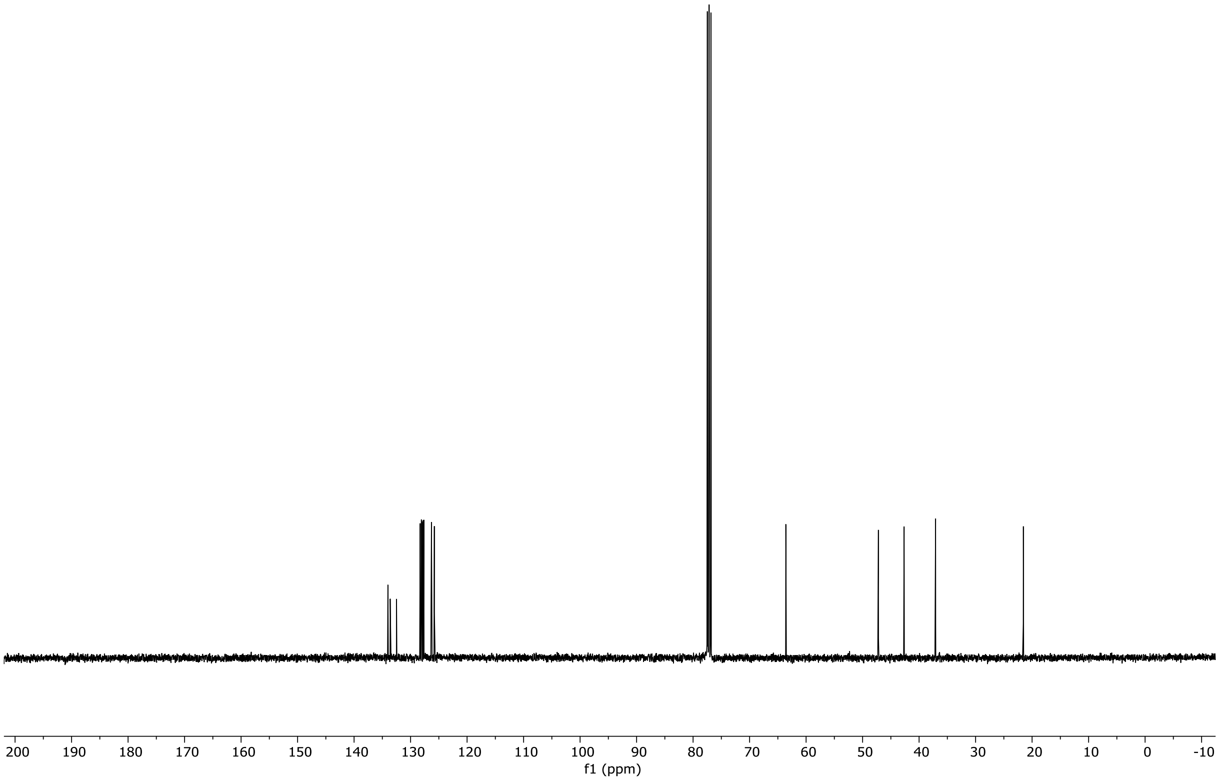

Supplementary Fig 202. ^{1}H (top) and ^{13}C (bottom) NMR spectra of compound **15c**.

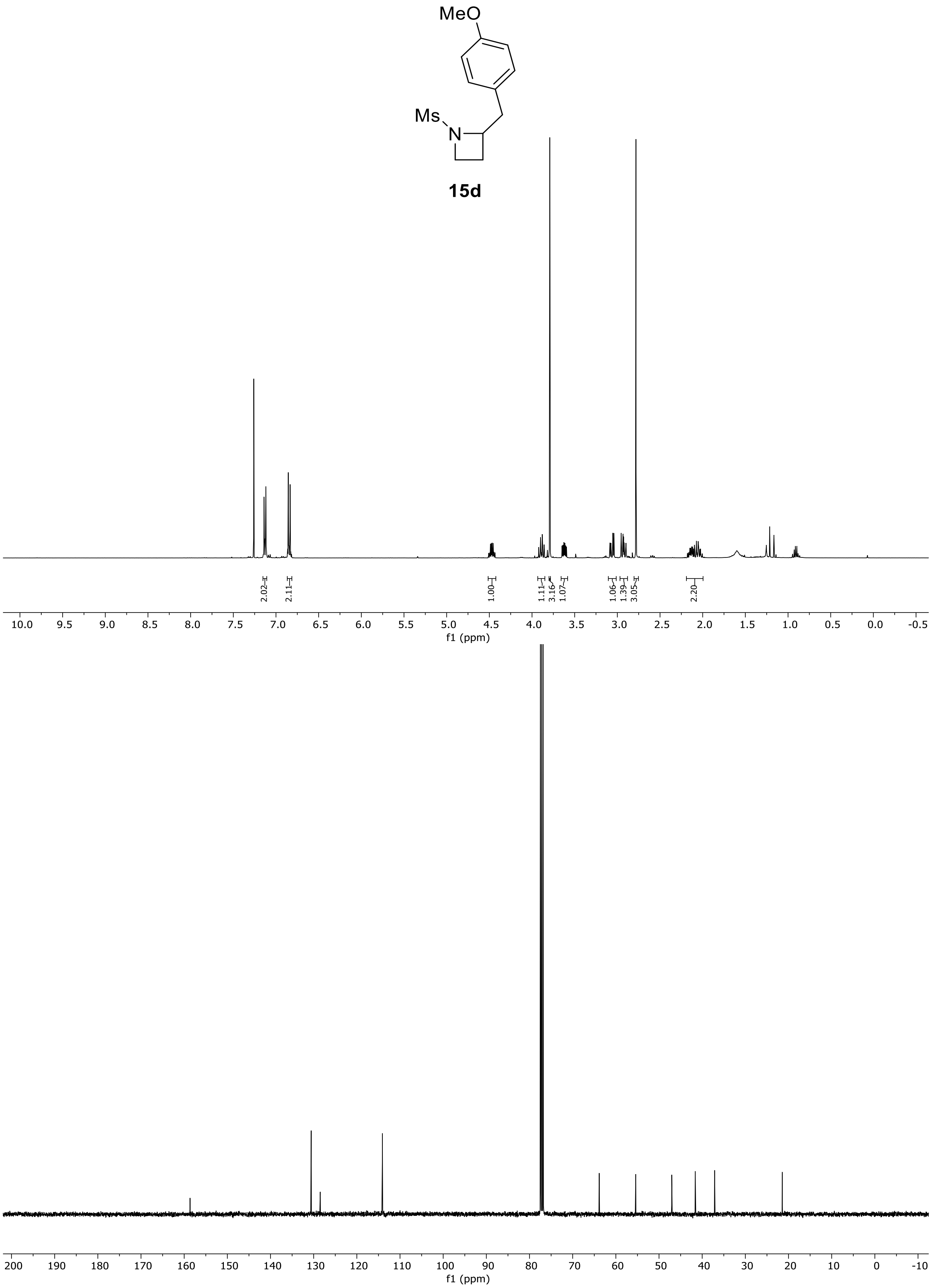

Supplementary Fig 203. ^{1}H (top) and ^{13}C (bottom) NMR spectra of compound **15d**.

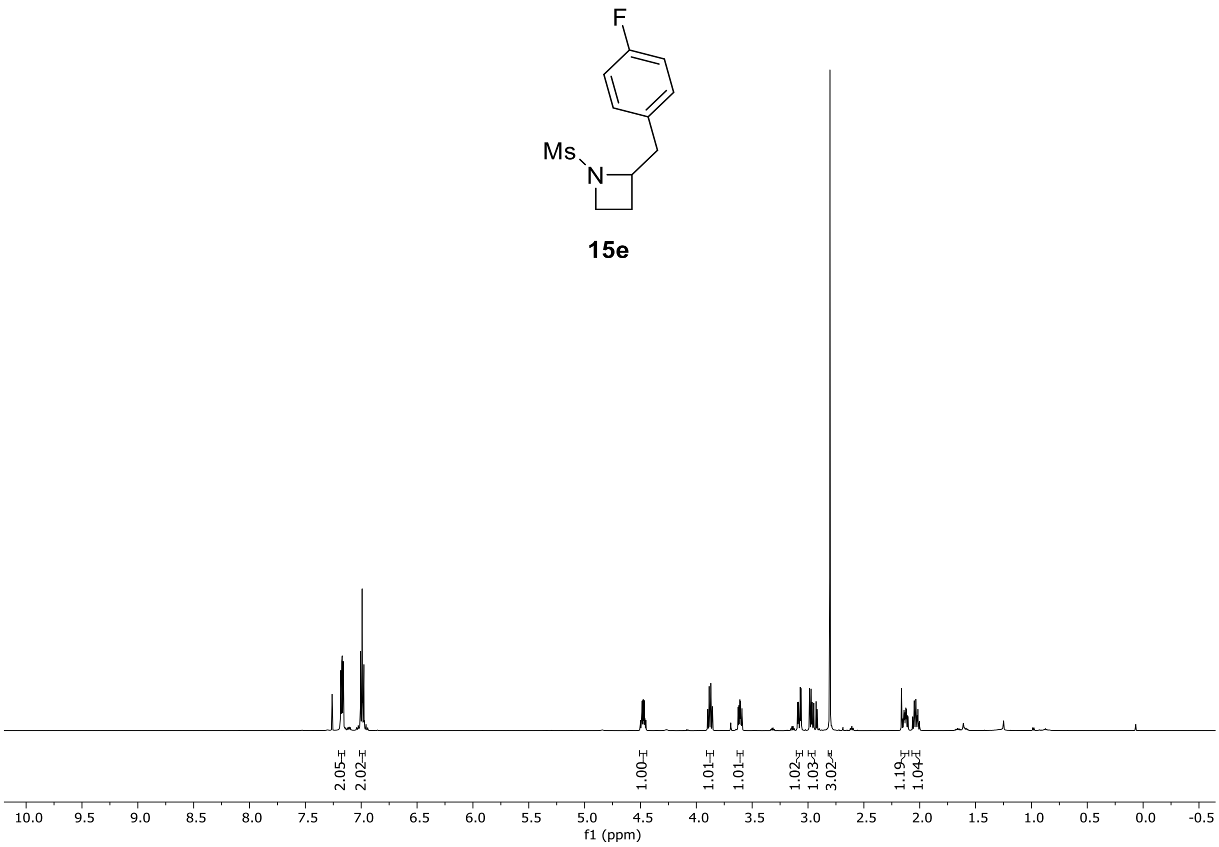

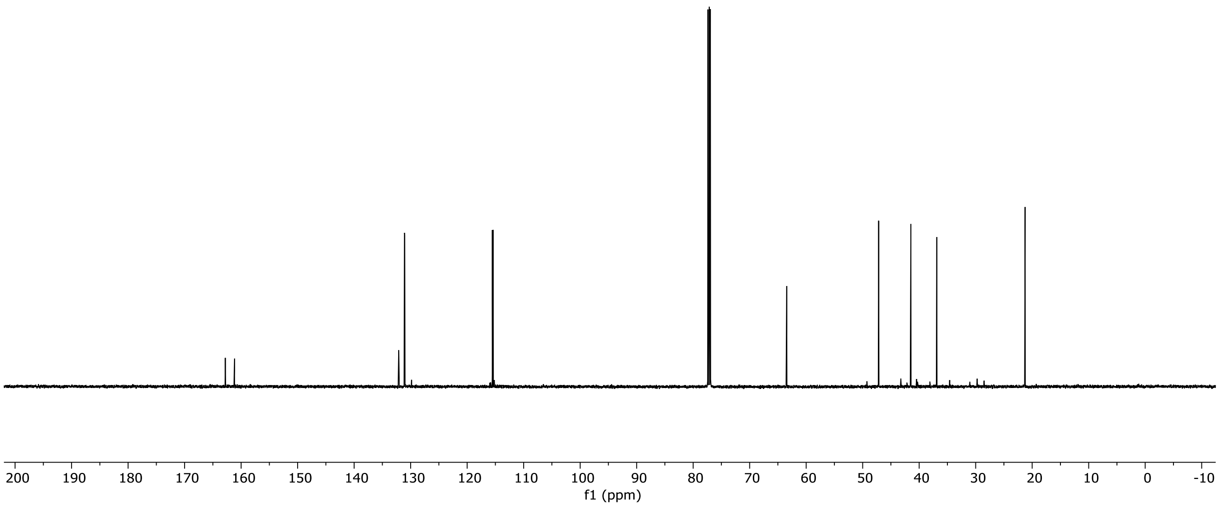

Supplementary Fig 204. ^{1}H (top) and ^{13}C (bottom) NMR spectra of compound **15e**.

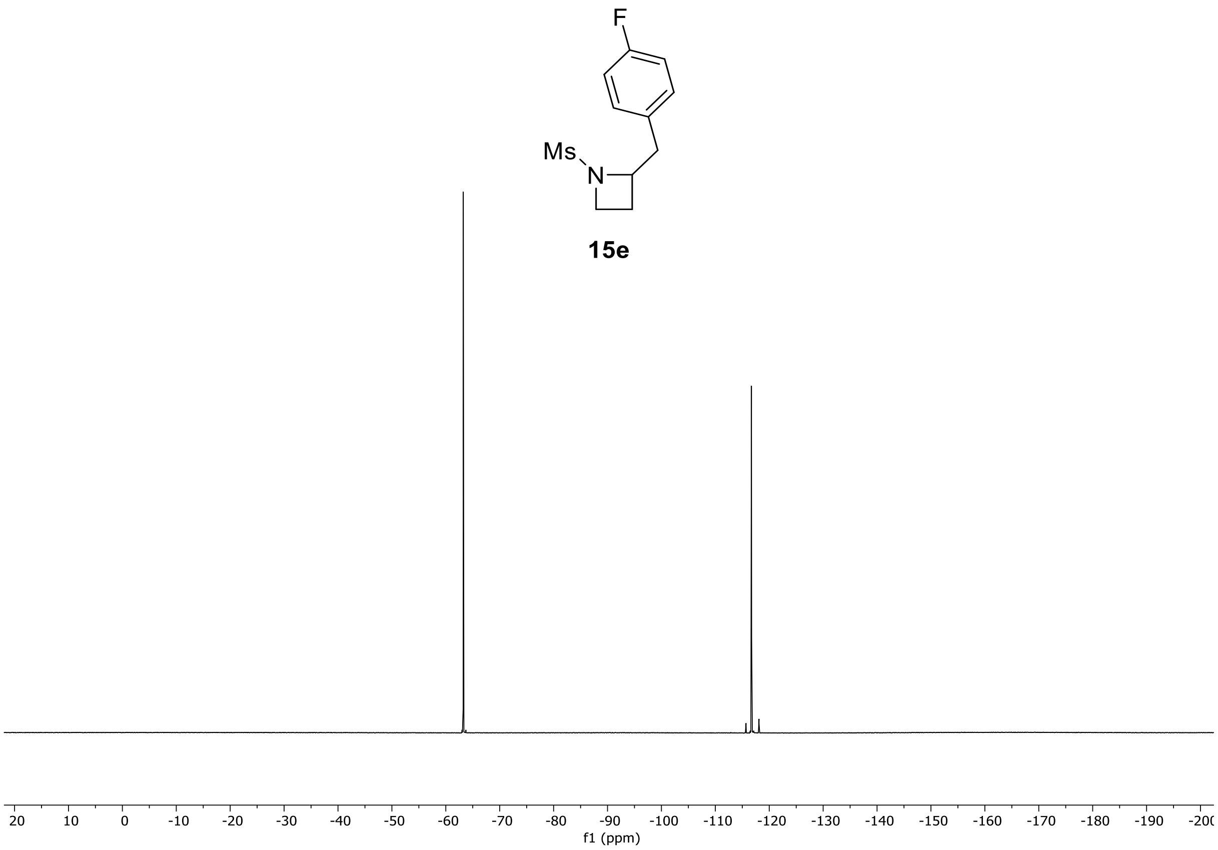

Supplementary Fig 205. ^{19}F NMR spectrum of compound **15e**.

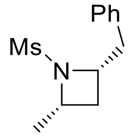

15f

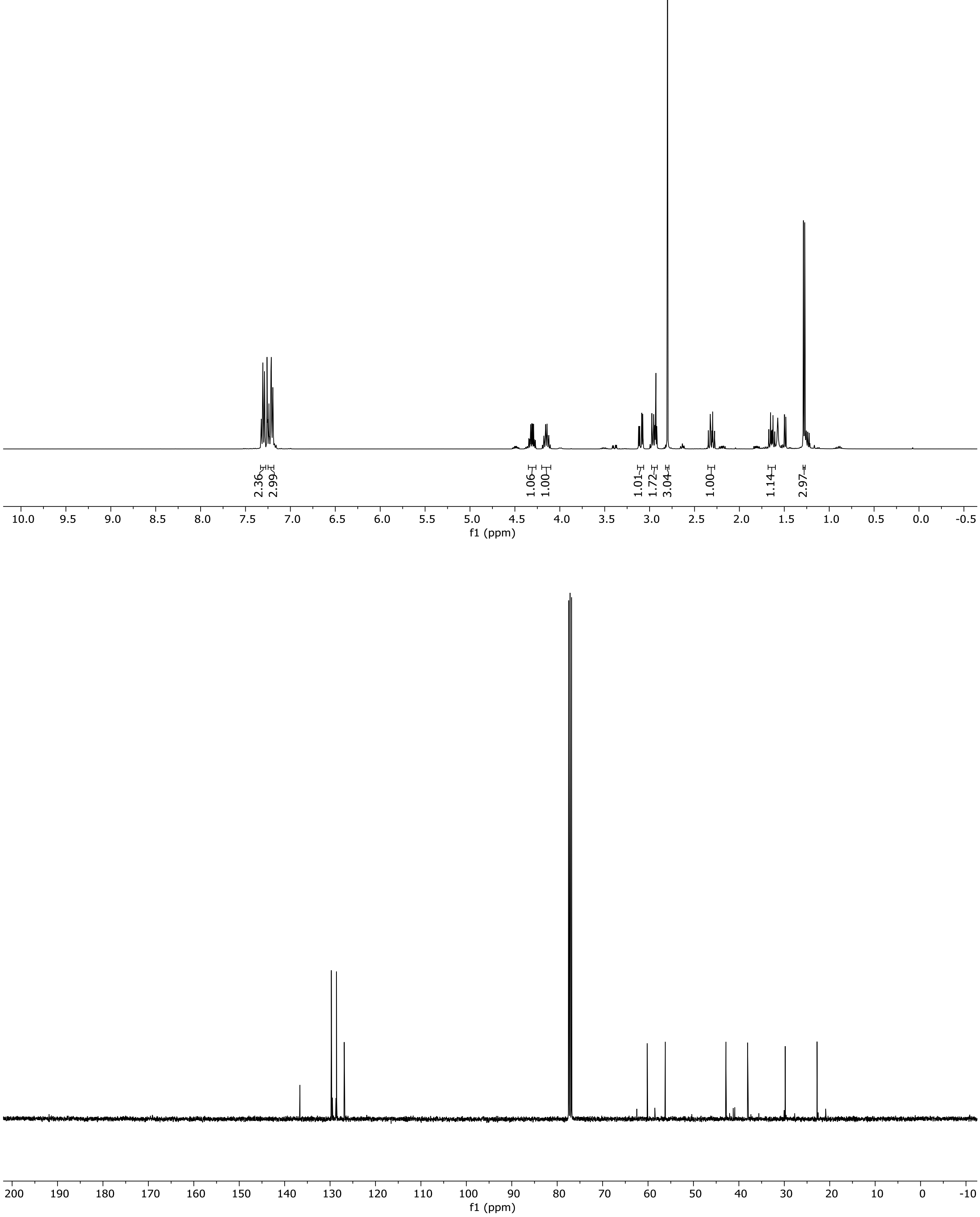

Supplementary Fig 206. ^{1}H (top) and ^{13}C (bottom) NMR spectra of compound **15f**.

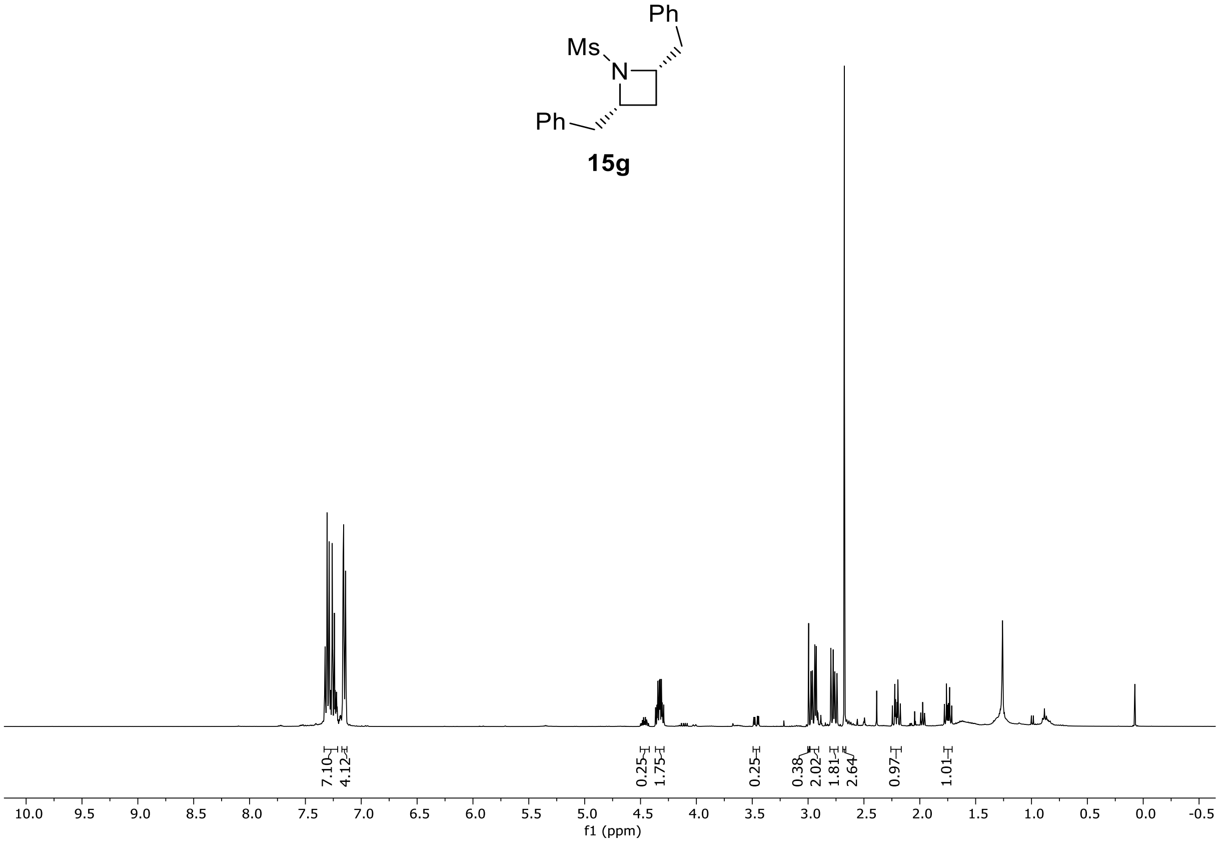

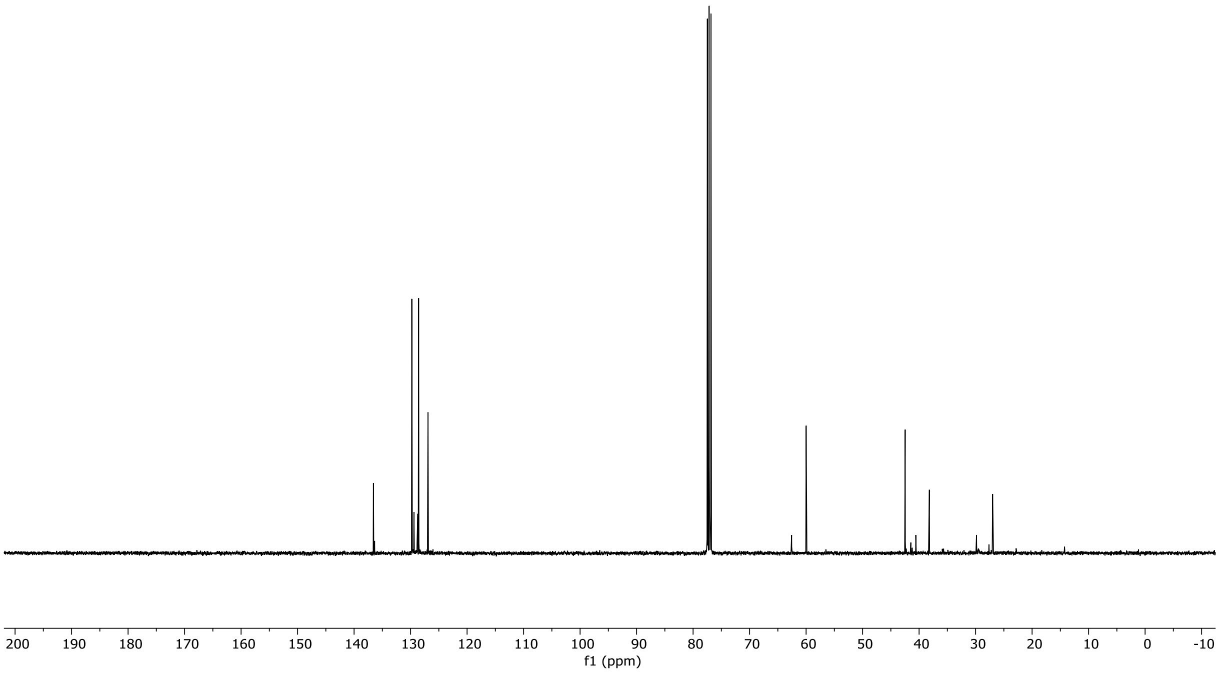

Supplementary Fig 207. ^{1}H (top) and ^{13}C (bottom) NMR spectra of compound **15g**.

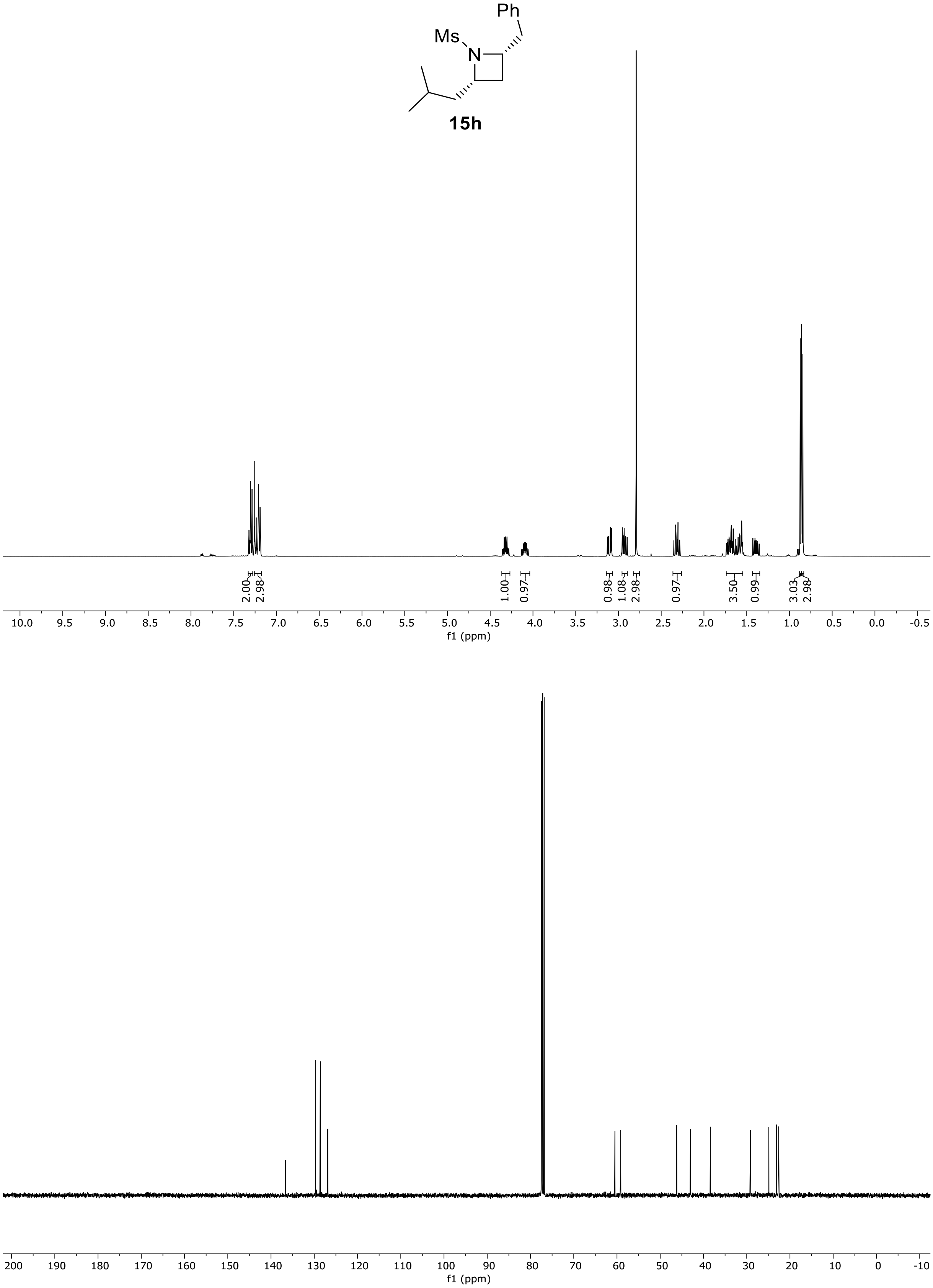

Supplementary Fig 208. ^{1}H (top) and ^{13}C (bottom) NMR spectra of compound **15h**.

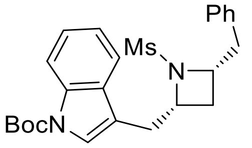

15i

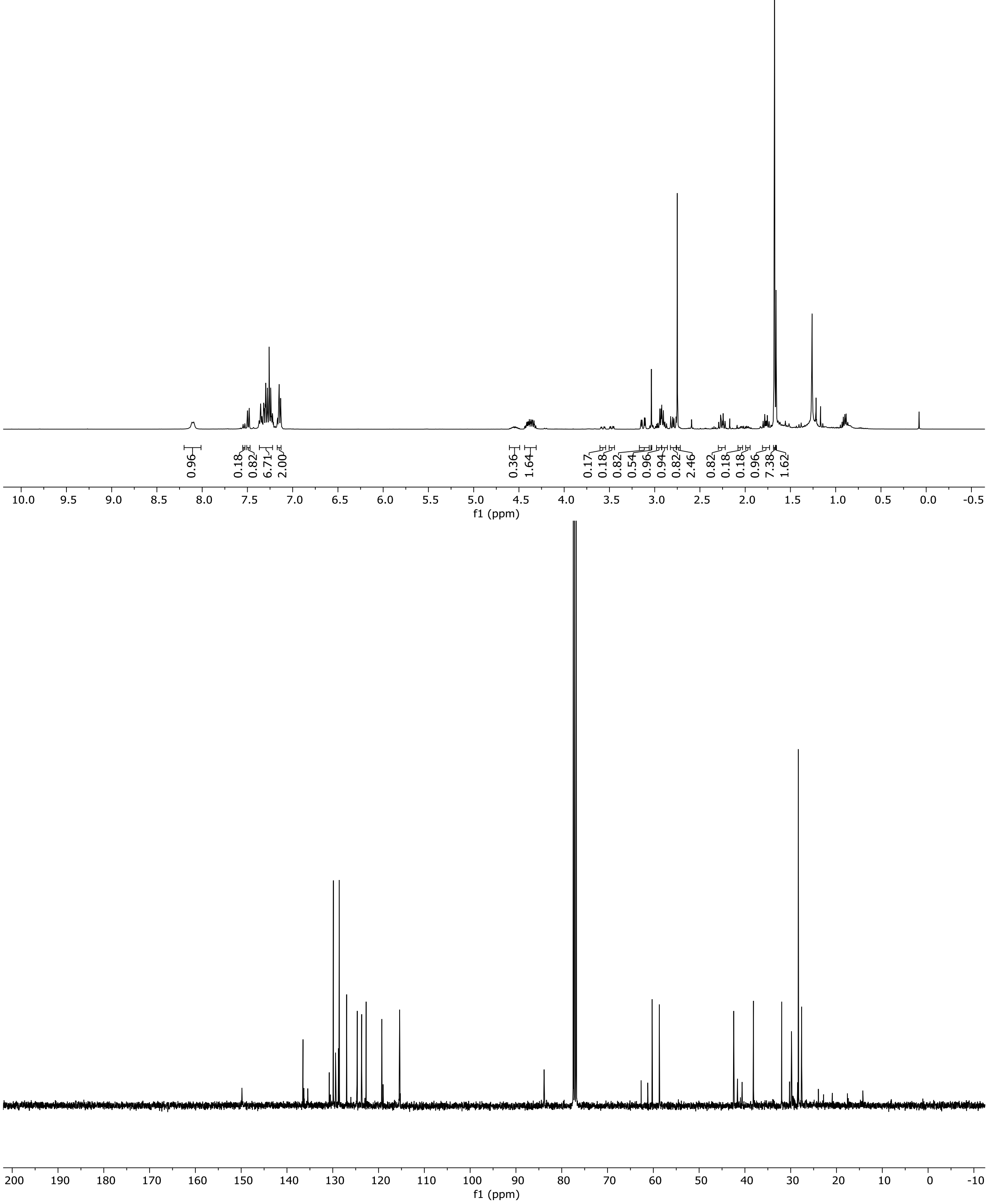

Supplementary Fig 209. ^{1}H (top) and ^{13}C (bottom) NMR spectra of compound **15i**.

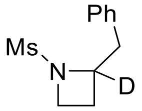

15a$_D$

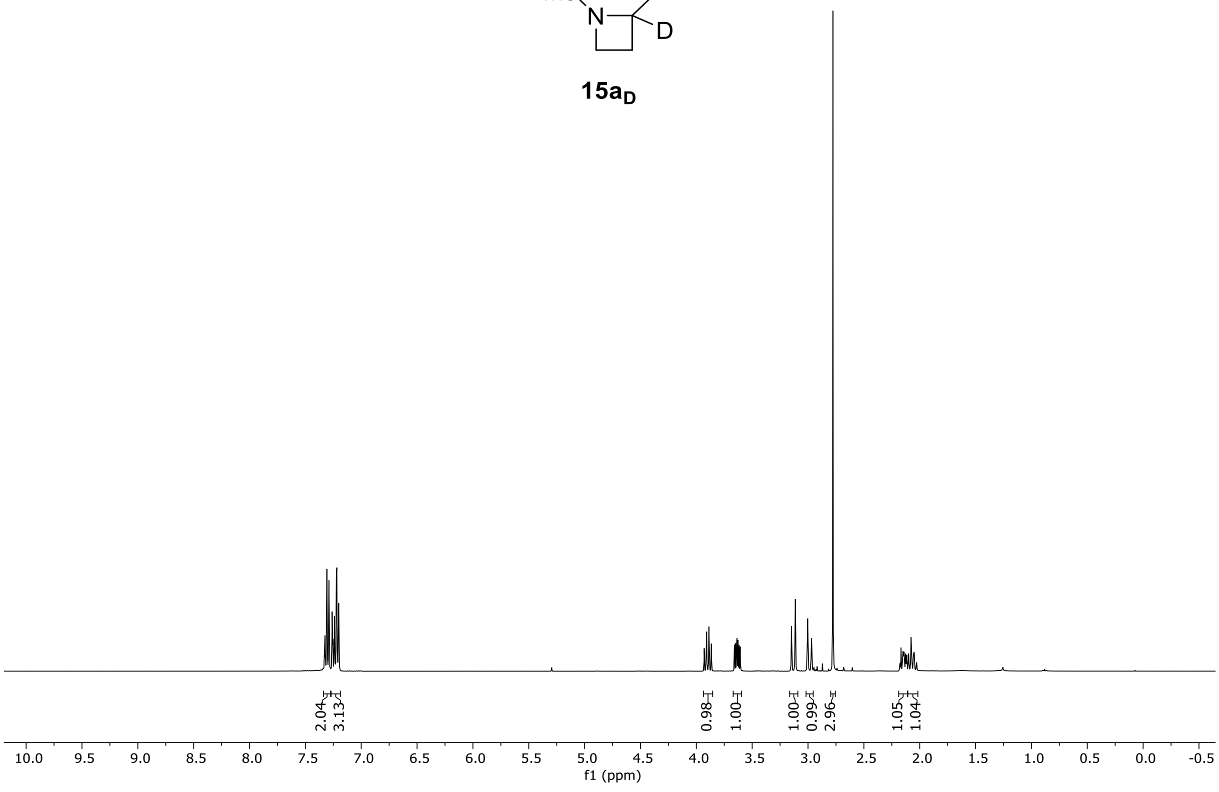

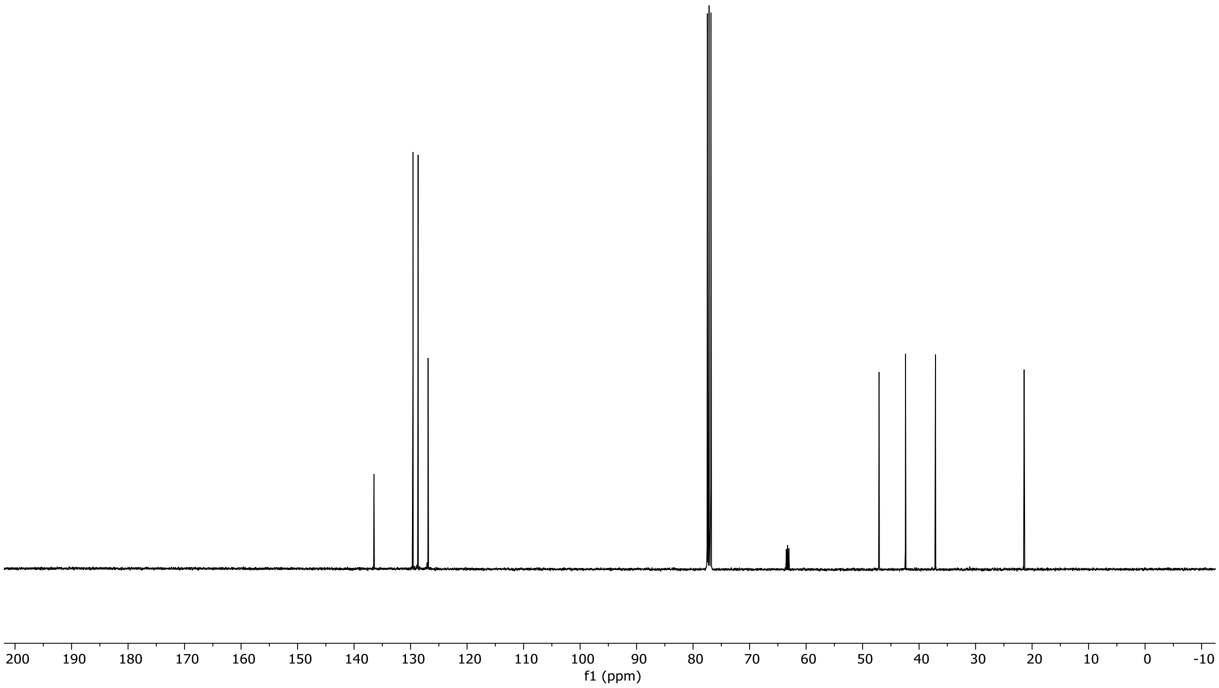

Supplementary Fig 210. ^{1}H (top) and ^{13}C (bottom) NMR spectra of compound **15a$_D$**.

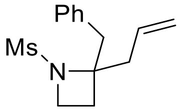

16

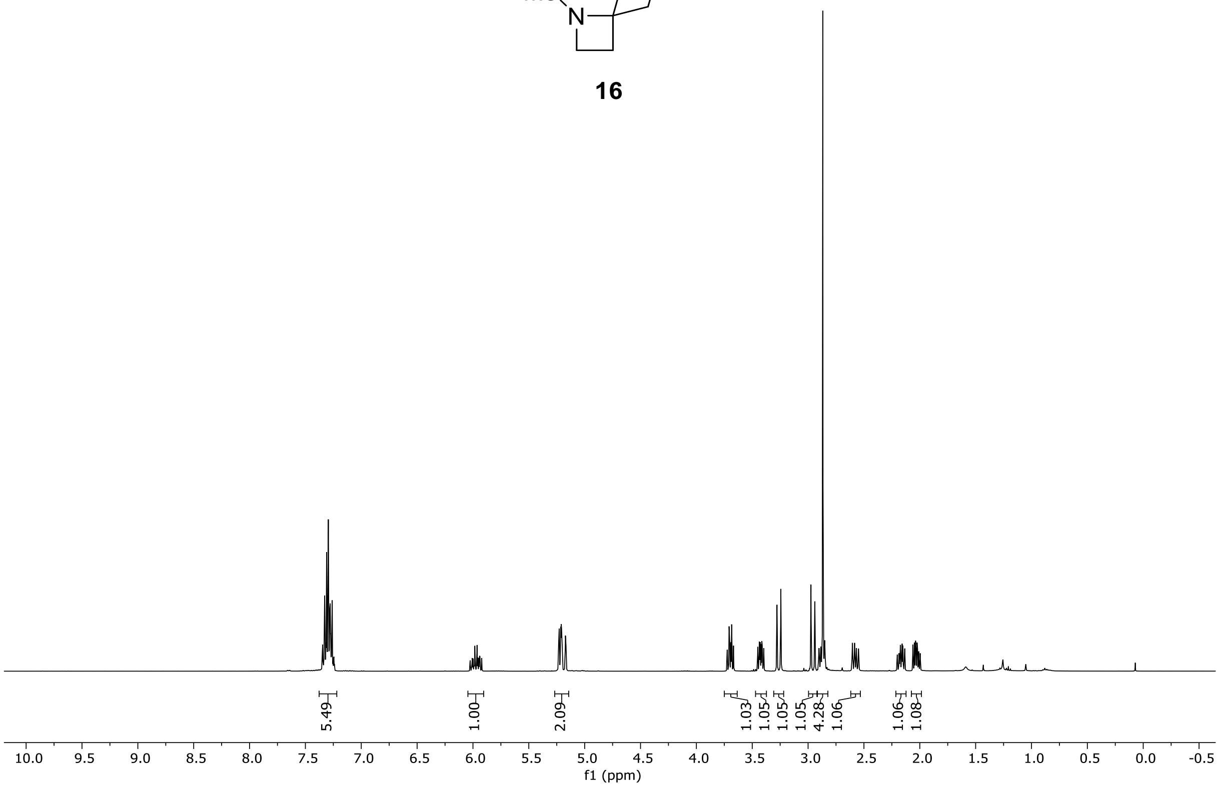

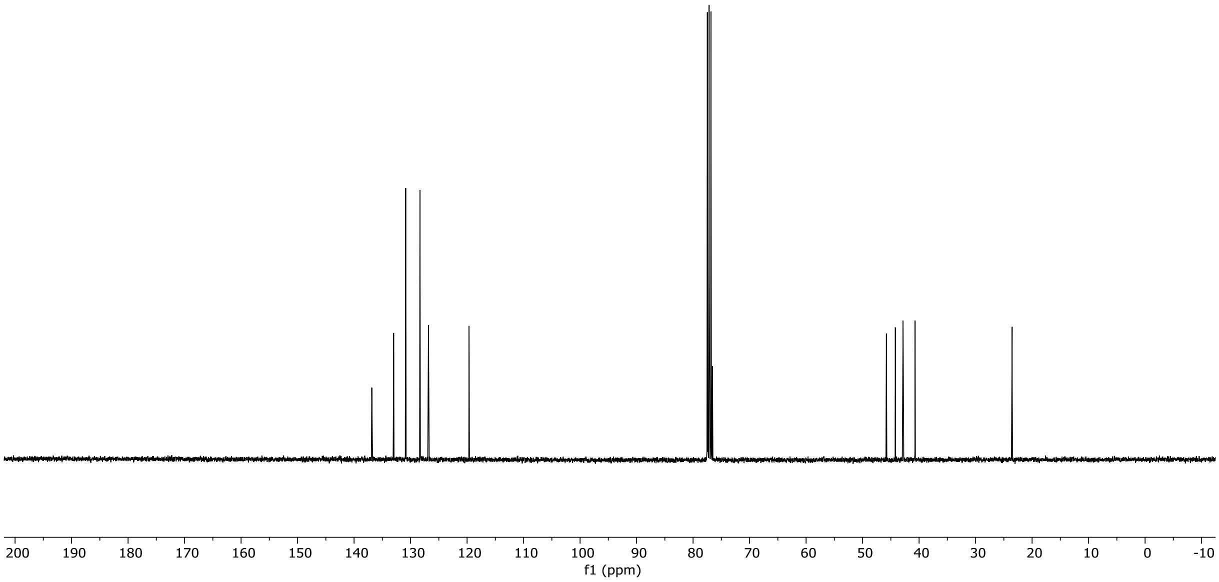

Supplementary Fig 211. ^{1}H (top) and ^{13}C (bottom) NMR spectra of compound **16**.

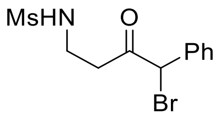

17

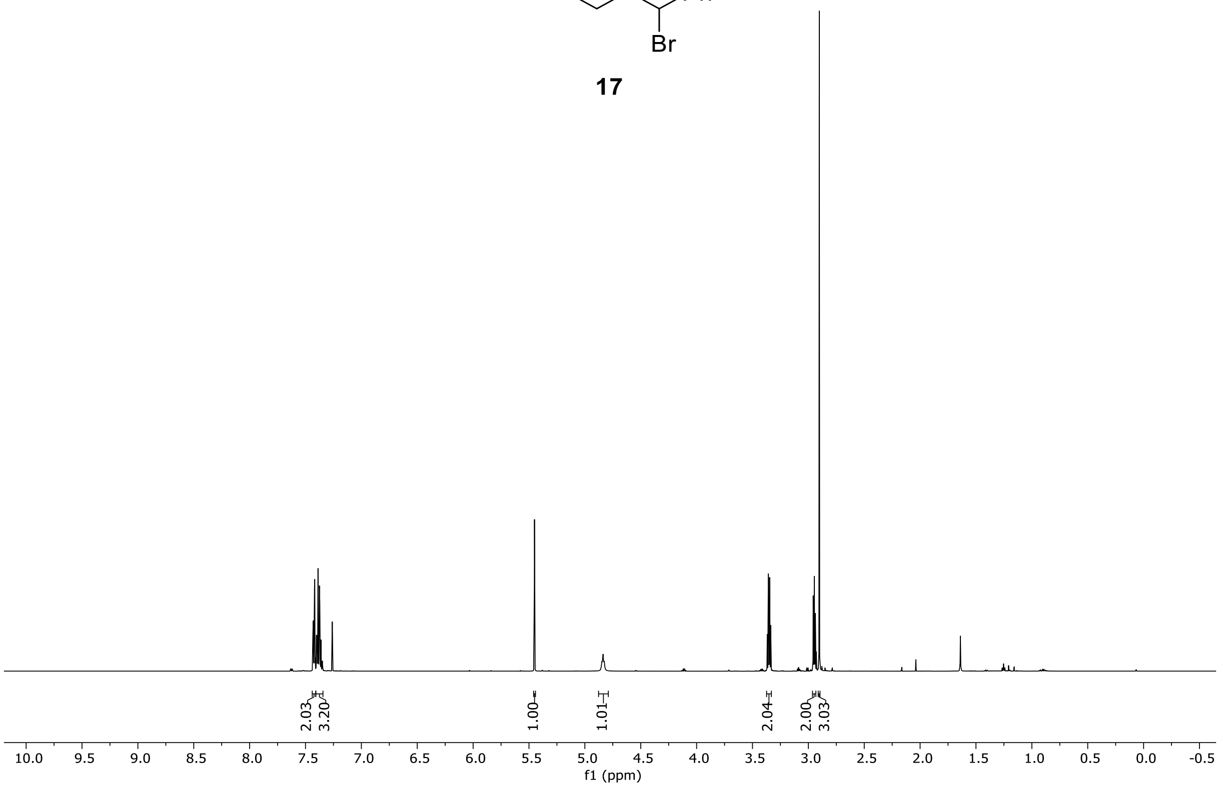

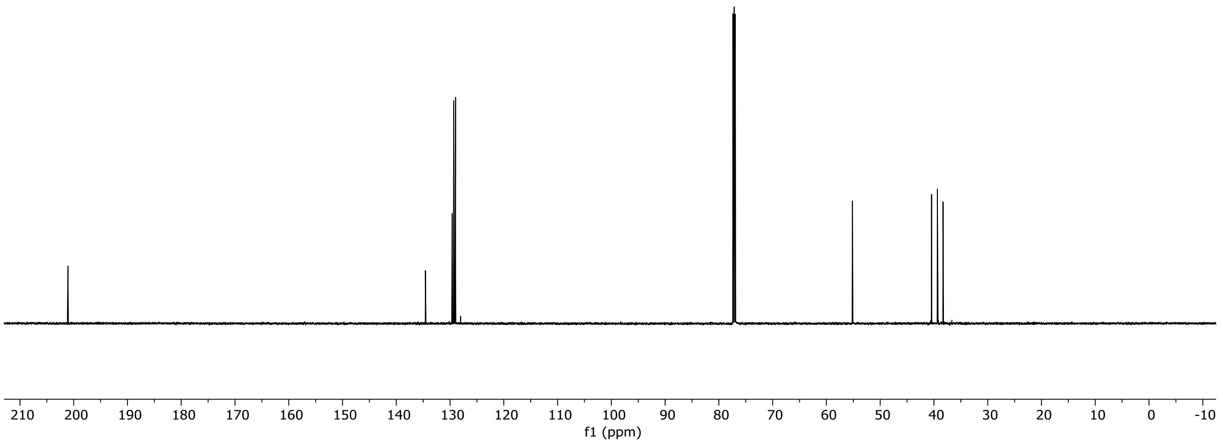

Supplementary Fig 212. ^{1}H (top) and ^{13}C (bottom) NMR spectra of compound **17**.

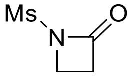

18

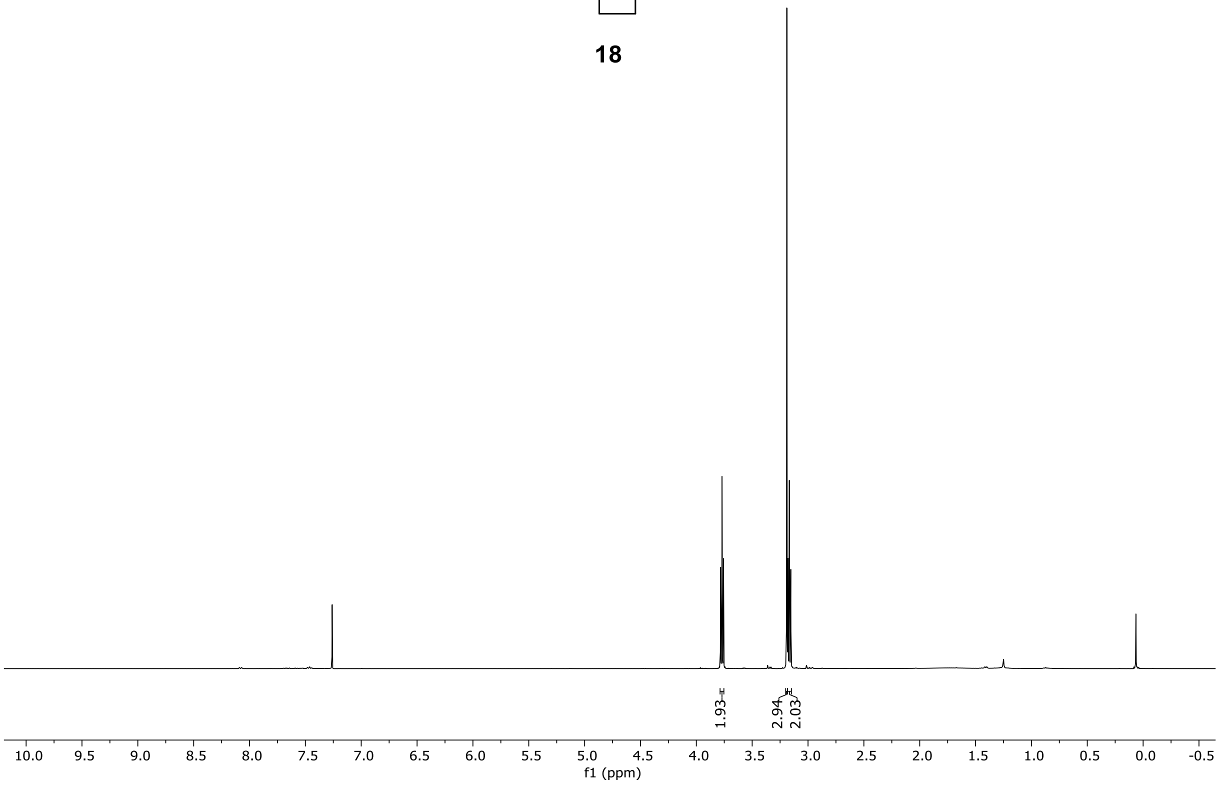

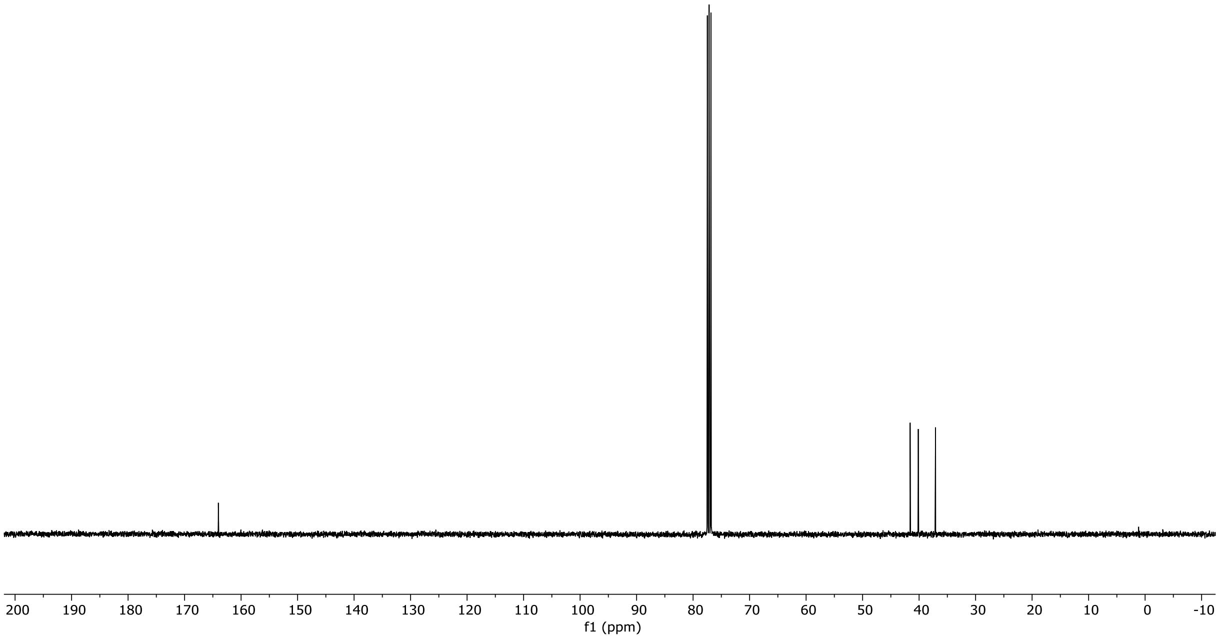

Supplementary Fig 213. ^{1}H (top) and ^{13}C (bottom) NMR spectra of compound **18**.

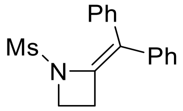

19

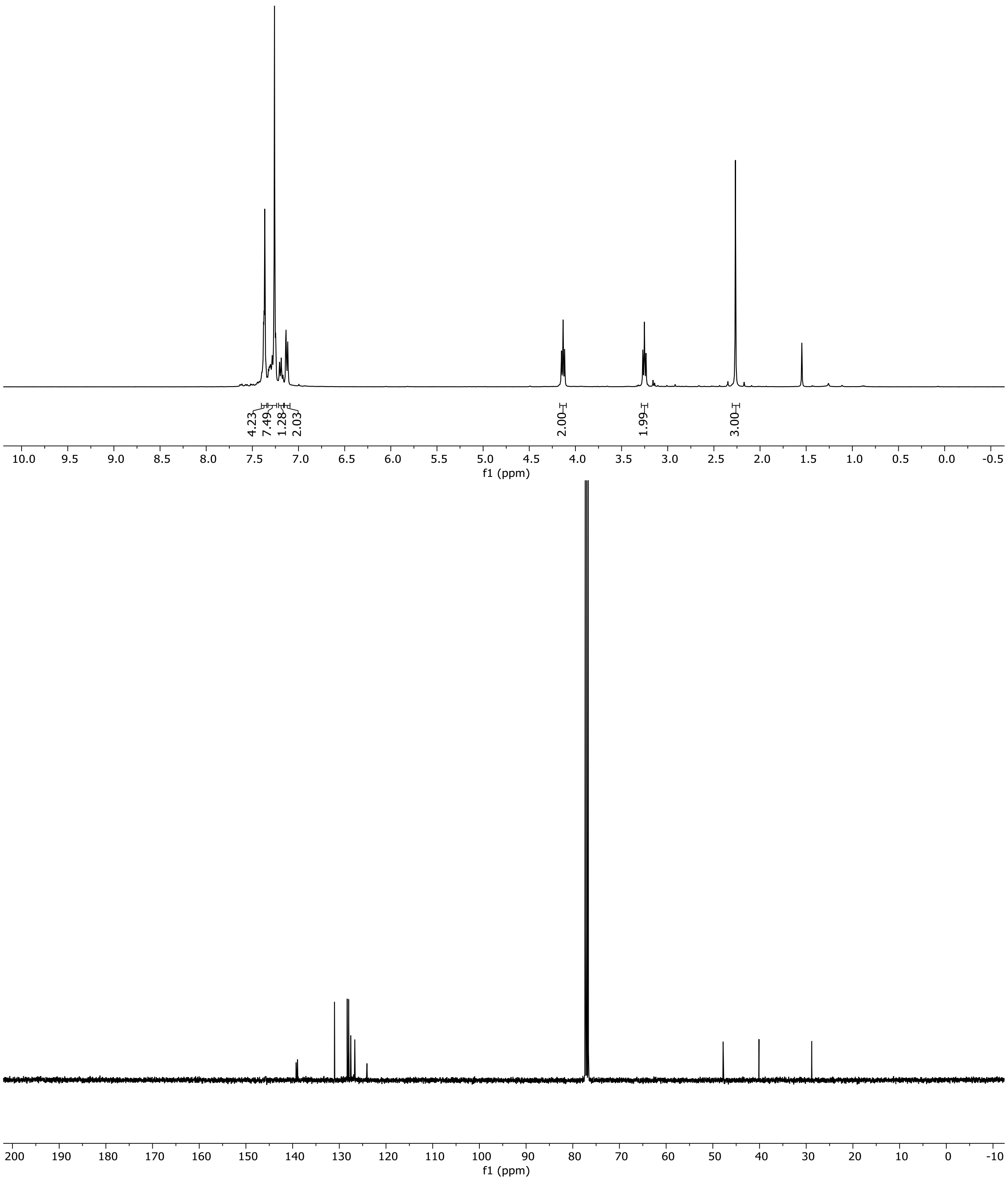

Supplementary Fig 214. ^{1}H (top) and ^{13}C (bottom) NMR spectra of compound **19**.

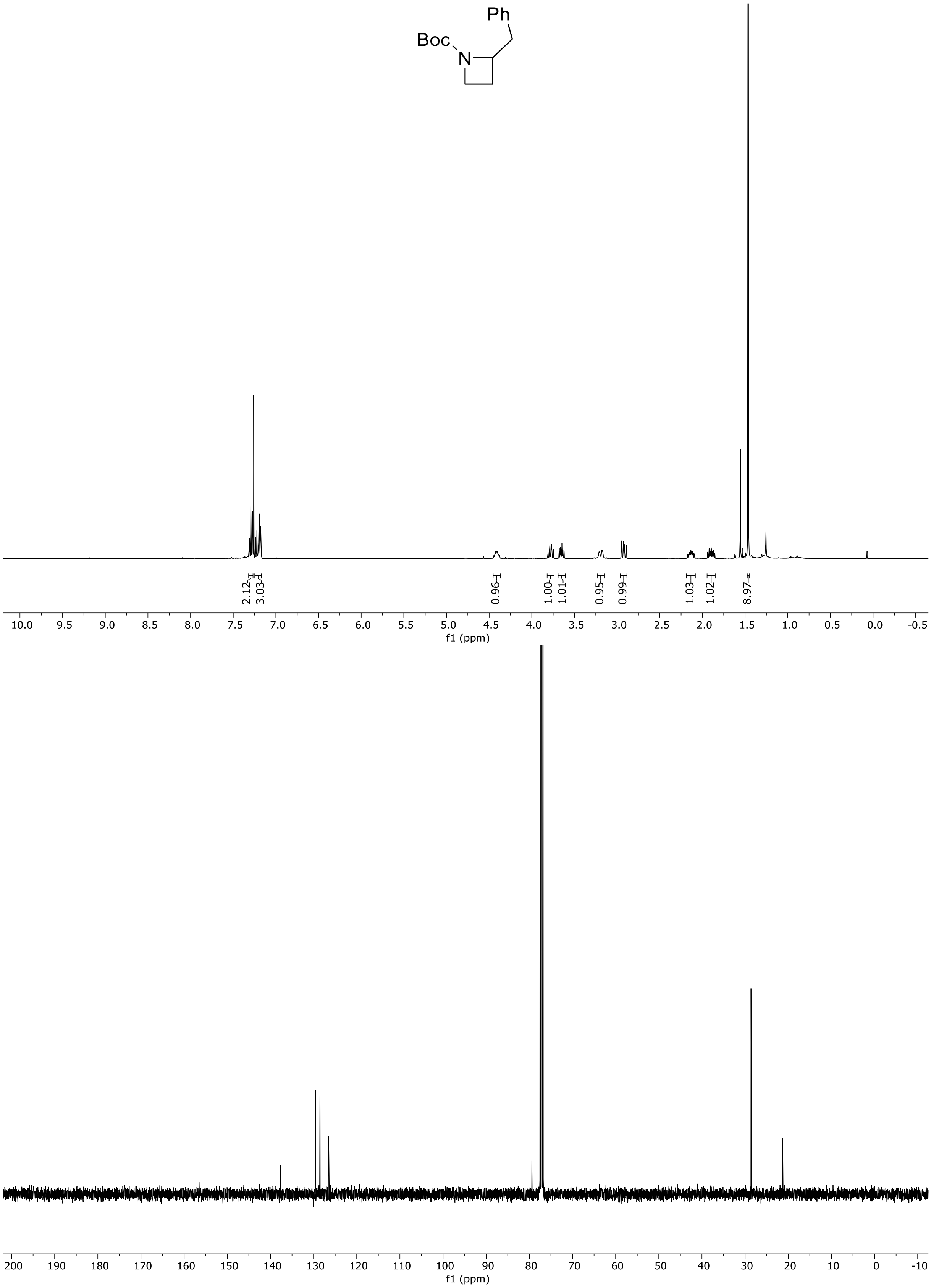

Supplementary Fig 215. ^{1}H (top) and ^{13}C (bottom) NMR spectra of 2-benzyl-1-(*tert*-butoxycarbonyl)azetidine.

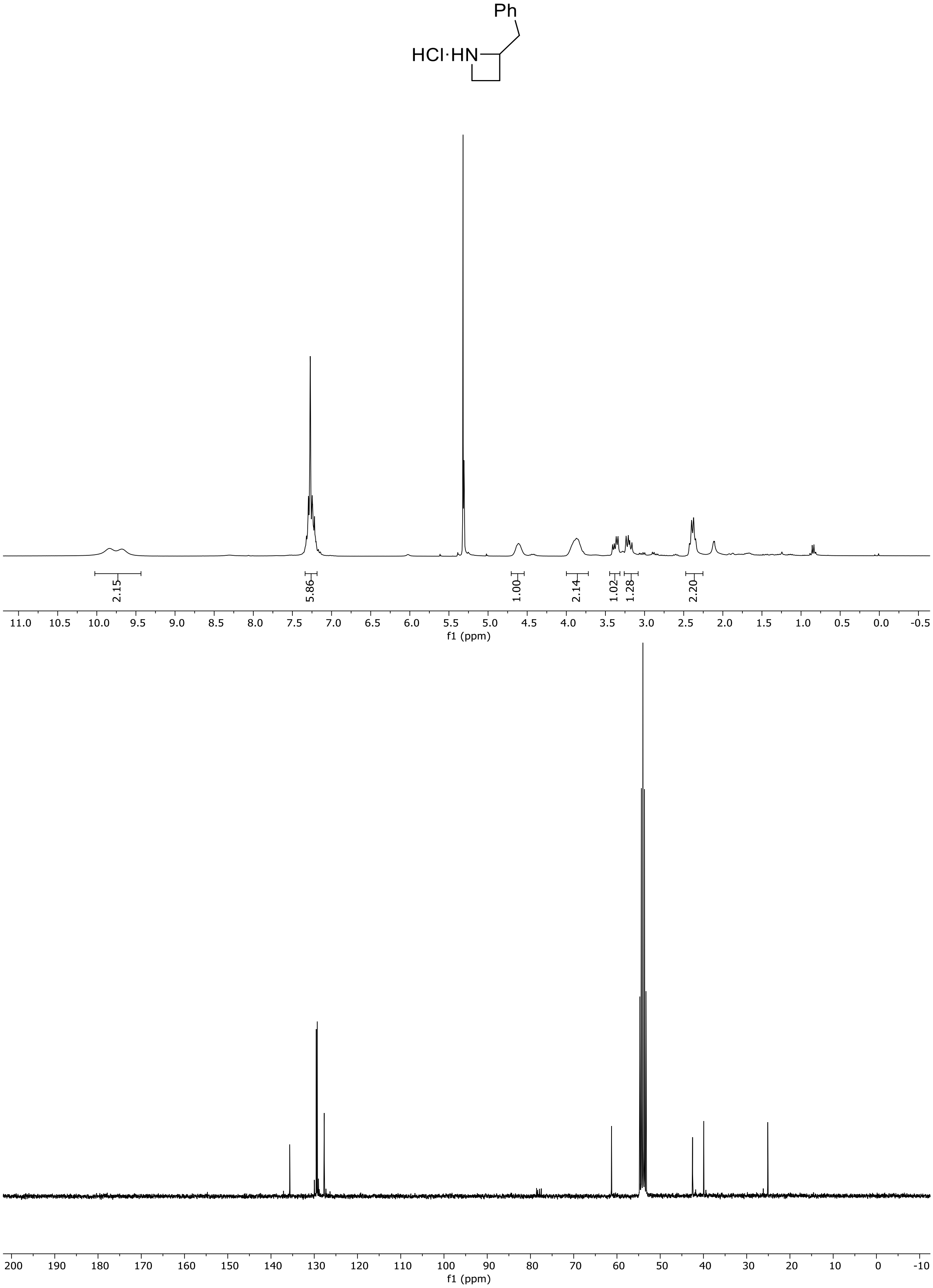

Supplementary Fig 216. ^{1}H (top) and ^{13}C (bottom) NMR spectra of 2-benzylazetidine hydrochloride.

2.15. NOESY Experiments on Azetidines (Z)-2a, (E)-2a, 2b, 15f, 15h and 15i

- **NOESY spectrum of (*Z*)-2-benzylidene-1-(methylsulfonyl)azetidine (*Z*)-2a**

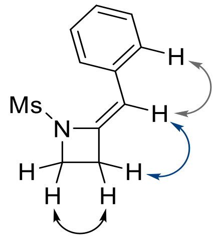

(*Z*)-2a

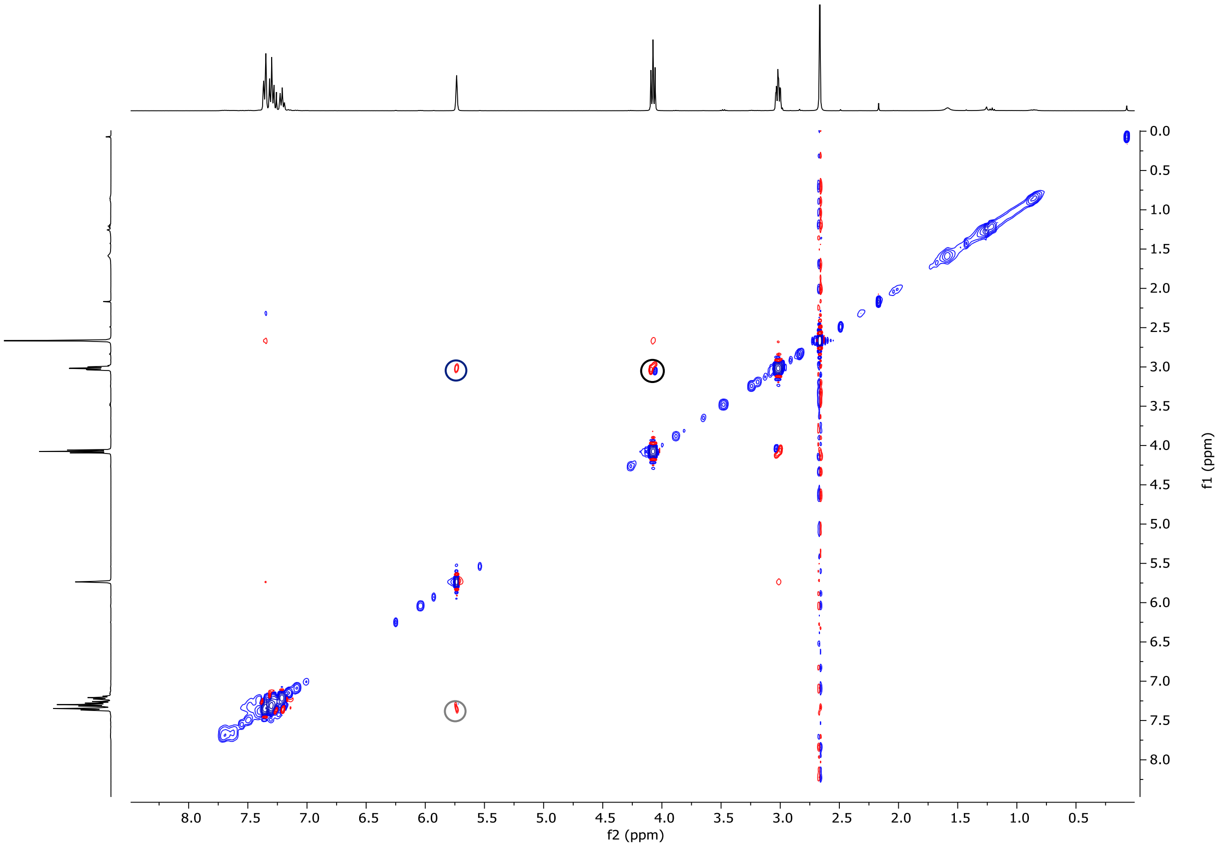

Supplementary Fig 217. NOESY spectrum of (*Z*)-2-benzylidene-1-(methylsulfonyl)azetidine **(*Z*)-2a**.

- **NOESY spectrum of (*E*)-2-benzylidene-1-(methylsulfonyl)azetidine (*E*)-2a**

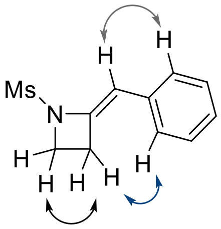

(*E*)-2a

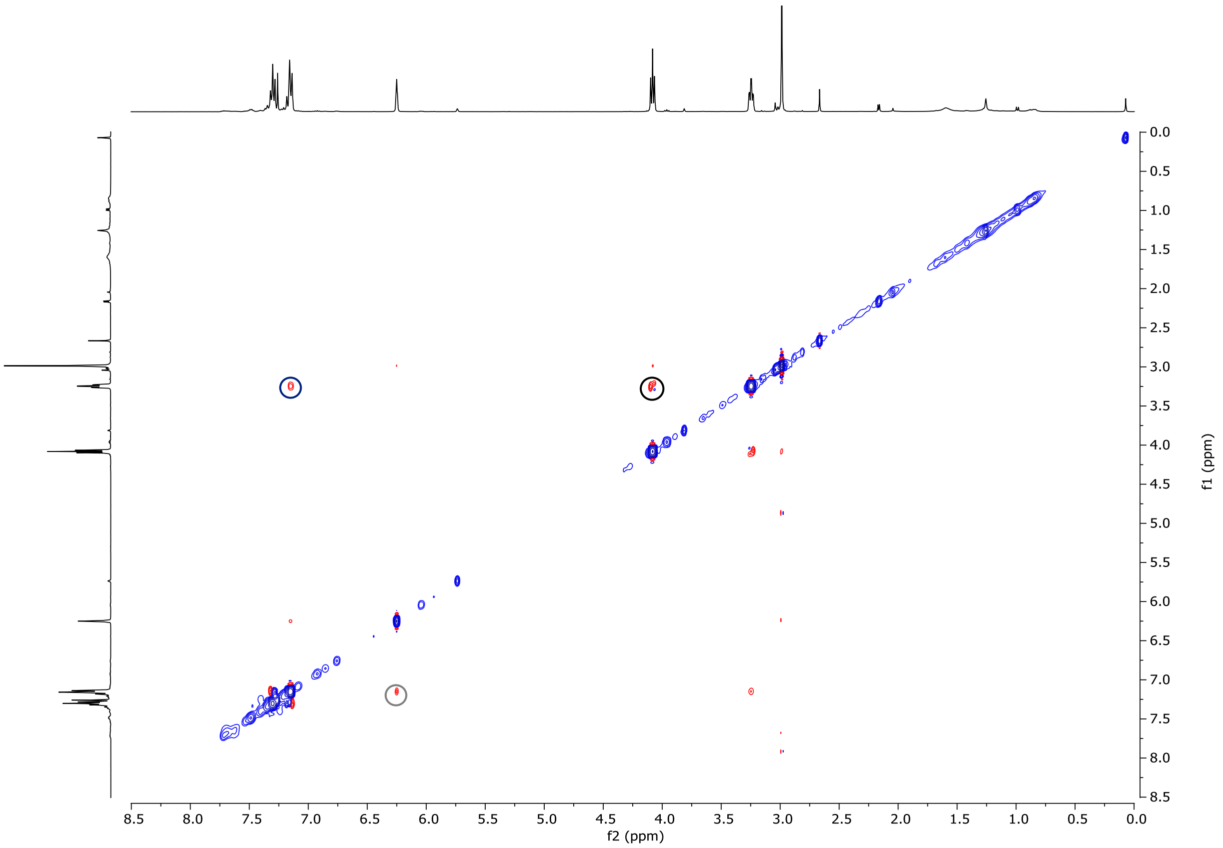

Supplementary Fig 218. NOESY spectrum of (*E*)-2-benzylidene-1-(methylsulfonyl)azetidine **(E)-2a**.

- **NOESY spectrum of the mixture of *Z* and *E* isomers of 2-benzylidene-1-(*tert*-butoxycarbonyl)azetidine 2b**

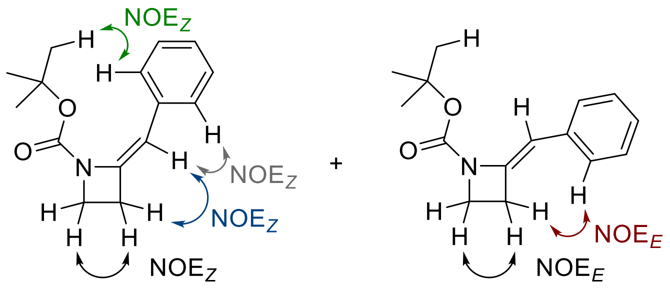

(*Z*)-2b **(*E*)-2b**

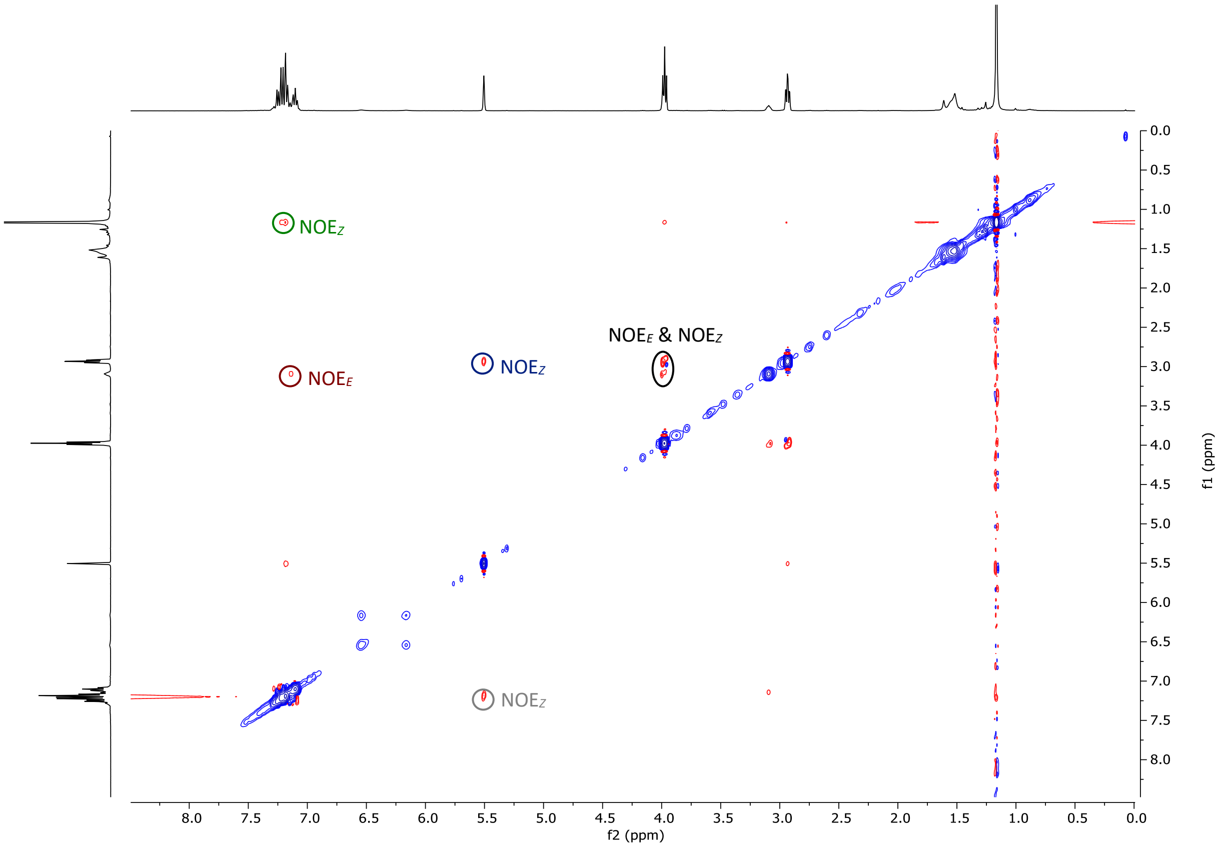

Supplementary Fig 219. NOESY spectrum of the mixture of *Z* and *E* isomers of 2-benzylidene-1-(*tert*-butoxycarbonyl)azetidine **2b**.

- **NOESY spectrum of (2*R*,4*S*)-2-benzyl-4-methyl-1-(methanesulfonyl)azetidine 15f**

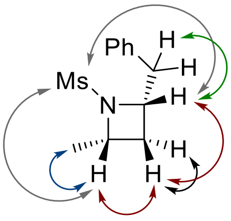

15f

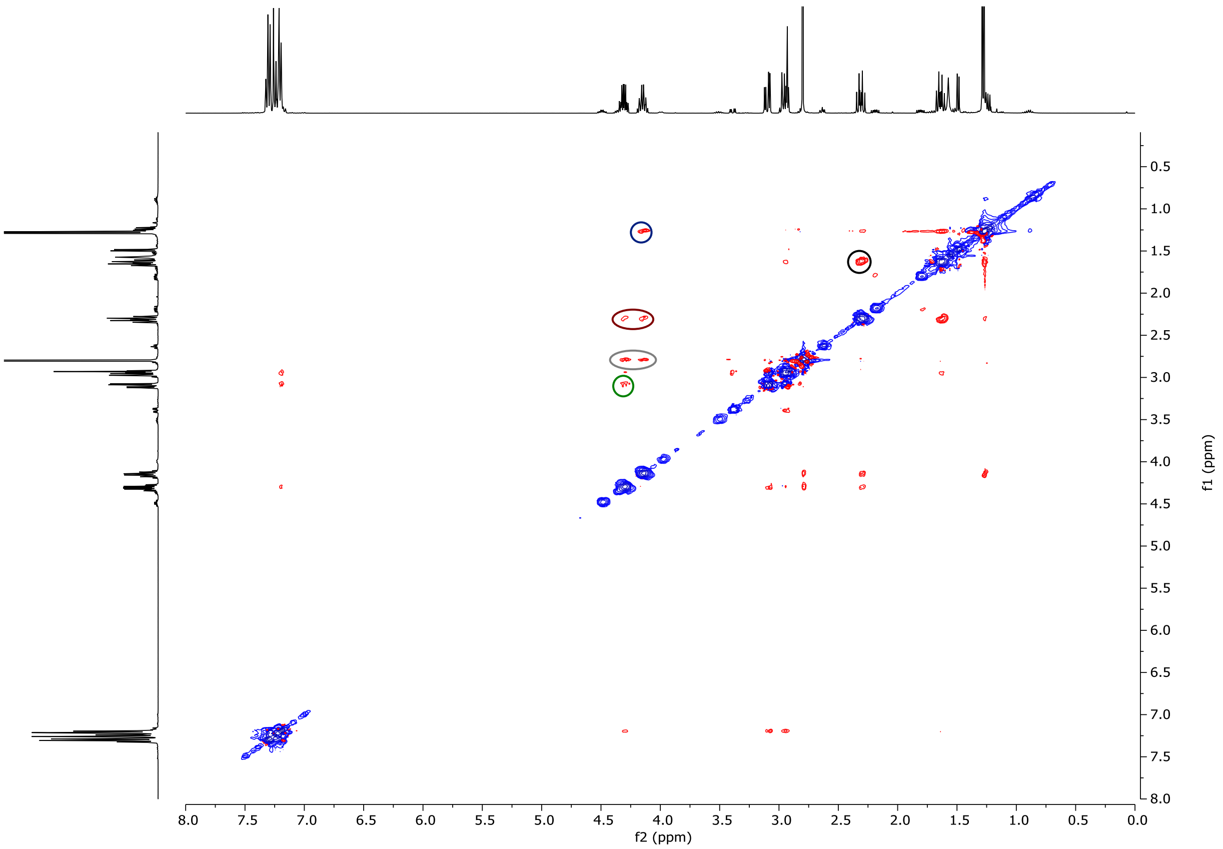

Supplementary Fig 220. NOESY spectrum of (2*R*,4*S*)-2-benzyl-4-methyl-1-(methanesulfonyl)azetidine **15f**.

- **NOESY spectrum of (2*R*,4*S*)-2-benzyl-4-isobutyl-1-(methanesulfonyl)azetidine 15h**

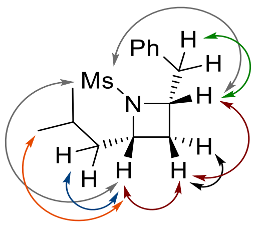

15h

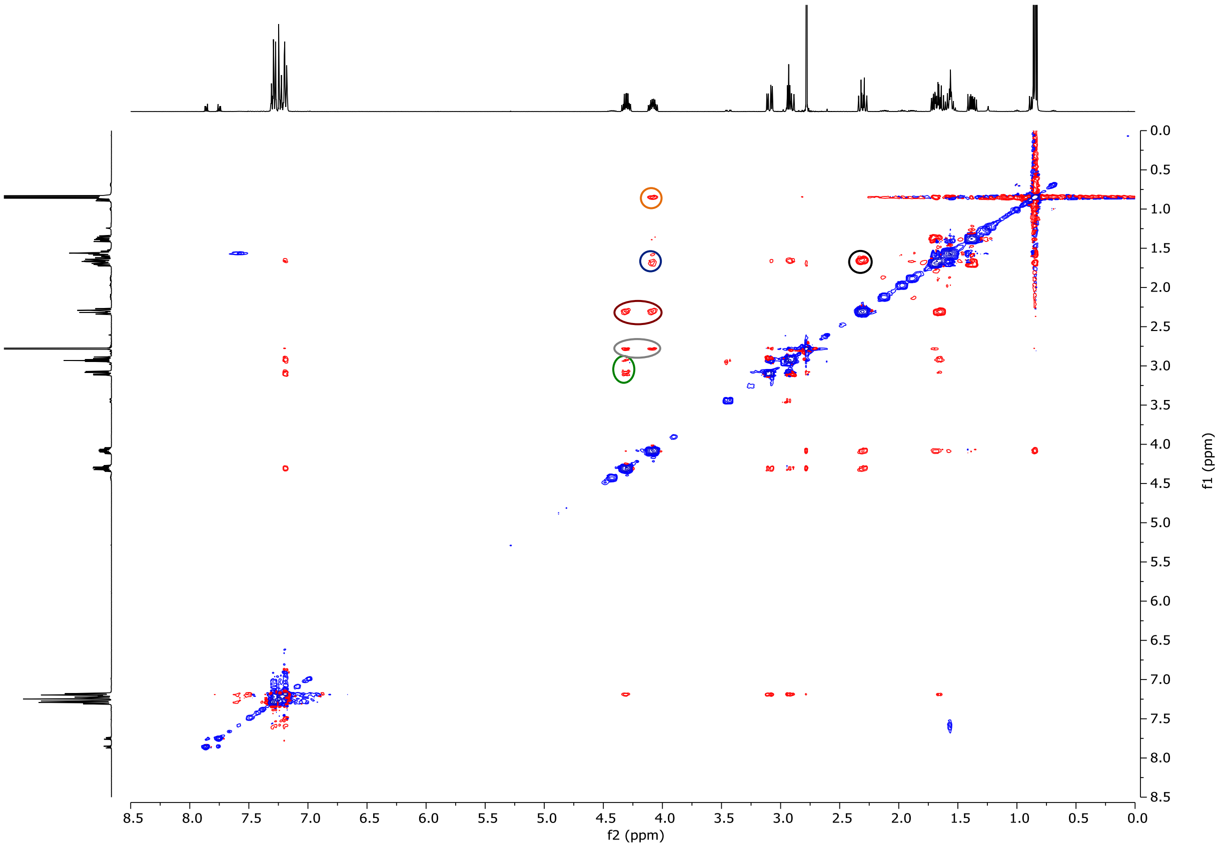

Supplementary Fig 221. NOESY spectrum of (2*R*,4*S*)-2-benzyl-4-isobutyl-1-(methanesulfonyl)azetidine **15h**.

- **NOESY spectrum of (2*R*,4*S*)-2-benzyl-4-[2-(*N*-*tert*-butoxycarbonyl-1*H*-indol-3-yl)ethyl]-1-(methanesulfonyl)azetidine 15i**

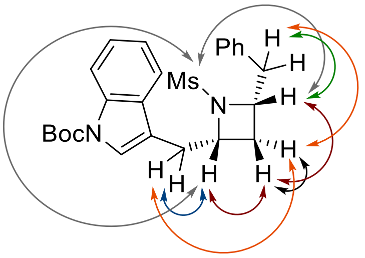

15i

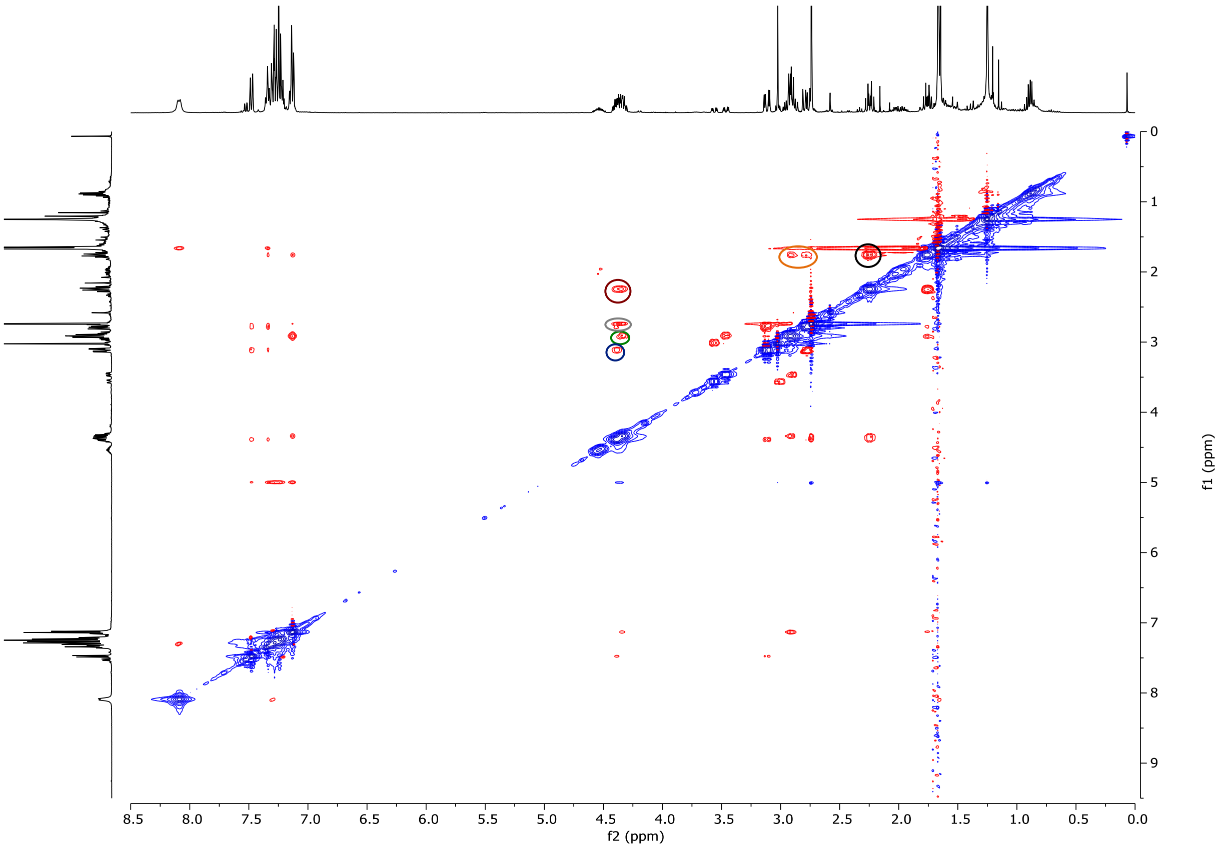

Supplementary Fig 222. NOESY spectrum of (2*R*,4*S*)-2-benzyl-4-[2-(*N*-*tert*-butoxycarbonyl-1*H*-indol-3-yl)ethyl]-1-(methanesulfonyl)azetidine **15i**.

2.16. Computational Studies

Computational Details.

1. DFT Optimizations

Density functional theory calculations were carried out with Gaussian 16 (Revision A.03).[10] Stationary points were characterized at the M052X-D3[11]/def2-TZVP[12] level, with the ultrafine integral grid option invoked. Analytical frequency calculations were carried out at the same level of theory to yield thermodynamic free energy corrections to the SCF energy. Minima along the energy profiles were confirmed with no imaginary frequencies, and saddle points were confirmed with one imaginary frequency constituting the relevant bond formation/breaking process. To characterize and confirm the minima either side of the saddle points, the transition state structures were perturbed along the forward and reverse directions of the imaginary frequency, with the resulting structures subsequently optimized. Single point energy corrections were obtained at the M052X-D3(MeCN)/def2-QZVPP3-5[12-14] level of theory, which was identified as a reliable functional in a benchmarking evaluation of main group kinetics and thermodynamics by Grimme,[15] and has literature precedent in the computational analysis of alkyne ring-closures.[16] Solvation corrections were carried out using the IEFPCM approximation[17-19] with acetonitrile as the solvent (where $\varepsilon = 35.688$). Empirical corrections for dispersion interactions were obtained with the zero-damping function.[20]

2. Conformational sampling

Conformational sampling of transition-states and minima was carried out with the CREST program,[21,22] which interfaces to xtb,[23] and uses a metadynamics-driven search algorithm at the semiempirical GFN2-xTB[24] level of theory to search the conformational space quickly and efficiently. This was particularly important in finding the lowest-energy conformers for the amine-assisted reduction transition-states, as $(i\text{-Pr})_2$NEt has many conformational degrees of freedom. The 20 most stable conformers identified at this GFN2-xTB level of theory in the ensemble were then reoptimized at the M052X-D3/def2-TZVP level and single-point corrected at the M052X-D3(MeCN)/def2-QZVPP level to identify the most stable conformer.

3. Functional testing

In addition to the standard M052X-D3(MeCN)/def2-QZVPP//M052X-D3/def2-TZVP level used throughout, functional testing was also carried out with a series of single-point calculations

(i.e. computed at the "DF",MeCN/def2-QZVPP level using optimized geometries at the M052X-D3/def2-TZVP level). The functionals selected as part of this evaluation were BP86-D3,[25,26] M06L,[27] PBE0-D3,[28,29] PBE-D3,[28] PW6B95-D3,[11] TPSSh-D3,[30] TPSS-D3,[30] and ωB97X-D.[31] A brief discussion of results is presented in Section S3.1.

4. Calculation of atomic charges and bond polarity indices

Computation of both atomic charges and bond polarity indices were carried out with the Multiwfn program (version 3.7).[32] Atomic charges were computed with Truhlar & Cramer's CM5 scheme, [33] and Hirshfeld, [34] Becke, [35] AIM [36] and Natural atomic charges [37] were also tested. All schemes were computed using the built-in atomic densities within Multiwfn. Natural charges were computed via NBO (version 3.1) [38] in Gaussian 16.

Topological analysis with both the Atoms In Molecules (AIM)[36] and Electron Localization Function (ELF) [39,40] density partitioning approaches were used to gain insight into the polarity of the triple bond in the substrates, and thus identify the contributions the N-Ms groups in the ynamides **1a** and **1r**, and the Ph groups in **1a** and **8** had on the overall triple bond polarity. Raub and Jansen proposed a quantitative measure for bond polarity by using both AIM and ELF schemes of partitioning the electron density into atomic and bond sub-basins, and it was this "bond polarity index" that was employed in this study. [41]

$$\boldsymbol{p}_{\mathbf{xy}} = \frac{\mathrm{V(X,Y)|X - V(X,Y)|Y}}{\bar{\mathrm{N}}[\mathrm{V(X,Y)}]}$$

where:

- $\mathrm{V(X,Y)}$ = nomenclature for a bond sub-basin between atoms X and Y
- $\bar{\mathrm{N}}[\mathrm{V(X,Y)}]$ = the total electron density in the X-Y bond sub-basin V(X,Y) identified by the ELF method
- $\mathrm{V(X,Y)|X}$ = electron density from atom X, identified by AIM, that contributes to bond sub-basin V(X,Y).

As an important note, while the reductant of the reaction is postulated to be Hünig's base radical cation (i-Pr)$_2$NEt$^{\bullet+}$, neutral Hünig's base was instead employed in the calculations for a simpler treatment of unpaired electron density of the resulting stationary points. We expected that this is a reasonable model to survey the energetic differences in the reduction of ***4P$_{1a}$*** *resulting from different steric contributions with the amine attacking the difference faces of the transient vinyl radical.*

Optimized Structures of Selected Species

- **4-exo-dig**

TS-4$_{1a}$

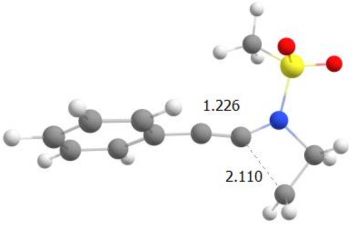

TS-4$_{1r}$

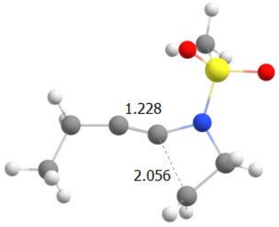

TS-4$_{8}$

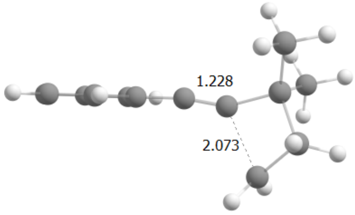

4P$_{1a}$

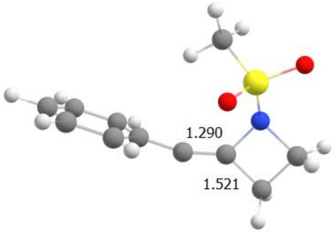

4P$_{1r}$

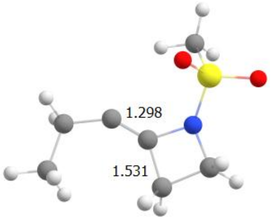

4P$_{8}$

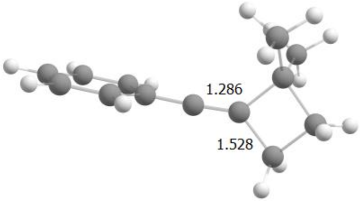

- **5-endo-dig**

TS-5$_{1a}$

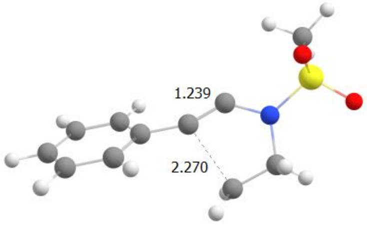

TS-5$_{1r}$

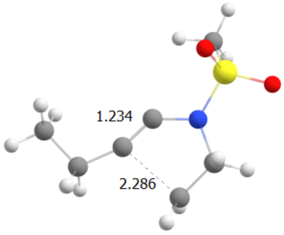

TS-5$_{8}$

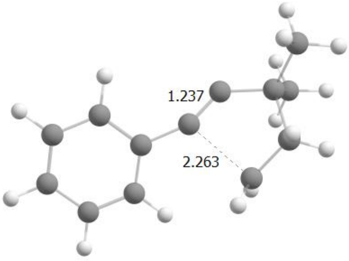

5P$_{1a}$

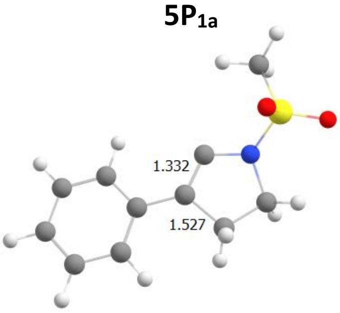

5P$_{1r}$

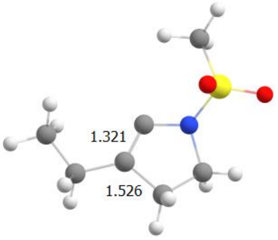

5P$_{8}$

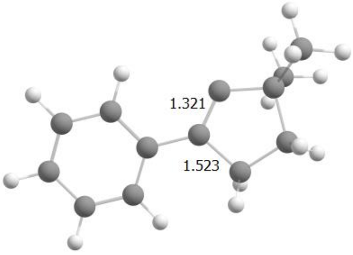

- **Reduction**

TS-Z$_{1a}$

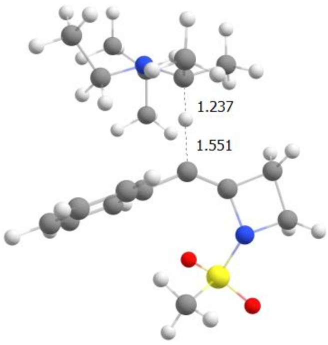

TS-E$_{1a}$

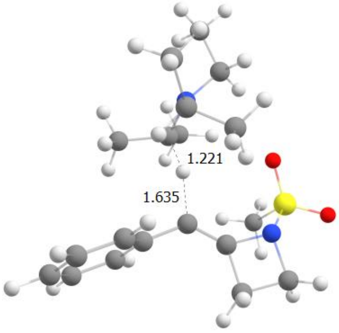

Computed free energy profiles for 4-exo-dig, 5-endo-dig and reduction for 1a, 1r and 8

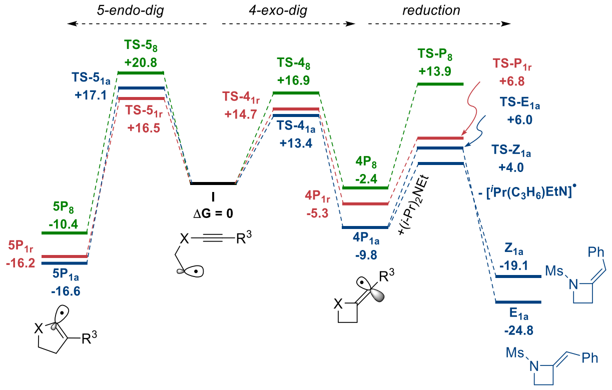

Supplementary Fig 223. Computed free energy profiles of 4-exo-dig, 5-endo-dig and reduction of **1a** (blue), **1r** (red) and **8** (green) from the respective radical species **I** at the M052X-D3,MeCN/def2-QZVPP/M052X-D3/def2-TZVP level of theory.

- **Discussion**

In addition to the computed free energy profiles for 4-exo-dig, 5-endo-dig and reduction for **1a** and **8** already discussed in the manuscript, the free energy profiles for 4-exo-dig, 5-endo-dig and reduction for **1r** have also been computed. Compound **1r** has a similar barrier for 4-exo-dig cyclization as **1a** (+14.7 kcal/mol for **1r**, +13.4 kcal/mol for **1a**) but displays a smaller thermodynamic drive (-5.3 kcal/mol for **1r**, -9.8 kcal+mol for **1a**). Meanwhile, the barrier for the 5-endo-dig cyclization pathway for **1r** is smaller than that for **1a** (+16.5 kcal/mol for **1r**, +17.1 kcal/mol for **1a**) while the thermodynamic drive is similar for both compounds (-16.2 kcal/mol for **1r**, -16.6 kcal/mol for **1a**). In order to rationalize the more favourable reduction of compounds **1r** and **8** over cyclizations, we first computed the barrier for reduction of **4P$_{1r}$** (labelled **TS-P$_{1r}$**, +6.8 kcal/mol). The energetic gap **4P$_{1r}$** - **TS-P$_{1r}$** is +12.1 kcal/mol and so is lower than that for **1a** (**4P$_{1a}$** - **TS-P$_{1a}$** is +15.8 kcal/mol), suggesting that if formation of **4P$_{1r}$** is accessible, onwards reduction should be too, which isn't in agreement with its reduction observed experimentally. As an important note, the chemical model employed for the reduction in the calculations was chosen simply to assess the steric competition between *E* and *Z* alkene formation for **1a**. We are confident that it can reliably be used in this vein to assay the sterics of intermolecular HAT at each face of **4P$_{1a}$** but anticipate that it is too approximate, with respect to experiment for optimal description of the quantum mechanical effects of hydrogen atom transfer (such as PCET), and more advanced levels of theory should be required.

Computed Spin-Densities and Functional Testing Results

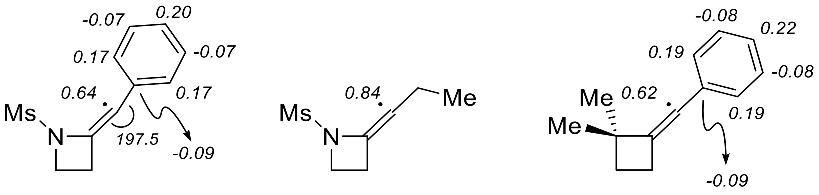

$4P_{1a}$ **$4P_{1r}$** **$4P_{8}$**

Computed atomic spin densities of the product vinyl radicals of 4-exo-dig cyclization, **$4P_{1a}$, $4P_{1r}$** and **$4P_{8}$**, computed at the M052X-D3,MeCN/def2-QZVPP//M052X-D3/def2-TZVP level. The spin density populations at atomic positions were computed by integrating in AIM basins corresponding to the relevant atoms, using MultiWFN.

- **Supplementary Table 1.** Computed partial atomic charges for the three substrates under four different partial atomic schemes.

	**(a) Hirshfeld**			**(b) CM5**			**(c) Natural**			**(d) AIM**		
	**C_1**	**C_2**	**Δq**	**C_1**	**C_2**	**Δq**	**C_1**	**C_2**	**Δq**	**C_1**	**C_2**	**Δq**
**1a**	-0.024	-0.068	**0.043**	0.049	-0.066	**0.116**	0.155	-0.028	**0.183**	0.054	0.168	**-0.114**
**1r**	-0.047	-0.061	**0.014**	0.025	-0.068	**0.093**	0.099	-0.004	**0.104**	0.055	0.117	**-0.062**
**8**	-0.049	-0.077	**0.028**	-0.055	-0.080	**0.025**	0.034	-0.045	**0.080**	-0.204	0.212	**0.008**

- **Supplementary Table 2:** Functional testing of computed kinetics and thermodynamics of 4-exo-dig cyclization, 5-endo-dig cyclization, and **$\Delta\Delta G^{\ddagger}$[Z:E]** of reduction of 4-exo-dig product vinyl radicals **$4P_{1a}$**.

	**1a**				**1r**				**8**				**$4P_{1a}$**
**DF**	**4-exo-dig**		**5-endo-dig**		**4-exo-dig**		**5-endo-dig**		**4-exo-dig**		**5-endo-dig**		**Reduction**
	**$\Delta G^{\ddagger}$**	**ΔG_r**	**$\Delta G^{\ddagger}$**	**ΔG_r**	**$\Delta G^{\ddagger}$**	**ΔG_r**	**$\Delta G^{\ddagger}$**	**ΔG_r**	**$\Delta G^{\ddagger}$**	**ΔG_r**	**$\Delta G^{\ddagger}$**	**ΔG_r**	**$\Delta\Delta G^{\ddagger}$[Z:E]**
**M052X-D3***	**13.4**	**-9.8**	**17.1**	**-16.6**	**14.7**	**-5.3**	**16.5**	**-16.2**	**16.9**	**-2.4**	**20.8**	**-10.4**	**-2.0**
BP86-D3	7.7	-13.8	12.6	-17.8	8.7	-8.3	11.9	-17.0	10.0	-8.5	14.7	-11.8	-1.3
M06L	13.0	-11.2	18.0	-13.2	12.9	-8.3	16.2	-13.7	14.7	-6.0	19.5	-9.2	-0.3
PBE0-D3	11.0	-14.3	16.0	-19.1	11.9	-9.7	15.3	-18.9	13.6	-8.3	18.5	-14.5	-1.6
PBE-D3	7.5	-14.8	12.8	-18.9	8.5	-9.5	12.1	-18.1	9.8	-9.7	15.1	-12.9	-1.2
PW6B95-D3	10.9	-13.1	16.2	-17.0	12.1	-7.8	15.6	-16.3	13.7	-6.8	18.8	-11.7	-1.0
TPSSh-D3	9.4	-13.3	14.2	-17.1	10.5	-8.6	13.7	-16.5	12.3	-6.8	16.9	-11.9	-1.5
TPSS-D3	8.1	-13.3	13.0	-16.8	9.2	-8.4	12.4	-16.1	10.9	-7.2	15.5	-11.1	-1.4
ωB97X-D	12.9	-13.5	17.8	-17.2	13.6	-8.9	17.1	-16.9	16.0	-6.7	20.9	-12.0	-1.7

- **Supplementary Table 3.** Computed bond polarity indices and atomic charges (CM5 scheme) in the V(**C_1-C_2**) triple bond acceptor (**C_1** refers to the C atom α to the N-Ms group in **1a** and **1r**, and α to the CMe_2 group in **8**).

	**$\bar{N}$ [V(C_1-C_2)]**	**V(C_1-C_2)|C_1 (% of total)**	**V(C_1-C_2)|C_2 (% of total)**	**p_{CC}**	**q_{C1}**	**q_{C2}**
**1a**	5.63	3.28 (58)	2.34 (41)	**0.17**	0.049	-0.066
**1r**	5.71	3.29 (57)	2.40 (42)	**0.15**	0.025	-0.068
**8**	5.47	2.72 (50)	2.75 (50)	**0.01**	-0.055	-0.080

- **Supplementary Table 4:** Functional testing of computed atomic charges in CM5 scheme (**C_1-C_2**) and bond polarity index of the C≡C triple bond (***p_{CC}***).

	1a		1r		8	
**DF**	**Δq_{CM5}**	***p_{CC}***	**Δq_{CM5}**	***p_{CC}***	**Δq_{CM5}**	***p_{CC}***
**M052X-D3***	**0.116**	**0.17**	**0.093**	**0.15**	**0.025**	**0.01**
BP86-D3	0.112	0.10	0.093	0.09	0.023	0.00
M06L	0.116	0.13	0.097	0.13	0.021	0.01
PBE0-D3	0.116	0.11	0.082	0.11	0.025	0.01
PBE-D3	0.112	0.10	0.093	0.09	0.023	0.00
PW6B95-D3	0.117	0.13	0.096	0.12	0.023	0.01
TPSSh-D3	0.115	0.12	0.095	0.12	0.023	0.01
TPSS-D3	0.113	0.12	0.095	0.11	0.022	0.00
ωB97X-D	0.119	0.10	0.097	0.10	0.025	0.01

- **Discussion**

Supplementary Table 2 presents the functional dependence on barriers of 4-exo-dig and 5-endo-dig cyclization for each substrate and the barrier height difference of stereodivergent (*Z* vs *E*) reduction. Quantitatively, the barrier heights for 4-exo-dig cyclization and 5-endo-dig cyclization vary significantly with respect to functional, where for instance, the difference in computed barriers of 4-exo-dig cyclization of **1a** between BP86 ($\Delta G^{‡}$ = 7.7 kcal/mol) and M052X ($\Delta G^{‡}$ = 13.4 kcal/mol) is 6.7 kcal/mol. However this functional-dependence is unsurprising given previous benchmarking studies identifying varied performances of functionals for main-group reaction barrier heights, and more broadly overall performances of rungs of Jacob's Ladder in this context.[13] Promisingly, despite this variance the main trends identified and discussed in the main text are qualitatively consistent amongst the functionals selected and are therefore robust, which are: (i) 4-exo-dig as the kinetically favored product, (ii) 5-endo-dig as the theromodynamically favored product and (iii) *Z* isomer product-formation is kinetically favored over *E* in **1a**. Moreover, the computed charges, specifically **Δq_{CM5}** between alkyne carbon atoms, and bond polarity indices (***p_{cc})*** are invariant with respect to functional choice (Supplementary Table 4). What exhibits more variance, however, is the choice of charge scheme (Supplementary Table 1). Despite most schemes computing an order of bond-polarity of **1a** > **1r** > **8** when **Δq** is used as the metric (with exception of the Hirshfeld scheme), quantitatively the charges and polarities were inconsistent, which led to the use of the alternative bond-polarity index first proposed by Raub and Jensen.[41]

Computed Energies for All Species

[(*i*-Pr)(CMe$_2$)NEt]$^{\cdot}$

SCF (M052X-D3/def2-TZVP) Energy = -370.458703794
Enthalpy 0K = -370.195438
Enthalpy 298K = -370.194493
Free Energy 298K = -370.246717
Lowest Frequency = 43.1026 cm^{-1}
Second Frequency = 59.2664 cm^{-1}
SCF (M052X-D3,MeCN/def2-QZVPP) Energy = -370.500569134
SCF (BP86-D3,MeCN/def2-QZVPP) Energy = -370.571735624
SCF (M06L,MeCN/def2-QZVPP) Energy = 370.498114208
SCF (PBE0-D3,MeCN/def2-QZVPP) Energy = -370.106665955
SCF (PBE-D3,MeCN/def2-QZVPP) Energy = -370.047948079
SCF (PW6B95-D3,MeCN/def2-QZVPP) Energy = -371.022699564
SCF (TPSSh-D3,MeCN/def2-QZVPP) Energy = -370.601984651
SCF (TPSS-D3,MeCN/def2-QZVPP) Energy -370.636690994
SCF (wB97XD,MeCN/def2-QZVPP) Energy = -370.440139447

N -0.293223 0.183862 -0.208619
C -1.409857 1.000963 0.247258
H -1.611034 0.842716 1.314463
H -1.135980 2.045035 0.126094
C -2.671688 0.736773 -0.563140
H -2.989924 -0.300666 -0.487154
H -3.488072 1.364160 -0.208063
H -2.486624 0.957882 -1.611676
C 0.960871 0.801196 -0.243339
C -0.367862 -1.205116 0.288422
H -1.424827 -1.387127 0.479195
C 0.081626 -2.234516 -0.740432
H 1.157813 -2.206797 -0.888126
H -0.181032 -3.233815 -0.395573
H -0.406359 -2.052419 -1.695290
C 0.387078 -1.373313 1.604002
H 0.235091 -2.372276 2.009661
H 1.454775 -1.226286 1.444766
H 0.049197 -0.647268 2.342230
C 2.016353 0.277624 -1.155221
H 1.574726 -0.197970 -2.028001
H 2.649512 1.098188 -1.497538
H 2.686301 -0.450459 -0.680032
C 1.297516 1.948994 0.653651
H 1.127631 2.927888 0.189984
H 0.722071 1.923950 1.578214
H 2.355070 1.912606 0.919970

(*i*-Pr)$_2$NEt

SCF (M052X-D3/def2-TZVP) Energy = -371.115571190
Enthalpy 0K = -370.838050
Enthalpy 298K = -370.837106
Free Energy 298K = -370.886493
Lowest Frequency = 58.7248 cm^{-1}
Second Frequency = 91.2339 cm^{-1}
SCF (M052X-D3,MeCN/def2-QZVPP) Energy = -371.158447681
SCF (BP86-D3,MeCN/def2-QZVPP) Energy = -371.226079358

SCF (M06L,MeCN/def2-QZVPP) Energy = 371.153294992
SCF (PBE0-D3,MeCN/def2-QZVPP) Energy = -370.760109105
SCF (PBE-D3,MeCN/def2-QZVPP) Energy = -370.697502717
SCF (PW6B95-D3,MeCN/def2-QZVPP) Energy = -371.679929204
SCF (TPSSh-D3,MeCN/def2-QZVPP) Energy = -371.257455245
SCF (TPSS-D3,MeCN/def2-QZVPP) Energy -371.291196606
SCF (wB97XD,MeCN/def2-QZVPP) Energy = -371.099839251

N -0.001851 0.270621 0.205058
C 0.316719 1.447428 -0.585620
H -0.019667 1.347627 -1.626841
H 1.399276 1.545369 -0.622106
C -0.246794 2.726070 0.019917
H 0.047419 3.588142 -0.576982
H 0.133518 2.849571 1.031482
H -1.333178 2.712022 0.066267
C -1.382452 -0.186175 0.053624
H -1.976700 0.718987 -0.064363
C -1.650124 -1.065671 -1.170955
H -1.318642 -0.583942 -2.089021
H -1.144968 -2.026556 -1.080018
H -2.717978 -1.259837 -1.260074
C -1.871532 -0.874572 1.323072
H -1.704373 -0.230680 2.182969
H -2.934623 -1.097688 1.245530
H -1.350935 -1.816302 1.491604
C 1.023864 -0.774348 0.202372
H 0.532202 -1.684842 0.541628
C 1.660313 -1.076690 -1.159791
H 0.914332 -1.255123 -1.928766
H 2.285705 -1.964751 -1.079350
H 2.298918 -0.257618 -1.486574
C 2.109641 -0.436045 1.218831
H 2.606902 0.495818 0.949982
H 1.672493 -0.314660 2.206616
H 2.865447 -1.219866 1.253904

I_{1a}
SCF (M052X-D3/def2-TZVP) Energy = -1029.81511621
Enthalpy 0K = -1029.588973
Enthalpy 298K = -1029.588029
Free Energy 298K = -1029.649818
Lowest Frequency = 17.4976 cm^{-1}
Second Frequency = 35.8184 cm^{-1}
SCF (M052X-D3,MeCN/def2-QZVPP) Energy = -1029.89825764
SCF (BP86-D3,MeCN/def2-QZVPP) Energy = -1030.05101732
SCF (M06L,MeCN/def2-QZVPP) Energy = 1029.87560782
SCF (PBE0-D3,MeCN/def2-QZVPP) Energy = -1029.11342321
SCF (PBE-D3,MeCN/def2-QZVPP) Energy = -1029.05263719
SCF (PW6B95-D3,MeCN/def2-QZVPP) Energy = -1031.08690309
SCF (TPSSh-D3,MeCN/def2-QZVPP) Energy = -1030.04119418
SCF (TPSS-D3,MeCN/def2-QZVPP) Energy -1030.12194451
SCF (wB97XD,MeCN/def2-QZVPP) Energy = -1029.73946376

C -2.860627 -1.184669 -0.215551
C -2.286494 0.085786 -0.147964

C -4.225567 -1.337639 -0.049524
H -4.661596 -2.323758 -0.101136
C -3.100692 1.194920 0.083083
C -5.032457 -0.231565 0.175709
H -2.654498 2.176108 0.135638
H -6.097418 -0.354631 0.300459
C -4.466130 1.033175 0.238724
H -5.089658 1.896993 0.412843
H -2.226903 -2.038481 -0.399657
C -0.876223 0.238012 -0.312133
C 0.311028 0.325652 -0.476513
N 1.642407 0.431460 -0.647999
C 2.219142 1.791055 -0.736794
H 3.250436 1.678630 -1.053195
H 1.672532 2.279305 -1.548231
C 2.105391 2.550724 0.529340
H 2.860150 3.265608 0.807755
H 1.190880 2.500263 1.098575
S 2.573236 -0.805920 -0.008117
O 1.979482 -2.033255 -0.428684
O 3.926482 -0.476834 -0.337070
C 2.358319 -0.663083 1.736583
H 2.767172 0.290508 2.055019
H 1.296830 -0.738214 1.952228
H 2.901581 -1.491323 2.181840

TS-4$_{1a}$

SCF (M052X-D3/def2-TZVP) Energy = -1029.79477792
Enthalpy 0K = -1029.569453
Enthalpy 298K = -1029.568509
Free Energy 298K = -1029.628465
Lowest Frequency = -662.0023 cm^{-1}
Second Frequency = 28.8493 cm^{-1}
SCF (M052X-D3,MeCN/def2-QZVPP) Energy = -1029.87792358
SCF (BP86-D3,MeCN/def2-QZVPP) Energy = -1030.03979523
SCF (M06L,MeCN/def2-QZVPP) Energy = 1029.85592938
SCF (PBE0-D3,MeCN/def2-QZVPP) Energy = -1029.09689383
SCF (PBE-D3,MeCN/def2-QZVPP) Energy = -1029.04172820
SCF (PW6B95-D3,MeCN/def2-QZVPP) Energy = -1031.07047755
SCF (TPSSh-D3,MeCN/def2-QZVPP) Energy = -1030.02717030
SCF (TPSS-D3,MeCN/def2-QZVPP) Energy -1030.11008435
SCF (wB97XD,MeCN/def2-QZVPP) Energy = -1029.71987305

C 2.941281 0.361737 -1.166095
C 2.095159 0.172513 -0.066636
C 4.287783 0.065724 -1.067520
H 4.933647 0.218797 -1.918789
C 2.630411 -0.326858 1.127550
C 4.809299 -0.428550 0.120647
H 1.972469 -0.478184 1.969296
H 5.860852 -0.660751 0.193420
C 3.976970 -0.624995 1.214811
H 4.380673 -1.011347 2.138319
H 2.526432 0.744545 -2.085753
C 0.713278 0.469070 -0.154717
C -0.449989 0.855835 -0.150268
N -1.810310 0.673868 -0.286464

C -2.443160 1.904253 0.239519
H -3.238630 2.235636 -0.419738
H -2.843712 1.740231 1.236807
C -1.220721 2.777869 0.252190
H -0.823899 3.128326 1.189084
H -0.980689 3.345306 -0.631254
S -2.475732 -0.818167 0.061560
O -1.873052 -1.387755 1.229294
O -3.891299 -0.592534 0.022565
C -1.968019 -1.761096 -1.333962
H -2.310623 -2.775840 -1.154517
H -2.427453 -1.336939 -2.219015
H -0.884131 -1.726879 -1.385569

$4P_{1a}$
SCF (M052X-D3/def2-TZVP) Energy = -1029.83682969
Enthalpy 0K = -1029.608804
Enthalpy 298K = -1029.607860
Free Energy 298K = -1029.666232
Lowest Frequency = 25.7704 cm^{-1}
Second Frequency = 39.8087 cm^{-1}
SCF (M052X-D3,MeCN/def2-QZVPP) Energy = -1029.91920041
SCF (BP86-D3,MeCN/def2-QZVPP) Energy = -1030.07837172
SCF (M06L,MeCN/def2-QZVPP) Energy = 1029.89870343
SCF (PBE0-D3,MeCN/def2-QZVPP) Energy = -1029.14150360
SCF (PBE-D3,MeCN/def2-QZVPP) Energy = -1029.08148257
SCF (PW6B95-D3,MeCN/def2-QZVPP) Energy = -1031.11304049
SCF (TPSSh-D3,MeCN/def2-QZVPP) Energy = -1030.06766995
SCF (TPSS-D3,MeCN/def2-QZVPP) Energy -1030.14851068
SCF (wB97XD,MeCN/def2-QZVPP) Energy = -1029.76631751

C -2.637462 0.838430 1.027151
C -1.738791 0.665941 -0.051821
C -3.876934 0.237871 1.004155
H -4.553259 0.376132 1.834162
C -2.146092 -0.130473 -1.149013
C -4.261776 -0.543468 -0.082044
H -1.452071 -0.279518 -1.961136
H -5.234533 -1.010029 -0.093501
C -3.389123 -0.723557 -1.152071
H -3.684902 -1.335919 -1.990408
H -2.333542 1.448624 1.863620
C -0.488058 1.262553 -0.048609
C 0.786164 1.448517 0.021536
N 1.851112 0.561197 0.441469
C 2.889117 1.466813 -0.132068
H 3.678987 1.691975 0.573507
H 3.303663 1.086351 -1.060910
C 1.788656 2.534013 -0.337750
H 1.692760 2.929313 -1.342141
H 1.819148 3.341899 0.386049
S 1.857992 -1.042164 0.019447
O 1.171609 -1.247280 -1.225590
O 3.223106 -1.463263 0.151502
C 0.880375 -1.748187 1.299146
H 0.805621 -2.808061 1.074416
H 1.379493 -1.577367 2.245730

H -0.101200 -1.283533 1.270208

TS-Z_{1a}
SCF (M052X-D3/def2-TZVP) Energy = -1400.95089877
Enthalpy 0K = -1400.446999
Enthalpy 298K = -1400.446055
Free Energy 298K = -1400.532413
Lowest Frequency = -944.7192 cm^{-1}
Second Frequency = 13.9591 cm^{-1}
SCF (M052X-D3,MeCN/def2-QZVPP) Energy = -1401.07439941
SCF (BP86-D3,MeCN/def2-QZVPP) Energy = -1401.31694300
SCF (M06L,MeCN/def2-QZVPP) Energy = 1401.04942416
SCF (PBE0-D3,MeCN/def2-QZVPP) Energy = -1399.90406548
SCF (PBE-D3,MeCN/def2-QZVPP) Energy = -1399.78830628
SCF (PW6B95-D3,MeCN/def2-QZVPP) Energy = -1402.79124614
SCF (TPSSh-D3,MeCN/def2-QZVPP) Energy = -1401.32841915
SCF (TPSS-D3,MeCN/def2-QZVPP) Energy -1401.44523783
SCF (wB97XD,MeCN/def2-QZVPP) Energy = -1400.86594655

C -0.249796 1.993622 1.481526
C -0.335940 1.165183 0.353869
C -0.179267 3.368801 1.343745
H -0.120760 3.993045 2.222921
C -0.334977 1.758366 -0.915059
C -0.183326 3.945437 0.080218
H -0.423065 1.126263 -1.784195
H -0.126307 5.017870 -0.026947
C -0.263337 3.133823 -1.044670
H -0.276163 3.576965 -2.029278
H -0.243095 1.539545 2.461838
C -0.350075 -0.265026 0.512546
C -1.281038 -1.176068 0.617593
N -2.725723 -1.093446 0.585391
C -2.861839 -2.577934 0.613972
H -3.496128 -2.918438 1.423602
H -3.205742 -2.984693 -0.331244
C -1.334594 -2.677596 0.827168
H -0.804609 -3.262709 0.083534
H -1.042167 -2.990030 1.824835
S -3.592979 -0.241738 -0.539761
O -2.819702 -0.042855 -1.733366
O -4.858143 -0.917278 -0.621212
C -3.828238 1.321015 0.231956
H -4.396876 1.918320 -0.474998
H -4.383232 1.163338 1.149446
H -2.857529 1.769807 0.417969
N 3.159518 -0.617498 0.008447
C 3.220476 0.846174 0.057712
H 2.360917 1.231127 0.601680
H 3.121539 1.245698 -0.949550
C 4.509664 1.359288 0.683421
H 4.616857 0.985199 1.698876
H 4.510207 2.448390 0.711270
H 5.371926 1.025803 0.111018
C 2.233197 -1.220780 0.906813
H 1.083955 -0.838041 0.655753
C 2.170839 -2.734213 0.817926

H 3.168305 -3.159031 0.943643
H 1.538368 -3.108772 1.618770
H 1.758854 -3.084194 -0.125023
C 2.433937 -0.784848 2.348499
H 1.628043 -1.186947 2.959521
H 3.385015 -1.168146 2.721650
H 2.429602 0.295224 2.462167
C 3.260248 -1.192267 -1.335785
H 3.349569 -2.266616 -1.203355
C 2.031227 -0.917740 -2.202805
H 1.944286 0.143916 -2.429675
H 1.116077 -1.228812 -1.700924
H 2.109049 -1.455948 -3.146235
C 4.539359 -0.743092 -2.030706
H 4.532021 0.322652 -2.251208
H 5.405708 -0.964911 -1.411680
H 4.640115 -1.273263 -2.975877

Z_{1a}

SCF (M052X-D3/def2-TZVP) Energy = -1030.50851425
Enthalpy 0K = -1030.267022
Enthalpy 298K = -1030.266078
Free Energy 298K = -1030.321953
Lowest Frequency = 39.5609 cm^{-1}
Second Frequency = 64.9348 cm^{-1}
SCF (M052X-D3,MeCN/def2-QZVPP) Energy = -1030.59072642
SCF (BP86-D3,MeCN/def2-QZVPP) Energy = -1030.74612344
SCF (M06L,MeCN/def2-QZVPP) Energy = 1030.56348571
SCF (PBE0-D3,MeCN/def2-QZVPP) Energy = -1029.80857166
SCF (PBE-D3,MeCN/def2-QZVPP) Energy = -1029.74323465
SCF (PW6B95-D3,MeCN/def2-QZVPP) Energy = -1031.78326888
SCF (TPSSh-D3,MeCN/def2-QZVPP) Energy = -1030.73534755
SCF (TPSS-D3,MeCN/def2-QZVPP) Energy -1030.81450276
SCF (wB97XD,MeCN/def2-QZVPP) Energy = -1030.43973451

C 2.027313 1.066494 -0.998305
C 1.413166 1.055302 0.251914
C 3.148628 0.287560 -1.242010
H 3.611740 0.301436 -2.217243
C 1.950434 0.261543 1.260798
C 3.670372 -0.509516 -0.233868
H 1.462665 0.231429 2.222551
H 4.540539 -1.120255 -0.421651
C 3.070573 -0.518110 1.017614
H 3.469621 -1.140430 1.804290
H 1.611151 1.683365 -1.781448
C 0.205279 1.883868 0.477446
C -1.017841 1.530336 0.124182
N -1.536314 0.343329 -0.481370
C -2.926237 0.892976 -0.481148
H -3.337227 0.955238 -1.482012
H -3.595838 0.341904 0.168261
C -2.368358 2.205257 0.117142
H -2.756437 2.444457 1.102116
H -2.432261 3.073553 -0.530080
S -1.302089 -1.206564 0.079463
O -0.831057 -1.173913 1.433722

O -2.521788 -1.895426 -0.238095
C -0.025769 -1.855157 -0.940682
H 0.089183 -2.890787 -0.632851
H -0.353184 -1.789819 -1.971993
H 0.888932 -1.298958 -0.769295
H 0.326134 2.861966 0.924037

TS-E_{1a}

SCF (M052X-D3/def2-TZVP) Energy = -1400.94686330
Enthalpy 0K = -1400.443287
Enthalpy 298K = -1400.442343
Free Energy 298K = -1400.529839
Lowest Frequency = -581.1941 cm^{-1}
Second Frequency = 15.1800 cm^{-1}
SCF (M052X-D3,MeCN/def2-QZVPP) Energy = -1401.06976923
SCF (BP86-D3,MeCN/def2-QZVPP) Energy = -1401.31345964
SCF (M06L,MeCN/def2-QZVPP) Energy = 1401.04744044
SCF (PBE0-D3,MeCN/def2-QZVPP) Energy = -1399.90010491
SCF (PBE-D3,MeCN/def2-QZVPP) Energy = -1399.78494505
SCF (PW6B95-D3,MeCN/def2-QZVPP) Energy = -1402.78821573
SCF (TPSSh-D3,MeCN/def2-QZVPP) Energy = -1401.32449281
SCF (TPSS-D3,MeCN/def2-QZVPP) Energy -1401.44150773
SCF (wB97XD,MeCN/def2-QZVPP) Energy = -1400.86174729

C -3.011353 -0.082609 0.953227
C -2.310879 -0.635344 -0.124792
C -4.374877 0.143233 0.870786
H -4.899248 0.568879 1.713397
C -3.022901 -0.952550 -1.286638
C -5.067370 -0.175202 -0.288774
H -2.488966 -1.372279 -2.127168
H -6.129515 0.004748 -0.353844
C -4.385532 -0.728619 -1.364853
H -4.919450 -0.983091 -2.268338
H -2.467992 0.164915 1.854084
C -0.878603 -0.831442 -0.045289
C -0.276849 -1.829239 0.554873
N 1.095242 -2.189801 0.773220
C 0.736907 -3.551272 1.237943
H 0.967270 -4.320482 0.504587
H 1.187087 -3.798370 2.191350
C -0.744123 -3.108061 1.242206
H -1.149509 -2.934725 2.234066
H -1.427457 -3.718057 0.662143
S 2.367182 -1.897310 -0.213801
O 2.618780 -0.486356 -0.207552
O 3.385970 -2.824781 0.181675
C 1.806238 -2.327586 -1.831418
H 1.578999 -3.388912 -1.849689
H 0.929749 -1.726770 -2.054839
H 2.619266 -2.099279 -2.513803
N 0.752489 2.414817 -0.002605
C 2.171283 2.731306 -0.052537
H 2.727110 1.943380 -0.551188
H 2.551385 2.755650 0.965649
C 2.441232 4.081453 -0.710045
H 2.089136 4.092653 -1.738596

H 3.509336 4.296880 -0.711083
H 1.925082 4.873385 -0.171314
C 0.125905 1.630014 -1.005717
H -0.111353 0.518287 -0.558827
C -1.249437 2.165824 -1.393969
H -1.896437 2.338595 -0.539028
H -1.113890 3.112297 -1.919043
H -1.755272 1.465727 -2.054666
C 0.943374 1.383986 -2.259596
H 1.159510 2.321141 -2.772359
H 1.872421 0.865218 -2.054090
H 0.348737 0.770723 -2.935036
C 0.142563 2.358388 1.323989
H -0.882469 2.030924 1.167630
C 0.805563 1.330847 2.245253
H 0.950264 0.385677 1.730475
H 0.174257 1.163075 3.117697
H 1.773058 1.679060 2.602230
C 0.104798 3.734984 1.974897
H -0.429215 4.442377 1.343989
H -0.388771 3.679278 2.943962
H 1.114212 4.111368 2.137906

E_{1a}
SCF (M052X-D3/def2-TZVP) Energy = -1030.51332316
Enthalpy 0K = -1030.271621
Enthalpy 298K = -1030.270677
Free Energy 298K = -1030.327103
Lowest Frequency = 28.3050 cm^{-1}
Second Frequency = 46.8787 cm^{-1}
SCF (M052X-D3,MeCN/def2-QZVPP) Energy = -1030.59951868
SCF (BP86-D3,MeCN/def2-QZVPP) Energy = -1030.75492278
SCF (M06L,MeCN/def2-QZVPP) Energy = 1030.57570813
SCF (PBE0-D3,MeCN/def2-QZVPP) Energy = -1029.81872735
SCF (PBE-D3,MeCN/def2-QZVPP) Energy = -1029.75383046
SCF (PW6B95-D3,MeCN/def2-QZVPP) Energy = -1031.79289825
SCF (TPSSh-D3,MeCN/def2-QZVPP) Energy = -1030.74504527
SCF (TPSS-D3,MeCN/def2-QZVPP) Energy -1030.82438360
SCF (wB97XD,MeCN/def2-QZVPP) Energy = -1030.44889450

C -2.717853 0.977605 -0.114573
C -2.022120 -0.228971 -0.220805
C -4.082890 0.989180 0.114435
H -4.602125 1.932967 0.189551
C -2.746012 -1.417338 -0.109788
C -4.786632 -0.200166 0.236470
H -2.223421 -2.358652 -0.203516
H -5.851351 -0.186837 0.412365
C -4.111080 -1.405143 0.118370
H -4.648298 -2.337932 0.201815
H -2.198190 1.914392 -0.233734
C -0.579157 -0.314975 -0.443944
C 0.292229 0.678405 -0.310156
N 1.707118 0.677391 -0.455765
C 1.842306 2.036844 0.115915
H 2.273548 2.039135 1.114010
H 2.392145 2.709702 -0.530462

C 0.297263 2.137093 0.093677
H -0.085731 2.806783 -0.670722
H -0.177375 2.353107 1.046013
S 2.709507 -0.569306 -0.085432
O 2.311404 -1.684862 -0.888039
O 4.035722 -0.033955 -0.142298
C 2.343471 -0.969427 1.596936
H 2.609540 -0.127286 2.227382
H 1.287501 -1.210917 1.671293
H 2.952356 -1.833639 1.844145
H -0.184679 -1.281779 -0.727414

TS-5$_{1a}$
SCF (M052X-D3/def2-TZVP) Energy = -1029.78730877
Enthalpy 0K = -1029.561987
Enthalpy 298K = -1029.561043
Free Energy 298K = -1029.621166
Lowest Frequency = -535.5937 cm^{-1}
Second Frequency = 7.5208 cm^{-1}
SCF (M052X-D3,MeCN/def2-QZVPP) Energy = -1029.87186760
SCF (BP86-D3,MeCN/def2-QZVPP) Energy = -1030.03175369
SCF (M06L,MeCN/def2-QZVPP) Energy = 1029.84779690
SCF (PBE0-D3,MeCN/def2-QZVPP) Energy = -1029.08882464
SCF (PBE-D3,MeCN/def2-QZVPP) Energy = -1029.03304622
SCF (PW6B95-D3,MeCN/def2-QZVPP) Energy = -1031.06196966
SCF (TPSSh-D3,MeCN/def2-QZVPP) Energy = -1030.01935443
SCF (TPSS-D3,MeCN/def2-QZVPP) Energy -1030.10209691
SCF (wB97XD,MeCN/def2-QZVPP) Energy = -1029.71193895

C 2.930212 0.722379 0.869807
C 2.217428 -0.139387 0.035135
C 4.308898 0.639639 0.943048
H 4.853092 1.309168 1.591536
C 2.909890 -1.084472 -0.723422
C 4.990011 -0.307823 0.191605
H 2.353646 -1.745739 -1.369629
H 6.065638 -0.373903 0.253285
C 4.288375 -1.170851 -0.637539
H 4.816766 -1.909215 -1.221244
H 2.389338 1.446686 1.459799
C 0.789016 -0.046910 -0.052400
C -0.405494 -0.377274 -0.045601
N -1.565638 0.211475 -0.459499
C -1.360025 1.696164 -0.602056
H -2.062105 2.056294 -1.348634
H -1.571879 2.176522 0.352058
C 0.064518 1.888643 -0.990352
H 0.648807 2.637715 -0.484306
H 0.367118 1.619729 -1.989318
S -2.994870 -0.249286 0.266205
O -3.998339 0.582550 -0.332007
O -2.860723 -0.279745 1.692344
C -3.154449 -1.902430 -0.312007
H -3.240593 -1.882529 -1.392034
H -2.284109 -2.462381 0.014839
H -4.056107 -2.292582 0.150866

$5P_{1a}$
SCF (M052X-D3/def2-TZVP) Energy = -1029.84811050
Enthalpy 0K = -1029.619044
Enthalpy 298K = -1029.618100
Free Energy 298K = -1029.675705
Lowest Frequency = 23.6268 cm^{-1}
Second Frequency = 31.7582 cm^{-1}
SCF (M052X-D3,MeCN/def2-QZVPP) Energy = -1029.93176963
SCF (BP86-D3,MeCN/def2-QZVPP) Energy = -1030.08652475
SCF (M06L,MeCN/def2-QZVPP) Energy = 1029.90379891
SCF (PBE0-D3,MeCN/def2-QZVPP) Energy = -1029.15102845
SCF (PBE-D3,MeCN/def2-QZVPP) Energy = -1029.08984440
SCF (PW6B95-D3,MeCN/def2-QZVPP) Energy = -1031.12110702
SCF (TPSSh-D3,MeCN/def2-QZVPP) Energy = -1030.07548640
SCF (TPSS-D3,MeCN/def2-QZVPP) Energy -1030.15582897
SCF (wB97XD,MeCN/def2-QZVPP) Energy = -1029.77394718

C 3.051616 1.040978 0.252282
C 2.097488 0.073174 -0.054155
C 4.393780 0.704600 0.343638
H 5.120499 1.465559 0.584814
C 2.519179 -1.243322 -0.259972
C 4.801190 -0.600911 0.127774
H 1.780548 -2.000696 -0.479331
H 5.845882 -0.862632 0.198641
C 3.856207 -1.574965 -0.174629
H 4.166919 -2.595953 -0.337583
H 2.745676 2.061628 0.425178
C 0.688936 0.422102 -0.157397
C -0.345049 -0.381374 -0.398818
N -1.539990 0.233540 -0.664790
C -1.271105 1.711504 -0.647508
H -1.276509 2.063114 -1.674286
H -2.057228 2.216734 -0.096905
C 0.125221 1.832964 -0.006968
H 0.081527 2.141428 1.037239
H 0.735785 2.551523 -0.548850
S -2.876057 -0.266933 0.225960
O -3.975960 0.522367 -0.244777
O -2.567066 -0.279074 1.626325
C -3.042464 -1.925346 -0.332484
H -3.223107 -1.912656 -1.400965
H -2.135351 -2.464488 -0.080064
H -3.893579 -2.330175 0.207319

I_8
SCF (M052X-D3/def2-TZVP) Energy = -504.427455699
Enthalpy 0K = -504.173897
Enthalpy 298K = -504.172953
Free Energy 298K = -504.231079
Lowest Frequency = 2.9401 cm^{-1}
Second Frequency = 49.7964 cm^{-1}
SCF (M052X-D3,MeCN/def2-QZVPP) Energy = -504.477904045
SCF (BP86-D3,MeCN/def2-QZVPP) Energy = -504.547503679
SCF (M06L,MeCN/def2-QZVPP) Energy = 504.452093336
SCF (PBE0-D3,MeCN/def2-QZVPP) Energy = -503.921521856

SCF (PBE-D3,MeCN/def2-QZVPP) Energy = -503.862939308
SCF (PW6B95-D3,MeCN/def2-QZVPP) Energy = -505.192360134
SCF (TPSSh-D3,MeCN/def2-QZVPP) Energy = -504.588762485
SCF (TPSS-D3,MeCN/def2-QZVPP) Energy -504.641908430
SCF (wB97XD,MeCN/def2-QZVPP) Energy = -504.346181690

C 2.259477 -1.190353 0.196466
C 1.553831 -0.022624 -0.094828
C 3.639766 -1.164929 0.292496
H 4.176883 -2.073651 0.518590
C 2.255622 1.167784 -0.287307
C 4.331777 0.021843 0.099620
H 1.707270 2.069458 -0.512383
H 5.408407 0.039056 0.175012
C 3.635914 1.186503 -0.190134
H 4.170083 2.112462 -0.340622
H 1.714136 -2.109500 0.344965
C 0.126618 -0.045011 -0.193711
C -1.072809 -0.061971 -0.275472
C -2.535506 -0.085850 -0.374384
C -3.138404 -0.460591 0.999696
H -4.222902 -0.502020 0.886001
H -2.808018 -1.481820 1.229518
C -2.763489 0.458244 2.098537
H -1.742642 0.791366 2.192863
H -3.464041 0.711736 2.875439
C -3.043272 1.292959 -0.807800
H -2.632683 1.560150 -1.779041
H -4.130568 1.271970 -0.878604
H -2.755444 2.051285 -0.083370
C -2.955197 -1.138484 -1.405426
H -2.544461 -0.897899 -2.383326
H -2.599782 -2.124401 -1.113615
H -4.042201 -1.163315 -1.477943

TS-4$_8$
SCF (M052X-D3/def2-TZVP) Energy = -504.403657574
Enthalpy 0K = -504.151173
Enthalpy 298K = -504.150228
Free Energy 298K = -504.204096
Lowest Frequency = -658.0540 cm^{-1}
Second Frequency = 37.1109 cm^{-1}
SCF (M052X-D3,MeCN/def2-QZVPP) Energy = -504.454121077
SCF (BP86-D3,MeCN/def2-QZVPP) Energy = -504.534705209
SCF (M06L,MeCN/def2-QZVPP) Energy = 504.431855055
SCF (PBE0-D3,MeCN/def2-QZVPP) Energy = -503.903034234
SCF (PBE-D3,MeCN/def2-QZVPP) Energy = -503.850577571
SCF (PW6B95-D3,MeCN/def2-QZVPP) Energy = -505.173754683
SCF (TPSSh-D3,MeCN/def2-QZVPP) Energy = -504.572355942
SCF (TPSS-D3,MeCN/def2-QZVPP) Energy -504.627791745
SCF (wB97XD,MeCN/def2-QZVPP) Energy = -504.323939767

C 2.164768 -1.185358 -0.164658
C 1.455759 0.018963 -0.054550
C 3.546558 -1.186666 -0.149891
H 4.079951 -2.121574 -0.234542
C 2.174592 1.216358 0.067121

C 4.249338 0.004796 -0.026993
H 1.631888 2.145272 0.150425
H 5.328468 -0.000601 -0.016247
C 3.556212 1.203443 0.081444
H 4.097257 2.132839 0.177444
H 1.614497 -2.108599 -0.260560
C 0.042734 0.025971 -0.069995
C -1.174632 0.010084 0.093609
C -2.610109 0.031379 -0.338899
C -3.221687 -0.641523 0.905287
H -4.205548 -0.250422 1.164289
H -3.304514 -1.715189 0.749190
C -2.133712 -0.332336 1.898663
H -1.643539 -1.125552 2.438412
H -2.128842 0.636191 2.374212
C -3.079178 1.481115 -0.456684
H -2.522579 1.996801 -1.236383
H -4.139549 1.501282 -0.706618
H -2.931246 2.014269 0.480575
C -2.836157 -0.714666 -1.646031
H -2.310898 -0.219940 -2.460907
H -2.471602 -1.736831 -1.570610
H -3.900659 -0.737311 -1.879217

4P$_8$
SCF (M052X-D3/def2-TZVP) Energy = -504.437898835
Enthalpy 0K = -504.183132
Enthalpy 298K = -504.182188
Free Energy 298K = -504.235981
Lowest Frequency = 31.5753 cm^{-1}
Second Frequency = 45.9879 cm^{-1}
SCF (M052X-D3,MeCN/def2-QZVPP) Energy = -504.487304039
SCF (BP86-D3,MeCN/def2-QZVPP) Energy = -504.566534570
SCF (M06L,MeCN/def2-QZVPP) Energy = 504.467136934
SCF (PBE0-D3,MeCN/def2-QZVPP) Energy = -503.940279176
SCF (PBE-D3,MeCN/def2-QZVPP) Energy = -503.884007829
SCF (PW6B95-D3,MeCN/def2-QZVPP) Energy = -505.208707552
SCF (TPSSh-D3,MeCN/def2-QZVPP) Energy = -504.605201632
SCF (TPSS-D3,MeCN/def2-QZVPP) Energy -504.658906466
SCF (wB97XD,MeCN/def2-QZVPP) Energy = -504.362418534

C 2.040984 -1.194973 0.130027
C 1.336316 0.034636 0.228175
C 3.399705 -1.207407 -0.083705
H 3.917489 -2.152179 -0.156753
C 2.080456 1.237850 0.101004
C 4.112123 -0.015824 -0.206838
H 1.558165 2.178989 0.174931
H 5.177531 -0.035133 -0.373929
C 3.438785 1.200865 -0.112302
H 3.987099 2.126277 -0.207147
H 1.488110 -2.116579 0.225251
C -0.020412 0.058103 0.442481
C -1.300087 0.056670 0.569097
C -2.451170 -0.030027 -0.452849
C -3.379374 -0.435111 0.725492
H -4.328186 0.091318 0.783407

H -3.557249 -1.507263 0.747851
C -2.285363 -0.007718 1.735151
H -2.037183 -0.707386 2.526571
H -2.463894 0.974770 2.166472
C -2.792618 1.345834 -1.006028
H -1.998678 1.696357 -1.663794
H -3.722713 1.300742 -1.573365
H -2.915043 2.070141 -0.201667
C -2.259217 -1.046342 -1.558367
H -1.442163 -0.741419 -2.211809
H -2.018508 -2.024354 -1.145857
H -3.165540 -1.133618 -2.158195

TS-5$_8$
SCF (M052X-D3/def2-TZVP) Energy = -504.398416803
Enthalpy 0K = -504.145679
Enthalpy 298K = -504.144735
Free Energy 298K = -504.197457
Lowest Frequency = -563.2951 cm^{-1}
Second Frequency = 26.6749 cm^{-1}
SCF (M052X-D3,MeCN/def2-QZVPP) Energy = -504.449384447
SCF (BP86-D3,MeCN/def2-QZVPP) Energy = -504.528607128
SCF (M06L,MeCN/def2-QZVPP) Energy = 504.425634650
SCF (PBE0-D3,MeCN/def2-QZVPP) Energy = -503.896615535
SCF (PBE-D3,MeCN/def2-QZVPP) Energy = -503.843528521
SCF (PW6B95-D3,MeCN/def2-QZVPP) Energy = -505.166924429
SCF (TPSSh-D3,MeCN/def2-QZVPP) Energy = -504.566426547
SCF (TPSS-D3,MeCN/def2-QZVPP) Energy -504.621726655
SCF (wB97XD,MeCN/def2-QZVPP) Energy = -504.317496646

C -2.069826 -1.323585 -0.102671
C -1.331465 -0.146094 0.040473
C -3.451934 -1.283692 -0.109126
H -4.011788 -2.200108 -0.220139
C -2.007116 1.065403 0.172726
C -4.118549 -0.073399 0.025176
H -1.440956 1.976775 0.282982
H -5.197515 -0.045103 0.018606
C -3.391731 1.098348 0.166719
H -3.903544 2.043011 0.272138
H -1.542807 -2.259421 -0.205916
C 0.104781 -0.211501 0.052150
C 1.218514 -0.732604 0.183113
C 2.618176 -0.272321 0.064097
C 2.461993 1.014490 -0.806043
H 3.326918 1.662003 -0.639585
H 2.465771 0.715754 -1.853026
C 1.175038 1.706468 -0.492906
H 0.567379 2.081937 -1.300550
H 1.079098 2.220524 0.451730
C 3.161261 0.081704 1.451053
H 2.543517 0.842204 1.924334
H 3.170046 -0.797427 2.091742
H 4.179867 0.458469 1.360643
C 3.538943 -1.277472 -0.616345
H 3.666434 -2.162489 0.003919
H 3.131142 -1.583627 -1.577382

H 4.517923 -0.826976 -0.779986

5P$_8$

SCF (M052X-D3/def2-TZVP) Energy = -504.454678693
Enthalpy 0K = -504.197942
Enthalpy 298K = -504.196998
Free Energy 298K = -504.248296
Lowest Frequency = 41.1284 cm^{-1}
Second Frequency = 68.2335 cm^{-1}
SCF (M052X-D3,MeCN/def2-QZVPP) Energy = -504.504426509
SCF (BP86-D3,MeCN/def2-QZVPP) Energy = -504.576341447
SCF (M06L,MeCN/def2-QZVPP) Energy = 504.476794235
SCF (PBE0-D3,MeCN/def2-QZVPP) Energy = -503.954616700
SCF (PBE-D3,MeCN/def2-QZVPP) Energy = -503.893560072
SCF (PW6B95-D3,MeCN/def2-QZVPP) Energy = -505.220935851
SCF (TPSSh-D3,MeCN/def2-QZVPP) Energy = -504.617655469
SCF (TPSS-D3,MeCN/def2-QZVPP) Energy -504.669617271
SCF (wB97XD,MeCN/def2-QZVPP) Energy = -504.375290851

C -1.808102 -1.252163 -0.076345
C -1.246588 0.026138 -0.047027
C -3.177586 -1.421281 -0.024284
H -3.596736 -2.416075 -0.049182
C -2.095061 1.126639 0.032720
C -4.016846 -0.315716 0.057531
H -1.678794 2.122507 0.058787
H -5.087298 -0.449011 0.096877
C -3.471195 0.956194 0.086307
H -4.115553 1.820208 0.149868
H -1.151321 -2.107674 -0.144418
C 0.204827 0.201024 -0.092340
C 1.150052 -0.719744 -0.026513
C 2.575562 -0.269408 0.026566
C 2.367648 1.194630 -0.457250
H 3.076516 1.872278 0.014781
H 2.528480 1.226213 -1.533390
C 0.897519 1.556160 -0.158662
H 0.474283 2.197549 -0.929742
H 0.787513 2.073296 0.796477
C 3.088964 -0.306831 1.467976
H 2.459395 0.301921 2.115058
H 3.087182 -1.326044 1.849694
H 4.108523 0.076250 1.509200
C 3.509194 -1.051510 -0.887454
H 3.620135 -2.076402 -0.537470
H 3.122567 -1.073633 -1.904318
H 4.494773 -0.586170 -0.899565

I$_{1r}$

SCF (M052X-D3/def2-TZVP) Energy = -877.335560963
Enthalpy 0K = -877.136186
Enthalpy 298K = -877.135242
Free Energy 298K = -877.193251
Lowest Frequency = 23.3405 cm^{-1}
Second Frequency = 30.5372 cm^{-1}
SCF (M052X-D3,MeCN/def2-QZVPP) Energy = -877.407821433

SCF (BP86-D3,MeCN/def2-QZVPP) Energy = -877.545365984
SCF (M06L,MeCN/def2-QZVPP) Energy = 877.401358324
SCF (PBE0-D3,MeCN/def2-QZVPP) Energy = -876.792602774
SCF (PBE-D3,MeCN/def2-QZVPP) Energy = -876.735413884
SCF (PW6B95-D3,MeCN/def2-QZVPP) Energy = -878.381533486
SCF (TPSSh-D3,MeCN/def2-QZVPP) Energy = -877.529544428
SCF (TPSS-D3,MeCN/def2-QZVPP) Energy -877.590812928
SCF (wB97XD,MeCN/def2-QZVPP) Energy = -877.307812763

C 1.960122 0.151109 -0.428306
C 0.793468 0.375809 -0.262678
N -0.528494 0.624466 -0.112350
C -0.917269 1.911563 0.508994
H -2.007985 1.920208 0.521375
H -0.569703 1.941671 1.542023
C -0.366532 3.038627 -0.272103
H -0.072007 3.948056 0.220536
H -0.340587 2.982352 -1.346815
S -1.493377 -0.721313 0.150086
O -0.906396 -1.575695 1.136123
O -2.821227 -0.214526 0.337985
C -1.383405 -1.526955 -1.410861
H -1.928514 -2.459969 -1.304555
H -1.835612 -0.886407 -2.158938
H -0.336237 -1.716888 -1.622117
C 3.385699 -0.141605 -0.555461
H 3.532838 -0.827244 -1.389686
H 3.903658 0.780485 -0.818505
C 3.978229 -0.733787 0.724207
H 3.864390 -0.037274 1.550633
H 3.466038 -1.655574 0.986903
H 5.036320 -0.946478 0.588611

TS-4$_{1r}$
SCF (M052X-D3/def2-TZVP) Energy = -877.313663939
Enthalpy 0K = -877.115109
Enthalpy 298K = -877.114165
Free Energy 298K = -877.169561
Lowest Frequency = -702.4059 cm^{-1}
Second Frequency = 29.4240 cm^{-1}
SCF (M052X-D3,MeCN/def2-QZVPP) Energy = -877.386217266
SCF (BP86-D3,MeCN/def2-QZVPP) Energy = -877.533291495
SCF (M06L,MeCN/def2-QZVPP) Energy = 877.382571123
SCF (PBE0-D3,MeCN/def2-QZVPP) Energy = -876.775419069
SCF (PBE-D3,MeCN/def2-QZVPP) Energy = -876.723651146
SCF (PW6B95-D3,MeCN/def2-QZVPP) Energy = -878.364050769
SCF (TPSSh-D3,MeCN/def2-QZVPP) Energy = -877.514597200
SCF (TPSS-D3,MeCN/def2-QZVPP) Energy -877.577929370
SCF (wB97XD,MeCN/def2-QZVPP) Energy = -877.287944190

C 1.726936 -0.513825 -0.252444
C 0.814382 0.306958 -0.290551
N -0.528262 0.640796 -0.372780
C -0.583453 2.078292 -0.027286
H -1.228663 2.615819 -0.714087
H -0.934964 2.224294 0.990849
C 0.888311 2.358193 -0.173591

H 1.458147 2.654456 0.690762
H 1.253880 2.683875 -1.133616
S -1.688891 -0.411360 0.194042
O -1.249590 -1.073822 1.385426
O -2.895855 0.363744 0.205572
C -1.746587 -1.600864 -1.101431
H -2.433938 -2.375636 -0.775257
H -2.098385 -1.109641 -2.001016
H -0.746548 -2.003774 -1.228647
C 2.996092 -1.216876 -0.115932
H 2.883632 -2.006985 0.627037
H 3.259146 -1.697477 -1.057811
C 4.119091 -0.264724 0.314291
H 4.248936 0.524837 -0.421537
H 3.878204 0.192998 1.270098
H 5.055563 -0.808893 0.411681

4P$_{1r}$
SCF (M052X-D3/def2-TZVP) Energy = -877.349401861
Enthalpy 0K = -877.147677
Enthalpy 298K = -877.146733
Free Energy 298K = -877.200619
Lowest Frequency = 32.1032 cm^{-1}
Second Frequency = 37.5672 cm^{-1}
SCF (M052X-D3,MeCN/def2-QZVPP) Energy = -877.422758620
SCF (BP86-D3,MeCN/def2-QZVPP) Energy = -877.565127522
SCF (M06L,MeCN/def2-QZVPP) Energy = 877.421065986
SCF (PBE0-D3,MeCN/def2-QZVPP) Energy = -876.814461159
SCF (PBE-D3,MeCN/def2-QZVPP) Energy = -876.757002348
SCF (PW6B95-D3,MeCN/def2-QZVPP) Energy = -878.400513596
SCF (TPSSh-D3,MeCN/def2-QZVPP) Energy = -877.549682286
SCF (TPSS-D3,MeCN/def2-QZVPP) Energy -877.610730634
SCF (wB97XD,MeCN/def2-QZVPP) Energy = -877.328523844

C 1.553127 -0.728477 -0.305618
C 0.879884 0.380807 -0.331466
N -0.524207 0.629285 -0.528526
C -0.386418 2.050803 -0.101824
H -0.827953 2.742287 -0.808634
H -0.777532 2.223926 0.896746
C 1.148709 1.875256 -0.138638
H 1.673730 2.137220 0.772356
H 1.624298 2.338762 -0.996947
S -1.695049 -0.301074 0.186303
O -1.263378 -0.790073 1.464735
O -2.897370 0.477794 0.100492
C -1.771676 -1.662509 -0.923881
H -2.482884 -2.364775 -0.499335
H -2.099414 -1.298205 -1.890407
H -0.780080 -2.103706 -0.975872
C 2.892745 -1.275554 -0.074674
H 2.843881 -2.024146 0.716859
H 3.244430 -1.786778 -0.971123
C 3.890758 -0.178029 0.323671
H 3.959695 0.578723 -0.454448
H 3.577244 0.301702 1.247418
H 4.878023 -0.608372 0.474996

TS-5$_{1r}$

SCF (M052X-D3/def2-TZVP) Energy = -877.311092301
Enthalpy 0K = -877.112513
Enthalpy 298K = -877.111569
Free Energy 298K = -877.165627
Lowest Frequency = -510.7510 cm^{-1}
Second Frequency = 31.8365 cm^{-1}
SCF (M052X-D3,MeCN/def2-QZVPP) Energy = -877.384738188
SCF (BP86-D3,MeCN/def2-QZVPP) Energy = -877.529551019
SCF (M06L,MeCN/def2-QZVPP) Energy = 877.378681041
SCF (PBE0-D3,MeCN/def2-QZVPP) Energy = -876.771332315
SCF (PBE-D3,MeCN/def2-QZVPP) Energy = -876.719266510
SCF (PW6B95-D3,MeCN/def2-QZVPP) Energy = -878.359822688
SCF (TPSSh-D3,MeCN/def2-QZVPP) Energy = -877.510833369
SCF (TPSS-D3,MeCN/def2-QZVPP) Energy -877.574142189
SCF (wB97XD,MeCN/def2-QZVPP) Energy = -877.283642188

C 1.946556 0.093450 -0.061980
C 0.763153 -0.233848 -0.188678
N -0.422658 0.429118 -0.353081
C -0.257662 1.861048 0.080128
H -0.982539 2.457083 -0.466589
H -0.462916 1.935761 1.146984
C 1.155007 2.232945 -0.214552
H 1.723998 2.752985 0.537165
H 1.443329 2.378323 -1.243176
S -1.823613 -0.319809 0.145013
O -2.858776 0.651231 -0.062492
O -1.660712 -0.912921 1.439534
C -1.960266 -1.611818 -1.040765
H -2.076016 -1.167139 -2.022128
H -1.067439 -2.225715 -0.978118
H -2.838715 -2.184189 -0.757832
C 3.399942 -0.026836 0.055064
H 3.875720 0.466057 -0.792958
H 3.737163 0.484073 0.956358
C 3.817385 -1.499626 0.104854
H 3.352629 -1.993659 0.953604
H 3.504703 -2.010813 -0.801618
H 4.897704 -1.578012 0.198897

5P$_{1r}$

SCF (M052X-D3/def2-TZVP) Energy = -877.370336672
Enthalpy 0K = -877.168067
Enthalpy 298K = -877.167123
Free Energy 298K = -877.219339
Lowest Frequency = 33.0297 cm^{-1}
Second Frequency = 61.9906 cm^{-1}
SCF (M052X-D3,MeCN/def2-QZVPP) Energy = -877.442281864
SCF (BP86-D3,MeCN/def2-QZVPP) Energy = -877.581190935
SCF (M06L,MeCN/def2-QZVPP) Energy = 877.431940138
SCF (PBE0-D3,MeCN/def2-QZVPP) Energy = -876.831383169
SCF (PBE-D3,MeCN/def2-QZVPP) Energy = -876.772926126
SCF (PW6B95-D3,MeCN/def2-QZVPP) Energy = -878.416232020
SCF (TPSSh-D3,MeCN/def2-QZVPP) Energy = -877.564569213

SCF (TPSS-D3,MeCN/def2-QZVPP) Energy -877.625105026
SCF (wB97XD,MeCN/def2-QZVPP) Energy = -877.343503571

C 1.788787 0.365416 -0.001678
C 0.779088 -0.358437 -0.449739
N -0.407059 0.307253 -0.717175
C -0.078214 1.763681 -0.532998
H 0.060155 2.189154 -1.522097
H -0.906171 2.264878 -0.043390
C 1.237123 1.762823 0.268146
H 1.077451 1.922957 1.334631
H 1.919912 2.529706 -0.092690
S -1.741016 -0.212814 0.162169
O -2.833301 0.625913 -0.237035
O -1.416637 -0.322970 1.556554
C -1.955270 -1.828682 -0.497413
H -2.153634 -1.742859 -1.559317
H -1.056234 -2.402873 -0.298186
H -2.805494 -2.250155 0.030735
C 3.201958 -0.029922 0.238860
H 3.853997 0.617795 -0.351742
H 3.445520 0.174925 1.284003
C 3.471203 -1.491009 -0.090108
H 2.848099 -2.137512 0.524177
H 3.240083 -1.693984 -1.134149
H 4.513444 -1.744548 0.086972

1a

SCF (M052X-D3/def2-TZVP) Energy = -1327.55076591
Enthalpy 0K = -1327.318165
Enthalpy 298K = -1327.317221
Free Energy 298K = -1327.384520
Lowest Frequency = 11.7321 cm^{-1}
Second Frequency = 16.1116 cm^{-1}
SCF (M052X-D3,MeCN/def2-QZVPP) Energy = -1327.63560567
SCF (BP86-D3,MeCN/def2-QZVPP) Energy = -1327.97260894
SCF (M06L,MeCN/def2-QZVPP) Energy = 1327.82321909
SCF (PBE0-D3,MeCN/def2-QZVPP) Energy = -1326.90973549
SCF (PBE-D3,MeCN/def2-QZVPP) Energy = -1326.82548342
SCF (PW6B95-D3,MeCN/def2-QZVPP) Energy = -1329.05555556
SCF (TPSSh-D3,MeCN/def2-QZVPP) Energy = -1327.73213598
SCF (TPSS-D3,MeCN/def2-QZVPP) Energy -1327.79318605
SCF (wB97XD,MeCN/def2-QZVPP) Energy = -1327.59987384

C 4.593288 -0.003137 -0.339632
C 3.407484 -0.707744 -0.127067
C 5.812721 -0.640509 -0.195198
H 6.725803 -0.088350 -0.357964
C 3.463495 -2.056687 0.224240
C 5.864029 -1.983650 0.148805
H 2.545104 -2.598807 0.389052
H 6.817026 -2.478943 0.255144
C 4.687360 -2.689045 0.354218
H 4.722757 -3.734433 0.620743
H 4.543647 1.038328 -0.617790
C 2.150408 -0.043957 -0.266787
C 1.111354 0.542563 -0.411295

N -0.052756 1.201964 -0.550200
C -1.284871 0.441700 -0.814622
H -2.017238 1.123256 -1.230732
H -1.031305 -0.306614 -1.559422
C -1.800253 -0.206217 0.452639
H -1.052036 -0.837792 0.916695
H -2.182389 0.525492 1.153349
S -0.108897 2.779124 0.004042
O 0.976133 3.489009 -0.590065
O -1.466972 3.192049 -0.181648
C 0.228484 2.641185 1.729855
H -0.580457 2.097940 2.205385
H 1.179523 2.131540 1.848686
H 0.288609 3.657080 2.108646
I -3.458900 -1.483470 -0.005704

8
SCF (M052X-D3/def2-TZVP) Energy = -802.164672390
Enthalpy 0K = -801.904453
Enthalpy 298K = -801.903509
Free Energy 298K = -801.964782
Lowest Frequency = 17.5002 cm^{-1}
Second Frequency = 19.3622 cm^{-1}
SCF (M052X-D3,MeCN/def2-QZVPP) Energy = -802.217526558
SCF (BP86-D3,MeCN/def2-QZVPP) Energy = -802.470639825
SCF (M06L,MeCN/def2-QZVPP) Energy = 802.401464980
SCF (PBE0-D3,MeCN/def2-QZVPP) Energy = -801.719390141
SCF (PBE-D3,MeCN/def2-QZVPP) Energy = -801.637197808
SCF (PW6B95-D3,MeCN/def2-QZVPP) Energy = -803.162358750
SCF (TPSSh-D3,MeCN/def2-QZVPP) Energy = -802.281359858
SCF (TPSS-D3,MeCN/def2-QZVPP) Energy -802.314775421
SCF (wB97XD,MeCN/def2-QZVPP) Energy = -802.208199881

C -3.172078 -1.400536 0.522335
C -3.207333 -0.092378 0.038707
C -4.309069 -2.188228 0.481435
H -4.272760 -3.199370 0.857545
C -4.398480 0.411194 -0.484234
C -5.490874 -1.682099 -0.039839
H -4.422272 1.423374 -0.857272
H -6.376345 -2.298585 -0.070173
C -5.531963 -0.381761 -0.521525
H -6.449689 0.015876 -0.927582
H -2.249562 -1.786904 0.927686
C -2.031510 0.722074 0.077519
C -1.040056 1.401310 0.110366
C 0.179409 2.215262 0.155817
C 1.390805 1.303967 0.450192
H 2.279773 1.931347 0.520632
H 1.239516 0.835991 1.421584
C 1.597724 0.234392 -0.598090
H 1.899799 0.629461 -1.558661
H 0.731303 -0.405311 -0.709194
C 0.355425 2.957466 -1.174404
H -0.458201 3.665526 -1.314209
H 1.297561 3.505306 -1.164742
H 0.352312 2.271968 -2.017658

C 0.065286 3.237261 1.293479
H -0.793960 3.884534 1.134105
H -0.052299 2.733552 2.250134
H 0.965898 3.849561 1.325119
I 3.205004 -1.084601 -0.031847

1r
SCF (M052X-D3/def2-TZVP) Energy = -1175.07414260
Enthalpy 0K = -1174.868290
Enthalpy 298K = -1174.867346
Free Energy 298K = -1174.929867
Lowest Frequency = 12.8060 cm^{-1}
Second Frequency = 29.7464 cm^{-1}
SCF (M052X-D3,MeCN/def2-QZVPP) Energy = -1175.14828672
SCF (BP86-D3,MeCN/def2-QZVPP) Energy = -1175.47029010
SCF (M06L,MeCN/def2-QZVPP) Energy = 1175.35341334
SCF (PBE0-D3,MeCN/def2-QZVPP) Energy = -1174.59206035
SCF (PBE-D3,MeCN/def2-QZVPP) Energy = -1174.51134079
SCF (PW6B95-D3,MeCN/def2-QZVPP) Energy = -1176.35314378
SCF (TPSSh-D3,MeCN/def2-QZVPP) Energy = -1175.22334055
SCF (TPSS-D3,MeCN/def2-QZVPP) Energy -1175.26487703
SCF (wB97XD,MeCN/def2-QZVPP) Energy = -1175.17145382

C -2.543285 1.626642 0.240016
C -1.882940 0.683556 -0.098707
N -1.128787 -0.376289 -0.458913
C 0.293278 -0.171568 -0.773664
H 0.633900 -1.014639 -1.362831
H 0.348341 0.729625 -1.377025
C 1.110608 -0.026268 0.492230
H 1.178785 -0.959067 1.037844
H 0.738012 0.770986 1.124151
S -1.728066 -1.895637 -0.121466
O -3.045559 -1.980769 -0.661989
O -0.695415 -2.804391 -0.520238
C -1.875885 -1.923108 1.636430
H -2.350077 -2.867239 1.886885
H -0.886179 -1.858580 2.074616
H -2.502432 -1.088782 1.935310
C -3.396799 2.767812 0.562916
H -3.845944 2.609592 1.543029
H -2.766458 3.652499 0.650465
C -4.486130 2.997502 -0.485970
H -4.040643 3.190369 -1.458198
H -5.119553 2.118710 -0.571957
H -5.103641 3.848675 -0.208397
I 3.129833 0.497848 -0.001882

TS-P_8
SCF (M052X-D3/def2-TZVP) Energy = -875.547146843
Enthalpy 0K = -875.016882
Enthalpy 298K = -875.015938
Free Energy 298K = -875.098677
Lowest Frequency = -1300.6236 cm-1
Second Frequency = 14.7937 cm-1
SCF (M052X-D3,MeCN/def2-QZVPP) Energy = -875.637219532

C -0.592638 1.833365 -1.230157
C -0.850811 1.248085 0.015443
C -0.397752 3.198194 -1.343594
H -0.205946 3.631963 -2.313936
C -0.898823 2.079107 1.141543
C -0.446379 4.010964 -0.218188
H -1.089379 1.635402 2.108115
H -0.288627 5.074793 -0.308291
C -0.702036 3.444207 1.023102
H -0.747234 4.069260 1.902677
H -0.549392 1.199517 -2.103586
C -0.952300 -0.187062 0.155293
C -1.963091 -1.016953 0.087472
C -3.454496 -0.819304 -0.216101
C -3.541585 -2.353826 -0.448738
H -4.358204 -2.863911 0.055601
H -3.558549 -2.601414 -1.507292
C -2.128931 -2.525098 0.166727
H -1.418093 -3.119494 -0.399711
H -2.154970 -2.889342 1.192601
C -4.212031 -0.383129 1.031365
H -5.287414 -0.438287 0.857938
H -3.966485 -1.022297 1.878911
H -3.952609 0.642828 1.289403
C -3.791079 0.063044 -1.401703
H -3.556018 1.103945 -1.184584
H -3.223538 -0.238507 -2.280692
H -4.854573 -0.008454 -1.633698
N 2.562347 -0.714550 0.241645
C 2.692464 0.745680 0.235942
H 1.773841 1.198605 0.601947
H 2.792798 1.096356 -0.789351
C 3.879062 1.231490 1.056060
H 3.933076 2.319551 1.037656
H 4.808668 0.829932 0.659489
H 3.787334 0.905184 2.089390
C 1.460546 -1.224489 0.987695
H 0.363990 -0.786183 0.513350
C 1.316431 -2.732392 0.952172
H 1.067017 -3.110358 -0.035806
H 2.240886 -3.208689 1.286006
H 0.517357 -3.024341 1.629076
C 1.425003 -0.721152 2.418921
H 0.499347 -1.047620 2.889257
H 2.268334 -1.131001 2.978286
H 1.464827 0.362432 2.481750
C 2.853298 -1.361478 -1.039462
H 2.861664 -2.430634 -0.847578
C 1.799573 -1.078040 -2.111184
H 0.801697 -1.327683 -1.752972
H 2.005797 -1.663427 -3.006236
H 1.802722 -0.025082 -2.390328
C 4.251731 -1.008683 -1.530474
H 4.339361 0.043358 -1.795151
H 4.990212 -1.237737 -0.765524
H 4.478728 -1.591988 -2.421052

TS-P_{1r}
SCF (M052X-D3/def2-TZVP) Energy = -1248.46615152
Enthalpy 0K = -1247.989374
Enthalpy 298K = -1247.988430
Free Energy 298K = -1248.069273
Lowest Frequency = -1079.2311 cm-1
Second Frequency = 17.0886 cm-1
SCF (M052X-D3,MeCN/def2-QZVPP) Energy = -1248.58097977

C -0.357112 0.159799 -0.199721
C -1.299883 -0.738524 -0.065105
N -2.675554 -0.804647 -0.550435
C -2.873931 -2.152267 0.044773
H -3.632979 -2.174952 0.821292
H -3.106888 -2.891933 -0.712274
C -1.413110 -2.126592 0.545796
H -0.760505 -2.869504 0.100543
H -1.312903 -2.152013 1.627282
S -3.793772 0.365114 -0.281471
O -3.381039 1.545297 -0.979783
O -5.068331 -0.237949 -0.538982
C -3.694167 0.722244 1.449660
H -2.675327 1.017579 1.681571
H -4.383879 1.539871 1.634802
H -3.993314 -0.156478 2.011708
N 3.197209 -0.143797 0.246259
C 3.277034 1.305454 0.061114
H 2.323947 1.758278 0.328300
H 3.420193 1.530478 -0.994481
C 4.399727 1.942101 0.868446
H 4.268744 1.740511 1.928845
H 4.416119 3.020898 0.717018
H 5.361594 1.534620 0.566977
C 2.063668 -0.593833 0.994595
H 1.010607 -0.270177 0.435763
C 1.987790 -2.102229 1.142260
H 2.928146 -2.486759 1.540821
H 1.196114 -2.350465 1.844287
H 1.773309 -2.607183 0.203664
C 1.963753 0.065965 2.360078
H 1.026863 -0.225389 2.831583
H 2.792610 -0.260487 2.990199
H 1.982050 1.150401 2.304760
C 3.575056 -0.933408 -0.929069
H 3.605236 -1.969157 -0.603535
C 2.576692 -0.828115 -2.082706
H 1.565204 -1.055972 -1.749600
H 2.845203 -1.525005 -2.875217
H 2.574548 0.173701 -2.510722
C 4.987564 -0.592924 -1.387226
H 5.058263 0.420586 -1.777886
H 5.688947 -0.697922 -0.562643
H 5.281554 -1.272683 -2.184796
C -0.268876 1.460187 -0.909090
H -1.045363 1.505807 -1.673467
H 0.694410 1.507634 -1.419454
C -0.409352 2.663346 0.024616

H 0.274373 2.587620 0.869006
H -0.197433 3.587736 -0.508475
H -1.424818 2.721642 0.406500

3. Supplementary References

[1] Luo, S.-P.; Mejía, E.; Friedrich, A.; Pazidis, A.; Junge, H.; Surkus, A.-E.; Jackstell, R.; Denurra, S.; Gladiali, S.; Lochbrunner, S.; Beller, M. Photocatalytic Water Reduction with Copper-Based Photosensitizers: A Noble-Metal-Free System. *Angew. Chem. Int. Ed.* **52**, 419-423 (2013).

[2] (a) Michelet, B.; Deldaele, C.; Kajouj, S.; Moucheron, C.; Evano, G. A General Copper Catalyst for Photoredox Transformations of Organic Halides. *Org. Lett.* **19**, 3576-3579 (2017); (b) Baguia, H.; Deldaele, C.; Michelet, B.; Beaudelot, J.; Theunissen, C.; Moucheron, C.; Evano, G. [(DPEPhos)(bcp)Cu]PF_6: A General and Broadly Applicable Copper-Based Photoredox Catalyst. *J. Viz. Exp.*, e59739 (2019).

[3] Zhang, Y.; Hsung, R. P.; Tracey, M. R.; Kurtz, K. C. M.; Vera, E. L. Copper Sulfate-Pentahydrate-1,10-Phenanthroline Catalyzed Amidations of Alkynyl Bromides. Synthesis of Heteroaromatic Amine Substituted Ynamides. *Org. Lett.* **6**, 1151-1154 (2014).

[4] Coste, A.; Karthikeyan, G.; Couty, F.; Evano, G. Copper-Mediated Coupling of 1,1-Dibromo-1-alkenes with Nitrogen Nucleophiles: A General Method for the Synthesis of Ynamides. *Angew. Chem. Int. Ed.* **48**, 4381-4385 (2009).

[5] Topolovčan, N.; Hara, S.; Cisařová, I.; Tošner, Z.; Kotora, M. A Study of Polarization and Directing Effects of Unsymmetrical Alkynes Using Regioselective Pd-Catalyzed Bromoallylation. *Eur. J. Org. Chem.*, 234-240 (2020).

[6] García Ruano, J. L.; Alemán, J.; Marzo, L.; Alvarado, C.; Tortosa, M.; Díaz-Tendero, S.; Fraile, A Expanding the Scope of Arylsulfonylacetylenes as Alkynylating Reagents and Mechanistic Insights in the Formation of Csp^2-Csp and Csp^3-Csp Bonds from Organolithiums. *Chem. Eur. J.* **18**, 8414-8422 (2012).

[7] Mai, W.-P.; Song, G.; Sun, G.-C.; Yang, L.-R.; Yuan, J.-W.; Xiao, Y.-M.; Mao, P.; Qu, L.-B. Cu/Ag-catalyzed double decarboxylative cross-coupling reaction between cinnamic acids and aliphatic acids in aqueous solution. *RSC. Adv.* **3**, 19264-19267 (2013).

[8] Qiu, Z.; Zhu, M.; Zheng, L.; Li, J.; Zou, D.; Wu, Y.; Wu, Y. Regioselective α-benzylation of 3-iodoazetidine via Suzuki cross-coupling. *Tetrahedron Lett.* 60, 1321-1324 (2019).

[9] Hogson, D. M.; Kloesges, J. Lithiation–Electrophilic Substitution of *N*-Thiopivaloylazetidine. *Angew. Chem. Int. Ed.* **49**, 2900-2903 (2010).

[10] Gaussian 16, Revision A.03, Frisch, M. J., Trucks, G. W., Schlegel, H. B., Scuseria, G. E., Robb, M. A., Cheeseman, J. R., Scalmani, G., Barone, V., Petersson, G. A., Nakatsuji, H., Li, X., Caricato, M., Marenich, A. V., Bloino, J., Janesko, B. G., Gomperts, R., Mennucci, B., Hratchian, H. P., Ortiz, J. V., Izmaylov, A. F., Sonnenberg, J. L., Williams-Young, D., Ding, F., Lipparini, F., Egidi, F., Goings, J., Peng, B., Petrone, A., Henderson, T., Ranasinghe, D., Zakrzewski, V. G., Gao, J., Rega, N., Zheng, G., Liang, W., Hada, M., Ehara, M., Toyota, K., Fukuda, R., Hasegawa, J., Ishida, M., Nakajima, T., Honda, Y., Kitao, O., Nakai, H., Vreven, T., Throssell, K., Montgomery, Jr., J. A., Peralta, J. E., Ogliaro, F., Bearpark, M. J., Heyd, J. J., Brothers, E. N., Kudin, K. N., Staroverov, V. N., Keith, T. A., Kobayashi, R., Normand, J., Raghavachari, K., Rendell, A. P., Burant, J. C., Iyengar, S. S., Tomasi, J., Cossi, M., Millam, J. M., Klene, M., Adamo, C., Cammi, R., Ochterski, J. W., Martin, R. L., Morokuma, K., Farkas, O., Foresman, J. B., and D. J. Fox. (2016). Gaussian, Inc., Wallingford CT.

[11] Zhao, Y.; Schultz, N. E.; Truhlar, D. G. Design of Density Functionals by Combining the Method of Constraint Satisfaction with Parametrization for Thermochemistry, Thermochemical Kinetics, and Noncovalent Interactions. *J. Chem. Theory Comput.* **2**, 364-382 (2006).

[12] Weigend, F.; Ahlrichs, R. Balanced basis sets of split valence, triple zeta valence and quadruple zeta valence quality for H to Rn: Design and assessment of accuracy. *Phys. Chem. Chem. Phys.* **7**, 3297-3305 (2005).

[13] Weigend, F.; Furche, F.; Ahlrichs, R. Gaussian basis sets of quadruple zeta valence quality for atoms H–Kr. *J. Chem. Phys.* **119**, 12753-12762 (2003).

[14] Peterson, K. A.; Figgen, D.; Goll, E.; Stoll, H.; Dolg, M. Systematically convergent basis sets with relativistic pseudopotentials. II. Small-core pseudopotentials and correlation consistent basis sets for the post-d group 16–18 elements. *J. Chem. Phys.* **119**, 11113-11123 (2003).

[15] Goerigk, L.; Hansen, A.; Bauer, C.; Ehrlich, S.; Najibi, A.; Grimme, S. A look at the density functional theory zoo with the advanced GMTKN55 database for general main group thermochemistry, kinetics and noncovalent interactions. *Phys. Chem. Chem. Phys.* **19**, 32184-32215 (2017).

[16] Alabugin, I. V.; Gilmore, K.; Manoharan, M. Rules for Anionic and Radical Ring Closure of Alkynes. *J. Am. Chem. Soc.* **133**, 12608-12623 (2011).

[17] Cancès, E.; Mennucci, B. New applications of integral equations methods for solvation continuum models: ionic solutions and liquid crystals. *J. Math. Chem.* **23**, 309-326 (1998).

[18] Cancès, E.; Mennucci, B.; Tomasi, J. A new integral equation formalism for the polarizable continuum model: Theoretical background and applications to isotropic and anisotropic dielectrics. *J. Chem. Phys.* **107**, 3032-3041 (1997).

[19] Mennucci, B.; Cancès, E.; Tomasi, J. Evaluation of Solvent Effects in Isotropic and Anisotropic Dielectrics and in Ionic Solutions with a Unified Integral Equation Method: Theoretical Bases, Computational Implementation, and Numerical Applications. *J. Phys. Chem. B* **101**, 10506-10517 (1997).

[20] Grimme, S.; Antony, J.; Ehrlich, S.; Krieg, H. A consistent and accurate ab initio parametrization of density functional dispersion correction (DFT-D) for the 94 elements H-Pu. *J. Chem. Phys.* **132**, 154104 (2010).

[21] Pracht, P.; Bohle, F.; Grimme, S. Automated exploration of the low-energy chemical space with fast quantum chemical methods. *Phys. Chem. Chem. Phys.* **22**, 7169-7192 (2020).

[22] Grimme, S. Exploration of Chemical Compound, Conformer, and Reaction Space with Meta-Dynamics Simulations Based on Tight-Binding Quantum Chemical Calculations. *J. Chem. Theory Comput.* **15**, 2847-2862 (2019).

[23] Bannwarth, C.; Caldeweyher, E.; Ehlert, S.; Hansen, A.; Pracht, P.; Seibert, J.; Spicher, S.; Grimme, S. Extended tight-binding quantum chemistry methods. *Wiley Interdiscip. Rev. Comput. Mol. Sci.* **11**, e1493 (2020).

[24] Bannwarth, C.; Ehlert, S.; Grimme, S. GFN2-xTB—An Accurate and Broadly Parametrized Self-Consistent Tight-Binding Quantum Chemical Method with Multipole Electrostatics and Density-Dependent Dispersion Contributions. *J. Chem. Theory Comput.* **15**, 1652-1671 (2019).

[25] Becke, A. D. Density-functional exchange-energy approximation with correct asymptotic behavior. *Phys. Rev. A* **38**, 3098-3100 (1988).

[26] Perdew, J. P. Density-functional approximation for the correlation energy of the inhomogeneous electron gas. *Phys. Rev. B* **33**, 8822-8824 (1986).

[27] Zhao, Y.; Truhlar, D. G. The M06 suite of density functionals for main group thermochemistry, thermochemical kinetics, noncovalent interactions, excited states, and transition elements: two new functionals and systematic testing of four M06-class functionals and 12 other functionals. *Theor. Chem. Acc.* **120**, 215-241 (2008).

[28] Perdew, J. P.; Burke, K.; Ernzerhof, M. Generalized Gradient Approximation Made Simple. *Phys. Rev. Lett.* **77**, 3865-3868 (1996).

[29] Adamo, C.; Barone, V. Toward reliable density functional methods without adjustable parameters: The PBE0 model. *J. Chem. Phys.* **110**, 6158-6170 (1999).

[30] Tao, J.; Perdew, J. P.; Staroverov, V. N.; Scuseria, G. E. Climbing the Density Functional Ladder: Nonempirical Meta--Generalized Gradient Approximation Designed for Molecules and Solids. *Phys. Rev. Lett.* **91**, 146401 (2003).

[31] Chai, J.-D.; Head-Gordon, M. Long-range corrected hybrid density functionals with damped atom-atom dispersion corrections. *Phys. Chem. Chem. Phys.* **10**, 6615-6620 (2008).

[32] Lu, T.; Chen, F. Multiwfn: A multifunctional wavefunction analyzer. *J. Comput. Chem.* **33**, 580-592 (2012).

[33] Marenich, A. V.; Jerome, S. V.; Cramer, C. J.; Truhlar, D. G. Charge Model 5: An Extension of Hirshfeld Population Analysis for the Accurate Description of Molecular Interactions in Gaseous and Condensed Phases. *J. Chem. Theory Comput.* **8**, 527-541 (2012).

[34] Hirshfeld, F. L. Bonded-atom fragments for describing molecular charge densities. *Theor. Chim. Acta* **44**, 129-138 (1977).

[35] Becke, A. D. A multicenter numerical integration scheme for polyatomic molecules. *J. Chem. Phys.* **88**, 2547-2553 (1988).

[36] Bader, R. F. W. A quantum theory of molecular structure and its applications. *Chem. Rev.* **91**, 893-928 (1991).

[37] Reed, A. E.; Weinstock, R. B.; Weinhold, F. Natural population analysis. *J. Chem. Phys.* **83**, 735-746 (1985).

[38] NBO Version 3.1, Glendening, E. D.; Reed, A. E.; Carpenter, J. E.; Weinhold, F.

[39] Becke, A. D.; Edgecombe, K. E. A simple measure of electron localization in atomic and molecular systems. *J. Chem. Phys.* **92**, 5397-5403 (1990).

[40] Savin, A.; Jepsen, O.; Flad, J.; Andersen, O. K.; Preuss, H.; von Schnering, H. G. Electron Localization in Solid-State Structures of the Elements: the Diamond Structure. *Angew. Chem. Int. Ed.* **31**, 187-188 (1992).

[41] Raub, S.; Jansen, G. A quantitative measure of bond polarity from the electron localization function and the theory of atoms in molecules. *Theor. Chem. Acc.* **106**, 223-232 (2001).
